# The influence of perceived stress of Chinese healthcare workers after the opening of COVID-19: the bidirectional mediation between mental health and job burnout

**DOI:** 10.3389/fpubh.2023.1252103

**Published:** 2023-08-17

**Authors:** Minhui Jiang, Zhangjie Li, Xiaomin Zheng, Min Liu, Yaling Feng

**Affiliations:** ^1^Department of Psychology, Wuxi Maternity and Child Health Care Hospital, Wuxi, Jiangsu, China; ^2^The First Clinical Medical College, Fujian Medical University, Fuzhou, Fujian, China

**Keywords:** healthcare workers, perceived stress, mental health, job burnout, the bidirectional mediation

## Abstract

**Objective:**

To explore the current status and interaction of perceived stress, job burnout and mental health among healthcare workers after the opening of COVID-19 which occurred in December 2022.

**Methods:**

A cross-sectional study of 792 healthcare workers from three tertiary hospitals in Wuxi was conducted from January 2023 to February 2023. Sociodemographic questionnaire, Perceived Stress Scale, Burnout Scale and Mental Health Self-Assessment Questionnaire were used for investigation. SPSS 26.0 was used to conduct data analysis. The significance of mediation was determined by the PROCESS macro using a bootstrap method.

**Results:**

The results showed that (1) The average scores of the participants for perceived stress, mental health and job burnout were 22.65 (7.67), 3.85 (4.21) and 1.88 (1.03), respectively. (2) The perceived stress score, mental health score and job burnout score of healthcare workers were positively correlated (*r* = 0.543–0.699, *p* < 0.05). (3) Mental health partially mediated the relationship between perceived stress and job burnout with a mediating effect of 17.17% of the total effect. Job burnout partially mediated the correlation between perceived stress and mental health with a mediating effect of 31.73% of the total effect.

**Conclusion:**

The results of this study suggested that perceived stress had an impact on job burnout and mental health, either directly or indirectly. Healthcare managers should intervene to reduce perceived stress to protect healthcare workers’ mental health, thereby alleviating burnout under the opening COVID-19 pandemic environment.

## 1. Introduction

The COVID-19 pandemic has caused a crisis in the physical and mental health of healthcare workers (1), leading to symptoms such as fear, depression, anxiety, and stress (2–4). A recent study in Spain showed a significant increase in the prevalence of physical and mental health problems among healthcare workers due to the COVID-19 pandemic, especially depression at 30.2%, anxiety at 46.5% and a high level of burnout reaching 51% (5). A survey of healthcare workers in Italy during the COVID-19 pandemic found that 31% of participants suffered from burnout, 12% participants experienced anxiety, and 7% participants experienced depression (6). In China, the prevalence of mental and emotional disorders among healthcare workers during the COVID-19 pandemic ranged from 48 to 63% (7, 8). World Health Organization (WHO) emphasizes that the COVID-19 pandemic is a long-term stressful event and stressor. In December 2022, China fully opened up its epidemic prevention and control measures. As China has been the country with the longest anti-epidemic period and the latest overall opening, the stress levels of Chinese healthcare workers are higher than those of other countries. Therefore, it is important to focus on the changes in stress levels and burnout among healthcare workers after the opening of epidemic prevention and control measures, as well as how to protect their physical and mental health.

Perceived stress refers to a psychological reaction that results from individuals’ cognitive evaluation of internal or external pressure events and threatening stimulus. The presence and intensity of stress are subjective to the individual (9). Healthcare workers are considered to experience higher levels of stress compared to other professions according to the nature of their work, such as high risk, high workload, and high levels of mental stress (10). The sources of stress for healthcare workers mainly include complex working environment, disproportion between income and effort, high pressure for promotion, conflicts between work and family, escalating doctor-patient conflicts, complicated interpersonal relationship (11). Since the outbreak of the COVID-19 pandemic, the stress level of healthcare workers had dramatically increased. During the early stage of the comprehensive opening of epidemic prevention and control in December 2022, external pressures on healthcare workers increased sharply due to events such as shortage of medical resources, surge in severe cases, and increased work intensity, as well as the stimulation of anxiety, tension, and sleep deprivation, which further increased perceived stress. High levels of stress tend to poor work performance, negative emotions, low occupational efficacy and job satisfaction, which seriously affects the provision of good healthcare services, sows the potential for doctor-patient conflict, and also affects the sustainable and healthy development of the medical talent team (12, 13). Therefore, attention to the stress level of Chinese healthcare workers and its impact on individuals and society is one of the current focal issues of social concern.

Job burnout refers to a state of persistent dysfunction that occurs after a long-term exposure to chronic stress and is a progressive psychological response (14). In 1981, Maslach and Jackson proposed a definition of job burnout that included three aspects: emotional exhaustion, depersonalization, and low personal accomplishment. Adverse job-related exposures, such as complex work environments, prominent personal and social conflicts, and unmet personal needs, often increase external pressure and lead to job burnout (15). The Conservation of Resources Theory (COR) emphasizes that people have the ability to seek, protect and renew personal resources to adapt to and regulate stress, and stress is a comprehensive stress response that is overloaded and subjectively perceived when personal resources are unstable, threatened, or even disappear (16). Based on this theory, Crawford proposed that stress increased when work demands were out of bounds, resources which needed for work were deprived, resources for routine tasks were forced to decrease, and burnout occurred when resource consumption continued to exist (17). The risk of work-related stress among young physicians is twice that of other physicians. Job burnout occurs early in young physicians’ career possibly due to their high workload and continuous consumption of work resources (18).

Mental health problems are typically described as negative emotions such as depression, anxiety, and panic. Perceived stress leads to poorer mental health (19). Manning et al. confirmed that perceived stress was significantly associated with symptoms of anxiety under the COVID-19 pandemic, additionally, specific stressors could significantly predict symptoms of anxiety, depression, and panic (20). The stress-cognitive vulnerability theory suggested that stress initiated negative cognitive-emotional processing by activating cognitive biases, which exacerbated negative emotions and behaviors (21). Shen et al. proposed that the relationship between stress and mental health depended on cognitive function (22). When cognitive resources to deal with stressors are depleted, physical and mental fatigue emerge. This led to impaired physical and/or cognitive function, and sustained stress usually results in severe fatigue, which in turn leads to mental health problems.

Previous research before the COVID-19 pandemic found that emotional disorders and stress could lead to occupational burnout (23). During the outbreak of COVID-19, it had also been observed that experiencing perceived pressure could lead to occupational burnout and fatigue, and it could significantly predict symptoms of anxiety, depression, and panic (20, 24, 25). Studies had shown that healthcare workers who experienced burnout may have suicidal tendencies, which possibly was related to depression (26, 27). Han et al. suggested that burnout could cause severe psychological damage among healthcare workers, which manifested as a feeling of exhaustion after the end of a shift. This study confirmed burnout significantly predicts mental health (28). Therefore, the relationship between perceived stress, mental health, and burnout has been confirmed both before and during the pandemic. Additionally, it is hypothesized that the relationship between job burnout and mental health is not only one-way, but also influenced by perceived stress in a two-way manner.

Although the relationships of stress-burnout, stress-mental health, and burnout-mental health have been discussed in different time, the mechanisms of their interactions have not been specifically clarified, the changes and associations among the three facets after the opening of the COVID-19 pandemic in China may have new findings, and the relationship between burnout and mental health still need further research and exploration. In summary, this study aimed to explore the interrelationships and interact mechanism among perceived stress, job burnout and mental health of clinical healthcare workers in China after the opening of the COVID-19 epidemic, hoping to provide empirical evidences and new theoretical references for healthcare managers to take targeted interventions to improve the mental health and reduce the burnout of healthcare workers.

The following hypotheses were developed based on the literature reviewed above.

*H1*: Perceived stress, mental health, and job burnout are correlated.

*H2*: Mental health can mediate the relationship between perceived stress and job burnout.

*H3*: Job burnout can mediate the relationship between perceived stress and mental health.

**Figure 1:**
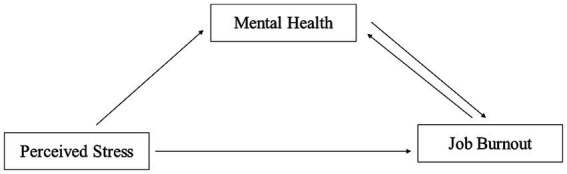
The hypothesized model.

The hypothetical model is shown in Figure 1.

## 2. Methods

### 2.1. Participants

A convenient sample of 792 healthcare workers from three tertiary hospitals in Wuxi City were surveyed from January 2023 to February 2023. The inclusion criteria were possession of a nurse’s license/physician’s qualification certificate, in-service nurses/physicians, and engaged in clinical work for ≥1 year. The exclusion criteria were intern nurses/physicians and nurses/physicians in further education or training. The study was approved by the Ethics Committees of Wuxi Maternity and Child Health Care Hospital, and all participants were informed consent (No 2023-01-0721-33).

### 2.2. Measuring scale

#### 2.2.1. Sociodemographic characteristics

The demographic questionnaire included age, gender, marital status, education level, job title, employment status, monthly salary level, and whether they had been front-line anti-epidemic workers.

#### 2.2.2. Perceived stress

The Perceived Stress Scale (PSS), developed by COHEN et al. (13) in 1983 and introduced and culturally adapted by YANG Tingzhong (29), was used to assess perceived stress levels. The scale consists of 14 items, including two dimensions of tension and loss of control, using a 5-point Likert scoring method. The total score ranges from 14 to 70, and the higher the score, the greater the perceived stress. In this study, Cronbach’s α of this entire scale was 0.845, and Cronbach’s α for two subscales were 0.826 and 0.886, respectively.

#### 2.2.3. Job burnout

The Maslach Burnout Inventory-General Survey (MBI-GS), compiled by MASLACH (30), and translated and revised by Chinese scholars, LI Chaoping (31), was used to assess job burnout. The scale includes three dimensions of emotional exhaustion, depersonalization, and low personal accomplishment, with a total of 15 items. The scale uses a 7-point Likert scoring method, ranging from 0 (never) to 6 (every day). The score of job burnout is calculated as follows: emotional exhaustion dimension score × 40% + depersonalization dimension score × 30% + low personal accomplishment dimension score × 30% (32). In this study, Cronbach’s α for three subscales in this study were 0.95, 0.93, and 0.95, respectively.

#### 2.2.4. Mental health

The Mental Health Self-Reporting Questionnaire-20 (SRQ-20) (33), published by the World Health Organization, was used to assess mental health. It consists of 20 items, each with a 2-point score. The answers are “yes” (1 point) or “no” (0 point). The highest score is 20 points, with a higher score indicating more prominent symptoms of mental disorders. A score of 7 or higher is generally accepted as emotional distress and need for professional help.

### 2.3. Data analysis

SPSS 26.0 was used to conduct descriptive statistical analysis, t-tests or analysis of variance, correlation analysis, and linear regression analysis. We used the method proposed by Wen Zhonglin and Ye Baojuan (34) to test the mediation effect, if the path coefficient from the predictor variable to the outcome variable is significant in both the direct effect model and the mediation effect model, but is reduced in the mediation effect model, then the mediator plays a partial mediating role, if the path coefficient from the predictor variable to the outcome variable is significant in the direct effect model, but not significant in the mediation effect model, then the mediator plays a complete mediating role. Model 4 in the PROCESS macro for SPSS developed by Hayes was used to identify the mediation effects. We used bootstrapping methods to test whether the mediating effect was statistically significant and performed 5,000 bootstrap resamples to determine the 95% confidence intervals of the various effects. The significance of mediating effects depends on whether the 95% confidence intervals include zero, and if the confidence interval does not contain 0, then the mediating effect is significant.

## 3. Results

### 3.1. Comparison of perceived stress, mental health, and job burnout scores among healthcare workers with different sociodemographic and work-related characteristics

Socio-demographic and job-related characteristics were shown in Table 1. Among the 792 healthcare workers surveyed, 96 (12.1%) were male and 696 (87.9%) were female, 79 (10%) had a junior college degree or below, 510 (64.4%) had a bachelor’s degree, 184 (23.2%) had a master’s degree, and 19 (2.4%) had a doctor’s degree, and 377 (47.6%) were at a junior level or below, 242 (30.6%) were at an intermediate level, 131 (16.5%) were associate senior, and 42 (5.3%) were positive senior. Among the participants, married healthcare workers were the majority at 78, and 60.5% of the participants were permanent employment. Healthcare workers who earned 6,000-10000RMB were the majority at 58.2%.

**Table 1:** Differences in perceived stress, mental health, and job burnout scores among healthcare workers on socio-demographic factors and job-related factors.

Demographic variables	*N* (%)	Perceived stress (M ± SD)	*t/F*	Mental health (M ± SD)	*t/F*	Job burnout (M ± SD)	*t/F*
Age(years old)			2.884^*^		1.700		3.486^*^
<26	144(18.2)	22.36 ± 7.26		3.53 ± 4.00		1.86 ± 0.92	
26–35	249 (31.5)	23.04 ± 7.53		3.80 ± 4.23		1.95 ± 0.98^a^	
36–45	127 (16.0)	24.05 ± 7.76^a^		4.59 ± 4.94		2.05 ± 1.20^a^	
>45	272 (34.3)	21.78 ± 7.87		3.71 ± 3.91		1.73 ± 1.02	
Gender			0.002		2.958		0.600
Male	96 (12.1)	22.61 ± 6.68		3.16 ± 4.12		1.80 ± 0.95	
Female	696 (87.9)	22.65 ± 7.80		3.94 ± 4.22		1.89 ± 1.04	
Marital status			1.560		1.012		5.090^*^
Married	618 (78)	22.47 ± 7.68		3.77 ± 4.13		1.83 ± 1.02	
Unmarried	174 (22)	23.29 ± 7.61		4.13 ± 4.49		2.03 ± 1.04	
Education level			3.430^*^		3.332^*^		4.647^**^
College and below	79 (10)	21.76 ± 7.91^b^		2.78 ± 3.78^b^		1.57 ± 0.98^bc^	
Bachelor’s degree	510 (64.4)	22.23 ± 7.68^b^		3.77 ± 4.16^b^		1.85 ± 1.04^b^	
Master’s degree	184 (23.2)	24.21 ± 7.57		4.52 ± 4.47		2.07 ± 0.99	
Doctor’s degree	19 (2.4)	22.32 ± 5.03		3.74 ± 3.89		1.83 ± 0.83	
Job Title			3.557^*^		1.470		5.316^**^
Junior and below	377 (47.6)	22.92 ± 7.74^d^		3.69 ± 4.40		1.93 ± 1.05^d^	
Intermediate	242 (30.6)	22.65 ± 7.06^d^		3.96 ± 4.00		1.92 ± 0.94^d^	
Associate senior	131 (16.5)	23.04 ± 8.15^d^		4.36 ± 4.34		1.84 ± 1.11^d^	
Positive senior	42 (5.3)	18.95 ± 8.06		2.98 ± 3.08		1.28 ± 0.85	
Employment			2.834		6.984^**^		1.019
Permanent	479 (60.5)	23.02 ± 7.76		4.17 ± 4.24		1.91 ± 1.04	
Contract	313 (39.5)	22.08 ± 7.49		3.36 ± 4.13		1.83 ± 1.01	
Monthly salary (RMB, yuan)			2.831^*^		1.099		5.227^**^
<3,000	6 (0.8)	23.33 ± 4.46		2.00 ± 2.45		1.99 ± 0.77	
3,000–6,000	206 (26)	23.26 ± 7.57^e^		3.98 ± 4.38		1.93 ± 1.05^e^	
6,000–10,000	461 (58.2)	22.84 ± 7.75^e^		3.94 ± 4.33		1.94 ± 1.01^e^	
>10,000	119 (15)	20.82 ± 7.45		3.34 ± 3.45		1.54 ± 1.01	
Whether they had been front-line anti-epidemic workers			2.938		5.409^*^		0.009
Yes	197 (24.9)	23.46 ± 7.36		4.45 ± 4.42		1.87 ± 0.97	
No	595 (75.1)	22.38 ± 7.75		3.65 ± 4.13		1.88 ± 1.05	

*p < 0.05, **p < 0.01, ***p < 0.001, ^a^compared with >45 *p* < 0.05, ^b^compared with Master’s degree *p* < 0.05, ^c^compared with Bachelor’s degree *p* < 0.05, ^d^compared with Positive senior *p* < 0.05, ^e^compared with >10,000 *p* < 0.05.

#### 3.1.1. Sex, marital status, and employment

According to the results shown in Table 1, unmarried healthcare workers (*p* = 0.024) reported significantly higher scores of job burnout than married ones (*p*<0.05), healthcare workers with permanent employment (*p* = 0.008) reported significantly higher scores of mental health than the contract ones (*p*<0.01). Gender differences in the scores of perceived stress, mental health, and job burnout were not statistically significant (*p* > 0.05).

#### 3.1.2. Age, job title, monthly salary, education, and other factors

Table 1 also displayed healthcare workers aged 36–45 (*p* = 0.006) reported significantly higher scores of perceived stress than those aged over 45 (*p*<0.05), while healthcare workers aged 26–35 (*p* = 0.014) and aged 36–45 (*p* = 0.004) had significantly higher scores of job burnout than those with aged over 45 (*p*<0.05). Healthcare workers with the job title of associate senior and below had significantly higher scores in the perceived stress and job burnout than those with a positive senior job title (average *p*<0.05). Healthcare workers with monthly salaries of 3,000-10000RMB had significantly higher scores in the perceived stress and job burnout than those with monthly salary of 10000RMB or more (average *p*<0.05). Healthcare workers with a master’s degree reported significantly higher scores of perceived stress, mental health, and job burnout than those with a college degree or lower and those with a bachelor’s degree (average *p*<0.05). Furthermore, the result in Table 1 showed that healthcare workers who had worked as front-line anti-epidemic workers (*p* = 0.02) had significantly higher scores in mental health than those who had not (*p*<0.05).

### 3.2. Correlation analysis of variables

The average scores of the participants for perceived stress, mental health and job burnout were 22.65 (7.67), 3.85 (4.21) and 1.88 (1.03), respectively. Correlation analysis(see Table 2) showed that perceived stress score and each dimension score of healthcare workers were positively correlated with mental health score (*r* = 0.332–0.603, *p* < 0.05), perceived stress score and each dimension score were positively correlated with job burnout score and each dimension score (r = 0.194–0.699, *p* < 0.05), mental health score was positively correlated with job burnout score and each dimension score (*r* = 0.212–0.558, *p* < 0.05), perceived stress score, mental health score and job burnout score were positively correlated(*r* = 0.543–0.699, *p* < 0.05).

**Table 2:** Means, standard deviations and correlation coefficients for all variables.

Variables	M ± SD	T	LOC	PS	MH	EX	D	LPA	JB
T	10.72 ± 4.48	1							
LOC	11.92 ± 5.32	0.218^**^	1						
PS	22.65 ± 7.67	0.736^**^	0.821^**^	1					
MH	3.85 ± 4.21	0.603^**^	0.332^**^	0.583^**^	1				
EX	2.09 ± 1.26	0.679^**^	0.281^**^	0.592^**^	0.558^**^	1			
D	1.50 ± 1.21	0.543^**^	0.308^**^	0.531^**^	0.507^**^	0.760^**^	1		
LPA	1.97 ± 1.48	0.194^**^	0.578^**^	0.515^**^	0.212^**^	0.197^**^	0.349^**^	1	
JB	1.88 ± 1.03	0.607^**^	0.495^**^	0.699^**^	0.543^**^	0.841^**^	0.875^**^	0.651^**^	1

*p < 0.05, **p < 0.01, ***p < 0.001; T, tension; LOC, loss of control; PS, perceived stress; MH, mental health; EX, emotional exhaustion; D depersonalization; LPA, low personal accomplishment; JB, job burnout.

### 3.3. Mediation effect analysis

#### 3.3.1. The mediating effect analysis of mental health between perceived stress and job burnout

The results of regression analysis (Table 3) showed that perceived stress had a significant positive predictive effect on job burnout (β = 0.699, *p* < 0.05), perceived stress can also positively predict mental health (β = 0.583, *p* < 0.05), but the effect of perceived stress on job burnout decreased after mental health and perceived stress were entered into the regression equation (β = 0.579, *p* < 0.05), indicating that mental health played a partial mediating role between perceived stress and job burnout. The results of the bootstrapping methods (Table 4) showed that the mediating effect of mental health between perceived stress and and job burnout was significant with a 95% confidence interval of (0.077, 0.162), excluding 0, and the mediating effect accounted for 17.17% of the total effects.

**Table 3:** The mediating effect of job mental health between perceived stress and job burnout.

Regression equation		Fitting index		Coefficient significance	
Outcome variables	Predictive variables	*R* ^2^	F	*β*	t
Job burnout	Perceived stress	0.488	753.100^**^	0.699	27.443^**^
Mental health	Perceived stress	0.340	406.291^**^	0.583	20.157^**^
Job burnout	Perceived stress	0.516	420.498^**^	0.579	18.990^**^
	Mental health			0.206	6.744^**^

**p < 0.01.

**Table 4:** Mediating model examination by bootstrap (mental health as the mediator).

Effect Type	Path	Effect	SE	LL 95% CI	UL 95% CI
Direct effect	Perceived stress → Job burnout	0.579	0.031	0.519	0.639
Indirect Effect	Perceived stress → Mental health → Job burnout	0.120	0.022	0.077	0.162
Total effect		0.699	0.026	0.649	0.749

#### 3.3.2. The mediating effect analysis of job burnout between perceived stress and mental health

Similarly, as shown in Table 5, we found that perceived stress had a significant positive predictive effect on mental health (β = 0.583, *p* < 0.05), but this effect decreased after job burnout and perceived stress was entered into the regression equation (β = 0.398, *p* < 0.05), indicating that job burnout played a partial mediating role between perceived stress and mental health. The Bootstrap test (Table 6) revealed that the mediating effect of job burnout between perceived stress and mental health was significant, with a 95% confidence interval of (0.120, 0.251), excluding 0, and the mediating effect accounted for 31.73% of the total effects.

**Table 5:** The mediating effect of job burnout between perceived stress and and mental health.

Regression equation		Fitting index		Coefficient significance	
Outcome Variables	Predictive variables	*R* ^2^	F	*β*	*t*
Mental health	Perceived stress	0.340	406.291^**^	0.583	20.157^**^
Job burnout	Perceived stress	0.488	753.100^**^	0.699	27.443^**^
Mental health	Perceived stress	0.376	237.329^**^	0.398	10.111^**^
	Job burnout			0.265	6.744^**^

**p < 0.01.

**Table 6:** Mediating model examination by bootstrap (job burnout as the mediator).

Effect Type	Path	Effect	SE	LL 95% CI	UL 95% CI
Direct effect	Perceived stress → Mental health	0.398	0.039	0.320	0.475
Indirect effect	Perceived stress → Job burnout → Mental health	0.185	0.033	0.120	0.251
Total effect		0.583	0.029	0.526	0.640

Therefore, by integrating the two mediation models mentioned above, this study tested a bidirectional mediation model with perceived stress as the independent variable, mental health and job burnout as the mutually mediating variables and the outcome variable.

## 4. Discussion

This study focuses on healthcare workers in the context of the opening and outbreak of COVID-19. This study found that the scores of perceived stress, mental health and job burnout were significantly positively correlated, supporting hypothesis 1. Our findings corroborated our hypothesis 2 and 3 that mental health could mediate the relationship between perceived stress and job burnout, and job burnout could mediate the relationship between perceived stress and mental health. In the mediated model, mental health and job burnout acted as each other’s mediators.

### 4.1. Current status of perceived stress, mental health, and job burnout in healthcare workers

The results of this cross-sectional study showed that the average scores of the participants for perceived stress, mental health and job burnout were 22.65 (7.67), 3.85 (4.21) and 1.88 (1.03), respectively. The level of perceived stress among healthcare workers was significantly higher than that of the general population (35), indicating that the special nature of clinical medical work (high technology and high risk) makes healthcare workers more sensitive to stress. The mental health status of healthcare workers was at a moderate level, mainly due to the increase in symptoms such as depression, anxiety, and insomnia after the outbreak of COVID-19, which led to a decline in their mental health status (36). The level of job burnout among healthcare workers was significantly higher than that of other populations (37), as healthcare workers are vulnerable and prone to job burnout due to the large workload, tedious content, monotonous form, high repetitiveness, and tense doctor-patient relationship. The occurrence of job burnout not only affects their physical and mental health, but also has a negative impact on medical quality, their own career development, and stability (38). The emotional exhaustion dimension had the highest score, which was consistent with the findings of Soler et al. (39), indicating that emotional exhaustion was the main manifestation of job burnout among healthcare workers. This was mainly due to the long-term exposure to the pain and suffering of patients, which can lead to vicarious trauma and empathy fatigue. In addition, the complex doctor-patient relationships exacerbate emotional exhaustion.

Our study also found that young healthcare workers with a master’s degree, permanent employment, low income and associate senior job title generally experienced more perceived stress, mental health problems, and job burnout. These results were consistent with previous studies (40). The concentrated outbreak of COVID-19 also had a certain impact on the mental health status of healthcare workers, especially those who were the main force in medical services and carried heavy workloads and great risks. This group of people was undoubtedly challenged in their ability to withstand stress and fatigue, as well as their ability to control and regulate their emotions. Therefore, healthcare management should focus on young healthcare workers with a master’s degree, low income, long working hours, and those who have participated in epidemic prevention and control work. This could be achieved by strengthening the screening of mental health problems, optimizing the allocation of medical services, improving the healthcare professional environment, enhancing support for healthcare talents and teams. And we suggest that healthcare workers can adopt a task-oriented approach to directly and efficiently address and handle problems (41), believe in one’s own abilities and enhance self-efficacy in order to reduce the stress on healthcare workers, prevent work burnout and mental health problems (42).

### 4.2. The correlation among perceived stress, mental health and job burnout of healthcare workers

In this study, the perceived stress score and each dimension score, mental health score and job burnout score and each dimension score of healthcare workers were positively correlated with each other, which was similar to the previous study (43). This suggested that medical work, as a high-stress profession, involved not only high-risk medical treatment, but also the pressure of career development, such as promotion, disciplinary development, and continuing education. After the outbreak of the epidemic, healthcare workers also had to undertake a large amount of prevention and treatment work, which could easily cause stress due to prolonged working hours, poor temporary working conditions, and long-term tension (44). When the level of perceived stress increases, it could easily induce tension and a sense of loss of control, leading to psychological and physiological dysfunction. This not only resulted in psychological problems such as somatization, anxiety, or terror, but also triggered job burnout and even intentions to leave the profession. Additionally, there was a mutual influence between mental health and job burnout. Healthcare workers with mental health problems tended to have negative emotions, difficulty in mobilizing positive resources and social support under difficult circumstances, and a lack of ability to cope with and solve problems, leading to a gradual loss of work enthusiasm and personal sense of achievement, and job burnout occurred. Healthcare workers experiencing job burnout often appeared tired, passive, and even learned helplessness, which undoubtedly exacerbates negative emotions such as anxiety, depression, and fear. To sum up, job burnout could have a spillover effect, affecting mental health removed from the work situation, correspondingly, workers with improved mental health could attenuate their symptoms of job burnout.

### 4.3. The bidirectional mediating effect of mental health and job burnout

Firstly, it was found that there was a predictive relationship between mental health and job burnout. This suggested that promoting mental health can not only reduce job burnout, but also potentially be a outcome of reducing it. Thus, reducing job burnout was not only a goal or outcome, but also a possible pathway to achieve mental health. This result was an innovative and improved contribution to the existing research on unidirectional effects, proving the interaction between psychological and environmental factors, and a remedy for the deficiency in previous practice of emphasizing mental health over burnout. In particular, improving the job burnout of healthcare workers could explore a new path for screening, assessment, and intervention in their mental health.

Subsequently, this study validated a bidirectional mediating model of mutual mediation between mental health and job burnout under the influence of perceived stress. On the one hand, mental health partially mediated the relationship between perceived stress and job burnout. On the other hand, job burnout partially mediated the relationship between perceived stress and mental health. This not only confirmed the expected hypothesis, but also enriched the COR and complemented the stress-cognitive vulnerability theory. In this bidirectional mediating mechanism, the concept and idea of the interaction among individuals, society, and the environment were reflected, and the positive cognition and adaptation of people to society and the environment were demonstrated. This also suggested that efforts to reduce job burnout and promote mental health among healthcare workers required reducing stressors, developing stress reduction courses and programs aimed at healthcare workers, improving the ability to actively cope with stress and utilize resources, and implementing effective coping strategies and mechanisms.

Given the previous experiences of scholars in coping strategies, for example, Koh et al. (45) identified coping mechanisms to reduce burnout: physical health, clinical diversity, setting boundaries, transcendence, enthusiasm for work, realistic expectations, and organizational activities. Whitebird et al. (46) found that reducing burnout could also be achieved by physical exercise and social support. Perez et al. (47) and Maresca et al. (48) both discovered three effective stress coping strategies, such as self-care, social and emotional support, and emotional and physical detachment from work. In addition, healthcare workers also spontaneously proposed some intervention measures: mind–body skills training, stress education, cognitive skills, real-time implementation of brief strategies, learning resilience-enhancing skills, etc. (47). Therefore, in order to promote the healthy development of human resources in healthcare teams and improve the overall quality of healthcare services, various measures can be taken to reduce sources of stress, prevent work burnout, and ensure physical and mental health: healthcare institutions can strengthen screening for stress factors, flexibly arrange work content and intensity, reduce the triggers of perceived stress (49), regularly implement screening and intervention strategies for mental health status. Healthcare workers can strengthen their own physical and mental health, seek social and emotional support resources, take necessary breaks to achieve work-life balance (47), timely release negative emotions (50), regularly engage in mindfulness therapy (51), stress inoculation training (52), and activities of the Balint group (53).

## 5. Limitations

Despite we found a two-way mediating role between mental health and job burnout under the influence of perceived stress, our study had some limitations that pave the way for future research. Firstly, the research was conducted only in Wuxi, resulting in geographical results. Given the difference of areas, is there a possible different relationship among perceived stress, mental health and job burnout based on more Chinese samples? Future studies need further broaden the scope of research objects and adopt a multi-center cross-provincial research method. Secondly, this study was a cross-sectional study, and longitudinal research was necessary in the future to investigate the longitudinal effects of perceived stress on other variables and the causal relationships between variables.

## 6. Conclusion

Mental health can mediate the relationship between perceived stress and job burnout. Job burnout can mediate the relationship correlation between perceived stress and mental health. Bidirectional mediation mechanism interacted between mental health and job burnout. Healthcare managers should take actions to reduce and release stress among Chinese healthcare workers to improve their mental health level, and reduce their sense of job burnout. This was beneficial to higher quality of healthcare services and better evaluation and recognition of the entire society toward the medical industry.

## Data availability statement

The original contributions presented in the study are included in the article/supplementary material, further inquiries can be directed to the corresponding author.

## Ethics statement

The studies involving humans were approved by The Ethics Committees of Wuxi Maternity and Child Health Care Hospital. The studies were conducted in accordance with the local legislation and institutional requirements. The participants provided their written informed consent to participate in this study.

## Author contributions

MJ implemented this study and was responsible for data collection and provided assistance in reviewing the manuscript. MJ collaborated with ZL to complete data analysis, manuscript writing and revising. XZ and ML supported investigation and data collation. YF guided the study design, manuscript revising, and editing. All authors contributed to the article and approved the submitted version.

## Funding

This study was supported by the Project of “Taihu Light” Science and Technology Research of Wuxi Science and Technology Bureau (No K20221034), Innovation and Entrepreneurship Program of Jiangsu Province (No JSSCRC2021569), “Taihu Talent Plan” High-end Medical and Health Talents Project of Wuxi City [No (2020)50 Document of Xiwei Party], Key Project of Maternal and Child Health Research of Wuxi Health Care Commission (No FYKY202201), General Project of Philosophy and Social Science Research of Colleges in Jiangsu (No 2023SJYB0897).

## Conflict of interest

The authors declare that the research was conducted in the absence of any commercial or financial relationships that could be construed as a potential conflict of interest.

## Publisher’s note

All claims expressed in this article are solely those of the authors and do not necessarily represent those of their affiliated organizations, or those of the publisher, the editors and the reviewers. Any product that may be evaluated in this article, or claim that may be made by its manufacturer, is not guaranteed or endorsed by the publisher.
