# Supplementary material for: The influence of perceived stress of Chinese healthcare workers after the opening of COVID-19: the bidirectional mediation between mental health and job burnout
Source: Front Public Health. 2023 Aug 17;11:1252103. doi: 10.3389/fpubh.2023.1252103 (PMC10470117; doi:10.3389/fpubh.2023.1252103)
Supplement: Supplementary file 1 [file Data_Sheet_1.doc]

* Encoding: UTF-8.

DATASET ACTIVATE Data set 1.

ONEWAY Stress perception scale score mental health score, job burnout score BY age classification

/STATISTICS DESCRIPTIVES

/MISSING ANALYSIS

/POSTHOC=LSD ALPHA(0.05).

**one way**

| **notes appended to a book** | | |
| --- | --- | --- |
| The output created | | 14-July-2023 11:39:59 seconds |
| explanatory note | |  |
| import | data | F: \ submitted version \ data.sav |
|  | Datasets of activities | data set 1 |
|  | filter | <none> |
|  | weight | <none> |
|  | Split files | <none> |
|  | The N rows in the working data file | 792 |
| Missing value processing | Deletion definition | User-defined missing values are treated as missing. |
|  | The cases used | The statistic of each analysis is based on cases where there is no missing data for any variable in that analysis. |
| grammar | | ONEWAY Stress perception scale score mental health score, burnout score BY age classification  /STATISTICS DESCRIPTIVES  /MISSING ANALYSIS  /POSTHOC=LSD ALPHA(0.05). |
| resource | Processor time | 00 00:00:00.079 |
|  | Time used | 00 00:00:00.100 |

[Dataset 1] F: \ submitted version \ data.sav

| **description** | | | | | | | |
| --- | --- | --- | --- | --- | --- | --- | --- |
|  | | N | mean | standard deviation | standard error | The 95% confidence interval of the mean | |
|  |  |  |  |  |  | lower limit | upper limit |
| Stress perception scale score | 1 | 144 | 22.3611 | 7.25616 | .60468 | 21.1658 | 23.5564 |
|  | 2 | 249 | 23.0442 | 7.53233 | .47734 | 22.1040 | 23.9843 |
|  | 3 | 127 | 24.0472 | 7.75913 | .68851 | 22.6847 | 25.4098 |
|  | 4 | 272 | 21.7794 | 7.87044 | .47722 | 20.8399 | 22.7189 |
|  | tote | 792 | 22.6465 | 7.66533 | .27238 | 22.1118 | 23.1811 |
| Mental health score | 1 | 144 | 3.53 | 4.000 | .333 | 2.87 | 4.19 |
|  | 2 | 249 | 3.80 | 4.233 | .268 | 3.27 | 4.33 |
|  | 3 | 127 | 4.59 | 4.937 | .438 | 3.72 | 5.46 |
|  | 4 | 272 | 3.71 | 3.909 | .237 | 3.25 | 4.18 |
|  | tote | 792 | 3.85 | 4.212 | .150 | 3.55 | 4.14 |
| Job burnout score | 1 | 144 | 1.8584 | .92305 | .07692 | 1.7064 | 2.0105 |
|  | 2 | 249 | 1.9548 | .98383 | .06235 | 1.8320 | 2.0776 |
|  | 3 | 127 | 2.0493 | 1.20042 | .10652 | 1.8385 | 2.2601 |
|  | 4 | 272 | 1.7330 | 1.02176 | .06195 | 1.6110 | 1.8550 |
|  | tote | 792 | 1.8762 | 1.02887 | .03656 | 1.8045 | 1.9480 |

| **description** | | | |
| --- | --- | --- | --- |
|  | | minima | maxima |
| Stress perception scale scores | 1 | 7.00 | 43.00 |
|  | 2 | 4.00 | 44.00 |
|  | 3 | 5.00 | 45.00 |
|  | 4 | 2.00 | 48.00 |
|  | tote | 2.00 | 48.00 |
| Mental health score | 1 | 0 | 18 |
|  | 2 | 0 | 19 |
|  | 3 | 0 | 19 |
|  | 4 | 0 | 15 |
|  | tote | 0 | 19 |
| Job burnout score | 1 | .00 | 4.92 |
|  | 2 | .16 | 4.88 |
|  | 3 | .08 | 5.15 |
|  | 4 | .00 | 6.00 |
|  | tote | .00 | 6.00 |

| **ANOVA** | | | | | | |
| --- | --- | --- | --- | --- | --- | --- |
|  | | quadratic sum | df | mean square | F | significance |
| Stress perception scale score | Inter group | 504.793 | 3 | 168.264 | 2.884 | .035 |
|  | Within group | 45972.218 | 788 | 58.340 |  |  |
|  | Total | 46477.010 | 791 |  |  |  |
| Mental health score | Inter group | 90.231 | 3 | 30.077 | 1.700 | .166 |
|  | Within group | 13943.587 | 788 | 17.695 |  |  |
|  | Total | 14033.818 | 791 |  |  |  |
| Job burnout score | Inter group | 10.966 | 3 | 3.655 | 3.486 | .015 |
|  | Within group | 826.373 | 788 | 1.049 |  |  |
|  | Total | 837.339 | 791 |  |  |  |

**Check after this**

| **multiple comparisons** | | | | | |
| --- | --- | --- | --- | --- | --- |
| LSD | | | | | |
| dependent variable | (I) character classification by age | (J) character classification by age | Mean difference (I-J) | standard error | significance |
| Stress perception scale score | 1 | 2 | -.68307 | .79965 | .393 |
|  |  | 3 | -1.68613 | .92979 | .070 |
|  |  | 4 | .58170 | .78716 | .460 |
|  | 2 | 1 | .68307 | .79965 | .393 |
|  |  | 3 | -1.00307 | .83287 | .229 |
|  |  | 4 | 1.26476 | .66991 | .059 |
|  | 3 | 1 | 1.68613 | .92979 | .070 |
|  |  | 2 | 1.00307 | .83287 | .229 |
|  |  | 4 | 2.26783^*^ | .82089 | .006 |
|  | 4 | 1 | -.58170 | .78716 | .460 |
|  |  | 2 | -1.26476 | .66991 | .059 |
|  |  | 3 | -2.26783^*^ | .82089 | .006 |
| Mental health score | 1 | 2 | -.275 | .440 | .532 |
|  |  | 3 | -1.063^*^ | .512 | .038 |
|  |  | 4 | -.185 | .434 | .669 |
|  | 2 | 1 | .275 | .440 | .532 |
|  |  | 3 | -.787 | .459 | .086 |
|  |  | 4 | .090 | .369 | .807 |
|  | 3 | 1 | 1.063^*^ | .512 | .038 |
|  |  | 2 | .787 | .459 | .086 |
|  |  | 4 | .877 | .452 | .053 |
|  | 4 | 1 | .185 | .434 | .669 |
|  |  | 2 | -.090 | .369 | .807 |
|  |  | 3 | -.877 | .452 | .053 |
| Job burnout score | 1 | 2 | -.09638 | .10721 | .369 |
|  |  | 3 | -.19089 | .12466 | .126 |
|  |  | 4 | .12541 | .10554 | .235 |
|  | 2 | 1 | .09638 | .10721 | .369 |
|  |  | 3 | -.09451 | .11167 | .398 |
|  |  | 4 | .22178^*^ | .08982 | .014 |
|  | 3 | 1 | .19089 | .12466 | .126 |
|  |  | 2 | .09451 | .11167 | .398 |
|  |  | 4 | .31630^*^ | .11006 | .004 |
|  | 4 | 1 | -.12541 | .10554 | .235 |
|  |  | 2 | -.22178^*^ | .08982 | .014 |
|  |  | 3 | -.31630^*^ | .11006 | .004 |

| **multiple comparisons** | | | | |
| --- | --- | --- | --- | --- |
| LSD | | | | |
| dependent variable | (I) character classification by age | (J) character classification by age | And the 95% confidence interval | |
|  |  |  | lower limit | upper limit |
| Stress perception scale scores | 1 | 2 | -2.2528 | .8866 |
|  |  | 3 | -3.5113 | .1390 |
|  |  | 4 | -.9635 | 2.1269 |
|  | 2 | 1 | -.8866 | 2.2528 |
|  |  | 3 | -2.6380 | .6318 |
|  |  | 4 | -.0503 | 2.5798 |
|  | 3 | 1 | -.1390 | 3.5113 |
|  |  | 2 | -.6318 | 2.6380 |
|  |  | 4 | .6564 | 3.8792 |
|  | 4 | 1 | -2.1269 | .9635 |
|  |  | 2 | -2.5798 | .0503 |
|  |  | 3 | -3.8792 | -.6564 |
| Mental health score | 1 | 2 | -1.14 | .59 |
|  |  | 3 | -2.07 | -.06 |
|  |  | 4 | -1.04 | .67 |
|  | 2 | 1 | -.59 | 1.14 |
|  |  | 3 | -1.69 | .11 |
|  |  | 4 | -.63 | .81 |
|  | 3 | 1 | .06 | 2.07 |
|  |  | 2 | -.11 | 1.69 |
|  |  | 4 | -.01 | 1.76 |
|  | 4 | 1 | -.67 | 1.04 |
|  |  | 2 | -.81 | .63 |
|  |  | 3 | -1.76 | .01 |
| Job burnout score | 1 | 2 | -.3068 | .1141 |
|  |  | 3 | -.4356 | .0538 |
|  |  | 4 | -.0818 | .3326 |
|  | 2 | 1 | -.1141 | .3068 |
|  |  | 3 | -.3137 | .1247 |
|  |  | 4 | .0455 | .3981 |
|  | 3 | 1 | -.0538 | .4356 |
|  |  | 2 | -.1247 | .3137 |
|  |  | 4 | .1003 | .5323 |
|  | 4 | 1 | -.3326 | .0818 |
|  |  | 2 | -.3981 | -.0455 |
|  |  | 3 | -.5323 | -.1003 |

|  |
| --- |
| *. The level of significance for the mean difference was 0.05. |

ONEWAY Stress perception scale score mental health score, job burnout score BY gender

/STATISTICS DESCRIPTIVES

/MISSING ANALYSIS

/POSTHOC=LSD ALPHA(0.05).

**one way**

| **notes appended to a book** | | |
| --- | --- | --- |
| The output created | | 14-July-2023 11:39:59 seconds |
| explanatory note | |  |
| import | data | F: \ submitted version \ data.sav |
|  | Datasets of activities | data set 1 |
|  | filter | <none> |
|  | weight | <none> |
|  | Split files | <none> |
|  | The N rows in the working data file | 792 |
| Missing value processing | Deletion definition | User-defined missing values are treated as missing. |
|  | The cases used | The statistic of each analysis is based on cases where there is no missing data for any variable in that analysis. |
| grammar | | ONEWAY Stress perception scale score mental health score, burnout score BY gender  /STATISTICS DESCRIPTIVES  /MISSING ANALYSIS  /POSTHOC=LSD ALPHA(0.05). |
| resource | Processor time | 00 00:00:00.000 |
|  | Time used | 00 00:00:00.010 |

[Dataset 1] F: \ submitted version \ data.sav

| **warn** |
| --- |
| No "after this" test was performed for the stress perception scale score because the number of groups was less than three. |
| A "after this" test was not performed for mental health scores, because the number of groups was less than three. |
| No "after this" test was performed for job burnout scores because the number of groups was less than three. |

| **description** | | | | | | | |
| --- | --- | --- | --- | --- | --- | --- | --- |
|  | | N | mean | standard deviation | standard error | The 95% confidence interval of the mean | |
|  |  |  |  |  |  | lower limit | upper  limit |
| Stress perception scale score | 1 | 96 | 22.6146 | 6.68127 | .68190 | 21.2608 | 23.9683 |
|  | 2 | 696 | 22.6509 | 7.79560 | .29549 | 22.0707 | 23.2310 |
|  | tote | 792 | 22.6465 | 7.66533 | .27238 | 22.1118 | 23.1811 |
| Mental health score | 1 | 96 | 3.16 | 4.120 | .421 | 2.32 | 3.99 |
|  | 2 | 696 | 3.94 | 4.219 | .160 | 3.63 | 4.26 |
|  | tote | 792 | 3.85 | 4.212 | .150 | 3.55 | 4.14 |
| Job burnout score | 1 | 96 | 1.8000 | .95300 | .09727 | 1.6069 | 1.9931 |
|  | 2 | 696 | 1.8868 | 1.03911 | .03939 | 1.8094 | 1.9641 |
|  | tote | 792 | 1.8762 | 1.02887 | .03656 | 1.8045 | 1.9480 |

| **description** | | | |
| --- | --- | --- | --- |
|  | | minima | maxima |
| Stress perception scale score | 1 | 4.00 | 40.00 |
|  | 2 | 2.00 | 48.00 |
|  | tote | 2.00 | 48.00 |
| Mental health score | 1 | 0 | 17 |
|  | 2 | 0 | 19 |
|  | tote | 0 | 19 |
| Job burnout score | 1 | .00 | 4.80 |
|  | 2 | .00 | 6.00 |
|  | tote | .00 | 6.00 |

| **ANOVA** | | | | | | |
| --- | --- | --- | --- | --- | --- | --- |
|  | | quadratic sum | df | mean square | F | significance |
| Stress perception scale score | Inter group | .111 | 1 | .111 | .002 | .965 |
|  | Within group | 46476.899 | 790 | 58.832 |  |  |
|  | Total | 46477.010 | 791 |  |  |  |
| Mental health score | Inter group | 52.347 | 1 | 52.347 | 2.958 | .086 |
|  | Within group | 13981.471 | 790 | 17.698 |  |  |
|  | Total | 14033.818 | 791 |  |  |  |
| Job burnout score | Inter group | .635 | 1 | .635 | .600 | .439 |
|  | Within group | 836.704 | 790 | 1.059 |  |  |
|  | Total | 837.339 | 791 |  |  |  |

ONEWAY Stress perception scale score mental health score, job burnout score BY marriage

/STATISTICS DESCRIPTIVES

/MISSING ANALYSIS

/POSTHOC=LSD ALPHA(0.05).

**one way**

| **notes appended to a book** | | |
| --- | --- | --- |
| The output created | | 14-July-2023 11:39:59 seconds |
| explanatory note | |  |
| import | data | F: \ submitted version \ data.sav |
|  | Datasets of activities | data set 1 |
|  | filter | <none> |
|  | weight | <none> |
|  | Split files | <none> |
|  | The N rows in the working data file | 792 |
| Missing value processing | Deletion definition | User-defined missing values are treated as missing. |
|  | The cases used | The statistic of each analysis is based on cases where there is no missing data for any variable in that analysis. |
| grammar | | ONEWAY Stress perception scale score mental health score, burnout score BY marriage  /STATISTICS DESCRIPTIVES  /MISSING ANALYSIS  /POSTHOC=LSD ALPHA(0.05). |
| resource | Processor time | 00 00:00:00.015 |
|  | Time used | 00 00:00:00.009 |

[Dataset 1] F: \ submitted version \ data.sav

| **warn** |
| --- |
| No "after this" test was performed for the stress perception scale score because the number of groups was less than three. |
| A "after this" test was not performed for mental health scores, because the number of groups was less than three. |
| No "after this" test was performed for burnout scores because the number of groups was less than three. |

| **description** | | | | | | | |
| --- | --- | --- | --- | --- | --- | --- | --- |
|  | | N | mean | standard deviation | standard error | The 95% confidence interval of the mean | |
|  |  |  |  |  |  | lower limit | upper limit |
| Stress perception scale score | 1 | 618 | 22.4660 | 7.67806 | .30886 | 21.8595 | 23.0726 |
|  | 2 | 174 | 23.2874 | 7.60728 | .57671 | 22.1491 | 24.4256 |
|  | total | 792 | 22.6465 | 7.66533 | .27238 | 22.1118 | 23.1811 |
| Mental health score | 1 | 618 | 3.77 | 4.132 | .166 | 3.44 | 4.10 |
|  | 2 | 174 | 4.13 | 4.486 | .340 | 3.46 | 4.80 |
|  | total | 792 | 3.85 | 4.212 | .150 | 3.55 | 4.14 |
| Job burnout score | 1 | 618 | 1.8326 | 1.02287 | .04115 | 1.7518 | 1.9134 |
|  | 2 | 174 | 2.0313 | 1.03811 | .07870 | 1.8760 | 2.1866 |
|  | total | 792 | 1.8762 | 1.02887 | .03656 | 1.8045 | 1.9480 |

| **description** | | | |
| --- | --- | --- | --- |
|  | | minima | maxima |
| Stress perception scale score | 1 | 2.00 | 45.00 |
|  | 2 | 3.00 | 48.00 |
|  | total | 2.00 | 48.00 |
| Mental health score | 1 | 0 | 19 |
|  | 2 | 0 | 19 |
|  | total | 0 | 19 |
| Job burnout score | 1 | .00 | 5.15 |
|  | 2 | .05 | 6.00 |
|  | total | .00 | 6.00 |

| **ANOVA** | | | | | | |
| --- | --- | --- | --- | --- | --- | --- |
|  | | quadratic sum | df | mean square | F | significance |
| Stress perception scale score | Inter group | 91.592 | 1 | 91.592 | 1.560 | .212 |
|  | Within group | 46385.419 | 790 | 58.716 |  |  |
|  | total | 46477.010 | 791 |  |  |  |
| Mental health score | Inter group | 17.947 | 1 | 17.947 | 1.012 | .315 |
|  | Within group | 14015.871 | 790 | 17.742 |  |  |
|  | total | 14033.818 | 791 |  |  |  |
| Job burnout score | Inter group | 5.361 | 1 | 5.361 | 5.090 | .024 |
|  | Within group | 831.978 | 790 | 1.053 |  |  |
|  | total | 837.339 | 791 |  |  |  |

ONEWAY Stress perception scale score mental health score, job burnout score BY education level

/STATISTICS DESCRIPTIVES

/MISSING ANALYSIS

/POSTHOC=LSD ALPHA(0.05).

**one way**

| **notes appended to a book** | | |
| --- | --- | --- |
| The output created | | 14-July-2023 11:39:59 seconds |
| explanatory note | |  |
| import | data | F: \ submitted version \ data.sav |
|  | Datasets of activities | data set 1 |
|  | filter | <none> |
|  | weight | <none> |
|  | Split files | <none> |
|  | The N rows in the working data file | 792 |
| Missing value processing | Deletion definition | User-defined missing values are treated as missing. |
|  | The cases used | The statistic of each analysis is based on cases where there is no missing data for any variable in that analysis. |
| grammar | | ONEWAY Stress perception scale score mental health score, burnout score BY education level  /STATISTICS DESCRIPTIVES  /MISSING ANALYSIS  /POSTHOC=LSD ALPHA(0.05). |
| resource | Processor time | 00 00:00:00.000 |
|  | Time used | 00 00:00:00.011 |

[Dataset 1] F: \ submitted version \ data.sav

| **description** | | | | | | | |
| --- | --- | --- | --- | --- | --- | --- | --- |
|  | | N | mean | standard deviation | standard error | The 95% confidence interval of the mean | |
|  |  |  |  |  |  | lower limit | upper limit |
| Stress perception scale score | 1 | 79 | 21.7595 | 7.91496 | .89050 | 19.9866 | 23.5323 |
|  | 2 | 510 | 22.2333 | 7.68399 | .34025 | 21.5649 | 22.9018 |
|  | 3 | 184 | 24.2065 | 7.56832 | .55794 | 23.1057 | 25.3074 |
|  | 4 | 19 | 22.3158 | 5.03380 | 1.15483 | 19.8896 | 24.7420 |
|  | total | 792 | 22.6465 | 7.66533 | .27238 | 22.1118 | 23.1811 |
| Mental health score | 1 | 79 | 2.78 | 3.781 | .425 | 1.94 | 3.63 |
|  | 2 | 510 | 3.77 | 4.161 | .184 | 3.41 | 4.14 |
|  | 3 | 184 | 4.52 | 4.472 | .330 | 3.87 | 5.17 |
|  | 4 | 19 | 3.74 | 3.885 | .891 | 1.86 | 5.61 |
|  | total | 792 | 3.85 | 4.212 | .150 | 3.55 | 4.14 |
| Job burnout score | 1 | 79 | 1.5716 | .97639 | .10985 | 1.3529 | 1.7903 |
|  | 2 | 510 | 1.8549 | 1.04474 | .04626 | 1.7640 | 1.9458 |
|  | 3 | 184 | 2.0707 | .99342 | .07324 | 1.9262 | 2.2152 |
|  | 4 | 19 | 1.8326 | .82511 | .18929 | 1.4349 | 2.2303 |
|  | total | 792 | 1.8762 | 1.02887 | .03656 | 1.8045 | 1.9480 |

| **description** | | | |
| --- | --- | --- | --- |
|  | | minima | maxima |
| Stress perception scale score | 1 | 4.00 | 37.00 |
|  | 2 | 2.00 | 48.00 |
|  | 3 | 9.00 | 44.00 |
|  | 4 | 12.00 | 30.00 |
|  | total | 2.00 | 48.00 |
| Mental health score | 1 | 0 | 14 |
|  | 2 | 0 | 18 |
|  | 3 | 0 | 19 |
|  | 4 | 0 | 15 |
|  | total | 0 | 19 |
| Job burnout score | 1 | .00 | 4.00 |
|  | 2 | .00 | 6.00 |
|  | 3 | .15 | 4.92 |
|  | 4 | .60 | 3.31 |
|  | total | .00 | 6.00 |

| **ANOVA** | | | | | | |
| --- | --- | --- | --- | --- | --- | --- |
|  | | quadratic sum | df | mean square | F | significance |
| Stress perception scale score | Inter group | 599.089 | 3 | 199.696 | 3.430 | .017 |
|  | Within group | 45877.921 | 788 | 58.221 |  |  |
|  | total | 46477.010 | 791 |  |  |  |
| Mental health score | Inter group | 175.811 | 3 | 58.604 | 3.332 | .019 |
|  | Within group | 13858.008 | 788 | 17.586 |  |  |
|  | total | 14033.818 | 791 |  |  |  |
| Job burnout score | Inter group | 14.556 | 3 | 4.852 | 4.647 | .003 |
|  | Within group | 822.782 | 788 | 1.044 |  |  |
|  | total | 837.339 | 791 |  |  |  |

**Check after this**

| **multiple comparisons** | | | | | |
| --- | --- | --- | --- | --- | --- |
| LSD | | | | | |
| dependent variable | (I) Education level | (J) Education level | Mean difference (I-J) | standard error | significance |
| Stress perception scale score | 1 | 2 | -.47384 | .92257 | .608 |
|  |  | 3 | -2.44703^*^ | 1.02635 | .017 |
|  |  | 4 | -.55630 | 1.94967 | .775 |
|  | 2 | 1 | .47384 | .92257 | .608 |
|  |  | 3 | -1.97319^*^ | .65618 | .003 |
|  |  | 4 | -.08246 | 1.78281 | .963 |
|  | 3 | 1 | 2.44703^*^ | 1.02635 | .017 |
|  |  | 2 | 1.97319^*^ | .65618 | .003 |
|  |  | 4 | 1.89073 | 1.83866 | .304 |
|  | 4 | 1 | .55630 | 1.94967 | .775 |
|  |  | 2 | .08246 | 1.78281 | .963 |
|  |  | 3 | -1.89073 | 1.83866 | .304 |
| Mental health score | 1 | 2 | -.990 | .507 | .051 |
|  |  | 3 | -1.737^*^ | .564 | .002 |
|  |  | 4 | -.952 | 1.072 | .375 |
|  | 2 | 1 | .990 | .507 | .051 |
|  |  | 3 | -.747^*^ | .361 | .039 |
|  |  | 4 | .038 | .980 | .969 |
|  | 3 | 1 | 1.737^*^ | .564 | .002 |
|  |  | 2 | .747^*^ | .361 | .039 |
|  |  | 4 | .785 | 1.011 | .438 |
|  | 4 | 1 | .952 | 1.072 | .375 |
|  |  | 2 | -.038 | .980 | .969 |
|  |  | 3 | -.785 | 1.011 | .438 |
| Job burnout score | 1 | 2 | -.28325^*^ | .12355 | .022 |
|  |  | 3 | -.49906^*^ | .13745 | .000 |
|  |  | 4 | -.26099 | .26110 | .318 |
|  | 2 | 1 | .28325^*^ | .12355 | .022 |
|  |  | 3 | -.21581^*^ | .08787 | .014 |
|  |  | 4 | .02226 | .23875 | .926 |
|  | 3 | 1 | .49906^*^ | .13745 | .000 |
|  |  | 2 | .21581^*^ | .08787 | .014 |
|  |  | 4 | .23807 | .24623 | .334 |
|  | 4 | 1 | .26099 | .26110 | .318 |
|  |  | 2 | -.02226 | .23875 | .926 |
|  |  | 3 | -.23807 | .24623 | .334 |

| **multiple comparisons** | | | | |
| --- | --- | --- | --- | --- |
| LSD | | | | |
| dependent variable | (I) Education level | (J) Education level | And the 95% confidence interval | |
|  |  |  | lower limit | upper limit |
| Stress perception scale score | 1 | 2 | -2.2848 | 1.3371 |
|  |  | 3 | -4.4617 | -.4323 |
|  |  | 4 | -4.3835 | 3.2709 |
|  | 2 | 1 | -1.3371 | 2.2848 |
|  |  | 3 | -3.2613 | -.6851 |
|  |  | 4 | -3.5821 | 3.4172 |
|  | 3 | 1 | .4323 | 4.4617 |
|  |  | 2 | .6851 | 3.2613 |
|  |  | 4 | -1.7185 | 5.5000 |
|  | 4 | 1 | -3.2709 | 4.3835 |
|  |  | 2 | -3.4172 | 3.5821 |
|  |  | 3 | -5.5000 | 1.7185 |
| Mental health score | 1 | 2 | -1.99 | .01 |
|  |  | 3 | -2.84 | -.63 |
|  |  | 4 | -3.06 | 1.15 |
|  | 2 | 1 | -.01 | 1.99 |
|  |  | 3 | -1.46 | -.04 |
|  |  | 4 | -1.89 | 1.96 |
|  | 3 | 1 | .63 | 2.84 |
|  |  | 2 | .04 | 1.46 |
|  |  | 4 | -1.20 | 2.77 |
|  | 4 | 1 | -1.15 | 3.06 |
|  |  | 2 | -1.96 | 1.89 |
|  |  | 3 | -2.77 | 1.20 |
| Job burnout score | 1 | 2 | -.5258 | -.0407 |
|  |  | 3 | -.7689 | -.2293 |
|  |  | 4 | -.7735 | .2515 |
|  | 2 | 1 | .0407 | .5258 |
|  |  | 3 | -.3883 | -.0433 |
|  |  | 4 | -.4464 | .4909 |
|  | 3 | 1 | .2293 | .7689 |
|  |  | 2 | .0433 | .3883 |
|  |  | 4 | -.2453 | .7214 |
|  | 4 | 1 | -.2515 | .7735 |
|  |  | 2 | -.4909 | .4464 |
|  |  | 3 | -.7214 | .2453 |

|  |
| --- |
| *. The level of significance for the mean difference was 0.05. |

ONEWAY Stress perception scale score mental health score, job burnout score BY job title

/STATISTICS DESCRIPTIVES

/MISSING ANALYSIS

/POSTHOC=LSD ALPHA(0.05).

**one way**

| **notes appended to a book** | | |
| --- | --- | --- |
| The output created | | 14-July-2023 11:39:59 seconds |
| explanatory note | |  |
| import | data | F: \ submitted version \ data.sav |
|  | Datasets of activities | data set 1 |
|  | filter | <none> |
|  | weight | <none> |
|  | Split files | <none> |
|  | The N rows in the working data file | 792 |
| Missing value processing | Deletion definition | User-defined missing values are treated as missing. |
|  | The cases used | The statistic of each analysis is based on cases where there is no missing data for any variable in that analysis. |
| grammar | | ONEWAY Stress perception scale score mental health score, job burnout score BY job title  /STATISTICS DESCRIPTIVES  /MISSING ANALYSIS  /POSTHOC=LSD ALPHA(0.05). |
| resource | Processor time | 00 00:00:00.016 |
|  | Time used | 00 00:00:00.014 |

[Dataset 1] F: \ submitted version \ data.sav

| **description** | | | | | | | |
| --- | --- | --- | --- | --- | --- | --- | --- |
|  | | N | mean | standard deviation | standard error | The 95% confidence interval of the mean | |
|  |  |  |  |  |  | lower limit | upper limit |
| Stress perception scale score | 1 | 377 | 22.9178 | 7.74261 | .39876 | 22.1337 | 23.7019 |
|  | 2 | 242 | 22.6529 | 7.06133 | .45392 | 21.7587 | 23.5470 |
|  | 3 | 131 | 23.0382 | 8.15089 | .71215 | 21.6293 | 24.4471 |
|  | 4 | 42 | 18.9524 | 8.05757 | 1.24331 | 16.4415 | 21.4633 |
|  | total | 792 | 22.6465 | 7.66533 | .27238 | 22.1118 | 23.1811 |
| Mental health score | 1 | 377 | 3.69 | 4.396 | .226 | 3.25 | 4.14 |
|  | 2 | 242 | 3.96 | 3.999 | .257 | 3.46 | 4.47 |
|  | 3 | 131 | 4.36 | 4.338 | .379 | 3.61 | 5.11 |
|  | 4 | 42 | 2.98 | 3.080 | .475 | 2.02 | 3.94 |
|  | total | 792 | 3.85 | 4.212 | .150 | 3.55 | 4.14 |
| Job burnout score | 1 | 377 | 1.9264 | 1.05164 | .05416 | 1.8199 | 2.0329 |
|  | 2 | 242 | 1.9237 | .94438 | .06071 | 1.8042 | 2.0433 |
|  | 3 | 131 | 1.8352 | 1.11045 | .09702 | 1.6432 | 2.0271 |
|  | 4 | 42 | 1.2807 | .85041 | .13122 | 1.0157 | 1.5457 |
|  | total | 792 | 1.8762 | 1.02887 | .03656 | 1.8045 | 1.9480 |

| **description** | | | |
| --- | --- | --- | --- |
|  | | minima | maxima |
| Stress perception scale score | 1 | 4.00 | 44.00 |
|  | 2 | 4.00 | 45.00 |
|  | 3 | 4.00 | 48.00 |
|  | 4 | 2.00 | 30.00 |
|  | tote | 2.00 | 48.00 |
| Mental health score | 1 | 0 | 18 |
|  | 2 | 0 | 19 |
|  | 3 | 0 | 19 |
|  | 4 | 0 | 12 |
|  | tote | 0 | 19 |
| Job burnout score | 1 | .00 | 5.15 |
|  | 2 | .15 | 4.84 |
|  | 3 | .15 | 6.00 |
|  | 4 | .08 | 3.31 |
|  | tote | .00 | 6.00 |

| **ANOVA** | | | | | | |
| --- | --- | --- | --- | --- | --- | --- |
|  | | quadratic sum | df | mean square | F | significance |
| Stress perception scale score | Inter group | 621.002 | 3 | 207.001 | 3.557 | .014 |
|  | Within group | 45856.008 | 788 | 58.193 |  |  |
|  | total | 46477.010 | 791 |  |  |  |
| Mental health score | Inter group | 78.119 | 3 | 26.040 | 1.470 | .221 |
|  | Within group | 13955.699 | 788 | 17.710 |  |  |
|  | total | 14033.818 | 791 |  |  |  |
| Job burnout score | Inter group | 16.610 | 3 | 5.537 | 5.316 | .001 |
|  | Within group | 820.729 | 788 | 1.042 |  |  |
|  | total | 837.339 | 791 |  |  |  |

**Check after this**

| **multiple comparisons** | | | | | | | |
| --- | --- | --- | --- | --- | --- | --- | --- |
| LSD | | | | | | | |
| dependent variable | (I) job title | (J) job title | Mean difference (I-J) | standard error | conspicuousness | And the 95% confidence interval | |
|  |  |  |  |  |  | lower limit | upper limit |
| Stress perception scale score | 1 | 2 | .26488 | .62835 | .673 | -.9686 | 1.4983 |
|  |  | 3 | -.12040 | .77368 | .876 | -1.6391 | 1.3983 |
|  |  | 4 | 3.96539^*^ | 1.24093 | .001 | 1.5295 | 6.4013 |
|  | 2 | 1 | -.26488 | .62835 | .673 | -1.4983 | .9686 |
|  |  | 3 | -.38528 | .82746 | .642 | -2.0096 | 1.2390 |
|  |  | 4 | 3.70051^*^ | 1.27515 | .004 | 1.1974 | 6.2036 |
|  | 3 | 1 | .12040 | .77368 | .876 | -1.3983 | 1.6391 |
|  |  | 2 | .38528 | .82746 | .642 | -1.2390 | 2.0096 |
|  |  | 4 | 4.08579^*^ | 1.35269 | .003 | 1.4305 | 6.7411 |
|  | 4 | 1 | -3.96539^*^ | 1.24093 | .001 | -6.4013 | -1.5295 |
|  |  | 2 | -3.70051^*^ | 1.27515 | .004 | -6.2036 | -1.1974 |
|  |  | 3 | -4.08579^*^ | 1.35269 | .003 | -6.7411 | -1.4305 |
| Mental health score | 1 | 2 | -.268 | .347 | .440 | -.95 | .41 |
|  |  | 3 | -.664 | .427 | .120 | -1.50 | .17 |
|  |  | 4 | .719 | .685 | .294 | -.63 | 2.06 |
|  | 2 | 1 | .268 | .347 | .440 | -.41 | .95 |
|  |  | 3 | -.396 | .456 | .386 | -1.29 | .50 |
|  |  | 4 | .987 | .703 | .161 | -.39 | 2.37 |
|  | 3 | 1 | .664 | .427 | .120 | -.17 | 1.50 |
|  |  | 2 | .396 | .456 | .386 | -.50 | 1.29 |
|  |  | 4 | 1.383 | .746 | .064 | -.08 | 2.85 |
|  | 4 | 1 | -.719 | .685 | .294 | -2.06 | .63 |
|  |  | 2 | -.987 | .703 | .161 | -2.37 | .39 |
|  |  | 3 | -1.383 | .746 | .064 | -2.85 | .08 |
| Job burnout score | 1 | 2 | .00264 | .08406 | .975 | -.1624 | .1677 |
|  |  | 3 | .09123 | .10351 | .378 | -.1120 | .2944 |
|  |  | 4 | .64567^*^ | .16602 | .000 | .3198 | .9715 |
|  | 2 | 1 | -.00264 | .08406 | .975 | -.1677 | .1624 |
|  |  | 3 | .08859 | .11070 | .424 | -.1287 | .3059 |
|  |  | 4 | .64303^*^ | .17059 | .000 | .3082 | .9779 |
|  | 3 | 1 | -.09123 | .10351 | .378 | -.2944 | .1120 |
|  |  | 2 | -.08859 | .11070 | .424 | -.3059 | .1287 |
|  |  | 4 | .55444^*^ | .18097 | .002 | .1992 | .9097 |
|  | 4 | 1 | -.64567^*^ | .16602 | .000 | -.9715 | -.3198 |
|  |  | 2 | -.64303^*^ | .17059 | .000 | -.9779 | -.3082 |
|  |  | 3 | -.55444^*^ | .18097 | .002 | -.9097 | -.1992 |
| *. The level of significance for the mean difference was 0.05. | | | | | | | |

ONEWAY Stress perception scale score mental health score, job burnout score BY employment method

/STATISTICS DESCRIPTIVES

/MISSING ANALYSIS

/POSTHOC=LSD ALPHA(0.05).

**one way**

| **notes appended to a book** | | |
| --- | --- | --- |
| The output created | | 14-July-2023 11:39:59 seconds |
| explanatory note | |  |
| import | data | F: \ submitted version \ data.sav |
|  | Datasets of activities | data set 1 |
|  | filter | <none> |
|  | weight | <none> |
|  | Split files | <none> |
|  | The N rows in the working data file | 792 |
| Missing value processing | Deletion definition | User-defined missing values are treated as missing. |
|  | The cases used | The statistic of each analysis is based on cases where there is no missing data for any variable in that analysis. |
| grammar | | ONEWAY Stress perception scale score mental health score, job burnout score BY employment method  /STATISTICS DESCRIPTIVES  /MISSING ANALYSIS  /POSTHOC=LSD ALPHA(0.05). |
| resource | Processor time | 00 00:00:00.015 |
|  | Time used | 00 00:00:00.007 |

[Dataset 1] F: \ submitted version \ data.sav

| **warn** |
| --- |
| No "after this" test was performed for the stress perception scale score because the number of groups was less than three. |
| A "after this" test was not performed for mental health scores, because the number of groups was less than three. |
| No "after this" test was performed for burnout scores because the number of groups was less than three. |

| **description** | | | | | | | |
| --- | --- | --- | --- | --- | --- | --- | --- |
|  | | N | mean | standard deviation | standard error | The 95% confidence interval of the mean | |
|  |  |  |  |  |  | lower limit | Upper limit |
| Stress perception scale score | 1 | 479 | 23.0167 | 7.76052 | .35459 | 22.3200 | 23.7134 |
|  | 2 | 313 | 22.0799 | 7.49423 | .42360 | 21.2464 | 22.9133 |
|  | total | 792 | 22.6465 | 7.66533 | .27238 | 22.1118 | 23.1811 |
| Mental health score | 1 | 479 | 4.17 | 4.242 | .194 | 3.79 | 4.55 |
|  | 2 | 313 | 3.36 | 4.126 | .233 | 2.90 | 3.82 |
|  | total | 792 | 3.85 | 4.212 | .150 | 3.55 | 4.14 |
| Job burnout score | 1 | 479 | 1.9061 | 1.04290 | .04765 | 1.8124 | 1.9997 |
|  | 2 | 313 | 1.8306 | 1.00697 | .05692 | 1.7186 | 1.9426 |
|  | total | 792 | 1.8762 | 1.02887 | .03656 | 1.8045 | 1.9480 |

| **description** | | | |
| --- | --- | --- | --- |
|  | | minima | maxima |
| Stress perception scale score | 1 | 2.00 | 48.00 |
|  | 2 | 4.00 | 43.00 |
|  | total | 2.00 | 48.00 |
| Mental health score | 1 | 0 | 19 |
|  | 2 | 0 | 19 |
|  | total | 0 | 19 |
| Job burnout score | 1 | .00 | 6.00 |
|  | 2 | .00 | 4.80 |
|  | total | .00 | 6.00 |

| **ANOVA** | | | | | | |
| --- | --- | --- | --- | --- | --- | --- |
|  | | quadratic sum | df | mean square | F | significance |
| Stress perception scale score | Inter group | 166.141 | 1 | 166.141 | 2.834 | .093 |
|  | Within group | 46310.870 | 790 | 58.621 |  |  |
|  | total | 46477.010 | 791 |  |  |  |
| Mental health score | Inter group | 122.975 | 1 | 122.975 | 6.984 | .008 |
|  | Within group | 13910.843 | 790 | 17.609 |  |  |
|  | total | 14033.818 | 791 |  |  |  |
| Job burnout score | Inter group | 1.079 | 1 | 1.079 | 1.019 | .313 |
|  | Within group | 836.260 | 790 | 1.059 |  |  |
|  | total | 837.339 | 791 |  |  |  |

ONEWAY Stress perception scale score mental health score, job burnout score BY monthly income level

/STATISTICS DESCRIPTIVES

/MISSING ANALYSIS

/POSTHOC=LSD ALPHA(0.05).

**one way**

| **notes appended to a book** | | |
| --- | --- | --- |
| The output created | | 14-July-2023 11:39:59 seconds |
| explanatory note | |  |
| import | data | F: \ submitted version \ data.sav |
|  | Datasets of activities | data set 1 |
|  | filter | <none> |
|  | weight | <none> |
|  | Split files | <none> |
|  | The N rows in the working data file | 792 |
| Missing value processing | Deletion definition | User-defined missing values are treated as missing. |
|  | The cases used | The statistic of each analysis is based on cases where there is no missing data for any variable in that analysis. |
| grammar | | ONEWAY Stress perception scale score mental health score, job burnout score BY monthly income level  /STATISTICS DESCRIPTIVES  /MISSING ANALYSIS  /POSTHOC=LSD ALPHA(0.05). |
| resource | Processor time | 00 00:00:00.000 |
|  | Time used | 00 00:00:00.013 |

[Dataset 1] F: \ submitted version \ data.sav

| **description** | | | | | | | |
| --- | --- | --- | --- | --- | --- | --- | --- |
|  | | N | mean | standard deviation | standard error | The 95% confidence interval of the mean | |
|  |  |  |  |  |  | lower limit | upper limit |
| Stress perception scale score | 1 | 6 | 23.3333 | 4.45720 | 1.81965 | 18.6558 | 28.0109 |
|  | 2 | 206 | 23.2573 | 7.56738 | .52724 | 22.2178 | 24.2968 |
|  | 3 | 461 | 22.8373 | 7.74636 | .36078 | 22.1283 | 23.5463 |
|  | 4 | 119 | 20.8151 | 7.44467 | .68245 | 19.4637 | 22.1666 |
|  | total | 792 | 22.6465 | 7.66533 | .27238 | 22.1118 | 23.1811 |
| Mental health score | 1 | 6 | 2.00 | 2.449 | 1.000 | -.57 | 4.57 |
|  | 2 | 206 | 3.98 | 4.380 | .305 | 3.38 | 4.58 |
|  | 3 | 461 | 3.94 | 4.326 | .201 | 3.55 | 4.34 |
|  | 4 | 119 | 3.34 | 3.450 | .316 | 2.72 | 3.97 |
|  | total | 792 | 3.85 | 4.212 | .150 | 3.55 | 4.14 |
| Job burnout score | 1 | 6 | 1.9883 | .77166 | .31503 | 1.1785 | 2.7981 |
|  | 2 | 206 | 1.9307 | 1.04836 | .07304 | 1.7867 | 2.0747 |
|  | 3 | 461 | 1.9385 | 1.01235 | .04715 | 1.8459 | 2.0312 |
|  | 4 | 119 | 1.5350 | 1.01383 | .09294 | 1.3509 | 1.7190 |
|  | total | 792 | 1.8762 | 1.02887 | .03656 | 1.8045 | 1.9480 |

| **description** | | | |
| --- | --- | --- | --- |
|  | | minima | maxima |
| Stress perception scale score | 1 | 17.00 | 28.00 |
|  | 2 | 5.00 | 43.00 |
|  | 3 | 2.00 | 45.00 |
|  | 4 | 4.00 | 48.00 |
|  | total | 2.00 | 48.00 |
| Mental health score | 1 | 0 | 5 |
|  | 2 | 0 | 19 |
|  | 3 | 0 | 17 |
|  | 4 | 0 | 14 |
|  | total | 0 | 19 |
| Job burnout score | 1 | .64 | 2.68 |
|  | 2 | .00 | 4.90 |
|  | 3 | .05 | 5.15 |
|  | 4 | .00 | 6.00 |
|  | total | .00 | 6.00 |

| **ANOVA** | | | | | | |
| --- | --- | --- | --- | --- | --- | --- |
|  | | quadratic sum | df | mean square | F | significance |
| Stress perception scale score | Inter group | 495.582 | 3 | 165.194 | 2.831 | .038 |
|  | Within group | 45981.428 | 788 | 58.352 |  |  |
|  | total | 46477.010 | 791 |  |  |  |
| Mental health score | Inter group | 58.488 | 3 | 19.496 | 1.099 | .349 |
|  | Within group | 13975.330 | 788 | 17.735 |  |  |
|  | total | 14033.818 | 791 |  |  |  |
| Professional burnout score | Inter group | 16.336 | 3 | 5.445 | 5.227 | .001 |
|  | Within group | 821.002 | 788 | 1.042 |  |  |
|  | total | 837.339 | 791 |  |  |  |

**Check after this**

| **multiple comparisons** | | | | | |
| --- | --- | --- | --- | --- | --- |
| LSD | | | | | |
| dependent variable | (I) Monthly income level | (J) Monthly income level | Mean difference (I-J) | standard error | significance |
| Stress perception scale score | 1 | 2 | .07605 | 3.16364 | .981 |
|  |  | 3 | .49602 | 3.13878 | .874 |
|  |  | 4 | 2.51821 | 3.19620 | .431 |
|  | 2 | 1 | -.07605 | 3.16364 | .981 |
|  |  | 3 | .41997 | .64019 | .512 |
|  |  | 4 | 2.44216^*^ | .87955 | .006 |
|  | 3 | 1 | -.49602 | 3.13878 | .874 |
|  |  | 2 | -.41997 | .64019 | .512 |
|  |  | 4 | 2.02218^*^ | .78545 | .010 |
|  | 4 | 1 | -2.51821 | 3.19620 | .431 |
|  |  | 2 | -2.44216^*^ | .87955 | .006 |
|  |  | 3 | -2.02218^*^ | .78545 | .010 |
| Mental health score | 1 | 2 | -1.981 | 1.744 | .256 |
|  |  | 3 | -1.944 | 1.730 | .262 |
|  |  | 4 | -1.345 | 1.762 | .446 |
|  | 2 | 1 | 1.981 | 1.744 | .256 |
|  |  | 3 | .037 | .353 | .917 |
|  |  | 4 | .636 | .485 | .190 |
|  | 3 | 1 | 1.944 | 1.730 | .262 |
|  |  | 2 | -.037 | .353 | .917 |
|  |  | 4 | .599 | .433 | .167 |
|  | 4 | 1 | 1.345 | 1.762 | .446 |
|  |  | 2 | -.636 | .485 | .190 |
|  |  | 3 | -.599 | .433 | .167 |
| Job burnout score | 1 | 2 | .05763 | .42273 | .892 |
|  |  | 3 | .04979 | .41941 | .906 |
|  |  | 4 | .45338 | .42709 | .289 |
|  | 2 | 1 | -.05763 | .42273 | .892 |
|  |  | 3 | -.00784 | .08554 | .927 |
|  |  | 4 | .39575^*^ | .11753 | .001 |
|  | 3 | 1 | -.04979 | .41941 | .906 |
|  |  | 2 | .00784 | .08554 | .927 |
|  |  | 4 | .40359^*^ | .10495 | .000 |
|  | 4 | 1 | -.45338 | .42709 | .289 |
|  |  | 2 | -.39575^*^ | .11753 | .001 |
|  |  | 3 | -.40359^*^ | .10495 | .000 |

| **multiple comparisons** | | | | |
| --- | --- | --- | --- | --- |
| LSD | | | | |
| dependent variable | (I) Monthly income level | (J) Monthly income level | And the 95% confidence interval | |
|  |  |  | lower limit | Upper limit |
| Stress perception scale score | 1 | 2 | -6.1341 | 6.2862 |
|  |  | 3 | -5.6653 | 6.6574 |
|  |  | 4 | -3.7559 | 8.7923 |
|  | 2 | 1 | -6.2862 | 6.1341 |
|  |  | 3 | -.8367 | 1.6766 |
|  |  | 4 | .7156 | 4.1687 |
|  | 3 | 1 | -6.6574 | 5.6653 |
|  |  | 2 | -1.6766 | .8367 |
|  |  | 4 | .4804 | 3.5640 |
|  | 4 | 1 | -8.7923 | 3.7559 |
|  |  | 2 | -4.1687 | -.7156 |
|  |  | 3 | -3.5640 | -.4804 |
| Mental health score | 1 | 2 | -5.40 | 1.44 |
|  |  | 3 | -5.34 | 1.45 |
|  |  | 4 | -4.80 | 2.11 |
|  | 2 | 1 | -1.44 | 5.40 |
|  |  | 3 | -.66 | .73 |
|  |  | 4 | -.32 | 1.59 |
|  | 3 | 1 | -1.45 | 5.34 |
|  |  | 2 | -.73 | .66 |
|  |  | 4 | -.25 | 1.45 |
|  | 4 | 1 | -2.11 | 4.80 |
|  |  | 2 | -1.59 | .32 |
|  |  | 3 | -1.45 | .25 |
| Job burnout score | 1 | 2 | -.7722 | .8874 |
|  |  | 3 | -.7735 | .8731 |
|  |  | 4 | -.3850 | 1.2917 |
|  | 2 | 1 | -.8874 | .7722 |
|  |  | 3 | -.1758 | .1601 |
|  |  | 4 | .1650 | .6265 |
|  | 3 | 1 | -.8731 | .7735 |
|  |  | 2 | -.1601 | .1758 |
|  |  | 4 | .1976 | .6096 |
|  | 4 | 1 | -1.2917 | .3850 |
|  |  | 2 | -.6265 | -.1650 |
|  |  | 3 | -.6096 | -.1976 |

|  |
| --- |
| *. The level of significance for the mean difference was 0.05. |

ONEWAY Stress perception scale score mental health score, job burnout score whether BY once belonged to the frontline anti-epidemic workers

/STATISTICS DESCRIPTIVES

/MISSING ANALYSIS

/POSTHOC=LSD ALPHA(0.05).

**one way**

| **notes appended to a book** | | |
| --- | --- | --- |
| The output created | | 14-July-2023 11:39:59 seconds |
| explanatory note | |  |
| import | data | F: \ submitted version \ data.sav |
|  | Datasets of activities | data set 1 |
|  | filter | <none> |
|  | weight | <none> |
|  | Split files | <none> |
|  | The N rows in the working data file | 792 |
| Missing value processing | Deletion definition | User-defined missing values are treated as missing. |
|  | The cases used | The statistic of each analysis is based on cases where there is no missing data for any variable in that analysis. |
| grammar | | ONEWAY Stress perception scale score mental health score, job burnout score whether BY once belonged to the frontline anti-epidemic workers  /STATISTICS DESCRIPTIVES  /MISSING ANALYSIS  /POSTHOC=LSD ALPHA(0.05). |
| resource | Processor time | 00 00:00:00.000 |
|  | Time used | 00 00:00:00.007 |

[Dataset 1] F: \ submitted version \ data.sav

| **warn** |
| --- |
| No "after this" test was performed for the stress perception scale score because the number of groups was less than three. |
| A "after this" test was not performed for mental health scores, because the number of groups was less than three. |
| No "after this" test was performed for burnout scores because the number of groups was less than three. |

| **description** | | | | | | | |
| --- | --- | --- | --- | --- | --- | --- | --- |
|  | | N | mean | standard deviation | standard error | The 95% confidence interval of the mean | |
|  |  |  |  |  |  | lower limit | upper limit |
| Stress perception scale score | 1 | 197 | 23.4569 | 7.36057 | .52442 | 22.4226 | 24.4911 |
|  | 2 | 595 | 22.3782 | 7.75095 | .31776 | 21.7541 | 23.0022 |
|  | total | 792 | 22.6465 | 7.66533 | .27238 | 22.1118 | 23.1811 |
| Mental health score | 1 | 197 | 4.45 | 4.422 | .315 | 3.83 | 5.07 |
|  | 2 | 595 | 3.65 | 4.125 | .169 | 3.32 | 3.98 |
|  | total | 792 | 3.85 | 4.212 | .150 | 3.55 | 4.14 |
| Job burnout score | 1 | 197 | 1.8702 | .97025 | .06913 | 1.7339 | 2.0066 |
|  | 2 | 595 | 1.8782 | 1.04834 | .04298 | 1.7938 | 1.9626 |
|  | total | 792 | 1.8762 | 1.02887 | .03656 | 1.8045 | 1.9480 |

| **description** | | | |
| --- | --- | --- | --- |
|  | | minima | maxima |
| Stress perception scale scores | 1 | 6.00 | 44.00 |
|  | 2 | 2.00 | 48.00 |
|  | total | 2.00 | 48.00 |
| Mental health score | 1 | 0 | 19 |
|  | 2 | 0 | 19 |
|  | total | 0 | 19 |
| Job burnout score | 1 | .15 | 4.88 |
|  | 2 | .00 | 6.00 |
|  | total | .00 | 6.00 |

| **ANOVA** | | | | | | |
| --- | --- | --- | --- | --- | --- | --- |
|  | | quadratic sum | df | mean square | F | significance |
| Stress perception scale score | Inter group | 172.211 | 1 | 172.211 | 2.938 | .087 |
|  | Within group | 46304.799 | 790 | 58.614 |  |  |
|  | total | 46477.010 | 791 |  |  |  |
| Mental health score | Inter group | 95.440 | 1 | 95.440 | 5.409 | .020 |
|  | Within group | 13938.378 | 790 | 17.644 |  |  |
|  | total | 14033.818 | 791 |  |  |  |
| Professional burnout score | Inter group | .009 | 1 | .009 | .009 | .925 |
|  | Within group | 837.329 | 790 | 1.060 |  |  |
|  | total | 837.339 | 791 |  |  |  |

CORRELATIONS

/ VARIABLES= Tension (stress perception) loss of control (coping perception) Stress perception Scale score mental health score emotional exhaustion mean score depersonalization mean score low personal accomplishment mean score job burnout score

/PRINT=TWOTAIL NOSIG

/STATISTICS DESCRIPTIVES

/MISSING=PAIRWISE.

**relativity**

| **notes appended to a book** | | |
| --- | --- | --- |
| The output created | | 14-July-2023 11:39:59 seconds |
| explanatory note | |  |
| import | data | F: \ submitted version \ data.sav |
|  | Datasets of activities | data set 1 |
|  | filter | <none> |
|  | weight | <none> |
|  | Split files | <none> |
|  | The N rows in the working data file | 792 |
| Missing value processing | Deletion definition | User-defined missing values are treated as missing. |
|  | The cases used | The statistics of each pair of variables are based on all cases with valid data for that pair of variables. |
| grammar | | CORRELATIONS  / VARIABLES= Tension (stress perception) loss of control (coping perception) Stress perception Scale score mental health score emotional exhaustion mean score depersonalization mean score low personal accomplishment mean score job burnout score/PRINT=TWOTAIL NOSIG  /STATISTICS DESCRIPTIVES  /MISSING=PAIRWISE. |
| resource | Processor time | 00 00:00:00.015 |
|  | Time used | 00 00:00:00.009 |

[Dataset 1] F: \ submitted version \ data.sav

| **Descriptive statistics** | | | |
| --- | --- | --- | --- |
|  | mean | standard error | N |
| Tension (stress perception) | 10.7235 | 4.48152 | 792 |
| Out of control (coping perception) | 11.9230 | 5.31871 | 792 |
| Stress perception scale score | 22.6465 | 7.66533 | 792 |
| Mental health score | 3.85 | 4.212 | 792 |
| Emotional exhaustion mean score | 2.0864 | 1.25730 | 792 |
| Depersonalization mean score | 1.4991 | 1.20802 | 792 |
| Low personal accomplishment mean score | 1.9733 | 1.47927 | 792 |
| Job burnout score | 1.8762 | 1.02887 | 792 |

| **relativity** | | | | |
| --- | --- | --- | --- | --- |
|  | | Tension (stress perception) | Out of control (coping perception) | Stress perception scale score |
| Tension (stress perception) | Pearson Correlation | 1 | .218^**^ | .736^**^ |
|  | Significance (Bilateral) |  | .000 | .000 |
|  | N | 792 | 792 | 792 |
| Out of control (coping perception) | Pearson Correlation | .218^**^ | 1 | .821^**^ |
|  | Significance (Bilateral) | .000 |  | .000 |
|  | N | 792 | 792 | 792 |
| Stress perception scale score | Pearson Correlation | .736^**^ | .821^**^ | 1 |
|  | Significance (Bilateral) | .000 | .000 |  |
|  | N | 792 | 792 | 792 |
| Mental health score | Pearson Correlation | .603^**^ | .332^**^ | .583^**^ |
|  | Significance (Bilateral) | .000 | .000 | .000 |
|  | N | 792 | 792 | 792 |
| Emotional exhaustion mean score | Pearson Correlation | .679^**^ | .281^**^ | .592^**^ |
|  | Significance (Bilateral) | .000 | .000 | .000 |
|  | N | 792 | 792 | 792 |
| Depersonalization mean score | Pearson Correlation | .543^**^ | .308^**^ | .531^**^ |
|  | Significance (Bilateral) | .000 | .000 | .000 |
|  | N | 792 | 792 | 792 |
| Low personal accomplishment mean score | Pearson Correlation | .194^**^ | .578^**^ | .515^**^ |
|  | Significance (Bilateral) | .000 | .000 | .000 |
|  | N | 792 | 792 | 792 |
| Job burnout score | Pearson Correlation | .607^**^ | .495^**^ | .699^**^ |
|  | Significance (Bilateral) | .000 | .000 | .000 |
|  | N | 792 | 792 | 792 |

| **relativity** | | | | |
| --- | --- | --- | --- | --- |
|  | | Mental health score | Emotional exhaustion mean score | Depersonalization mean score |
| Tension (stress perception) | Pearson Correlation | .603^**^ | .679^**^ | .543^**^ |
|  | Significance (Bilateral) | .000 | .000 | .000 |
|  | N | 792 | 792 | 792 |
| Out of control (coping perception) | Pearson Correlation | .332^**^ | .281^**^ | .308^**^ |
|  | Significance (Bilateral) | .000 | .000 | .000 |
|  | N | 792 | 792 | 792 |
| Stress perception scale score | Pearson Correlation | .583^**^ | .592^**^ | .531^**^ |
|  | Significance (Bilateral) | .000 | .000 | .000 |
|  | N | 792 | 792 | 792 |
| Mental health score | Pearson Correlation | 1 | .558^**^ | .507^**^ |
|  | Significance (Bilateral) |  | .000 | .000 |
|  | N | 792 | 792 | 792 |
| Emotional exhaustion mean score | Pearson Correlation | .558^**^ | 1 | .760^**^ |
|  | Significance (Bilateral) | .000 |  | .000 |
|  | N | 792 | 792 | 792 |
| Depersonalization mean score | Pearson Correlation | .507^**^ | .760^**^ | 1 |
|  | Significance (Bilateral) | .000 | .000 |  |
|  | N | 792 | 792 | 792 |
| Low personal accomplishment mean score | Pearson Correlation | .212^**^ | .197^**^ | .349^**^ |
|  | Significance (Bilateral) | .000 | .000 | .000 |
|  | N | 792 | 792 | 792 |
| Job burnout score | Pearson Correlation | .543^**^ | .841^**^ | .875^**^ |
|  | Significance (Bilateral) | .000 | .000 | .000 |
|  | N | 792 | 792 | 792 |

| **relativity** | | | |
| --- | --- | --- | --- |
|  | | Low personal accomplishment mean score | Job burnout score |
| Tension (stress perception) | Pearson Correlation | .194^**^ | .607^**^ |
|  | Significance (Bilateral) | .000 | .000 |
|  | N | 792 | 792 |
| Out of control (coping perception) | Pearson Correlation | .578^**^ | .495^**^ |
|  | Significance (Bilateral) | .000 | .000 |
|  | N | 792 | 792 |
| Stress perception scale score | Pearson Correlation | .515^**^ | .699^**^ |
|  | Significance (Bilateral) | .000 | .000 |
|  | N | 792 | 792 |
| Mental health score | Pearson Correlation | .212^**^ | .543^**^ |
|  | Significance (Bilateral) | .000 | .000 |
|  | N | 792 | 792 |
| Emotional exhaustion mean score | Pearson Correlation | .197^**^ | .841^**^ |
|  | Significance (Bilateral) | .000 | .000 |
|  | N | 792 | 792 |
| Depersonalization mean score | Pearson Correlation | .349^**^ | .875^**^ |
|  | Significance (Bilateral) | .000 | .000 |
|  | N | 792 | 792 |
| Low personal accomplishment mean score | Pearson Correlation | 1 | .651^**^ |
|  | Significance (Bilateral) |  | .000 |
|  | N | 792 | 792 |
| Job burnout score | Pearson Correlation | .651^**^ | 1 |
|  | Significance (Bilateral) | .000 |  |
|  | N | 792 | 792 |

|  |
| --- |
| **. In.01 significant correlation on level (bilateral). |

* Encoding: UTF-8.

/* PROCESS version 3.4.1 */.

/* Written by Andrew F.Hayes */.

/* www.afhayes.com */.

/* www.processmacro.org */.

/* Copyright 2020 by Andrew F.Hayes */.

/* Documented in http://www.guilford.com/p/hayes3 */.

/* PROCESS workshop schedule at http://www.processmacro.org/workshops.html */.

/* Distribution of this code in any form, except through processmacro.org, is prohibited */.

/* without the permission of the copyright holder */.

/* THIS SOFTWARE IS PROVIDED "AS IS", WITHOUT WARRANTY OF ANY KIND */.

/* EXPRESS OR IMPLIED, INCLUDING BUT NOT LIMITED TO THE WARRANTIES OF */.

/* MERCHANTABILITY, FITNESS FOR A PARTICULAR PURPOSE AND NONINFRINGEMENT */.

/* IN NO EVENT SHALL THE COPYRIGHT HOLDERS BE LIABLE FOR ANY CLAIM, */.

/* DAMAGES OR OTHER LIABILITY, WHETHER IN AN ACTION OF CONTRACT, TORT */.

/* OR OTHERWISE, ARISING FROM, OUT OF OR IN CONNECTION WITH THE */.

/* SOFTWARE OR THE USE OR OTHER DEALINGS IN THE SOFTWARE */.

/* USE OF THIS SOFTWARE IMPLIES AGREEMENT WITH THESE TERMS */.

set printback=off.

**matrix**

| **notes appended to a book** | | |
| --- | --- | --- |
| The output created | | 14-July-2023 11:40:05 seconds |
| explanatory note | |  |
| import | data | F: \ submitted version \ data.sav |
|  | Datasets of activities | data set 1 |
|  | filter | <none> |
|  | weight | <none> |
|  | Split files | <none> |
|  | The N rows in the working data file | 792 |
| grammar | | MATRIX.  compute wnames='xxxxx'.  compute znames='xxxxx'.  compute mcerpt=0.  compute wiscov=0.  compute ziscov=0.  compute errcode=make(100,1,0).  compute notecode=make(100,1,0).  compute model = trunc( 4 ).  compute iterate = abs(trunc( 100 )).  compute converge = abs( 0.00001 ).  compute itprobtg=0.  compute v2tag=0.  compute maxwwarn=0.  compute minwwarn=0.  compute maxzwarn=0.  compute minzwarn=0.  compute toomany=0.  compute wdich=0.  compute zdich=0.  compute wnotev=0.  compute znotev=0.  compute nxpval=1.  compute nwpval=1.  compute nzpval=1.  compute errs=1.  compute notes=1.  compute criterr=0.  compute novar=0.  compute adjust=0.  compute ncs=0.  compute serial=0.  compute sobelok=0.  compute hasw=0.  compute hasz=0.  compute printw=0.  compute printz=0.  compute counterf=0.  compute wmodcust=0.  compute zmodcust=0.  compute booting=0.  compute bootiter=0.  compute iterrmod=0.  compute cov = 'xxxxx'.  compute varorder=( 0 <> 0).  compute nws=0.  compute w= 'xxxxx'.  compute nzs=0.  compute z = 'xxxxx'.  compute nms=0.  compute m = 'Z mental health score'.  compute nys=0.  compute y = 'Z burnout score'.  compute nxs=0.  compute x = 'Z Stress Sense Scale score'.  compute v = 'xxxxx'.  compute q = 'xxxxx'.  compute oldvars= 'xxxxx'.  compute mcxok=0.  compute mcwok=0.  compute mczok=0.  compute xprod=0.  compute zprod=0.  compute wprod=0.  compute modcok=0.  compute hc3=trunc( 0 ).  compute jn=( 0 = 1).  compute effsize=( 1 =1).  compute normal=( 0 =1).  compute xmtest=( 0 =1).  compute stand=( 1 =1).  do if (stand=1).  compute effsize=1.  end if.  compute pstog=0.  compute sobelok=0.  compute normal=( 0 =1).  compute mdichok=( 0 =1).  compute contrast={ 999 }.  compute ncontr=ncol(contrast).  compute ncontrow=nrow(contrast).  do if (contrast(1,1) = 999).  compute ncontr=1.  compute contrast=0.  end if.  do if (ncontr = 1).  compute contrast=trunc(contrast).  do if (contrast > 2 or contrast < 0)).  compute ncontr=1.  compute contrast = 0.  end if.  end if.  do if (ncontr > 1).  compute contvec=contrast.  compute contrast=3.  do if (ncontrow > 1).  compute contrast=0.  compute modcok=1.  compute wcontval=contvec(:,1).  compute zcontval=contvec(:,2).  do if ((ncontr <> 2) or (ncontrow <> 2)).  compute notecode(notes,1) = 19.  compute notes = notes + 1.  compute modcok=0.  end if.  end if.  end if.  do if (varorder = 1).  compute notecode(notes,1) = 21.  compute notes = notes + 1.  end if.  do if ( 999 <> 999 or 999 <> 999).  compute notecode(notes,1) = 22.  compute notes = notes + 1.  end if.  compute modelbt=( 1 =1).  compute cluster= 'xxxxx'.  compute matrices=( 0 =1).  compute covcoeff=( 0 =1).  compute covmy=trunc( 0 ).  do if (covmy < 0 or covmy > 2).  compute covmy = 0.  end if.  compute boot = abs(trunc( 5000 )).  compute mc=abs(trunc( 0 )).  compute hc=trunc( 5 ).  compute intprobe = .1.  do if (intprobe < 0 or intprobe > 1).  compute intprobe = .10.  end if.  compute plot=trunc( 0 ).  do if (plot < 0 or plot > 2).  compute plot=0.  end if.  compute total=( 1 =1).  compute dototal=0.  compute saveboot = ( 0 = 1).  compute saveest=( 0 = 2).  do if (hc >= 0 and hc < 5).  compute notecode(notes,1) = 4.  compute notes = notes + 1.  end if.  do if (hc > 5 or hc < 0).  compute hc=5.  end if.  compute mcw=trunc( 0 ).  compute mcz=trunc( 0 ).  compute mcx=trunc( 0 ).  do if (mcx > 0 and model = 74).  compute mcw=mcx.  end if.  do if (mcw > 0 and model = 74).  compute mcx=mcw.  end if.  do if (mcx > 0 and contrast > 0).  compute notecode(notes,1) = 28.  compute notes = notes + 1.  compute contrast=0.  end if.  compute nxvls=1.  compute nmvls=1.  compute nwvls=1.  compute nzvls=1.  compute paths=999.  compute pathsw=999.  compute pathsz=999.  compute pathswz=999.  compute pathsmod=999.  compute pathtype=999.  compute obscoeff=999.  compute pathsdv={' '}.  compute quantile=1.  do if ( 999 <>999).  compute notecode(notes,1) = 23.  compute notes = notes + 1.  end if.  compute moments=( 0 =1).  do if (moments=1).  compute quantile=0.  end if.  compute center=trunc( 1 ).  compute bmatrix={ -999 }.  compute wmatrix={ -999 }.  compute zmatrix={ -999 }.  compute wzmatrix={ -999 }.  compute cmatrix={ -999 }.  compute xcatcode={ -999 }.  compute wcatcode={ -999 }.  compute zcatcode={ -999 }.  compute needed=0.  compute conf= 95.  do if (trunc( 95 ) >= 100 or (trunc( 95 ) <= 50)).  compute conf = 95.  compute notecode(notes,1)=2.  compute notes=notes+1.  end if.  do if (model > 0 and model < 4 and modelbt=0).  compute boot=0.  compute mc=0.  end if.  do if (boot > 0 and mc > 0).  compute boot=0.  end if.  compute p0=-.322232431088.  compute p1 = -1.  compute p2 = -.342242088547.  compute p3 = -.0204231210245.  compute p4 = -.0000453642210148.  compute q0 = .0993484626060.  compute q1 = .588581570495.  compute q2 = .531103462366.  compute q3 = .103537752850.  compute q4 = .0038560700634.  compute alpha2 = (1-(conf/100))/2.  compute cilm=alpha2*2.  compute y5=sqrt(-2*ln(alpha2)).  compute xp2=(y5+((((y5*p4+p3)*y5+p2)*y5+p1)*y5+p0)/((((y5*q4+q3)*y5+q2)*y5+q1)*y5+q0)).  compute medlb={' M1 :';' M2 :';' M3 :';' M4 :';' M5 :';' M6 :';' M7 :';' M8 :';' M9 :';' M10 :'}.  compute medlb2={'(M1)','(M2)','(M3)','(M4)','(M5)','(M6)','(M7)','(M8)','(M9)','(M10)'}.  compute xlb={' X1 :';' X2 :';' X3 :';' X4 :';' X5 :';' X6 :';' X7 :';' X8 :';' X9 :'}.  compute highlbw={'M1*W'; 'M2*W'; 'M3*W'; 'M4*W'; 'M5*W'; 'M6*W'; 'M7*W'; 'M8*W'; 'M9*W'; 'M10*W'}.  compute highlbz={'M1*Z'; 'M2*Z'; 'M3*Z'; 'M4*Z'; 'M5*Z'; 'M6*Z'; 'M7*Z'; 'M8*Z'; 'M9*Z';'M10*Z'}.  compute highlbwz={'M1*W*Z'; 'M2*W*Z'; 'M3*W*Z'; 'M4*W*Z'; 'M5*W*Z'; 'M6*W*Z'; 'M7*W*Z'; 'M8*W*Z'; 'M9*W*Z';'M10*W*Z'}.  compute highlbbt={'BOTH(M1)'; 'BOTH(M2)'; 'BOTH(M3)'; 'BOTH(M4)'; 'BOTH(M5)'; 'BOTH(M6)'; 'BOTH(M7)'; 'BOTH(M8)'; 'BOTH(M9)';'BTH(M10)'}.  compute highlbx={'M1*X'; 'M2*X'; 'M3*X'; 'M4*X'; 'M5*X'; 'M6*X'; 'M7*X'; 'M8*X'; 'M9*X'; 'M10*X'}.  compute skipwz=0.  compute validm={1,1,1,1,1,1,1,1,1,1,1,1,1,1,1,1,1,1,1,1,1,1,0,0,0,0,0,1,1,0,0,0,0, 0,0,0,0,0,0,0,0,0,0,0,0,0,0,0,0,0,0,0,0,0,0,0,0,1,1,1,1,1,1,1,1,1,1,1,1,1,1,1,1,0,1,1,0,0,0,1,1, 1,1,1,1,1,1,1,1,1,1,1}.  do if (( 0 =1)=1).  compute errcode(errs,1)=42.  compute errs=errs+1.  compute criterr=1.  end if.  do if (model > 0 and model < 93).  do if (validm(1,model)=0).  do if (model <> 74).  compute errcode(errs,1)=6.  compute errs=errs+1.  compute criterr=1.  end if.  do if (model = 74).  compute errcode(errs,1)=46.  compute errs=errs+1.  compute criterr=1.  end if.  end if.  release validm.  end if.  do if ((model > 92 or model < 0) and model <> 999)).  compute errcode(errs,1)=7.  compute errs=errs+1.  compute criterr=1.  end if.  do if (model = 999 and bmatrix(1,1)=-999).  compute errcode(errs,1)=24.  compute errs=errs+1.  compute criterr=1.  end if.  do if (model <> 999 and bmatrix(1,1) <> -999).  compute errcode(errs,1)=25.  compute errs=errs+1.  compute criterr=1.  end if.  do if ((model = 74 or (model > 0 and model < 4)) and ((wmatrix(1,1) <> -999) or (zmatrix(1,1)<>-999) or (wzmatrix(1,1)<>-999)))).  compute errcode(errs,1)=41.  compute errs=errs+1.  compute criterr=1.  end if.  do if (hc3 <> 0).  compute notecode(notes,1) = 5.  compute notes = notes + 1.  do if (hc3 = 1).  compute hc=3.  end if.  end if.  do if ((v <> 'xxxxx') or (q <> 'xxxxx')).  compute errcode(errs,1)=14.  compute errs=errs+1.  compute errcode(errs,1)=48.  compute errs=errs+1.  compute criterr=1.  end if.  do if (oldvars <> 'xxxxx').  compute errcode(errs,1)=48.  compute errs=errs+1.  compute criterr=1.  end if.  do if (cluster <> 'xxxxx').  compute errcode(errs,1)=27.  compute errs=errs+1.  compute criterr=1.  end if.  do if ((y = 'xxxxx') or (x = 'xxxxx')).  compute errcode(errs,1)=1.  compute errs=errs+1.  compute criterr=1.  end if.  do if ((m = 'xxxxx') and model > 3).  compute errcode(errs,1)=8.  compute errs=errs+1.  compute criterr=1.  end if.  do if (criterr=0).  get ytmp / variables = Z Burnout score / names = ynames / MISSING = 99999.  compute nys=ncol(ytmp).  compute needed=nys.  compute n=nrow(ytmp).  compute varnames={ynames}.  compute dat=ytmp.  .  compute toomany=1.  .  compute modelvar={ '4' ;t(ynames)}.  do if ( 4 =999).  compute modelvar(1,1)='CUSTOM'.  end if.  get xtmp / variables = Z Pressure Perception Scale score / names = xnames / MISSING = 99999.  compute nxs=ncol(xtmp).  compute n=nrow(xtmp).  compute needed=needed+nxs.  compute varnames={varnames,xnames}.  compute xcatlab=t(xnames).  compute dat={dat,xtmp}.  .  compute toomany=1.  .  compute modelvar={modelvar;t(xnames)}.  do if (nxs = 1).  compute modelvlb={'Model :';' Y :';' X :'}.  else.  compute modelvlb={'Model :';' Y :';xlb(1:nxs,1)}.  end if.  do if (m <> 'xxxxx').  get mtmp / variables = Z mental health score / names = mnames / MISSING = 99999.  compute nms=ncol(mtmp).  compute mprod=make(1,nms,0).  compute n=nrow(mtmp).  compute needed=needed+nms.  compute varnames={varnames,mnames}.  compute dat={dat,mtmp}.  compute modelvar={modelvar;t(mnames)}.  compute x2m=make(99,nms,0).  compute m2y=make(99,nms,0).  compute onem=make(nms,1,1).  .  compute toomany=1.  .  do if (nms > 1 and nms < 11).  compute modelvlb={modelvlb;medlb(1:nms,1)}.  else.  compute modelvlb={modelvlb;' M :'}.  end if.  do if (nms > 0 and model < 4).  compute errcode(errs,1)=9.  compute errs=errs+1.  compute errcode(errs,1)=48.  compute errs=errs+1.  compute criterr=1.  end if.  end if.  compute wlocatet=0.  compute wlocate=0.  do if (w <> 'xxxxx').  get wtmp/variables = xxxxx /names = wnames/MISSING = 99999.  compute nws=ncol(wtmp).  compute n=nrow(wtmp).  .  .  compute varnames={varnames,wnames}.  compute wlocate=ncol(varnames).  do if (model=74).  compute wlocatet=1.  do if (xnames <> wnames).  compute errcode(errs,1)=45.  compute errs=errs+1.  compute criterr=1.  end if.  end if.  compute wcatlab=t(wnames).  compute dat={dat,wtmp}.  compute modelvar={modelvar;t(wnames)}.  compute modelvlb={modelvlb;' W :'}.  end if.  do if (z <> 'xxxxx').  get ztmp/variables = xxxxx /names = znames/MISSING = 99999.  compute nzs=ncol(ztmp).  compute n=nrow(ztmp).  .  .  compute varnames={varnames,znames}.  compute zcatlab=t(znames).  compute dat={dat,ztmp}.  compute modelvar={modelvar;t(znames)}.  compute modelvlb={modelvlb;' Z :'}.  end if.  do if (cov <> 'xxxxx').  get ctmp/variables = xxxxx /names = covnames/MISSING = 99999.  compute ncs=ncol(ctmp).  compute n=nrow(ctmp).  .  .  compute varnames={varnames,covnames}.  compute dat={dat,ctmp}.  end if.  do if (nws > 1 or nzs > 1 or nys > 1 or nxs > 1).  compute errcode(errs,1)=3.  compute errs=errs+1.  compute criterr=1.  end if.  do if ((model = 80 or model = 81) and (nms < 3 or nms > 6)).  compute errcode(errs,1)=32.  compute errs=errs+1.  compute criterr=1.  end if.  do if (model = 82 and nms <> 4).  compute errcode(errs,1)=33.  compute errs=errs+1.  compute criterr=1.  end if.  do if (nms > 10).  compute errcode(errs,1)=37.  compute errs=errs+1.  compute criterr=1.  end if.  do if ((model = 6 or (model > 82 and model < 999)) and (nms < 2 or nms > 6)).  compute errcode(errs,1)=34.  compute errs=errs+1.  compute criterr=1.  end if.  compute match=0.  compute match2=0.  compute mcwzcov=0.  loop i = 1 to (ncol(varnames)-1).  loop j = (i+1) to ncol(varnames).  do if (varnames(i)=varnames(j)).  do if (i < (nxs+nms+nys+1)).  compute match2=match2+1.  end if.  do if (wlocatet=1 and i=2 and j=wlocate).  compute match2=match2-1.  end if.  do if ((wnames=znames) and (nws > 0 or nzs > 0))).  compute match2=match2+1.  end if.  do if (i < (ncol(varnames)-ncs+1)) and j > (ncol(varnames)-ncs)).  do if ((varnames(j)=wnames) and mcw=0)).  compute match=0.  compute wiscov=(j-(ncol(varnames)-ncs)).  end if.  do if ((varnames(j)=wnames) and mcw <>0)).  compute mcwzcov=1.  end if.  do if ((varnames(j)=znames) and mcz=0).  compute match=0.  compute ziscov=(j-(ncol(varnames)-ncs)).  end if.  do if ((varnames(j)=znames) and mcz<>0)).  compute mcwzcov=1.  end if.  end if.  end if.  end loop.  end loop.  do if (match2>0 or match=1).  compute errcode(errs,1)=2.  compute errs=errs+1.  compute criterr=1.  end if.  do if (mcwzcov=1).  compute errcode(errs,1)=50.  compute errs=errs+1.  compute criterr=1.  end if.  compute ninit=nrow(dat).  compute rownum=make(ninit,1,0).  loop i = 1 to ninit.  compute rownum(i,1)=i.  end loop.  compute dat={rownum,dat}.  compute j=1.  compute missrow=0.  loop i = 1 to n.  do if (rsum(dat(i,2:ncol(dat))=99999)=0).  compute dat(j,:)=dat(i,:).  compute j=j+1.  else.  compute missrow={missrow;dat(i,1)}.  end if.  end loop.  compute rownum=dat(1:(j-1),1).  do if (nrow(missrow) > 1).  compute missrow=t(missrow(2:nrow(missrow),1)).  end if.  compute dat=dat(1:(j-1),2:ncol(dat)).  compute n=nrow(dat).  compute nmiss=ninit-n.  compute ytmp=dat(:,1:nys).  .  compute desctmp=make((8-(4* 0 )),ncol( ytmp ),-999).  loop jd=1 to ncol( ytmp ).  compute descdat= ytmp (:,jd).  compute desctmp(1,jd) = csum(descdat)/nrow(descdat).  compute desctmp(2,jd) = (nrow(descdat)*sscp(descdat))-(t(csum(descdat))*(csum(descdat))).  compute desctmp(2,jd) = sqrt(desctmp(2,jd)/(nrow(descdat)*(nrow(descdat)-1))).  compute desctmp(3,jd)=cmin(descdat).  compute desctmp(4,jd)=cmax(descdat).  do if ( 0 =0).  compute minwarn=0.  compute maxwarn=0.  do if ((desctmp(3,jd)=desctmp(4,jd)) and novar=0).  compute errcode(errs,1)=15.  compute errs=errs+1.  compute criterr=1.  compute novar=1.  end if.  compute tmp=((descdat(:,1)=desctmp(3,jd))+(descdat(:,1)=desctmp(4,jd))).  compute desctmp(8,jd)=(csum(tmp)=nrow(tmp)).  compute tmp = descdat.  compute tmp(GRADE(descdat),:) = descdat.  compute descdat = tmp.  release tmp.  compute decval={.16;.5;.84}.  loop kd=1 to 3.  compute low=trunc(decval(kd,1)*(nrow(descdat)+1)).  compute lowdec=decval(kd,1)*(nrow(descdat)+1)-low.  compute value=descdat(low,1)+(descdat((low+1),1)-descdat(low,1))*lowdec.  compute desctmp((4+kd),jd)=value.  end loop.  compute mnotev=1.  compute modvals=desctmp(5:7,:).  do if (quantile <> 1).  compute desctmp(5,jd)=desctmp(1,jd)-desctmp(2,jd).  compute desctmp(6,jd)=desctmp(1,jd).  compute desctmp(7,jd)=desctmp(1,jd)+desctmp(2,jd).  compute modvals=desctmp(5:7,:).  compute mnotev=2.  do if (modvals(1,1) < desctmp(3,1)).  compute modvals(1,1)=desctmp(3,1).  compute minwarn=1.  end if.  do if (modvals(3,1) > desctmp(4,1)).  compute modvals(3,1)=desctmp(4,1).  compute maxwarn=1.  end if.  end if.  do if (desctmp(8,1)=1).  compute modvals={desctmp(3,1);desctmp(4,1)}.  compute mnotev=0.  compute minwarn=0.  compute maxwarn=0.  end if.  end if.  end loop  .  compute ysd=desctmp(2,:).  compute ovsd=ysd.  compute ydich=0.  do if (desctmp(8,1)=1).  compute ydich=1.  do if (total=1).  compute total=0.  compute notecode(notes,1) = 24.  compute notes = notes + 1.  end if.  do if (effsize=1).  compute effsize=0.  compute notecode(notes,1) = 25.  compute notes = notes + 1.  end if.  compute omx = cmax(ytmp).  compute omn = cmin(ytmp).  compute ytmp = (ytmp = omx).  compute dat(:,1:nys)=(dat(:,1:nys)=omx).  compute rcd = {omn, 0; omx, 1}.  end if.  compute xtmp=dat(:,(nys+1):(nys+nxs)).  .  compute desctmp=make((8-(4* 0 )),ncol( xtmp ),-999).  loop jd=1 to ncol( xtmp ).  compute descdat= xtmp (:,jd).  compute desctmp(1,jd) = csum(descdat)/nrow(descdat).  compute desctmp(2,jd) = (nrow(descdat)*sscp(descdat))-(t(csum(descdat))*(csum(descdat))).  compute desctmp(2,jd) = sqrt(desctmp(2,jd)/(nrow(descdat)*(nrow(descdat)-1))).  compute desctmp(3,jd)=cmin(descdat).  compute desctmp(4,jd)=cmax(descdat).  do if ( 0 =0).  compute minwarn=0.  compute maxwarn=0.  do if ((desctmp(3,jd)=desctmp(4,jd)) and novar=0).  compute errcode(errs,1)=15.  compute errs=errs+1.  compute criterr=1.  compute novar=1.  end if.  compute tmp=((descdat(:,1)=desctmp(3,jd))+(descdat(:,1)=desctmp(4,jd))).  compute desctmp(8,jd)=(csum(tmp)=nrow(tmp)).  compute tmp = descdat.  compute tmp(GRADE(descdat),:) = descdat.  compute descdat = tmp.  release tmp.  compute decval={.16;.5;.84}.  loop kd=1 to 3.  compute low=trunc(decval(kd,1)*(nrow(descdat)+1)).  compute lowdec=decval(kd,1)*(nrow(descdat)+1)-low.  compute value=descdat(low,1)+(descdat((low+1),1)-descdat(low,1))*lowdec.  compute desctmp((4+kd),jd)=value.  end loop.  compute mnotev=1.  compute modvals=desctmp(5:7,:).  do if (quantile <> 1).  compute desctmp(5,jd)=desctmp(1,jd)-desctmp(2,jd).  compute desctmp(6,jd)=desctmp(1,jd).  compute desctmp(7,jd)=desctmp(1,jd)+desctmp(2,jd).  compute modvals=desctmp(5:7,:).  compute mnotev=2.  do if (modvals(1,1) < desctmp(3,1)).  compute modvals(1,1)=desctmp(3,1).  compute minwarn=1.  end if.  do if (modvals(3,1) > desctmp(4,1)).  compute modvals(3,1)=desctmp(4,1).  compute maxwarn=1.  end if.  end if.  do if (desctmp(8,1)=1).  compute modvals={desctmp(3,1);desctmp(4,1)}.  compute mnotev=0.  compute minwarn=0.  compute maxwarn=0.  end if.  end if.  end loop  .  compute xsd=desctmp(2,:).  compute xmodvals=modvals.  compute nxpval=nrow(xmodvals).  compute xprobval=xmodvals.  compute xdich=desctmp(8,1).  do if (xdich =1 and mcx > 0).  compute mcx=0.  compute errcode(errs,1) = 52.  compute errs = errs + 1.  compute criterr = 1.  end if.  do if (model = 74 and xdich=1).  compute counterf=1.  end if.  do if (nms > 0).  compute mtmp=dat(:,(nys+nxs+1):(nys+nxs+nms)).  .  compute desctmp=make((8-(4* 0 )),ncol( mtmp ),-999).  loop jd=1 to ncol( mtmp ).  compute descdat= mtmp (:,jd).  compute desctmp(1,jd) = csum(descdat)/nrow(descdat).  compute desctmp(2,jd) = (nrow(descdat)*sscp(descdat))-(t(csum(descdat))*(csum(descdat))).  compute desctmp(2,jd) = sqrt(desctmp(2,jd)/(nrow(descdat)*(nrow(descdat)-1))).  compute desctmp(3,jd)=cmin(descdat).  compute desctmp(4,jd)=cmax(descdat).  do if ( 0 =0).  compute minwarn=0.  compute maxwarn=0.  do if ((desctmp(3,jd)=desctmp(4,jd)) and novar=0).  compute errcode(errs,1)=15.  compute errs=errs+1.  compute criterr=1.  compute novar=1.  end if.  compute tmp=((descdat(:,1)=desctmp(3,jd))+(descdat(:,1)=desctmp(4,jd))).  compute desctmp(8,jd)=(csum(tmp)=nrow(tmp)).  compute tmp = descdat.  compute tmp(GRADE(descdat),:) = descdat.  compute descdat = tmp.  release tmp.  compute decval={.16;.5;.84}.  loop kd=1 to 3.  compute low=trunc(decval(kd,1)*(nrow(descdat)+1)).  compute lowdec=decval(kd,1)*(nrow(descdat)+1)-low.  compute value=descdat(low,1)+(descdat((low+1),1)-descdat(low,1))*lowdec.  compute desctmp((4+kd),jd)=value.  end loop.  compute mnotev=1.  compute modvals=desctmp(5:7,:).  do if (quantile <> 1).  compute desctmp(5,jd)=desctmp(1,jd)-desctmp(2,jd).  compute desctmp(6,jd)=desctmp(1,jd).  compute desctmp(7,jd)=desctmp(1,jd)+desctmp(2,jd).  compute modvals=desctmp(5:7,:).  compute mnotev=2.  do if (modvals(1,1) < desctmp(3,1)).  compute modvals(1,1)=desctmp(3,1).  compute minwarn=1.  end if.  do if (modvals(3,1) > desctmp(4,1)).  compute modvals(3,1)=desctmp(4,1).  compute maxwarn=1.  end if.  end if.  do if (desctmp(8,1)=1).  compute modvals={desctmp(3,1);desctmp(4,1)}.  compute mnotev=0.  compute minwarn=0.  compute maxwarn=0.  end if.  end if.  end loop  .  compute ovsd={desctmp(2,:),ysd}.  do if ((rsum(desctmp(8,:))>0) and (mdichok <> 1)).  compute errcode(errs,1)=43.  compute errs=errs+1.  compute criterr=1.  end if.  compute mmodvals=modvals.  compute mprobval=mmodvals.  end if.  do if (nws > 0).  compute wtmp=dat(:,(nys+nxs+nms+1):(nys+nxs+nms+nws)).  .  compute desctmp=make((8-(4* 0 )),ncol( wtmp ),-999).  loop jd=1 to ncol( wtmp ).  compute descdat= wtmp (:,jd).  compute desctmp(1,jd) = csum(descdat)/nrow(descdat).  compute desctmp(2,jd) = (nrow(descdat)*sscp(descdat))-(t(csum(descdat))*(csum(descdat))).  compute desctmp(2,jd) = sqrt(desctmp(2,jd)/(nrow(descdat)*(nrow(descdat)-1))).  compute desctmp(3,jd)=cmin(descdat).  compute desctmp(4,jd)=cmax(descdat).  do if ( 0 =0).  compute minwarn=0.  compute maxwarn=0.  do if ((desctmp(3,jd)=desctmp(4,jd)) and novar=0).  compute errcode(errs,1)=15.  compute errs=errs+1.  compute criterr=1.  compute novar=1.  end if.  compute tmp=((descdat(:,1)=desctmp(3,jd))+(descdat(:,1)=desctmp(4,jd))).  compute desctmp(8,jd)=(csum(tmp)=nrow(tmp)).  compute tmp = descdat.  compute tmp(GRADE(descdat),:) = descdat.  compute descdat = tmp.  release tmp.  compute decval={.16;.5;.84}.  loop kd=1 to 3.  compute low=trunc(decval(kd,1)*(nrow(descdat)+1)).  compute lowdec=decval(kd,1)*(nrow(descdat)+1)-low.  compute value=descdat(low,1)+(descdat((low+1),1)-descdat(low,1))*lowdec.  compute desctmp((4+kd),jd)=value.  end loop.  compute mnotev=1.  compute modvals=desctmp(5:7,:).  do if (quantile <> 1).  compute desctmp(5,jd)=desctmp(1,jd)-desctmp(2,jd).  compute desctmp(6,jd)=desctmp(1,jd).  compute desctmp(7,jd)=desctmp(1,jd)+desctmp(2,jd).  compute modvals=desctmp(5:7,:).  compute mnotev=2.  do if (modvals(1,1) < desctmp(3,1)).  compute modvals(1,1)=desctmp(3,1).  compute minwarn=1.  end if.  do if (modvals(3,1) > desctmp(4,1)).  compute modvals(3,1)=desctmp(4,1).  compute maxwarn=1.  end if.  end if.  do if (desctmp(8,1)=1).  compute modvals={desctmp(3,1);desctmp(4,1)}.  compute mnotev=0.  compute minwarn=0.  compute maxwarn=0.  end if.  end if.  end loop  .  compute wmodvals=modvals.  compute wdich=desctmp(8,1).  do if (wdich =1 and mcw > 0).  compute mcw=0.  compute errcode(errs,1) = 52.  compute errs = errs + 1.  compute criterr = 1.  end if.  compute wmin=desctmp(3,1).  compute wmax=desctmp(4,1).  compute minwwarn=minwarn.  compute maxwwarn=maxwarn.  compute wnotev=mnotev.  compute wmodval={ 999 }.  compute nwcontr=ncol(wmodval).  do if (wmodval(1,1) <> 999).  compute wmodvals=wmodval(1,1).  compute wmodcust=1.  do if (nwcontr > 1).  compute wmodvals=t(wmodval).  end if.  compute minwwarn=0.  compute maxwwarn=0.  compute wnotev=0.  end if.  compute wprobval=wmodvals.  compute nwpval=nrow(wmodvals).  end if.  do if (nzs > 0).  compute ztmp=dat(:,(nys+nxs+nms+nws+1):(nys+nxs+nms+nws+nzs)).  .  compute desctmp=make((8-(4* 0 )),ncol( ztmp ),-999).  loop jd=1 to ncol( ztmp ).  compute descdat= ztmp (:,jd).  compute desctmp(1,jd) = csum(descdat)/nrow(descdat).  compute desctmp(2,jd) = (nrow(descdat)*sscp(descdat))-(t(csum(descdat))*(csum(descdat))).  compute desctmp(2,jd) = sqrt(desctmp(2,jd)/(nrow(descdat)*(nrow(descdat)-1))).  compute desctmp(3,jd)=cmin(descdat).  compute desctmp(4,jd)=cmax(descdat).  do if ( 0 =0).  compute minwarn=0.  compute maxwarn=0.  do if ((desctmp(3,jd)=desctmp(4,jd)) and novar=0).  compute errcode(errs,1)=15.  compute errs=errs+1.  compute criterr=1.  compute novar=1.  end if.  compute tmp=((descdat(:,1)=desctmp(3,jd))+(descdat(:,1)=desctmp(4,jd))).  compute desctmp(8,jd)=(csum(tmp)=nrow(tmp)).  compute tmp = descdat.  compute tmp(GRADE(descdat),:) = descdat.  compute descdat = tmp.  release tmp.  compute decval={.16;.5;.84}.  loop kd=1 to 3.  compute low=trunc(decval(kd,1)*(nrow(descdat)+1)).  compute lowdec=decval(kd,1)*(nrow(descdat)+1)-low.  compute value=descdat(low,1)+(descdat((low+1),1)-descdat(low,1))*lowdec.  compute desctmp((4+kd),jd)=value.  end loop.  compute mnotev=1.  compute modvals=desctmp(5:7,:).  do if (quantile <> 1).  compute desctmp(5,jd)=desctmp(1,jd)-desctmp(2,jd).  compute desctmp(6,jd)=desctmp(1,jd).  compute desctmp(7,jd)=desctmp(1,jd)+desctmp(2,jd).  compute modvals=desctmp(5:7,:).  compute mnotev=2.  do if (modvals(1,1) < desctmp(3,1)).  compute modvals(1,1)=desctmp(3,1).  compute minwarn=1.  end if.  do if (modvals(3,1) > desctmp(4,1)).  compute modvals(3,1)=desctmp(4,1).  compute maxwarn=1.  end if.  end if.  do if (desctmp(8,1)=1).  compute modvals={desctmp(3,1);desctmp(4,1)}.  compute mnotev=0.  compute minwarn=0.  compute maxwarn=0.  end if.  end if.  end loop  .  compute zmodvals=modvals.  compute zdich=desctmp(8,1).  do if (zdich =1 and mcz > 0).  compute mcz=0.  compute errcode(errs,1) = 52.  compute errs = errs + 1.  compute criterr = 1.  end if.  compute zmin=desctmp(3,1).  compute zmax=desctmp(4,1).  compute minzwarn=minwarn.  compute maxzwarn=maxwarn.  compute znotev=mnotev.  compute zmodval={ 999 }.  compute nzcontr=ncol(zmodval).  do if (zmodval(1,1) <> 999).  compute zmodvals=zmodval(1,1).  compute zmodcust=1.  do if (nzcontr > 1).  compute zmodvals=t(zmodval).  end if.  compute minzwarn=0.  compute maxzwarn=0.  compute znotev=0.  end if.  compute zprobval=zmodvals.  compute nzpval=nrow(zmodvals).  end if.  do if (ncs > 0).  compute ctmp=dat(:,(nys+nxs+nms+nws+nzs+1):(nys+nxs+nms+nws+nzs+ncs)).  .  compute desctmp=make((8-(4* 0 )),ncol( ctmp ),-999).  loop jd=1 to ncol( ctmp ).  compute descdat= ctmp (:,jd).  compute desctmp(1,jd) = csum(descdat)/nrow(descdat).  compute desctmp(2,jd) = (nrow(descdat)*sscp(descdat))-(t(csum(descdat))*(csum(descdat))).  compute desctmp(2,jd) = sqrt(desctmp(2,jd)/(nrow(descdat)*(nrow(descdat)-1))).  compute desctmp(3,jd)=cmin(descdat).  compute desctmp(4,jd)=cmax(descdat).  do if ( 0 =0).  compute minwarn=0.  compute maxwarn=0.  do if ((desctmp(3,jd)=desctmp(4,jd)) and novar=0).  compute errcode(errs,1)=15.  compute errs=errs+1.  compute criterr=1.  compute novar=1.  end if.  compute tmp=((descdat(:,1)=desctmp(3,jd))+(descdat(:,1)=desctmp(4,jd))).  compute desctmp(8,jd)=(csum(tmp)=nrow(tmp)).  compute tmp = descdat.  compute tmp(GRADE(descdat),:) = descdat.  compute descdat = tmp.  release tmp.  compute decval={.16;.5;.84}.  loop kd=1 to 3.  compute low=trunc(decval(kd,1)*(nrow(descdat)+1)).  compute lowdec=decval(kd,1)*(nrow(descdat)+1)-low.  compute value=descdat(low,1)+(descdat((low+1),1)-descdat(low,1))*lowdec.  compute desctmp((4+kd),jd)=value.  end loop.  compute mnotev=1.  compute modvals=desctmp(5:7,:).  do if (quantile <> 1).  compute desctmp(5,jd)=desctmp(1,jd)-desctmp(2,jd).  compute desctmp(6,jd)=desctmp(1,jd).  compute desctmp(7,jd)=desctmp(1,jd)+desctmp(2,jd).  compute modvals=desctmp(5:7,:).  compute mnotev=2.  do if (modvals(1,1) < desctmp(3,1)).  compute modvals(1,1)=desctmp(3,1).  compute minwarn=1.  end if.  do if (modvals(3,1) > desctmp(4,1)).  compute modvals(3,1)=desctmp(4,1).  compute maxwarn=1.  end if.  end if.  do if (desctmp(8,1)=1).  compute modvals={desctmp(3,1);desctmp(4,1)}.  compute mnotev=0.  compute minwarn=0.  compute maxwarn=0.  end if.  end if.  end loop  .  end if.  compute n=nrow(ytmp).  compute ones=make(n,1,1).  release dat.  do if (nws > 0 and mcw > 0).  compute tmp={rownum,wtmp(:,1)}.  .  compute dd= tmp.  compute temp = dd.  compute temp(GRADE(dd(:,2)),:) = dd.  compute dd = temp.  compute dummy = design(dd(:,2)).  compute nvls = ncol(dummy).  compute nnvls = csum(dummy).  compute mnvls = cmin(t(nnvls)).  compute conmat1=1.  do if (mnvls < 2).  compute errcode(errs,1) = 5.  compute errs = errs + 1.  compute criterr = 1.  end if.  do if (nvls > 9).  compute errcode(errs,1) = 4.  compute errs = errs+1.  compute criterr = 1.  end if.  do if (criterr = 0).  compute dumok = 1.  compute nnvls=make(nvls,1,0).  compute nnvls(1,1)=dd(1,2).  compute temp = 2.  loop i = 2 to n.  do if (dd(i,2) <> nnvls((temp-1),1)).  compute nnvls(temp,1)=dd(i,2).  compute temp = temp+1.  end if.  end loop.  do if ( mcw > 0).  compute x = dummy(:,2:ncol(dummy)).  compute nx = ncol(x).  compute minus1 = make(1,ncol(x),-1).  compute xdes=make((nx+1),3,0).  compute xdes(1,1)=dd(1,2).  compute xdes(1,2)=1.  compute temp = 2.  loop k = 2 to n.  do if (dd(k,2) <> dd((k-1),2)).  compute xdes(temp,2) = k.  compute xdes(temp,1) = dd(k,2).  compute xdes((temp-1),3) = k-1.  compute temp=temp+1.  end if.  end loop.  compute xdes((temp-1),3)=n.  compute xdes = {xdes, (xdes(:,3)-xdes(:,2)+1)}.  do if ( mcw = 4).  loop k = 1 to n.  do if (rsum(x(k,:)) = 0).  compute x(k,:) = minus1.  end if.  end loop.  end if.  do if ( mcw = 2 or mcw = 3 or mcw =5).  loop k = 1 to n.  do if (rsum(x(k,:)) > 0).  loop i = 1 to ncol(x).  do if (x(k,i) = 0).  compute x(k,i) = 1.  else.  break.  end if.  end loop.  end if.  end loop.  do if ( mcw = 3).  compute conmat1={-8,1,1,1,1,1,1,1,1; 0,-7,1,1,1,1,1,1,1; 0,0,-6,1,1,1,1,1,1; 0,0,0,-5,1,1,1,1,1; 0,0,0,0,-4,1,1,1,1; 0,0,0,0,0,-3,1,1,1; 0,0,0,0,0,0,-2,1,1; 0,0,0,0,0,0,0,-1,1}.  loop i = 1 to 8.  compute conmat1(i,:)=conmat1(i,:)/(10-i).  end loop.  compute conmat1=t(conmat1((10-nvls):8,(10-nvls):9)).  loop k=1 to n.  compute x(k,:)=conmat1((rsum(x(k,:))+1),:).  end loop.  end if.  end if.  do if ( mcw = 5).  compute custcode={ -999 }.  do if (ncol(custcode) <> (nvls*(nvls-1))).  compute errcode(errs,1) = (37+ 2 ).  compute errs = errs + 1.  compute criterr = 1.  end if.  do if (ncol(custcode) = (nvls*(nvls-1))).  compute conmat1=make(nvls,(nvls-1),0).  compute cnt=1.  loop i = 1 to nvls.  loop k = 1 to (nvls-1).  compute conmat1(i,k)=custcode(1,cnt).  compute cnt=cnt+1.  end loop.  end loop.  loop k=1 to n.  compute x(k,:)=conmat1((rsum(x(k,:))+1),:).  end loop.  end if.  end if.  compute xskip = 1.  compute dummat = make((nx+1),nx,0).  compute dummat((2:nrow(dummat)),:)=ident(nx).  do if ( mcw = 4).  compute dummat(1,:) = minus1.  end if.  do if ( mcw = 2).  loop i = 2 to nrow(dummat).  loop j = 1 to (i-1).  compute dummat(i,j) = 1.  end loop.  end loop.  end if.  do if ( mcw = 3).  compute dummat=conmat1.  end if.  do if ( mcw = 5 and criterr=0).  compute dummat=conmat1.  end if.  compute dummat={nnvls, dummat}.  compute x={dd(:,1),x}.  compute temp = x.  compute temp(GRADE(x(:,1)),:) = x.  compute x = temp.  release conmat1,temp,dd,xskip,xdes,dummy.  end if.  end if  .  compute wmodvals=nnvls.  compute nwpval=nrow(wmodvals).  do if (criterr=0).  compute minwwarn=0.  compute maxwwarn=0.  compute wnotev=0.  compute wtmp=x(:,2:ncol(x)).  compute wcatlab={'W1';'W2';'W3';'W4';'W5';'W6';'W7';'W8';'W9'}.  compute nwvls=nvls-1.  compute mcwok=1.  compute dummatw=dummat.  compute wprobval=dummatw(:,2:ncol(dummatw)).  do if (modcok=1).  compute wcontval=make(2,ncol(wprobval),-999).  compute temp=0.  loop i = 1 to 2.  loop j = 1 to nrow(dummatw).  do if (contvec(i,1)=dummatw(j,1)).  compute wcontval(i,:)=wprobval(j,:).  compute temp=temp+1.  end if.  end loop.  end loop.  do if (temp < 2).  compute notecode(notes,1) = 20.  compute notes = notes + 1.  compute modcok=0.  end if.  end if.  do if (wmodval(1,1) <> 999).  compute notecode(notes,1) = 9.  compute notes = notes + 1.  end if.  release tmp, dummat.  end if.  end if.  do if (nzs > 0 and mcz > 0).  compute tmp={rownum,ztmp(:,1)}.  .  compute dd= tmp.  compute temp = dd.  compute temp(GRADE(dd(:,2)),:) = dd.  compute dd = temp.  compute dummy = design(dd(:,2)).  compute nvls = ncol(dummy).  compute nnvls = csum(dummy).  compute mnvls = cmin(t(nnvls)).  compute conmat1=1.  do if (mnvls < 2).  compute errcode(errs,1) = 5.  compute errs = errs + 1.  compute criterr = 1.  end if.  do if (nvls > 9).  compute errcode(errs,1) = 4.  compute errs = errs+1.  compute criterr = 1.  end if.  do if (criterr = 0).  compute dumok = 1.  compute nnvls=make(nvls,1,0).  compute nnvls(1,1)=dd(1,2).  compute temp = 2.  loop i = 2 to n.  do if (dd(i,2) <> nnvls((temp-1),1)).  compute nnvls(temp,1)=dd(i,2).  compute temp = temp+1.  end if.  end loop.  do if ( mcz > 0).  compute x = dummy(:,2:ncol(dummy)).  compute nx = ncol(x).  compute minus1 = make(1,ncol(x),-1).  compute xdes=make((nx+1),3,0).  compute xdes(1,1)=dd(1,2).  compute xdes(1,2)=1.  compute temp = 2.  loop k = 2 to n.  do if (dd(k,2) <> dd((k-1),2)).  compute xdes(temp,2) = k.  compute xdes(temp,1) = dd(k,2).  compute xdes((temp-1),3) = k-1.  compute temp=temp+1.  end if.  end loop.  compute xdes((temp-1),3)=n.  compute xdes = {xdes, (xdes(:,3)-xdes(:,2)+1)}.  do if ( mcz = 4).  loop k = 1 to n.  do if (rsum(x(k,:)) = 0).  compute x(k,:) = minus1.  end if.  end loop.  end if.  do if ( mcz = 2 or mcz = 3 or mcz =5).  loop k = 1 to n.  do if (rsum(x(k,:)) > 0).  loop i = 1 to ncol(x).  do if (x(k,i) = 0).  compute x(k,i) = 1.  else.  break.  end if.  end loop.  end if.  end loop.  do if ( mcz = 3).  compute conmat1={-8,1,1,1,1,1,1,1,1; 0,-7,1,1,1,1,1,1,1; 0,0,-6,1,1,1,1,1,1; 0,0,0,-5,1,1,1,1,1; 0,0,0,0,-4,1,1,1,1; 0,0,0,0,0,-3,1,1,1; 0,0,0,0,0,0,-2,1,1; 0,0,0,0,0,0,0,-1,1}.  loop i = 1 to 8.  compute conmat1(i,:)=conmat1(i,:)/(10-i).  end loop.  compute conmat1=t(conmat1((10-nvls):8,(10-nvls):9)).  loop k=1 to n.  compute x(k,:)=conmat1((rsum(x(k,:))+1),:).  end loop.  end if.  end if.  do if ( mcz = 5).  compute custcode={ -999 }.  do if (ncol(custcode) <> (nvls*(nvls-1))).  compute errcode(errs,1) = (37+ 3 ).  compute errs = errs + 1.  compute criterr = 1.  end if.  do if (ncol(custcode) = (nvls*(nvls-1))).  compute conmat1=make(nvls,(nvls-1),0).  compute cnt=1.  loop i = 1 to nvls.  loop k = 1 to (nvls-1).  compute conmat1(i,k)=custcode(1,cnt).  compute cnt=cnt+1.  end loop.  end loop.  loop k=1 to n.  compute x(k,:)=conmat1((rsum(x(k,:))+1),:).  end loop.  end if.  end if.  compute xskip = 1.  compute dummat = make((nx+1),nx,0).  compute dummat((2:nrow(dummat)),:)=ident(nx).  do if ( mcz = 4).  compute dummat(1,:) = minus1.  end if.  do if ( mcz = 2).  loop i = 2 to nrow(dummat).  loop j = 1 to (i-1).  compute dummat(i,j) = 1.  end loop.  end loop.  end if.  do if ( mcz = 3).  compute dummat=conmat1.  end if.  do if ( mcz = 5 and criterr=0).  compute dummat=conmat1.  end if.  compute dummat={nnvls, dummat}.  compute x={dd(:,1),x}.  compute temp = x.  compute temp(GRADE(x(:,1)),:) = x.  compute x = temp.  release conmat1,temp,dd,xskip,xdes,dummy.  end if.  end if  .  compute zmodvals=nnvls.  compute nzpval=nrow(zmodvals).  do if (criterr=0).  compute minzwarn=0.  compute maxzwarn=0.  compute znotev=0.  compute ztmp=x(:,2:ncol(x)).  compute zcatlab={'Z1';'Z2';'Z3';'Z4';'Z5';'Z6';'Z7';'Z8';'Z9'}.  compute nzvls=nvls-1.  compute mczok=1.  compute dummatz=dummat.  compute zprobval=dummatz(:,2:ncol(dummatz)).  do if (modcok=1).  compute zcontval=make(2,ncol(zprobval),-999).  compute temp=0.  loop i = 1 to 2.  loop j = 1 to nrow(dummatz).  do if (contvec(i,2)=dummatz(j,1)).  compute zcontval(i,:)=zprobval(j,:).  compute temp=temp+1.  end if.  end loop.  end loop.  do if (temp < 2).  compute notecode(notes,1) = 20.  compute notes = notes + 1.  compute modcok=0.  end if.  end if.  do if (zmodval(1,1) <> 999).  compute notecode(notes,1) = 10.  compute notes = notes + 1.  end if.  release tmp, dummat.  end if.  end if.  do if (nxs > 0 and mcx > 0).  compute tmp={rownum,xtmp(:,1)}.  .  compute dd= tmp.  compute temp = dd.  compute temp(GRADE(dd(:,2)),:) = dd.  compute dd = temp.  compute dummy = design(dd(:,2)).  compute nvls = ncol(dummy).  compute nnvls = csum(dummy).  compute mnvls = cmin(t(nnvls)).  compute conmat1=1.  do if (mnvls < 2).  compute errcode(errs,1) = 5.  compute errs = errs + 1.  compute criterr = 1.  end if.  do if (nvls > 9).  compute errcode(errs,1) = 4.  compute errs = errs+1.  compute criterr = 1.  end if.  do if (criterr = 0).  compute dumok = 1.  compute nnvls=make(nvls,1,0).  compute nnvls(1,1)=dd(1,2).  compute temp = 2.  loop i = 2 to n.  do if (dd(i,2) <> nnvls((temp-1),1)).  compute nnvls(temp,1)=dd(i,2).  compute temp = temp+1.  end if.  end loop.  do if ( mcx > 0).  compute x = dummy(:,2:ncol(dummy)).  compute nx = ncol(x).  compute minus1 = make(1,ncol(x),-1).  compute xdes=make((nx+1),3,0).  compute xdes(1,1)=dd(1,2).  compute xdes(1,2)=1.  compute temp = 2.  loop k = 2 to n.  do if (dd(k,2) <> dd((k-1),2)).  compute xdes(temp,2) = k.  compute xdes(temp,1) = dd(k,2).  compute xdes((temp-1),3) = k-1.  compute temp=temp+1.  end if.  end loop.  compute xdes((temp-1),3)=n.  compute xdes = {xdes, (xdes(:,3)-xdes(:,2)+1)}.  do if ( mcx = 4).  loop k = 1 to n.  do if (rsum(x(k,:)) = 0).  compute x(k,:) = minus1.  end if.  end loop.  end if.  do if ( mcx = 2 or mcx = 3 or mcx =5).  loop k = 1 to n.  do if (rsum(x(k,:)) > 0).  loop i = 1 to ncol(x).  do if (x(k,i) = 0).  compute x(k,i) = 1.  else.  break.  end if.  end loop.  end if.  end loop.  do if ( mcx = 3).  compute conmat1={-8,1,1,1,1,1,1,1,1; 0,-7,1,1,1,1,1,1,1; 0,0,-6,1,1,1,1,1,1; 0,0,0,-5,1,1,1,1,1; 0,0,0,0,-4,1,1,1,1; 0,0,0,0,0,-3,1,1,1; 0,0,0,0,0,0,-2,1,1; 0,0,0,0,0,0,0,-1,1}.  loop i = 1 to 8.  compute conmat1(i,:)=conmat1(i,:)/(10-i).  end loop.  compute conmat1=t(conmat1((10-nvls):8,(10-nvls):9)).  loop k=1 to n.  compute x(k,:)=conmat1((rsum(x(k,:))+1),:).  end loop.  end if.  end if.  do if ( mcx = 5).  compute custcode={ -999 }.  do if (ncol(custcode) <> (nvls*(nvls-1))).  compute errcode(errs,1) = (37+ 1 ).  compute errs = errs + 1.  compute criterr = 1.  end if.  do if (ncol(custcode) = (nvls*(nvls-1))).  compute conmat1=make(nvls,(nvls-1),0).  compute cnt=1.  loop i = 1 to nvls.  loop k = 1 to (nvls-1).  compute conmat1(i,k)=custcode(1,cnt).  compute cnt=cnt+1.  end loop.  end loop.  loop k=1 to n.  compute x(k,:)=conmat1((rsum(x(k,:))+1),:).  end loop.  end if.  end if.  compute xskip = 1.  compute dummat = make((nx+1),nx,0).  compute dummat((2:nrow(dummat)),:)=ident(nx).  do if ( mcx = 4).  compute dummat(1,:) = minus1.  end if.  do if ( mcx = 2).  loop i = 2 to nrow(dummat).  loop j = 1 to (i-1).  compute dummat(i,j) = 1.  end loop.  end loop.  end if.  do if ( mcx = 3).  compute dummat=conmat1.  end if.  do if ( mcx = 5 and criterr=0).  compute dummat=conmat1.  end if.  compute dummat={nnvls, dummat}.  compute x={dd(:,1),x}.  compute temp = x.  compute temp(GRADE(x(:,1)),:) = x.  compute x = temp.  release conmat1,temp,dd,xskip,xdes,dummy.  end if.  end if  .  do if (criterr=0).  compute xtmp=x(:,2:ncol(x)).  compute xcatlab={'X1';'X2';'X3';'X4';'X5';'X6';'X7';'X8';'X9'}.  compute nxvls=nvls-1.  compute xdich=(nvls=2).  compute mcxok=1.  compute dummatx=dummat.  compute xmodvals=dummatx(:,1).  compute nxpval=nrow(xmodvals).  release tmp, dummat.  end if.  end if.  compute intlab=make(100,1,' ').  compute intlab( 1 ,1)= 'Int_1'.  compute intlab( 2 ,1)= 'Int_2'.  compute intlab( 3 ,1)= 'Int_3'.  compute intlab( 4 ,1)= 'Int_4'.  compute intlab( 5 ,1)= 'Int_5'.  compute intlab( 6 ,1)= 'Int_6'.  compute intlab( 7 ,1)= 'Int_7'.  compute intlab( 8 ,1)= 'Int_8'.  compute intlab( 9 ,1)= 'Int_9'.  compute intlab( 10 ,1)= 'Int_10'.  compute intlab( 11 ,1)= 'Int_11'.  compute intlab( 12 ,1)= 'Int_12'.  compute intlab( 13 ,1)= 'Int_13'.  compute intlab( 14 ,1)= 'Int_14'.  compute intlab( 15 ,1)= 'Int_15'.  compute intlab( 16 ,1)= 'Int_16'.  compute intlab( 17 ,1)= 'Int_17'.  compute intlab( 18 ,1)= 'Int_18'.  compute intlab( 19 ,1)= 'Int_19'.  compute intlab( 20 ,1)= 'Int_20'.  compute intlab( 21 ,1)= 'Int_21'.  compute intlab( 22 ,1)= 'Int_22'.  compute intlab( 23 ,1)= 'Int_23'.  compute intlab( 24 ,1)= 'Int_24'.  compute intlab( 25 ,1)= 'Int_25'.  compute intlab( 26 ,1)= 'Int_26'.  compute intlab( 27 ,1)= 'Int_27'.  compute intlab( 28 ,1)= 'Int_28'.  compute intlab( 29 ,1)= 'Int_29'.  compute intlab( 30 ,1)= 'Int_30'.  compute intlab( 31 ,1)= 'Int_31'.  compute intlab( 32 ,1)= 'Int_32'.  compute intlab( 33 ,1)= 'Int_33'.  compute intlab( 34 ,1)= 'Int_34'.  compute intlab( 35 ,1)= 'Int_35'.  compute intlab( 36 ,1)= 'Int_36'.  compute intlab( 37 ,1)= 'Int_37'.  compute intlab( 38 ,1)= 'Int_38'.  compute intlab( 39 ,1)= 'Int_39'.  compute intlab( 40 ,1)= 'Int_40'.  compute intlab( 41 ,1)= 'Int_41'.  compute intlab( 42 ,1)= 'Int_42'.  compute intlab( 43 ,1)= 'Int_43'.  compute intlab( 44 ,1)= 'Int_44'.  compute intlab( 45 ,1)= 'Int_45'.  compute intlab( 46 ,1)= 'Int_46'.  compute intlab( 47 ,1)= 'Int_47'.  compute intlab( 48 ,1)= 'Int_48'.  compute intlab( 49 ,1)= 'Int_49'.  compute intlab( 50 ,1)= 'Int_50'.  compute intlab( 51 ,1)= 'Int_51'.  compute intlab( 52 ,1)= 'Int_52'.  compute intlab( 53 ,1)= 'Int_53'.  compute intlab( 54 ,1)= 'Int_54'.  compute intlab( 55 ,1)= 'Int_55'.  compute intlab( 56 ,1)= 'Int_56'.  compute intlab( 57 ,1)= 'Int_57'.  compute intlab( 58 ,1)= 'Int_58'.  compute intlab( 59 ,1)= 'Int_59'.  compute intlab( 60 ,1)= 'Int_60'.  compute intlab( 61 ,1)= 'Int_61'.  compute intlab( 62 ,1)= 'Int_62'.  compute intlab( 63 ,1)= 'Int_63'.  compute intlab( 64 ,1)= 'Int_64'.  compute intlab( 65 ,1)= 'Int_65'.  compute intlab( 66 ,1)= 'Int_66'.  compute intlab( 67 ,1)= 'Int_67'.  compute intlab( 68 ,1)= 'Int_68'.  compute intlab( 69 ,1)= 'Int_69'.  compute intlab( 70 ,1)= 'Int_70'.  compute intlab( 71 ,1)= 'Int_71'.  compute intlab( 72 ,1)= 'Int_72'.  compute intlab( 73 ,1)= 'Int_73'.  compute intlab( 74 ,1)= 'Int_74'.  compute intlab( 75 ,1)= 'Int_75'.  compute intlab( 76 ,1)= 'Int_76'.  compute intlab( 77 ,1)= 'Int_77'.  compute intlab( 78 ,1)= 'Int_78'.  compute intlab( 79 ,1)= 'Int_79'.  compute intlab( 80 ,1)= 'Int_80'.  compute intlab( 81 ,1)= 'Int_81'.  compute intlab( 82 ,1)= 'Int_82'.  compute intlab( 83 ,1)= 'Int_83'.  compute intlab( 84 ,1)= 'Int_84'.  compute intlab( 85 ,1)= 'Int_85'.  compute intlab( 86 ,1)= 'Int_86'.  compute intlab( 87 ,1)= 'Int_87'.  compute intlab( 88 ,1)= 'Int_88'.  compute intlab( 89 ,1)= 'Int_89'.  compute intlab( 90 ,1)= 'Int_90'.  compute intlab( 91 ,1)= 'Int_91'.  compute intlab( 92 ,1)= 'Int_92'.  compute intlab( 93 ,1)= 'Int_93'.  compute intlab( 94 ,1)= 'Int_94'.  compute intlab( 95 ,1)= 'Int_95'.  compute intlab( 96 ,1)= 'Int_96'.  compute intlab( 97 ,1)= 'Int_97'.  compute intlab( 98 ,1)= 'Int_98'.  compute intlab( 99 ,1)= 'Int_99'.  compute intlab( 100 ,1)= 'Int_100'.  compute bcmat=make(needed,needed,0).  compute wcmat=make(needed,needed,0).  compute zcmat=make(needed,needed,0).  compute wzcmat=make(needed,needed,0).  compute wsum=0.  compute zsum=0.  compute wzsum=0.  end if.  do if (criterr = 0 and model <> 999).  compute modelmat= {1,0,0,0,0,0,0,1,0,0;2,0,0,0,0,0,0,1,1,0;3,0,0,0,0,0,0,1,1,1;4,0,0,0,0,0,0,0,0,0; 5,0,0,0,0,0,0,1,0,0;6,0,0,0,0,0,0,0,0,0;7,1,0,0,0,0,0,0,0,0;8,1,0,0,0,0,0,1,0,0; 9,1,1,0,0,0,0,0,0,0;10,1,1,0,0,0,0,1,1,0;11,1,1,1,0,0,0,0,0,0;12,1,1,1,0,0,0,1,1,1; 13,1,1,1,0,0,0,1,0,0;14,0,0,0,1,0,0,0,0,0;15,0,0,0,1,0,0,1,0,0;16,0,0,0,1,1,0,0,0,0; 17,0,0,0,1,1,0,1,1,0;18,0,0,0,1,1,1,0,0,0;19,0,0,0,1,1,1,1,1,1;20,0,0,0,1,1,1,1,0,0; 21,1,0,0,0,1,0,0,0,0;22,1,0,0,0,1,0,1,0,0;23,0,0,0,0,0,0,0,0,0;24,0,0,0,0,0,0,0,0,0; 25,0,0,0,0,0,0,0,0,0;26,0,0,0,0,0,0,0,0,0;27,0,0,0,0,0,0,0,0,0;28,1,0,0,0,1,0,0,1,0; 29,1,0,0,0,1,0,1,1,0;30,0,0,0,0,0,0,0,0,0;31,0,0,0,0,0,0,0,0,0;32,0,0,0,0,0,0,0,0,0; 33,0,0,0,0,0,0,0,0,0;34,0,0,0,0,0,0,0,0,0;35,0,0,0,0,0,0,0,0,0;36,0,0,0,0,0,0,0,0,0; 37,0,0,0,0,0,0,0,0,0;38,0,0,0,0,0,0,0,0,0;39,0,0,0,0,0,0,0,0,0;40,0,0,0,0,0,0,0,0,0; 41,0,0,0,0,0,0,0,0,0;42,0,0,0,0,0,0,0,0,0;43,0,0,0,0,0,0,0,0,0;44,0,0,0,0,0,0,0,0,0; 45,0,0,0,0,0,0,0,0,0;46,0,0,0,0,0,0,0,0,0;47,0,0,0,0,0,0,0,0,0;48,0,0,0,0,0,0,0  ,0,0; 49,0,0,0,0,0,0,0,0,0;50,0,0,0,0,0,0,0,0,0;51,0,0,0,0,0,0,0,0,0;52,0,0,0,0,0,0,0,0,0; 53,0,0,0,0,0,0,0,0,0;54,0,0,0,0,0,0,0,0,0;55,0,0,0,0,0,0,0,0,0;56,0,0,0,0,0,0,0,0,0; 57,0,0,0,0,0,0,0,0,0;58,1,0,0,1,0,0,0,0,0;59,1,0,0,1,0,0,1,0,0;60,1,1,0,1,0,0,0,0,0; 61,1,1,0,1,0,0,1,0,0;62,1,1,0,1,0,0,0,1,0;63,1,1,0,1,0,0,1,1,0;64,1,0,0,1,1,0,0,0,0; 65,1,0,0,1,1,0,1,0,0;66,1,0,0,1,1,0,0,1,0;67,1,0,0,1,1,0,1,1,0;68,1,1,1,1,0,0,0,0,0; 69,1,1,1,1,0,0,1,1,1;70,1,0,0,1,1,1,0,0,0;71,1,0,0,1,1,1,1,1,1;72,1,1,1,1,1,1,0,0,0; 73,1,1,1,1,1,1,1,1,1;74,0,0,0,1,0,0,0,0,0;75,1,1,0,1,1,0,0,0,0;76,1,1,0,1,1,0,1,1,0; 77,0,0,0,0,0,0,0,0,0;78,0,0,0,0,0,0,0,0,0;79,0,0,0,0,0,0,0,0,0;80,0,0,0,0,0,0,0,0,0; 81,0,0,0,0,0,0,0,0,0;82,0,0,0,0,0,0,0,0,0;83,1,0,0,0,0,0,0,0,0;84,1,0,0,0,0,0,0,0,0; 85,1,0,0,0,0,0,1,0,0;86,1,0,0,0,0,0,1,0,0;87,0,0,0,1,0,0,0,0,0;88,0,0,0,1,0,0,0,0,0; 89,0,0,0,1,0,0,1,0,0;90,0,0,0,1,0,0,1,0,0;91,0,0,0,0,0,0,0,0,0;92,1,0,0,1,0,0,1,0,0}.  compute tmp=modelmat(model,2:ncol(modelmat)).  do if (model < 4).  compute bcmat((nxs+1),1)=1.  end if.  do if ((model > 3) and (model <> 6)).  compute bcmat((nxs+1):(nxs+nms),1)=onem.  compute bcmat(nrow(bcmat),(nxs+1):(nxs+nms))=t(onem).  compute bcmat(nrow(bcmat),1)=1.  end if.  do if ((model = 6) or (model > 82 and model < 93)).  loop j = 2 to nrow(bcmat).  loop i = 1 to (j-1).  compute bcmat(j,i)=1.  end loop.  end loop.  end if.  do if (model = 80).  loop i = 1 to nms.  compute bcmat((nrow(bcmat)-1),i)=1.  end loop.  end if.  do if (model = 81).  loop j = 3 to nrow(bcmat).  compute bcmat(j,2)=1.  end loop.  end if.  do if (model = 82).  compute bcmat(3,2)=1.  compute bcmat(5,4)=1.  end if.  do if (tmp(1,1)=1).  compute wcmat((nxs+1):(nxs+nms),1)=onem.  compute wprod=1.  compute xprod=1.  do if (model = 83 or model = 86).  compute onemsx=onem.  loop i = 1 to (nms-1).  compute onemsx(i+1,1)=0.  end loop.  compute wcmat((nxs+1):(nxs+nms),1)=onemsx.  end if.  end if.  do if (tmp(1,4)=1).  compute wcmat(nrow(wcmat),(nxs+1):(nxs+nms))=t(onem).  compute wprod=1.  do if (model = 87 or model = 90).  compute onemsx=onem.  loop i = 1 to (nms-1).  compute onemsx(i,1)=0.  end loop.  compute wcmat(nrow(wcmat),(nxs+1):(nxs+nms))=t(onemsx).  end if.  end if.  do if (tmp(1,7)=1).  compute wcmat(nrow(wcmat),1)=1.  compute wprod=1.  compute xprod=1.  end if.  do if (tmp(1,2)=1).  compute zcmat((nxs+1):(nxs+nms),1)=onem.  compute zprod=1.  compute xprod=1.  end if.  do if (tmp(1,5)=1).  compute zcmat(nrow(zcmat),(nxs+1):(nxs+nms))=t(onem).  compute zprod=1.  end if.  do if (tmp(1,8)=1).  compute zcmat(nrow(zcmat),1)=1.  compute zprod=1.  compute xprod=1.  end if.  do if (tmp(1,3)=1).  compute wzcmat((nxs+1):(nxs+nms),1)=onem.  compute xprod=1.  compute wprod=1.  compute zprod=1.  end if.  do if (tmp(1,6)=1).  compute wzcmat(nrow(wzcmat),(nxs+1):(nxs+nms))=t(onem).  compute zprod=1.  compute wprod=1.  end if.  do if (tmp(1,9)=1).  compute wzcmat(nrow(wzcmat),1)=1.  compute xprod=1.  compute wprod=1.  compute zprod=1.  end if.  do if (model = 91 or model = 92).  loop j = 1 to (nms-1).  loop i = 1 to j.  compute wcmat((nxs+1+j),(nxs+i))=1.  end loop.  end loop.  end if.  do if (nms < 0).  loop i = 1 to nms.  compute tmp=csum(wcmat(:,(1+i)))+csum(zcmat(:,(1+i)))+csum(wzcmat(:,(1+i))).  compute mprod(1,i)=(tmp>0).  end loop.  end if.  end if.  do if (ncs > 0).  compute ccmat=make((nms+nys),ncs,1).  compute ccmatoff=ccmat.  do if (covmy=1).  compute ccmat(nrow(ccmat),:)=make(1,ncs,0).  end if.  do if (covmy=2).  compute ccmat(1:nms,:)=make(nms,ncs,0).  end if.  do if (cmatrix(1,1) <> -999).  do if (ncol(cmatrix) <> ((nms+nys)*ncs)).  compute errcode(errs,1)=29.  compute errs=errs+1.  compute criterr=1.  end if.  do if (criterr = 0).  compute tmp=1.  loop i = 1 to (nms+nys).  loop j = 1 to ncs.  compute ccmat(i,j)=1-(cmatrix(1,tmp) = 0).  compute tmp=tmp+1.  end loop.  end loop.  do if (rsum((csum(ccmat)=0)) <> 0).  compute errcode(errs,1)=30.  compute errs=errs+1.  compute criterr=1.  end if.  end if.  do if (covmy <> 0).  compute notecode(notes,1)=1.  compute notes=notes+1.  end if.  end if.  end if.  do if (criterr=0).  compute needed=needed*(needed-1)/2.  compute nopath=0.  do if (bmatrix(1,1) <> -999).  compute tmp=1.  do if ((ncol(bmatrix) <> needed) or (csum(rsum(bmatrix))=0)).  compute errcode(errs,1)=16.  compute errs=errs+1.  compute criterr=1.  else.  loop i = 2 to nrow(bcmat).  loop j = 1 to (i-1).  compute bcmat(i,j)=1-(bmatrix(1,tmp) = 0).  compute tmp=tmp+1.  end loop.  end loop.  end if.  do if ((csum(bcmat(:,1))=0) and criterr=0).  compute errcode(errs,1)=22.  compute errs=errs+1.  compute criterr=1.  end if.  do if ((rsum(bcmat(nrow(bcmat),:))=0) and criterr=0).  compute errcode(errs,1)=23.  compute errs=errs+1.  compute criterr=1.  end if.  compute dm=0.  do if (nms > 0).  loop i = 1 to nms.  do if (((rsum(bcmat((nxs+i),:)) = 0) or (csum(bcmat(:,(nxs+i))) = 0)) and (dm=0) and (criterr=0)).  compute errcode(errs,1)=26.  compute errs=errs+1.  compute criterr=1.  compute dm=1.  end if.  end loop.  end if.  release dm.  end if.  end if.  do if (criterr=0).  do if (wmatrix(1,1) <> -999).  compute tmp=1.  do if (ncol(wmatrix) <> needed).  compute errcode(errs,1)=17.  compute errs=errs+1.  compute criterr=1.  else.  compute modelvar(1,1)='CUSTOM'.  loop i = 2 to nrow(wcmat).  loop j = 1 to (i-1).  compute wcmat(i,j)=1-(wmatrix(1,tmp) = 0).  do if ((wcmat(i,j)=1) and (bcmat(i,j)=0) and (nopath=0)).  compute errcode(errs,1)=20.  compute errs=errs+1.  compute criterr=1.  compute nopath=1.  end if.  compute tmp=tmp+1.  end loop.  end loop.  end if.  end if.  do if (zmatrix(1,1) <> -999).  compute tmp=1.  do if (ncol(zmatrix) <> needed).  compute errcode(errs,1)=18.  compute errs=errs+1.  compute criterr=1.  else.  compute modelvar(1,1)='CUSTOM'.  do if (csum(rsum(wcmat))=0 and model=999).  compute errcode(errs,1)=21.  compute errs=errs+1.  compute criterr=1.  end if.  loop i = 2 to nrow(zcmat).  loop j = 1 to (i-1).  compute zcmat(i,j)=1-(zmatrix(1,tmp) = 0).  do if ((zcmat(i,j)=1) and (bcmat(i,j)=0) and (nopath=0)).  compute errcode(errs,1)=20.  compute errs=errs+1.  compute criterr=1.  compute nopath=1.  end if.  compute tmp=tmp+1.  end loop.  end loop.  end if.  end if.  compute tmp=1.  do if (wzmatrix(1,1) <> -999).  do if (ncol(wzmatrix) <> needed).  compute errcode(errs,1)=19.  compute errs=errs+1.  compute criterr=1.  end if.  compute modelvar(1,1)='CUSTOM'.  end if.  do if (criterr=0).  loop i = 2 to nrow(wzcmat).  loop j = 1 to (i-1).  do if (wzmatrix(1,1) <> -999).  compute wzcmat(i,j)=1-(wzmatrix(1,tmp) = 0).  end if.  do if (wzcmat(i,j)=1).  compute wcmat(i,j)=1.  compute zcmat(i,j)=1.  end if.  do if ((wzcmat(i,j)=1) and (bcmat(i,j)=0) and (nopath=0)).  compute errcode(errs,1)=20.  compute errs=errs+1.  compute criterr=1.  compute nopath=1.  end if.  compute tmp=tmp+1.  end loop.  end loop.  end if.  end if.  do if (criterr=0).  compute xprod=csum(wcmat(:,1))+csum(zcmat(:,1))+csum(wzcmat(:,1)).  compute xprod=(xprod > 0).  compute wsum=csum(rsum(wcmat)).  compute wprod=(wsum > 0).  do if (nms > 0).  loop i = 1 to nms.  compute tmp=csum(wcmat(:,(1+i)))+csum(zcmat(:,(1+i)))+csum(wzcmat(:,(1+i))).  compute mprod(1,i)=(tmp>0).  end loop.  end if.  do if ((wsum > 0) and (w = 'xxxxx')).  compute errcode(errs,1)=11.  compute errs=errs+1.  compute criterr=1.  end if.  do if ((wsum = 0) and (w <> 'xxxxx')).  compute errcode(errs,1)=10.  compute errs=errs+1.  compute criterr=1.  end if.  compute zsum=csum(rsum(zcmat)).  compute zprod=(zsum > 0).  do if ((zsum > 0) and (z = 'xxxxx')).  compute errcode(errs,1)=13.  compute errs=errs+1.  compute criterr=1.  end if.  do if ((zsum = 0) and (z <> 'xxxxx')).  compute errcode(errs,1)=12.  compute errs=errs+1.  compute criterr=1.  end if.  do if ((zsum > 0) and (wsum = 0)).  compute errcode(errs,1)=35.  compute errs=errs+1.  compute criterr=1.  end if.  end if.  do if (criterr=0 and nms > 1).  compute serchk=bcmat(2:(nrow(bcmat)-1),2:ncol(bcmat)).  do if (csum(rsum(serchk))) > 0.  compute serial=1.  do if (nms > 6).  compute errcode(errs,1)=36.  compute errs=errs+1.  compute criterr=1.  end if.  end if.  end if.  do if (center > 0 and criterr=0).  compute centvar={' '}.  do if (criterr=0).  do if ((center = 1) or (center = 2 and wdich = 0)).  do if (wprod=1 and mcwok=0 and nwpval > 0).  loop i = 1 to nws.  compute wtmp(:,i)=wtmp(:,i)-(csum(wtmp(:,i))/n).  compute centvar={centvar,wnames(1,i)}.  end loop.  .  compute desctmp=make((8-(4* wmodcust )),ncol( wtmp ),-999).  loop jd=1 to ncol( wtmp ).  compute descdat= wtmp (:,jd).  compute desctmp(1,jd) = csum(descdat)/nrow(descdat).  compute desctmp(2,jd) = (nrow(descdat)*sscp(descdat))-(t(csum(descdat))*(csum(descdat))).  compute desctmp(2,jd) = sqrt(desctmp(2,jd)/(nrow(descdat)*(nrow(descdat)-1))).  compute desctmp(3,jd)=cmin(descdat).  compute desctmp(4,jd)=cmax(descdat).  do if ( wmodcust =0).  compute minwarn=0.  compute maxwarn=0.  do if ((desctmp(3,jd)=desctmp(4,jd)) and novar=0).  compute errcode(errs,1)=15.  compute errs=errs+1.  compute criterr=1.  compute novar=1.  end if.  compute tmp=((descdat(:,1)=desctmp(3,jd))+(descdat(:,1)=desctmp(4,jd))).  compute desctmp(8,jd)=(csum(tmp)=nrow(tmp)).  compute tmp = descdat.  compute tmp(GRADE(descdat),:) = descdat.  compute descdat = tmp.  release tmp.  compute decval={.16;.5;.84}.  loop kd=1 to 3.  compute low=trunc(decval(kd,1)*(nrow(descdat)+1)).  compute lowdec=decval(kd,1)*(nrow(descdat)+1)-low.  compute value=descdat(low,1)+(descdat((low+1),1)-descdat(low,1))*lowdec.  compute desctmp((4+kd),jd)=value.  end loop.  compute mnotev=1.  compute modvals=desctmp(5:7,:).  do if (quantile <> 1).  compute desctmp(5,jd)=desctmp(1,jd)-desctmp(2,jd).  compute desctmp(6,jd)=desctmp(1,jd).  compute desctmp(7,jd)=desctmp(1,jd)+desctmp(2,jd).  compute modvals=desctmp(5:7,:).  compute mnotev=2.  do if (modvals(1,1) < desctmp(3,1)).  compute modvals(1,1)=desctmp(3,1).  compute minwarn=1.  end if.  do if (modvals(3,1) > desctmp(4,1)).  compute modvals(3,1)=desctmp(4,1).  compute maxwarn=1.  end if.  end if.  do if (desctmp(8,1)=1).  compute modvals={desctmp(3,1);desctmp(4,1)}.  compute mnotev=0.  compute minwarn=0.  compute maxwarn=0.  end if.  end if.  end loop  .  compute wmin=desctmp(3,1).  compute wmax=desctmp(4,1).  do if (wmodcust=0).  compute wmodvals=modvals.  compute wprobval=wmodvals.  end if.  end if.  end if.  do if ((center = 1) or (center = 2 and zdich = 0)).  do if (zprod=1 and mczok=0 and nzpval > 0).  loop i = 1 to nzs.  compute ztmp(:,i)=ztmp(:,i)-(csum(ztmp(:,i))/n).  compute centvar={centvar,znames(1,i)}.  end loop.  .  compute desctmp=make((8-(4* zmodcust )),ncol( ztmp ),-999).  loop jd=1 to ncol( ztmp ).  compute descdat= ztmp (:,jd).  compute desctmp(1,jd) = csum(descdat)/nrow(descdat).  compute desctmp(2,jd) = (nrow(descdat)*sscp(descdat))-(t(csum(descdat))*(csum(descdat))).  compute desctmp(2,jd) = sqrt(desctmp(2,jd)/(nrow(descdat)*(nrow(descdat)-1))).  compute desctmp(3,jd)=cmin(descdat).  compute desctmp(4,jd)=cmax(descdat).  do if ( zmodcust =0).  compute minwarn=0.  compute maxwarn=0.  do if ((desctmp(3,jd)=desctmp(4,jd)) and novar=0).  compute errcode(errs,1)=15.  compute errs=errs+1.  compute criterr=1.  compute novar=1.  end if.  compute tmp=((descdat(:,1)=desctmp(3,jd))+(descdat(:,1)=desctmp(4,jd))).  compute desctmp(8,jd)=(csum(tmp)=nrow(tmp)).  compute tmp = descdat.  compute tmp(GRADE(descdat),:) = descdat.  compute descdat = tmp.  release tmp.  compute decval={.16;.5;.84}.  loop kd=1 to 3.  compute low=trunc(decval(kd,1)*(nrow(descdat)+1)).  compute lowdec=decval(kd,1)*(nrow(descdat)+1)-low.  compute value=descdat(low,1)+(descdat((low+1),1)-descdat(low,1))*lowdec.  compute desctmp((4+kd),jd)=value.  end loop.  compute mnotev=1.  compute modvals=desctmp(5:7,:).  do if (quantile <> 1).  compute desctmp(5,jd)=desctmp(1,jd)-desctmp(2,jd).  compute desctmp(6,jd)=desctmp(1,jd).  compute desctmp(7,jd)=desctmp(1,jd)+desctmp(2,jd).  compute modvals=desctmp(5:7,:).  compute mnotev=2.  do if (modvals(1,1) < desctmp(3,1)).  compute modvals(1,1)=desctmp(3,1).  compute minwarn=1.  end if.  do if (modvals(3,1) > desctmp(4,1)).  compute modvals(3,1)=desctmp(4,1).  compute maxwarn=1.  end if.  end if.  do if (desctmp(8,1)=1).  compute modvals={desctmp(3,1);desctmp(4,1)}.  compute mnotev=0.  compute minwarn=0.  compute maxwarn=0.  end if.  end if.  end loop  .  compute zmin=desctmp(3,1).  compute zmax=desctmp(4,1).  do if (zmodcust=0).  compute zmodvals=modvals.  compute zprobval=zmodvals.  end if.  end if.  end if.  do if ((center = 1) or (center = 2 and xdich = 0)).  do if (xprod=1 and mcxok=0).  loop i = 1 to nxs.  compute xtmp(:,i)=xtmp(:,i)-(csum(xtmp(:,i))/n).  compute centvar={centvar,xnames(1,i)}.  end loop.  .  compute desctmp=make((8-(4* 0 )),ncol( xtmp ),-999).  loop jd=1 to ncol( xtmp ).  compute descdat= xtmp (:,jd).  compute desctmp(1,jd) = csum(descdat)/nrow(descdat).  compute desctmp(2,jd) = (nrow(descdat)*sscp(descdat))-(t(csum(descdat))*(csum(descdat))).  compute desctmp(2,jd) = sqrt(desctmp(2,jd)/(nrow(descdat)*(nrow(descdat)-1))).  compute desctmp(3,jd)=cmin(descdat).  compute desctmp(4,jd)=cmax(descdat).  do if ( 0 =0).  compute minwarn=0.  compute maxwarn=0.  do if ((desctmp(3,jd)=desctmp(4,jd)) and novar=0).  compute errcode(errs,1)=15.  compute errs=errs+1.  compute criterr=1.  compute novar=1.  end if.  compute tmp=((descdat(:,1)=desctmp(3,jd))+(descdat(:,1)=desctmp(4,jd))).  compute desctmp(8,jd)=(csum(tmp)=nrow(tmp)).  compute tmp = descdat.  compute tmp(GRADE(descdat),:) = descdat.  compute descdat = tmp.  release tmp.  compute decval={.16;.5;.84}.  loop kd=1 to 3.  compute low=trunc(decval(kd,1)*(nrow(descdat)+1)).  compute lowdec=decval(kd,1)*(nrow(descdat)+1)-low.  compute value=descdat(low,1)+(descdat((low+1),1)-descdat(low,1))*lowdec.  compute desctmp((4+kd),jd)=value.  end loop.  compute mnotev=1.  compute modvals=desctmp(5:7,:).  do if (quantile <> 1).  compute desctmp(5,jd)=desctmp(1,jd)-desctmp(2,jd).  compute desctmp(6,jd)=desctmp(1,jd).  compute desctmp(7,jd)=desctmp(1,jd)+desctmp(2,jd).  compute modvals=desctmp(5:7,:).  compute mnotev=2.  do if (modvals(1,1) < desctmp(3,1)).  compute modvals(1,1)=desctmp(3,1).  compute minwarn=1.  end if.  do if (modvals(3,1) > desctmp(4,1)).  compute modvals(3,1)=desctmp(4,1).  compute maxwarn=1.  end if.  end if.  do if (desctmp(8,1)=1).  compute modvals={desctmp(3,1);desctmp(4,1)}.  compute mnotev=0.  compute minwarn=0.  compute maxwarn=0.  end if.  end if.  end loop  .  compute xmodvals=modvals.  compute xprobval=xmodvals.  end if.  end if.  do if (nms > 0).  loop i = 1 to nms.  do if (mprod(1,i)=1).  compute mtmp(:,i)=mtmp(:,i)-(csum(mtmp(:,i))/n).  compute centvar={centvar,mnames(1,i)}.  end if.  end loop.  .  compute desctmp=make((8-(4* 0 )),ncol( mtmp ),-999).  loop jd=1 to ncol( mtmp ).  compute descdat= mtmp (:,jd).  compute desctmp(1,jd) = csum(descdat)/nrow(descdat).  compute desctmp(2,jd) = (nrow(descdat)*sscp(descdat))-(t(csum(descdat))*(csum(descdat))).  compute desctmp(2,jd) = sqrt(desctmp(2,jd)/(nrow(descdat)*(nrow(descdat)-1))).  compute desctmp(3,jd)=cmin(descdat).  compute desctmp(4,jd)=cmax(descdat).  do if ( 0 =0).  compute minwarn=0.  compute maxwarn=0.  do if ((desctmp(3,jd)=desctmp(4,jd)) and novar=0).  compute errcode(errs,1)=15.  compute errs=errs+1.  compute criterr=1.  compute novar=1.  end if.  compute tmp=((descdat(:,1)=desctmp(3,jd))+(descdat(:,1)=desctmp(4,jd))).  compute desctmp(8,jd)=(csum(tmp)=nrow(tmp)).  compute tmp = descdat.  compute tmp(GRADE(descdat),:) = descdat.  compute descdat = tmp.  release tmp.  compute decval={.16;.5;.84}.  loop kd=1 to 3.  compute low=trunc(decval(kd,1)*(nrow(descdat)+1)).  compute lowdec=decval(kd,1)*(nrow(descdat)+1)-low.  compute value=descdat(low,1)+(descdat((low+1),1)-descdat(low,1))*lowdec.  compute desctmp((4+kd),jd)=value.  end loop.  compute mnotev=1.  compute modvals=desctmp(5:7,:).  do if (quantile <> 1).  compute desctmp(5,jd)=desctmp(1,jd)-desctmp(2,jd).  compute desctmp(6,jd)=desctmp(1,jd).  compute desctmp(7,jd)=desctmp(1,jd)+desctmp(2,jd).  compute modvals=desctmp(5:7,:).  compute mnotev=2.  do if (modvals(1,1) < desctmp(3,1)).  compute modvals(1,1)=desctmp(3,1).  compute minwarn=1.  end if.  do if (modvals(3,1) > desctmp(4,1)).  compute modvals(3,1)=desctmp(4,1).  compute maxwarn=1.  end if.  end if.  do if (desctmp(8,1)=1).  compute modvals={desctmp(3,1);desctmp(4,1)}.  compute mnotev=0.  compute minwarn=0.  compute maxwarn=0.  end if.  end if.  end loop  .  compute mmodvals=modvals.  compute mprobval=mmodvals.  end if.  end if.  do if (ncol(centvar) > 1).  compute notecode(notes,1)=3.  compute notes=notes+1.  end if.  end if.  do if (criterr=0).  compute wsum=rsum(csum(wcmat)).  compute zsum=rsum(csum(zcmat)).  compute wzsum=rsum(csum(wzcmat)).  compute nump=make(1,(nys+nms),-999).  compue numint=make(1,(nys+nms),0).  compute datcount=1.  compute xtmpuse=0.  compute wtmpuse=0.  compute ztmpuse=0.  compute xwtmpus=0.  compute xztmpus=0.  compute wztmpus=0.  compute xwztmpu=0.  compute xtmploc=-999.  compute wtmploc=-999.  compute xwtmplo=-999.  compute ztmploc=-999.  compute xztmplo=-999.  compute wztmplo=-999.  compute xwztmplo=-999.  compute vlabs={' '}.  do if (ncs > 0).  compute ctmpuse=make(1,ncs,0).  end if.  do if (nms > 0).  compute mtmpuse=make(1,nms,0).  compute mwtmpus=make(1,nms,0).  compute mztmpus=make(1,nms,0).  compute mwztmpu=make(1,nms,0).  compute mtmploc=make(1,nms,0).  compute mwtmplo=make(nwvls,nms,-999).  compute mztmplo=make(nzvls,nms,-999).  compute mwztmplo=make((nwvls*nzvls),nms,-999).  end if.  do if (ncs > 0).  compute ctmploc=make(1,ncs,0).  end if.  compute fulldat=make(n,1,1).  compute datindx=make(1000,(nms+nys),-999).  compute wherew=make(2,(nms+nys),-999).  compute wherex=make(2,(nms+nys),-999).  compute wherez=make(2,(nms+nys),-999).  compute wherexw=make(2,(nms+nys),-999).  compute wherexz=make(2,(nms+nys),-999).  compute wherewz=make(2,(nms+nys),-999).  compute wherexwz=make(2,(nms+nys),-999).  do if (nms > 0).  compute wherem=make(nms,(nms+nys),-999).  compute wheremw = make(nms*2,(nms+nys),-999).  compute wheremz = make(nms*2,(nms+nys),-999).  compute wheremwz = make(nms*2,(nms+nys),-999).  end if.  compute wzhigh=make(1000,(((nms+1)*(nms+2))/2),0).  compute whigh=make(1000,(((nms+1)*(nms+2))/2),0).  compute zhigh=make(1000,(((nms+1)*(nms+2))/2),0).  compute fochigh=make(1000,(((nms+1)*(nms+2))/2),0).  compute xcoefloc={1;2;3;4;5;6;7;8;9}.  compute intkey = {' ', ' ', ' ', ' ', ' ', ' ', ' '}.  compute wzhighct=0.  compute whighct=0.  compute zhighct=0.  compute foccnt=0.  loop i = 2 to nrow(bcmat).  compute wdid=0.  compute zdid=0.  compute wzdid=0.  compute cntmp=1.  compute start=1.  do if (i < nrow(bcmat)).  compute outv=mtmp(:,(i-1)).  compute modlabel={mnames(1,(i-1));'constant'}.  end if.  do if (i = nrow(bcmat)).  compute outv=ytmp.  compute modlabel={ynames;'constant'}.  end if.  loop j = 1 to (i-1).  compute foccnt=foccnt+1.  do if (j = 1 and bcmat(i,j)=1).  compute outv={outv,xtmp}.  compute modlabel={modlabel;xcatlab(1:nxvls,1)}.  do if (xtmpuse=0).  compute fulldat={fulldat,xtmp}.  compute xtmpuse=1.  loop k4=datcount to (datcount+(nxvls-1)).  compute xtmploc={xtmploc;k4}.  end loop.  compute xtmploc=xtmploc(2:nrow(xtmploc),1).  compute datcount=datcount+nxvls.  end if.  compute datindx(start:(start+nrow(xtmploc)-1),(i-1))=xtmploc.  compute wherex(1,(i-1))=start+1.  compute wherex(2,(i-1))=start+nrow(xtmploc)-1+1.  do if (model = 74).  end if.  compute onebl=make(nrow(xtmploc),1,1).  compute fochigh((start+1):(start+nrow(xtmploc)),foccnt)=onebl.  compute start=start+nrow(xtmploc).  end if.  do if (j > 1 and bcmat(i,j)=1).  compute outv={outv,mtmp(:,(j-1))}.  compute modlabel={modlabel;mnames(1,(j-1))}.  do if (mtmpuse(1,(j-1))=0).  compute fulldat={fulldat,mtmp(:,(j-1))}.  compute mtmpuse(1,(j-1))=1.  compute mtmploc(1,(j-1))=datcount.  compute datcount=datcount+1.  end if.  compute datindx(start:(start+nrow(mtmploc)-1),(i-1))=mtmploc(1,(j-1)).  compute wherem((j-1),(i-1))=start+1.  compute onebl=make(nrow(mtmploc(1,j-1)),1,1).  compute ttt=nrow(mtmploc(1,(j-1)))+start-1.  compute fochigh((start+1):(start+nrow(mtmploc(1,(j-1)))),foccnt)=onebl.  compute start=start+nrow(mtmploc(1,(j-1))).  end if.  end loop.  do if (wsum > 0).  loop j = 1 to (i-1).  compute whighct=whighct+1.  do if (j = 1 and wcmat(i,j)=1).  do if (wdid=0).  compute outv={outv,wtmp}.  do if (ncs > 0 and wiscov > 0).  compute ccmatoff((i-1),wiscov)=0.  end if.  compute modlabel={modlabel;wcatlab(1:nwvls,1)}.  compute wdid=1.  do if (wtmpuse=0).  compute fulldat={fulldat,wtmp}.  do if (ncs > 0 and wiscov > 0).  compute ccmatoff((i-1),wiscov)=0.  end if.  compute wtmpuse=1.  loop k4=datcount to (datcount+(nwvls-1)).  compute wtmploc={wtmploc;k4}.  end loop.  compute wtmploc=wtmploc(2:nrow(wtmploc),1).  compute datcount=datcount+nwvls.  end if.  end if.  compute datindx(start:(start+nrow(wtmploc)-1),(i-1))=wtmploc.  compute wherew(1,(i-1))=start+1.  compute wherew(2,(i-1))=start+nrow(wtmploc)-1+1.  compute start=start+nrow(wtmploc).  loop k1=1 to nxvls.  loop k2 = 1 to nwvls.  compute outv={outv,(xtmp(:,k1)&*wtmp(:,k2))}.  do if (ncs > 0 and wiscov > 0).  compute ccmatoff((i-1),wiscov)=0.  end if.  compute modlabel={modlabel;intlab(cntmp,1)}.  compute intkey={intkey;intlab(cntmp,1),':',xcatlab(k1,1),'x',wcatlab(k2,1),' ',' '}.  compute cntmp=cntmp+1.  end loop.  end loop.  do if (xwtmpus=0).  compute fulldat={fulldat,outv(:,(ncol(outv)-(nxvls*nwvls)+1):ncol(outv))}.  compute xwtmpus=1.  do if (ncs > 0 and wiscov > 0).  compute ccmatoff((i-1),wiscov)=0.  end if.  loop k4=datcount to (datcount+((nwvls*nxvls)-1)).  compute xwtmplo={xwtmplo;k4}.  end loop.  compute xwtmplo=xwtmplo(2:nrow(xwtmplo),1).  compute datcount=datcount+(nxvls*nwvls).  end if.  compute datindx(start:(start+nrow(xwtmplo)-1),(i-1))=xwtmplo.  compute wherexw(1,(i-1))=start+1.  compute wherexw(2,(i-1))=start+nrow(xwtmplo)-1+1.  compute onebl=make(nrow(xwtmplo),1,1).  compute whigh((start+1):(start+nrow(xwtmplo)),whighct)=onebl.  compute start=start+nrow(xwtmplo).  end if.  do if (j > 1 and wcmat(i,j)=1).  do if (wdid=0 and model <> 74).  compute outv={outv,wtmp}.  do if (ncs > 0 and wiscov > 0).  compute ccmatoff((i-1),wiscov)=0.  end if.  compute modlabel={modlabel;wcatlab(1:nwvls,1)}.  compute wdid=1.  do if (wtmpuse=0).  compute fulldat={fulldat,wtmp}.  do if (ncs > 0 and wiscov > 0).  compute ccmatoff((i-1),wiscov)=0.  end if.  compute wtmpuse=1.  loop k4=datcount to (datcount+(nwvls-1)).  compute wtmploc={wtmploc;k4}.  end loop.  compute wtmploc=wtmploc(2:nrow(wtmploc),1).  compute datcount=datcount+nwvls.  end if.  compute datindx(start:(start+nrow(wtmploc)-1),(i-1))=wtmploc.  compute wherew(1,(i-1))=start+1.  compute wherew(2,(i-1))=start+nrow(wtmploc)-1+1.  compute start=start+nrow(wtmploc).  end if.  loop k2 = 1 to nwvls.  compute outv={outv,(mtmp(:,(j-1))&*wtmp(:,k2))}.  do if (ncs > 0 and wiscov > 0).  compute ccmatoff((i-1),wiscov)=0.  end if.  compute modlabel={modlabel;intlab(cntmp,1)}.  compute intkey={intkey;intlab(cntmp,1),':', mnames(1,(j-1)),'x',wcatlab(k2,1),' ',' '}.  compute cntmp=cntmp+1.  end loop.  do if (mwtmpus(1,(j-1))=0).  compute fulldat={fulldat,outv(:,(ncol(outv)-nwvls+1):ncol(outv))}.  do if (ncs > 0 and wiscov > 0).  compute ccmatoff((i-1),wiscov)=0.  end if.  compute mwtmpus(1,(j-1))=1.  compute mw22=-999.  loop k4=datcount to (datcount+(nwvls-1)).  compute mw22={mw22;k4}.  end loop.  compute mwtmplo(:,(j-1))=mw22(2:nrow(mw22),1).  compute datcount=datcount+nwvls.  end if.  compute datindx(start:(start+nrow(mwtmplo)-1),(i-1))=mwtmplo(:,(j-1)).  compute wheremw(((2*j)-3),(i-1))=start+1.  compute wheremw(((2*j)-2),(i-1))=start+nrow(mwtmplo)-1+1.  compute onebl=make(nrow(mwtmplo),1,1).  compute whigh((start+1):(start+nrow(mwtmplo)),whighct)=onebl.  compute start=start+nrow(mwtmplo).  end if.  end loop.  end if.  do if (zsum > 0).  loop j = 1 to (i-1).  compute zhighct=zhighct+1.  do if (j = 1 and zcmat(i,j)=1).  do if (zdid=0).  compute outv={outv,ztmp}.  do if (ncs > 0 and ziscov > 0).  compute ccmatoff((i-1),ziscov)=0.  end if.  compute modlabel={modlabel;zcatlab(1:nzvls,1)}.  compute zdid=1.  do if (ztmpuse=0).  compute fulldat={fulldat,ztmp}.  do if (ncs > 0 and ziscov > 0).  compute ccmatoff((i-1),ziscov)=0.  end if.  compute ztmpuse=1.  loop k4=datcount to (datcount+(nzvls-1)).  compute ztmploc={ztmploc;k4}.  end loop.  compute ztmploc=ztmploc(2:nrow(ztmploc),1).  compute datcount=datcount+nzvls.  end if.  end if.  compute datindx(start:(start+nrow(ztmploc)-1),(i-1))=ztmploc.  compute wherez(1,(i-1))=start+1.  compute wherez(2,(i-1))=start+nrow(ztmploc)-1+1.  compute start=start+nrow(ztmploc).  loop k1=1 to nxvls.  loop k2 = 1 to nzvls.  compute outv={outv,(xtmp(:,k1)&*ztmp(:,k2))}.  do if (ncs > 0 and ziscov > 0).  compute ccmatoff((i-1),ziscov)=0.  end if.  compute modlabel={modlabel;intlab(cntmp,1)}.  compute intkey={intkey;intlab(cntmp,1),':',xcatlab(k1,1),'x',zcatlab(k2,1),' ',' '}.  compute cntmp=cntmp+1.  end loop.  end loop.  do if (xztmpus=0).  compute fulldat={fulldat,outv(:,(ncol(outv)-(nxvls*nzvls)+1):ncol(outv))}.  do if (ncs > 0 and ziscov > 0).  compute ccmatoff((i-1),ziscov)=0.  end if.  compute xztmpus=1.  loop k4=datcount to (datcount+((nzvls*nxvls)-1)).  compute xztmplo={xztmplo;k4}.  end loop.  compute xztmplo=xztmplo(2:nrow(xztmplo),1).  compute datcount=datcount+(nxvls*nzvls).  end if.  compute datindx(start:(start+nrow(xztmplo)-1),(i-1))=xztmplo.  compute wherexz(1,(i-1))=start+1.  compute wherexz(2,(i-1))=start+nrow(xztmplo)-1+1.  compute onebl=make(nrow(xztmplo),1,1).  compute zhigh((start+1):(start+nrow(xztmplo)),zhighct)=onebl.  compute start=start+nrow(xztmplo).  end if.  do if (j > 1 and zcmat(i,j)=1).  do if (zdid=0).  compute outv={outv,ztmp}.  do if (ncs > 0 and ziscov > 0).  compute ccmatoff((i-1),ziscov)=0.  end if.  compute modlabel={modlabel;zcatlab(1:nzvls,1)}.  compute zdid=1.  do if (ztmpuse=0).  compute fulldat={fulldat,ztmp}.  do if (ncs > 0 and ziscov > 0).  compute ccmatoff((i-1),ziscov)=0.  end if.  compute ztmpuse=1.  loop k4=datcount to (datcount+(nzvls-1)).  compute ztmploc={ztmploc;k4}.  end loop.  compute ztmploc=ztmploc(2:nrow(ztmploc),1).  compute datcount=datcount+nzvls.  end if.  compute datindx(start:(start+nrow(ztmploc)-1),(i-1))=ztmploc.  compute wherez(1,(i-1))=start+1.  compute wherez(2,(i-1))=start+nrow(ztmploc)-1+1.  compute start=start+nrow(ztmploc).  end if.  loop k2 = 1 to nzvls.  compute outv={outv,(mtmp(:,(j-1))&*ztmp(:,k2))}.  do if (ncs > 0 and ziscov > 0).  compute ccmatoff((i-1),ziscov)=0.  end if.  compute modlabel={modlabel;intlab(cntmp,1)}.  compute intkey={intkey;intlab(cntmp,1),':', mnames(1,(j-1)),'x',zcatlab(k2,1),' ',' '}.  compute cntmp=cntmp+1.  end loop.  do if (mztmpus(1,(j-1))=0).  compute fulldat={fulldat,outv(:,(ncol(outv)-nzvls+1):ncol(outv))}.  do if (ncs > 0 and ziscov > 0).  compute ccmatoff((i-1),ziscov)=0.  end if.  compute mztmpus(1,(j-1))=1.  compute mz22=-999.  loop k4=datcount to (datcount+(nzvls-1)).  compute mz22={mz22;k4}.  end loop.  compute mztmplo(:,(j-1))=mz22(2:nrow(mz22),1).  compute datcount=datcount+nzvls.  end if.  compute datindx(start:(start+nrow(mztmplo)-1),(i-1))=mztmplo(:,(j-1)).  compute wheremz(((2*j)-3),(i-1))=start+1.  compute wheremz(((2*j)-2),(i-1))=start+nrow(mztmplo)-1+1.  compute onebl=make(nrow(mztmplo),1,1).  compute zhigh((start+1):(start+nrow(mztmplo)),zhighct)=onebl.  compute start=start+nrow(mztmplo).  end if.  end loop.  end if.  do if (wzsum > 0).  loop j = 1 to (i-1).  compute wzhighct=wzhighct+1.  do if (j = 1 and wzcmat(i,j)=1).  do if (wzdid=0).  loop k1=1 to nwvls.  loop k2 = 1 to nzvls.  compute outv={outv,(wtmp(:,k1)&*ztmp(:,k2))}.  do if (ncs > 0 and (ziscov > 0)).  compute ccmatoff((i-1),ziscov)=0.  end if.  do if (ncs > 0 and (wiscov > 0)).  compute ccmatoff((i-1),wiscov)=0.  end if.  compute modlabel={modlabel;intlab(cntmp,1)}.  compute intkey={intkey;intlab(cntmp,1),':',wcatlab(k1,1),'x',zcatlab(k2,1),' ',' '}.  compute cntmp=cntmp+1.  end loop.  end loop.  do if (wztmpus=0).  compute fulldat={fulldat,outv(:,(ncol(outv)-(nwvls*nzvls)+1):ncol(outv))}.  do if (ncs > 0 and (ziscov > 0)).  compute ccmatoff((i-1),ziscov)=0.  end if.  do if (ncs > 0 and (wiscov > 0)).  compute ccmatoff((i-1),wiscov)=0.  end if.  compute wztmpus=1.  loop k4=datcount to (datcount+((nwvls*nzvls)-1)).  compute wztmplo={wztmplo;k4}.  end loop.  compute wztmplo=wztmplo(2:nrow(wztmplo),1).  compute datcount=datcount+(nzvls*nwvls).  end if.  compute wzdid=1.  end if.  compute datindx(start:(start+nrow(wztmplo)-1),(i-1))=wztmplo.  compute wherewz(1,(i-1))=start+1.  compute wherewz(2,(i-1))=start+nrow(wztmplo)-1+1.  compute start=start+nrow(wztmplo).  loop k1=1 to nxvls.  loop k2=1 to nwvls.  loop k3=1 to nzvls.  compute outv={outv,(xtmp(:,k1)&*wtmp(:,k2)&*ztmp(:,k3))}.  do if (ncs > 0 and (ziscov > 0)).  compute ccmatoff((i-1),ziscov)=0.  end if.  do if (ncs > 0 and (wiscov > 0)).  compute ccmatoff((i-1),wiscov)=0.  end if.  compute modlabel={modlabel;intlab(cntmp,1)}.  compute intkey={intkey;intlab(cntmp,1),':',xcatlab(k1,1),'x',wcatlab(k2,1),'x', zcatlab(k3,1)}.  compute cntmp=cntmp+1.  end loop.  end loop.  end loop.  do if (xwztmpu=0).  compute fulldat={fulldat,outv(:,(ncol(outv)-(nxvls*nwvls*nzvls)+1):ncol(outv))}.  do if (ncs > 0 and (ziscov > 0)).  compute ccmatoff((i-1),ziscov)=0.  end if.  do if (ncs > 0 and (wiscov > 0)).  compute ccmatoff((i-1),wiscov)=0.  end if.  compute xwztmpu=1.  loop k4=datcount to (datcount+((nzvls*nxvls*nwvls)-1)).  compute xwztmplo={xwztmplo;k4}.  end loop.  compute xwztmplo=xwztmplo(2:nrow(xwztmplo),1).  compute datcount=datcount+(nxvls*nzvls*nwvls).  end if.  compute datindx(start:(start+nrow(xwztmplo)-1),(i-1))=xwztmplo.  compute wherexwz(1,(i-1))=start+1.  compute wherexwz(2,(i-1))=start+nrow(xwztmplo)-1+1.  compute onebl=make(nrow(xwztmplo),1,1).  compute wzhigh((start+1):(start+nrow(xwztmplo)),wzhighct)=onebl.  compute start=start+nrow(xwztmplo).  end if.  do if (j > 1 and wzcmat(i,j)=1).  do if (wzdid=0).  loop k1=1 to nwvls.  loop k2 = 1 to nzvls.  compute outv={outv,(wtmp(:,k1)&*ztmp(:,k2))}.  do if (ncs > 0 and (ziscov > 0)).  compute ccmatoff((i-1),ziscov)=0.  end if.  do if (ncs > 0 and (wiscov > 0)).  compute ccmatoff((i-1),wiscov)=0.  end if.  compute modlabel={modlabel;intlab(cntmp,1)}.  compute intkey={intkey;intlab(cntmp,1),':',wcatlab(k1,1),'x',zcatlab(k2,1),' ',' '}.  compute cntmp=cntmp+1.  end loop.  end loop.  do if (wztmpus=0).  compute fulldat={fulldat,outv(:,(ncol(outv)-(nwvls*nzvls)+1):ncol(outv))}.  do if (ncs > 0 and (ziscov > 0)).  compute ccmatoff((i-1),ziscov)=0.  end if.  do if (ncs > 0 and (wiscov > 0)).  compute ccmatoff((i-1),wiscov)=0.  end if.  compute wztmpus=1.  loop k4=datcount to (datcount+((nwvls*nzvls)-1)).  compute wztmplo={wztmplo;k4}.  end loop.  compute wztmplo=wztmplo(2:nrow(wztmplo),1).  compute datcount=datcount+(nzvls*nwvls).  end if.  compute wzdid=1.  compute datindx(start:(start+nrow(wztmplo)-1),(i-1))=wztmplo.  compute wherewz(1,(i-1))=start+1.  compute wherewz(2,(i-1))=start+nrow(wztmplo)-1+1.  compute start=start+nrow(wztmplo).  end if.  loop k1 = 1 to nwvls.  loop k2 = 1 to nzvls.  compute outv={outv,(mtmp(:,(j-1))&*wtmp(:,k1)&*ztmp(:,k2))}.  do if (ncs > 0 and (ziscov > 0)).  compute ccmatoff((i-1),ziscov)=0.  end if.  do if (ncs > 0 and (wiscov > 0)).  compute ccmatoff((i-1),wiscov)=0.  end if.  compute modlabel={modlabel;intlab(cntmp,1)}.  compute intkey={intkey;intlab(cntmp,1),':',mnames(1,(j-1)),'x',wcatlab(k1,1),'x', zcatlab(k2,1)}.  compute cntmp=cntmp+1.  end loop.  end loop.  do if (mwztmpu(1,(j-1))=0).  compute fulldat={fulldat,outv(:,(ncol(outv)-(nwvls*nzvls)+1):ncol(outv))}.  do if (ncs > 0 and (ziscov > 0)).  compute ccmatoff((i-1),ziscov)=0.  end if.  do if (ncs > 0 and (wiscov > 0)).  compute ccmatoff((i-1),wiscov)=0.  end if.  compute mwztmpu(1,(j-1))=1.  compute mz22=-999.  loop k4=datcount to (datcount+(nwvls*nzvls)-1).  compute mz22={mz22;k4}.  end loop.  compute mwztmplo(:,(j-1))=mz22(2:nrow(mz22),1).  compute datcount=datcount+(nwvls*nzvls).  end if.  compute datindx(start:(start+nrow(mwztmplo)-1),(i-1))=mwztmplo(:,(j-1)).  compute wheremwz(((2*j)-3),(i-1))=start+1.  compute wheremwz(((2*j)-2),(i-1))=start+nrow(mwztmplo)-1+1.  compute onebl=make(nrow(mwztmplo),1,1).  compute wzhigh((start+1):(start+nrow(mwztmplo)),wzhighct)=onebl.  compute start=start+nrow(mwztmplo).  end if.  end loop.  end if.  do if (ncs > 0).  compute ccmat=ccmat&*ccmatoff.  loop j = 1 to ncs.  do if (ccmat((i-1),j))=1.  do if (j=wiscov).  compute ctmp(:,j)=wtmp.  end if.  do if (j=ziscov).  compute ctmp(:,j)=ztmp.  end if.  compute outv={outv,ctmp(:,j)}.  compute modlabel={modlabel;covnames(1,j)}.  do if (ctmpuse(1,j)=0).  compute fulldat={fulldat,ctmp(:,j)}.  compute ctmpuse(1,j)=1.  compute ctmploc(1,j)=datcount.  compute datcount=datcount+1.  end if.  compute datindx(start:(start+nrow(ctmploc)-1),(i-1))=ctmploc(1,j).  compute start=start+nrow(ctmploc(1,j)).  end if.  end loop.  end if.  compute wdid=0.  compute zdid=0.  compute wzdid=0.  compute vlabs={vlabs;modlabel(2:nrow(modlabel),1)}.  compute numint(1,(i-1))=cntmp-1.  compute nump(1,(i-1))=nrow(modlabel)-1.  end loop.  release datcount, xtmpuse, wtmpuse, ztmpuse, xwtmpus, xztmpus, wztmpus, xwztmpu.  release xtmploc, wtmploc, xwtmplo, ztmploc, xztmplo, wztmplo, xwztmplo, foccnt.  do if (modcok=1 and ((nms > 0) or (zcmat(2,1) <> 1) or (mcx <> 0))).  compute notecode(notes,1) = 19.  compute notes = notes + 1.  compute modcok=0.  end if.  do if ((serial = 1 or (rsum(numint)>0) or nms=0) and mc > 0).  compute notecode(notes,1) = 15.  compute notes = notes + 1.  compute boot=mc.  compute mc=0.  end if.  do if (boot <> 0 or mc <> 0).  compute bootsz=boot.  do if (mc > 0).  compute bootsz=mc.  compute saveboot=0.  end if.  loop.  compute cilow = rnd(bootsz*(1-(conf/100))/2).  compute cihigh = trunc((bootsz*(conf/100)+(bootsz*(1-(conf/100))/2)))+1.  do if (cilow < 1 or cihigh > bootsz).  compute bootsz=trunc((bootsz+1000)/1000)*1000.  compute adjust = 1.  end if.  end loop if (cilow gt 0 and cihigh le bootsz).  do if (boot > 0).  compute boot=bootsz.  end if.  do if (mc > 0).  compute mc=bootsz.  end if.  do if (adjust = 1 and boot > 0).  compute notecode(notes,1) = 8.  compute notes = notes + 1.  end if.  do if (adjust = 1 and mc > 0).  compute notecode(notes,1) = 16.  compute notes = notes + 1.  end if.  end if.  compute maxboot = trunc(2*boot).  do if ( 0 > maxboot).  compute maxboot=trunc( 0 ).  end if.  do if (nms > 0).  release mtmpuse, mwtmpus, mwztmpu, mtmploc, mwtmplo, mztmplo, mwztmplo.  end if.  release wdid, zdid, wzdid, start,modlabel.  compute vlabs=vlabs(2:nrow(vlabs),1).  do if (rsum(numint) > 0).  compute intkey=intkey(2:nrow(intkey),:).  end if.  compute fulldat=fulldat(:,2:ncol(fulldat)).  compute fochigh=fochigh(1:rmax(nump),:).  compute whigh=whigh(1:rmax(nump),:).  compute zhigh=zhigh(1:rmax(nump),:).  compute wzhigh=wzhigh(1:rmax(nump),:).  compute coeffs=fochigh+whigh+zhigh+wzhigh.  compute bootloc=make(rmax(nump),ncol(nump),0).  do if (nms > 0).  compute cntmp=1.  loop i = 1 to ncol(nump).  loop j = 1 to nump(1,i).  compute bootloc(j,i)=cntmp.  compute cntmp=cntmp+1.  end loop.  end loop.  compute fochighb=make(nrow(fochigh),ncol(fochigh),0).  compute whighb=fochighb.  compute zhighb=fochighb.  compute wzhighb=fochighb.  compute thetaxmb=make(nrow(fochighb),nms,0).  compute thetaxyb=make(nrow(fochighb),1,0).  compute pathsfoc=make(nxvls,1,0).  compute cntmp=1.  loop i = 1 to (nms+nys).  loop j = 1 to i.  compute fochighb(:,cntmp)=fochigh(:,cntmp)&*bootloc(:,i).  compute whighb(:,cntmp)=whigh(:,cntmp)&*bootloc(:,i).  compute zhighb(:,cntmp)=zhigh(:,cntmp)&*bootloc(:,i).  compute wzhighb(:,cntmp)=wzhigh(:,cntmp)&*bootloc(:,i).  compute coeffsb=fochighb+whighb+zhighb+wzhighb.  do if ((i < (nms+nys)) and (j = 1)).  compute thetaxmb(:,i)=coeffsb(:,cntmp).  end if.  do if ((i = (nms+nys)) and (j = 1)).  compute thetaxyb(:,1)=coeffsb(:,cntmp).  end if.  compute cntmp=cntmp+1.  end loop.  end loop.  compute thetamyb=coeffsb(:,(ncol(coeffsb)-nms+1):ncol(coeffsb)).  do if (serial = 1).  compute thetammb=make(nrow(coeffsb),((nms*(nms-1))/2),0).  end if.  compute cntmp=1.  do if (nms > 1 and serial = 1).  loop i = 1 to (nms-1).  compute start=((i+2)*(i+1))/2.  loop j = 2 to (nms-i+1).  compute thetammb(:,cntmp)=coeffsb(:,start).  compute start=start+j+i-1.  compute cntmp=cntmp+1.  end loop.  end loop.  end if.  end if.  do if ((total = 1) and rsum(numint)=0).  compute dototal=1.  do if ((csum(bcmat(:,1)) <> (nms+nys)) or (rsum(bcmat(nrow(bcmat),:)) <> (nms+nys))).  compute dototal=0.  compute notecode(notes,1) = 12.  compute notes = notes + 1.  end if.  do if (ncs > 0).  do if ((csum(rsum(ccmat))) < (nrow(ccmat)*ncol(ccmat))).  compute dototal=0.  compute notecode(notes,1) = 11.  compute notes = notes + 1.  end if.  end if.  end if.  end if.  do if (criterr=0 and ncs > 0).  do if (rsum((csum(ccmat)=0)) <> 0).  compute errcode(errs,1)=51.  compute errs=errs+1.  compute criterr=1.  end if.  end if.  compute debug= 0.  print/title = '**************** PROCESS Procedure for SPSS Version 3.4.1 ****************'.  print/title = ' Written by Andrew F.Hayes, Ph.D.www.afhayes.com'.  print/title = ' Documentation available in Hayes (2018).www.guilford.com/p/hayes3'/space=0.  do if (criterr=0).  do if (stand=1 and ydich=1).  compute stand=0.  end if.  compute anymod2=csum(rsum(wcmat+zcmat+wzcmat)).  do if (anymod2 > 0 and stand = 1).  compute notecode(notes,1) = 27.  compute notes = notes + 1.  compute stand=0.  end if.  compute funny=1.  print modelvar/title = '**************************************************************************'/format = A8/rnames=modelvlb.  do if (ncs > 0).  print covnames/title='Covariates:'/format=A8.  end if.  print n/title='Sample'/rlabel='Size:'.  do if (( 'random' <> 'random')).  compute seedt= 'random'.  print seedt/title='Custom'/format=A12/rlabel = 'Seed:'.  end if.  do if (mcxok=1).  compute labtmp={xnames,t(xcatlab(1:nxvls,1))}.  print dummatx/title = 'Coding of categorical X variable for analysis:'/cnames = labtmp/format = F6.3.  end if.  do if (mcwok=1).  compute labtmp={wnames,t(wcatlab(1:nwvls,1))}.  print dummatw/title = 'Coding of categorical W variable for analysis:'/cnames = labtmp/format = F6.3.  end if.  do if (mczok=1).  compute labtmp={znames,t(zcatlab(1:nzvls,1))}.  print dummatz/title = 'Coding of categorical Z variable for analysis:'/cnames = labtmp/format = F6.3.  end if.  end if.  do if (criterr = 0).  compute outnames=ynames.  compute outvars=ytmp.  do if (nms > 0).  compute outnames={mnames,ynames}.  compute outvars={mtmp,ytmp}.  compute indcov=make(((nms*2)+(nms*(nxvls-1))),((nms*2)+(nms*(nxvls-1))),0).  compute mcsopath=make(((nms*2)+(nms*(nxvls-1))),1,0).  end if.  compute labstart=1.  compute intstart=1.  compute start=1.  compute coeffmat=make(1,6,0).  compute conseq={' '}.  compute dfmat=0.  compute coeffcol=0.  compute pathscnt=1.  compute pathscn2=1.  loop i = 1 to (nms+nys).  print/title = '**************************************************************************'.  compute highf=make(1,5,0).  compute highf2=highf.  do if ((i = (nms+nys)) and (ydich=1)).  compute highf=make(1,3,0).  compute highf2=highf.  end if.  compute flabel={' '}.  compute y=outvars(:,i).  compute xindx=datindx(1:(nump(1,i)-1),i).  compute x = fulldat(:,xindx).  compute x={ones,x}.  compute xsq=t(x)*x.  compute exsq=eval(xsq).  release xsq.  compute zeroeig=csum(exsq <= 0.000000000002).  print outnames(1,i)/title = 'OUTCOME VARIABLE:'/format = A8/space=0.  do if (ydich=1 and (i = (nms+nys))).  compute nmsd = {outnames(1,i), 'Analysis'}.  print rcd/title = 'Coding of binary Y for logistic regression analysis:'/cnames = nmsd/format = F9.2.  end if.  do if (zeroeig > 0).  print / title = 'SINGULAR OR NEAR SINGULAR DATA MATRIX.'.  compute criterr=1.  compute errcode(errs,1)=31.  compute errs=errs+1.  end if.  compute means=csum(x)/n.  compute vlabsm=vlabs(labstart:(labstart+(nump(1,i)-1)),1).  do if (criterr=0).  do if (ydich=0 or (i < (nms+nys))).  .  do if ( 1 =1).  compute b = inv(t( x )* x )*t( x )* y.  compute modres=b.  do if ( 1 =1).  compute n1=nrow( x ).  compute dfres=n1-(ncol( x )).  compute sstotal = t( y -(csum( y )/n1))*( y -(csum( y )/n1)).  compute resid= y - x *b.  compute ssresid = csum((resid)&**2).  compute r2 = (sstotal-ssresid)/sstotal.  compute adjr2 = 1-((1-r2)*(n1-1)/(dfres)).  compute mse=ssresid/(n1-ncol( x )).  .  compute n1=nrow( x ).  compute invXtX = inv(t( x )* x ).  compute varb = mse *invXtX.  compute k3 = ncol( x ).  compute xhc=0.  do if ( hc <> 5).  compute xhc= x.  compute hat = xhc(:,1).  loop i3=1 to nrow(xhc).  compute hat(i3,1)= xhc(i3,:)*invXtX*t(xhc(i3,:)).  end loop.  do if ( hc = 0 or hc =1).  loop i3 = 1 to k3.  compute xhc(:,i3)=xhc(:,i3)&* resid.  end loop.  end if.  do if ( hc =3 or hc =2).  loop i3=1 to k3.  compute xhc(:,i3) = ( resid &/(1-hat)&**(1/(4hc )))&*xhc(:,i3).  end loop.  end if.  do if ( hc = 4).  compute hcmn=make(n,2,4).  compute hcmn(:,2)=(n1*hat)/k3.  loop i3= 1 to k3.  compute xhc(:,i3) = ( resid &/(1-hat)&**(rmin(hcmn)/2))&*xhc(:,i3).  end loop.  end if.  compute varb=(invXtX*t(xhc)*xhc*invXtX).  do if ( hc =1).  compute varb=(n1/(n1-ncol( x )))&*varb.  end if.  end if.  compute hclab={'se(HC0)','se(HC1)','se(HC2)','se(HC3)','se(HC4)','se'}.  compute hclab=hclab(1,( hc +1)).  compute hcflab={'F(HC0)','F(HC1)','F(HC2)','F(HC3)','F(HC4)','F'}.  compute hcflab=hcflab(1,( hc +1)).  release xhc  .  compute seb=sqrt(diag(varb)).  compute trat = b&/seb.  compute p = 2*(1-tcdf(abs(trat), (dfres))).  compute tval = sqrt(dfres* (exp((dfres-(5/6))*((xp2/(dfres-(2/3)+(.11/dfres)))* (xp2/(dfres-(2/3)+(.11/dfres)))))-1)).  compute modres={modres,seb,trat,p}.  compute modres={modres,(b-tval&*seb),(b+tval&*seb)}.  compute modresl={'coeff',hclab,'t','p','LLCI','ULCI'}.  compute lmat = ident(ncol( x )).  compute lmat = lmat(:,2:ncol(lmat)).  compute fratio = (t(t(lmat)*b)*inv(t(lmat)*varb*lmat)*((t(lmat)*b)))/(ncol( x )-1).  compute pfr = 1-fcdf(fratio,(ncol( x )-1),dfres).  compute modsum={sqrt(r2),r2,mse,fratio,(ncol( x )-1),dfres,pfr}.  compute modsuml={'R','R-sq','MSE',hcflab,'df1','df2', 'p'}.  end if.  end if.  do if ( 1 = 2 or 1 =3).  compute xlp= x.  compute ylp= y.  compute pt2 = make(nrow(ylp),1,(csum(ylp)/nrow(ylp))).  do if ( 1 =2).  compute LL3 = ylp&*ln(pt2)+(1-ylp)&*ln(1-pt2).  end if.  compute LL3 = -2*csum(LL3).  compute bt1 = make(ncol(xlp),1,0).  compute LL1 = 0.  compute pt1 = make(nrow(ylp),1,0.5).  compute pt1lp=pt1.  loop jjj = 1 to iterate.  compute vt1 = mdiag(pt1lp&*(1-pt1lp)).  compute b = bt1+inv(t(xlp)*vt1*xlp)*t(xlp)*(ylp-pt1lp).  do if ( 1 =2).  compute xlpb=xlp*b.  compute xlpbt=(xlpb > -709.7).  compute xlpb709=(1-xlpbt)*(-709.7).  compute xlpb=(xlpb&*xlpbt)+xlpb709.  compute pt1lp = 1/(1+exp(-(xlpb))).  end if.  compute itprob = csum((pt1lp < .00000001) or (pt1lp > .9999999)).  do if (itprob > 0).  loop kkk = 1 to nrow(pt1lp).  do if (pt1lp(kkk,1) > .9999999).  compute pt1lp(kkk,1) = .9999999.  end if.  do if (pt1lp(kkk,1) < .00000001).  compute pt1lp(kkk,1) = .00000001.  end if.  end loop.  compute itprob = 0.  end if.  do if (itprob = 0).  do if ( 1 =2).  compute LL = ylp&*ln(pt1lp)+(1-ylp)&*ln(1-pt1lp).  end if.  compute LL2 = -2*csum(ll).  end if.  do if (abs(LL1-LL2) < converge).  do if ( 1 =1).  compute vt1 = mdiag(pt1lp&*(1-pt1lp)).  compute varb = inv(t(xlp)*vt1*xlp).  compute seb = sqrt(diag(varb)).  end if.  break.  end if.  compute bt1 = b.  compute LL1 = LL2.  end loop.  compute modres=b.  do if (jjj > iterate).  compute itprob = 2.  do if (booting=0).  compute iterrmod=1.  end if.  do if (booting=1).  compute bootiter=1.  end if.  do if (itprobtg=0).  compute itprobtg=1.  compute errcode(errs,1) = 47.  compute errs = errs + 1.  do if (booting = 0 and 1 =1).  compute vt1 = mdiag(pt1lp&*(1-pt1lp)).  compute varb = inv(t(xlp)*vt1*xlp).  compute seb = sqrt(diag(varb)).  end if.  end if.  end if.  do if ( 1 =1).  compute trat = b&/seb.  compute dfres=nrow(xlp).  compute p = 2*(1-cdfnorm(abs(trat))).  compute modres={modres,seb,trat,p}.  compute modres={modres,(b-xp2&*seb),(b+xp2&*seb)}.  compute pvchi=1-chicdf((LL3-LL2),(nrow(modres)-1)).  compute mcF = (LL3-LL2)/LL3.  compute cox = 1-exp(-(LL3-LL2)/nrow(xlp)).  compute nagel = cox/(1-exp(-(LL3)/nrow(xlp))).  compute modsum={LL2,(LL3-LL2),(nrow(modres)-1),pvchi, mcF,cox,nagel}.  compute modsuml={'-2LL','ModelLL', 'df', 'p', 'McFadden', 'CoxSnell', 'Nagelkrk'}.  compute modresl={'coeff','se','Z','p','LLCI','ULCI'}.  end if.  end if  .  compute dfmatt=make(nrow(modres),1,modsum(1,6)).  end if.  do if (ydich=1 and (i = (nms+nys))).  .  do if ( 2 =1).  compute b = inv(t( x )* x )*t( x )* y.  compute modres=b.  do if ( 1 =1).  compute n1=nrow( x ).  compute dfres=n1-(ncol( x )).  compute sstotal = t( y -(csum( y )/n1))*( y -(csum( y )/n1)).  compute resid= y - x *b.  compute ssresid = csum((resid)&**2).  compute r2 = (sstotal-ssresid)/sstotal.  compute adjr2 = 1-((1-r2)*(n1-1)/(dfres)).  compute mse=ssresid/(n1-ncol( x )).  .  compute n1=nrow( x ).  compute invXtX = inv(t( x )* x ).  compute varb = mse *invXtX.  compute k3 = ncol( x ).  compute xhc=0.  do if ( hc <> 5).  compute xhc= x.  compute hat = xhc(:,1).  loop i3=1 to nrow(xhc).  compute hat(i3,1)= xhc(i3,:)*invXtX*t(xhc(i3,:)).  end loop.  do if ( hc = 0 or hc =1).  loop i3 = 1 to k3.  compute xhc(:,i3)=xhc(:,i3)&* resid.  end loop.  end if.  do if ( hc =3 or hc =2).  loop i3=1 to k3.  compute xhc(:,i3) = ( resid &/(1-hat)&**(1/(4hc )))&*xhc(:,i3).  end loop.  end if.  do if ( hc = 4).  compute hcmn=make(n,2,4).  compute hcmn(:,2)=(n1*hat)/k3.  loop i3= 1 to k3.  compute xhc(:,i3) = ( resid &/(1-hat)&**(rmin(hcmn)/2))&*xhc(:,i3).  end loop.  end if.  compute varb=(invXtX*t(xhc)*xhc*invXtX).  do if ( hc =1).  compute varb=(n1/(n1-ncol( x )))&*varb.  end if.  end if.  compute hclab={'se(HC0)','se(HC1)','se(HC2)','se(HC3)','se(HC4)','se'}.  compute hclab=hclab(1,( hc +1)).  compute hcflab={'F(HC0)','F(HC1)','F(HC2)','F(HC3)','F(HC4)','F'}.  compute hcflab=hcflab(1,( hc +1)).  release xhc  .  compute seb=sqrt(diag(varb)).  compute trat = b&/seb.  compute p = 2*(1-tcdf(abs(trat), (dfres))).  compute tval = sqrt(dfres* (exp((dfres-(5/6))*((xp2/(dfres-(2/3)+(.11/dfres)))* (xp2/(dfres-(2/3)+(.11/dfres)))))-1)).  compute modres={modres,seb,trat,p}.  compute modres={modres,(b-tval&*seb),(b+tval&*seb)}.  compute modresl={'coeff',hclab,'t','p','LLCI','ULCI'}.  compute lmat = ident(ncol( x )).  compute lmat = lmat(:,2:ncol(lmat)).  compute fratio = (t(t(lmat)*b)*inv(t(lmat)*varb*lmat)*((t(lmat)*b)))/(ncol( x )-1).  compute pfr = 1-fcdf(fratio,(ncol( x )-1),dfres).  compute modsum={sqrt(r2),r2,mse,fratio,(ncol( x )-1),dfres,pfr}.  compute modsuml={'R','R-sq','MSE',hcflab,'df1','df2', 'p'}.  end if.  end if.  do if ( 2 = 2 or 2 =3).  compute xlp= x.  compute ylp= y.  compute pt2 = make(nrow(ylp),1,(csum(ylp)/nrow(ylp))).  do if ( 2 =2).  compute LL3 = ylp&*ln(pt2)+(1-ylp)&*ln(1-pt2).  end if.  compute LL3 = -2*csum(LL3).  compute bt1 = make(ncol(xlp),1,0).  compute LL1 = 0.  compute pt1 = make(nrow(ylp),1,0.5).  compute pt1lp=pt1.  loop jjj = 1 to iterate.  compute vt1 = mdiag(pt1lp&*(1-pt1lp)).  compute b = bt1+inv(t(xlp)*vt1*xlp)*t(xlp)*(ylp-pt1lp).  do if ( 2 =2).  compute xlpb=xlp*b.  compute xlpbt=(xlpb > -709.7).  compute xlpb709=(1-xlpbt)*(-709.7).  compute xlpb=(xlpb&*xlpbt)+xlpb709.  compute pt1lp = 1/(1+exp(-(xlpb))).  end if.  compute itprob = csum((pt1lp < .00000001) or (pt1lp > .9999999)).  do if (itprob > 0).  loop kkk = 1 to nrow(pt1lp).  do if (pt1lp(kkk,1) > .9999999).  compute pt1lp(kkk,1) = .9999999.  end if.  do if (pt1lp(kkk,1) < .00000001).  compute pt1lp(kkk,1) = .00000001.  end if.  end loop.  compute itprob = 0.  end if.  do if (itprob = 0).  do if ( 2 =2).  compute LL = ylp&*ln(pt1lp)+(1-ylp)&*ln(1-pt1lp).  end if.  compute LL2 = -2*csum(ll).  end if.  do if (abs(LL1-LL2) < converge).  do if ( 1 =1).  compute vt1 = mdiag(pt1lp&*(1-pt1lp)).  compute varb = inv(t(xlp)*vt1*xlp).  compute seb = sqrt(diag(varb)).  end if.  break.  end if.  compute bt1 = b.  compute LL1 = LL2.  end loop.  compute modres=b.  do if (jjj > iterate).  compute itprob = 2.  do if (booting=0).  compute iterrmod=1.  end if.  do if (booting=1).  compute bootiter=1.  end if.  do if (itprobtg=0).  compute itprobtg=1.  compute errcode(errs,1) = 47.  compute errs = errs + 1.  do if (booting = 0 and 1 =1).  compute vt1 = mdiag(pt1lp&*(1-pt1lp)).  compute varb = inv(t(xlp)*vt1*xlp).  compute seb = sqrt(diag(varb)).  end if.  end if.  end if.  do if ( 1 =1).  compute trat = b&/seb.  compute dfres=nrow(xlp).  compute p = 2*(1-cdfnorm(abs(trat))).  compute modres={modres,seb,trat,p}.  compute modres={modres,(b-xp2&*seb),(b+xp2&*seb)}.  compute pvchi=1-chicdf((LL3-LL2),(nrow(modres)-1)).  compute mcF = (LL3-LL2)/LL3.  compute cox = 1-exp(-(LL3-LL2)/nrow(xlp)).  compute nagel = cox/(1-exp(-(LL3)/nrow(xlp))).  compute modsum={LL2,(LL3-LL2),(nrow(modres)-1),pvchi, mcF,cox,nagel}.  compute modsuml={'-2LL','ModelLL', 'df', 'p', 'McFadden', 'CoxSnell', 'Nagelkrk'}.  compute modresl={'coeff','se','Z','p','LLCI','ULCI'}.  end if.  end if  .  compute dfmatt=make(nrow(modres),1,-999).  end if.  compute obscoeff={obscoeff,t(b)}.  print modsum/title = 'Model Summary'/cnames = modsuml/format= F10.4.  print modres/title='Model'/rnames=vlabsm/cnames=modresl/format= F10.4.  compute basemod=modsum(1,1).  compute basemodx=basemod.  do if (ydich=1 and (i = (nms+nys))).  print/title='These results are expressed in a log-odds metric.'.  compute notecode(notes,1) = 26.  compute notes = notes + 1.  end if.  compute coeffmat={coeffmat;modres}.  compute conseqt=make(nrow(modres),1,outnames(1,i)).  compute conseq={conseq;conseqt}.  compute dfmat={dfmat;dfmatt}.  compute labstart=labstart+nump(1,i).  do if (stand=1).  compute predsd=make(nrow(modres),1,0).  compute stdmod=modres(:,1)&/ovsd(1,i).  loop jd=1 to ncol(x).  compute descdat=x(:,jd).  compute predsd(jd,1) = (nrow(descdat)*sscp(descdat))-(t(csum(descdat))*(csum(descdat))).  compute predsd(jd,1) = sqrt(predsd(jd,1)/(nrow(descdat)*(nrow(descdat)-1))).  end loop.  do if (wherex(1,i) <> -999 and ((nxvls > 1) or (xdich=1))).  compute sdmsone=make(nxvls,1,1).  compute predsd(wherex(1,i):wherex(2,i),1)=sdmsone.  compute pstog=1.  end if.  compute predsd(1,1)=1.  compute stdmod=stdmod&*predsd.  compute stdmod=stdmod(2:nrow(stdmod),1).  compute sdvlabs=vlabsm(2:nrow(vlabsm),1).  print stdmod/title='Standardized coefficients'/clabels='coeff'/rnames=sdvlabs/format= F10.4.  end if.  do if (nms > 0 and serial = 0 and (rsum(numint) = 0) and (normal=1 or mc > 0)).  do if (i < (nms+nys)).  compute indcov((((i-1)*nxvls)+1):(i*nxvls),(((i-1)*nxvls)+1):(i*nxvls))=varb(2:(1+nxvls), 2:(1+nxvls)).  compute mcsopath((((i-1)*nxvls)+1):(i*nxvls) ,1)=modres(2:(1+nxvls),1).  end if.  do if (i = (nms+nys)).  compute atm=ncol(wherem).  compute indcov(((nms*nxvls)+1):nrow(mcsopath),((nms*nxvls)+1):nrow(mcsopath))= varb(wherem(1,atm):(wherem(1,atm)+nms-1),wherem(1,atm):(wherem(1,atm)+nms-1)).  compute mcsopath(((nms*nxvls)+1):nrow(mcsopath),1)=modres(wherem(1,atm):(wherem(1,atm)+ nms-1),1).  compute sobelok=1.  end if.  end if.  do if ((i = (nms+nys)) and (bcmat(nrow(bcmat),1)=1)).  compute direff=modres(2:(1+nxvls),:).  compute direfflb=modresl.  compute direffl2=vlabsm(2:(1+nxvls),:).  compute lmat=make(nrow(b),1,0).  compute lmat2=make(nxvls,1,1).  compute lmat(2:(1+nxvls),1)=lmat2.  do if (ydich <> 1).  .  compute lmat2= lmat.  do if ( 0 =0).  compute lmat2 = mdiag( lmat ).  compute lmat3=make(nrow(lmat2),1,0).  loop flp=1 to ncol(lmat2).  do if (csum(lmat2(:,flp))=1).  compute lmat3={lmat3,lmat2(:,flp)}.  end if.  end loop.  compute lmat2=lmat3(:,2:ncol(lmat3)).  end if.  compute fratio = (t(t(lmat2)* b )*inv(t(lmat2)* varb *lmat2)*((t(lmat2)* b )))/ncol(lmat2).  compute pfr = 1-fcdf(fratio,ncol(lmat2),(n-nrow( b ))).  compute fresult={fratio,ncol(lmat2),(n-nrow( b )),pfr}.  do if (i = (nms+nys) and (ydich=1)).  compute fratio=fratio*ncol(lmat2).  compute pfr=1-chicdf(fratio,ncol(lmat2)).  compute fresult={fratio,ncol(lmat2),pfr}.  end if.  do if ( 1 =1).  compute lmat3=1-rsum(lmat2).  compute xfm=make(n,csum(lmat3),0).  compute flpc=1.  loop flp=1 to nrow(lmat3).  do if (lmat3(flp,1)=1).  compute xfm(:,flpc)=x(:,flp).  compute flpc=flpc+1.  end if.  end loop.  compute bfm=inv(t(xfm)*xfm)*t(xfm)*y.  compute resid=y-(xfm*bfm).  compute sstotal=(y-(csum(y)/n)).  compute sstotal=csum(sstotal&*sstotal).  compute ssresid=csum(resid&*resid).  compute rsqch= r2 -((sstotal-ssresid)/sstotal).  compute fresult={rsqch,fresult}.  release xfm,flpc, resid, ssresid, bfm.  end if  .  compute diromni=fresult.  end if.  do if (ydich = 1 ).  .  compute btemphld=b.  compute llrdat=make(nrow(x),nrow( lmat )-csum( lmat ),-999).  compute llrdf=ncol(x)-ncol(llrdat).  compute llrcnt=0.  loop llri=1 to nrow( lmat ).  do if ( lmat (llri,1)=0).  compute llrcnt=llrcnt+1.  compute llrdat(:,llrcnt)=x(:,llri).  end if.  end loop.  .  do if ( 2 =1).  compute b = inv(t( llrdat )* llrdat )*t( llrdat )* y.  compute modres=b.  do if ( 0 =1).  compute n1=nrow( llrdat ).  compute dfres=n1-(ncol( llrdat )).  compute sstotal = t( y -(csum( y )/n1))*( y -(csum( y )/n1)).  compute resid= y - llrdat *b.  compute ssresid = csum((resid)&**2).  compute r2 = (sstotal-ssresid)/sstotal.  compute adjr2 = 1-((1-r2)*(n1-1)/(dfres)).  compute mse=ssresid/(n1-ncol( llrdat )).  .  compute n1=nrow( x ).  compute invXtX = inv(t( x )* x ).  compute varb = mse *invXtX.  compute k3 = ncol( x ).  compute xhc=0.  do if ( hc <> 5).  compute xhc= x.  compute hat = xhc(:,1).  loop i3=1 to nrow(xhc).  compute hat(i3,1)= xhc(i3,:)*invXtX*t(xhc(i3,:)).  end loop.  do if ( hc = 0 or hc =1).  loop i3 = 1 to k3.  compute xhc(:,i3)=xhc(:,i3)&* resid.  end loop.  end if.  do if ( hc =3 or hc =2).  loop i3=1 to k3.  compute xhc(:,i3) = ( resid &/(1-hat)&**(1/(4hc )))&*xhc(:,i3).  end loop.  end if.  do if ( hc = 4).  compute hcmn=make(n,2,4).  compute hcmn(:,2)=(n1*hat)/k3.  loop i3= 1 to k3.  compute xhc(:,i3) = ( resid &/(1-hat)&**(rmin(hcmn)/2))&*xhc(:,i3).  end loop.  end if.  compute varb=(invXtX*t(xhc)*xhc*invXtX).  do if ( hc =1).  compute varb=(n1/(n1-ncol( x )))&*varb.  end if.  end if.  compute hclab={'se(HC0)','se(HC1)','se(HC2)','se(HC3)','se(HC4)','se'}.  compute hclab=hclab(1,( hc +1)).  compute hcflab={'F(HC0)','F(HC1)','F(HC2)','F(HC3)','F(HC4)','F'}.  compute hcflab=hcflab(1,( hc +1)).  release xhc  .  compute seb=sqrt(diag(varb)).  compute trat = b&/seb.  compute p = 2*(1-tcdf(abs(trat), (dfres))).  compute tval = sqrt(dfres* (exp((dfres-(5/6))*((xp2/(dfres-(2/3)+(.11/dfres)))* (xp2/(dfres-(2/3)+(.11/dfres)))))-1)).  compute modres={modres,seb,trat,p}.  compute modres={modres,(b-tval&*seb),(b+tval&*seb)}.  compute modresl={'coeff',hclab,'t','p','LLCI','ULCI'}.  compute lmat = ident(ncol( llrdat )).  compute lmat = lmat(:,2:ncol(lmat)).  compute fratio = (t(t(lmat)*b)*inv(t(lmat)*varb*lmat)*((t(lmat)*b)))/(ncol( llrdat )-1).  compute pfr = 1-fcdf(fratio,(ncol( llrdat )-1),dfres).  compute modsum={sqrt(r2),r2,mse,fratio,(ncol( llrdat )-1),dfres,pfr}.  compute modsuml={'R','R-sq','MSE',hcflab,'df1','df2', 'p'}.  end if.  end if.  do if ( 2 = 2 or 2 =3).  compute xlp= llrdat.  compute ylp= y.  compute pt2 = make(nrow(ylp),1,(csum(ylp)/nrow(ylp))).  do if ( 2 =2).  compute LL3 = ylp&*ln(pt2)+(1-ylp)&*ln(1-pt2).  end if.  compute LL3 = -2*csum(LL3).  compute bt1 = make(ncol(xlp),1,0).  compute LL1 = 0.  compute pt1 = make(nrow(ylp),1,0.5).  compute pt1lp=pt1.  loop jjj = 1 to iterate.  compute vt1 = mdiag(pt1lp&*(1-pt1lp)).  compute b = bt1+inv(t(xlp)*vt1*xlp)*t(xlp)*(ylp-pt1lp).  do if ( 2 =2).  compute xlpb=xlp*b.  compute xlpbt=(xlpb > -709.7).  compute xlpb709=(1-xlpbt)*(-709.7).  compute xlpb=(xlpb&*xlpbt)+xlpb709.  compute pt1lp = 1/(1+exp(-(xlpb))).  end if.  compute itprob = csum((pt1lp < .00000001) or (pt1lp > .9999999)).  do if (itprob > 0).  loop kkk = 1 to nrow(pt1lp).  do if (pt1lp(kkk,1) > .9999999).  compute pt1lp(kkk,1) = .9999999.  end if.  do if (pt1lp(kkk,1) < .00000001).  compute pt1lp(kkk,1) = .00000001.  end if.  end loop.  compute itprob = 0.  end if.  do if (itprob = 0).  do if ( 2 =2).  compute LL = ylp&*ln(pt1lp)+(1-ylp)&*ln(1-pt1lp).  end if.  compute LL2 = -2*csum(ll).  end if.  do if (abs(LL1-LL2) < converge).  do if ( 0 =1).  compute vt1 = mdiag(pt1lp&*(1-pt1lp)).  compute varb = inv(t(xlp)*vt1*xlp).  compute seb = sqrt(diag(varb)).  end if.  break.  end if.  compute bt1 = b.  compute LL1 = LL2.  end loop.  compute modres=b.  do if (jjj > iterate).  compute itprob = 2.  do if (booting=0).  compute iterrmod=1.  end if.  do if (booting=1).  compute bootiter=1.  end if.  do if (itprobtg=0).  compute itprobtg=1.  compute errcode(errs,1) = 47.  compute errs = errs + 1.  do if (booting = 0 and 0 =1).  compute vt1 = mdiag(pt1lp&*(1-pt1lp)).  compute varb = inv(t(xlp)*vt1*xlp).  compute seb = sqrt(diag(varb)).  end if.  end if.  end if.  do if ( 0 =1).  compute trat = b&/seb.  compute dfres=nrow(xlp).  compute p = 2*(1-cdfnorm(abs(trat))).  compute modres={modres,seb,trat,p}.  compute modres={modres,(b-xp2&*seb),(b+xp2&*seb)}.  compute pvchi=1-chicdf((LL3-LL2),(nrow(modres)-1)).  compute mcF = (LL3-LL2)/LL3.  compute cox = 1-exp(-(LL3-LL2)/nrow(xlp)).  compute nagel = cox/(1-exp(-(LL3)/nrow(xlp))).  compute modsum={LL2,(LL3-LL2),(nrow(modres)-1),pvchi, mcF,cox,nagel}.  compute modsuml={'-2LL','ModelLL', 'df', 'p', 'McFadden', 'CoxSnell', 'Nagelkrk'}.  compute modresl={'coeff','se','Z','p','LLCI','ULCI'}.  end if.  end if  .  compute b=btemphld.  compute fresult={(LL2-basemod),llrdf,1-chicdf((LL2-basemod),llrdf)}  .  compute diromni=fresult.  end if.  end if.  do if (numint(1,i) > 0).  compute intkeym=intkey(intstart:(intstart+numint(1,i)-1),:).  print intkeym/title='Product terms key:'/format = A8.  end if.  do if (covcoeff=1).  print varb/title='Covariance matrix of regression parameter estimates:'/rnames=vlabsm /cnames=vlabsm/format= F10.4.  end if.  do if (model <> 74 and xmtest=1 and nms > 0).  compute r2tmp=r2.  compute btmp=b.  compute varbtmp=varb.  compute dfrestmp=dfres.  compute tvaltmp=tval.  compute xmtst=make(nms,4,0).  compute xmtstlbc={hcflab,'df1','df2','p'}.  do if ((i=(nms+nys)) and (ydich=1)).  compute xmtst=make(nms,3,0).  compute xmtstlbc={'Chi-sq','df','p'}.  end if.  compute xmtstlb={' '}.  compute xmtmat=x.  compute numxint=0.  loop xmint=2 to i.  compute x=xmtmat.  do if ((bcmat((i+1),xmint)=1) and (wzcmat((i+1),xmint) <> 1)).  do if (bcmat((i+1),1)=0).  compute x={xmtmat,xtmp}.  do if ((ydich=1) and (i=(nms+nys))).  .  do if ( 2 =1).  compute b = inv(t( x )* x )*t( x )* y.  compute modres=b.  do if ( 1 =1).  compute n1=nrow( x ).  compute dfres=n1-(ncol( x )).  compute sstotal = t( y -(csum( y )/n1))*( y -(csum( y )/n1)).  compute resid= y - x *b.  compute ssresid = csum((resid)&**2).  compute r2 = (sstotal-ssresid)/sstotal.  compute adjr2 = 1-((1-r2)*(n1-1)/(dfres)).  compute mse=ssresid/(n1-ncol( x )).  .  compute n1=nrow( x ).  compute invXtX = inv(t( x )* x ).  compute varb = mse *invXtX.  compute k3 = ncol( x ).  compute xhc=0.  do if ( hc <> 5).  compute xhc= x.  compute hat = xhc(:,1).  loop i3=1 to nrow(xhc).  compute hat(i3,1)= xhc(i3,:)*invXtX*t(xhc(i3,:)).  end loop.  do if ( hc = 0 or hc =1).  loop i3 = 1 to k3.  compute xhc(:,i3)=xhc(:,i3)&* resid.  end loop.  end if.  do if ( hc =3 or hc =2).  loop i3=1 to k3.  compute xhc(:,i3) = ( resid &/(1-hat)&**(1/(4hc )))&*xhc(:,i3).  end loop.  end if.  do if ( hc = 4).  compute hcmn=make(n,2,4).  compute hcmn(:,2)=(n1*hat)/k3.  loop i3= 1 to k3.  compute xhc(:,i3) = ( resid &/(1-hat)&**(rmin(hcmn)/2))&*xhc(:,i3).  end loop.  end if.  compute varb=(invXtX*t(xhc)*xhc*invXtX).  do if ( hc =1).  compute varb=(n1/(n1-ncol( x )))&*varb.  end if.  end if.  compute hclab={'se(HC0)','se(HC1)','se(HC2)','se(HC3)','se(HC4)','se'}.  compute hclab=hclab(1,( hc +1)).  compute hcflab={'F(HC0)','F(HC1)','F(HC2)','F(HC3)','F(HC4)','F'}.  compute hcflab=hcflab(1,( hc +1)).  release xhc  .  compute seb=sqrt(diag(varb)).  compute trat = b&/seb.  compute p = 2*(1-tcdf(abs(trat), (dfres))).  compute tval = sqrt(dfres* (exp((dfres-(5/6))*((xp2/(dfres-(2/3)+(.11/dfres)))* (xp2/(dfres-(2/3)+(.11/dfres)))))-1)).  compute modres={modres,seb,trat,p}.  compute modres={modres,(b-tval&*seb),(b+tval&*seb)}.  compute modresl={'coeff',hclab,'t','p','LLCI','ULCI'}.  compute lmat = ident(ncol( x )).  compute lmat = lmat(:,2:ncol(lmat)).  compute fratio = (t(t(lmat)*b)*inv(t(lmat)*varb*lmat)*((t(lmat)*b)))/(ncol( x )-1).  compute pfr = 1-fcdf(fratio,(ncol( x )-1),dfres).  compute modsum={sqrt(r2),r2,mse,fratio,(ncol( x )-1),dfres,pfr}.  compute modsuml={'R','R-sq','MSE',hcflab,'df1','df2', 'p'}.  end if.  end if.  do if ( 2 = 2 or 2 =3).  compute xlp= x.  compute ylp= y.  compute pt2 = make(nrow(ylp),1,(csum(ylp)/nrow(ylp))).  do if ( 2 =2).  compute LL3 = ylp&*ln(pt2)+(1-ylp)&*ln(1-pt2).  end if.  compute LL3 = -2*csum(LL3).  compute bt1 = make(ncol(xlp),1,0).  compute LL1 = 0.  compute pt1 = make(nrow(ylp),1,0.5).  compute pt1lp=pt1.  loop jjj = 1 to iterate.  compute vt1 = mdiag(pt1lp&*(1-pt1lp)).  compute b = bt1+inv(t(xlp)*vt1*xlp)*t(xlp)*(ylp-pt1lp).  do if ( 2 =2).  compute xlpb=xlp*b.  compute xlpbt=(xlpb > -709.7).  compute xlpb709=(1-xlpbt)*(-709.7).  compute xlpb=(xlpb&*xlpbt)+xlpb709.  compute pt1lp = 1/(1+exp(-(xlpb))).  end if.  compute itprob = csum((pt1lp < .00000001) or (pt1lp > .9999999)).  do if (itprob > 0).  loop kkk = 1 to nrow(pt1lp).  do if (pt1lp(kkk,1) > .9999999).  compute pt1lp(kkk,1) = .9999999.  end if.  do if (pt1lp(kkk,1) < .00000001).  compute pt1lp(kkk,1) = .00000001.  end if.  end loop.  compute itprob = 0.  end if.  do if (itprob = 0).  do if ( 2 =2).  compute LL = ylp&*ln(pt1lp)+(1-ylp)&*ln(1-pt1lp).  end if.  compute LL2 = -2*csum(ll).  end if.  do if (abs(LL1-LL2) < converge).  do if ( 1 =1).  compute vt1 = mdiag(pt1lp&*(1-pt1lp)).  compute varb = inv(t(xlp)*vt1*xlp).  compute seb = sqrt(diag(varb)).  end if.  break.  end if.  compute bt1 = b.  compute LL1 = LL2.  end loop.  compute modres=b.  do if (jjj > iterate).  compute itprob = 2.  do if (booting=0).  compute iterrmod=1.  end if.  do if (booting=1).  compute bootiter=1.  end if.  do if (itprobtg=0).  compute itprobtg=1.  compute errcode(errs,1) = 47.  compute errs = errs + 1.  do if (booting = 0 and 1 =1).  compute vt1 = mdiag(pt1lp&*(1-pt1lp)).  compute varb = inv(t(xlp)*vt1*xlp).  compute seb = sqrt(diag(varb)).  end if.  end if.  end if.  do if ( 1 =1).  compute trat = b&/seb.  compute dfres=nrow(xlp).  compute p = 2*(1-cdfnorm(abs(trat))).  compute modres={modres,seb,trat,p}.  compute modres={modres,(b-xp2&*seb),(b+xp2&*seb)}.  compute pvchi=1-chicdf((LL3-LL2),(nrow(modres)-1)).  compute mcF = (LL3-LL2)/LL3.  compute cox = 1-exp(-(LL3-LL2)/nrow(xlp)).  compute nagel = cox/(1-exp(-(LL3)/nrow(xlp))).  compute modsum={LL2,(LL3-LL2),(nrow(modres)-1),pvchi, mcF,cox,nagel}.  compute modsuml={'-2LL','ModelLL', 'df', 'p', 'McFadden', 'CoxSnell', 'Nagelkrk'}.  compute modresl={'coeff','se','Z','p','LLCI','ULCI'}.  end if.  end if  .  compute basemodx=LL2.  end if.  end if.  loop xmtlp1=1 to nxvls.  compute x={x,xtmp(:,xmtlp1)&*(mtmp(:,(xmint-1))-(csum(mtmp(:,(xmint-1)))/nrow(mtmp))) }.  end loop.  do if ((i < (nms+nys)) or (ydich=0)).  .  do if ( 1 =1).  compute b = inv(t( x )* x )*t( x )* y.  compute modres=b.  do if ( 1 =1).  compute n1=nrow( x ).  compute dfres=n1-(ncol( x )).  compute sstotal = t( y -(csum( y )/n1))*( y -(csum( y )/n1)).  compute resid= y - x *b.  compute ssresid = csum((resid)&**2).  compute r2 = (sstotal-ssresid)/sstotal.  compute adjr2 = 1-((1-r2)*(n1-1)/(dfres)).  compute mse=ssresid/(n1-ncol( x )).  .  compute n1=nrow( x ).  compute invXtX = inv(t( x )* x ).  compute varb = mse *invXtX.  compute k3 = ncol( x ).  compute xhc=0.  do if ( hc <> 5).  compute xhc= x.  compute hat = xhc(:,1).  loop i3=1 to nrow(xhc).  compute hat(i3,1)= xhc(i3,:)*invXtX*t(xhc(i3,:)).  end loop.  do if ( hc = 0 or hc =1).  loop i3 = 1 to k3.  compute xhc(:,i3)=xhc(:,i3)&* resid.  end loop.  end if.  do if ( hc =3 or hc =2).  loop i3=1 to k3.  compute xhc(:,i3) = ( resid &/(1-hat)&**(1/(4hc )))&*xhc(:,i3).  end loop.  end if.  do if ( hc = 4).  compute hcmn=make(n,2,4).  compute hcmn(:,2)=(n1*hat)/k3.  loop i3= 1 to k3.  compute xhc(:,i3) = ( resid &/(1-hat)&**(rmin(hcmn)/2))&*xhc(:,i3).  end loop.  end if.  compute varb=(invXtX*t(xhc)*xhc*invXtX).  do if ( hc =1).  compute varb=(n1/(n1-ncol( x )))&*varb.  end if.  end if.  compute hclab={'se(HC0)','se(HC1)','se(HC2)','se(HC3)','se(HC4)','se'}.  compute hclab=hclab(1,( hc +1)).  compute hcflab={'F(HC0)','F(HC1)','F(HC2)','F(HC3)','F(HC4)','F'}.  compute hcflab=hcflab(1,( hc +1)).  release xhc  .  compute seb=sqrt(diag(varb)).  compute trat = b&/seb.  compute p = 2*(1-tcdf(abs(trat), (dfres))).  compute tval = sqrt(dfres* (exp((dfres-(5/6))*((xp2/(dfres-(2/3)+(.11/dfres)))* (xp2/(dfres-(2/3)+(.11/dfres)))))-1)).  compute modres={modres,seb,trat,p}.  compute modres={modres,(b-tval&*seb),(b+tval&*seb)}.  compute modresl={'coeff',hclab,'t','p','LLCI','ULCI'}.  compute lmat = ident(ncol( x )).  compute lmat = lmat(:,2:ncol(lmat)).  compute fratio = (t(t(lmat)*b)*inv(t(lmat)*varb*lmat)*((t(lmat)*b)))/(ncol( x )-1).  compute pfr = 1-fcdf(fratio,(ncol( x )-1),dfres).  compute modsum={sqrt(r2),r2,mse,fratio,(ncol( x )-1),dfres,pfr}.  compute modsuml={'R','R-sq','MSE',hcflab,'df1','df2', 'p'}.  end if.  end if.  do if ( 1 = 2 or 1 =3).  compute xlp= x.  compute ylp= y.  compute pt2 = make(nrow(ylp),1,(csum(ylp)/nrow(ylp))).  do if ( 1 =2).  compute LL3 = ylp&*ln(pt2)+(1-ylp)&*ln(1-pt2).  end if.  compute LL3 = -2*csum(LL3).  compute bt1 = make(ncol(xlp),1,0).  compute LL1 = 0.  compute pt1 = make(nrow(ylp),1,0.5).  compute pt1lp=pt1.  loop jjj = 1 to iterate.  compute vt1 = mdiag(pt1lp&*(1-pt1lp)).  compute b = bt1+inv(t(xlp)*vt1*xlp)*t(xlp)*(ylp-pt1lp).  do if ( 1 =2).  compute xlpb=xlp*b.  compute xlpbt=(xlpb > -709.7).  compute xlpb709=(1-xlpbt)*(-709.7).  compute xlpb=(xlpb&*xlpbt)+xlpb709.  compute pt1lp = 1/(1+exp(-(xlpb))).  end if.  compute itprob = csum((pt1lp < .00000001) or (pt1lp > .9999999)).  do if (itprob > 0).  loop kkk = 1 to nrow(pt1lp).  do if (pt1lp(kkk,1) > .9999999).  compute pt1lp(kkk,1) = .9999999.  end if.  do if (pt1lp(kkk,1) < .00000001).  compute pt1lp(kkk,1) = .00000001.  end if.  end loop.  compute itprob = 0.  end if.  do if (itprob = 0).  do if ( 1 =2).  compute LL = ylp&*ln(pt1lp)+(1-ylp)&*ln(1-pt1lp).  end if.  compute LL2 = -2*csum(ll).  end if.  do if (abs(LL1-LL2) < converge).  do if ( 1 =1).  compute vt1 = mdiag(pt1lp&*(1-pt1lp)).  compute varb = inv(t(xlp)*vt1*xlp).  compute seb = sqrt(diag(varb)).  end if.  break.  end if.  compute bt1 = b.  compute LL1 = LL2.  end loop.  compute modres=b.  do if (jjj > iterate).  compute itprob = 2.  do if (booting=0).  compute iterrmod=1.  end if.  do if (booting=1).  compute bootiter=1.  end if.  do if (itprobtg=0).  compute itprobtg=1.  compute errcode(errs,1) = 47.  compute errs = errs + 1.  do if (booting = 0 and 1 =1).  compute vt1 = mdiag(pt1lp&*(1-pt1lp)).  compute varb = inv(t(xlp)*vt1*xlp).  compute seb = sqrt(diag(varb)).  end if.  end if.  end if.  do if ( 1 =1).  compute trat = b&/seb.  compute dfres=nrow(xlp).  compute p = 2*(1-cdfnorm(abs(trat))).  compute modres={modres,seb,trat,p}.  compute modres={modres,(b-xp2&*seb),(b+xp2&*seb)}.  compute pvchi=1-chicdf((LL3-LL2),(nrow(modres)-1)).  compute mcF = (LL3-LL2)/LL3.  compute cox = 1-exp(-(LL3-LL2)/nrow(xlp)).  compute nagel = cox/(1-exp(-(LL3)/nrow(xlp))).  compute modsum={LL2,(LL3-LL2),(nrow(modres)-1),pvchi, mcF,cox,nagel}.  compute modsuml={'-2LL','ModelLL', 'df', 'p', 'McFadden', 'CoxSnell', 'Nagelkrk'}.  compute modresl={'coeff','se','Z','p','LLCI','ULCI'}.  end if.  end if  .  end if.  do if ((i = (nms+nys)) and (ydich=1)).  .  do if ( 2 =1).  compute b = inv(t( x )* x )*t( x )* y.  compute modres=b.  do if ( 1 =1).  compute n1=nrow( x ).  compute dfres=n1-(ncol( x )).  compute sstotal = t( y -(csum( y )/n1))*( y -(csum( y )/n1)).  compute resid= y - x *b.  compute ssresid = csum((resid)&**2).  compute r2 = (sstotal-ssresid)/sstotal.  compute adjr2 = 1-((1-r2)*(n1-1)/(dfres)).  compute mse=ssresid/(n1-ncol( x )).  .  compute n1=nrow( x ).  compute invXtX = inv(t( x )* x ).  compute varb = mse *invXtX.  compute k3 = ncol( x ).  compute xhc=0.  do if ( hc <> 5).  compute xhc= x.  compute hat = xhc(:,1).  loop i3=1 to nrow(xhc).  compute hat(i3,1)= xhc(i3,:)*invXtX*t(xhc(i3,:)).  end loop.  do if ( hc = 0 or hc =1).  loop i3 = 1 to k3.  compute xhc(:,i3)=xhc(:,i3)&* resid.  end loop.  end if.  do if ( hc =3 or hc =2).  loop i3=1 to k3.  compute xhc(:,i3) = ( resid &/(1-hat)&**(1/(4hc )))&*xhc(:,i3).  end loop.  end if.  do if ( hc = 4).  compute hcmn=make(n,2,4).  compute hcmn(:,2)=(n1*hat)/k3.  loop i3= 1 to k3.  compute xhc(:,i3) = ( resid &/(1-hat)&**(rmin(hcmn)/2))&*xhc(:,i3).  end loop.  end if.  compute varb=(invXtX*t(xhc)*xhc*invXtX).  do if ( hc =1).  compute varb=(n1/(n1-ncol( x )))&*varb.  end if.  end if.  compute hclab={'se(HC0)','se(HC1)','se(HC2)','se(HC3)','se(HC4)','se'}.  compute hclab=hclab(1,( hc +1)).  compute hcflab={'F(HC0)','F(HC1)','F(HC2)','F(HC3)','F(HC4)','F'}.  compute hcflab=hcflab(1,( hc +1)).  release xhc  .  compute seb=sqrt(diag(varb)).  compute trat = b&/seb.  compute p = 2*(1-tcdf(abs(trat), (dfres))).  compute tval = sqrt(dfres* (exp((dfres-(5/6))*((xp2/(dfres-(2/3)+(.11/dfres)))* (xp2/(dfres-(2/3)+(.11/dfres)))))-1)).  compute modres={modres,seb,trat,p}.  compute modres={modres,(b-tval&*seb),(b+tval&*seb)}.  compute modresl={'coeff',hclab,'t','p','LLCI','ULCI'}.  compute lmat = ident(ncol( x )).  compute lmat = lmat(:,2:ncol(lmat)).  compute fratio = (t(t(lmat)*b)*inv(t(lmat)*varb*lmat)*((t(lmat)*b)))/(ncol( x )-1).  compute pfr = 1-fcdf(fratio,(ncol( x )-1),dfres).  compute modsum={sqrt(r2),r2,mse,fratio,(ncol( x )-1),dfres,pfr}.  compute modsuml={'R','R-sq','MSE',hcflab,'df1','df2', 'p'}.  end if.  end if.  do if ( 2 = 2 or 2 =3).  compute xlp= x.  compute ylp= y.  compute pt2 = make(nrow(ylp),1,(csum(ylp)/nrow(ylp))).  do if ( 2 =2).  compute LL3 = ylp&*ln(pt2)+(1-ylp)&*ln(1-pt2).  end if.  compute LL3 = -2*csum(LL3).  compute bt1 = make(ncol(xlp),1,0).  compute LL1 = 0.  compute pt1 = make(nrow(ylp),1,0.5).  compute pt1lp=pt1.  loop jjj = 1 to iterate.  compute vt1 = mdiag(pt1lp&*(1-pt1lp)).  compute b = bt1+inv(t(xlp)*vt1*xlp)*t(xlp)*(ylp-pt1lp).  do if ( 2 =2).  compute xlpb=xlp*b.  compute xlpbt=(xlpb > -709.7).  compute xlpb709=(1-xlpbt)*(-709.7).  compute xlpb=(xlpb&*xlpbt)+xlpb709.  compute pt1lp = 1/(1+exp(-(xlpb))).  end if.  compute itprob = csum((pt1lp < .00000001) or (pt1lp > .9999999)).  do if (itprob > 0).  loop kkk = 1 to nrow(pt1lp).  do if (pt1lp(kkk,1) > .9999999).  compute pt1lp(kkk,1) = .9999999.  end if.  do if (pt1lp(kkk,1) < .00000001).  compute pt1lp(kkk,1) = .00000001.  end if.  end loop.  compute itprob = 0.  end if.  do if (itprob = 0).  do if ( 2 =2).  compute LL = ylp&*ln(pt1lp)+(1-ylp)&*ln(1-pt1lp).  end if.  compute LL2 = -2*csum(ll).  end if.  do if (abs(LL1-LL2) < converge).  do if ( 1 =1).  compute vt1 = mdiag(pt1lp&*(1-pt1lp)).  compute varb = inv(t(xlp)*vt1*xlp).  compute seb = sqrt(diag(varb)).  end if.  break.  end if.  compute bt1 = b.  compute LL1 = LL2.  end loop.  compute modres=b.  do if (jjj > iterate).  compute itprob = 2.  do if (booting=0).  compute iterrmod=1.  end if.  do if (booting=1).  compute bootiter=1.  end if.  do if (itprobtg=0).  compute itprobtg=1.  compute errcode(errs,1) = 47.  compute errs = errs + 1.  do if (booting = 0 and 1 =1).  compute vt1 = mdiag(pt1lp&*(1-pt1lp)).  compute varb = inv(t(xlp)*vt1*xlp).  compute seb = sqrt(diag(varb)).  end if.  end if.  end if.  do if ( 1 =1).  compute trat = b&/seb.  compute dfres=nrow(xlp).  compute p = 2*(1-cdfnorm(abs(trat))).  compute modres={modres,seb,trat,p}.  compute modres={modres,(b-xp2&*seb),(b+xp2&*seb)}.  compute pvchi=1-chicdf((LL3-LL2),(nrow(modres)-1)).  compute mcF = (LL3-LL2)/LL3.  compute cox = 1-exp(-(LL3-LL2)/nrow(xlp)).  compute nagel = cox/(1-exp(-(LL3)/nrow(xlp))).  compute modsum={LL2,(LL3-LL2),(nrow(modres)-1),pvchi, mcF,cox,nagel}.  compute modsuml={'-2LL','ModelLL', 'df', 'p', 'McFadden', 'CoxSnell', 'Nagelkrk'}.  compute modresl={'coeff','se','Z','p','LLCI','ULCI'}.  end if.  end if  .  compute chidfxm=basemodx-LL2.  end if.  compute lmat=make(nrow(b),nxvls,0).  compute lmattmp=ident(nxvls).  compute lmat((nrow(lmat)-nxvls+1):nrow(lmat),:)=lmattmp.  .  compute lmat2= lmat.  do if ( 1 =0).  compute lmat2 = mdiag( lmat ).  compute lmat3=make(nrow(lmat2),1,0).  loop flp=1 to ncol(lmat2).  do if (csum(lmat2(:,flp))=1).  compute lmat3={lmat3,lmat2(:,flp)}.  end if.  end loop.  compute lmat2=lmat3(:,2:ncol(lmat3)).  end if.  compute fratio = (t(t(lmat2)* b )*inv(t(lmat2)* varb *lmat2)*((t(lmat2)* b )))/ncol(lmat2).  compute pfr = 1-fcdf(fratio,ncol(lmat2),(n-nrow( b ))).  compute fresult={fratio,ncol(lmat2),(n-nrow( b )),pfr}.  do if (i = (nms+nys) and (ydich=1)).  compute fratio=fratio*ncol(lmat2).  compute pfr=1-chicdf(fratio,ncol(lmat2)).  compute fresult={fratio,ncol(lmat2),pfr}.  end if.  do if ( 0 =1).  compute lmat3=1-rsum(lmat2).  compute xfm=make(n,csum(lmat3),0).  compute flpc=1.  loop flp=1 to nrow(lmat3).  do if (lmat3(flp,1)=1).  compute xfm(:,flpc)=x(:,flp).  compute flpc=flpc+1.  end if.  end loop.  compute bfm=inv(t(xfm)*xfm)*t(xfm)*y.  compute resid=y-(xfm*bfm).  compute sstotal=(y-(csum(y)/n)).  compute sstotal=csum(sstotal&*sstotal).  compute ssresid=csum(resid&*resid).  compute rsqch= 0 -((sstotal-ssresid)/sstotal).  compute fresult={rsqch,fresult}.  release xfm,flpc, resid, ssresid, bfm.  end if  .  compute numxint=numxint+1.  compute xmtst(numxint,:)=fresult.  do if ((i = (nms+nys)) and (ydich=1)).  compute xmtst(numxint,1)=chidfxm.  compute xmtst(numxint,3)=1-chicdf(chidfxm,nxvls).  end if.  compute xmtstlb={xmtstlb;highlbx((xmint-1),1)}.  end if.  end loop.  compute x=xmtmat.  release xmtmat.  do if (numxint>0).  compute xmtstlb=xmtstlb((2:(numxint+1)),:).  compute xmtst=xmtst(1:numxint,:).  do if (nms=1).  compute xmtstlb=' '.  end if.  do if ((i < (nms+nys)) or (ydich=0)).  print xmtst/title='Test(s) of X by M interaction:'/rnames=xmtstlb/cnames=xmtstlbc/format= F10.4.  end if.  do if ((i = (nms+nys)) and (ydich=1)).  print xmtst/title='Likelihood ratio test(s) of X by M interaction:'/rnames=xmtstlb/cnames=xmtstlbc/format= F10.4.  end if.  end if.  compute r2=r2tmp.  compute b=btmp.  compute varb=varbtmp.  compute dfres=dfrestmp.  compute tval=tvaltmp.  end if.  do if (criterr = 0).  compute jj=0.  loop j = start to ((start+i)-1).  compute dbint=0.  compute lmat=whigh(1:nump(1,i),j).  compute lmat2=wzhigh(1:nump(1,i),j).  do if ((csum(lmat) > 0) and (csum(lmat2) = 0)).  do if ((i < (nms+nys)) or (ydich <> 1)).  .  compute lmat2= lmat.  do if ( 0 =0).  compute lmat2 = mdiag( lmat ).  compute lmat3=make(nrow(lmat2),1,0).  loop flp=1 to ncol(lmat2).  do if (csum(lmat2(:,flp))=1).  compute lmat3={lmat3,lmat2(:,flp)}.  end if.  end loop.  compute lmat2=lmat3(:,2:ncol(lmat3)).  end if.  compute fratio = (t(t(lmat2)* b )*inv(t(lmat2)* varb *lmat2)*((t(lmat2)* b )))/ncol(lmat2).  compute pfr = 1-fcdf(fratio,ncol(lmat2),(n-nrow( b ))).  compute fresult={fratio,ncol(lmat2),(n-nrow( b )),pfr}.  do if (i = (nms+nys) and (ydich=1)).  compute fratio=fratio*ncol(lmat2).  compute pfr=1-chicdf(fratio,ncol(lmat2)).  compute fresult={fratio,ncol(lmat2),pfr}.  end if.  do if ( 1 =1).  compute lmat3=1-rsum(lmat2).  compute xfm=make(n,csum(lmat3),0).  compute flpc=1.  loop flp=1 to nrow(lmat3).  do if (lmat3(flp,1)=1).  compute xfm(:,flpc)=x(:,flp).  compute flpc=flpc+1.  end if.  end loop.  compute bfm=inv(t(xfm)*xfm)*t(xfm)*y.  compute resid=y-(xfm*bfm).  compute sstotal=(y-(csum(y)/n)).  compute sstotal=csum(sstotal&*sstotal).  compute ssresid=csum(resid&*resid).  compute rsqch= r2 -((sstotal-ssresid)/sstotal).  compute fresult={rsqch,fresult}.  release xfm,flpc, resid, ssresid, bfm.  end if  .  compute lmatdb=lmat.  compute dbint=dbint+1.  end if.  do if ((ydich = 1) and (i = (nms+nys))).  .  compute btemphld=b.  compute llrdat=make(nrow(x),nrow( lmat )-csum( lmat ),-999).  compute llrdf=ncol(x)-ncol(llrdat).  compute llrcnt=0.  loop llri=1 to nrow( lmat ).  do if ( lmat (llri,1)=0).  compute llrcnt=llrcnt+1.  compute llrdat(:,llrcnt)=x(:,llri).  end if.  end loop.  .  do if ( 2 =1).  compute b = inv(t( llrdat )* llrdat )*t( llrdat )* y.  compute modres=b.  do if ( 0 =1).  compute n1=nrow( llrdat ).  compute dfres=n1-(ncol( llrdat )).  compute sstotal = t( y -(csum( y )/n1))*( y -(csum( y )/n1)).  compute resid= y - llrdat *b.  compute ssresid = csum((resid)&**2).  compute r2 = (sstotal-ssresid)/sstotal.  compute adjr2 = 1-((1-r2)*(n1-1)/(dfres)).  compute mse=ssresid/(n1-ncol( llrdat )).  .  compute n1=nrow( x ).  compute invXtX = inv(t( x )* x ).  compute varb = mse *invXtX.  compute k3 = ncol( x ).  compute xhc=0.  do if ( hc <> 5).  compute xhc= x.  compute hat = xhc(:,1).  loop i3=1 to nrow(xhc).  compute hat(i3,1)= xhc(i3,:)*invXtX*t(xhc(i3,:)).  end loop.  do if ( hc = 0 or hc =1).  loop i3 = 1 to k3.  compute xhc(:,i3)=xhc(:,i3)&* resid.  end loop.  end if.  do if ( hc =3 or hc =2).  loop i3=1 to k3.  compute xhc(:,i3) = ( resid &/(1-hat)&**(1/(4hc )))&*xhc(:,i3).  end loop.  end if.  do if ( hc = 4).  compute hcmn=make(n,2,4).  compute hcmn(:,2)=(n1*hat)/k3.  loop i3= 1 to k3.  compute xhc(:,i3) = ( resid &/(1-hat)&**(rmin(hcmn)/2))&*xhc(:,i3).  end loop.  end if.  compute varb=(invXtX*t(xhc)*xhc*invXtX).  do if ( hc =1).  compute varb=(n1/(n1-ncol( x )))&*varb.  end if.  end if.  compute hclab={'se(HC0)','se(HC1)','se(HC2)','se(HC3)','se(HC4)','se'}.  compute hclab=hclab(1,( hc +1)).  compute hcflab={'F(HC0)','F(HC1)','F(HC2)','F(HC3)','F(HC4)','F'}.  compute hcflab=hcflab(1,( hc +1)).  release xhc  .  compute seb=sqrt(diag(varb)).  compute trat = b&/seb.  compute p = 2*(1-tcdf(abs(trat), (dfres))).  compute tval = sqrt(dfres* (exp((dfres-(5/6))*((xp2/(dfres-(2/3)+(.11/dfres)))* (xp2/(dfres-(2/3)+(.11/dfres)))))-1)).  compute modres={modres,seb,trat,p}.  compute modres={modres,(b-tval&*seb),(b+tval&*seb)}.  compute modresl={'coeff',hclab,'t','p','LLCI','ULCI'}.  compute lmat = ident(ncol( llrdat )).  compute lmat = lmat(:,2:ncol(lmat)).  compute fratio = (t(t(lmat)*b)*inv(t(lmat)*varb*lmat)*((t(lmat)*b)))/(ncol( llrdat )-1).  compute pfr = 1-fcdf(fratio,(ncol( llrdat )-1),dfres).  compute modsum={sqrt(r2),r2,mse,fratio,(ncol( llrdat )-1),dfres,pfr}.  compute modsuml={'R','R-sq','MSE',hcflab,'df1','df2', 'p'}.  end if.  end if.  do if ( 2 = 2 or 2 =3).  compute xlp= llrdat.  compute ylp= y.  compute pt2 = make(nrow(ylp),1,(csum(ylp)/nrow(ylp))).  do if ( 2 =2).  compute LL3 = ylp&*ln(pt2)+(1-ylp)&*ln(1-pt2).  end if.  compute LL3 = -2*csum(LL3).  compute bt1 = make(ncol(xlp),1,0).  compute LL1 = 0.  compute pt1 = make(nrow(ylp),1,0.5).  compute pt1lp=pt1.  loop jjj = 1 to iterate.  compute vt1 = mdiag(pt1lp&*(1-pt1lp)).  compute b = bt1+inv(t(xlp)*vt1*xlp)*t(xlp)*(ylp-pt1lp).  do if ( 2 =2).  compute xlpb=xlp*b.  compute xlpbt=(xlpb > -709.7).  compute xlpb709=(1-xlpbt)*(-709.7).  compute xlpb=(xlpb&*xlpbt)+xlpb709.  compute pt1lp = 1/(1+exp(-(xlpb))).  end if.  compute itprob = csum((pt1lp < .00000001) or (pt1lp > .9999999)).  do if (itprob > 0).  loop kkk = 1 to nrow(pt1lp).  do if (pt1lp(kkk,1) > .9999999).  compute pt1lp(kkk,1) = .9999999.  end if.  do if (pt1lp(kkk,1) < .00000001).  compute pt1lp(kkk,1) = .00000001.  end if.  end loop.  compute itprob = 0.  end if.  do if (itprob = 0).  do if ( 2 =2).  compute LL = ylp&*ln(pt1lp)+(1-ylp)&*ln(1-pt1lp).  end if.  compute LL2 = -2*csum(ll).  end if.  do if (abs(LL1-LL2) < converge).  do if ( 0 =1).  compute vt1 = mdiag(pt1lp&*(1-pt1lp)).  compute varb = inv(t(xlp)*vt1*xlp).  compute seb = sqrt(diag(varb)).  end if.  break.  end if.  compute bt1 = b.  compute LL1 = LL2.  end loop.  compute modres=b.  do if (jjj > iterate).  compute itprob = 2.  do if (booting=0).  compute iterrmod=1.  end if.  do if (booting=1).  compute bootiter=1.  end if.  do if (itprobtg=0).  compute itprobtg=1.  compute errcode(errs,1) = 47.  compute errs = errs + 1.  do if (booting = 0 and 0 =1).  compute vt1 = mdiag(pt1lp&*(1-pt1lp)).  compute varb = inv(t(xlp)*vt1*xlp).  compute seb = sqrt(diag(varb)).  end if.  end if.  end if.  do if ( 0 =1).  compute trat = b&/seb.  compute dfres=nrow(xlp).  compute p = 2*(1-cdfnorm(abs(trat))).  compute modres={modres,seb,trat,p}.  compute modres={modres,(b-xp2&*seb),(b+xp2&*seb)}.  compute pvchi=1-chicdf((LL3-LL2),(nrow(modres)-1)).  compute mcF = (LL3-LL2)/LL3.  compute cox = 1-exp(-(LL3-LL2)/nrow(xlp)).  compute nagel = cox/(1-exp(-(LL3)/nrow(xlp))).  compute modsum={LL2,(LL3-LL2),(nrow(modres)-1),pvchi, mcF,cox,nagel}.  compute modsuml={'-2LL','ModelLL', 'df', 'p', 'McFadden', 'CoxSnell', 'Nagelkrk'}.  compute modresl={'coeff','se','Z','p','LLCI','ULCI'}.  end if.  end if  .  compute b=btemphld.  compute fresult={(LL2-basemod),llrdf,1-chicdf((LL2-basemod),llrdf)}  .  compute lmatdb=lmat.  compute dbint=dbint+1.  end if.  compute highf={highf;fresult}.  compute highf2={highf2;fresult}.  do if (j = start).  compute flabel={flabel;'X*W'}.  end if.  do if (j > start).  do if (nms > 1).  compute flabel={flabel;highlbw(jj,1)}.  else if (nms = 1).  compute flabel={flabel;'M*W'}.  end if.  end if.  end if.  compute lmat=zhigh(1:nump(1,i),j).  compute lmat2=wzhigh(1:nump(1,i),j).  do if ((csum(lmat) > 0) and (csum(lmat2) = 0)).  do if ((i < (nms+nys)) or (ydich <> 1)).  .  compute lmat2= lmat.  do if ( 0 =0).  compute lmat2 = mdiag( lmat ).  compute lmat3=make(nrow(lmat2),1,0).  loop flp=1 to ncol(lmat2).  do if (csum(lmat2(:,flp))=1).  compute lmat3={lmat3,lmat2(:,flp)}.  end if.  end loop.  compute lmat2=lmat3(:,2:ncol(lmat3)).  end if.  compute fratio = (t(t(lmat2)* b )*inv(t(lmat2)* varb *lmat2)*((t(lmat2)* b )))/ncol(lmat2).  compute pfr = 1-fcdf(fratio,ncol(lmat2),(n-nrow( b ))).  compute fresult={fratio,ncol(lmat2),(n-nrow( b )),pfr}.  do if (i = (nms+nys) and (ydich=1)).  compute fratio=fratio*ncol(lmat2).  compute pfr=1-chicdf(fratio,ncol(lmat2)).  compute fresult={fratio,ncol(lmat2),pfr}.  end if.  do if ( 1 =1).  compute lmat3=1-rsum(lmat2).  compute xfm=make(n,csum(lmat3),0).  compute flpc=1.  loop flp=1 to nrow(lmat3).  do if (lmat3(flp,1)=1).  compute xfm(:,flpc)=x(:,flp).  compute flpc=flpc+1.  end if.  end loop.  compute bfm=inv(t(xfm)*xfm)*t(xfm)*y.  compute resid=y-(xfm*bfm).  compute sstotal=(y-(csum(y)/n)).  compute sstotal=csum(sstotal&*sstotal).  compute ssresid=csum(resid&*resid).  compute rsqch= r2 -((sstotal-ssresid)/sstotal).  compute fresult={rsqch,fresult}.  release xfm,flpc, resid, ssresid, bfm.  end if  .  compute dbint=dbint+1.  end if.  do if ((ydich = 1) and (i = (nms+nys))).  .  compute btemphld=b.  compute llrdat=make(nrow(x),nrow( lmat )-csum( lmat ),-999).  compute llrdf=ncol(x)-ncol(llrdat).  compute llrcnt=0.  loop llri=1 to nrow( lmat ).  do if ( lmat (llri,1)=0).  compute llrcnt=llrcnt+1.  compute llrdat(:,llrcnt)=x(:,llri).  end if.  end loop.  .  do if ( 2 =1).  compute b = inv(t( llrdat )* llrdat )*t( llrdat )* y.  compute modres=b.  do if ( 0 =1).  compute n1=nrow( llrdat ).  compute dfres=n1-(ncol( llrdat )).  compute sstotal = t( y -(csum( y )/n1))*( y -(csum( y )/n1)).  compute resid= y - llrdat *b.  compute ssresid = csum((resid)&**2).  compute r2 = (sstotal-ssresid)/sstotal.  compute adjr2 = 1-((1-r2)*(n1-1)/(dfres)).  compute mse=ssresid/(n1-ncol( llrdat )).  .  compute n1=nrow( x ).  compute invXtX = inv(t( x )* x ).  compute varb = mse *invXtX.  compute k3 = ncol( x ).  compute xhc=0.  do if ( hc <> 5).  compute xhc= x.  compute hat = xhc(:,1).  loop i3=1 to nrow(xhc).  compute hat(i3,1)= xhc(i3,:)*invXtX*t(xhc(i3,:)).  end loop.  do if ( hc = 0 or hc =1).  loop i3 = 1 to k3.  compute xhc(:,i3)=xhc(:,i3)&* resid.  end loop.  end if.  do if ( hc =3 or hc =2).  loop i3=1 to k3.  compute xhc(:,i3) = ( resid &/(1-hat)&**(1/(4hc )))&*xhc(:,i3).  end loop.  end if.  do if ( hc = 4).  compute hcmn=make(n,2,4).  compute hcmn(:,2)=(n1*hat)/k3.  loop i3= 1 to k3.  compute xhc(:,i3) = ( resid &/(1-hat)&**(rmin(hcmn)/2))&*xhc(:,i3).  end loop.  end if.  compute varb=(invXtX*t(xhc)*xhc*invXtX).  do if ( hc =1).  compute varb=(n1/(n1-ncol( x )))&*varb.  end if.  end if.  compute hclab={'se(HC0)','se(HC1)','se(HC2)','se(HC3)','se(HC4)','se'}.  compute hclab=hclab(1,( hc +1)).  compute hcflab={'F(HC0)','F(HC1)','F(HC2)','F(HC3)','F(HC4)','F'}.  compute hcflab=hcflab(1,( hc +1)).  release xhc  .  compute seb=sqrt(diag(varb)).  compute trat = b&/seb.  compute p = 2*(1-tcdf(abs(trat), (dfres))).  compute tval = sqrt(dfres* (exp((dfres-(5/6))*((xp2/(dfres-(2/3)+(.11/dfres)))* (xp2/(dfres-(2/3)+(.11/dfres)))))-1)).  compute modres={modres,seb,trat,p}.  compute modres={modres,(b-tval&*seb),(b+tval&*seb)}.  compute modresl={'coeff',hclab,'t','p','LLCI','ULCI'}.  compute lmat = ident(ncol( llrdat )).  compute lmat = lmat(:,2:ncol(lmat)).  compute fratio = (t(t(lmat)*b)*inv(t(lmat)*varb*lmat)*((t(lmat)*b)))/(ncol( llrdat )-1).  compute pfr = 1-fcdf(fratio,(ncol( llrdat )-1),dfres).  compute modsum={sqrt(r2),r2,mse,fratio,(ncol( llrdat )-1),dfres,pfr}.  compute modsuml={'R','R-sq','MSE',hcflab,'df1','df2', 'p'}.  end if.  end if.  do if ( 2 = 2 or 2 =3).  compute xlp= llrdat.  compute ylp= y.  compute pt2 = make(nrow(ylp),1,(csum(ylp)/nrow(ylp))).  do if ( 2 =2).  compute LL3 = ylp&*ln(pt2)+(1-ylp)&*ln(1-pt2).  end if.  compute LL3 = -2*csum(LL3).  compute bt1 = make(ncol(xlp),1,0).  compute LL1 = 0.  compute pt1 = make(nrow(ylp),1,0.5).  compute pt1lp=pt1.  loop jjj = 1 to iterate.  compute vt1 = mdiag(pt1lp&*(1-pt1lp)).  compute b = bt1+inv(t(xlp)*vt1*xlp)*t(xlp)*(ylp-pt1lp).  do if ( 2 =2).  compute xlpb=xlp*b.  compute xlpbt=(xlpb > -709.7).  compute xlpb709=(1-xlpbt)*(-709.7).  compute xlpb=(xlpb&*xlpbt)+xlpb709.  compute pt1lp = 1/(1+exp(-(xlpb))).  end if.  compute itprob = csum((pt1lp < .00000001) or (pt1lp > .9999999)).  do if (itprob > 0).  loop kkk = 1 to nrow(pt1lp).  do if (pt1lp(kkk,1) > .9999999).  compute pt1lp(kkk,1) = .9999999.  end if.  do if (pt1lp(kkk,1) < .00000001).  compute pt1lp(kkk,1) = .00000001.  end if.  end loop.  compute itprob = 0.  end if.  do if (itprob = 0).  do if ( 2 =2).  compute LL = ylp&*ln(pt1lp)+(1-ylp)&*ln(1-pt1lp).  end if.  compute LL2 = -2*csum(ll).  end if.  do if (abs(LL1-LL2) < converge).  do if ( 0 =1).  compute vt1 = mdiag(pt1lp&*(1-pt1lp)).  compute varb = inv(t(xlp)*vt1*xlp).  compute seb = sqrt(diag(varb)).  end if.  break.  end if.  compute bt1 = b.  compute LL1 = LL2.  end loop.  compute modres=b.  do if (jjj > iterate).  compute itprob = 2.  do if (booting=0).  compute iterrmod=1.  end if.  do if (booting=1).  compute bootiter=1.  end if.  do if (itprobtg=0).  compute itprobtg=1.  compute errcode(errs,1) = 47.  compute errs = errs + 1.  do if (booting = 0 and 0 =1).  compute vt1 = mdiag(pt1lp&*(1-pt1lp)).  compute varb = inv(t(xlp)*vt1*xlp).  compute seb = sqrt(diag(varb)).  end if.  end if.  end if.  do if ( 0 =1).  compute trat = b&/seb.  compute dfres=nrow(xlp).  compute p = 2*(1-cdfnorm(abs(trat))).  compute modres={modres,seb,trat,p}.  compute modres={modres,(b-xp2&*seb),(b+xp2&*seb)}.  compute pvchi=1-chicdf((LL3-LL2),(nrow(modres)-1)).  compute mcF = (LL3-LL2)/LL3.  compute cox = 1-exp(-(LL3-LL2)/nrow(xlp)).  compute nagel = cox/(1-exp(-(LL3)/nrow(xlp))).  compute modsum={LL2,(LL3-LL2),(nrow(modres)-1),pvchi, mcF,cox,nagel}.  compute modsuml={'-2LL','ModelLL', 'df', 'p', 'McFadden', 'CoxSnell', 'Nagelkrk'}.  compute modresl={'coeff','se','Z','p','LLCI','ULCI'}.  end if.  end if  .  compute b=btemphld.  compute fresult={(LL2-basemod),llrdf,1-chicdf((LL2-basemod),llrdf)}  .  compute dbint=dbint+1.  end if.  compute highf={highf;fresult}.  compute highf2={highf2;fresult}.  do if (j = start).  compute flabel={flabel;'X*Z'}.  end if.  do if (j > start).  do if (nms > 1).  compute flabel={flabel;highlbz(jj,1)}.  else if (nms = 1).  compute flabel={flabel;'M*Z'}.  end if.  end if.  end if.  do if (dbint=2).  compute lmatdb=lmatdb+lmat.  do if ((ydich = 1) and (i = (nms+nys))).  .  compute btemphld=b.  compute llrdat=make(nrow(x),nrow( lmatdb )-csum( lmatdb ),-999).  compute llrdf=ncol(x)-ncol(llrdat).  compute llrcnt=0.  loop llri=1 to nrow( lmatdb ).  do if ( lmatdb (llri,1)=0).  compute llrcnt=llrcnt+1.  compute llrdat(:,llrcnt)=x(:,llri).  end if.  end loop.  .  do if ( 2 =1).  compute b = inv(t( llrdat )* llrdat )*t( llrdat )* y.  compute modres=b.  do if ( 0 =1).  compute n1=nrow( llrdat ).  compute dfres=n1-(ncol( llrdat )).  compute sstotal = t( y -(csum( y )/n1))*( y -(csum( y )/n1)).  compute resid= y - llrdat *b.  compute ssresid = csum((resid)&**2).  compute r2 = (sstotal-ssresid)/sstotal.  compute adjr2 = 1-((1-r2)*(n1-1)/(dfres)).  compute mse=ssresid/(n1-ncol( llrdat )).  .  compute n1=nrow( x ).  compute invXtX = inv(t( x )* x ).  compute varb = mse *invXtX.  compute k3 = ncol( x ).  compute xhc=0.  do if ( hc <> 5).  compute xhc= x.  compute hat = xhc(:,1).  loop i3=1 to nrow(xhc).  compute hat(i3,1)= xhc(i3,:)*invXtX*t(xhc(i3,:)).  end loop.  do if ( hc = 0 or hc =1).  loop i3 = 1 to k3.  compute xhc(:,i3)=xhc(:,i3)&* resid.  end loop.  end if.  do if ( hc =3 or hc =2).  loop i3=1 to k3.  compute xhc(:,i3) = ( resid &/(1-hat)&**(1/(4hc )))&*xhc(:,i3).  end loop.  end if.  do if ( hc = 4).  compute hcmn=make(n,2,4).  compute hcmn(:,2)=(n1*hat)/k3.  loop i3= 1 to k3.  compute xhc(:,i3) = ( resid &/(1-hat)&**(rmin(hcmn)/2))&*xhc(:,i3).  end loop.  end if.  compute varb=(invXtX*t(xhc)*xhc*invXtX).  do if ( hc =1).  compute varb=(n1/(n1-ncol( x )))&*varb.  end if.  end if.  compute hclab={'se(HC0)','se(HC1)','se(HC2)','se(HC3)','se(HC4)','se'}.  compute hclab=hclab(1,( hc +1)).  compute hcflab={'F(HC0)','F(HC1)','F(HC2)','F(HC3)','F(HC4)','F'}.  compute hcflab=hcflab(1,( hc +1)).  release xhc  .  compute seb=sqrt(diag(varb)).  compute trat = b&/seb.  compute p = 2*(1-tcdf(abs(trat), (dfres))).  compute tval = sqrt(dfres* (exp((dfres-(5/6))*((xp2/(dfres-(2/3)+(.11/dfres)))* (xp2/(dfres-(2/3)+(.11/dfres)))))-1)).  compute modres={modres,seb,trat,p}.  compute modres={modres,(b-tval&*seb),(b+tval&*seb)}.  compute modresl={'coeff',hclab,'t','p','LLCI','ULCI'}.  compute lmat = ident(ncol( llrdat )).  compute lmat = lmat(:,2:ncol(lmat)).  compute fratio = (t(t(lmat)*b)*inv(t(lmat)*varb*lmat)*((t(lmat)*b)))/(ncol( llrdat )-1).  compute pfr = 1-fcdf(fratio,(ncol( llrdat )-1),dfres).  compute modsum={sqrt(r2),r2,mse,fratio,(ncol( llrdat )-1),dfres,pfr}.  compute modsuml={'R','R-sq','MSE',hcflab,'df1','df2', 'p'}.  end if.  end if.  do if ( 2 = 2 or 2 =3).  compute xlp= llrdat.  compute ylp= y.  compute pt2 = make(nrow(ylp),1,(csum(ylp)/nrow(ylp))).  do if ( 2 =2).  compute LL3 = ylp&*ln(pt2)+(1-ylp)&*ln(1-pt2).  end if.  compute LL3 = -2*csum(LL3).  compute bt1 = make(ncol(xlp),1,0).  compute LL1 = 0.  compute pt1 = make(nrow(ylp),1,0.5).  compute pt1lp=pt1.  loop jjj = 1 to iterate.  compute vt1 = mdiag(pt1lp&*(1-pt1lp)).  compute b = bt1+inv(t(xlp)*vt1*xlp)*t(xlp)*(ylp-pt1lp).  do if ( 2 =2).  compute xlpb=xlp*b.  compute xlpbt=(xlpb > -709.7).  compute xlpb709=(1-xlpbt)*(-709.7).  compute xlpb=(xlpb&*xlpbt)+xlpb709.  compute pt1lp = 1/(1+exp(-(xlpb))).  end if.  compute itprob = csum((pt1lp < .00000001) or (pt1lp > .9999999)).  do if (itprob > 0).  loop kkk = 1 to nrow(pt1lp).  do if (pt1lp(kkk,1) > .9999999).  compute pt1lp(kkk,1) = .9999999.  end if.  do if (pt1lp(kkk,1) < .00000001).  compute pt1lp(kkk,1) = .00000001.  end if.  end loop.  compute itprob = 0.  end if.  do if (itprob = 0).  do if ( 2 =2).  compute LL = ylp&*ln(pt1lp)+(1-ylp)&*ln(1-pt1lp).  end if.  compute LL2 = -2*csum(ll).  end if.  do if (abs(LL1-LL2) < converge).  do if ( 0 =1).  compute vt1 = mdiag(pt1lp&*(1-pt1lp)).  compute varb = inv(t(xlp)*vt1*xlp).  compute seb = sqrt(diag(varb)).  end if.  break.  end if.  compute bt1 = b.  compute LL1 = LL2.  end loop.  compute modres=b.  do if (jjj > iterate).  compute itprob = 2.  do if (booting=0).  compute iterrmod=1.  end if.  do if (booting=1).  compute bootiter=1.  end if.  do if (itprobtg=0).  compute itprobtg=1.  compute errcode(errs,1) = 47.  compute errs = errs + 1.  do if (booting = 0 and 0 =1).  compute vt1 = mdiag(pt1lp&*(1-pt1lp)).  compute varb = inv(t(xlp)*vt1*xlp).  compute seb = sqrt(diag(varb)).  end if.  end if.  end if.  do if ( 0 =1).  compute trat = b&/seb.  compute dfres=nrow(xlp).  compute p = 2*(1-cdfnorm(abs(trat))).  compute modres={modres,seb,trat,p}.  compute modres={modres,(b-xp2&*seb),(b+xp2&*seb)}.  compute pvchi=1-chicdf((LL3-LL2),(nrow(modres)-1)).  compute mcF = (LL3-LL2)/LL3.  compute cox = 1-exp(-(LL3-LL2)/nrow(xlp)).  compute nagel = cox/(1-exp(-(LL3)/nrow(xlp))).  compute modsum={LL2,(LL3-LL2),(nrow(modres)-1),pvchi, mcF,cox,nagel}.  compute modsuml={'-2LL','ModelLL', 'df', 'p', 'McFadden', 'CoxSnell', 'Nagelkrk'}.  compute modresl={'coeff','se','Z','p','LLCI','ULCI'}.  end if.  end if  .  compute b=btemphld.  compute fresult={(LL2-basemod),llrdf,1-chicdf((LL2-basemod),llrdf)}  .  end if.  do if (ydich <> 1 or i < (nms+nys)).  .  compute lmat2= lmatdb.  do if ( 0 =0).  compute lmat2 = mdiag( lmatdb ).  compute lmat3=make(nrow(lmat2),1,0).  loop flp=1 to ncol(lmat2).  do if (csum(lmat2(:,flp))=1).  compute lmat3={lmat3,lmat2(:,flp)}.  end if.  end loop.  compute lmat2=lmat3(:,2:ncol(lmat3)).  end if.  compute fratio = (t(t(lmat2)* b )*inv(t(lmat2)* varb *lmat2)*((t(lmat2)* b )))/ncol(lmat2).  compute pfr = 1-fcdf(fratio,ncol(lmat2),(n-nrow( b ))).  compute fresult={fratio,ncol(lmat2),(n-nrow( b )),pfr}.  do if (i = (nms+nys) and (ydich=1)).  compute fratio=fratio*ncol(lmat2).  compute pfr=1-chicdf(fratio,ncol(lmat2)).  compute fresult={fratio,ncol(lmat2),pfr}.  end if.  do if ( 1 =1).  compute lmat3=1-rsum(lmat2).  compute xfm=make(n,csum(lmat3),0).  compute flpc=1.  loop flp=1 to nrow(lmat3).  do if (lmat3(flp,1)=1).  compute xfm(:,flpc)=x(:,flp).  compute flpc=flpc+1.  end if.  end loop.  compute bfm=inv(t(xfm)*xfm)*t(xfm)*y.  compute resid=y-(xfm*bfm).  compute sstotal=(y-(csum(y)/n)).  compute sstotal=csum(sstotal&*sstotal).  compute ssresid=csum(resid&*resid).  compute rsqch= r2 -((sstotal-ssresid)/sstotal).  compute fresult={rsqch,fresult}.  release xfm,flpc, resid, ssresid, bfm.  end if  .  end if.  compute dbint=0.  compute highf={highf;fresult}.  do if (jj=0 and nms > 0).  compute flabel={flabel;'BOTH(X)'}.  end if.  do if (jj=0 and nms = 0).  compute flabel={flabel;'BOTH'}.  end if.  do if (jj>0 and nms = 1).  compute flabel={flabel;'BOTH(M)'}.  end if.  do if (nms > 1 and jj > 0).  compute flabel={flabel;highlbbt(jj,1)}.  end if.  end if.  compute lmat2=wzhigh(1:nump(1,i),j).  do if (csum(lmat2) > 0).  do if ((i < (nms+nys)) or (ydich <> 1)).  .  compute lmat2= lmat2.  do if ( 0 =0).  compute lmat2 = mdiag( lmat2 ).  compute lmat3=make(nrow(lmat2),1,0).  loop flp=1 to ncol(lmat2).  do if (csum(lmat2(:,flp))=1).  compute lmat3={lmat3,lmat2(:,flp)}.  end if.  end loop.  compute lmat2=lmat3(:,2:ncol(lmat3)).  end if.  compute fratio = (t(t(lmat2)* b )*inv(t(lmat2)* varb *lmat2)*((t(lmat2)* b )))/ncol(lmat2).  compute pfr = 1-fcdf(fratio,ncol(lmat2),(n-nrow( b ))).  compute fresult={fratio,ncol(lmat2),(n-nrow( b )),pfr}.  do if (i = (nms+nys) and (ydich=1)).  compute fratio=fratio*ncol(lmat2).  compute pfr=1-chicdf(fratio,ncol(lmat2)).  compute fresult={fratio,ncol(lmat2),pfr}.  end if.  do if ( 1 =1).  compute lmat3=1-rsum(lmat2).  compute xfm=make(n,csum(lmat3),0).  compute flpc=1.  loop flp=1 to nrow(lmat3).  do if (lmat3(flp,1)=1).  compute xfm(:,flpc)=x(:,flp).  compute flpc=flpc+1.  end if.  end loop.  compute bfm=inv(t(xfm)*xfm)*t(xfm)*y.  compute resid=y-(xfm*bfm).  compute sstotal=(y-(csum(y)/n)).  compute sstotal=csum(sstotal&*sstotal).  compute ssresid=csum(resid&*resid).  compute rsqch= r2 -((sstotal-ssresid)/sstotal).  compute fresult={rsqch,fresult}.  release xfm,flpc, resid, ssresid, bfm.  end if  .  end if.  do if ((ydich = 1) and (i = (nms+nys))).  .  compute btemphld=b.  compute llrdat=make(nrow(x),nrow( lmat2 )-csum( lmat2 ),-999).  compute llrdf=ncol(x)-ncol(llrdat).  compute llrcnt=0.  loop llri=1 to nrow( lmat2 ).  do if ( lmat2 (llri,1)=0).  compute llrcnt=llrcnt+1.  compute llrdat(:,llrcnt)=x(:,llri).  end if.  end loop.  .  do if ( 2 =1).  compute b = inv(t( llrdat )* llrdat )*t( llrdat )* y.  compute modres=b.  do if ( 0 =1).  compute n1=nrow( llrdat ).  compute dfres=n1-(ncol( llrdat )).  compute sstotal = t( y -(csum( y )/n1))*( y -(csum( y )/n1)).  compute resid= y - llrdat *b.  compute ssresid = csum((resid)&**2).  compute r2 = (sstotal-ssresid)/sstotal.  compute adjr2 = 1-((1-r2)*(n1-1)/(dfres)).  compute mse=ssresid/(n1-ncol( llrdat )).  .  compute n1=nrow( x ).  compute invXtX = inv(t( x )* x ).  compute varb = mse *invXtX.  compute k3 = ncol( x ).  compute xhc=0.  do if ( hc <> 5).  compute xhc= x.  compute hat = xhc(:,1).  loop i3=1 to nrow(xhc).  compute hat(i3,1)= xhc(i3,:)*invXtX*t(xhc(i3,:)).  end loop.  do if ( hc = 0 or hc =1).  loop i3 = 1 to k3.  compute xhc(:,i3)=xhc(:,i3)&* resid.  end loop.  end if.  do if ( hc =3 or hc =2).  loop i3=1 to k3.  compute xhc(:,i3) = ( resid &/(1-hat)&**(1/(4hc )))&*xhc(:,i3).  end loop.  end if.  do if ( hc = 4).  compute hcmn=make(n,2,4).  compute hcmn(:,2)=(n1*hat)/k3.  loop i3= 1 to k3.  compute xhc(:,i3) = ( resid &/(1-hat)&**(rmin(hcmn)/2))&*xhc(:,i3).  end loop.  end if.  compute varb=(invXtX*t(xhc)*xhc*invXtX).  do if ( hc =1).  compute varb=(n1/(n1-ncol( x )))&*varb.  end if.  end if.  compute hclab={'se(HC0)','se(HC1)','se(HC2)','se(HC3)','se(HC4)','se'}.  compute hclab=hclab(1,( hc +1)).  compute hcflab={'F(HC0)','F(HC1)','F(HC2)','F(HC3)','F(HC4)','F'}.  compute hcflab=hcflab(1,( hc +1)).  release xhc  .  compute seb=sqrt(diag(varb)).  compute trat = b&/seb.  compute p = 2*(1-tcdf(abs(trat), (dfres))).  compute tval = sqrt(dfres* (exp((dfres-(5/6))*((xp2/(dfres-(2/3)+(.11/dfres)))* (xp2/(dfres-(2/3)+(.11/dfres)))))-1)).  compute modres={modres,seb,trat,p}.  compute modres={modres,(b-tval&*seb),(b+tval&*seb)}.  compute modresl={'coeff',hclab,'t','p','LLCI','ULCI'}.  compute lmat = ident(ncol( llrdat )).  compute lmat = lmat(:,2:ncol(lmat)).  compute fratio = (t(t(lmat)*b)*inv(t(lmat)*varb*lmat)*((t(lmat)*b)))/(ncol( llrdat )-1).  compute pfr = 1-fcdf(fratio,(ncol( llrdat )-1),dfres).  compute modsum={sqrt(r2),r2,mse,fratio,(ncol( llrdat )-1),dfres,pfr}.  compute modsuml={'R','R-sq','MSE',hcflab,'df1','df2', 'p'}.  end if.  end if.  do if ( 2 = 2 or 2 =3).  compute xlp= llrdat.  compute ylp= y.  compute pt2 = make(nrow(ylp),1,(csum(ylp)/nrow(ylp))).  do if ( 2 =2).  compute LL3 = ylp&*ln(pt2)+(1-ylp)&*ln(1-pt2).  end if.  compute LL3 = -2*csum(LL3).  compute bt1 = make(ncol(xlp),1,0).  compute LL1 = 0.  compute pt1 = make(nrow(ylp),1,0.5).  compute pt1lp=pt1.  loop jjj = 1 to iterate.  compute vt1 = mdiag(pt1lp&*(1-pt1lp)).  compute b = bt1+inv(t(xlp)*vt1*xlp)*t(xlp)*(ylp-pt1lp).  do if ( 2 =2).  compute xlpb=xlp*b.  compute xlpbt=(xlpb > -709.7).  compute xlpb709=(1-xlpbt)*(-709.7).  compute xlpb=(xlpb&*xlpbt)+xlpb709.  compute pt1lp = 1/(1+exp(-(xlpb))).  end if.  compute itprob = csum((pt1lp < .00000001) or (pt1lp > .9999999)).  do if (itprob > 0).  loop kkk = 1 to nrow(pt1lp).  do if (pt1lp(kkk,1) > .9999999).  compute pt1lp(kkk,1) = .9999999.  end if.  do if (pt1lp(kkk,1) < .00000001).  compute pt1lp(kkk,1) = .00000001.  end if.  end loop.  compute itprob = 0.  end if.  do if (itprob = 0).  do if ( 2 =2).  compute LL = ylp&*ln(pt1lp)+(1-ylp)&*ln(1-pt1lp).  end if.  compute LL2 = -2*csum(ll).  end if.  do if (abs(LL1-LL2) < converge).  do if ( 0 =1).  compute vt1 = mdiag(pt1lp&*(1-pt1lp)).  compute varb = inv(t(xlp)*vt1*xlp).  compute seb = sqrt(diag(varb)).  end if.  break.  end if.  compute bt1 = b.  compute LL1 = LL2.  end loop.  compute modres=b.  do if (jjj > iterate).  compute itprob = 2.  do if (booting=0).  compute iterrmod=1.  end if.  do if (booting=1).  compute bootiter=1.  end if.  do if (itprobtg=0).  compute itprobtg=1.  compute errcode(errs,1) = 47.  compute errs = errs + 1.  do if (booting = 0 and 0 =1).  compute vt1 = mdiag(pt1lp&*(1-pt1lp)).  compute varb = inv(t(xlp)*vt1*xlp).  compute seb = sqrt(diag(varb)).  end if.  end if.  end if.  do if ( 0 =1).  compute trat = b&/seb.  compute dfres=nrow(xlp).  compute p = 2*(1-cdfnorm(abs(trat))).  compute modres={modres,seb,trat,p}.  compute modres={modres,(b-xp2&*seb),(b+xp2&*seb)}.  compute pvchi=1-chicdf((LL3-LL2),(nrow(modres)-1)).  compute mcF = (LL3-LL2)/LL3.  compute cox = 1-exp(-(LL3-LL2)/nrow(xlp)).  compute nagel = cox/(1-exp(-(LL3)/nrow(xlp))).  compute modsum={LL2,(LL3-LL2),(nrow(modres)-1),pvchi, mcF,cox,nagel}.  compute modsuml={'-2LL','ModelLL', 'df', 'p', 'McFadden', 'CoxSnell', 'Nagelkrk'}.  compute modresl={'coeff','se','Z','p','LLCI','ULCI'}.  end if.  end if  .  compute b=btemphld.  compute fresult={(LL2-basemod),llrdf,1-chicdf((LL2-basemod),llrdf)}  .  end if.  compute highf={highf;fresult}.  compute highf2={highf2;fresult}.  do if (j = start).  compute flabel={flabel;'X*W*Z'}.  end if.  do if (j > start).  do if (nms > 1).  compute flabel={flabel;highlbwz(jj,1)}.  else if (nms = 1).  compute flabel={flabel;'M*W*Z'}.  end if.  end if.  end if.  compute jj=jj+1.  end loop.  release jj.  compute start=start+i.  end if.  do if (nrow(highf) > 1).  compute highf=highf(2:nrow(highf),:).  compute highf2=highf2(2:nrow(highf2),:).  compute flabel=flabel(2:nrow(flabel),1).  do if ((i < nms+nys) or (ydich=0)).  compute clabtmp={'R2-chng', hcflab,'df1','df2','p'}.  print highf/format = F10.4 /rnames=flabel/cnames=clabtmp/ title = 'Test(s) of highest order unconditional interaction(s):'.  end if.  do if (ydich=1 and i=(nms+nys)).  compute clabtmp={'Chi-sq', 'df','p'}.  print/title='Likelihood ratio test(s) of highest order'.  print highf/format = F10.4 /rnames=flabel/cnames=clabtmp/ title = 'unconditional interactions(s):'/space=0.  end if.  compute intpb=highf2(:,ncol(highf2)).  end if.  compute intstart=intstart+numint(1,i).  end if.  .  do if (criterr=0).  compute threeway=0.  compute didprint=0.  compute didsome=0.  compute sigintct=0.  loop jmed =1 to (nms+1).  compute hasw=0.  compute hasz=0.  compute jnok=0.  compute nm1vls=0.  compute nm2vls=0.  compute panelgrp=0.  compute graphixs={'WITH', outnames(1,i), 'BY'}.  compute focpred4={' '}.  compute intprint=0.  compute modcat=0.  do if (jmed <= i).  do if ((jmed = 1) and ((i+1) = nrow(bcmat))).  compute pathscnt=pathscnt+1.  else.  compute paths={paths,bcmat((i+1),jmed)}.  compute pathsw={pathsw,wcmat((i+1),jmed)}.  compute pathsz={pathsz,zcmat((i+1),jmed)}.  compute pathswz={pathswz,wzcmat((i+1),jmed)}.  compute temp=fochigh(:,pathscnt)&*bootloc(:,i).  compute pathsfoc={pathsfoc,pathsfoc(:,1)}.  do if (jmed=1).  compute pathtype={pathtype,1}.  end if.  do if ((i+1)=nrow(bcmat)).  compute pathtype={pathtype,3}.  end if.  do if (jmed > 1) and ((i+1) < nrow(bcmat)).  compute pathtype={pathtype,2}.  end if.  do if (jmed=1 and nxvls > 1 and (bcmat((i+1),jmed)=1)).  compute pathsfoc(:,(pathscn2+1))=temp(2:(nxvls+1),1).  end if.  do if ((jmed > 1) or (jmed=1 and nxvls=1)).  compute temp=cmax(temp).  compute pathsfoc(1,(pathscn2+1))=temp.  end if.  compute pathscnt=pathscnt+1.  compute pathscn2=pathscn2+1.  do if (i <= nms).  compute pathsdv={pathsdv,mnames(1,i)}.  end if.  do if (i > nms).  compute pathsdv={pathsdv,ynames}.  end if.  end if.  compute coeffcol=coeffcol+1.  compute probettt=coeffs(1:nrow(b),coeffcol).  do if (jmed=1 and (bcmat((i+1),jmed)=1)).  compute omni=make(nrow(probettt),nxvls,0).  compute omnitmp=ident(nxvls).  compute omni(2:(1+nxvls),:)=omnitmp.  end if.  do if (csum(probettt)>0).  compute probvarb=make(csum(probettt),csum(probettt),999).  compute probcoef=make(csum(probettt),1,999).  compute coefflp2=1.  loop coefflp=1 to nrow(probettt).  do if (probettt(coefflp,1)=1).  compute probcoef(coefflp2,1)=b(coefflp,1).  compute coefflp2=coefflp2+1.  end if.  end loop.  compute coefflp=0.  compute coefflp2=0.  loop iclp=1 to nrow(probettt).  do if probettt(iclp,1)=1.  compute coefflp=coefflp+1.  compute coefflp2=coefflp.  compute probvarb(coefflp,coefflp) = varb(iclp,iclp).  do if (iclp < nrow(probettt)).  loop jclp=(iclp+1) to nrow(probettt).  do if (probettt(jclp,1)=1).  compute coefflp2=coefflp2+1.  compute probvarb(coefflp,coefflp2)=varb(iclp, jclp).  compute probvarb(coefflp2,coefflp)=varb(iclp, jclp).  end if.  end loop.  end if.  end if.  end loop.  end if.  end if.  compute xprobval=xmodvals.  do if (nxvls > 1 or mcx > 0).  compute xprobval=dummatx(:,2:ncol(dummatx)).  end if.  do if ((wcmat((i+1),jmed)=1) and (zcmat((i+1),jmed)=0)).  compute numplps=1.  compute modvals=wmodvals.  compute probeval=wmodvals.  compute wheremv1=wherexw.  compute nm1vls=nwvls.  compute lpstsp={1,1}.  compute modcat=0.  compute jnmod=wtmp.  compute jnmodlab=wnames.  compute jnok=1.  compute jnmin=wmin.  compute jnmax=wmax.  compute wherejn1=2.  do if (jmed=1).  compute wherejn3=wherexw(1,i).  do if (nxvls > 1).  compute jnok=0.  end if.  end if.  do if (jmed > 1).  compute wherejn1=wherem((jmed-1),i).  compute wherejn3=wheremw(((2*jmed)-3),i).  end if.  do if (nwvls > 1).  compute probeval=wprobval.  compute lpstsp(1,2)=ncol(probeval).  compute modcat=1.  compute jnok=0.  end if.  do if (wdich = 1).  compute modcat=1.  compute jnok=0.  end if.  compute problabs=wnames.  compute focpred3={wnames,'(W)'}.  compute hasw=1.  compute modgrph=wnames.  compute intprint=1.  compute sigintct=sigintct+1.  compute printpbe=intpb(sigintct,1).  end if.  do if ((wcmat((i+1),jmed)=0) and (zcmat((i+1),jmed)=1)).  compute numplps=1.  compute modvals=zmodvals.  compute probeval=zmodvals.  compute wheremv1=wherexz.  compute nm1vls=nzvls.  compute lpstsp={1,1}.  compute jnok=1.  compute jnmod=ztmp.  compute jnmin=zmin.  compute jnmax=zmax.  compute jnmodlab=znames.  compute wherejn1=2.  do if (jmed=1).  compute wherejn3=wherexz(1,i).  do if (nxvls > 1).  compute jnok=0.  end if.  end if.  do if (jmed > 1).  compute wherejn1=wherem((jmed-1),i).  compute wherejn3=wheremz(((2*jmed)-3),i).  end if.  do if (nzvls > 1).  compute probeval=zprobval.  compute lpstsp(1,2)=ncol(probeval).  compute modcat=1.  compute jnok=0.  end if.  do if (zdich = 1).  compute modcat=1.  compute jnok=0.  end if.  compute problabs=znames.  compute focpred3={znames,'(Z)'}.  compute modgrph=znames.  compute hasz=1.  compute intprint=1.  compute sigintct=sigintct+1.  compute printpbe=intpb(sigintct,1).  end if.  do if ((wzcmat((i+1),jmed)=1) or ((wcmat((i+1),jmed)=1) and (zcmat((i+1),jmed)=1))).  compute numplps=2.  compute probecnt=1.  compute intprint=1.  do if (wzcmat((i+1),jmed)=1).  compute sigintct=sigintct+1.  compute printpbe=intpb(sigintct,1).  else.  compute sigintct=sigintct+2.  compute printpbe=cmin(intpb((sigintct-1):sigintct,1)).  end if.  compute panelgrp=1.  compute hasw=1.  compute hasz=1.  compute panelcde={'/PANEL','ROWVAR=',znames,'.'}.  compute modgrph=wnames.  compute lpstsp={1,1;1,1}.  compute wheremv1=wherexw.  compute nm1vls=nwvls.  compute wheremv2=wherexz.  compute nm2vls=nzvls.  compute jnok=0.  do if (wzcmat((i+1),jmed)=1).  compute jnok=1.  end if.  do if (jmed > 1).  compute mprobval=mmodvals.  end if.  do if (jmed=1).  do if (nxvls > 1).  compute jnok=0.  end if.  end if.  do if (nwvls > 1).  compute lpstsp(1,2)=ncol(wprobval).  compute modcat=1.  compute jnok=0.  end if.  do if (zdich=1).  compute modcat=1.  compute jnok=0.  end if.  compute lpstsp(2,1)=lpstsp(1,2)+1.  compute lpstsp(2,2)=lpstsp(1,2)+1.  do if (nzvls > 1).  compute lpstsp(2,1)=lpstsp(1,2)+1.  compute lpstsp(2,2)=lpstsp(1,2)+ncol(zprobval).  compute jnok=0.  end if.  do if (zdich=1).  compute jnok=0.  end if.  compute omni3=make(nrow(b),(nxvls*nwvls),0).  do if (jmed > 1).  compute omni3=make(nrow(b),nwvls,0).  end if.  compute focpred3={wnames,'(W)'}.  compute focpred4={' ', 'Mod var:', znames, '(Z)'}.  compute modvals=make((nrow(wmodvals)*nrow(zmodvals)),2,0).  compute probeval=make((nrow(wmodvals)*nrow(zmodvals)),(ncol(wprobval)+ncol(zprobval)),0).  loop probei= 1 to nrow(wmodvals).  loop probej =1 to nrow(zmodvals).  compute modvals(probecnt,1)=wmodvals(probei,1).  compute probeval(probecnt,1:nwvls)=wprobval(probei,:).  compute modvals(probecnt,2)=zmodvals(probej,1).  compute probeval(probecnt,(nwvls+1):(nwvls+nzvls))=zprobval(probej,:).  compute probecnt=probecnt+1.  end loop.  end loop.  do if (wzcmat((i+1),jmed)=1).  compute numplps=numplps+1.  compute probprod=make(1,(ncol(wprobval)*ncol(zprobval)),0).  compute lpstsp2={1,1}.  compute lpstsp={lpstsp;lpstsp2}.  compute lpstsp(3,1)=lpstsp(2,2)+1.  compute lpstsp(3,2)=lpstsp(2,2)+ncol(probprod).  compute jnmod=ztmp.  compute jnmin=zmin.  compute jnmax=zmax.  compute jnmodlab=znames.  do if (jmed = 1).  compute wherejn1=wherexw(1,i).  compute wherejn3=wherexwz(1,i).  end if.  do if (jmed > 1).  compute wherejn1=wheremw(((2*jmed)-3),i).  compute wherejn3=wheremwz(((2*jmed)-3),i).  end if.  loop probei = 1 to nrow(wmodvals).  loop probej = 1 to nrow(zmodvals).  compute probtemp=1.  loop probek = 1 to ncol(wprobval).  compute probtemp={probtemp,(wprobval(probei,probek)&*zprobval(probej,:))}.  end loop.  compute probprod={probprod;probtemp(1,2:ncol(probtemp))}.  end loop.  end loop.  compute probprod=probprod(2:nrow(probprod),:).  compute probeval={probeval,probprod}.  end if.  compute problabs={wnames,znames}.  release probecnt, probei, probej.  end if.  do if (intprint=1).  compute focpred={' Focal', 'predict:'}.  do if (jmed=1).  compute focpred={focpred,xnames,'(X)'}.  compute focplotv=xmodvals.  end if.  do if (jmed >1).  do if (nms > 1).  compute focpred={focpred,mnames(1,(jmed-1)), medlb2(1,(jmed-1))}.  end if.  do if (nms = 1).  compute focpred={focpred,mnames(1,(jmed-1)), '(M)'}.  end if.  compute focplotv=mmodvals(:,(jmed-1)).  end if.  compute focpred2={' ', 'Mod var:',focpred3}.  compute focpred={focpred;focpred2}.  do if (ncol(focpred4) > 1).  compute focpred={focpred;focpred4}.  compute focpred4={' '}.  end if.  release focpred2,focpred3.  do if ((plot = 1 or plot = 2) or (printpbe <= intprobe)).  print focpred/title='----------'/format=A8/space=0.  end if.  compute foctmp=make(nrow(modvals),1,1).  compute probexpl=1.  compute probeva2={foctmp,probeval}.  do if (jmed=1 and nxs > 0 and mcx > 0).  compute probexpl=nxvls.  end if.  compute foctmp=make(nrow(modvals),1,1).  compute modvals3=make(1,(6+ncol(problabs)),0).  compute probrown=make(nrow(probeval),1,0).  compute jtmp=1.  loop probei = 1 to nrow(probeval).  compute probrown(probei,1)=jtmp.  compute jtmp=jtmp+nxvls.  end loop.  release jtmp.  compute probrow=999.  compute modvarl=problabs.  do if (plot = 1 or plot = 2 or nxvls > 1).  compute plotvals=make((nrow(modvals)*nrow(focplotv)),(ncol(modvals)+1),999).  loop ploti=1 to nrow(modvals).  loop plotj=1 to nrow(focplotv).  compute plotvals((((ploti-1)*nrow(focplotv))+plotj),2:ncol(plotvals))=modvals(ploti,:).  compute plotvals((((ploti-1)*nrow(focplotv))+plotj),1)=focplotv(plotj,1).  end loop.  end loop.  compute focpredn=3.  do if (jmed=1).  do if (nxvls > 1).  compute focpredn=(nxvls+1).  end if.  do if (nxvls=1 and xdich=1).  compute focpredn=2.  end if.  end if.  compute meanmat=mdiag(means).  compute onesmat=make(nrow(meanmat),(nrow(probeval)*focpredn),1).  compute probeplt=t(mdiag(means)*onesmat).  do if (jmed=1).  do if ((wcmat((i+1),1)=1) or (zcmat((i+1),1)=1)).  compute plotcnt=1.  compute iloops=nwpval*nzpval.  compute plotmx=nxpval*nzpval.  do if ((wcmat((i+1),1)=1) and (zcmat((i+1),1)=0)).  compute iloops=nwpval.  compute plotmx=nxpval.  end if.  do if ((wcmat((i+1),1)=0) and (zcmat((i+1),1)=1)).  compute iloops=nzpval.  compute plotmx=nxpval.  end if.  compute xestvals=make((nxpval*iloops),ncol(xprobval),-999).  do if (wcmat((i+1),1)=1).  compute westvals=make(nrow(xestvals),ncol(wprobval),-999).  end if.  do if (zcmat((i+1),1)=1).  compute zestvals=make(nrow(xestvals),ncol(zprobval),-999).  end if.  loop ploti=1 to iloops.  loop plotj=1 to nxpval.  compute xestvals(plotcnt,:)=xprobval(plotj,:).  compute plotcnt=plotcnt+1.  end loop.  end loop.  compute plotcnt=1.  compute plotcnt1=1.  compute plotcnt2=1.  compute plotcntz=1.  loop ploti = 1 to (iloops*nxpval).  do if (wcmat((i+1),1)=1).  compute westvals(ploti,:)=wprobval(plotcnt1,:).  end if.  do if ((wcmat((i+1),1)=0) and (zcmat((i+1),1)=1)).  compute zestvals(ploti,:)=zprobval(plotcnt1,:).  end if.  do if ((wcmat((i+1),1)=1) and (zcmat((i+1),1)=1)).  compute zestvals(ploti,:)=zprobval(plotcnt2,:).  compute plotcntz=plotcntz+1.  end if.  compute plotcnt=plotcnt+1.  do if (plotcnt > plotmx).  compute plotcnt=1.  compute plotcnt1=plotcnt1+1.  end if.  do if (plotcntz > nxpval).  compute plotcnt2=plotcnt2+1.  compute plotcntz=1.  do if (plotcnt2 > nzpval).  compute plotcnt2=1.  end if.  end if.  end loop.  compute probeplt(:,2:(1+(ncol(xestvals))))=xestvals.  do if (wcmat((i+1),1)=1).  compute probeplt(:,(wherew(1,i)):(wherew(2,i)))=westvals.  end if.  do if (zcmat((i+1),1)=1).  compute probeplt(:,(wherez(1,i)):(wherez(2,i)))=zestvals.  end if.  end if.  end if.  do if (jmed > 1).  do if ((wcmat((i+1),jmed)=1) or (zcmat((i+1),jmed)=1)).  compute plotcnt=1.  compute iloops=nwpval*nzpval.  compute plotmx=3*nzpval.  do if ((wcmat((i+1),jmed)=1) and (zcmat((i+1),jmed)=0)).  compute iloops=nwpval.  compute plotmx=3.  end if.  do if ((wcmat((i+1),jmed)=0) and (zcmat((i+1),jmed)=1)).  compute iloops=nzpval.  compute plotmx=3.  end if.  compute mestvals=make((3*iloops),1,-999).  do if (wcmat((i+1),jmed)=1).  compute westvals=make(nrow(mestvals),ncol(wprobval),-999).  end if.  do if (zcmat((i+1),jmed)=1).  compute zestvals=make(nrow(mestvals),ncol(zprobval),-999).  end if.  loop ploti=1 to iloops.  loop plotj=1 to 3.  compute mestvals(plotcnt,:)=mprobval(plotj,(jmed-1)).  compute plotcnt=plotcnt+1.  end loop.  end loop.  compute plotcnt=1.  compute plotcnt1=1.  compute plotcnt2=1.  compute plotcntz=1.  loop ploti = 1 to (iloops*3).  do if (wcmat((i+1),jmed)=1).  compute westvals(ploti,:)=wprobval(plotcnt1,:).  end if.  do if ((wcmat((i+1),jmed)=0) and (zcmat((i+1),jmed)=1)).  compute zestvals(ploti,:)=zprobval(plotcnt1,:).  end if.  do if ((wcmat((i+1),jmed)=1) and (zcmat((i+1),jmed)=1)).  compute zestvals(ploti,:)=zprobval(plotcnt2,:).  compute plotcntz=plotcntz+1.  end if.  compute plotcnt=plotcnt+1.  do if (plotcnt > plotmx).  compute plotcnt=1.  compute plotcnt1=plotcnt1+1.  end if.  do if (plotcntz > 3).  compute plotcnt2=plotcnt2+1.  compute plotcntz=1.  do if (plotcnt2 > nzpval).  compute plotcnt2=1.  end if.  end if.  end loop.  compute probeplt(:,wherem((jmed-1),i))=mestvals.  do if (wcmat((i+1),jmed)=1).  do if (model <> 74).  compute probeplt(:,(wherew(1,i)):(wherew(2,i)))=westvals.  end if.  do if (model = 74).  compute probeplt(:,(wherex(1,i)):(wherex(2,i)))=westvals.  end if.  end if.  do if (zcmat((i+1),jmed)=1).  compute probeplt(:,(wherez(1,i)):(wherez(2,i)))=zestvals.  end if.  end if.  end if.  compute prodloop = 1.  do if (jmed=1).  compute prodloop=ncol(xestvals).  end if.  do if (wcmat((i+1),jmed))=1.  compute plotcnt=0.  loop ploti = 1 to prodloop.  loop plotj = 1 to ncol(westvals).  do if (jmed=1).  compute probeplt(:,(wherexw(1,i)+plotcnt))=xestvals(:,ploti)&*westvals(:,plotj).  end if.  do if (jmed > 1).  compute probeplt(:,(wheremw(((jmed*2)-3) ,i)+plotcnt))=mestvals(:,ploti)&*westvals(:,plotj).  end if.  compute plotcnt=plotcnt+1.  end loop.  end loop.  end if.  do if (zcmat((i+1),jmed))=1.  compute plotcnt=0.  loop ploti = 1 to prodloop.  loop plotj = 1 to ncol(zestvals).  do if (jmed = 1).  compute probeplt(:,(wherexz(1,i)+plotcnt))=xestvals(:,ploti)&*zestvals(:,plotj).  end if.  do if (jmed > 1).  compute probeplt(:,(wheremz(((jmed*2)-3),i)+plotcnt))=mestvals(:,ploti)&*zestvals(:, plotj).  end if.  compute plotcnt=plotcnt+1.  end loop.  end loop.  end if.  do if (wzcmat((i+1),jmed))=1.  compute plotcnt=0.  compute threeway=1.  loop ploti = 1 to ncol(westvals).  loop plotj = 1 to ncol(zestvals).  compute probeplt(:,(wherewz(1,i)+plotcnt))=westvals(:,ploti)&*zestvals(:,plotj).  compute plotcnt=plotcnt+1.  end loop.  end loop.  compute plotcnt=0.  loop plotk = 1 to prodloop.  loop ploti = 1 to ncol(westvals).  loop plotj = 1 to ncol(zestvals).  do if (jmed = 1).  compute probeplt(:,(wherexwz(1,i)+plotcnt))=xestvals(:,plotk)&*westvals(:,ploti)&* zestvals(:,plotj).  end if.  do if (jmed > 1).  compute probeplt(:,(wheremwz(((jmed*2)-3),i)+plotcnt))=mestvals(:,plotk)&* westvals(:,ploti)&*zestvals(:,plotj).  end if.  compute plotcnt=plotcnt+1.  end loop.  end loop.  end loop.  end if.  loop newplp=1 to i.  do if (newplp <> jmed).  do if (wcmat((i+1),newplp))=1.  compute prodloop=1.  do if (newplp=1).  compute prodloop=nxvls.  end if.  compute plotcnt=0.  loop ploti = 1 to prodloop.  loop plotj = 1 to nwvls.  do if (newplp = 1).  compute probeplt(:,(wherexw(1,i)+plotcnt))=probeplt(:,(1+ploti))&*probeplt(:, (wherew(1,i)+plotj-1)).  end if.  do if (newplp > 1).  do if (model <> 74).  compute probeplt(:,(wheremw(((newplp*2)-3) ,i)+plotcnt))=probeplt(:,wherem((newplp-1),i))&*probeplt(:,(wherew(1,i)+plotj-1)).  end if.  do if (model = 74).  compute probeplt(:,(wheremw(((newplp*2)-3) ,i)+plotcnt))=probeplt(:,wherem((newplp-1),i))&*probeplt(:,(wherex(1,i)+plotj-1)).  end if.  end if.  compute plotcnt=plotcnt+1.  end loop.  end loop.  end if.  do if (zcmat((i+1),newplp))=1.  compute prodloop=1.  do if (newplp=1).  compute prodloop=nxvls.  end if.  compute plotcnt=0.  loop ploti = 1 to prodloop.  loop plotj = 1 to nzvls.  do if (newplp = 1).  compute probeplt(:,(wherexz(1,i)+plotcnt))=probeplt(:,(1+ploti))&*probeplt(:, (wherez(1,i)+plotj-1)).  end if.  do if (newplp > 1).  compute probeplt(:,(wheremz(((newplp*2)-3),i)+plotcnt))=probeplt(:, wherem((newplp-1),i))&*probeplt(:,(wherez(1,i)+plotj-1)).  end if.  compute plotcnt=plotcnt+1.  end loop.  end loop.  end if.  do if (wzcmat((i+1),newplp))=1.  compute plotcnt=0.  do if (threeway=0).  loop ploti = 1 to nwvls.  loop plotj = 1 to nzvls.  compute probeplt(:,(wherewz(1,i)+plotcnt))=probeplt(:,(wherew(1,i)+ploti-1))&* probeplt(:,(wherez(1,i)+plotj-1)).  compute plotcnt=plotcnt+1.  end loop.  end loop.  end if.  compute prodloop=1.  do if (newplp=1).  compute prodloop=nxvls.  end if.  compute plotcnt=0.  loop plotk = 1 to prodloop.  loop ploti = 1 to nwvls.  loop plotj = 1 to nzvls.  do if (newplp = 1).  compute probeplt(:,(wherexwz(1,i)+plotcnt))=probeplt(:,(1+plotk))&*probeplt(:, (wherew(1,i)+ploti-1))&*probeplt(:,(wherez(1,i)+plotj-1)).  end if.  do if (newplp > 1).  compute probeplt(:,(wheremwz(((newplp*2)-3),i)+plotcnt))=probeplt(:, wherem((newplp-1),i))&*probeplt(:,(wherew(1,i)+ploti-1))&*probeplt(:,(wherez(1,i)+plotj-1)).  end if.  compute plotcnt=plotcnt+1.  end loop.  end loop.  end loop.  end if.  end if.  end loop.  compute predvals=probeplt*b.  do if (debug <> 0).  print probeplt.  end if.  do if (i = nms+nys) and (ydich=1).  compute predvalt=(predvals < 709.7).  compute prevalt7=(1-predvalt)*(709.7).  compute predvals=(predvals&*predvalt)+prevalt7.  compute expyhat=exp(predvals)&/(1+exp(predvals)).  end if.  compute sepred=make(nrow(plotvals),3,999).  loop sei=1 to nrow(plotvals).  compute ask=probeplt(sei,:).  compute sepred(sei,1)=sqrt(ask*varb*t(ask)).  do if ((i < nms+nys) or (ydich=0)).  compute sepred(sei,2)=predvals(sei,1)-tval*sepred(sei,1).  compute sepred(sei,3)=predvals(sei,1)+tval*sepred(sei,1).  end if.  do if ((i = nms+nys) and (ydich=1)).  compute sepred(sei,2)=predvals(sei,1)-xp2*sepred(sei,1).  compute sepred(sei,3)=predvals(sei,1)+xp2*sepred(sei,1).  end if.  end loop.  compute prevloc=ncol(plotvals)+1.  compute probeplt={plotvals,predvals}.  do if (plot = 2).  compute probeplt={probeplt,sepred}.  end if.  do if ((i = nms+nys) and (ydich=1)).  compute probeplt={probeplt,expyhat}.  end if.  compute didsome=0.  end if.  do if ((wzcmat((i+1),jmed)=1) and (printpbe <= intprobe)).  do if (jmed=1).  compute omnilp2=nxvls*nwvls.  compute omnitmp=ident(omnilp2).  compute omni3(wherexw(1,i):wherexw(2,i),:)=omnitmp.  end if.  do if (jmed>1).  compute omnilp2=nwvls.  compute omnitmp=ident(omnilp2).  compute omni3(wheremw(((jmed*2)-3),i):wheremw(((jmed*2)-2),i),:)=omnitmp.  end if.  compute omnif=make(1,4,0).  do if ((i = nms+nys) and (ydich=1)).  compute omnif=make(1,3,0).  end if.  compute condeff3=0.  loop omnilp1=1 to nrow(zprobval).  loop omnilp=1 to (omnilp2).  do if (jmed=1).  compute omni3((wherexwz(1,i)+((omnilp-1)*nzvls)):(wherexwz(1,i)+((omnilp-1)*nzvls)+ (nzvls-1)),omnilp)=t(zprobval(omnilp1,:)).  end if.  do if (jmed > 1).  compute omni3((wheremwz(((jmed*2)-3),i)+((omnilp-1)*nzvls)):(wheremwz(((jmed*2)-3),i)+ ((omnilp-1)*nzvls)+(nzvls-1)),omnilp)=t(zprobval(omnilp1,:)).  end if.  end loop.  compute condeff=t(omni3)*b.  compute condeff3={condeff3;condeff}.  .  compute lmat2= omni3.  do if ( 1 =0).  compute lmat2 = mdiag( omni3 ).  compute lmat3=make(nrow(lmat2),1,0).  loop flp=1 to ncol(lmat2).  do if (csum(lmat2(:,flp))=1).  compute lmat3={lmat3,lmat2(:,flp)}.  end if.  end loop.  compute lmat2=lmat3(:,2:ncol(lmat3)).  end if.  compute fratio = (t(t(lmat2)* b )*inv(t(lmat2)* varb *lmat2)*((t(lmat2)* b )))/ncol(lmat2).  compute pfr = 1-fcdf(fratio,ncol(lmat2),(n-nrow( b ))).  compute fresult={fratio,ncol(lmat2),(n-nrow( b )),pfr}.  do if (i = (nms+nys) and (ydich=1)).  compute fratio=fratio*ncol(lmat2).  compute pfr=1-chicdf(fratio,ncol(lmat2)).  compute fresult={fratio,ncol(lmat2),pfr}.  end if.  do if ( 0 =1).  compute lmat3=1-rsum(lmat2).  compute xfm=make(n,csum(lmat3),0).  compute flpc=1.  loop flp=1 to nrow(lmat3).  do if (lmat3(flp,1)=1).  compute xfm(:,flpc)=x(:,flp).  compute flpc=flpc+1.  end if.  end loop.  compute bfm=inv(t(xfm)*xfm)*t(xfm)*y.  compute resid=y-(xfm*bfm).  compute sstotal=(y-(csum(y)/n)).  compute sstotal=csum(sstotal&*sstotal).  compute ssresid=csum(resid&*resid).  compute rsqch= 0 -((sstotal-ssresid)/sstotal).  compute fresult={rsqch,fresult}.  release xfm,flpc, resid, ssresid, bfm.  end if  .  compute omnif={omnif;fresult}.  end loop.  compute omnif=omnif(2:nrow(omnif),:).  compute clabtmp=znames.  compute condeff3=condeff3(2:nrow(condeff3),:).  do if ((nxvls*nwvls)=1).  compute omnif={condeff3,omnif}.  compute clabtmp={clabtmp,'Effect'}.  end if.  compute omnif={zmodvals,omnif}.  do if ((i < nms+nys) or (ydich=0)).  compute clabtmp={clabtmp,hcflab,'df1','df2','p'}.  end if.  do if ((i = (nms+nys)) and (ydich=1)).  compute clabtmp={clabtmp,'Chi-sq','df','p'}.  end if.  do if (jmed=1).  print omnif/title='Test of conditional X*W interaction at value(s) of Z:'/cnames=clabtmp/format= F10.4.  end if.  do if (jmed>1).  print omnif/title='Test of conditional M*W interaction at value(s) of Z:'/cnames=clabtmp/format= F10.4.  end if.  release omni3.  end if.  loop probei = 1 to probexpl.  do if (probexpl > 1).  compute foctmp=make(nrow(modvals),probexpl,0).  compute foctmp(:,probei)=foctmp(:,probei)+1.  compute probtemp=make(nrow(modvals),1,0).  loop probem = 1 to numplps.  loop probek = 1 to nxvls.  loop probej=lpstsp(probem,1) to lpstsp(probem,2).  compute probtemp={probtemp,foctmp(:,probek)&*probeval(:,probej)}.  end loop.  end loop.  end loop.  compute probeva2=probtemp(:,2:ncol(probtemp)).  compute probeva2={foctmp,probeva2}.  end if.  compute probres=probeva2*probcoef.  compute probrese=sqrt(diag(probeva2*probvarb*t(probeva2))).  compute tratio = probres&/probrese.  compute p = 2*(1-tcdf(abs(tratio), dfres)).  do if (ydich=1 and i = (nms+nys)).  compute p = 2*(1-cdfnorm(abs(tratio))).  end if.  compute modvals2={modvals,probres,probrese,tratio, p}.  do if ((i < nms+nys) or (ydich=0)).  compute modvals2={modvals2,(probres-tval&*probrese),(probres+tval&*probrese)}.  compute problabs={problabs,'Effect',hclab,'t', 'p', 'LLCI', 'ULCI'}.  end if.  do if (ydich=1 and i = (nms+nys)).  compute modvals2={modvals2,(probres-xp2&*probrese),(probres+xp2&*probrese)}.  compute problabs={problabs,'Effect','se','Z', 'p', 'LLCI', 'ULCI'}.  end if.  do if (probexpl > 1 and (printpbe <= intprobe)).  do if (hasz = 1).  compute printz=1.  end if.  do if (hasw=1).  compute printw=1.  end if.  compute probrlab=make(nrow(modvals),1,xcatlab(probei,1)).  compute modvals3={modvals3; modvals2}.  compute probrow={probrow;probrown}.  compute probrown=probrown+1.  do if (probei=probexpl).  compute xproblab=xcatlab(1:nxvls,1).  compute probrow=probrow(2:nrow(probrow),1).  compute modvals3=modvals3(2:nrow(modvals3),:).  compute temp=modvals3.  compute temp(GRADE(probrow(:,1)),:)=modvals3.  compute modvals3=temp.  compute start2=1.  compute problabs=problabs(1,(1+(ncol(modvarl))):ncol(problabs)).  compute pstart=1.  loop probek= 1 to nrow(probeval).  compute endstart=start2+(nxvls-1).  compute temp=modvals3(start2:endstart,(1+ncol(modvarl)):ncol(modvals3)).  compute temp2=t(modvals3(start2:start2,1:ncol(modvarl))).  compute trnames=t(modvarl).  do if (probek > 1).  print/title='----------'/space=0.  else.  print/title = 'Conditional effects of the focal predictor at values of the moderator(s):'.  do if ((jmed=1) and (i = (nms+nys)) and (nms > 0)).  do if (nxvls = 1).  print/title = '(These are also the conditional direct effects of X on Y)'/space=0.  else.  print/title = '(These are also the relative conditional direct effects of X on Y)'/space=0.  end if.  end if.  print.  end if.  print temp2/title = 'Moderator value(s):'/rnames=trnames/format= F10.4 /space=0.  print temp/title = ' '/cnames=problabs/rnames=xproblab/format= F10.4 /space=0.  compute start2=start2+nxvls.  compute didsome=1.  do if (jmed=1).  compute mod1val=probeval(probek,1:nm1vls).  loop omnilp=1 to nxvls.  compute omni((wheremv1(1,i)+((omnilp-1)*nm1vls)):(wheremv1(1,i)+((omnilp-1)* nm1vls)+(nm1vls-1)),omnilp)=t(mod1val).  do if (nm1vls < ncol(probeval)).  compute mod2val=probeval(probek,(nm1vls+1):(nm1vls+nm2vls)).  compute omni((wheremv2(1,i)+((omnilp-1)*nm2vls)):(wheremv2(1,i)+((omnilp-1)* nm2vls)+(nm2vls-1)),omnilp)=t(mod2val).  do if ((nm1vls+nm2vls) < ncol(probeval)).  compute intlen=nm1vls*nm2vls.  compute modintvl=probeval(probek,(nm1vls+nm2vls+1):ncol(probeval)).  compute omni((wherexwz(1,i)+((omnilp-1)*intlen)):(wherexwz(1,i)+((omnilp-1)* intlen)+(intlen-1)),omnilp)=t(modintvl).  end if.  end if.  end loop.  .  compute lmat2= omni.  do if ( 1 =0).  compute lmat2 = mdiag( omni ).  compute lmat3=make(nrow(lmat2),1,0).  loop flp=1 to ncol(lmat2).  do if (csum(lmat2(:,flp))=1).  compute lmat3={lmat3,lmat2(:,flp)}.  end if.  end loop.  compute lmat2=lmat3(:,2:ncol(lmat3)).  end if.  compute fratio = (t(t(lmat2)* b )*inv(t(lmat2)* varb *lmat2)*((t(lmat2)* b )))/ncol(lmat2).  compute pfr = 1-fcdf(fratio,ncol(lmat2),(n-nrow( b ))).  compute fresult={fratio,ncol(lmat2),(n-nrow( b )),pfr}.  do if (i = (nms+nys) and (ydich=1)).  compute fratio=fratio*ncol(lmat2).  compute pfr=1-chicdf(fratio,ncol(lmat2)).  compute fresult={fratio,ncol(lmat2),pfr}.  end if.  do if ( 0 =1).  compute lmat3=1-rsum(lmat2).  compute xfm=make(n,csum(lmat3),0).  compute flpc=1.  loop flp=1 to nrow(lmat3).  do if (lmat3(flp,1)=1).  compute xfm(:,flpc)=x(:,flp).  compute flpc=flpc+1.  end if.  end loop.  compute bfm=inv(t(xfm)*xfm)*t(xfm)*y.  compute resid=y-(xfm*bfm).  compute sstotal=(y-(csum(y)/n)).  compute sstotal=csum(sstotal&*sstotal).  compute ssresid=csum(resid&*resid).  compute rsqch= 0 -((sstotal-ssresid)/sstotal).  compute fresult={rsqch,fresult}.  release xfm,flpc, resid, ssresid, bfm.  end if  .  do if (i < (nms + nys) or (ydich = 0)).  compute clabtmp={hcflab,'df1','df2','p'}.  print fresult/title='Test of equality of conditional means'/cnames=clabtmp/format= F10.4.  end if.  do if (i = (nms + nys) and (ydich = 1)).  compute clabtmp={'Chi-sq','df','p'}.  print fresult/title='Test of equality of conditional logits or probabilities'/cnames=clabtmp/format= F10.4.  end if.  compute probetmp=probeplt(pstart:(pstart+nxvls),1).  compute probetmp={probetmp,probeplt(pstart:(pstart+nxvls),prevloc:ncol(probeplt))}.  compute pstart=pstart+(nxvls+1).  do if (i < ((nms + nys)) or (ydich = 0)).  compute clabtmp={xnames, outnames(1,i), hclab, 'LLCI', 'ULCI'}.  print probetmp/title = 'Estimated conditional means being compared:'/cnames=clabtmp/format= F10.4.  end if.  do if ((i = (nms+nys)) and (ydich = 1)).  compute clabtmp={xnames, outnames(1,i), 'prob'}.  compute probetm2=probetmp(:,1:2).  compute probetm2={probetm2,probetmp(:,ncol(probetmp))}.  print probetm2/title = 'Estimated conditional logits and probabilities:'/cnames=clabtmp/format= F10.4.  end if.  end if.  end loop.  release probrow, start2, endstart, temp, temp2.  end if.  end if.  do if (probexpl = 1 and (printpbe <= intprobe)).  print/title = 'Conditional effects of the focal predictor at values of the moderator(s):'.  print modvals2/cnames=problabs/title = ' '/space=0/format= F10.4.  compute didsome=1.  do if (hasz = 1).  compute printz=1.  end if.  do if (hasw=1).  compute printw=1.  end if.  do if (jn = 1 and jnok=1).  do if (criterr = 0).  compute dfres=n-nrow(b).  compute jncrit =(dfres* (exp((dfres-(5/6))*((xp2/(dfres-(2/3)+(.11/dfres)))* (xp2/(dfres-(2/3)+(.11/dfres)))))-1)).  do if (i = (nms+nys) and (ydich=1)).  compute jncrit=xp2*xp2.  end if.  compute jnb1=b(wherejn1,1).  compute jnb3=b(wherejn3,1).  compute jnsb1=varb(wherejn1,wherejn1).  compute jnsb3=varb(wherejn3,wherejn3).  compute jnsb1b3=varb(wherejn1,wherejn3).  compute ajn =(jncrit*jnsb3)-(jnb3*jnb3).  compute bjn = 2*((jncrit*jnsb1b3)-(jnb1*jnb3)).  compute cjn = (jncrit*jnsb1)-(jnb1*jnb1).  compute radarg = (bjn*bjn)-(4*ajn*cjn).  compute den = 2*ajn.  compute nrts = 0.  do if (radarg >= 0 and den <> 0).  compute x21 = (-bjn+sqrt(radarg))/den.  compute x22 = (-bjn-sqrt(radarg))/den.  compute roots = 0.  do if (x21 >= jnmin and x21 <= jnmax).  compute nrts = 1.  compute roots = {roots; x21}.  end if.  do if (x22 >= jnmin and x22 <= jnmax).  compute nrts = nrts + 1.  compute roots = {roots; x22}.  end if.  compute roots={roots,make(nrow(roots),2,0)}.  end if.  do if (nrts > 0).  compute roots = roots(2:nrow(roots),1:3).  compute roots(1,2)=(csum(jnmod < roots(1,1))/n)*100.  compute roots(1,3)=(csum(jnmod > roots(1,1))/n)*100.  do if (nrow(roots)=2).  compute roots(2,2)=(csum(jnmod < roots(2,1))/n)*100.  compute roots(2,3)=(csum(jnmod > roots(2,1))/n)*100.  end if.  print roots/title = 'Moderator value(s) defining Johnson-Neyman significance region(s):'/clabels = 'Value', '% below', '% above'/format F10.4.  end if.  do if (nrts = 0).  print/title = 'There are no statistical significance transition points within the observed'.  print/title = 'range of the moderator found using the Johnson-Neyman method.'/space=0.  end if.  compute jnvals=make((21+nrts),7,0).  loop jni= 0 to 20.  compute jnvals((jni+1),1)=jnmin+(jni*((jnmax-jnmin)/20)).  end loop.  do if (nrts > 0).  loop jni = 1 to nrts.  loop jnj = 2 to nrow(jnvals).  do if ((roots(jni,1) > jnvals((jnj-1),1)) and (roots(jni,1) < jnvals(jnj,1))).  compute jnvals((jnj+1):(21+jni),1)=jnvals(jnj:(20+jni),1).  compute jnvals(jnj,1)=roots(jni,1).  end if.  end loop.  end loop.  end if.  loop jni = 1 to nrow(jnvals).  compute jnvals(jni,2)=jnb1+jnb3*jnvals(jni,1).  compute jnvals(jni,3)=sqrt(jnsb1+2*jnvals(jni,1)*jnsb1b3+(jnvals(jni,1)*jnvals(jni, 1))*jnsb3).  compute jnvals(jni,4)=jnvals(jni,2)/jnvals(jni,3).  compute jnvals(jni,5)=2*(1-tcdf(abs(jnvals(jni,4)), dfres)).  compute jnvals(jni,6)=jnvals(jni,2)-sqrt(jncrit)*jnvals(jni,3).  compute jnvals(jni,7)=jnvals(jni,2)+sqrt(jncrit)*jnvals(jni,3).  do if ((i = nms + nys) and (ydich=1)).  compute jnvals(jni,5)=2*(1-cdfnorm(abs(jnvals(jni,4)))).  compute jnvals(jni,6)=jnvals(jni,2)-xp2*jnvals(jni,3).  compute jnvals(jni,7)=jnvals(jni,2)+xp2*jnvals(jni,3).  end if.  end loop.  do if ((i < nms+nys) or (ydich=0)).  compute jnclbs={jnmodlab,'Effect',hclab,'t', 'p', 'LLCI', 'ULCI'}.  end if.  do if ((i = nms + nys) and (ydich=1)).  compute jnclbs={jnmodlab,'Effect','se','Z', 'p', 'LLCI', 'ULCI'}.  end if.  do if (((wcmat((i+1),jmed)=1) or (zcmat((i+1),jmed)=1)) and (wzcmat((i+1),jmed)=0)).  print jnvals/title = 'Conditional effect of focal predictor at values of the moderator:'/cnames =jnclbs/format = F10.4.  end if.  do if ((jmed = 1) and (wzcmat((i+1),jmed)=1)).  print jnvals/title = 'Conditional X*W interaction at values of the moderator Z:'/cnames =jnclbs/format = F10.4.  end if.  do if ((jmed > 1) and (wzcmat((i+1),jmed)=1)).  print jnvals/title = 'Conditional M*W interaction at values of the moderator Z:'/cnames =jnclbs/format = F10.4.  end if.  end if.  end if.  end if.  do if ((i = (nms+nys)) and (jmed=1) and (bcmat(nrow(bcmat),1)=1)).  do if (probei=1).  compute direfflb=problabs.  compute direff=modvals2.  end if.  do if (probei>1).  compute direff={direff;modvals2}.  end if.  end if.  compute intprint=0.  do if ((jmed=1) and (i=1) and nms=0) and modcok=1).  compute contvec2=make(2,1,1).  compute contvec2={contvec2,wcontval,zcontval}.  do if (wzcmat((i+1),jmed)=1).  loop conti= 1 to ncol(wcontval).  loop contj = 1 to ncol(zcontval).  compute contvec2={contvec2,wcontval(:,conti)&*zcontval(:,contj)}.  end loop.  end loop.  end if.  compute conteff=contvec2*probcoef.  compute contdiff=contvec2(1,:)-contvec2(2,:).  compute contse=sqrt(contdiff*probvarb*t(contdiff)).  compute conteffd=conteff(1,1)-conteff(2,1).  compute contvec={contvec,conteff}.  print/title='Contrast between conditional effects of X:'.  print contvec/title=' '/rlabels='Effect1:','Effect2:'/cnames=problabs/format = F10.4 /space=0.  do if (ydich=0).  compute p=2*(1-tcdf(abs(conteffd/contse), dfres)).  compute contvec={conteffd,contse,conteffd/contse, p}.  compute contvec={contvec,(conteffd-(tval*contse))}.  compute contvec={contvec,(conteffd+(tval*contse))}.  compute contlabs={'Contrast', hclab, 't', 'p', 'LLCI', 'ULCI'}.  end if.  do if (ydich=1).  compute p=2*(1-cdfnorm(abs(conteffd/contse))).  compute contvec={conteffd,contse,conteffd/contse, p}.  compute contvec={contvec,(conteffd-(xp2*contse))}.  compute contvec={contvec,(conteffd+(xp2*contse))}.  compute contlabs={'Contrast', 'se', 'Z', 'p', 'LLCI', 'ULCI'}.  end if.  print contvec/title='Test of Effect1 minus Effect2'/format= F10.4 /cnames=contlabs.  end if.  end loop.  do if (plot = 1 or plot = 2).  compute datalabs={t(focpred(:,3)),outnames(1,i)}.  do if (plot = 1).  compute datalabs={datalabs}.  end if.  do if (plot = 2).  compute datalabs={datalabs,'se', 'LLCI', 'ULCI'}.  end if.  do if ((i = nms+nys) and (ydich=1)).  compute datalabs={datalabs,'prob'}.  end if.  compute datalabs={datalabs,'.'}.  print/title = 'Data for visualizing the conditional effect of the focal predictor:'.  print/title = 'Paste text below into a SPSS syntax window and execute to produce plot.'/space=0.  compute dumb = {' ', ' ', ' ', ' ', ' ', ' ', ' '}.  print datalabs/title = 'DATA LIST FREE/'/format=A10.  print probeplt/title = 'BEGIN DATA.'/format.= F10.4 /space=0.  print/title = 'END DATA.'/space=0.  compute focgrph=datalabs(1,1).  compute graphix={focgrph,graphixs,modgrph}.  do if (((xdich=1) or (nxvls > 1)) and ((modcat=0) and (focgrph = xnames))).  compute graphix={modgrph,graphixs,focgrph}.  end if.  do if (panelgrp = 0).  compute graphix={graphix,'.'}.  else.  compute graphix={graphix,panelcde}.  end if.  print graphix/title = 'GRAPH/SCATTERPLOT='/format=A8/space=0.  do if (i = (nms + nys) and ydich = 1).  compute graphixd=graphix.  compute graphixd(1,3)='prob'.  print graphixd/title = 'GRAPH/SCATTERPLOT='/format=A8/space=0.  end if.  end if.  end if.  end loop.  release jmed, intprint,didprint.  end if  .  do if (model = 74 and i <= nms).  print/title='Here are estimates of M'.  compute onetemp=make(nrow(xprobval),1,1).  compute mestmt74={onetemp,xprobval}.  do if (ncs > 0).  compute ncovmdl=rsum(ccmat(i,:)).  do if (ncovmdl > 0).  compute cvmnc=make(nrow(mestmt74),ncovmdl,1).  compute cvmnctmp=csum(x(:,(ncol(x)-ncovmdl+1):ncol(x)))/nrow(x).  loop mestlp=1 to ncovmdl.  compute cvmnc(:,mestlp)=cvmnc(:,mestlp)*cvmnctmp(:,mestlp).  end loop.  compute mestmt74={mestmt74,cvmnc}.  end if.  end if.  print mestmt74.  compute mest74t=mestmt74*b.  do if (i = 1).  compute mest74=mest74t.  end if.  do if (i > 1).  compute mest74={mest74,mest74t}.  end if.  print mest74.  end if.  end loop if criterr=1.  do if (criterr=0 and dototal = 1).  print/title = '************************** TOTAL EFFECT MODEL ****************************'.  print outnames(1,ncol(outnames))/title = 'OUTCOME VARIABLE:'/format = A8/space=0.  compute x=xtmp.  compute vlabsm={'constant';xcatlab(1:nxvls,1)}.  do if (ncs > 0).  compute x = {x,ctmp}.  compute vlabsm={vlabsm;t(covnames)}.  end if.  compute x = {ones,x}.  .  do if ( 1 =1).  compute b = inv(t( x )* x )*t( x )* y.  compute modres=b.  do if ( 1 =1).  compute n1=nrow( x ).  compute dfres=n1-(ncol( x )).  compute sstotal = t( y -(csum( y )/n1))*( y -(csum( y )/n1)).  compute resid= y - x *b.  compute ssresid = csum((resid)&**2).  compute r2 = (sstotal-ssresid)/sstotal.  compute adjr2 = 1-((1-r2)*(n1-1)/(dfres)).  compute mse=ssresid/(n1-ncol( x )).  .  compute n1=nrow( x ).  compute invXtX = inv(t( x )* x ).  compute varb = mse *invXtX.  compute k3 = ncol( x ).  compute xhc=0.  do if ( hc <> 5).  compute xhc= x.  compute hat = xhc(:,1).  loop i3=1 to nrow(xhc).  compute hat(i3,1)= xhc(i3,:)*invXtX*t(xhc(i3,:)).  end loop.  do if ( hc = 0 or hc =1).  loop i3 = 1 to k3.  compute xhc(:,i3)=xhc(:,i3)&* resid.  end loop.  end if.  do if ( hc =3 or hc =2).  loop i3=1 to k3.  compute xhc(:,i3) = ( resid &/(1-hat)&**(1/(4hc )))&*xhc(:,i3).  end loop.  end if.  do if ( hc = 4).  compute hcmn=make(n,2,4).  compute hcmn(:,2)=(n1*hat)/k3.  loop i3= 1 to k3.  compute xhc(:,i3) = ( resid &/(1-hat)&**(rmin(hcmn)/2))&*xhc(:,i3).  end loop.  end if.  compute varb=(invXtX*t(xhc)*xhc*invXtX).  do if ( hc =1).  compute varb=(n1/(n1-ncol( x )))&*varb.  end if.  end if.  compute hclab={'se(HC0)','se(HC1)','se(HC2)','se(HC3)','se(HC4)','se'}.  compute hclab=hclab(1,( hc +1)).  compute hcflab={'F(HC0)','F(HC1)','F(HC2)','F(HC3)','F(HC4)','F'}.  compute hcflab=hcflab(1,( hc +1)).  release xhc  .  compute seb=sqrt(diag(varb)).  compute trat = b&/seb.  compute p = 2*(1-tcdf(abs(trat), (dfres))).  compute tval = sqrt(dfres* (exp((dfres-(5/6))*((xp2/(dfres-(2/3)+(.11/dfres)))* (xp2/(dfres-(2/3)+(.11/dfres)))))-1)).  compute modres={modres,seb,trat,p}.  compute modres={modres,(b-tval&*seb),(b+tval&*seb)}.  compute modresl={'coeff',hclab,'t','p','LLCI','ULCI'}.  compute lmat = ident(ncol( x )).  compute lmat = lmat(:,2:ncol(lmat)).  compute fratio = (t(t(lmat)*b)*inv(t(lmat)*varb*lmat)*((t(lmat)*b)))/(ncol( x )-1).  compute pfr = 1-fcdf(fratio,(ncol( x )-1),dfres).  compute modsum={sqrt(r2),r2,mse,fratio,(ncol( x )-1),dfres,pfr}.  compute modsuml={'R','R-sq','MSE',hcflab,'df1','df2', 'p'}.  end if.  end if.  do if ( 1 = 2 or 1 =3).  compute xlp= x.  compute ylp= y.  compute pt2 = make(nrow(ylp),1,(csum(ylp)/nrow(ylp))).  do if ( 1 =2).  compute LL3 = ylp&*ln(pt2)+(1-ylp)&*ln(1-pt2).  end if.  compute LL3 = -2*csum(LL3).  compute bt1 = make(ncol(xlp),1,0).  compute LL1 = 0.  compute pt1 = make(nrow(ylp),1,0.5).  compute pt1lp=pt1.  loop jjj = 1 to iterate.  compute vt1 = mdiag(pt1lp&*(1-pt1lp)).  compute b = bt1+inv(t(xlp)*vt1*xlp)*t(xlp)*(ylp-pt1lp).  do if ( 1 =2).  compute xlpb=xlp*b.  compute xlpbt=(xlpb > -709.7).  compute xlpb709=(1-xlpbt)*(-709.7).  compute xlpb=(xlpb&*xlpbt)+xlpb709.  compute pt1lp = 1/(1+exp(-(xlpb))).  end if.  compute itprob = csum((pt1lp < .00000001) or (pt1lp > .9999999)).  do if (itprob > 0).  loop kkk = 1 to nrow(pt1lp).  do if (pt1lp(kkk,1) > .9999999).  compute pt1lp(kkk,1) = .9999999.  end if.  do if (pt1lp(kkk,1) < .00000001).  compute pt1lp(kkk,1) = .00000001.  end if.  end loop.  compute itprob = 0.  end if.  do if (itprob = 0).  do if ( 1 =2).  compute LL = ylp&*ln(pt1lp)+(1-ylp)&*ln(1-pt1lp).  end if.  compute LL2 = -2*csum(ll).  end if.  do if (abs(LL1-LL2) < converge).  do if ( 1 =1).  compute vt1 = mdiag(pt1lp&*(1-pt1lp)).  compute varb = inv(t(xlp)*vt1*xlp).  compute seb = sqrt(diag(varb)).  end if.  break.  end if.  compute bt1 = b.  compute LL1 = LL2.  end loop.  compute modres=b.  do if (jjj > iterate).  compute itprob = 2.  do if (booting=0).  compute iterrmod=1.  end if.  do if (booting=1).  compute bootiter=1.  end if.  do if (itprobtg=0).  compute itprobtg=1.  compute errcode(errs,1) = 47.  compute errs = errs + 1.  do if (booting = 0 and 1 =1).  compute vt1 = mdiag(pt1lp&*(1-pt1lp)).  compute varb = inv(t(xlp)*vt1*xlp).  compute seb = sqrt(diag(varb)).  end if.  end if.  end if.  do if ( 1 =1).  compute trat = b&/seb.  compute dfres=nrow(xlp).  compute p = 2*(1-cdfnorm(abs(trat))).  compute modres={modres,seb,trat,p}.  compute modres={modres,(b-xp2&*seb),(b+xp2&*seb)}.  compute pvchi=1-chicdf((LL3-LL2),(nrow(modres)-1)).  compute mcF = (LL3-LL2)/LL3.  compute cox = 1-exp(-(LL3-LL2)/nrow(xlp)).  compute nagel = cox/(1-exp(-(LL3)/nrow(xlp))).  compute modsum={LL2,(LL3-LL2),(nrow(modres)-1),pvchi, mcF,cox,nagel}.  compute modsuml={'-2LL','ModelLL', 'df', 'p', 'McFadden', 'CoxSnell', 'Nagelkrk'}.  compute modresl={'coeff','se','Z','p','LLCI','ULCI'}.  end if.  end if  .  print modsum/title = 'Model Summary'/cnames = modsuml/format= F10.4.  print modres/title='Model'/rnames=vlabsm/cnames=modresl/format= F10.4.  compute toteff=modres(2:(1+nxvls),:).  compute totefflb=modresl.  compute toteffl2=vlabsm(2:(1+nxvls),:).  compute lmat=make(nrow(b),1,0).  compute lmat2=make(nxvls,1,1).  compute lmat(2:(1+nxvls),1)=lmat2.  do if (ydich <> 1).  .  compute lmat2= lmat.  do if ( 0 =0).  compute lmat2 = mdiag( lmat ).  compute lmat3=make(nrow(lmat2),1,0).  loop flp=1 to ncol(lmat2).  do if (csum(lmat2(:,flp))=1).  compute lmat3={lmat3,lmat2(:,flp)}.  end if.  end loop.  compute lmat2=lmat3(:,2:ncol(lmat3)).  end if.  compute fratio = (t(t(lmat2)* b )*inv(t(lmat2)* varb *lmat2)*((t(lmat2)* b )))/ncol(lmat2).  compute pfr = 1-fcdf(fratio,ncol(lmat2),(n-nrow( b ))).  compute fresult={fratio,ncol(lmat2),(n-nrow( b )),pfr}.  do if (i = (nms+nys) and (ydich=1)).  compute fratio=fratio*ncol(lmat2).  compute pfr=1-chicdf(fratio,ncol(lmat2)).  compute fresult={fratio,ncol(lmat2),pfr}.  end if.  do if ( 1 =1).  compute lmat3=1-rsum(lmat2).  compute xfm=make(n,csum(lmat3),0).  compute flpc=1.  loop flp=1 to nrow(lmat3).  do if (lmat3(flp,1)=1).  compute xfm(:,flpc)=x(:,flp).  compute flpc=flpc+1.  end if.  end loop.  compute bfm=inv(t(xfm)*xfm)*t(xfm)*y.  compute resid=y-(xfm*bfm).  compute sstotal=(y-(csum(y)/n)).  compute sstotal=csum(sstotal&*sstotal).  compute ssresid=csum(resid&*resid).  compute rsqch= r2 -((sstotal-ssresid)/sstotal).  compute fresult={rsqch,fresult}.  release xfm,flpc, resid, ssresid, bfm.  end if  .  compute totomni=fresult.  end if.  do if (stand=1).  compute predsd=make(nrow(modres),1,0).  compute stdmod=modres(:,1)&/ovsd(1,ncol(ovsd)).  loop jd=1 to ncol(x).  compute descdat=x(:,jd).  compute predsd(jd,1) = (nrow(descdat)*sscp(descdat))-(t(csum(descdat))*(csum(descdat))).  compute predsd(jd,1) = sqrt(predsd(jd,1)/(nrow(descdat)*(nrow(descdat)-1))).  end loop.  do if (wherex(1,ncol(wherex)) <> -999 and ((nxvls > 1) or (xdich=1))).  compute sdmsone=make(nxvls,1,1).  compute predsd(wherex(1,ncol(wherex)):wherex(2,ncol(wherex)),1)=sdmsone.  compute pstog=1.  end if.  compute predsd(1,1)=1.  compute stdmod=stdmod&*predsd.  compute stdmod=stdmod(2:nrow(stdmod),1).  compute sdvlabs=vlabsm(2:nrow(vlabsm),1).  print stdmod/title='Standardized coefficients'/clabels='coeff'/rnames=sdvlabs/format= F10.4.  end if.  do if (covcoeff=1).  print varb/title='Covariance matrix of regression parameter estimates:'/rnames=vlabsm /cnames=vlabsm/format= F10.4.  end if.  end if.  end if.  do if (criterr=0 and boot > 0).  compute bootres=make(1,rsum(nump),-999).  do if (effsize=1).  compute bootysd=make(1,1,-999).  compute bootxsd=make(1,1,-999).  end if.  compute badboot=0.  compute goodboot=0.  compute smallest=1.  compute booting=1.  loop j = 1 to maxboot.  compute nobootx=1.  compute modres2=999.  compute v=trunc(uniform(n,1)*n)+1.  compute bad=0.  loop i = 1 to (nms+nys).  compute y=outvars(v,i).  compute ynovar= (nrow(y)*sscp(y))-(t(csum(y))*(csum(y))).  do if (ynovar = 0).  compute bad=1.  end if.  compute xindx=datindx(1:(nump(1,i)-1),i).  compute hello=0.  compute x = fulldat(v,xindx).  compute x={ones,x}.  compute xsq=t(x)*x.  compute exsq=eval(xsq).  release xsq.  compute holymoly=cmin(exsq).  compute zeroeig=csum(exsq <= 0.000000000002).  compute bad=bad + (zeroeig > 0).  .  compute desctmp=make((8-(4* 1 )),ncol( y ),-999).  loop jd=1 to ncol( y ).  compute descdat= y (:,jd).  compute desctmp(1,jd) = csum(descdat)/nrow(descdat).  compute desctmp(2,jd) = (nrow(descdat)*sscp(descdat))-(t(csum(descdat))*(csum(descdat))).  compute desctmp(2,jd) = sqrt(desctmp(2,jd)/(nrow(descdat)*(nrow(descdat)-1))).  compute desctmp(3,jd)=cmin(descdat).  compute desctmp(4,jd)=cmax(descdat).  do if ( 1 =0).  compute minwarn=0.  compute maxwarn=0.  do if ((desctmp(3,jd)=desctmp(4,jd)) and novar=0).  compute errcode(errs,1)=15.  compute errs=errs+1.  compute criterr=1.  compute novar=1.  end if.  compute tmp=((descdat(:,1)=desctmp(3,jd))+(descdat(:,1)=desctmp(4,jd))).  compute desctmp(8,jd)=(csum(tmp)=nrow(tmp)).  compute tmp = descdat.  compute tmp(GRADE(descdat),:) = descdat.  compute descdat = tmp.  release tmp.  compute decval={.16;.5;.84}.  loop kd=1 to 3.  compute low=trunc(decval(kd,1)*(nrow(descdat)+1)).  compute lowdec=decval(kd,1)*(nrow(descdat)+1)-low.  compute value=descdat(low,1)+(descdat((low+1),1)-descdat(low,1))*lowdec.  compute desctmp((4+kd),jd)=value.  end loop.  compute mnotev=1.  compute modvals=desctmp(5:7,:).  do if (quantile <> 1).  compute desctmp(5,jd)=desctmp(1,jd)-desctmp(2,jd).  compute desctmp(6,jd)=desctmp(1,jd).  compute desctmp(7,jd)=desctmp(1,jd)+desctmp(2,jd).  compute modvals=desctmp(5:7,:).  compute mnotev=2.  do if (modvals(1,1) < desctmp(3,1)).  compute modvals(1,1)=desctmp(3,1).  compute minwarn=1.  end if.  do if (modvals(3,1) > desctmp(4,1)).  compute modvals(3,1)=desctmp(4,1).  compute maxwarn=1.  end if.  end if.  do if (desctmp(8,1)=1).  compute modvals={desctmp(3,1);desctmp(4,1)}.  compute mnotev=0.  compute minwarn=0.  compute maxwarn=0.  end if.  end if.  end loop  .  compute bad=bad+ (desctmp(2,1) <= 0.00000000001).  do if (bad = 0).  do if (holymoly < smallest).  compute smallest=holymoly.  end if.  do if (ydich=0 or (i < (nms+nys))).  .  do if ( 1 =1).  compute b = inv(t( x )* x )*t( x )* y.  compute modres=b.  do if ( 0 =1).  compute n1=nrow( x ).  compute dfres=n1-(ncol( x )).  compute sstotal = t( y -(csum( y )/n1))*( y -(csum( y )/n1)).  compute resid= y - x *b.  compute ssresid = csum((resid)&**2).  compute r2 = (sstotal-ssresid)/sstotal.  compute adjr2 = 1-((1-r2)*(n1-1)/(dfres)).  compute mse=ssresid/(n1-ncol( x )).  .  compute n1=nrow( x ).  compute invXtX = inv(t( x )* x ).  compute varb = mse *invXtX.  compute k3 = ncol( x ).  compute xhc=0.  do if ( hc <> 5).  compute xhc= x.  compute hat = xhc(:,1).  loop i3=1 to nrow(xhc).  compute hat(i3,1)= xhc(i3,:)*invXtX*t(xhc(i3,:)).  end loop.  do if ( hc = 0 or hc =1).  loop i3 = 1 to k3.  compute xhc(:,i3)=xhc(:,i3)&* resid.  end loop.  end if.  do if ( hc =3 or hc =2).  loop i3=1 to k3.  compute xhc(:,i3) = ( resid &/(1-hat)&**(1/(4hc )))&*xhc(:,i3).  end loop.  end if.  do if ( hc = 4).  compute hcmn=make(n,2,4).  compute hcmn(:,2)=(n1*hat)/k3.  loop i3= 1 to k3.  compute xhc(:,i3) = ( resid &/(1-hat)&**(rmin(hcmn)/2))&*xhc(:,i3).  end loop.  end if.  compute varb=(invXtX*t(xhc)*xhc*invXtX).  do if ( hc =1).  compute varb=(n1/(n1-ncol( x )))&*varb.  end if.  end if.  compute hclab={'se(HC0)','se(HC1)','se(HC2)','se(HC3)','se(HC4)','se'}.  compute hclab=hclab(1,( hc +1)).  compute hcflab={'F(HC0)','F(HC1)','F(HC2)','F(HC3)','F(HC4)','F'}.  compute hcflab=hcflab(1,( hc +1)).  release xhc  .  compute seb=sqrt(diag(varb)).  compute trat = b&/seb.  compute p = 2*(1-tcdf(abs(trat), (dfres))).  compute tval = sqrt(dfres* (exp((dfres-(5/6))*((xp2/(dfres-(2/3)+(.11/dfres)))* (xp2/(dfres-(2/3)+(.11/dfres)))))-1)).  compute modres={modres,seb,trat,p}.  compute modres={modres,(b-tval&*seb),(b+tval&*seb)}.  compute modresl={'coeff',hclab,'t','p','LLCI','ULCI'}.  compute lmat = ident(ncol( x )).  compute lmat = lmat(:,2:ncol(lmat)).  compute fratio = (t(t(lmat)*b)*inv(t(lmat)*varb*lmat)*((t(lmat)*b)))/(ncol( x )-1).  compute pfr = 1-fcdf(fratio,(ncol( x )-1),dfres).  compute modsum={sqrt(r2),r2,mse,fratio,(ncol( x )-1),dfres,pfr}.  compute modsuml={'R','R-sq','MSE',hcflab,'df1','df2', 'p'}.  end if.  end if.  do if ( 1 = 2 or 1 =3).  compute xlp= x.  compute ylp= y.  compute pt2 = make(nrow(ylp),1,(csum(ylp)/nrow(ylp))).  do if ( 1 =2).  compute LL3 = ylp&*ln(pt2)+(1-ylp)&*ln(1-pt2).  end if.  compute LL3 = -2*csum(LL3).  compute bt1 = make(ncol(xlp),1,0).  compute LL1 = 0.  compute pt1 = make(nrow(ylp),1,0.5).  compute pt1lp=pt1.  loop jjj = 1 to iterate.  compute vt1 = mdiag(pt1lp&*(1-pt1lp)).  compute b = bt1+inv(t(xlp)*vt1*xlp)*t(xlp)*(ylp-pt1lp).  do if ( 1 =2).  compute xlpb=xlp*b.  compute xlpbt=(xlpb > -709.7).  compute xlpb709=(1-xlpbt)*(-709.7).  compute xlpb=(xlpb&*xlpbt)+xlpb709.  compute pt1lp = 1/(1+exp(-(xlpb))).  end if.  compute itprob = csum((pt1lp < .00000001) or (pt1lp > .9999999)).  do if (itprob > 0).  loop kkk = 1 to nrow(pt1lp).  do if (pt1lp(kkk,1) > .9999999).  compute pt1lp(kkk,1) = .9999999.  end if.  do if (pt1lp(kkk,1) < .00000001).  compute pt1lp(kkk,1) = .00000001.  end if.  end loop.  compute itprob = 0.  end if.  do if (itprob = 0).  do if ( 1 =2).  compute LL = ylp&*ln(pt1lp)+(1-ylp)&*ln(1-pt1lp).  end if.  compute LL2 = -2*csum(ll).  end if.  do if (abs(LL1-LL2) < converge).  do if ( 0 =1).  compute vt1 = mdiag(pt1lp&*(1-pt1lp)).  compute varb = inv(t(xlp)*vt1*xlp).  compute seb = sqrt(diag(varb)).  end if.  break.  end if.  compute bt1 = b.  compute LL1 = LL2.  end loop.  compute modres=b.  do if (jjj > iterate).  compute itprob = 2.  do if (booting=0).  compute iterrmod=1.  end if.  do if (booting=1).  compute bootiter=1.  end if.  do if (itprobtg=0).  compute itprobtg=1.  compute errcode(errs,1) = 47.  compute errs = errs + 1.  do if (booting = 0 and 0 =1).  compute vt1 = mdiag(pt1lp&*(1-pt1lp)).  compute varb = inv(t(xlp)*vt1*xlp).  compute seb = sqrt(diag(varb)).  end if.  end if.  end if.  do if ( 0 =1).  compute trat = b&/seb.  compute dfres=nrow(xlp).  compute p = 2*(1-cdfnorm(abs(trat))).  compute modres={modres,seb,trat,p}.  compute modres={modres,(b-xp2&*seb),(b+xp2&*seb)}.  compute pvchi=1-chicdf((LL3-LL2),(nrow(modres)-1)).  compute mcF = (LL3-LL2)/LL3.  compute cox = 1-exp(-(LL3-LL2)/nrow(xlp)).  compute nagel = cox/(1-exp(-(LL3)/nrow(xlp))).  compute modsum={LL2,(LL3-LL2),(nrow(modres)-1),pvchi, mcF,cox,nagel}.  compute modsuml={'-2LL','ModelLL', 'df', 'p', 'McFadden', 'CoxSnell', 'Nagelkrk'}.  compute modresl={'coeff','se','Z','p','LLCI','ULCI'}.  end if.  end if  .  end if.  do if (ydich=1 and (i = (nms+nys))).  .  do if ( 2 =1).  compute b = inv(t( x )* x )*t( x )* y.  compute modres=b.  do if ( 0 =1).  compute n1=nrow( x ).  compute dfres=n1-(ncol( x )).  compute sstotal = t( y -(csum( y )/n1))*( y -(csum( y )/n1)).  compute resid= y - x *b.  compute ssresid = csum((resid)&**2).  compute r2 = (sstotal-ssresid)/sstotal.  compute adjr2 = 1-((1-r2)*(n1-1)/(dfres)).  compute mse=ssresid/(n1-ncol( x )).  .  compute n1=nrow( x ).  compute invXtX = inv(t( x )* x ).  compute varb = mse *invXtX.  compute k3 = ncol( x ).  compute xhc=0.  do if ( hc <> 5).  compute xhc= x.  compute hat = xhc(:,1).  loop i3=1 to nrow(xhc).  compute hat(i3,1)= xhc(i3,:)*invXtX*t(xhc(i3,:)).  end loop.  do if ( hc = 0 or hc =1).  loop i3 = 1 to k3.  compute xhc(:,i3)=xhc(:,i3)&* resid.  end loop.  end if.  do if ( hc =3 or hc =2).  loop i3=1 to k3.  compute xhc(:,i3) = ( resid &/(1-hat)&**(1/(4hc )))&*xhc(:,i3).  end loop.  end if.  do if ( hc = 4).  compute hcmn=make(n,2,4).  compute hcmn(:,2)=(n1*hat)/k3.  loop i3= 1 to k3.  compute xhc(:,i3) = ( resid &/(1-hat)&**(rmin(hcmn)/2))&*xhc(:,i3).  end loop.  end if.  compute varb=(invXtX*t(xhc)*xhc*invXtX).  do if ( hc =1).  compute varb=(n1/(n1-ncol( x )))&*varb.  end if.  end if.  compute hclab={'se(HC0)','se(HC1)','se(HC2)','se(HC3)','se(HC4)','se'}.  compute hclab=hclab(1,( hc +1)).  compute hcflab={'F(HC0)','F(HC1)','F(HC2)','F(HC3)','F(HC4)','F'}.  compute hcflab=hcflab(1,( hc +1)).  release xhc  .  compute seb=sqrt(diag(varb)).  compute trat = b&/seb.  compute p = 2*(1-tcdf(abs(trat), (dfres))).  compute tval = sqrt(dfres* (exp((dfres-(5/6))*((xp2/(dfres-(2/3)+(.11/dfres)))* (xp2/(dfres-(2/3)+(.11/dfres)))))-1)).  compute modres={modres,seb,trat,p}.  compute modres={modres,(b-tval&*seb),(b+tval&*seb)}.  compute modresl={'coeff',hclab,'t','p','LLCI','ULCI'}.  compute lmat = ident(ncol( x )).  compute lmat = lmat(:,2:ncol(lmat)).  compute fratio = (t(t(lmat)*b)*inv(t(lmat)*varb*lmat)*((t(lmat)*b)))/(ncol( x )-1).  compute pfr = 1-fcdf(fratio,(ncol( x )-1),dfres).  compute modsum={sqrt(r2),r2,mse,fratio,(ncol( x )-1),dfres,pfr}.  compute modsuml={'R','R-sq','MSE',hcflab,'df1','df2', 'p'}.  end if.  end if.  do if ( 2 = 2 or 2 =3).  compute xlp= x.  compute ylp= y.  compute pt2 = make(nrow(ylp),1,(csum(ylp)/nrow(ylp))).  do if ( 2 =2).  compute LL3 = ylp&*ln(pt2)+(1-ylp)&*ln(1-pt2).  end if.  compute LL3 = -2*csum(LL3).  compute bt1 = make(ncol(xlp),1,0).  compute LL1 = 0.  compute pt1 = make(nrow(ylp),1,0.5).  compute pt1lp=pt1.  loop jjj = 1 to iterate.  compute vt1 = mdiag(pt1lp&*(1-pt1lp)).  compute b = bt1+inv(t(xlp)*vt1*xlp)*t(xlp)*(ylp-pt1lp).  do if ( 2 =2).  compute xlpb=xlp*b.  compute xlpbt=(xlpb > -709.7).  compute xlpb709=(1-xlpbt)*(-709.7).  compute xlpb=(xlpb&*xlpbt)+xlpb709.  compute pt1lp = 1/(1+exp(-(xlpb))).  end if.  compute itprob = csum((pt1lp < .00000001) or (pt1lp > .9999999)).  do if (itprob > 0).  loop kkk = 1 to nrow(pt1lp).  do if (pt1lp(kkk,1) > .9999999).  compute pt1lp(kkk,1) = .9999999.  end if.  do if (pt1lp(kkk,1) < .00000001).  compute pt1lp(kkk,1) = .00000001.  end if.  end loop.  compute itprob = 0.  end if.  do if (itprob = 0).  do if ( 2 =2).  compute LL = ylp&*ln(pt1lp)+(1-ylp)&*ln(1-pt1lp).  end if.  compute LL2 = -2*csum(ll).  end if.  do if (abs(LL1-LL2) < converge).  do if ( 0 =1).  compute vt1 = mdiag(pt1lp&*(1-pt1lp)).  compute varb = inv(t(xlp)*vt1*xlp).  compute seb = sqrt(diag(varb)).  end if.  break.  end if.  compute bt1 = b.  compute LL1 = LL2.  end loop.  compute modres=b.  do if (jjj > iterate).  compute itprob = 2.  do if (booting=0).  compute iterrmod=1.  end if.  do if (booting=1).  compute bootiter=1.  end if.  do if (itprobtg=0).  compute itprobtg=1.  compute errcode(errs,1) = 47.  compute errs = errs + 1.  do if (booting = 0 and 0 =1).  compute vt1 = mdiag(pt1lp&*(1-pt1lp)).  compute varb = inv(t(xlp)*vt1*xlp).  compute seb = sqrt(diag(varb)).  end if.  end if.  end if.  do if ( 0 =1).  compute trat = b&/seb.  compute dfres=nrow(xlp).  compute p = 2*(1-cdfnorm(abs(trat))).  compute modres={modres,seb,trat,p}.  compute modres={modres,(b-xp2&*seb),(b+xp2&*seb)}.  compute pvchi=1-chicdf((LL3-LL2),(nrow(modres)-1)).  compute mcF = (LL3-LL2)/LL3.  compute cox = 1-exp(-(LL3-LL2)/nrow(xlp)).  compute nagel = cox/(1-exp(-(LL3)/nrow(xlp))).  compute modsum={LL2,(LL3-LL2),(nrow(modres)-1),pvchi, mcF,cox,nagel}.  compute modsuml={'-2LL','ModelLL', 'df', 'p', 'McFadden', 'CoxSnell', 'Nagelkrk'}.  compute modresl={'coeff','se','Z','p','LLCI','ULCI'}.  end if.  end if  .  end if.  compute modres2={modres2,t(modres)}.  do if ( (bcmat((i+1),1) = 1) and (nobootx=1) and (effsize=1)).  compute nobootx=0.  compute xsdtemp= (nrow(x)*sscp(x(:,2)))-(t(csum(x(:,2)))*(csum(x(:,2)))).  compute xsdtemp= sqrt(xsdtemp/(nrow(x)*(nrow(x)-1))).  compute bootxsd={bootxsd;xsdtemp}.  end if.  end if.  end loop.  do if (bad = 0).  compute bootres={bootres;modres2(:,2:ncol(modres2))}.  do if (effsize=1).  compute ysdtemp= (nrow(y)*sscp(y))-(t(csum(y))*(csum(y))).  compute ysdtemp= sqrt(ysdtemp/(nrow(y)*(nrow(y)-1))).  compute bootysd={bootysd;ysdtemp}.  end if.  compute goodboot=goodboot+1.  end if.  do if (bad <> 0).  compute badboot=badboot+1.  end if.  end loop if (goodboot = boot).  compute bootres=bootres(2:nrow(bootres),:).  do if (effsize=1).  compute bootysd=bootysd(2:nrow(bootysd),:).  do if (nrow(bootxsd) > 1).  compute bootxsd=bootxsd(2:nrow(bootxsd),:).  end if.  end if.  do if (goodboot < (boot)).  compute boot=0.  compute modelbt=0.  compute notecode(notes,1) = 7.  compute notes = notes + 1.  end if.  do if (boot > 0).  do if (effsize=1).  compute bootysd={ysd;bootysd}.  compute bootxsd={xsd;bootxsd}.  end if.  do if (saveboot = 1).  save bootres/outfile = *.  end if.  compute bootcim=make(ncol(bootres),5,-99999).  compute bootcim(:,2) = t(csum(bootres)/nrow(bootres)).  compute bootcim(:,1) = coeffmat(2:nrow(coeffmat),1).  loop i = 1 to ncol(bootres).  .  compute temp = bootres(:,i).  compute temp(GRADE( bootres(:,i) )) = bootres(:,i).  compute badlo = 0.  compute badhi = 0.  do if ( 9999 <> 9999).  compute pv=csum(temp < 9999 )/nrow(temp).  compute ppv = pv.  do if (pv > .5).  compute ppv = 1-pv.  end if.  compute y5=sqrt(-2*ln(ppv)).  compute xp=y5+((((y5*p4+p3)*y5+p2)*y5+p1)*y5+p0)/((((y5*q4+q3)*y5+q2)*y5+q1)*y5+q0).  do if (pv <= .5).  compute xp = -xp.  end if.  compute cilow=rnd(nrow(temp)*(cdfnorm(2*xp+xp2))).  compute cihigh=trunc(nrow(temp)*(cdfnorm(2*xp+(-xp2))))+1.  do if (cilow < 1).  compute cilow = 1.  compute booterr=1.  compute badlo = 1.  end if.  do if (cihigh > nrow(temp)).  compute cihigh = boot.  compute booterr=1.  compute badhi = 1.  end if.  compute llcit=temp(cilow,1).  compute ulcit=temp(cihigh,1).  end if.  do if ( 9999 = 9999).  compute llcit=temp(cilow,1).  compute ulcit=temp(cihigh,1).  end if.  compute bootse=t(sqrt((cssq(temp)-((csum(temp)&**2)/nrow(temp)))/(nrow(temp)-1)))  .  compute bootcim(i,4:5)={llcit,ulcit}.  compute bootcim(i,3)=bootse.  end loop.  end if.  do if (badboot > 0).  compute notecode(notes,1) = 6.  compute notes = notes + 1.  end if.  end if.  do if (criterr=0).  release fulldat,x,y.  end if.  .  do if (criterr = 0 and nms > 0).  compute paths=paths(:,2:ncol(paths)).  compute pathsw=pathsw(:,2:ncol(pathsw)).  compute pathsz=pathsz(:,2:ncol(pathsz)).  compute pathswz=pathswz(:,2:ncol(pathswz)).  compute pathsmod=pathsw+pathsz+pathswz.  compute pathsdv=pathsdv(:,2:ncol(pathsdv)).  compute pathsfoc=pathsfoc(:,2:ncol(pathsfoc)).  compute pathtype=pathtype(:,2:ncol(pathtype)).  compute anymod=(rsum(pathsmod) > 0).  compute obscoeff=obscoeff(1,2:ncol(obscoeff)).  do if (dototal = 0).  print/title = '****************** DIRECT AND INDIRECT EFFECTS OF X ON Y *****************'.  end if.  do if (dototal = 1).  print/title = '************** TOTAL, DIRECT, AND INDIRECT EFFECTS OF X ON Y **************'.  compute totefflb(1,1)='Effect'.  do if (counterf <> 1).  do if (effsize=1).  compute toteffsz=toteff(:,1)/ysd.  compute totefflb={totefflb,'c_ps'}.  do if (xdich = 0 and mcx = 0).  compute toteffsz={toteffsz, (toteffsz*xsd)}.  compute totefflb={totefflb,'c_cs'}.  end if.  compute toteff={toteff,toteffsz}.  end if.  do if (nxvls > 1).  print toteff/title='Relative total effects of X on Y:'/rnames=toteffl2/cnames=totefflb/format= F10.4.  compute clabtmp={'R2-chng', hcflab, 'df1','df2','p'}.  print totomni/title='Omnibus test of total effect of X on Y:'/cnames=clabtmp/format= F10.4.  print/title= '----------'/space=0.  else.  print toteff/title='Total effect of X on Y'/cnames=totefflb/format= F10.4.  end if.  end if.  end if.  compute moddir=wcmat(nrow(bcmat),1)+zcmat(nrow(bcmat),1).  do if (bcmat(nrow(bcmat),1)=1 and counterf <> 1).  do if (ydich=1).  compute direfflb(:,(ncol(direfflb)-5):ncol(direfflb))={'Effect','se','Z','p','LLCI','ULCI'}.  end if.  do if (moddir=0).  compute direfflb(1,1)='Effect'.  end if.  do if (effsize=1 and moddir=0 and anymod = 0).  compute direffsz=direff(:,1)/ysd.  compute direfflb={direfflb,'c''_ps'}.  do if (xdich = 0 and mcx = 0).  compute direffsz={direffsz, (direffsz*xsd)}.  compute direfflb={direfflb,'c''_cs'}.  end if.  compute direff={direff,direffsz}.  end if.  do if (moddir = 0 and nxvls=1).  print direff/title='Direct effect of X on Y'/cnames=direfflb/format= F10.4.  end if.  do if (moddir = 0 and nxvls>1).  print direff/title='Relative direct effects of X on Y'/rnames=direffl2/cnames=direfflb/format= F10.4.  do if (ydich = 0).  compute clabtmp={'R2-chng', hcflab, 'df1','df2','p'}.  print diromni/title='Omnibus test of direct effect of X on Y:'/cnames=clabtmp/format= F10.4.  end if.  do if (ydich = 1).  compute clabtmp={'Chi-sq', 'df', 'p'}.  print diromni/title='Omnibus likelihood ratio test of direct effect of X on Y:'/cnames=clabtmp/format= F10.4.  end if.  print/title= '----------'/space=0.  end if.  do if (moddir > 0 and nxvls=1).  print direff/title='Conditional direct effect(s) of X on Y:'/cnames=direfflb/format= F10.4.  end if.  do if (moddir > 0 and nxvls>1).  compute direffl2={' '}.  loop i = 1 to nxvls.  loop j = 1 to (nrow(direff)/nxvls).  compute direffl2={direffl2;xcatlab(i,1)}.  end loop.  end loop.  compute direffl2=direffl2(2:nrow(direffl2),1).  print direff/title='Relative conditional direct effect(s) of X on Y:'/cnames=direfflb/rnames=direffl2/format= F10.4.  end if.  end if.  do if (bcmat(nrow(bcmat),1)=0 and counterf <> 1).  print/title='The direct effect of X on Y is fixed to zero.'.  end if.  do if (nms = 1).  compute indmark={2}.  compute indsets={1,2}.  end if.  do if (nms = 2).  compute indmark={2,2,3}.  compute indsets={1,4,2,5,1,3,5}.  compute thetam=1.  end if.  do if (nms = 3).  compute indmark={2,2,2,3,3,3,4}.  compute indsets={1,7,2,8,4,9,1,3,8,1,5,9,2,6,9,1,3,6,9}.  compute thetam={1,2,3}.  end if.  do if (nms = 4).  compute indmark={2,2,2,2,3,3,3,3,3,3,4,4,4,4,5}.  compute indsets={1,11,2,12,4,13,7,14,1,3,12,1,5,13,1,8,14,2,6,13,2,9,14,4,10,14,1,3,6,13,1,3,9, 14,1,5,10,14,2,6,10,14,1,3,6,10,14}.  compute thetam={1,2,4,3,5,6}.  end if.  do if (nms = 5).  compute indmark={2,2,2,2,2,3,3,3,3,3,3,3,3,3,3,4,4,4,4,4,4,4,4,4,4,5,5,5,5,5,6}.  compute indsets={1,16,2,17,4,18,7,19,11,20,1,3,17,1,5,18,1,8,19,1,12,20,2,6,18,2,9,19,2,13,20,4, 10,19,4,14,20,7,15,20,1,3,6,18, 1,3,9,19,1,3,13,20,1,5,10,19,1,5,14,20,1,8,15,20,2,6,10,19,2,6, 14,20,2,9,15,20,4,10,15,20,1,3,6,10,19, 1,3,6,14,20,1,3,9,15,20,1,5,10,15,20,2,6,10,15,20,1,3,6,10,15, 20}.  compute thetam={1,2,5,3,6,8,4,7,9,10}.  end if.  do if (nms = 6).  compute indmark={2,2,2,2,2,2,3,3,3,3,3,3,3,3,3,3,3,3,3,3,3,4,4,4,4,4,4,4,4,4,4,4,4,4,4,4,4,4,4, 4,4,5,5,5,5,5,5,5,5,5,5,5,5,5,5,5,6,6,6,6,6,6,7}.  compute indsets={1,22,2,23,4,24,7,25,11,26,16,27,1,3,23,1,5,24,1,8,25,1,12,26,1,17,27,2,6,24,2, 9,25,2,13,26,2,18,27,4,10,25,4,14,26, 4,19,27,7,15,26,7,20,27,11,21,27,1,3,6,24,1,3,9,25,1,3,13,26,1, 3,18,27,1,5,10,25,1,5,14,26,1,5,19,27,1,8,15,26,1,8,20,27, 1,12,21,27,2,6,10,25,2,6,14,26,2,6,19,27,2,9,15,26,2,9,20,27,2, 13,21,27,4,10,15,26,4,10,20,27,4,14,21,27,7,15,21,27, 1,3,6,10,25,1,3,6,14,26,1,3,6,19,27,1,3,9,15,26,1,3,9,20,27,1,3, 13,21,27,1,5,10,15,26,1,5,10,20,27,1,5,14,21,27, 1,8,15,21,27,2,6,10,15,26,2,6,10,20,27,2,6,14,21,27,2,9,15,21, 27,4,10,15,21,27,1,3,6,10,15,26,1,3,6,10,20,27, 1,3,6,14,21,27,1,3,9,15,21,27,1,5,10,15,21,27,2,6,10,15,21,27, 1,3,6,10,15,21,27}.  compute thetam={1,2,6,3,7,10,4,8,11,13,5,9,12,14,15}.  end if.  do if (nms = 7).  compute indmark={2,2,2,2,2,2,2}.  compute indsets={1,29,2,30,4,31,7,32,11,33,16,34,22,35}.  end if.  do if (nms = 8).  compute indmark={2,2,2,2,2,2,2,2}.  compute indsets={1,37,2,38,4,39,7,40,11,41,16,42,22,43,29,44}.  end if.  do if (nms = 9).  compute indmark={2,2,2,2,2,2,2,2,2}.  compute indsets={1,46,2,47,4,48,7,49,11,50,16,51,22,52,29,53,37,54}.  end if.  do if (nms=10).  compute indmark={2,2,2,2,2,2,2,2,2,2}.  compute indsets={1,56,2,57,4,58,7,59,11,60,16,61,22,62,29,63,37,64,46,65}.  end if.  compute indlbl = {'Ind1'; 'Ind2'; 'Ind3'; 'Ind4'; 'Ind5'; 'Ind6'; 'Ind7'; 'Ind8'; 'Ind9'; 'Ind10'; 'Ind11'; 'Ind12'; 'Ind13'; 'Ind14'; 'Ind15'}.  compute indlbl = {indlbl; 'Ind16';'Ind17';'Ind18';'Ind19';'Ind20';'Ind21';'Ind22';'Ind23';'Ind24';'Ind25';'Ind26';'Ind27';'Ind28';'Ind29';'Ind30'}.  compute indlbl = {indlbl; 'Ind31';'Ind32';'Ind33';'Ind34';'Ind35';'Ind36';'Ind37';'Ind38';'Ind39';'Ind40';'Ind41';'Ind42';'Ind43';'Ind44';'Ind45'}.  compute indlbl = {indlbl; 'Ind46';'Ind47';'Ind48';'Ind49';'Ind50';'Ind51';'Ind52';'Ind53';'Ind54';'Ind55';'Ind56';'Ind57';'Ind58';'Ind59';'Ind60'}.  compute indlbl = {indlbl; 'Ind61';'Ind62';'Ind63'}.  compute cntname={'(C1)';'(C2)';'(C3)';'(C4)';'(C5)';'(C6)';'(C7)';'(C8)';'(C9)';'(C10)';'(C11)';'(C12)';'(C13)';'(C14)';'(C15)';'(C16)';'(C17)'}.  compute cntname={cntname;'(C18)';'(C19)';'(C20)';'(C21)';'(C22)';'(C23)';'(C24)';'(C25)';'(C26)';'(C27)';'(C28)';'(C29)';'(C30)';'(C31)'}.  compute cntname={cntname;'(C32)';'(C33)';'(C34)';'(C35)';'(C36)';'(C37)';'(C38)';'(C39)';'(C40)';'(C41)';'(C42)';'(C43)';'(C44)';'(C45)'}.  compute cntname={cntname;'(C46)';'(C47)';'(C48)';'(C49)';'(C50)';'(C51)';'(C52)';'(C53)';'(C54)';'(C55)';'(C56)';'(C57)';'(C58)';'(C59)'}.  compute cntname={cntname;'(C60)';'(C61)';'(C62)';'(C63)';'(C64)';'(C65)';'(C66)';'(C67)';'(C68)';'(C69)';'(C70)';'(C71)';'(C72)';'(C73)'}.  compute cntname={cntname;'(C74)';'(C75)';'(C76)';'(C77)';'(C78)';'(C79)';'(C80)';'(C81)';'(C82)';'(C83)';'(C84)';'(C85)';'(C86)';'(C87)'}.  compute cntname={cntname;'(C88)';'(C89)';'(C90)';'(C91)';'(C92)';'(C93)';'(C94)';'(C95)';'(C96)';'(C97)';'(C98)';'(C99)';'(C100)';'(C101)'}.  compute cntname={cntname;'(C102)';'(C103)';'(C104)';'(C105)'}.  compute indmake=make(ncol(indmark),(nms+2),0).  compute indmod=make(ncol(indmark),1,999).  compute indmmm=make(ncol(indmark),1,0).  compute indmmmt=make(ncol(indmark),1,0).  compute start=1.  compute end=0.  compute nindfx=0.  compute indlocs=make(nrow(thetaxmb),ncol(paths),999).  compute indkey=make(ncol(indmark),1+((rmax(indmark)*2)+1),' ').  compute c1=1.  compute c2=1.  compute c3=1.  loop i = 1 to ncol(paths).  do if (pathtype(1,i)=1).  compute indlocs(:,i)=thetaxmb(:,c1).  compute c1=c1+1.  end if.  do if (pathtype(1,i)=3).  compute indlocs(:,i)=thetamyb(:,c2).  compute c2=c2+1.  end if.  do if (pathtype(1,i)=2 and nms < 7 and serial=1).  compute indlocs(:,i)=thetammb(:,thetam(1,c3)).  compute c3=c3+1.  end if.  end loop.  loop i = 1 to ncol(indlocs).  compute c1=2.  loop j = 2 to nrow(indlocs).  do if (indlocs(j,i) <> 0).  compute indlocs(c1,i)=indlocs(j,i).  compute c1=c1+1.  end if.  end loop.  compute indlocs(1,i)=c1-2.  end loop.  compute indlocs=indlocs(1:rmax((indlocs(1,:))+1),:).  loop i = 1 to ncol(indmark).  compute numget=indmark(1,i).  compute end=end+numget.  compute gotcha=indsets(1,start:end).  compute start=end+1.  compute ok=1.  compute temp=0.  compute repoman=make(4,1,0).  loop j = 1 to ncol(gotcha).  do if paths(1,gotcha(1,j))=0.  compute ok=0.  end if.  do if (pathsmod(1,gotcha(1,j)) > 0).  compute temp=1.  compute temp2={pathsw(1,gotcha(1,j));pathsz(1,gotcha(1,j));pathswz(1,gotcha(1,j));0}.  do if (temp2(1,1)=1 and temp2(2,1)=1 and temp2(3,1)=0).  compute temp2(4,1)=1.  end if.  compute repoman=repoman+temp2.  end if.  end loop.  compute temp=0.  compute tempmmm=0.  compute typemmm=0.  do if ((repoman(1,1) > 0) and (repoman(2,1) = 0)).  compute temp=1.  do if (repoman(1,1)=1).  compute tempmmm=1.  end if.  do if (repoman(1,1) > 1 and (wdich=1 or mcw > 0)).  compute tempmmm=12.  compute typemmm=mcw.  do if (wdich=1).  compute typemmm=1.  end if.  end if.  do if (repoman(1,1) > 1 and (wdich=0 and mcw = 0)).  compute tempmmm=101.  end if.  end if.  do if ((repoman(1,1) = 0) and (repoman(2,1) > 0)).  compute temp=2.  do if (repoman(2,1)=1).  compute tempmmm=2.  end if.  do if (repoman(2,1) > 1 and (zdich = 1 or mcz > 0)).  compute tempmmm=22.  compute typemmm=mcz.  do if (zdich=1).  compute typemmm=1.  end if.  end if.  do if (repoman(2,1) > 1 and (zdich = 0 and mcw = 0)).  compute tempmmm=102.  end if.  end if.  do if (repoman(1,1)>0 and repoman(2,1)>0).  compute temp=3.  do if (repoman(1,1)=1 and repoman(2,1)=1).  do if (repoman(4,1)=1).  compute tempmmm=31.  end if.  do if (repoman(3,1)=1).  compute tempmmm=41.  end if.  end if.  end if.  do if (repoman(1,1)=1 and repoman(2,1)=1 and repoman(3,1)=0 and repoman(4,1)=0).  compute tempmmm=51.  end if.  do if (ok = 1).  compute nindfx=nindfx+1.  compute indmake(nindfx,1)=numget.  compute indmod(nindfx,1)=temp.  compute indmmm(nindfx,1)=tempmmm.  compute indmmmt(nindfx,1)=typemmm.  compute indmake(nindfx,2:(1+numget))=gotcha.  compute indkey(nindfx,1)=xnames.  loop j = 1 to numget.  compute indkey(nindfx,(j*2+1))=pathsdv(1,gotcha(1,j)).  compute indkey(nindfx,(j*2))=' -> '.  end loop.  end if.  end loop.  compute indkey=indkey(1:nindfx,1:((cmax(indmake(:,1))*2)+1)).  compute indmake=indmake(1:nindfx,1:(cmax(indmake(:,1))+1)).  compute indmod=indmod(1:nrow(indmake),1).  compute indmmm=indmmm(1:nrow(indmake),1).  compute indmmmt=indmmmt(1:nrow(indmake),1).  compute ncpairs = (((nindfx)*(nindfx-1))/2).  do if ((contrast = 1 or contrast = 2) and (ncpairs > 105)).  compute contrast=0.  compute notecode(notes,1) = 13.  compute notes = notes + 1.  end if.  do if (contrast = 3).  do if (ncol(contvec) <> nindfx).  compute contrast=0.  compute notecode(notes,1) = 14.  compute notes = notes + 1.  end if.  end if.  do if (anymod=0).  do if (nms = 1 and contrast > 0).  compute contrast=0.  end if.  compute efloop=(((1-(effsize=0))*2)+1)-(((mcx>0 or xdich=1))*(1-(effsize=0))).  loop kk=1 to efloop.  do if (boot = 0).  compute bootres=obscoeff.  compute indtab=999.  compute inddiff=999.  compute bootysd=ysd.  compute bootxsd=xsd.  end if.  do if (boot > 0).  compute bootres={obscoeff;bootres}.  compute indtab=make(1,4,999).  compute inddiff=make(nrow(bootres),1,999).  end if.  compute indtotal=make(nrow(bootres),1,0).  loop i = 1 to nrow(indmake).  loop j = 1 to nxvls.  compute indtemp=make(nrow(bootres),1,1).  loop k = 1 to indmake(i,1).  compute jtemp=1.  do if (j > 1 and k=1).  compute jtemp=j.  end if.  compute indtemp=indtemp&*bootres(:,pathsfoc(jtemp,indmake(i,(k+1)))).  end loop.  do if (kk = 2).  compute indtemp=indtemp/bootysd.  end if.  do if (kk = 3).  compute indtemp=(bootxsd&*indtemp)/bootysd.  end if.  do if (contrast <> 0).  compute inddiff={inddiff,indtemp}.  end if.  do if (nxvls=1).  compute indtotal=indtotal+indtemp.  end if.  compute indeff=indtemp(1,1).  do if (boot > 0).  .  compute temp = indtemp(2:nrow(indtemp),1).  compute temp(GRADE( indtemp(2:nrow(indtemp),1) )) = indtemp(2:nrow(indtemp),1).  compute badlo = 0.  compute badhi = 0.  do if ( 9999 <> 9999).  compute pv=csum(temp < 9999 )/nrow(temp).  compute ppv = pv.  do if (pv > .5).  compute ppv = 1-pv.  end if.  compute y5=sqrt(-2*ln(ppv)).  compute xp=y5+((((y5*p4+p3)*y5+p2)*y5+p1)*y5+p0)/((((y5*q4+q3)*y5+q2)*y5+q1)*y5+q0).  do if (pv <= .5).  compute xp = -xp.  end if.  compute cilow=rnd(nrow(temp)*(cdfnorm(2*xp+xp2))).  compute cihigh=trunc(nrow(temp)*(cdfnorm(2*xp+(-xp2))))+1.  do if (cilow < 1).  compute cilow = 1.  compute booterr=1.  compute badlo = 1.  end if.  do if (cihigh > nrow(temp)).  compute cihigh = boot.  compute booterr=1.  compute badhi = 1.  end if.  compute llcit=temp(cilow,1).  compute ulcit=temp(cihigh,1).  end if.  do if ( 9999 = 9999).  compute llcit=temp(cilow,1).  compute ulcit=temp(cihigh,1).  end if.  compute bootse=t(sqrt((cssq(temp)-((csum(temp)&**2)/nrow(temp)))/(nrow(temp)-1)))  .  compute indeff={indeff,bootse,llcit,ulcit}.  end if.  compute indtab={indtab;indeff}.  end loop.  end loop.  compute indtab=indtab(2:nrow(indtab),:).  compute rowlbs=indlbl(1:nrow(indtab),1).  do if (mc > 0).  compute inddiff=make(mc,1,-999).  compute indtab2=make(nrow(indtab),4,-999).  compute indtab2(:,1)=indtab.  compute indtab=indtab2.  release indtab2.  compute mcct=0.  compute indtotal=make(mc,1,0).  do if (kk = 1).  compute x1 = sqrt(-2*ln(uniform(mc,nrow(mcsopath))))&*cos((2*3.14159265358979)*uniform(mc, nrow(mcsopath))).  compute x1=x1*chol(indcov).  loop ii=1 to nrow(x1).  compute x1(ii,:)=x1(ii,:)+t(mcsopath).  end loop.  end if.  loop ii=1 to nms.  compute tmpb=x1(:,((nms*nxvls)+ii)).  compute tmpb2=tmpb.  do if (nxvls > 1).  loop jj=1 to (nxvls-1).  compute tmpb2={tmpb2,tmpb}.  end loop.  end if.  compute indtemp=x1(:,(((ii-1)*nxvls)+1):(ii*nxvls))&*tmpb2.  loop jj=1 to ncol(indtemp).  do if (kk = 2).  compute indtemp(:,jj)=indtemp(:,jj)/ysd.  end if.  do if (kk = 3).  compute indtemp(:,jj)=(xsd*indtemp(:,jj))/ysd.  end if.  .  compute temp = indtemp(:,jj).  compute temp(GRADE( indtemp(:,jj) )) = indtemp(:,jj).  compute badlo = 0.  compute badhi = 0.  do if ( 9999 <> 9999).  compute pv=csum(temp < 9999 )/nrow(temp).  compute ppv = pv.  do if (pv > .5).  compute ppv = 1-pv.  end if.  compute y5=sqrt(-2*ln(ppv)).  compute xp=y5+((((y5*p4+p3)*y5+p2)*y5+p1)*y5+p0)/((((y5*q4+q3)*y5+q2)*y5+q1)*y5+q0).  do if (pv <= .5).  compute xp = -xp.  end if.  compute cilow=rnd(nrow(temp)*(cdfnorm(2*xp+xp2))).  compute cihigh=trunc(nrow(temp)*(cdfnorm(2*xp+(-xp2))))+1.  do if (cilow < 1).  compute cilow = 1.  compute booterr=1.  compute badlo = 1.  end if.  do if (cihigh > nrow(temp)).  compute cihigh = boot.  compute booterr=1.  compute badhi = 1.  end if.  compute llcit=temp(cilow,1).  compute ulcit=temp(cihigh,1).  end if.  do if ( 9999 = 9999).  compute llcit=temp(cilow,1).  compute ulcit=temp(cihigh,1).  end if.  compute bootse=t(sqrt((cssq(temp)-((csum(temp)&**2)/nrow(temp)))/(nrow(temp)-1)))  .  compute mcct=mcct+1.  compute indtab(mcct,2:4)={bootse,llcit,ulcit}.  end loop.  do if (nxvls=1).  compute indtotal=indtotal+indtemp.  do if (contrast <> 0).  compute inddiff={inddiff,indtemp}.  end if.  end if.  end loop.  release indtemp,tmpb.  end if.  do if (normal = 1 and sobelok=1).  compute sobelmat=indtab(:,1).  compute sobelmat={sobelmat,(sobelmat/2),sobelmat,sobelmat}.  loop ii=1 to nms.  compute se2b=indcov(((nms*nxvls)+ii),((nms*nxvls)+ii)).  compute bpath2=mcsopath(((nms*nxvls)+ii),1)&**2.  compute se2a=diag(indcov((((ii-1)*nxvls)+1):(ii*nxvls),(((ii-1)*nxvls)+1):(ii*nxvls))).  compute apath2=mcsopath((((ii-1)*nxvls)+1):(ii*nxvls) ,1)&**2.  compute sesobel=sqrt(apath2*se2b+bpath2*se2a+se2a*se2b).  compute sobelmat((((ii-1)*nxvls)+1):(ii*nxvls),2)=sesobel.  end loop.  release se2b,bpath2,se2a,apath2,sesobel,ii.  compute sobelmat(:,3)=sobelmat(:,1)&/sobelmat(:,2).  compute sobelmat(:,4) = 2*(1-cdfnorm(abs(sobelmat(:,3)))).  end if.  do if (serial = 0).  compute rowlbs=t(mnames).  end if.  do if (nxvls=1 and nms > 1).  compute rowlbs={'TOTAL';rowlbs}.  compute indtemp=indtotal(1,1).  do if (boot > 0 and nxvls=1).  .  compute temp = indtotal(2:nrow(indtotal),1).  compute temp(GRADE( indtotal(2:nrow(indtotal),1) )) = indtotal(2:nrow(indtotal),1).  compute badlo = 0.  compute badhi = 0.  do if ( 9999 <> 9999).  compute pv=csum(temp < 9999 )/nrow(temp).  compute ppv = pv.  do if (pv > .5).  compute ppv = 1-pv.  end if.  compute y5=sqrt(-2*ln(ppv)).  compute xp=y5+((((y5*p4+p3)*y5+p2)*y5+p1)*y5+p0)/((((y5*q4+q3)*y5+q2)*y5+q1)*y5+q0).  do if (pv <= .5).  compute xp = -xp.  end if.  compute cilow=rnd(nrow(temp)*(cdfnorm(2*xp+xp2))).  compute cihigh=trunc(nrow(temp)*(cdfnorm(2*xp+(-xp2))))+1.  do if (cilow < 1).  compute cilow = 1.  compute booterr=1.  compute badlo = 1.  end if.  do if (cihigh > nrow(temp)).  compute cihigh = boot.  compute booterr=1.  compute badhi = 1.  end if.  compute llcit=temp(cilow,1).  compute ulcit=temp(cihigh,1).  end if.  do if ( 9999 = 9999).  compute llcit=temp(cilow,1).  compute ulcit=temp(cihigh,1).  end if.  compute bootse=t(sqrt((cssq(temp)-((csum(temp)&**2)/nrow(temp)))/(nrow(temp)-1)))  .  compute indtemp={indtemp, bootse,llcit,ulcit}.  end if.  do if (mc > 0).  compute obtmc=indtab(:,1).  compute indtemp=csum(obtmc).  .  compute temp = indtotal(:,1).  compute temp(GRADE( indtotal(:,1) )) = indtotal(:,1).  compute badlo = 0.  compute badhi = 0.  do if ( 9999 <> 9999).  compute pv=csum(temp < 9999 )/nrow(temp).  compute ppv = pv.  do if (pv > .5).  compute ppv = 1-pv.  end if.  compute y5=sqrt(-2*ln(ppv)).  compute xp=y5+((((y5*p4+p3)*y5+p2)*y5+p1)*y5+p0)/((((y5*q4+q3)*y5+q2)*y5+q1)*y5+q0).  do if (pv <= .5).  compute xp = -xp.  end if.  compute cilow=rnd(nrow(temp)*(cdfnorm(2*xp+xp2))).  compute cihigh=trunc(nrow(temp)*(cdfnorm(2*xp+(-xp2))))+1.  do if (cilow < 1).  compute cilow = 1.  compute booterr=1.  compute badlo = 1.  end if.  do if (cihigh > nrow(temp)).  compute cihigh = boot.  compute booterr=1.  compute badhi = 1.  end if.  compute llcit=temp(cilow,1).  compute ulcit=temp(cihigh,1).  end if.  do if ( 9999 = 9999).  compute llcit=temp(cilow,1).  compute ulcit=temp(cihigh,1).  end if.  compute bootse=t(sqrt((cssq(temp)-((csum(temp)&**2)/nrow(temp)))/(nrow(temp)-1)))  .  compute indtemp={indtemp, bootse,llcit,ulcit}.  end if.  compute indtab={indtemp;indtab}.  end if.  compute bootlbs={'Effect', 'BootSE','BootLLCI','BootULCI'}.  do if (mc > 0).  compute bootlbs={'Effect', 'MC SE','MC LLCI','MC ULCI'}.  end if.  do if (nxvls = 1).  do if (contrast <> 0).  compute inddiff=inddiff(:,2:ncol(inddiff)).  do if (mc > 0).  compute inddiff={t(obtmc);inddiff}.  end if.  do if (contrast = 3).  compute inddifft=inddiff*t(contvec).  compute indtemp=inddifft(1,1).  do if (boot > 0 or mc > 0).  .  compute temp = inddifft(2:nrow(inddifft),1).  compute temp(GRADE( inddifft(2:nrow(inddifft),1) )) = inddifft(2:nrow(inddifft),1).  compute badlo = 0.  compute badhi = 0.  do if ( 9999 <> 9999).  compute pv=csum(temp < 9999 )/nrow(temp).  compute ppv = pv.  do if (pv > .5).  compute ppv = 1-pv.  end if.  compute y5=sqrt(-2*ln(ppv)).  compute xp=y5+((((y5*p4+p3)*y5+p2)*y5+p1)*y5+p0)/((((y5*q4+q3)*y5+q2)*y5+q1)*y5+q0).  do if (pv <= .5).  compute xp = -xp.  end if.  compute cilow=rnd(nrow(temp)*(cdfnorm(2*xp+xp2))).  compute cihigh=trunc(nrow(temp)*(cdfnorm(2*xp+(-xp2))))+1.  do if (cilow < 1).  compute cilow = 1.  compute booterr=1.  compute badlo = 1.  end if.  do if (cihigh > nrow(temp)).  compute cihigh = boot.  compute booterr=1.  compute badhi = 1.  end if.  compute llcit=temp(cilow,1).  compute ulcit=temp(cihigh,1).  end if.  do if ( 9999 = 9999).  compute llcit=temp(cilow,1).  compute ulcit=temp(cihigh,1).  end if.  compute bootse=t(sqrt((cssq(temp)-((csum(temp)&**2)/nrow(temp)))/(nrow(temp)-1)))  .  compute indtemp={indtemp, bootse,llcit,ulcit}.  end if.  compute indtab={indtab;indtemp}.  end if.  do if (contrast = 1 or contrast = 2).  compute conkey=make(1,4,' ').  loop i = 1 to ncol(inddiff)-1.  loop j = (i+1) to ncol(inddiff).  compute inddifft=inddiff(:,i)-inddiff(:,j).  do if (contrast=2).  compute inddifft=abs(inddiff(:,i))-abs(inddiff(:,j)).  end if.  compute indtemp=inddifft(1,1).  compute conkeyt={' ', rowlbs((i+1),1),' minus ',rowlbs((j+1),1)}.  compute conkey={conkey;conkeyt}.  do if (boot > 0 or mc > 0).  .  compute temp = inddifft(2:nrow(inddifft),1).  compute temp(GRADE( inddifft(2:nrow(inddifft),1) )) = inddifft(2:nrow(inddifft),1).  compute badlo = 0.  compute badhi = 0.  do if ( 9999 <> 9999).  compute pv=csum(temp < 9999 )/nrow(temp).  compute ppv = pv.  do if (pv > .5).  compute ppv = 1-pv.  end if.  compute y5=sqrt(-2*ln(ppv)).  compute xp=y5+((((y5*p4+p3)*y5+p2)*y5+p1)*y5+p0)/((((y5*q4+q3)*y5+q2)*y5+q1)*y5+q0).  do if (pv <= .5).  compute xp = -xp.  end if.  compute cilow=rnd(nrow(temp)*(cdfnorm(2*xp+xp2))).  compute cihigh=trunc(nrow(temp)*(cdfnorm(2*xp+(-xp2))))+1.  do if (cilow < 1).  compute cilow = 1.  compute booterr=1.  compute badlo = 1.  end if.  do if (cihigh > nrow(temp)).  compute cihigh = boot.  compute booterr=1.  compute badhi = 1.  end if.  compute llcit=temp(cilow,1).  compute ulcit=temp(cihigh,1).  end if.  do if ( 9999 = 9999).  compute llcit=temp(cilow,1).  compute ulcit=temp(cihigh,1).  end if.  compute bootse=t(sqrt((cssq(temp)-((csum(temp)&**2)/nrow(temp)))/(nrow(temp)-1)))  .  compute indtemp={indtemp, bootse,llcit,ulcit}.  end if.  compute indtab={indtab;indtemp}.  end loop.  end loop.  end if.  release inddiff.  compute contlbs=cntname(1:(((nindfx)*(nindfx-1))/2),1).  compute rowlbs={rowlbs;contlbs}.  end if.  do if (kk=1).  print indtab/title = 'Indirect effect(s) of X on Y:'/rnames=rowlbs/cnames=bootlbs/format= F10.4.  end if.  do if (kk = 2).  print indtab/title = 'Partially standardized indirect effect(s) of X on Y:'/rnames=rowlbs/cnames=bootlbs/format= F10.4.  end if.  do if (kk = 3).  print indtab/title = 'Completely standardized indirect effect(s) of X on Y:'/rnames=rowlbs/cnames=bootlbs/format= F10.4.  end if.  do if (normal=1 and sobelok=1 and kk=1).  compute sobellab={'Effect',hclab,'Z','p'}.  compute sobelrlb=rowlbs.  do if (nms > 1).  compute sobelrlb=rowlbs(2:(1+nms),1).  end if.  print sobelmat/title=' Normal theory test for indirect effect(s):'/cnames=sobellab/rnames=sobelrlb/format= F10.4.  end if.  do if (contrast <> 0).  do if ((contrast=1 or contrast = 2) and kk=efloop ).  compute conkey=conkey(2:nrow(conkey),:).  print conkey/title = 'Specific indirect effect contrast definition(s):'/rnames=contlbs/format=A8.  end if.  do if (contrast = 3 and kk=efloop).  compute crowlbs=rowlbs(2:(nindfx+1),1).  print contvec/title = 'Specific indirect effect contrast weights:'/cnames=crowlbs/rlabels='(C1)'/format= F10.4.  end if.  do if (contrast = 2 and kk=efloop).  print/title = 'Contrasts are differences between absolute values of indirect effects'.  end if.  end if.  do if (serial = 1 and kk=efloop).  compute rowlbst=rowlbs(2:nrow(rowlbs),1).  print indkey/title = 'Indirect effect key:'/rnames=rowlbst/format = A8.  end if.  else.  do if (kk = 1).  print/title = 'Relative indirect effects of X on Y'.  end if.  do if (kk = 2).  print/title = 'Partially standardized relative indirect effect(s) of X on Y:'.  end if.  do if (kk = 3).  print/title = 'Completely standardized relative indirect effect(s) of X on Y:'.  end if.  loop i = 1 to nrow(indmake).  compute indtabsm=indtab((((i-1)*nxvls)+1):(nxvls*i),:).  compute indkeyt=indkey(i,:).  print indkeyt/title=' '/space=0/format=A8.  do if (bcmat(nrow(bcmat),1)=0)).  compute direffl2=xcatlab(1:nxvls,1).  end if.  print indtabsm/title = ' '/cnames=bootlbs/rnames=direffl2/format= F10.4 /space=0.  do if (normal=1 and sobelok=1 and kk=1).  compute sobelsm=sobelmat((((i-1)*nxvls)+1):(nxvls*i),:).  compute sobellab={'Effect',hclab,'Z','p'}.  print sobelsm/title=' Normal theory test for relative indirect effects:'/cnames=sobellab/rnames=direffl2/format= F10.4.  end if.  end loop.  end if.  do if (effsize = 1 and boot > 0).  compute bootres=bootres(2:nrow(bootres),:).  end if.  end loop.  end if.  do if (anymod > 0).  do if (boot = 0).  compute bootres=obscoeff.  compute indtab=999.  end if.  do if (boot > 0).  compute bootres={obscoeff;bootres}.  compute indtab=make(1,4,999).  end if.  do if (csum((indmod > 0))=nrow(indmod)).  do if (nxvls > 1).  print/title= 'Relative conditional indirect effects of X on Y:'.  end if.  do if (nxvls = 1).  print/title= 'Conditional indirect effects of X on Y:'.  end if.  end if.  do if (csum((indmod > 0)) < nrow(indmod)).  do if (nxvls > 1).  print/title= 'Relative conditional and unconditional indirect effects of X on Y:'.  end if.  do if (nxvls = 1).  print/title= 'Conditional and unconditional indirect effects of X on Y:'.  end if.  end if.  loop i = 1 to nrow(indmake).  compute indtab=indtab(1,:)*0.  print indkey(i,:)/title = 'INDIRECT EFFECT:'/format=A8.  do if (indmod(i,1)=0).  loop j = 1 to nxvls.  compute indtemp=make(nrow(bootres),1,1).  loop k = 1 to indmake(i,1).  compute jtemp=1.  do if (j > 1 and k=1).  compute jtemp=j.  end if.  compute indtemp=indtemp&*bootres(:,pathsfoc(jtemp,indmake(i,(k+1)))).  end loop.  compute indeff=indtemp(1,1).  do if (boot > 0).  .  compute temp = indtemp(2:nrow(indtemp),1).  compute temp(GRADE( indtemp(2:nrow(indtemp),1) )) = indtemp(2:nrow(indtemp),1).  compute badlo = 0.  compute badhi = 0.  do if ( 9999 <> 9999).  compute pv=csum(temp < 9999 )/nrow(temp).  compute ppv = pv.  do if (pv > .5).  compute ppv = 1-pv.  end if.  compute y5=sqrt(-2*ln(ppv)).  compute xp=y5+((((y5*p4+p3)*y5+p2)*y5+p1)*y5+p0)/((((y5*q4+q3)*y5+q2)*y5+q1)*y5+q0).  do if (pv <= .5).  compute xp = -xp.  end if.  compute cilow=rnd(nrow(temp)*(cdfnorm(2*xp+xp2))).  compute cihigh=trunc(nrow(temp)*(cdfnorm(2*xp+(-xp2))))+1.  do if (cilow < 1).  compute cilow = 1.  compute booterr=1.  compute badlo = 1.  end if.  do if (cihigh > nrow(temp)).  compute cihigh = boot.  compute booterr=1.  compute badhi = 1.  end if.  compute llcit=temp(cilow,1).  compute ulcit=temp(cihigh,1).  end if.  do if ( 9999 = 9999).  compute llcit=temp(cilow,1).  compute ulcit=temp(cihigh,1).  end if.  compute bootse=t(sqrt((cssq(temp)-((csum(temp)&**2)/nrow(temp)))/(nrow(temp)-1)))  .  compute indeff={indeff,bootse,llcit,ulcit}.  end if.  compute indtab={indtab;indeff}.  end loop.  do if (nxvls > 1).  compute indefflb=xcatlab(1:nxvls,1).  print indtab(2:nrow(indtab),:)/title = ' '/clabels='Effect', 'BootSE', 'BootLLCI', 'BootULCI'/rnames=indefflb/format= F10.4 /space=0.  end if.  do if (nxvls = 1).  print indtab(2:nrow(indtab),:)/title = ' '/clabels='Effect', 'BootSE', 'BootLLCI', 'BootULCI'/format= F10.4 /space=0.  end if.  end if.  do if (indmod(i,1)>0).  do if (indmod(i,1)=1).  compute indmodva=wmodvals.  compute indprova=wprobval.  compute condlbs={wnames}.  compute printw=1.  else if (indmod(i,1)=2).  compute indmodva=zmodvals.  compute indprova=zprobval.  compute condlbs={znames}.  compute printz=1.  else if (indmod(i,1)=3).  compute cntmp=1.  compute printz=1.  compute printw=1.  compute indmodva=make((nrow(wmodvals)*nrow(zmodvals)),2,999).  loop k7 = 1 to nrow(wmodvals).  loop k8 = 1 to nrow(zmodvals).  compute indmodva(cntmp,:)={wmodvals(k7,1),zmodvals(k8,1)}.  compute cntmp=cntmp+1.  end loop.  end loop.  compute condlbs={wnames,znames}.  end if.  compute condres=make(nrow(indmodva),1,999).  do if (boot > 0).  compute condres=make(nrow(indmodva),4,999).  end if.  compute condres={indmodva,condres}.  loop k4 = 1 to nxvls.  compute imm3=make(nrow(bootres),1,1).  compute imm4=make(nrow(bootres),1,1).  compute indcontr=0.  do if (indmod(i,1)=3).  compute tihsw=wprobval.  compute tihsz=zprobval.  end if.  loop k1=1 to nrow(indmodva).  compute tucker2=make(nrow(bootres),1,1).  compute imm2=make(nrow(bootres),1,1).  compute wfirst=0.  compute zfirst=0.  compute immset=0.  loop k2=1 to indmake(i,1).  compute colnumb=indmake(i,(k2+1)).  do if (k2=1).  compute wbb=make(nrow(bootres),(nwvls*nxvls),0).  compute zbb=make(nrow(bootres),(nzvls*nxvls),0).  compute wzbb=make(nrow(bootres),(nwvls*nzvls*nxvls),0).  end if.  do if (k2<>1).  compute wbb=make(nrow(bootres),nwvls,0).  compute zbb=make(nrow(bootres),nzvls,0).  compute wzbb=make(nrow(bootres),(nwvls*nzvls),0).  end if.  compute cnt=1.  compute tihs=indlocs(2:((indlocs(1,colnumb))+1),colnumb).  do if (k2 = 1).  compute focbb=tihs(1:nxvls,1).  compute focbb=bootres(:,focbb).  do if (indmmm(i,1)>0).  compute imm=focbb(:,k4).  compute condbb=imm.  end if.  compute focaddon=make(1,nxvls,0).  compute focaddon(1,k4)=1.  compute cnt=cnt+nxvls.  compute placeh=nxvls.  do if (indmod(i,1)=1).  compute tihsz=make(nrow(wprobval),(nzvls*nxvls),0).  compute tihswz=make(nrow(wprobval),(nwvls*nzvls*nxvls),0).  do if (pathsw(1,colnumb)=1).  compute temp=make(nrow(wprobval),(nxvls*nwvls),0).  loop k5 = 1 to nrow(wprobval).  loop k6=1 to nwvls.  compute temp(k5, (((k4-1)*nwvls)+k6))=wprobval(k5,k6).  end loop.  end loop.  compute indprova={temp,tihsz,tihswz}.  else.  compute indprova={wprobval,tihsz,tihswz}.  end if.  end if.  do if (indmod(i,1)=2).  compute tihsw=make(nrow(zprobval),(nwvls*nxvls),0).  compute tihswz=make(nrow(zprobval),(nwvls*nzvls*nxvls),0).  do if (pathsz(1,colnumb)=1).  compute temp=make(nrow(zprobval),(nxvls*nzvls),0).  loop k5 = 1 to nrow(zprobval).  loop k6 =1 to nzvls.  compute temp(k5,(((k4-1)*nzvls)+k6))=zprobval(k5,k6).  end loop.  end loop.  compute indprova={tihsw,temp,tihswz}.  else.  compute indprova={tihsw,zprobval,tihswz}.  end if.  end if.  do if (indmod(i,1)=3).  compute indprova=make((nrow(wprobval)*nrow(zprobval)),((ncol(wprobval)*nxvls)+ (ncol(zprobval)*nxvls)+(nwvls*nzvls*nxvls)),0).  compute cntemp=1.  loop k7=1 to nrow(wprobval).  loop k8 =1 to nrow(zprobval).  compute temp=wprobval(k7,:)*focaddon(1,k4).  compute indprova(cntemp,(((k4-1)*nwvls)+1):(k4*(nwvls)))=temp.  compute temp=zprobval(k8,:)*focaddon(1,k4).  compute indprova(cntemp, ((((k4-1)*nzvls)+1)+(nxvls*nwvls)) : ((((k4-1)*nzvls)+1)+(nxvls*nwvls)+(nzvls-1)))=temp.  compute cntemp=cntemp+1.  end loop.  end loop.  do if (pathsz(1,colnumb)=0).  compute temp=make(nrow(indprova),(ncol(zprobval)*nxvls),0).  compute indprova(:,((ncol(wprobval)*nxvls)+1):((ncol(wprobval)+ncol(zprobval))* nxvls))=temp.  end if.  do if (pathsw(1,colnumb)=0).  compute temp=make(nrow(indprova),(ncol(wprobval)*nxvls),0).  compute indprova(:,1:(ncol(wprobval)*nxvls))=temp.  end if.  do if (pathswz(1,colnumb)=1).  compute cntemp=(ncol(wprobval)*nxvls)+(ncol(zprobval)*nxvls)+((k4-1)* ncol(wprobval)*ncol(zprobval))+1.  loop k7=1 to ncol(wprobval).  loop k8=1 to ncol(zprobval).  compute indprova(:,cntemp)=indprova(:,((ncol(wprobval)*(k4-1))+k7))&* indprova(:,((((k4-1)*ncol(zprobval))+k8)+(nxvls*ncol(wprobval)))).  compute cntemp=cntemp+1.  end loop.  end loop.  end if.  end if.  end if.  do if (k2 > 1).  compute focbb=tihs(1,1).  compute focbb=bootres(:,focbb).  do if (indmmm(i,1)>0).  compute imm=focbb(:,1).  compute condbb=imm.  end if.  compute focaddon=1.  compute cnt=cnt+1.  compute placeh=1.  do if (indmod(i,1)=1).  compute tihsz=make(nrow(wprobval),nzvls,0).  compute tihswz=make(nrow(wprobval),(nwvls*nzvls),0).  compute indprova={wprobval,tihsz,tihswz}.  end if.  do if (indmod(i,1)=2).  compute tihsw=make(nrow(zprobval),nwvls,0).  compute tihswz=make(nrow(zprobval),(nwvls*nzvls),0).  compute indprova={tihsw,zprobval,tihswz}.  end if.  do if (indmod(i,1)=3).  compute indprova=make((nrow(wprobval)*nrow(zprobval)),((ncol(wprobval)+ ncol(zprobval))+(nwvls*nzvls)),0).  compute cntemp=1.  loop k7=1 to nrow(wprobval).  loop k8 =1 to nrow(zprobval).  compute indprova(cntemp,1:(ncol(wprobval)+ncol(zprobval)))={wprobval(k7,:), zprobval(k8,:)}.  compute cntemp=cntemp+1.  end loop.  end loop.  do if (pathsz(1,colnumb)=0).  compute temp=make(nrow(indprova),ncol(zprobval),0).  compute indprova(:,(ncol(wprobval)+1):(ncol(wprobval)+ncol(zprobval)))=temp.  end if.  do if (pathsw(1,colnumb)=0).  compute temp=make(nrow(indprova),ncol(wprobval),0).  compute indprova(:,1:ncol(wprobval))=temp.  end if.  do if (pathswz(1,colnumb)=1).  compute cntemp=ncol(wprobval)+ncol(zprobval)+1.  loop k7=1 to ncol(wprobval).  loop k8=1 to ncol(zprobval).  compute indprova(:,cntemp)=indprova(:,k7)&*indprova(:,(ncol(wprobval)+k8)).  compute cntemp=cntemp+1.  end loop.  end loop.  end if.  end if.  end if.  do if (pathsw(1,colnumb)) = 1.  compute wbb=tihs(cnt:(cnt+(placeh*nwvls)-1),1).  compute wbb=bootres(:,wbb).  compute immlbs2=wcatlab(1:nwvls,1).  do if (zfirst=0).  compute wfirst=1.  end if.  do if (indmmm(i,1)=1 or indmmm(i,1)=31 or indmmm(i,1)=51).  compute imm=wbb(:,1).  loop k7 = 1 to nwvls.  compute imm={imm,wbb(:,(((k4-1)*nwvls*(k2=1))+k7))}.  end loop.  compute imm=imm(:,2:ncol(imm)).  end if.  do if (indmmm(i,1)=41 or indmmm(i,1)=51).  compute condbb=make(nrow(bootres),1,0).  loop k7 = 1 to nwvls.  compute condbb={condbb,wbb(:,(((k4-1)*nwvls*(k2=1))+k7))}.  end loop.  compute condbb=condbb(:,2:ncol(condbb)).  end if.  compute cnt=cnt+(placeh*nwvls).  end if.  do if (pathsz(1,colnumb)) = 1.  compute zbb=tihs(cnt:(cnt+(placeh*nzvls)-1),1).  compute zbb=bootres(:,zbb).  do if (wfirst=0).  compute zfirst=1.  end if.  do if (indmmm(i,1) <> 31).  compute immlbs2=zcatlab(1:nzvls,1).  end if.  do if (indmmm(i,1)=2 or indmmm(i,1)=31 or indmmm(i,1)=51).  do if (indmmm(i,1)=2).  compute imm=zbb(:,1).  end if.  loop k7 = 1 to nzvls.  compute imm={imm,zbb(:,(((k4-1)*nzvls*(k2=1))+k7))}.  end loop.  do if (indmmm(i,1)=2 or indmmm(i,1)=51).  compute imm=imm(:,2:ncol(imm)).  do if (indmmm(i,1)=51).  compute condbb={condbb,imm}.  end if.  end if.  end if.  compute cnt=cnt+(placeh*nzvls).  end if.  do if (pathswz(1,colnumb)) = 1.  compute wzbb=tihs(cnt:(cnt+(placeh*nwvls*nzvls)-1),1).  compute wzbb=bootres(:,wzbb).  do if (indmmm(i,1)=41).  compute imm=wzbb(:,1).  loop k7=1 to nwvls*nzvls.  compute imm={imm,wzbb(:,(((k4-1)*nzvls*nwvls*(k2=1))+k7))}.  end loop.  end if.  do if (indmmm(i,1)=41).  compute imm=imm(:,2:ncol(imm)).  compute condbb={condbb,imm(:,(ncol(imm)-(nwvls*nzvls)+1):ncol(imm))}.  end if.  compute cnt=cnt+(placeh*nzvls*nwvls).  end if.  compute indprobe={focaddon,indprova(k1,:)}.  compute tucker={focbb,wbb,zbb,wzbb}.  loop k3=1 to ncol(indprobe).  compute tucker(:,k3)=tucker(:,k3)*indprobe(1,k3).  end loop.  compute tucker2=tucker2&*rsum(tucker).  do if (indmmm(i,1) = 1 or indmmm(i,1)=2 or indmmm(i,1)=31 or indmmm(i,1)=41 or indmmm(i,1)=51).  do if (immset=1).  do if (ncol(imm2)=1 and ncol(imm) = 1).  compute imm2=imm2&*imm.  end if.  do if (indmmm(i,1)=41 or indmmm(i,1)=51).  do if ((ncol(condbb2) > 1) and (ncol(condbb)>1)).  compute condbb2t=make(nrow(condbb2),(ncol(condbb2)*ncol(condbb)),-999999).  compute k9=1.  do if (wfirst=1).  loop k7=1 to ncol(condbb2).  loop k8 = 1 to ncol(condbb).  compute condbb2t(:,k9)=condbb2(:,k7)&*condbb(:,k8).  compute k9=k9+1.  end loop.  end loop.  end if.  do if (zfirst=1).  loop k7=1 to ncol(condbb).  loop k8 = 1 to ncol(condbb2).  compute condbb2t(:,k9)=condbb(:,k7)&*condbb2(:,k8).  compute k9=k9+1.  end loop.  end loop.  end if.  compute condbb2=condbb2t.  release condbb2t.  end if.  do if ((ncol(condbb2) > 1) and (ncol(condbb)=1)).  loop k7 = 1 to ncol(condbb2).  compute condbb2(:,k7)=condbb2(:,k7)&*condbb.  end loop.  end if.  do if ((ncol(condbb2) = 1) and (ncol(condbb)>1)).  loop k7 = 1 to ncol(condbb).  compute condbb(:,k7)=condbb2&*condbb(:,k7).  end loop.  compute condbb2=condbb.  end if.  end if.  do if (ncol(imm2) <> 1 and ncol(imm) <> 1).  compute imm2t=make(nrow(imm2),(ncol(imm2)*ncol(imm)),-999999).  compute k9=1.  do if (wfirst=1).  loop k7=1 to ncol(imm2).  loop k8 = 1 to ncol(imm).  compute imm2t(:,k9)=imm2(:,k7)&*imm(:,k8).  compute k9=k9+1.  end loop.  end loop.  end if.  do if (zfirst=1).  loop k7=1 to ncol(imm).  loop k8 = 1 to ncol(imm2).  compute imm2t(:,k9)=imm(:,k7)&*imm2(:,k8).  compute k9=k9+1.  end loop.  end loop.  end if.  compute imm2=imm2t.  release imm2t.  end if.  do if ((ncol(imm2) > 1) and (ncol(imm)=1)).  loop k7=1 to ncol(imm2).  compute imm2(:,k7)=imm2(:,k7)&*imm.  end loop.  end if.  do if ((ncol(imm2) = 1) and (ncol(imm) > 1)).  loop k7=1 to ncol(imm).  compute imm(:,k7)=imm2&*imm(:,k7).  end loop.  compute imm2=imm.  end if.  end if.  do if (immset=0).  compute imm2=imm.  do if (indmmm(i,1)=41 or indmmm(i,1)=51).  compute condbb2=condbb.  end if.  compute immset=1.  end if.  end if.  end loop.  compute indtemp=tucker2(1,1).  do if (indmmm(i,1)=12 or indmmm(i,1)=22).  compute imm3={imm3,tucker2}.  do if (k1=nrow(indmodva)).  compute imm3=imm3(:,2:ncol(imm3)).  compute immstop=ncol(imm3).  loop k8=2 to immstop.  do if (indmmmt(i,1)=1).  compute imm3={imm3,(imm3(:,k8)-imm3(:,1))}.  end if.  do if (indmmmt(i,1)=2).  compute imm3={imm3,(imm3(:,k8)-imm3(:,(k8-1)))}.  end if.  do if (indmmmt(i,1)=3).  compute imm3={imm3,((rsum(imm3(:,(k8:immstop)))/(immstop-k8+1))-imm3(:,(k8-1)))}.  end if.  do if (indmmmt(i,1)=4).  compute imm3={imm3,(imm3(:,k8)-(rsum(imm3(:,1:immstop))/immstop))}.  end if.  end loop.  do if (indmmmt(i,1)<5).  compute imm2=imm3(:,(immstop+1):ncol(imm3)).  release imm3.  end if.  end if.  end if.  do if (indmmm(i,1)>-1 and (contrast = 1 or contrast = 2)).  compute imm4={imm4,tucker2}.  do if (k1=nrow(indmodva) and k1 > 1).  compute imm4=imm4(:,2:ncol(imm4)).  compute immstop=ncol(imm4).  compute condcont=make((immstop*(immstop-1)/2),6,-999).  loop k8 = 1 to (immstop-1).  loop k9 = (k8+1) to immstop.  do if (contrast=1).  compute imm4={imm4,(imm4(:,k9)-imm4(:,k8))}.  end if.  do if (contrast=2).  compute imm4={imm4,(abs(imm4(:,k9))-abs(imm4(:,k8)))}.  end if.  compute condcont((ncol(imm4)-immstop),1)=imm4(1,k9).  compute condcont((ncol(imm4)-immstop),2)=imm4(1,k8).  end loop.  end loop.  compute imm4=imm4(:,(immstop+1):ncol(imm4)).  loop k8=1 to ncol(imm4).  compute condcont(k8,3)=imm4(1,k8).  do if (boot > 0).  .  compute temp = imm4(2:nrow(imm4),k8).  compute temp(GRADE( imm4(2:nrow(imm4),k8) )) = imm4(2:nrow(imm4),k8).  compute badlo = 0.  compute badhi = 0.  do if ( 9999 <> 9999).  compute pv=csum(temp < 9999 )/nrow(temp).  compute ppv = pv.  do if (pv > .5).  compute ppv = 1-pv.  end if.  compute y5=sqrt(-2*ln(ppv)).  compute xp=y5+((((y5*p4+p3)*y5+p2)*y5+p1)*y5+p0)/((((y5*q4+q3)*y5+q2)*y5+q1)*y5+q0).  do if (pv <= .5).  compute xp = -xp.  end if.  compute cilow=rnd(nrow(temp)*(cdfnorm(2*xp+xp2))).  compute cihigh=trunc(nrow(temp)*(cdfnorm(2*xp+(-xp2))))+1.  do if (cilow < 1).  compute cilow = 1.  compute booterr=1.  compute badlo = 1.  end if.  do if (cihigh > nrow(temp)).  compute cihigh = boot.  compute booterr=1.  compute badhi = 1.  end if.  compute llcit=temp(cilow,1).  compute ulcit=temp(cihigh,1).  end if.  do if ( 9999 = 9999).  compute llcit=temp(cilow,1).  compute ulcit=temp(cihigh,1).  end if.  compute bootse=t(sqrt((cssq(temp)-((csum(temp)&**2)/nrow(temp)))/(nrow(temp)-1)))  .  compute condcont(k8,4:6)={bootse,llcit,ulcit}.  end if.  end loop.  do if (boot=0).  compute condcont=condcont(:,1:3).  end if.  compute indcontr=1.  end if.  end if.  do if (boot > 0).  .  compute temp = tucker2(2:nrow(tucker2),1).  compute temp(GRADE( tucker2(2:nrow(tucker2),1) )) = tucker2(2:nrow(tucker2),1).  compute badlo = 0.  compute badhi = 0.  do if ( 9999 <> 9999).  compute pv=csum(temp < 9999 )/nrow(temp).  compute ppv = pv.  do if (pv > .5).  compute ppv = 1-pv.  end if.  compute y5=sqrt(-2*ln(ppv)).  compute xp=y5+((((y5*p4+p3)*y5+p2)*y5+p1)*y5+p0)/((((y5*q4+q3)*y5+q2)*y5+q1)*y5+q0).  do if (pv <= .5).  compute xp = -xp.  end if.  compute cilow=rnd(nrow(temp)*(cdfnorm(2*xp+xp2))).  compute cihigh=trunc(nrow(temp)*(cdfnorm(2*xp+(-xp2))))+1.  do if (cilow < 1).  compute cilow = 1.  compute booterr=1.  compute badlo = 1.  end if.  do if (cihigh > nrow(temp)).  compute cihigh = boot.  compute booterr=1.  compute badhi = 1.  end if.  compute llcit=temp(cilow,1).  compute ulcit=temp(cihigh,1).  end if.  do if ( 9999 = 9999).  compute llcit=temp(cilow,1).  compute ulcit=temp(cihigh,1).  end if.  compute bootse=t(sqrt((cssq(temp)-((csum(temp)&**2)/nrow(temp)))/(nrow(temp)-1)))  .  compute indtemp={indtemp, bootse,llcit,ulcit}.  end if.  compute condres(k1,(ncol(indmodva)+1):ncol(condres))=indtemp.  end loop.  compute condlbs={condlbs,'Effect'}.  do if (boot > 0).  compute condlbs={condlbs,'BootSE', 'BootLLCI', 'BootULCI'}.  end if.  do if (nxvls=1).  print condres/title=' '/cnames=condlbs/format= F10.4 /space=0.  else.  compute condrlb=make(nrow(condres),1,xcatlab(k4,1)).  print condres/title=' '/cnames=condlbs/rnames=condrlb/format= F10.4 /space=0.  end if.  compute dichadj=0.  compute immcat=0.  do if (indmmm(i,1)>0).  do if (indmmm(i,1)=1 or indmmm(i,1)=12 or indmmm(i,1)=31).  do if (wdich=1 and mcw=0).  do if (indmmm(i,1) <> 12).  compute imm2(:,1)=imm2(:,1)*(wmax-wmin).  end if.  do if (indmmm(i,1) <> 31).  compute dichadj=1.  end if.  end if.  do if ((mcw = 1 or mcw = 2) and indmmm(i,1) <> 31)).  compute immcat=1.  end if.  end if.  do if (indmmm(i,1)=2 or indmmm(i,1)=22 or indmmm(i,1)=31).  do if (zdich=1 and mcz=0).  do if (indmmm(i,1) = 31).  compute imm2(:,(nwvls+1):ncol(imm2))=imm2(:,(nwvls+1):ncol(imm2))*(zmax-zmin).  end if.  do if (indmmm(i,1) = 2).  compute imm2(:,1)=imm2(:,1)*(zmax-zmin).  end if.  do if (indmmm(i,1) <> 31).  compute dichadj=1.  end if.  end if.  do if ((mcz = 1 or mcz = 2) and indmmm(i,1) <> 31)).  compute immcat=1.  end if.  end if.  compute immtemp2=t(imm2(1,:)).  compute immtemp=immtemp2.  compute immlbs={'Index'}.  do if (boot > 0).  compute immtemp=make(1,3,0).  loop k7=1 to ncol(imm2).  .  compute temp = imm2(2:nrow(imm2),k7).  compute temp(GRADE( imm2(2:nrow(imm2),k7) )) = imm2(2:nrow(imm2),k7).  compute badlo = 0.  compute badhi = 0.  do if ( 9999 <> 9999).  compute pv=csum(temp < 9999 )/nrow(temp).  compute ppv = pv.  do if (pv > .5).  compute ppv = 1-pv.  end if.  compute y5=sqrt(-2*ln(ppv)).  compute xp=y5+((((y5*p4+p3)*y5+p2)*y5+p1)*y5+p0)/((((y5*q4+q3)*y5+q2)*y5+q1)*y5+q0).  do if (pv <= .5).  compute xp = -xp.  end if.  compute cilow=rnd(nrow(temp)*(cdfnorm(2*xp+xp2))).  compute cihigh=trunc(nrow(temp)*(cdfnorm(2*xp+(-xp2))))+1.  do if (cilow < 1).  compute cilow = 1.  compute booterr=1.  compute badlo = 1.  end if.  do if (cihigh > nrow(temp)).  compute cihigh = boot.  compute booterr=1.  compute badhi = 1.  end if.  compute llcit=temp(cilow,1).  compute ulcit=temp(cihigh,1).  end if.  do if ( 9999 = 9999).  compute llcit=temp(cilow,1).  compute ulcit=temp(cihigh,1).  end if.  compute bootse=t(sqrt((cssq(temp)-((csum(temp)&**2)/nrow(temp)))/(nrow(temp)-1)))  .  compute temp={bootse,llcit,ulcit}.  compute immtemp={immtemp;temp}.  end loop.  compute immtemp=immtemp(2:nrow(immtemp),:).  compute immtemp={immtemp2,immtemp}.  compute immlbs={immlbs,'BootSE', 'BootLLCI', 'BootULCI'}.  end if.  do if (dichadj=0 and immcat=0 and indmmmt(i,1)<>5 and indmmm(i,1) < 100 ).  do if (indmmm(i,1) < 30).  print immtemp/title=' Index of moderated mediation:'/cnames=immlbs/rnames=immlbs2/format= F10.4.  end if.  do if (indmmm(i,1) = 31).  compute immlbs2={immlbs2;zcatlab(1:nzvls,1)}.  print immtemp/title=' Indices of partial moderated mediation:'/cnames=immlbs/rnames=immlbs2/format= F10.4.  end if.  do if ((nzvls = 1) and (nwvls = 1)).  do if (indmmm(i,1) = 41 or indmmm(i,1)=51).  loop k7=1 to nwvls.  compute immlbs2=zcatlab(1:nzvls,1).  compute immtemp2=immtemp((((k7-1)*nzvls)+1):(((k7-1)*nzvls)+nzvls),:).  do if (nwvls > 1).  compute primodv={' ', wcatlab(k7,1)}.  print primodv/title=' Primary moderator:'/format=A8.  end if.  do if (nzvls=1).  print immtemp2/title=' Index of moderated moderated mediation'/cnames=immlbs/format= F10.4.  else.  print immtemp2/title=' Indices of moderated moderated mediation'/cnames=immlbs/rnames=immlbs2/format= F10.4.  end if.  compute cmmtemp=make(nrow(zprobval),4,0).  loop k8=1 to nrow(zprobval).  compute condbb3=condbb2(:,((nwvls+1)+((k7-1)*nzvls)):((nwvls+1)+((k7-1)*nzvls)+ (nzvls-1))).  do if (ncol(zprobval) > 1).  compute condbb3=condbb3*mdiag(zprobval(k8,:)).  else.  compute condbb3=condbb3&*zprobval(k8,:).  end if.  compute condbb3={condbb2(:,k7),condbb3}.  compute icmm=rsum(condbb3).  compute cmmtemp(k8,1)=icmm(1,1).  do if (boot > 0).  .  compute temp = icmm(2:nrow(icmm),1).  compute temp(GRADE( icmm(2:nrow(icmm),1) )) = icmm(2:nrow(icmm),1).  compute badlo = 0.  compute badhi = 0.  do if ( 9999 <> 9999).  compute pv=csum(temp < 9999 )/nrow(temp).  compute ppv = pv.  do if (pv > .5).  compute ppv = 1-pv.  end if.  compute y5=sqrt(-2*ln(ppv)).  compute xp=y5+((((y5*p4+p3)*y5+p2)*y5+p1)*y5+p0)/((((y5*q4+q3)*y5+q2)*y5+q1)*y5+q0).  do if (pv <= .5).  compute xp = -xp.  end if.  compute cilow=rnd(nrow(temp)*(cdfnorm(2*xp+xp2))).  compute cihigh=trunc(nrow(temp)*(cdfnorm(2*xp+(-xp2))))+1.  do if (cilow < 1).  compute cilow = 1.  compute booterr=1.  compute badlo = 1.  end if.  do if (cihigh > nrow(temp)).  compute cihigh = boot.  compute booterr=1.  compute badhi = 1.  end if.  compute llcit=temp(cilow,1).  compute ulcit=temp(cihigh,1).  end if.  do if ( 9999 = 9999).  compute llcit=temp(cilow,1).  compute ulcit=temp(cihigh,1).  end if.  compute bootse=t(sqrt((cssq(temp)-((csum(temp)&**2)/nrow(temp)))/(nrow(temp)-1)))  .  compute cmmtemp(k8,2:4)={bootse,llcit,ulcit}.  end if.  end loop.  compute cmmtemp={zmodvals,cmmtemp}.  do if (boot=0).  compute cmmtemp=cmmtemp(:,1:2).  end if.  compute cmmlbs={znames,immlbs}.  print cmmtemp/title=' Indices of conditional moderated mediation by W'/cnames=cmmlbs/format= F10.4.  end loop.  end if.  end if.  end if.  do if (dichadj=1 or immcat=1 and indmmm(i,1) < 30).  print immtemp/title='Index of moderated mediation (difference between conditional indirect effects):'/cnames=immlbs/rnames=immlbs2/format= F10.4.  end if.  end if.  do if (indcontr=1).  compute condctlb={'Effect1','Effect2','Contrast','BootSE', 'BootLLCI','BootULCI'}.  print condcont/title=' Pairwise contrasts between conditional indirect effects (Effect1 minus Effect2)'/cnames=condctlb/format= F10.4.  end if.  print/title= '---'/space=0.  end loop.  end if.  end loop.  end if.  end if  .  do if (criterr=0 and (saveest = 1 or saveboot=1)).  compute coeffsav=coeffmat(2:nrow(coeffmat),:).  compute conseq=conseq(2:nrow(conseq),1).  compute dfmat=dfmat(2:nrow(dfmat),1).  compute dfmat=dfmat.  compute saven={'conseqnt','antecdnt','coeff','se','t','p','LLCI','ULCI','df'}.  do if (ydich=1).  compute saven={'conseqnt','antecdnt','coeff','se','t_or_Z','p','LLCI','ULCI','df'}.  end if.  do if (saveest=1).  save {conseq,vlabs,coeffsav,dfmat}/outfile = */names=saven/strings=conseqnt antecdnt.  end if.  do if (saveboot=1 and boot > 0 ).  print/title = '**************************************************************************'.  print/title='Bootstrap estimates were saved to a file'/space=0.  compute colslab=make(300,1,' ').  compute colslab( 1 ,1)= 'COL1'.  compute colslab( 2 ,1)= 'COL2'.  compute colslab( 3 ,1)= 'COL3'.  compute colslab( 4 ,1)= 'COL4'.  compute colslab( 5 ,1)= 'COL5'.  compute colslab( 6 ,1)= 'COL6'.  compute colslab( 7 ,1)= 'COL7'.  compute colslab( 8 ,1)= 'COL8'.  compute colslab( 9 ,1)= 'COL9'.  compute colslab( 10 ,1)= 'COL10'.  compute colslab( 11 ,1)= 'COL11'.  compute colslab( 12 ,1)= 'COL12'.  compute colslab( 13 ,1)= 'COL13'.  compute colslab( 14 ,1)= 'COL14'.  compute colslab( 15 ,1)= 'COL15'.  compute colslab( 16 ,1)= 'COL16'.  compute colslab( 17 ,1)= 'COL17'.  compute colslab( 18 ,1)= 'COL18'.  compute colslab( 19 ,1)= 'COL19'.  compute colslab( 20 ,1)= 'COL20'.  compute colslab( 21 ,1)= 'COL21'.  compute colslab( 22 ,1)= 'COL22'.  compute colslab( 23 ,1)= 'COL23'.  compute colslab( 24 ,1)= 'COL24'.  compute colslab( 25 ,1)= 'COL25'.  compute colslab( 26 ,1)= 'COL26'.  compute colslab( 27 ,1)= 'COL27'.  compute colslab( 28 ,1)= 'COL28'.  compute colslab( 29 ,1)= 'COL29'.  compute colslab( 30 ,1)= 'COL30'.  compute colslab( 31 ,1)= 'COL31'.  compute colslab( 32 ,1)= 'COL32'.  compute colslab( 33 ,1)= 'COL33'.  compute colslab( 34 ,1)= 'COL34'.  compute colslab( 35 ,1)= 'COL35'.  compute colslab( 36 ,1)= 'COL36'.  compute colslab( 37 ,1)= 'COL37'.  compute colslab( 38 ,1)= 'COL38'.  compute colslab( 39 ,1)= 'COL39'.  compute colslab( 40 ,1)= 'COL40'.  compute colslab( 41 ,1)= 'COL41'.  compute colslab( 42 ,1)= 'COL42'.  compute colslab( 43 ,1)= 'COL43'.  compute colslab( 44 ,1)= 'COL44'.  compute colslab( 45 ,1)= 'COL45'.  compute colslab( 46 ,1)= 'COL46'.  compute colslab( 47 ,1)= 'COL47'.  compute colslab( 48 ,1)= 'COL48'.  compute colslab( 49 ,1)= 'COL49'.  compute colslab( 50 ,1)= 'COL50'.  compute colslab( 51 ,1)= 'COL51'.  compute colslab( 52 ,1)= 'COL52'.  compute colslab( 53 ,1)= 'COL53'.  compute colslab( 54 ,1)= 'COL54'.  compute colslab( 55 ,1)= 'COL55'.  compute colslab( 56 ,1)= 'COL56'.  compute colslab( 57 ,1)= 'COL57'.  compute colslab( 58 ,1)= 'COL58'.  compute colslab( 59 ,1)= 'COL59'.  compute colslab( 60 ,1)= 'COL60'.  compute colslab( 61 ,1)= 'COL61'.  compute colslab( 62 ,1)= 'COL62'.  compute colslab( 63 ,1)= 'COL63'.  compute colslab( 64 ,1)= 'COL64'.  compute colslab( 65 ,1)= 'COL65'.  compute colslab( 66 ,1)= 'COL66'.  compute colslab( 67 ,1)= 'COL67'.  compute colslab( 68 ,1)= 'COL68'.  compute colslab( 69 ,1)= 'COL69'.  compute colslab( 70 ,1)= 'COL70'.  compute colslab( 71 ,1)= 'COL71'.  compute colslab( 72 ,1)= 'COL72'.  compute colslab( 73 ,1)= 'COL73'.  compute colslab( 74 ,1)= 'COL74'.  compute colslab( 75 ,1)= 'COL75'.  compute colslab( 76 ,1)= 'COL76'.  compute colslab( 77 ,1)= 'COL77'.  compute colslab( 78 ,1)= 'COL78'.  compute colslab( 79 ,1)= 'COL79'.  compute colslab( 80 ,1)= 'COL80'.  compute colslab( 81 ,1)= 'COL81'.  compute colslab( 82 ,1)= 'COL82'.  compute colslab( 83 ,1)= 'COL83'.  compute colslab( 84 ,1)= 'COL84'.  compute colslab( 85 ,1)= 'COL85'.  compute colslab( 86 ,1)= 'COL86'.  compute colslab( 87 ,1)= 'COL87'.  compute colslab( 88 ,1)= 'COL88'.  compute colslab( 89 ,1)= 'COL89'.  compute colslab( 90 ,1)= 'COL90'.  compute colslab( 91 ,1)= 'COL91'.  compute colslab( 92 ,1)= 'COL92'.  compute colslab( 93 ,1)= 'COL93'.  compute colslab( 94 ,1)= 'COL94'.  compute colslab( 95 ,1)= 'COL95'.  compute colslab( 96 ,1)= 'COL96'.  compute colslab( 97 ,1)= 'COL97'.  compute colslab( 98 ,1)= 'COL98'.  compute colslab( 99 ,1)= 'COL99'.  compute colslab( 100 ,1)= 'COL100'.  compute colslab( 101 ,1)= 'COL101'.  compute colslab( 102 ,1)= 'COL102'.  compute colslab( 103 ,1)= 'COL103'.  compute colslab( 104 ,1)= 'COL104'.  compute colslab( 105 ,1)= 'COL105'.  compute colslab( 106 ,1)= 'COL106'.  compute colslab( 107 ,1)= 'COL107'.  compute colslab( 108 ,1)= 'COL108'.  compute colslab( 109 ,1)= 'COL109'.  compute colslab( 110 ,1)= 'COL110'.  compute colslab( 111 ,1)= 'COL111'.  compute colslab( 112 ,1)= 'COL112'.  compute colslab( 113 ,1)= 'COL113'.  compute colslab( 114 ,1)= 'COL114'.  compute colslab( 115 ,1)= 'COL115'.  compute colslab( 116 ,1)= 'COL116'.  compute colslab( 117 ,1)= 'COL117'.  compute colslab( 118 ,1)= 'COL118'.  compute colslab( 119 ,1)= 'COL119'.  compute colslab( 120 ,1)= 'COL120'.  compute colslab( 121 ,1)= 'COL121'.  compute colslab( 122 ,1)= 'COL122'.  compute colslab( 123 ,1)= 'COL123'.  compute colslab( 124 ,1)= 'COL124'.  compute colslab( 125 ,1)= 'COL125'.  compute colslab( 126 ,1)= 'COL126'.  compute colslab( 127 ,1)= 'COL127'.  compute colslab( 128 ,1)= 'COL128'.  compute colslab( 129 ,1)= 'COL129'.  compute colslab( 130 ,1)= 'COL130'.  compute colslab( 131 ,1)= 'COL131'.  compute colslab( 132 ,1)= 'COL132'.  compute colslab( 133 ,1)= 'COL133'.  compute colslab( 134 ,1)= 'COL134'.  compute colslab( 135 ,1)= 'COL135'.  compute colslab( 136 ,1)= 'COL136'.  compute colslab( 137 ,1)= 'COL137'.  compute colslab( 138 ,1)= 'COL138'.  compute colslab( 139 ,1)= 'COL139'.  compute colslab( 140 ,1)= 'COL140'.  compute colslab( 141 ,1)= 'COL141'.  compute colslab( 142 ,1)= 'COL142'.  compute colslab( 143 ,1)= 'COL143'.  compute colslab( 144 ,1)= 'COL144'.  compute colslab( 145 ,1)= 'COL145'.  compute colslab( 146 ,1)= 'COL146'.  compute colslab( 147 ,1)= 'COL147'.  compute colslab( 148 ,1)= 'COL148'.  compute colslab( 149 ,1)= 'COL149'.  compute colslab( 150 ,1)= 'COL150'.  compute colslab( 151 ,1)= 'COL151'.  compute colslab( 152 ,1)= 'COL152'.  compute colslab( 153 ,1)= 'COL153'.  compute colslab( 154 ,1)= 'COL154'.  compute colslab( 155 ,1)= 'COL155'.  compute colslab( 156 ,1)= 'COL156'.  compute colslab( 157 ,1)= 'COL157'.  compute colslab( 158 ,1)= 'COL158'.  compute colslab( 159 ,1)= 'COL159'.  compute colslab( 160 ,1)= 'COL160'.  compute colslab( 161 ,1)= 'COL161'.  compute colslab( 162 ,1)= 'COL162'.  compute colslab( 163 ,1)= 'COL163'.  compute colslab( 164 ,1)= 'COL164'.  compute colslab( 165 ,1)= 'COL165'.  compute colslab( 166 ,1)= 'COL166'.  compute colslab( 167 ,1)= 'COL167'.  compute colslab( 168 ,1)= 'COL168'.  compute colslab( 169 ,1)= 'COL169'.  compute colslab( 170 ,1)= 'COL170'.  compute colslab( 171 ,1)= 'COL171'.  compute colslab( 172 ,1)= 'COL172'.  compute colslab( 173 ,1)= 'COL173'.  compute colslab( 174 ,1)= 'COL174'.  compute colslab( 175 ,1)= 'COL175'.  compute colslab( 176 ,1)= 'COL176'.  compute colslab( 177 ,1)= 'COL177'.  compute colslab( 178 ,1)= 'COL178'.  compute colslab( 179 ,1)= 'COL179'.  compute colslab( 180 ,1)= 'COL180'.  compute colslab( 181 ,1)= 'COL181'.  compute colslab( 182 ,1)= 'COL182'.  compute colslab( 183 ,1)= 'COL183'.  compute colslab( 184 ,1)= 'COL184'.  compute colslab( 185 ,1)= 'COL185'.  compute colslab( 186 ,1)= 'COL186'.  compute colslab( 187 ,1)= 'COL187'.  compute colslab( 188 ,1)= 'COL188'.  compute colslab( 189 ,1)= 'COL189'.  compute colslab( 190 ,1)= 'COL190'.  compute colslab( 191 ,1)= 'COL191'.  compute colslab( 192 ,1)= 'COL192'.  compute colslab( 193 ,1)= 'COL193'.  compute colslab( 194 ,1)= 'COL194'.  compute colslab( 195 ,1)= 'COL195'.  compute colslab( 196 ,1)= 'COL196'.  compute colslab( 197 ,1)= 'COL197'.  compute colslab( 198 ,1)= 'COL198'.  compute colslab( 199 ,1)= 'COL199'.  compute colslab( 200 ,1)= 'COL200'.  compute colslab( 201 ,1)= 'COL201'.  compute colslab( 202 ,1)= 'COL202'.  compute colslab( 203 ,1)= 'COL203'.  compute colslab( 204 ,1)= 'COL204'.  compute colslab( 205 ,1)= 'COL205'.  compute colslab( 206 ,1)= 'COL206'.  compute colslab( 207 ,1)= 'COL207'.  compute colslab( 208 ,1)= 'COL208'.  compute colslab( 209 ,1)= 'COL209'.  compute colslab( 210 ,1)= 'COL210'.  compute colslab( 211 ,1)= 'COL211'.  compute colslab( 212 ,1)= 'COL212'.  compute colslab( 213 ,1)= 'COL213'.  compute colslab( 214 ,1)= 'COL214'.  compute colslab( 215 ,1)= 'COL215'.  compute colslab( 216 ,1)= 'COL216'.  compute colslab( 217 ,1)= 'COL217'.  compute colslab( 218 ,1)= 'COL218'.  compute colslab( 219 ,1)= 'COL219'.  compute colslab( 220 ,1)= 'COL220'.  compute colslab( 221 ,1)= 'COL221'.  compute colslab( 222 ,1)= 'COL222'.  compute colslab( 223 ,1)= 'COL223'.  compute colslab( 224 ,1)= 'COL224'.  compute colslab( 225 ,1)= 'COL225'.  compute colslab( 226 ,1)= 'COL226'.  compute colslab( 227 ,1)= 'COL227'.  compute colslab( 228 ,1)= 'COL228'.  compute colslab( 229 ,1)= 'COL229'.  compute colslab( 230 ,1)= 'COL230'.  compute colslab( 231 ,1)= 'COL231'.  compute colslab( 232 ,1)= 'COL232'.  compute colslab( 233 ,1)= 'COL233'.  compute colslab( 234 ,1)= 'COL234'.  compute colslab( 235 ,1)= 'COL235'.  compute colslab( 236 ,1)= 'COL236'.  compute colslab( 237 ,1)= 'COL237'.  compute colslab( 238 ,1)= 'COL238'.  compute colslab( 239 ,1)= 'COL239'.  compute colslab( 240 ,1)= 'COL240'.  compute colslab( 241 ,1)= 'COL241'.  compute colslab( 242 ,1)= 'COL242'.  compute colslab( 243 ,1)= 'COL243'.  compute colslab( 244 ,1)= 'COL244'.  compute colslab( 245 ,1)= 'COL245'.  compute colslab( 246 ,1)= 'COL246'.  compute colslab( 247 ,1)= 'COL247'.  compute colslab( 248 ,1)= 'COL248'.  compute colslab( 249 ,1)= 'COL249'.  compute colslab( 250 ,1)= 'COL250'.  compute colslab( 251 ,1)= 'COL251'.  compute colslab( 252 ,1)= 'COL252'.  compute colslab( 253 ,1)= 'COL253'.  compute colslab( 254 ,1)= 'COL254'.  compute colslab( 255 ,1)= 'COL255'.  compute colslab( 256 ,1)= 'COL256'.  compute colslab( 257 ,1)= 'COL257'.  compute colslab( 258 ,1)= 'COL258'.  compute colslab( 259 ,1)= 'COL259'.  compute colslab( 260 ,1)= 'COL260'.  compute colslab( 261 ,1)= 'COL261'.  compute colslab( 262 ,1)= 'COL262'.  compute colslab( 263 ,1)= 'COL263'.  compute colslab( 264 ,1)= 'COL264'.  compute colslab( 265 ,1)= 'COL265'.  compute colslab( 266 ,1)= 'COL266'.  compute colslab( 267 ,1)= 'COL267'.  compute colslab( 268 ,1)= 'COL268'.  compute colslab( 269 ,1)= 'COL269'.  compute colslab( 270 ,1)= 'COL270'.  compute colslab( 271 ,1)= 'COL271'.  compute colslab( 272 ,1)= 'COL272'.  compute colslab( 273 ,1)= 'COL273'.  compute colslab( 274 ,1)= 'COL274'.  compute colslab( 275 ,1)= 'COL275'.  compute colslab( 276 ,1)= 'COL276'.  compute colslab( 277 ,1)= 'COL277'.  compute colslab( 278 ,1)= 'COL278'.  compute colslab( 279 ,1)= 'COL279'.  compute colslab( 280 ,1)= 'COL280'.  compute colslab( 281 ,1)= 'COL281'.  compute colslab( 282 ,1)= 'COL282'.  compute colslab( 283 ,1)= 'COL283'.  compute colslab( 284 ,1)= 'COL284'.  compute colslab( 285 ,1)= 'COL285'.  compute colslab( 286 ,1)= 'COL286'.  compute colslab( 287 ,1)= 'COL287'.  compute colslab( 288 ,1)= 'COL288'.  compute colslab( 289 ,1)= 'COL289'.  compute colslab( 290 ,1)= 'COL290'.  compute colslab( 291 ,1)= 'COL291'.  compute colslab( 292 ,1)= 'COL292'.  compute colslab( 293 ,1)= 'COL293'.  compute colslab( 294 ,1)= 'COL294'.  compute colslab( 295 ,1)= 'COL295'.  compute colslab( 296 ,1)= 'COL296'.  compute colslab( 297 ,1)= 'COL297'.  compute colslab( 298 ,1)= 'COL298'.  compute colslab( 299 ,1)= 'COL299'.  compute colslab( 300 ,1)= 'COL300'.  compute colslab=colslab(1:ncol(bootres),1).  compute colslab={colslab,conseq,vlabs}.  print colslab/title='Map of column names to model coefficients:'/clabels=' ','Conseqnt','Antecdnt'/format=a8.  end if.  end if.  do if (criterr=0 and boot > 0 and modelbt=1).  compute labstart=1.  print/title = '*********** BOOTSTRAP RESULTS FOR REGRESSION MODEL PARAMETERS ************'.  loop iboot = 1 to (nms+nys).  print outnames(1,iboot)/title = 'OUTCOME VARIABLE:'/format = A8.  compute vlabsm=vlabs(labstart:(labstart+(nump(1,iboot)-1)),1).  print bootcim(labstart:(labstart+(nump(1,iboot)-1)),:)/title=' '/rnames=vlabsm/ clabels='Coeff' 'BootMean' 'BootSE' 'BootLLCI' 'BootULCI'/format= F10.4 /space=0.  compute labstart=labstart+nump(1,iboot).  do if (iboot < (nms+nys)).  print/title= '----------'.  end if.  end loop.  end if.  do if (criterr = 0 and matrices=1).  print/title = '************************ MODEL DEFINITION MATRICES ************************'.  print/title = 'FROM variables are columns, TO variables are rows.'.  compute temp2=make(nrow(bcmat),ncol(bcmat),'0').  loop i = 2 to nrow(bcmat).  loop j = 1 to (ncol(bcmat)-1).  do if (bcmat(i,j)=1).  compute temp2(i,j)='1'.  end if.  do if (j >= i).  compute temp2(i,j)=' '.  end if.  end loop.  end loop.  compute temp2=temp2(2:nrow(bcmat),(1:(ncol(bcmat)-1))).  do if (nms > 0).  compute cmatlabs={xnames,mnames}.  compute rmatlabs={mnames,ynames}.  end if.  do if (nms = 0).  compute cmatlabs={xnames}.  compute rmatlabs={ynames}.  end if.  print temp2/title='BMATRIX: Paths freely estimated (1) and fixed to zero (0):'/cnames=cmatlabs/rnames=rmatlabs/format A3.  compute z=0.  do if (rsum(csum(wcmat))<>0).  compute temp2=make(nrow(wcmat),ncol(wcmat),'0').  loop i = 2 to nrow(wcmat).  loop j = 1 to (ncol(wcmat)-1).  do if (wcmat(i,j)=1).  compute temp2(i,j)='1'.  end if.  do if (j >= i).  compute temp2(i,j)=' '.  end if.  end loop.  end loop.  compute temp2=temp2(2:nrow(wcmat),(1:(ncol(wcmat)-1))).  print temp2/title='WMATRIX: Paths moderated (1) and not moderated (0) by W:'/cnames=cmatlabs/rnames=rmatlabs/format A3.  end if.  do if (rsum(csum(zcmat))<>0).  compute temp2=make(nrow(zcmat),ncol(zcmat),'0').  loop i = 2 to nrow(zcmat).  loop j = 1 to (ncol(zcmat)-1).  do if (zcmat(i,j)=1).  compute temp2(i,j)='1'.  end if.  do if (j >= i).  compute temp2(i,j)=' '.  end if.  end loop.  end loop.  compute temp2=temp2(2:nrow(zcmat),(1:(ncol(zcmat)-1))).  print temp2/title='ZMATRIX: Paths moderated (1) and not moderated (0) by Z:'/cnames=cmatlabs/rnames=rmatlabs/format a3.  end if.  do if (rsum(csum(wzcmat))<>0).  compute temp2=make(nrow(wzcmat),ncol(wzcmat),'0').  loop i = 2 to nrow(wzcmat).  loop j = 1 to (ncol(wzcmat)-1).  do if (wzcmat(i,j)=1).  compute temp2(i,j)='1'.  end if.  do if (j >= i).  compute temp2(i,j)=' '.  end if.  end loop.  end loop.  compute temp2=temp2(2:nrow(wzcmat),(1:(ncol(wzcmat)-1))).  print temp2/title='WZMATRIX: W moderated paths moderated (1) and not moderated (0) by Z:'/cnames=cmatlabs/rnames=rmatlabs/format a3.  end if.  do if (ncs > 0).  print ccmat/title='CMATRIX: Covariates (columns) in (1) and not in (0) the models of M and Y (rows):'/rnames=rmatlabs/cnames=covnames.  end if.  end if.  print/title = '*********************** ANALYSIS NOTES AND ERRORS ************************'.  do if (criterr=0).  print conf/title = 'Level of confidence for all confidence intervals in output:'/format = F8.4.  do if (boot > 0).  do if (goodboot = boot).  print boot/title='Number of bootstrap samples for percentile bootstrap confidence intervals:'.  end if.  end if.  do if (mc > 0).  print mc/title='Number of samples for Monte Carlo confidence intervals:'.  end if.  do if (wnotev > 0 and printw=1).  do if (wnotev=1).  print/title = 'W values in conditional tables are the 16th, 50th, and 84th percentiles.'.  else if (wnotev=2).  do if (minwwarn=0 and maxwwarn=0).  print/title = 'W values in conditional tables are the mean and +/- SD from the mean.'.  end if.  do if (minwwarn=1).  print/title = 'W values in conditional tables are the minimum, the mean, and 1 SD above the mean.'.  end if.  do if (maxwwarn=1).  print/title = 'W values in conditional tables are 1 SD below the mean, the mean, and the maximum.'.  end if.  end if.  end if.  do if (znotev > 0 and printz=1).  do if (znotev=1).  print/title = 'Z values in conditional tables are the 16th, 50th, and 84th percentiles.'.  else if (znotev=2).  do if (minzwarn=0 and maxzwarn=0).  print/title = 'Z values in conditional tables are the mean and +/- SD from the mean.'.  end if.  do if (minzwarn=1).  print/title = 'Z values in conditional tables are the minimum, the mean, and 1 SD above the mean.'.  end if.  do if (maxzwarn=1).  print/title = 'Z values in conditional tables are 1 SD below the mean, the mean, and the maximum.'.  end if.  end if.  end if.  do if (minwwarn > 0).  print/title = 'NOTE: One SD below the mean is below the minimum observed in the data for W,'.  print/title = ' so the minimum measurement on W is used for conditioning instead.'/space=0.  end if.  do if (maxwwarn > 0).  print/title = 'NOTE: One SD above the mean is above the maximum observed in the data for W,'.  print/title = ' so the maximum measurement for W is used for conditioning instead.'/space=0.  end if.  do if (minzwarn > 0).  print/title = 'NOTE: One SD below the mean is below the minimum observed in the data for Z,'.  print/title = ' so the minimum measurement for Z is used for conditioning instead.'/space=0.  end if.  do if (maxzwarn > 0).  print/title = 'NOTE: One SD above the mean is above the maximum observed in the data for Z,'.  print/title = ' so the maximum measurement for Z is used for conditioning instead.'/space=0.  end if.  do if (pstog=1).  print/title= 'NOTE: Standardized coefficients for dichotomous or multicategorical X are in'.  print/title= ' partially standardized form.'/space=0.  end if.  loop i = 1 to 100.  do if (notecode(i,1)=1).  print/title = 'NOTE: COVMY is ignored when using CMATRIX option.'.  end if.  do if (notecode(i,1)=2).  print/title = 'NOTE: Confidence level restricted to between 50 and 99.9999%.95% confidence is provided in output'.  end if.  do if (notecode(i,1)=3).  print centvar/title = 'NOTE: The following variables were mean centered prior to analysis:'/format = a8.  end if.  do if (notecode(i,1) = 4).  print/title = 'NOTE: A heteroscedasticity consistent standard error and covariance matrix estimator was used.'.  end if.  do if (notecode(i,1) = 5).  print/title = 'NOTE: The HC3 option has been replaced with HC.See the documentation.'.  end if.  do if (notecode(i,1) = 6).  print/title = 'NOTE: Due to estimation problems, some bootstrap samples had to be replaced.'.  print badboot/title=' The number of times this happened was:'/space=0/format=F8.0.  end if.  do if (notecode(i,1) = 7).  print/title = 'NOTE: The bootstrapping was not completed due to problematic bootstrap samples.'.  print/title = ' Bootstrap confidence intervals are therefore suppressed.'/space=0.  end if.  do if (notecode(i,1) = 8).  print/title = 'NOTE: The number of bootstrap samples was adjusted upward given your desired confidence.'.  end if.  do if (notecode(i,1) = 9).  print/title = 'NOTE: WMODVAL is ignored when W is specified as multicategorical.'.  end if.  do if (notecode(i,1) = 10).  print/title = 'NOTE: ZMODVAL is ignored when Z is specified as multicategorical.'.  end if.  do if (notecode(i,1) = 11).  print/title = 'NOTE: Total effect model and estimate generated only when all covariates are specified in all'.  print/title = ' models of M and Y.'/space=0.  end if.  do if (notecode(i,1) = 12).  print/title = 'NOTE: Total effect model and estimate generated only when X is freely estimated to affect each M'.  print/title = ' and both X and M are freely estimated to affect Y'/space=0.  end if.  do if (notecode(i,1) = 13).  print/title = 'NOTE: There are too many pairwise contrasts to conduct with this model.'.  end if.  do if (notecode(i,1) = 14).  print/title = 'NOTE: The number of contrast weights must equal the number of indirect effects.'.  end if.  do if (notecode(i,1) = 15).  print/title = 'NOTE: Monte Carlo confidence intervals not available for this model.'.  print/title = ' Bootstrapping is used instead.'/space=0.  end if.  do if (notecode(i,1) = 16).  print/title = 'NOTE: The number of Monte Carlo samples was adjusted upward given your desired confidence.'.  end if.  do if (notecode(i,1) = 19).  print/title = 'NOTE: Your contrast matrix is invalid or not applicable to this model.'.  end if.  do if (notecode(i,1) = 20).  print/title = 'NOTE: One of the groups specified by your contrast matrix does not exist in the data.'.  end if.  do if (notecode(i,1) = 21).  print/title = 'NOTE: The VARORDER option is not available in this release.'.  end if.  do if (notecode(i,1) = 22).  print/title = 'NOTE: The VMODVAL and QMODVAL options are not available in this release.'.  end if.  do if (notecode(i,1) = 23).  print/title = 'NOTE: The QUANTILE option is not available in this release.'.  end if.  do if (notecode(i,1) = 24).  print/title = 'NOTE: Total effect model not available with dichotomous Y'.  end if.  do if (notecode(i,1) = 25).  print/title = 'NOTE: Effect size option not available with dichotomous Y'.  end if.  do if ((notecode(i,1) = 26) and nms > 0).  print/title = 'NOTE: Direct and indirect effects of X on Y are on a log-odds metric.'.  end if.  do if (notecode(i,1) = 27).  print/title = 'NOTE: Standardized coefficients not available for models with moderators.'.  end if.  do if (notecode(i,1) = 28).  print/title = 'NOTE: The contrast option is not available with a multicategorical X.'.  end if.  end loop.  do if (toomany=1).  print/title='NOTE: Variables names longer than eight characters can produce incorrect output.'.  print/title=' Shorter variable names are recommended.'/space=0.  end if.  end if.  loop i = 1 to 100.  do if (errcode(i,1)=1).  print/title = 'ERROR: You must specify a Y and an X variable.'.  end if.  do if (errcode(i,1)=2).  print/title = 'ERROR: X, M, or Y variable used more than once or W and Z are the same variable.'.  do if (toomany = 1).  print/title = ' This could be caused by the use of variables names longer'/space=0.  print varnames/title = ' than eight characters.Here are the variables I see:'/space=0/format=A8.  end if.  end if.  do if (errcode(i,1)=3).  print/title = 'ERROR: You have specified more than one variable for W, Y, X, or Z'.  end if.  do if (errcode(i,1)=4).  print/title = 'ERROR: A variable specified as multicategorical has more than nine categories.'.  end if.  do if (errcode(i,1)=5).  print/title = 'ERROR: One of the categories contains only a single case.'.  end if.  do if (errcode(i,1)=6).  print/title = 'ERROR: Invalid model number in this version of PROCESS.'.  end if.  do if (errcode(i,1)=7).  print/title = 'ERROR: Invalid model number.'.  end if.  do if (errcode(i,1)=8).  print/title = 'ERROR: You must specify an M variable for this model.'.  end if.  do if (errcode(i,1)=9).  print/title = 'ERROR: You have specified an M variable in a model that does not use it.'.  print/title = 'In this release of PROCESS, moderators are W and Z in models 1, 2, and 3.'/space=0.  end if.  do if (errcode(i,1)=10).  print/title = 'ERROR: You have specified a W variable in a model that does not use it.'.  end if.  do if (errcode(i,1)=11).  print/title = 'ERROR: You have not specified a W variable in a model that requires it.'.  end if.  do if (errcode(i,1)=12).  print/title = 'ERROR: You have specified a Z variable in a model that does not use it.'.  end if.  do if (errcode(i,1)=13).  print/title = 'ERROR: You have not specified a Z variable in a model that requires it.'.  end if.  do if (errcode(i,1)=14).  print/title = 'ERROR: V and Q are not proper specifications in this release of PROCESS.'.  print/title = ' Moderators must be specified as W and/or Z.'/space=0.  end if.  do if (errcode(i,1)=15).  print/title = 'ERROR: One of your model variables exhibits no variation (it is a constant).'.  end if.  do if (errcode(i,1)=16).  print/title = 'ERROR: BMATRIX is not the correct length or is otherwise invalid.'.  end if.  do if (errcode(i,1)=17).  print/title = 'ERROR: WMATRIX is not the correct length or is otherwise invalid.'.  end if.  do if (errcode(i,1)=18).  print/title = 'ERROR: ZMATRIX is not the correct length or is otherwise invalid.'.  end if.  do if (errcode(i,1)=19).  print/title = 'ERROR: WZMATRIX is not the correct length or is otherwise invalid.'.  end if.  do if (errcode(i,1)=20).  print/title = 'ERROR: A path fixed at zero cannot be moderated.'.  end if.  do if (errcode(i,1)=21).  print/title = 'ERROR: If only one moderator is specified, it must be specified as W.'.  end if.  do if (errcode(i,1)=22).  print/title = 'ERROR: In BMATRIX, X must be specified to affect at least one variable.'.  end if.  do if (errcode(i,1)=23).  print/title = 'ERROR: In BMATRIX, at least one variable must be specified to affect Y.'.  end if.  do if (errcode(i,1)=24).  print/title = 'ERROR: You must specify a model number or a custom BMATRIX specification.'.  end if.  do if (errcode(i,1)=25).  print/title = 'ERROR: BMATRIX cannot be used in conjunction with a model number.'.  end if.  do if (errcode(i,1)=26).  print/title = 'ERROR: Your model has a dangling mediator (all Ms must affect and be affected).'.  end if.  do if (errcode(i,1)=27).  print/title = 'ERROR: CLUSTER is not available on this release of PROCESS.'.  end if.  do if (errcode(i,1)=29).  print/title = 'ERROR: CMATRIX is not the correct length or is otherwise invalid.'.  end if.  do if (errcode(i,1)=30).  print/title = 'ERROR: In CMATRIX, all covariates must be assigned to an M or a Y.'.  end if.  do if (errcode(i,1)=31).  print/title = 'ERROR: A linear or near linear dependency (singularity) exists in the data.'.  end if.  do if (errcode(i,1)=32).  print/title = 'ERROR: Models 80 and 81 require between 3 and 6 mediators.'.  end if.  do if (errcode(i,1)=33).  print/title = 'ERROR: Model 82 requires 4 mediators.'.  end if.  do if (errcode(i,1)=34).  print/title = 'ERROR: This model number requires between 2 and 6 mediators.'.  end if.  do if (errcode(i,1)=35).  print/title = 'ERROR: In a model with only one moderator, that moderator must be W.'.  end if.  do if (errcode(i,1)=36).  print/title = 'ERROR: A serial mediation model cannot have more than 6 mediators.'.  end if.  do if (errcode(i,1)=37).  print/title = 'ERROR: No more than 10 mediators are allowed in a PROCESS command.'.  end if.  do if (errcode(i,1)=38).  print/title = 'ERROR: XCATCODE is not provided, not the correct length, or is otherwise invalid.'.  end if.  do if (errcode(i,1)=39).  print/title = 'ERROR: WCATCODE is not provided, not the correct length, or is otherwise invalid.'.  end if.  do if (errcode(i,1)=40).  print/title = 'ERROR: ZCATCODE is not provided, not the correct length, or is otherwise invalid.'.  end if.  do if (errcode(i,1)=41).  print/title = 'ERROR: Models 1, 2, 3, and 74 cannot be customized.'.  end if.  do if (errcode(i,1)=42).  print/title = 'ERROR: WS option available only in PROCESS v2. Or use the MEMORE macro instead.'.  print/title = ' MEMORE can be downloaded from www.akmontoya.com.'/space=0.  end if.  do if (errcode(i,1)=43).  print/title = 'ERROR: PROCESS does not allow dichotomous mediators.'.  end if.  do if (errcode(i,1)=45).  print/title = 'ERROR: In model 74, X and W must be the same variable.'.  end if.  do if (errcode(i,1)=46).  print/title = 'ERROR: Model 74 is temporarily disabled in this release of PROCESS.'.  end if.  do if (errcode(i,1)=50).  print/title = 'ERROR: A multicategorical moderator cannot be specified as a covariate.'.  end if.  do if (errcode(i,1)=51).  print/title = 'ERROR: A variable you specified as a covariate is a moderator in all equations.'.  end if.  do if (errcode(i,1)=52 and mcerpt=0).  compute mcerpt=1.  print/title = 'ERROR: A variable specified as multicategorical must have at least three categories'.  end if.  do if (errcode(i,1) = 47).  do if (iterrmod=1).  print/title = 'ERROR: Iteration for Y model didn''t converge to a solution.Interpret results with caution.'.  print/title = ' Try increasing the number of iterations, though this will slow down computation.'/space=0.  end if.  do if (bootiter=1).  print/title = 'ERROR: Nonconvergence during bootstrapping.Interpet bootstrap results with caution.'.  end if.  end if.  end loop.  END MATRIX. |
| resource | Processor time | 00 00:00:13.250 |
|  | Time used | 00 00:00:13.565 |

[Dataset 1] F: \ submitted version \ data.sav

Run MATRIX procedure:

**************** PROCESS Procedure for SPSS Version 3.4.1 ****************

Written by Andrew F.Hayes, Ph.D.www.afhayes.com

Documentation available in Hayes (2018).www.guilford.com/p/hayes3

**************************************************************************

Model : 4

Y: Z job burnout

X: Z stress perception

M: Z Mental health

Sample

Size: 792

**************************************************************************

OUTCOME VARIABLE:

Z mental health

Model Summary

R R-sq MSE F df1 df2 p

.5828 .3396 .6612 406.2912 1.0000 790.0000 .0000

Model

coeff se t p LLCI ULCI

constant .0000 .0289 .0000 1.0000 -.0567 .0567

Z stress perception.5828 .0289 20.1567 .0000 .5260 .6395

Standardized coefficients

coeff

Z stress perception .5828

**************************************************************************

OUTCOME VARIABLE:

Z job burnout

Model Summary

R R-sq MSE F df1 df2 p

.7183 .5159 .4853 420.4984 2.0000 789.0000 .0000

Model

coeff se t p LLCI ULCI

constant .0000 .0248 .0000 1.0000 -.0486 .0486

Z stress perception.5788 .0305 18.9897 .0000 .5190 .6386

Z Mental health.2056 .0305 6.7444 .0000 .1457 .2654

Standardized coefficients

coeff

Z stress perception.5788

Z Mental health.2056

************************** TOTAL EFFECT MODEL ****************************

OUTCOME VARIABLE:

Z job burnout

Model Summary

R R-sq MSE F df1 df2 p

.6986 .4880 .5126 753.1001 1.0000 790.0000 .0000

Model

coeff se t p LLCI ULCI

constant .0000 .0254 .0000 1.0000 -.0499 .0499

Z stress perception.6986 .0255 27.4427 .0000 .6486 .7486

Standardized coefficients

coeff

Z stress perception.6986

************** TOTAL, DIRECT, AND INDIRECT EFFECTS OF X ON Y **************

Total effect of X on Y

Effect se t p LLCI ULCI c_ps c_cs

.6986 .0255 27.4427 .0000 .6486 .7486 .6986 .6986

Direct effect of X on Y

Effect se t p LLCI ULCI c'_ps c'_cs

.5788 .0305 18.9897 .0000 .5190 .6386 .5788 .5788

Indirect effect(s) of X on Y:

Effect BootSE BootLLCI BootULCI

Z Mental health.1198 .0219 .0770 .1624

Partially standardized indirect effect(s) of X on Y:

Effect BootSE BootLLCI BootULCI

Z Mental health.1198 .0216 .0769 .1623

Completely standardized indirect effect(s) of X on Y:

Effect BootSE BootLLCI BootULCI

Z Mental health.1198 .0215 .0776 .1625

*********** BOOTSTRAP RESULTS FOR REGRESSION MODEL PARAMETERS ************

OUTCOME VARIABLE:

Z mental health

Coeff BootMean BootSE BootLLCI BootULCI

constant .0000 -.0004 .0286 -.0552 .0565

Z stress perception.5828 .5829 .0308 .5212 .6424

----------

OUTCOME VARIABLE:

Z job burnout

Coeff BootMean BootSE BootLLCI BootULCI

constant .0000 -.0007 .0242 -.0485 .0467

Z stress perception.5788 .5789 .0333 .5136 .6475

Z Mental health.2056 .2050 .0342 .1368 .2707

*********************** ANALYSIS NOTES AND ERRORS ************************

Level of confidence for all confidence intervals in output:

95.0000

Number of bootstrap samples for percentile bootstrap confidence intervals:

5000

NOTE: Variables names longer than eight characters can produce incorrect output.

Shorter variable names are recommended.

------ END MATRIX -----

* Encoding: UTF-8.

/* PROCESS version 3.4.1 */.

/* Written by Andrew F.Hayes */.

/* www.afhayes.com */.

/* www.processmacro.org */.

/* Copyright 2020 by Andrew F.Hayes */.

/* Documented in http://www.guilford.com/p/hayes3 */.

/* PROCESS workshop schedule at http://www.processmacro.org/workshops.html */.

/* Distribution of this code in any form, except through processmacro.org, is prohibited */.

/* without the permission of the copyright holder */.

/* THIS SOFTWARE IS PROVIDED "AS IS", WITHOUT WARRANTY OF ANY KIND */.

/* EXPRESS OR IMPLIED, INCLUDING BUT NOT LIMITED TO THE WARRANTIES OF */.

/* MERCHANTABILITY, FITNESS FOR A PARTICULAR PURPOSE AND NONINFRINGEMENT */.

/* IN NO EVENT SHALL THE COPYRIGHT HOLDERS BE LIABLE FOR ANY CLAIM, */.

/* DAMAGES OR OTHER LIABILITY, WHETHER IN AN ACTION OF CONTRACT, TORT */.

/* OR OTHERWISE, ARISING FROM, OUT OF OR IN CONNECTION WITH THE */.

/* SOFTWARE OR THE USE OR OTHER DEALINGS IN THE SOFTWARE */.

/* USE OF THIS SOFTWARE IMPLIES AGREEMENT WITH THESE TERMS */.

set printback=off.

**matrix**

| **notes appended to a book** | | |
| --- | --- | --- |
| The output created | | 14-July-2023 at 11:41:51 seconds |
| explanatory note | |  |
| import | data | F: \ submitted version \ data.sav |
|  | Datasets of activities | data set 1 |
|  | filter | <none> |
|  | weight | <none> |
|  | Split files | <none> |
|  | The N rows in the working data file | 792 |
| grammar | | MATRIX.  compute wnames='xxxxx'.  compute znames='xxxxx'.  compute mcerpt=0.  compute wiscov=0.  compute ziscov=0.  compute errcode=make(100,1,0).  compute notecode=make(100,1,0).  compute model = trunc( 4 ).  compute iterate = abs(trunc( 100 )).  compute converge = abs( 0.00001 ).  compute itprobtg=0.  compute v2tag=0.  compute maxwwarn=0.  compute minwwarn=0.  compute maxzwarn=0.  compute minzwarn=0.  compute toomany=0.  compute wdich=0.  compute zdich=0.  compute wnotev=0.  compute znotev=0.  compute nxpval=1.  compute nwpval=1.  compute nzpval=1.  compute errs=1.  compute notes=1.  compute criterr=0.  compute novar=0.  compute adjust=0.  compute ncs=0.  compute serial=0.  compute sobelok=0.  compute hasw=0.  compute hasz=0.  compute printw=0.  compute printz=0.  compute counterf=0.  compute wmodcust=0.  compute zmodcust=0.  compute booting=0.  compute bootiter=0.  compute iterrmod=0.  compute cov = 'xxxxx'.  compute varorder=( 0 <> 0).  compute nws=0.  compute w= 'xxxxx'.  compute nzs=0.  compute z = 'xxxxx'.  compute nms=0.  compute m = 'Z burnout score'.  compute nys=0.  compute y = 'Z mental health score'.  compute nxs=0.  compute x = 'Z Stress Sense Scale score'.  compute v = 'xxxxx'.  compute q = 'xxxxx'.  compute oldvars= 'xxxxx'.  compute mcxok=0.  compute mcwok=0.  compute mczok=0.  compute xprod=0.  compute zprod=0.  compute wprod=0.  compute modcok=0.  compute hc3=trunc( 0 ).  compute jn=( 0 = 1).  compute effsize=( 1 =1).  compute normal=( 0 =1).  compute xmtest=( 0 =1).  compute stand=( 1 =1).  do if (stand=1).  compute effsize=1.  end if.  compute pstog=0.  compute sobelok=0.  compute normal=( 0 =1).  compute mdichok=( 0 =1).  compute contrast={ 999 }.  compute ncontr=ncol(contrast).  compute ncontrow=nrow(contrast).  do if (contrast(1,1) = 999).  compute ncontr=1.  compute contrast=0.  end if.  do if (ncontr = 1).  compute contrast=trunc(contrast).  do if (contrast > 2 or contrast < 0)).  compute ncontr=1.  compute contrast = 0.  end if.  end if.  do if (ncontr > 1).  compute contvec=contrast.  compute contrast=3.  do if (ncontrow > 1).  compute contrast=0.  compute modcok=1.  compute wcontval=contvec(:,1).  compute zcontval=contvec(:,2).  do if ((ncontr <> 2) or (ncontrow <> 2)).  compute notecode(notes,1) = 19.  compute notes = notes + 1.  compute modcok=0.  end if.  end if.  end if.  do if (varorder = 1).  compute notecode(notes,1) = 21.  compute notes = notes + 1.  end if.  do if ( 999 <> 999 or 999 <> 999).  compute notecode(notes,1) = 22.  compute notes = notes + 1.  end if.  compute modelbt=( 1 =1).  compute cluster= 'xxxxx'.  compute matrices=( 0 =1).  compute covcoeff=( 0 =1).  compute covmy=trunc( 0 ).  do if (covmy < 0 or covmy > 2).  compute covmy = 0.  end if.  compute boot = abs(trunc( 5000 )).  compute mc=abs(trunc( 0 )).  compute hc=trunc( 5 ).  compute intprobe = .1.  do if (intprobe < 0 or intprobe > 1).  compute intprobe = .10.  end if.  compute plot=trunc( 0 ).  do if (plot < 0 or plot > 2).  compute plot=0.  end if.  compute total=( 1 =1).  compute dototal=0.  compute saveboot = ( 0 = 1).  compute saveest=( 0 = 2).  do if (hc >= 0 and hc < 5).  compute notecode(notes,1) = 4.  compute notes = notes + 1.  end if.  do if (hc > 5 or hc < 0).  compute hc=5.  end if.  compute mcw=trunc( 0 ).  compute mcz=trunc( 0 ).  compute mcx=trunc( 0 ).  do if (mcx > 0 and model = 74).  compute mcw=mcx.  end if.  do if (mcw > 0 and model = 74).  compute mcx=mcw.  end if.  do if (mcx > 0 and contrast > 0).  compute notecode(notes,1) = 28.  compute notes = notes + 1.  compute contrast=0.  end if.  compute nxvls=1.  compute nmvls=1.  compute nwvls=1.  compute nzvls=1.  compute paths=999.  compute pathsw=999.  compute pathsz=999.  compute pathswz=999.  compute pathsmod=999.  compute pathtype=999.  compute obscoeff=999.  compute pathsdv={' '}.  compute quantile=1.  do if ( 999 <>999).  compute notecode(notes,1) = 23.  compute notes = notes + 1.  end if.  compute moments=( 0 =1).  do if (moments=1).  compute quantile=0.  end if.  compute center=trunc( 1 ).  compute bmatrix={ -999 }.  compute wmatrix={ -999 }.  compute zmatrix={ -999 }.  compute wzmatrix={ -999 }.  compute cmatrix={ -999 }.  compute xcatcode={ -999 }.  compute wcatcode={ -999 }.  compute zcatcode={ -999 }.  compute needed=0.  compute conf= 95.  do if (trunc( 95 ) >= 100 or (trunc( 95 ) <= 50)).  compute conf = 95.  compute notecode(notes,1)=2.  compute notes=notes+1.  end if.  do if (model > 0 and model < 4 and modelbt=0).  compute boot=0.  compute mc=0.  end if.  do if (boot > 0 and mc > 0).  compute boot=0.  end if.  compute p0=-.322232431088.  compute p1 = -1.  compute p2 = -.342242088547.  compute p3 = -.0204231210245.  compute p4 = -.0000453642210148.  compute q0 = .0993484626060.  compute q1 = .588581570495.  compute q2 = .531103462366.  compute q3 = .103537752850.  compute q4 = .0038560700634.  compute alpha2 = (1-(conf/100))/2.  compute cilm=alpha2*2.  compute y5=sqrt(-2*ln(alpha2)).  compute xp2=(y5+((((y5*p4+p3)*y5+p2)*y5+p1)*y5+p0)/((((y5*q4+q3)*y5+q2)*y5+q1)*y5+q0)).  compute medlb={' M1 :';' M2 :';' M3 :';' M4 :';' M5 :';' M6 :';' M7 :';' M8 :';' M9 :';' M10 :'}.  compute medlb2={'(M1)','(M2)','(M3)','(M4)','(M5)','(M6)','(M7)','(M8)','(M9)','(M10)'}.  compute xlb={' X1 :';' X2 :';' X3 :';' X4 :';' X5 :';' X6 :';' X7 :';' X8 :';' X9 :'}.  compute highlbw={'M1*W'; 'M2*W'; 'M3*W'; 'M4*W'; 'M5*W'; 'M6*W'; 'M7*W'; 'M8*W'; 'M9*W'; 'M10*W'}.  compute highlbz={'M1*Z'; 'M2*Z'; 'M3*Z'; 'M4*Z'; 'M5*Z'; 'M6*Z'; 'M7*Z'; 'M8*Z'; 'M9*Z';'M10*Z'}.  compute highlbwz={'M1*W*Z'; 'M2*W*Z'; 'M3*W*Z'; 'M4*W*Z'; 'M5*W*Z'; 'M6*W*Z'; 'M7*W*Z'; 'M8*W*Z'; 'M9*W*Z';'M10*W*Z'}.  compute highlbbt={'BOTH(M1)'; 'BOTH(M2)'; 'BOTH(M3)'; 'BOTH(M4)'; 'BOTH(M5)'; 'BOTH(M6)'; 'BOTH(M7)'; 'BOTH(M8)'; 'BOTH(M9)';'BTH(M10)'}.  compute highlbx={'M1*X'; 'M2*X'; 'M3*X'; 'M4*X'; 'M5*X'; 'M6*X'; 'M7*X'; 'M8*X'; 'M9*X'; 'M10*X'}.  compute skipwz=0.  compute validm={1,1,1,1,1,1,1,1,1,1,1,1,1,1,1,1,1,1,1,1,1,1,0,0,0,0,0,1,1,0,0,0,0, 0,0,0,0,0,0,0,0,0,0,0,0,0,0,0,0,0,0,0,0,0,0,0,0,1,1,1,1,1,1,1,1,1,1,1,1,1,1,1,1,0,1,1,0,0,0,1,1, 1,1,1,1,1,1,1,1,1,1,1}.  do if (( 0 =1)=1).  compute errcode(errs,1)=42.  compute errs=errs+1.  compute criterr=1.  end if.  do if (model > 0 and model < 93).  do if (validm(1,model)=0).  do if (model <> 74).  compute errcode(errs,1)=6.  compute errs=errs+1.  compute criterr=1.  end if.  do if (model = 74).  compute errcode(errs,1)=46.  compute errs=errs+1.  compute criterr=1.  end if.  end if.  release validm.  end if.  do if ((model > 92 or model < 0) and model <> 999)).  compute errcode(errs,1)=7.  compute errs=errs+1.  compute criterr=1.  end if.  do if (model = 999 and bmatrix(1,1)=-999).  compute errcode(errs,1)=24.  compute errs=errs+1.  compute criterr=1.  end if.  do if (model <> 999 and bmatrix(1,1) <> -999).  compute errcode(errs,1)=25.  compute errs=errs+1.  compute criterr=1.  end if.  do if ((model = 74 or (model > 0 and model < 4)) and ((wmatrix(1,1) <> -999) or (zmatrix(1,1)<>-999) or (wzmatrix(1,1)<>-999)))).  compute errcode(errs,1)=41.  compute errs=errs+1.  compute criterr=1.  end if.  do if (hc3 <> 0).  compute notecode(notes,1) = 5.  compute notes = notes + 1.  do if (hc3 = 1).  compute hc=3.  end if.  end if.  do if ((v <> 'xxxxx') or (q <> 'xxxxx')).  compute errcode(errs,1)=14.  compute errs=errs+1.  compute errcode(errs,1)=48.  compute errs=errs+1.  compute criterr=1.  end if.  do if (oldvars <> 'xxxxx').  compute errcode(errs,1)=48.  compute errs=errs+1.  compute criterr=1.  end if.  do if (cluster <> 'xxxxx').  compute errcode(errs,1)=27.  compute errs=errs+1.  compute criterr=1.  end if.  do if ((y = 'xxxxx') or (x = 'xxxxx')).  compute errcode(errs,1)=1.  compute errs=errs+1.  compute criterr=1.  end if.  do if ((m = 'xxxxx') and model > 3).  compute errcode(errs,1)=8.  compute errs=errs+1.  compute criterr=1.  end if.  do if (criterr=0).  get ytmp / variables = Z mental health score / names = ynames / MISSING = 99999.  compute nys=ncol(ytmp).  compute needed=nys.  compute n=nrow(ytmp).  compute varnames={ynames}.  compute dat=ytmp.  .  compute toomany=1.  .  compute modelvar={ '4' ;t(ynames)}.  do if ( 4 =999).  compute modelvar(1,1)='CUSTOM'.  end if.  get xtmp / variables = Z Pressure Perception Scale score / names = xnames / MISSING = 99999.  compute nxs=ncol(xtmp).  compute n=nrow(xtmp).  compute needed=needed+nxs.  compute varnames={varnames,xnames}.  compute xcatlab=t(xnames).  compute dat={dat,xtmp}.  .  compute toomany=1.  .  compute modelvar={modelvar;t(xnames)}.  do if (nxs = 1).  compute modelvlb={'Model :';' Y :';' X :'}.  else.  compute modelvlb={'Model :';' Y :';xlb(1:nxs,1)}.  end if.  do if (m <> 'xxxxx').  get mtmp / variables = Z Burnout score / names = mnames / MISSING = 99999.  compute nms=ncol(mtmp).  compute mprod=make(1,nms,0).  compute n=nrow(mtmp).  compute needed=needed+nms.  compute varnames={varnames,mnames}.  compute dat={dat,mtmp}.  compute modelvar={modelvar;t(mnames)}.  compute x2m=make(99,nms,0).  compute m2y=make(99,nms,0).  compute onem=make(nms,1,1).  .  compute toomany=1.  .  do if (nms > 1 and nms < 11).  compute modelvlb={modelvlb;medlb(1:nms,1)}.  else.  compute modelvlb={modelvlb;' M :'}.  end if.  do if (nms > 0 and model < 4).  compute errcode(errs,1)=9.  compute errs=errs+1.  compute errcode(errs,1)=48.  compute errs=errs+1.  compute criterr=1.  end if.  end if.  compute wlocatet=0.  compute wlocate=0.  do if (w <> 'xxxxx').  get wtmp/variables = xxxxx /names = wnames/MISSING = 99999.  compute nws=ncol(wtmp).  compute n=nrow(wtmp).  .  .  compute varnames={varnames,wnames}.  compute wlocate=ncol(varnames).  do if (model=74).  compute wlocatet=1.  do if (xnames <> wnames).  compute errcode(errs,1)=45.  compute errs=errs+1.  compute criterr=1.  end if.  end if.  compute wcatlab=t(wnames).  compute dat={dat,wtmp}.  compute modelvar={modelvar;t(wnames)}.  compute modelvlb={modelvlb;' W :'}.  end if.  do if (z <> 'xxxxx').  get ztmp/variables = xxxxx /names = znames/MISSING = 99999.  compute nzs=ncol(ztmp).  compute n=nrow(ztmp).  .  .  compute varnames={varnames,znames}.  compute zcatlab=t(znames).  compute dat={dat,ztmp}.  compute modelvar={modelvar;t(znames)}.  compute modelvlb={modelvlb;' Z :'}.  end if.  do if (cov <> 'xxxxx').  get ctmp/variables = xxxxx /names = covnames/MISSING = 99999.  compute ncs=ncol(ctmp).  compute n=nrow(ctmp).  .  .  compute varnames={varnames,covnames}.  compute dat={dat,ctmp}.  end if.  do if (nws > 1 or nzs > 1 or nys > 1 or nxs > 1).  compute errcode(errs,1)=3.  compute errs=errs+1.  compute criterr=1.  end if.  do if ((model = 80 or model = 81) and (nms < 3 or nms > 6)).  compute errcode(errs,1)=32.  compute errs=errs+1.  compute criterr=1.  end if.  do if (model = 82 and nms <> 4).  compute errcode(errs,1)=33.  compute errs=errs+1.  compute criterr=1.  end if.  do if (nms > 10).  compute errcode(errs,1)=37.  compute errs=errs+1.  compute criterr=1.  end if.  do if ((model = 6 or (model > 82 and model < 999)) and (nms < 2 or nms > 6)).  compute errcode(errs,1)=34.  compute errs=errs+1.  compute criterr=1.  end if.  compute match=0.  compute match2=0.  compute mcwzcov=0.  loop i = 1 to (ncol(varnames)-1).  loop j = (i+1) to ncol(varnames).  do if (varnames(i)=varnames(j)).  do if (i < (nxs+nms+nys+1)).  compute match2=match2+1.  end if.  do if (wlocatet=1 and i=2 and j=wlocate).  compute match2=match2-1.  end if.  do if ((wnames=znames) and (nws > 0 or nzs > 0))).  compute match2=match2+1.  end if.  do if (i < (ncol(varnames)-ncs+1)) and j > (ncol(varnames)-ncs)).  do if ((varnames(j)=wnames) and mcw=0)).  compute match=0.  compute wiscov=(j-(ncol(varnames)-ncs)).  end if.  do if ((varnames(j)=wnames) and mcw <>0)).  compute mcwzcov=1.  end if.  do if ((varnames(j)=znames) and mcz=0).  compute match=0.  compute ziscov=(j-(ncol(varnames)-ncs)).  end if.  do if ((varnames(j)=znames) and mcz<>0)).  compute mcwzcov=1.  end if.  end if.  end if.  end loop.  end loop.  do if (match2>0 or match=1).  compute errcode(errs,1)=2.  compute errs=errs+1.  compute criterr=1.  end if.  do if (mcwzcov=1).  compute errcode(errs,1)=50.  compute errs=errs+1.  compute criterr=1.  end if.  compute ninit=nrow(dat).  compute rownum=make(ninit,1,0).  loop i = 1 to ninit.  compute rownum(i,1)=i.  end loop.  compute dat={rownum,dat}.  compute j=1.  compute missrow=0.  loop i = 1 to n.  do if (rsum(dat(i,2:ncol(dat))=99999)=0).  compute dat(j,:)=dat(i,:).  compute j=j+1.  else.  compute missrow={missrow;dat(i,1)}.  end if.  end loop.  compute rownum=dat(1:(j-1),1).  do if (nrow(missrow) > 1).  compute missrow=t(missrow(2:nrow(missrow),1)).  end if.  compute dat=dat(1:(j-1),2:ncol(dat)).  compute n=nrow(dat).  compute nmiss=ninit-n.  compute ytmp=dat(:,1:nys).  .  compute desctmp=make((8-(4* 0 )),ncol( ytmp ),-999).  loop jd=1 to ncol( ytmp ).  compute descdat= ytmp (:,jd).  compute desctmp(1,jd) = csum(descdat)/nrow(descdat).  compute desctmp(2,jd) = (nrow(descdat)*sscp(descdat))-(t(csum(descdat))*(csum(descdat))).  compute desctmp(2,jd) = sqrt(desctmp(2,jd)/(nrow(descdat)*(nrow(descdat)-1))).  compute desctmp(3,jd)=cmin(descdat).  compute desctmp(4,jd)=cmax(descdat).  do if ( 0 =0).  compute minwarn=0.  compute maxwarn=0.  do if ((desctmp(3,jd)=desctmp(4,jd)) and novar=0).  compute errcode(errs,1)=15.  compute errs=errs+1.  compute criterr=1.  compute novar=1.  end if.  compute tmp=((descdat(:,1)=desctmp(3,jd))+(descdat(:,1)=desctmp(4,jd))).  compute desctmp(8,jd)=(csum(tmp)=nrow(tmp)).  compute tmp = descdat.  compute tmp(GRADE(descdat),:) = descdat.  compute descdat = tmp.  release tmp.  compute decval={.16;.5;.84}.  loop kd=1 to 3.  compute low=trunc(decval(kd,1)*(nrow(descdat)+1)).  compute lowdec=decval(kd,1)*(nrow(descdat)+1)-low.  compute value=descdat(low,1)+(descdat((low+1),1)-descdat(low,1))*lowdec.  compute desctmp((4+kd),jd)=value.  end loop.  compute mnotev=1.  compute modvals=desctmp(5:7,:).  do if (quantile <> 1).  compute desctmp(5,jd)=desctmp(1,jd)-desctmp(2,jd).  compute desctmp(6,jd)=desctmp(1,jd).  compute desctmp(7,jd)=desctmp(1,jd)+desctmp(2,jd).  compute modvals=desctmp(5:7,:).  compute mnotev=2.  do if (modvals(1,1) < desctmp(3,1)).  compute modvals(1,1)=desctmp(3,1).  compute minwarn=1.  end if.  do if (modvals(3,1) > desctmp(4,1)).  compute modvals(3,1)=desctmp(4,1).  compute maxwarn=1.  end if.  end if.  do if (desctmp(8,1)=1).  compute modvals={desctmp(3,1);desctmp(4,1)}.  compute mnotev=0.  compute minwarn=0.  compute maxwarn=0.  end if.  end if.  end loop  .  compute ysd=desctmp(2,:).  compute ovsd=ysd.  compute ydich=0.  do if (desctmp(8,1)=1).  compute ydich=1.  do if (total=1).  compute total=0.  compute notecode(notes,1) = 24.  compute notes = notes + 1.  end if.  do if (effsize=1).  compute effsize=0.  compute notecode(notes,1) = 25.  compute notes = notes + 1.  end if.  compute omx = cmax(ytmp).  compute omn = cmin(ytmp).  compute ytmp = (ytmp = omx).  compute dat(:,1:nys)=(dat(:,1:nys)=omx).  compute rcd = {omn, 0; omx, 1}.  end if.  compute xtmp=dat(:,(nys+1):(nys+nxs)).  .  compute desctmp=make((8-(4* 0 )),ncol( xtmp ),-999).  loop jd=1 to ncol( xtmp ).  compute descdat= xtmp (:,jd).  compute desctmp(1,jd) = csum(descdat)/nrow(descdat).  compute desctmp(2,jd) = (nrow(descdat)*sscp(descdat))-(t(csum(descdat))*(csum(descdat))).  compute desctmp(2,jd) = sqrt(desctmp(2,jd)/(nrow(descdat)*(nrow(descdat)-1))).  compute desctmp(3,jd)=cmin(descdat).  compute desctmp(4,jd)=cmax(descdat).  do if ( 0 =0).  compute minwarn=0.  compute maxwarn=0.  do if ((desctmp(3,jd)=desctmp(4,jd)) and novar=0).  compute errcode(errs,1)=15.  compute errs=errs+1.  compute criterr=1.  compute novar=1.  end if.  compute tmp=((descdat(:,1)=desctmp(3,jd))+(descdat(:,1)=desctmp(4,jd))).  compute desctmp(8,jd)=(csum(tmp)=nrow(tmp)).  compute tmp = descdat.  compute tmp(GRADE(descdat),:) = descdat.  compute descdat = tmp.  release tmp.  compute decval={.16;.5;.84}.  loop kd=1 to 3.  compute low=trunc(decval(kd,1)*(nrow(descdat)+1)).  compute lowdec=decval(kd,1)*(nrow(descdat)+1)-low.  compute value=descdat(low,1)+(descdat((low+1),1)-descdat(low,1))*lowdec.  compute desctmp((4+kd),jd)=value.  end loop.  compute mnotev=1.  compute modvals=desctmp(5:7,:).  do if (quantile <> 1).  compute desctmp(5,jd)=desctmp(1,jd)-desctmp(2,jd).  compute desctmp(6,jd)=desctmp(1,jd).  compute desctmp(7,jd)=desctmp(1,jd)+desctmp(2,jd).  compute modvals=desctmp(5:7,:).  compute mnotev=2.  do if (modvals(1,1) < desctmp(3,1)).  compute modvals(1,1)=desctmp(3,1).  compute minwarn=1.  end if.  do if (modvals(3,1) > desctmp(4,1)).  compute modvals(3,1)=desctmp(4,1).  compute maxwarn=1.  end if.  end if.  do if (desctmp(8,1)=1).  compute modvals={desctmp(3,1);desctmp(4,1)}.  compute mnotev=0.  compute minwarn=0.  compute maxwarn=0.  end if.  end if.  end loop  .  compute xsd=desctmp(2,:).  compute xmodvals=modvals.  compute nxpval=nrow(xmodvals).  compute xprobval=xmodvals.  compute xdich=desctmp(8,1).  do if (xdich =1 and mcx > 0).  compute mcx=0.  compute errcode(errs,1) = 52.  compute errs = errs + 1.  compute criterr = 1.  end if.  do if (model = 74 and xdich=1).  compute counterf=1.  end if.  do if (nms > 0).  compute mtmp=dat(:,(nys+nxs+1):(nys+nxs+nms)).  .  compute desctmp=make((8-(4* 0 )),ncol( mtmp ),-999).  loop jd=1 to ncol( mtmp ).  compute descdat= mtmp (:,jd).  compute desctmp(1,jd) = csum(descdat)/nrow(descdat).  compute desctmp(2,jd) = (nrow(descdat)*sscp(descdat))-(t(csum(descdat))*(csum(descdat))).  compute desctmp(2,jd) = sqrt(desctmp(2,jd)/(nrow(descdat)*(nrow(descdat)-1))).  compute desctmp(3,jd)=cmin(descdat).  compute desctmp(4,jd)=cmax(descdat).  do if ( 0 =0).  compute minwarn=0.  compute maxwarn=0.  do if ((desctmp(3,jd)=desctmp(4,jd)) and novar=0).  compute errcode(errs,1)=15.  compute errs=errs+1.  compute criterr=1.  compute novar=1.  end if.  compute tmp=((descdat(:,1)=desctmp(3,jd))+(descdat(:,1)=desctmp(4,jd))).  compute desctmp(8,jd)=(csum(tmp)=nrow(tmp)).  compute tmp = descdat.  compute tmp(GRADE(descdat),:) = descdat.  compute descdat = tmp.  release tmp.  compute decval={.16;.5;.84}.  loop kd=1 to 3.  compute low=trunc(decval(kd,1)*(nrow(descdat)+1)).  compute lowdec=decval(kd,1)*(nrow(descdat)+1)-low.  compute value=descdat(low,1)+(descdat((low+1),1)-descdat(low,1))*lowdec.  compute desctmp((4+kd),jd)=value.  end loop.  compute mnotev=1.  compute modvals=desctmp(5:7,:).  do if (quantile <> 1).  compute desctmp(5,jd)=desctmp(1,jd)-desctmp(2,jd).  compute desctmp(6,jd)=desctmp(1,jd).  compute desctmp(7,jd)=desctmp(1,jd)+desctmp(2,jd).  compute modvals=desctmp(5:7,:).  compute mnotev=2.  do if (modvals(1,1) < desctmp(3,1)).  compute modvals(1,1)=desctmp(3,1).  compute minwarn=1.  end if.  do if (modvals(3,1) > desctmp(4,1)).  compute modvals(3,1)=desctmp(4,1).  compute maxwarn=1.  end if.  end if.  do if (desctmp(8,1)=1).  compute modvals={desctmp(3,1);desctmp(4,1)}.  compute mnotev=0.  compute minwarn=0.  compute maxwarn=0.  end if.  end if.  end loop  .  compute ovsd={desctmp(2,:),ysd}.  do if ((rsum(desctmp(8,:))>0) and (mdichok <> 1)).  compute errcode(errs,1)=43.  compute errs=errs+1.  compute criterr=1.  end if.  compute mmodvals=modvals.  compute mprobval=mmodvals.  end if.  do if (nws > 0).  compute wtmp=dat(:,(nys+nxs+nms+1):(nys+nxs+nms+nws)).  .  compute desctmp=make((8-(4* 0 )),ncol( wtmp ),-999).  loop jd=1 to ncol( wtmp ).  compute descdat= wtmp (:,jd).  compute desctmp(1,jd) = csum(descdat)/nrow(descdat).  compute desctmp(2,jd) = (nrow(descdat)*sscp(descdat))-(t(csum(descdat))*(csum(descdat))).  compute desctmp(2,jd) = sqrt(desctmp(2,jd)/(nrow(descdat)*(nrow(descdat)-1))).  compute desctmp(3,jd)=cmin(descdat).  compute desctmp(4,jd)=cmax(descdat).  do if ( 0 =0).  compute minwarn=0.  compute maxwarn=0.  do if ((desctmp(3,jd)=desctmp(4,jd)) and novar=0).  compute errcode(errs,1)=15.  compute errs=errs+1.  compute criterr=1.  compute novar=1.  end if.  compute tmp=((descdat(:,1)=desctmp(3,jd))+(descdat(:,1)=desctmp(4,jd))).  compute desctmp(8,jd)=(csum(tmp)=nrow(tmp)).  compute tmp = descdat.  compute tmp(GRADE(descdat),:) = descdat.  compute descdat = tmp.  release tmp.  compute decval={.16;.5;.84}.  loop kd=1 to 3.  compute low=trunc(decval(kd,1)*(nrow(descdat)+1)).  compute lowdec=decval(kd,1)*(nrow(descdat)+1)-low.  compute value=descdat(low,1)+(descdat((low+1),1)-descdat(low,1))*lowdec.  compute desctmp((4+kd),jd)=value.  end loop.  compute mnotev=1.  compute modvals=desctmp(5:7,:).  do if (quantile <> 1).  compute desctmp(5,jd)=desctmp(1,jd)-desctmp(2,jd).  compute desctmp(6,jd)=desctmp(1,jd).  compute desctmp(7,jd)=desctmp(1,jd)+desctmp(2,jd).  compute modvals=desctmp(5:7,:).  compute mnotev=2.  do if (modvals(1,1) < desctmp(3,1)).  compute modvals(1,1)=desctmp(3,1).  compute minwarn=1.  end if.  do if (modvals(3,1) > desctmp(4,1)).  compute modvals(3,1)=desctmp(4,1).  compute maxwarn=1.  end if.  end if.  do if (desctmp(8,1)=1).  compute modvals={desctmp(3,1);desctmp(4,1)}.  compute mnotev=0.  compute minwarn=0.  compute maxwarn=0.  end if.  end if.  end loop  .  compute wmodvals=modvals.  compute wdich=desctmp(8,1).  do if (wdich =1 and mcw > 0).  compute mcw=0.  compute errcode(errs,1) = 52.  compute errs = errs + 1.  compute criterr = 1.  end if.  compute wmin=desctmp(3,1).  compute wmax=desctmp(4,1).  compute minwwarn=minwarn.  compute maxwwarn=maxwarn.  compute wnotev=mnotev.  compute wmodval={ 999 }.  compute nwcontr=ncol(wmodval).  do if (wmodval(1,1) <> 999).  compute wmodvals=wmodval(1,1).  compute wmodcust=1.  do if (nwcontr > 1).  compute wmodvals=t(wmodval).  end if.  compute minwwarn=0.  compute maxwwarn=0.  compute wnotev=0.  end if.  compute wprobval=wmodvals.  compute nwpval=nrow(wmodvals).  end if.  do if (nzs > 0).  compute ztmp=dat(:,(nys+nxs+nms+nws+1):(nys+nxs+nms+nws+nzs)).  .  compute desctmp=make((8-(4* 0 )),ncol( ztmp ),-999).  loop jd=1 to ncol( ztmp ).  compute descdat= ztmp (:,jd).  compute desctmp(1,jd) = csum(descdat)/nrow(descdat).  compute desctmp(2,jd) = (nrow(descdat)*sscp(descdat))-(t(csum(descdat))*(csum(descdat))).  compute desctmp(2,jd) = sqrt(desctmp(2,jd)/(nrow(descdat)*(nrow(descdat)-1))).  compute desctmp(3,jd)=cmin(descdat).  compute desctmp(4,jd)=cmax(descdat).  do if ( 0 =0).  compute minwarn=0.  compute maxwarn=0.  do if ((desctmp(3,jd)=desctmp(4,jd)) and novar=0).  compute errcode(errs,1)=15.  compute errs=errs+1.  compute criterr=1.  compute novar=1.  end if.  compute tmp=((descdat(:,1)=desctmp(3,jd))+(descdat(:,1)=desctmp(4,jd))).  compute desctmp(8,jd)=(csum(tmp)=nrow(tmp)).  compute tmp = descdat.  compute tmp(GRADE(descdat),:) = descdat.  compute descdat = tmp.  release tmp.  compute decval={.16;.5;.84}.  loop kd=1 to 3.  compute low=trunc(decval(kd,1)*(nrow(descdat)+1)).  compute lowdec=decval(kd,1)*(nrow(descdat)+1)-low.  compute value=descdat(low,1)+(descdat((low+1),1)-descdat(low,1))*lowdec.  compute desctmp((4+kd),jd)=value.  end loop.  compute mnotev=1.  compute modvals=desctmp(5:7,:).  do if (quantile <> 1).  compute desctmp(5,jd)=desctmp(1,jd)-desctmp(2,jd).  compute desctmp(6,jd)=desctmp(1,jd).  compute desctmp(7,jd)=desctmp(1,jd)+desctmp(2,jd).  compute modvals=desctmp(5:7,:).  compute mnotev=2.  do if (modvals(1,1) < desctmp(3,1)).  compute modvals(1,1)=desctmp(3,1).  compute minwarn=1.  end if.  do if (modvals(3,1) > desctmp(4,1)).  compute modvals(3,1)=desctmp(4,1).  compute maxwarn=1.  end if.  end if.  do if (desctmp(8,1)=1).  compute modvals={desctmp(3,1);desctmp(4,1)}.  compute mnotev=0.  compute minwarn=0.  compute maxwarn=0.  end if.  end if.  end loop  .  compute zmodvals=modvals.  compute zdich=desctmp(8,1).  do if (zdich =1 and mcz > 0).  compute mcz=0.  compute errcode(errs,1) = 52.  compute errs = errs + 1.  compute criterr = 1.  end if.  compute zmin=desctmp(3,1).  compute zmax=desctmp(4,1).  compute minzwarn=minwarn.  compute maxzwarn=maxwarn.  compute znotev=mnotev.  compute zmodval={ 999 }.  compute nzcontr=ncol(zmodval).  do if (zmodval(1,1) <> 999).  compute zmodvals=zmodval(1,1).  compute zmodcust=1.  do if (nzcontr > 1).  compute zmodvals=t(zmodval).  end if.  compute minzwarn=0.  compute maxzwarn=0.  compute znotev=0.  end if.  compute zprobval=zmodvals.  compute nzpval=nrow(zmodvals).  end if.  do if (ncs > 0).  compute ctmp=dat(:,(nys+nxs+nms+nws+nzs+1):(nys+nxs+nms+nws+nzs+ncs)).  .  compute desctmp=make((8-(4* 0 )),ncol( ctmp ),-999).  loop jd=1 to ncol( ctmp ).  compute descdat= ctmp (:,jd).  compute desctmp(1,jd) = csum(descdat)/nrow(descdat).  compute desctmp(2,jd) = (nrow(descdat)*sscp(descdat))-(t(csum(descdat))*(csum(descdat))).  compute desctmp(2,jd) = sqrt(desctmp(2,jd)/(nrow(descdat)*(nrow(descdat)-1))).  compute desctmp(3,jd)=cmin(descdat).  compute desctmp(4,jd)=cmax(descdat).  do if ( 0 =0).  compute minwarn=0.  compute maxwarn=0.  do if ((desctmp(3,jd)=desctmp(4,jd)) and novar=0).  compute errcode(errs,1)=15.  compute errs=errs+1.  compute criterr=1.  compute novar=1.  end if.  compute tmp=((descdat(:,1)=desctmp(3,jd))+(descdat(:,1)=desctmp(4,jd))).  compute desctmp(8,jd)=(csum(tmp)=nrow(tmp)).  compute tmp = descdat.  compute tmp(GRADE(descdat),:) = descdat.  compute descdat = tmp.  release tmp.  compute decval={.16;.5;.84}.  loop kd=1 to 3.  compute low=trunc(decval(kd,1)*(nrow(descdat)+1)).  compute lowdec=decval(kd,1)*(nrow(descdat)+1)-low.  compute value=descdat(low,1)+(descdat((low+1),1)-descdat(low,1))*lowdec.  compute desctmp((4+kd),jd)=value.  end loop.  compute mnotev=1.  compute modvals=desctmp(5:7,:).  do if (quantile <> 1).  compute desctmp(5,jd)=desctmp(1,jd)-desctmp(2,jd).  compute desctmp(6,jd)=desctmp(1,jd).  compute desctmp(7,jd)=desctmp(1,jd)+desctmp(2,jd).  compute modvals=desctmp(5:7,:).  compute mnotev=2.  do if (modvals(1,1) < desctmp(3,1)).  compute modvals(1,1)=desctmp(3,1).  compute minwarn=1.  end if.  do if (modvals(3,1) > desctmp(4,1)).  compute modvals(3,1)=desctmp(4,1).  compute maxwarn=1.  end if.  end if.  do if (desctmp(8,1)=1).  compute modvals={desctmp(3,1);desctmp(4,1)}.  compute mnotev=0.  compute minwarn=0.  compute maxwarn=0.  end if.  end if.  end loop  .  end if.  compute n=nrow(ytmp).  compute ones=make(n,1,1).  release dat.  do if (nws > 0 and mcw > 0).  compute tmp={rownum,wtmp(:,1)}.  .  compute dd= tmp.  compute temp = dd.  compute temp(GRADE(dd(:,2)),:) = dd.  compute dd = temp.  compute dummy = design(dd(:,2)).  compute nvls = ncol(dummy).  compute nnvls = csum(dummy).  compute mnvls = cmin(t(nnvls)).  compute conmat1=1.  do if (mnvls < 2).  compute errcode(errs,1) = 5.  compute errs = errs + 1.  compute criterr = 1.  end if.  do if (nvls > 9).  compute errcode(errs,1) = 4.  compute errs = errs+1.  compute criterr = 1.  end if.  do if (criterr = 0).  compute dumok = 1.  compute nnvls=make(nvls,1,0).  compute nnvls(1,1)=dd(1,2).  compute temp = 2.  loop i = 2 to n.  do if (dd(i,2) <> nnvls((temp-1),1)).  compute nnvls(temp,1)=dd(i,2).  compute temp = temp+1.  end if.  end loop.  do if ( mcw > 0).  compute x = dummy(:,2:ncol(dummy)).  compute nx = ncol(x).  compute minus1 = make(1,ncol(x),-1).  compute xdes=make((nx+1),3,0).  compute xdes(1,1)=dd(1,2).  compute xdes(1,2)=1.  compute temp = 2.  loop k = 2 to n.  do if (dd(k,2) <> dd((k-1),2)).  compute xdes(temp,2) = k.  compute xdes(temp,1) = dd(k,2).  compute xdes((temp-1),3) = k-1.  compute temp=temp+1.  end if.  end loop.  compute xdes((temp-1),3)=n.  compute xdes = {xdes, (xdes(:,3)-xdes(:,2)+1)}.  do if ( mcw = 4).  loop k = 1 to n.  do if (rsum(x(k,:)) = 0).  compute x(k,:) = minus1.  end if.  end loop.  end if.  do if ( mcw = 2 or mcw = 3 or mcw =5).  loop k = 1 to n.  do if (rsum(x(k,:)) > 0).  loop i = 1 to ncol(x).  do if (x(k,i) = 0).  compute x(k,i) = 1.  else.  break.  end if.  end loop.  end if.  end loop.  do if ( mcw = 3).  compute conmat1={-8,1,1,1,1,1,1,1,1; 0,-7,1,1,1,1,1,1,1; 0,0,-6,1,1,1,1,1,1; 0,0,0,-5,1,1,1,1,1; 0,0,0,0,-4,1,1,1,1; 0,0,0,0,0,-3,1,1,1; 0,0,0,0,0,0,-2,1,1; 0,0,0,0,0,0,0,-1,1}.  loop i = 1 to 8.  compute conmat1(i,:)=conmat1(i,:)/(10-i).  end loop.  compute conmat1=t(conmat1((10-nvls):8,(10-nvls):9)).  loop k=1 to n.  compute x(k,:)=conmat1((rsum(x(k,:))+1),:).  end loop.  end if.  end if.  do if ( mcw = 5).  compute custcode={ -999 }.  do if (ncol(custcode) <> (nvls*(nvls-1))).  compute errcode(errs,1) = (37+ 2 ).  compute errs = errs + 1.  compute criterr = 1.  end if.  do if (ncol(custcode) = (nvls*(nvls-1))).  compute conmat1=make(nvls,(nvls-1),0).  compute cnt=1.  loop i = 1 to nvls.  loop k = 1 to (nvls-1).  compute conmat1(i,k)=custcode(1,cnt).  compute cnt=cnt+1.  end loop.  end loop.  loop k=1 to n.  compute x(k,:)=conmat1((rsum(x(k,:))+1),:).  end loop.  end if.  end if.  compute xskip = 1.  compute dummat = make((nx+1),nx,0).  compute dummat((2:nrow(dummat)),:)=ident(nx).  do if ( mcw = 4).  compute dummat(1,:) = minus1.  end if.  do if ( mcw = 2).  loop i = 2 to nrow(dummat).  loop j = 1 to (i-1).  compute dummat(i,j) = 1.  end loop.  end loop.  end if.  do if ( mcw = 3).  compute dummat=conmat1.  end if.  do if ( mcw = 5 and criterr=0).  compute dummat=conmat1.  end if.  compute dummat={nnvls, dummat}.  compute x={dd(:,1),x}.  compute temp = x.  compute temp(GRADE(x(:,1)),:) = x.  compute x = temp.  release conmat1,temp,dd,xskip,xdes,dummy.  end if.  end if  .  compute wmodvals=nnvls.  compute nwpval=nrow(wmodvals).  do if (criterr=0).  compute minwwarn=0.  compute maxwwarn=0.  compute wnotev=0.  compute wtmp=x(:,2:ncol(x)).  compute wcatlab={'W1';'W2';'W3';'W4';'W5';'W6';'W7';'W8';'W9'}.  compute nwvls=nvls-1.  compute mcwok=1.  compute dummatw=dummat.  compute wprobval=dummatw(:,2:ncol(dummatw)).  do if (modcok=1).  compute wcontval=make(2,ncol(wprobval),-999).  compute temp=0.  loop i = 1 to 2.  loop j = 1 to nrow(dummatw).  do if (contvec(i,1)=dummatw(j,1)).  compute wcontval(i,:)=wprobval(j,:).  compute temp=temp+1.  end if.  end loop.  end loop.  do if (temp < 2).  compute notecode(notes,1) = 20.  compute notes = notes + 1.  compute modcok=0.  end if.  end if.  do if (wmodval(1,1) <> 999).  compute notecode(notes,1) = 9.  compute notes = notes + 1.  end if.  release tmp, dummat.  end if.  end if.  do if (nzs > 0 and mcz > 0).  compute tmp={rownum,ztmp(:,1)}.  .  compute dd= tmp.  compute temp = dd.  compute temp(GRADE(dd(:,2)),:) = dd.  compute dd = temp.  compute dummy = design(dd(:,2)).  compute nvls = ncol(dummy).  compute nnvls = csum(dummy).  compute mnvls = cmin(t(nnvls)).  compute conmat1=1.  do if (mnvls < 2).  compute errcode(errs,1) = 5.  compute errs = errs + 1.  compute criterr = 1.  end if.  do if (nvls > 9).  compute errcode(errs,1) = 4.  compute errs = errs+1.  compute criterr = 1.  end if.  do if (criterr = 0).  compute dumok = 1.  compute nnvls=make(nvls,1,0).  compute nnvls(1,1)=dd(1,2).  compute temp = 2.  loop i = 2 to n.  do if (dd(i,2) <> nnvls((temp-1),1)).  compute nnvls(temp,1)=dd(i,2).  compute temp = temp+1.  end if.  end loop.  do if ( mcz > 0).  compute x = dummy(:,2:ncol(dummy)).  compute nx = ncol(x).  compute minus1 = make(1,ncol(x),-1).  compute xdes=make((nx+1),3,0).  compute xdes(1,1)=dd(1,2).  compute xdes(1,2)=1.  compute temp = 2.  loop k = 2 to n.  do if (dd(k,2) <> dd((k-1),2)).  compute xdes(temp,2) = k.  compute xdes(temp,1) = dd(k,2).  compute xdes((temp-1),3) = k-1.  compute temp=temp+1.  end if.  end loop.  compute xdes((temp-1),3)=n.  compute xdes = {xdes, (xdes(:,3)-xdes(:,2)+1)}.  do if ( mcz = 4).  loop k = 1 to n.  do if (rsum(x(k,:)) = 0).  compute x(k,:) = minus1.  end if.  end loop.  end if.  do if ( mcz = 2 or mcz = 3 or mcz =5).  loop k = 1 to n.  do if (rsum(x(k,:)) > 0).  loop i = 1 to ncol(x).  do if (x(k,i) = 0).  compute x(k,i) = 1.  else.  break.  end if.  end loop.  end if.  end loop.  do if ( mcz = 3).  compute conmat1={-8,1,1,1,1,1,1,1,1; 0,-7,1,1,1,1,1,1,1; 0,0,-6,1,1,1,1,1,1; 0,0,0,-5,1,1,1,1,1; 0,0,0,0,-4,1,1,1,1; 0,0,0,0,0,-3,1,1,1; 0,0,0,0,0,0,-2,1,1; 0,0,0,0,0,0,0,-1,1}.  loop i = 1 to 8.  compute conmat1(i,:)=conmat1(i,:)/(10-i).  end loop.  compute conmat1=t(conmat1((10-nvls):8,(10-nvls):9)).  loop k=1 to n.  compute x(k,:)=conmat1((rsum(x(k,:))+1),:).  end loop.  end if.  end if.  do if ( mcz = 5).  compute custcode={ -999 }.  do if (ncol(custcode) <> (nvls*(nvls-1))).  compute errcode(errs,1) = (37+ 3 ).  compute errs = errs + 1.  compute criterr = 1.  end if.  do if (ncol(custcode) = (nvls*(nvls-1))).  compute conmat1=make(nvls,(nvls-1),0).  compute cnt=1.  loop i = 1 to nvls.  loop k = 1 to (nvls-1).  compute conmat1(i,k)=custcode(1,cnt).  compute cnt=cnt+1.  end loop.  end loop.  loop k=1 to n.  compute x(k,:)=conmat1((rsum(x(k,:))+1),:).  end loop.  end if.  end if.  compute xskip = 1.  compute dummat = make((nx+1),nx,0).  compute dummat((2:nrow(dummat)),:)=ident(nx).  do if ( mcz = 4).  compute dummat(1,:) = minus1.  end if.  do if ( mcz = 2).  loop i = 2 to nrow(dummat).  loop j = 1 to (i-1).  compute dummat(i,j) = 1.  end loop.  end loop.  end if.  do if ( mcz = 3).  compute dummat=conmat1.  end if.  do if ( mcz = 5 and criterr=0).  compute dummat=conmat1.  end if.  compute dummat={nnvls, dummat}.  compute x={dd(:,1),x}.  compute temp = x.  compute temp(GRADE(x(:,1)),:) = x.  compute x = temp.  release conmat1,temp,dd,xskip,xdes,dummy.  end if.  end if  .  compute zmodvals=nnvls.  compute nzpval=nrow(zmodvals).  do if (criterr=0).  compute minzwarn=0.  compute maxzwarn=0.  compute znotev=0.  compute ztmp=x(:,2:ncol(x)).  compute zcatlab={'Z1';'Z2';'Z3';'Z4';'Z5';'Z6';'Z7';'Z8';'Z9'}.  compute nzvls=nvls-1.  compute mczok=1.  compute dummatz=dummat.  compute zprobval=dummatz(:,2:ncol(dummatz)).  do if (modcok=1).  compute zcontval=make(2,ncol(zprobval),-999).  compute temp=0.  loop i = 1 to 2.  loop j = 1 to nrow(dummatz).  do if (contvec(i,2)=dummatz(j,1)).  compute zcontval(i,:)=zprobval(j,:).  compute temp=temp+1.  end if.  end loop.  end loop.  do if (temp < 2).  compute notecode(notes,1) = 20.  compute notes = notes + 1.  compute modcok=0.  end if.  end if.  do if (zmodval(1,1) <> 999).  compute notecode(notes,1) = 10.  compute notes = notes + 1.  end if.  release tmp, dummat.  end if.  end if.  do if (nxs > 0 and mcx > 0).  compute tmp={rownum,xtmp(:,1)}.  .  compute dd= tmp.  compute temp = dd.  compute temp(GRADE(dd(:,2)),:) = dd.  compute dd = temp.  compute dummy = design(dd(:,2)).  compute nvls = ncol(dummy).  compute nnvls = csum(dummy).  compute mnvls = cmin(t(nnvls)).  compute conmat1=1.  do if (mnvls < 2).  compute errcode(errs,1) = 5.  compute errs = errs + 1.  compute criterr = 1.  end if.  do if (nvls > 9).  compute errcode(errs,1) = 4.  compute errs = errs+1.  compute criterr = 1.  end if.  do if (criterr = 0).  compute dumok = 1.  compute nnvls=make(nvls,1,0).  compute nnvls(1,1)=dd(1,2).  compute temp = 2.  loop i = 2 to n.  do if (dd(i,2) <> nnvls((temp-1),1)).  compute nnvls(temp,1)=dd(i,2).  compute temp = temp+1.  end if.  end loop.  do if ( mcx > 0).  compute x = dummy(:,2:ncol(dummy)).  compute nx = ncol(x).  compute minus1 = make(1,ncol(x),-1).  compute xdes=make((nx+1),3,0).  compute xdes(1,1)=dd(1,2).  compute xdes(1,2)=1.  compute temp = 2.  loop k = 2 to n.  do if (dd(k,2) <> dd((k-1),2)).  compute xdes(temp,2) = k.  compute xdes(temp,1) = dd(k,2).  compute xdes((temp-1),3) = k-1.  compute temp=temp+1.  end if.  end loop.  compute xdes((temp-1),3)=n.  compute xdes = {xdes, (xdes(:,3)-xdes(:,2)+1)}.  do if ( mcx = 4).  loop k = 1 to n.  do if (rsum(x(k,:)) = 0).  compute x(k,:) = minus1.  end if.  end loop.  end if.  do if ( mcx = 2 or mcx = 3 or mcx =5).  loop k = 1 to n.  do if (rsum(x(k,:)) > 0).  loop i = 1 to ncol(x).  do if (x(k,i) = 0).  compute x(k,i) = 1.  else.  break.  end if.  end loop.  end if.  end loop.  do if ( mcx = 3).  compute conmat1={-8,1,1,1,1,1,1,1,1; 0,-7,1,1,1,1,1,1,1; 0,0,-6,1,1,1,1,1,1; 0,0,0,-5,1,1,1,1,1; 0,0,0,0,-4,1,1,1,1; 0,0,0,0,0,-3,1,1,1; 0,0,0,0,0,0,-2,1,1; 0,0,0,0,0,0,0,-1,1}.  loop i = 1 to 8.  compute conmat1(i,:)=conmat1(i,:)/(10-i).  end loop.  compute conmat1=t(conmat1((10-nvls):8,(10-nvls):9)).  loop k=1 to n.  compute x(k,:)=conmat1((rsum(x(k,:))+1),:).  end loop.  end if.  end if.  do if ( mcx = 5).  compute custcode={ -999 }.  do if (ncol(custcode) <> (nvls*(nvls-1))).  compute errcode(errs,1) = (37+ 1 ).  compute errs = errs + 1.  compute criterr = 1.  end if.  do if (ncol(custcode) = (nvls*(nvls-1))).  compute conmat1=make(nvls,(nvls-1),0).  compute cnt=1.  loop i = 1 to nvls.  loop k = 1 to (nvls-1).  compute conmat1(i,k)=custcode(1,cnt).  compute cnt=cnt+1.  end loop.  end loop.  loop k=1 to n.  compute x(k,:)=conmat1((rsum(x(k,:))+1),:).  end loop.  end if.  end if.  compute xskip = 1.  compute dummat = make((nx+1),nx,0).  compute dummat((2:nrow(dummat)),:)=ident(nx).  do if ( mcx = 4).  compute dummat(1,:) = minus1.  end if.  do if ( mcx = 2).  loop i = 2 to nrow(dummat).  loop j = 1 to (i-1).  compute dummat(i,j) = 1.  end loop.  end loop.  end if.  do if ( mcx = 3).  compute dummat=conmat1.  end if.  do if ( mcx = 5 and criterr=0).  compute dummat=conmat1.  end if.  compute dummat={nnvls, dummat}.  compute x={dd(:,1),x}.  compute temp = x.  compute temp(GRADE(x(:,1)),:) = x.  compute x = temp.  release conmat1,temp,dd,xskip,xdes,dummy.  end if.  end if  .  do if (criterr=0).  compute xtmp=x(:,2:ncol(x)).  compute xcatlab={'X1';'X2';'X3';'X4';'X5';'X6';'X7';'X8';'X9'}.  compute nxvls=nvls-1.  compute xdich=(nvls=2).  compute mcxok=1.  compute dummatx=dummat.  compute xmodvals=dummatx(:,1).  compute nxpval=nrow(xmodvals).  release tmp, dummat.  end if.  end if.  compute intlab=make(100,1,' ').  compute intlab( 1 ,1)= 'Int_1'.  compute intlab( 2 ,1)= 'Int_2'.  compute intlab( 3 ,1)= 'Int_3'.  compute intlab( 4 ,1)= 'Int_4'.  compute intlab( 5 ,1)= 'Int_5'.  compute intlab( 6 ,1)= 'Int_6'.  compute intlab( 7 ,1)= 'Int_7'.  compute intlab( 8 ,1)= 'Int_8'.  compute intlab( 9 ,1)= 'Int_9'.  compute intlab( 10 ,1)= 'Int_10'.  compute intlab( 11 ,1)= 'Int_11'.  compute intlab( 12 ,1)= 'Int_12'.  compute intlab( 13 ,1)= 'Int_13'.  compute intlab( 14 ,1)= 'Int_14'.  compute intlab( 15 ,1)= 'Int_15'.  compute intlab( 16 ,1)= 'Int_16'.  compute intlab( 17 ,1)= 'Int_17'.  compute intlab( 18 ,1)= 'Int_18'.  compute intlab( 19 ,1)= 'Int_19'.  compute intlab( 20 ,1)= 'Int_20'.  compute intlab( 21 ,1)= 'Int_21'.  compute intlab( 22 ,1)= 'Int_22'.  compute intlab( 23 ,1)= 'Int_23'.  compute intlab( 24 ,1)= 'Int_24'.  compute intlab( 25 ,1)= 'Int_25'.  compute intlab( 26 ,1)= 'Int_26'.  compute intlab( 27 ,1)= 'Int_27'.  compute intlab( 28 ,1)= 'Int_28'.  compute intlab( 29 ,1)= 'Int_29'.  compute intlab( 30 ,1)= 'Int_30'.  compute intlab( 31 ,1)= 'Int_31'.  compute intlab( 32 ,1)= 'Int_32'.  compute intlab( 33 ,1)= 'Int_33'.  compute intlab( 34 ,1)= 'Int_34'.  compute intlab( 35 ,1)= 'Int_35'.  compute intlab( 36 ,1)= 'Int_36'.  compute intlab( 37 ,1)= 'Int_37'.  compute intlab( 38 ,1)= 'Int_38'.  compute intlab( 39 ,1)= 'Int_39'.  compute intlab( 40 ,1)= 'Int_40'.  compute intlab( 41 ,1)= 'Int_41'.  compute intlab( 42 ,1)= 'Int_42'.  compute intlab( 43 ,1)= 'Int_43'.  compute intlab( 44 ,1)= 'Int_44'.  compute intlab( 45 ,1)= 'Int_45'.  compute intlab( 46 ,1)= 'Int_46'.  compute intlab( 47 ,1)= 'Int_47'.  compute intlab( 48 ,1)= 'Int_48'.  compute intlab( 49 ,1)= 'Int_49'.  compute intlab( 50 ,1)= 'Int_50'.  compute intlab( 51 ,1)= 'Int_51'.  compute intlab( 52 ,1)= 'Int_52'.  compute intlab( 53 ,1)= 'Int_53'.  compute intlab( 54 ,1)= 'Int_54'.  compute intlab( 55 ,1)= 'Int_55'.  compute intlab( 56 ,1)= 'Int_56'.  compute intlab( 57 ,1)= 'Int_57'.  compute intlab( 58 ,1)= 'Int_58'.  compute intlab( 59 ,1)= 'Int_59'.  compute intlab( 60 ,1)= 'Int_60'.  compute intlab( 61 ,1)= 'Int_61'.  compute intlab( 62 ,1)= 'Int_62'.  compute intlab( 63 ,1)= 'Int_63'.  compute intlab( 64 ,1)= 'Int_64'.  compute intlab( 65 ,1)= 'Int_65'.  compute intlab( 66 ,1)= 'Int_66'.  compute intlab( 67 ,1)= 'Int_67'.  compute intlab( 68 ,1)= 'Int_68'.  compute intlab( 69 ,1)= 'Int_69'.  compute intlab( 70 ,1)= 'Int_70'.  compute intlab( 71 ,1)= 'Int_71'.  compute intlab( 72 ,1)= 'Int_72'.  compute intlab( 73 ,1)= 'Int_73'.  compute intlab( 74 ,1)= 'Int_74'.  compute intlab( 75 ,1)= 'Int_75'.  compute intlab( 76 ,1)= 'Int_76'.  compute intlab( 77 ,1)= 'Int_77'.  compute intlab( 78 ,1)= 'Int_78'.  compute intlab( 79 ,1)= 'Int_79'.  compute intlab( 80 ,1)= 'Int_80'.  compute intlab( 81 ,1)= 'Int_81'.  compute intlab( 82 ,1)= 'Int_82'.  compute intlab( 83 ,1)= 'Int_83'.  compute intlab( 84 ,1)= 'Int_84'.  compute intlab( 85 ,1)= 'Int_85'.  compute intlab( 86 ,1)= 'Int_86'.  compute intlab( 87 ,1)= 'Int_87'.  compute intlab( 88 ,1)= 'Int_88'.  compute intlab( 89 ,1)= 'Int_89'.  compute intlab( 90 ,1)= 'Int_90'.  compute intlab( 91 ,1)= 'Int_91'.  compute intlab( 92 ,1)= 'Int_92'.  compute intlab( 93 ,1)= 'Int_93'.  compute intlab( 94 ,1)= 'Int_94'.  compute intlab( 95 ,1)= 'Int_95'.  compute intlab( 96 ,1)= 'Int_96'.  compute intlab( 97 ,1)= 'Int_97'.  compute intlab( 98 ,1)= 'Int_98'.  compute intlab( 99 ,1)= 'Int_99'.  compute intlab( 100 ,1)= 'Int_100'.  compute bcmat=make(needed,needed,0).  compute wcmat=make(needed,needed,0).  compute zcmat=make(needed,needed,0).  compute wzcmat=make(needed,needed,0).  compute wsum=0.  compute zsum=0.  compute wzsum=0.  end if.  do if (criterr = 0 and model <> 999).  compute modelmat= {1,0,0,0,0,0,0,1,0,0;2,0,0,0,0,0,0,1,1,0;3,0,0,0,0,0,0,1,1,1;4,0,0,0,0,0,0,0,0,0; 5,0,0,0,0,0,0,1,0,0;6,0,0,0,0,0,0,0,0,0;7,1,0,0,0,0,0,0,0,0;8,1,0,0,0,0,0,1,0,0; 9,1,1,0,0,0,0,0,0,0;10,1,1,0,0,0,0,1,1,0;11,1,1,1,0,0,0,0,0,0;12,1,1,1,0,0,0,1,1,1; 13,1,1,1,0,0,0,1,0,0;14,0,0,0,1,0,0,0,0,0;15,0,0,0,1,0,0,1,0,0;16,0,0,0,1,1,0,0,0,0; 17,0,0,0,1,1,0,1,1,0;18,0,0,0,1,1,1,0,0,0;19,0,0,0,1,1,1,1,1,1;20,0,0,0,1,1,1,1,0,0; 21,1,0,0,0,1,0,0,0,0;22,1,0,0,0,1,0,1,0,0;23,0,0,0,0,0,0,0,0,0;24,0,0,0,0,0,0,0,0,0; 25,0,0,0,0,0,0,0,0,0;26,0,0,0,0,0,0,0,0,0;27,0,0,0,0,0,0,0,0,0;28,1,0,0,0,1,0,0,1,0; 29,1,0,0,0,1,0,1,1,0;30,0,0,0,0,0,0,0,0,0;31,0,0,0,0,0,0,0,0,0;32,0,0,0,0,0,0,0,0,0; 33,0,0,0,0,0,0,0,0,0;34,0,0,0,0,0,0,0,0,0;35,0,0,0,0,0,0,0,0,0;36,0,0,0,0,0,0,0,0,0; 37,0,0,0,0,0,0,0,0,0;38,0,0,0,0,0,0,0,0,0;39,0,0,0,0,0,0,0,0,0;40,0,0,0,0,0,0,0,0,0; 41,0,0,0,0,0,0,0,0,0;42,0,0,0,0,0,0,0,0,0;43,0,0,0,0,0,0,0,0,0;44,0,0,0,0,0,0,0,0,0; 45,0,0,0,0,0,0,0,0,0;46,0,0,0,0,0,0,0,0,0;47,0,0,0,0,0,0,0,0,0;48,0,0,0,0,0,0,0  ,0,0; 49,0,0,0,0,0,0,0,0,0;50,0,0,0,0,0,0,0,0,0;51,0,0,0,0,0,0,0,0,0;52,0,0,0,0,0,0,0,0,0; 53,0,0,0,0,0,0,0,0,0;54,0,0,0,0,0,0,0,0,0;55,0,0,0,0,0,0,0,0,0;56,0,0,0,0,0,0,0,0,0; 57,0,0,0,0,0,0,0,0,0;58,1,0,0,1,0,0,0,0,0;59,1,0,0,1,0,0,1,0,0;60,1,1,0,1,0,0,0,0,0; 61,1,1,0,1,0,0,1,0,0;62,1,1,0,1,0,0,0,1,0;63,1,1,0,1,0,0,1,1,0;64,1,0,0,1,1,0,0,0,0; 65,1,0,0,1,1,0,1,0,0;66,1,0,0,1,1,0,0,1,0;67,1,0,0,1,1,0,1,1,0;68,1,1,1,1,0,0,0,0,0; 69,1,1,1,1,0,0,1,1,1;70,1,0,0,1,1,1,0,0,0;71,1,0,0,1,1,1,1,1,1;72,1,1,1,1,1,1,0,0,0; 73,1,1,1,1,1,1,1,1,1;74,0,0,0,1,0,0,0,0,0;75,1,1,0,1,1,0,0,0,0;76,1,1,0,1,1,0,1,1,0; 77,0,0,0,0,0,0,0,0,0;78,0,0,0,0,0,0,0,0,0;79,0,0,0,0,0,0,0,0,0;80,0,0,0,0,0,0,0,0,0; 81,0,0,0,0,0,0,0,0,0;82,0,0,0,0,0,0,0,0,0;83,1,0,0,0,0,0,0,0,0;84,1,0,0,0,0,0,0,0,0; 85,1,0,0,0,0,0,1,0,0;86,1,0,0,0,0,0,1,0,0;87,0,0,0,1,0,0,0,0,0;88,0,0,0,1,0,0,0,0,0; 89,0,0,0,1,0,0,1,0,0;90,0,0,0,1,0,0,1,0,0;91,0,0,0,0,0,0,0,0,0;92,1,0,0,1,0,0,1,0,0}.  compute tmp=modelmat(model,2:ncol(modelmat)).  do if (model < 4).  compute bcmat((nxs+1),1)=1.  end if.  do if ((model > 3) and (model <> 6)).  compute bcmat((nxs+1):(nxs+nms),1)=onem.  compute bcmat(nrow(bcmat),(nxs+1):(nxs+nms))=t(onem).  compute bcmat(nrow(bcmat),1)=1.  end if.  do if ((model = 6) or (model > 82 and model < 93)).  loop j = 2 to nrow(bcmat).  loop i = 1 to (j-1).  compute bcmat(j,i)=1.  end loop.  end loop.  end if.  do if (model = 80).  loop i = 1 to nms.  compute bcmat((nrow(bcmat)-1),i)=1.  end loop.  end if.  do if (model = 81).  loop j = 3 to nrow(bcmat).  compute bcmat(j,2)=1.  end loop.  end if.  do if (model = 82).  compute bcmat(3,2)=1.  compute bcmat(5,4)=1.  end if.  do if (tmp(1,1)=1).  compute wcmat((nxs+1):(nxs+nms),1)=onem.  compute wprod=1.  compute xprod=1.  do if (model = 83 or model = 86).  compute onemsx=onem.  loop i = 1 to (nms-1).  compute onemsx(i+1,1)=0.  end loop.  compute wcmat((nxs+1):(nxs+nms),1)=onemsx.  end if.  end if.  do if (tmp(1,4)=1).  compute wcmat(nrow(wcmat),(nxs+1):(nxs+nms))=t(onem).  compute wprod=1.  do if (model = 87 or model = 90).  compute onemsx=onem.  loop i = 1 to (nms-1).  compute onemsx(i,1)=0.  end loop.  compute wcmat(nrow(wcmat),(nxs+1):(nxs+nms))=t(onemsx).  end if.  end if.  do if (tmp(1,7)=1).  compute wcmat(nrow(wcmat),1)=1.  compute wprod=1.  compute xprod=1.  end if.  do if (tmp(1,2)=1).  compute zcmat((nxs+1):(nxs+nms),1)=onem.  compute zprod=1.  compute xprod=1.  end if.  do if (tmp(1,5)=1).  compute zcmat(nrow(zcmat),(nxs+1):(nxs+nms))=t(onem).  compute zprod=1.  end if.  do if (tmp(1,8)=1).  compute zcmat(nrow(zcmat),1)=1.  compute zprod=1.  compute xprod=1.  end if.  do if (tmp(1,3)=1).  compute wzcmat((nxs+1):(nxs+nms),1)=onem.  compute xprod=1.  compute wprod=1.  compute zprod=1.  end if.  do if (tmp(1,6)=1).  compute wzcmat(nrow(wzcmat),(nxs+1):(nxs+nms))=t(onem).  compute zprod=1.  compute wprod=1.  end if.  do if (tmp(1,9)=1).  compute wzcmat(nrow(wzcmat),1)=1.  compute xprod=1.  compute wprod=1.  compute zprod=1.  end if.  do if (model = 91 or model = 92).  loop j = 1 to (nms-1).  loop i = 1 to j.  compute wcmat((nxs+1+j),(nxs+i))=1.  end loop.  end loop.  end if.  do if (nms < 0).  loop i = 1 to nms.  compute tmp=csum(wcmat(:,(1+i)))+csum(zcmat(:,(1+i)))+csum(wzcmat(:,(1+i))).  compute mprod(1,i)=(tmp>0).  end loop.  end if.  end if.  do if (ncs > 0).  compute ccmat=make((nms+nys),ncs,1).  compute ccmatoff=ccmat.  do if (covmy=1).  compute ccmat(nrow(ccmat),:)=make(1,ncs,0).  end if.  do if (covmy=2).  compute ccmat(1:nms,:)=make(nms,ncs,0).  end if.  do if (cmatrix(1,1) <> -999).  do if (ncol(cmatrix) <> ((nms+nys)*ncs)).  compute errcode(errs,1)=29.  compute errs=errs+1.  compute criterr=1.  end if.  do if (criterr = 0).  compute tmp=1.  loop i = 1 to (nms+nys).  loop j = 1 to ncs.  compute ccmat(i,j)=1-(cmatrix(1,tmp) = 0).  compute tmp=tmp+1.  end loop.  end loop.  do if (rsum((csum(ccmat)=0)) <> 0).  compute errcode(errs,1)=30.  compute errs=errs+1.  compute criterr=1.  end if.  end if.  do if (covmy <> 0).  compute notecode(notes,1)=1.  compute notes=notes+1.  end if.  end if.  end if.  do if (criterr=0).  compute needed=needed*(needed-1)/2.  compute nopath=0.  do if (bmatrix(1,1) <> -999).  compute tmp=1.  do if ((ncol(bmatrix) <> needed) or (csum(rsum(bmatrix))=0)).  compute errcode(errs,1)=16.  compute errs=errs+1.  compute criterr=1.  else.  loop i = 2 to nrow(bcmat).  loop j = 1 to (i-1).  compute bcmat(i,j)=1-(bmatrix(1,tmp) = 0).  compute tmp=tmp+1.  end loop.  end loop.  end if.  do if ((csum(bcmat(:,1))=0) and criterr=0).  compute errcode(errs,1)=22.  compute errs=errs+1.  compute criterr=1.  end if.  do if ((rsum(bcmat(nrow(bcmat),:))=0) and criterr=0).  compute errcode(errs,1)=23.  compute errs=errs+1.  compute criterr=1.  end if.  compute dm=0.  do if (nms > 0).  loop i = 1 to nms.  do if (((rsum(bcmat((nxs+i),:)) = 0) or (csum(bcmat(:,(nxs+i))) = 0)) and (dm=0) and (criterr=0)).  compute errcode(errs,1)=26.  compute errs=errs+1.  compute criterr=1.  compute dm=1.  end if.  end loop.  end if.  release dm.  end if.  end if.  do if (criterr=0).  do if (wmatrix(1,1) <> -999).  compute tmp=1.  do if (ncol(wmatrix) <> needed).  compute errcode(errs,1)=17.  compute errs=errs+1.  compute criterr=1.  else.  compute modelvar(1,1)='CUSTOM'.  loop i = 2 to nrow(wcmat).  loop j = 1 to (i-1).  compute wcmat(i,j)=1-(wmatrix(1,tmp) = 0).  do if ((wcmat(i,j)=1) and (bcmat(i,j)=0) and (nopath=0)).  compute errcode(errs,1)=20.  compute errs=errs+1.  compute criterr=1.  compute nopath=1.  end if.  compute tmp=tmp+1.  end loop.  end loop.  end if.  end if.  do if (zmatrix(1,1) <> -999).  compute tmp=1.  do if (ncol(zmatrix) <> needed).  compute errcode(errs,1)=18.  compute errs=errs+1.  compute criterr=1.  else.  compute modelvar(1,1)='CUSTOM'.  do if (csum(rsum(wcmat))=0 and model=999).  compute errcode(errs,1)=21.  compute errs=errs+1.  compute criterr=1.  end if.  loop i = 2 to nrow(zcmat).  loop j = 1 to (i-1).  compute zcmat(i,j)=1-(zmatrix(1,tmp) = 0).  do if ((zcmat(i,j)=1) and (bcmat(i,j)=0) and (nopath=0)).  compute errcode(errs,1)=20.  compute errs=errs+1.  compute criterr=1.  compute nopath=1.  end if.  compute tmp=tmp+1.  end loop.  end loop.  end if.  end if.  compute tmp=1.  do if (wzmatrix(1,1) <> -999).  do if (ncol(wzmatrix) <> needed).  compute errcode(errs,1)=19.  compute errs=errs+1.  compute criterr=1.  end if.  compute modelvar(1,1)='CUSTOM'.  end if.  do if (criterr=0).  loop i = 2 to nrow(wzcmat).  loop j = 1 to (i-1).  do if (wzmatrix(1,1) <> -999).  compute wzcmat(i,j)=1-(wzmatrix(1,tmp) = 0).  end if.  do if (wzcmat(i,j)=1).  compute wcmat(i,j)=1.  compute zcmat(i,j)=1.  end if.  do if ((wzcmat(i,j)=1) and (bcmat(i,j)=0) and (nopath=0)).  compute errcode(errs,1)=20.  compute errs=errs+1.  compute criterr=1.  compute nopath=1.  end if.  compute tmp=tmp+1.  end loop.  end loop.  end if.  end if.  do if (criterr=0).  compute xprod=csum(wcmat(:,1))+csum(zcmat(:,1))+csum(wzcmat(:,1)).  compute xprod=(xprod > 0).  compute wsum=csum(rsum(wcmat)).  compute wprod=(wsum > 0).  do if (nms > 0).  loop i = 1 to nms.  compute tmp=csum(wcmat(:,(1+i)))+csum(zcmat(:,(1+i)))+csum(wzcmat(:,(1+i))).  compute mprod(1,i)=(tmp>0).  end loop.  end if.  do if ((wsum > 0) and (w = 'xxxxx')).  compute errcode(errs,1)=11.  compute errs=errs+1.  compute criterr=1.  end if.  do if ((wsum = 0) and (w <> 'xxxxx')).  compute errcode(errs,1)=10.  compute errs=errs+1.  compute criterr=1.  end if.  compute zsum=csum(rsum(zcmat)).  compute zprod=(zsum > 0).  do if ((zsum > 0) and (z = 'xxxxx')).  compute errcode(errs,1)=13.  compute errs=errs+1.  compute criterr=1.  end if.  do if ((zsum = 0) and (z <> 'xxxxx')).  compute errcode(errs,1)=12.  compute errs=errs+1.  compute criterr=1.  end if.  do if ((zsum > 0) and (wsum = 0)).  compute errcode(errs,1)=35.  compute errs=errs+1.  compute criterr=1.  end if.  end if.  do if (criterr=0 and nms > 1).  compute serchk=bcmat(2:(nrow(bcmat)-1),2:ncol(bcmat)).  do if (csum(rsum(serchk))) > 0.  compute serial=1.  do if (nms > 6).  compute errcode(errs,1)=36.  compute errs=errs+1.  compute criterr=1.  end if.  end if.  end if.  do if (center > 0 and criterr=0).  compute centvar={' '}.  do if (criterr=0).  do if ((center = 1) or (center = 2 and wdich = 0)).  do if (wprod=1 and mcwok=0 and nwpval > 0).  loop i = 1 to nws.  compute wtmp(:,i)=wtmp(:,i)-(csum(wtmp(:,i))/n).  compute centvar={centvar,wnames(1,i)}.  end loop.  .  compute desctmp=make((8-(4* wmodcust )),ncol( wtmp ),-999).  loop jd=1 to ncol( wtmp ).  compute descdat= wtmp (:,jd).  compute desctmp(1,jd) = csum(descdat)/nrow(descdat).  compute desctmp(2,jd) = (nrow(descdat)*sscp(descdat))-(t(csum(descdat))*(csum(descdat))).  compute desctmp(2,jd) = sqrt(desctmp(2,jd)/(nrow(descdat)*(nrow(descdat)-1))).  compute desctmp(3,jd)=cmin(descdat).  compute desctmp(4,jd)=cmax(descdat).  do if ( wmodcust =0).  compute minwarn=0.  compute maxwarn=0.  do if ((desctmp(3,jd)=desctmp(4,jd)) and novar=0).  compute errcode(errs,1)=15.  compute errs=errs+1.  compute criterr=1.  compute novar=1.  end if.  compute tmp=((descdat(:,1)=desctmp(3,jd))+(descdat(:,1)=desctmp(4,jd))).  compute desctmp(8,jd)=(csum(tmp)=nrow(tmp)).  compute tmp = descdat.  compute tmp(GRADE(descdat),:) = descdat.  compute descdat = tmp.  release tmp.  compute decval={.16;.5;.84}.  loop kd=1 to 3.  compute low=trunc(decval(kd,1)*(nrow(descdat)+1)).  compute lowdec=decval(kd,1)*(nrow(descdat)+1)-low.  compute value=descdat(low,1)+(descdat((low+1),1)-descdat(low,1))*lowdec.  compute desctmp((4+kd),jd)=value.  end loop.  compute mnotev=1.  compute modvals=desctmp(5:7,:).  do if (quantile <> 1).  compute desctmp(5,jd)=desctmp(1,jd)-desctmp(2,jd).  compute desctmp(6,jd)=desctmp(1,jd).  compute desctmp(7,jd)=desctmp(1,jd)+desctmp(2,jd).  compute modvals=desctmp(5:7,:).  compute mnotev=2.  do if (modvals(1,1) < desctmp(3,1)).  compute modvals(1,1)=desctmp(3,1).  compute minwarn=1.  end if.  do if (modvals(3,1) > desctmp(4,1)).  compute modvals(3,1)=desctmp(4,1).  compute maxwarn=1.  end if.  end if.  do if (desctmp(8,1)=1).  compute modvals={desctmp(3,1);desctmp(4,1)}.  compute mnotev=0.  compute minwarn=0.  compute maxwarn=0.  end if.  end if.  end loop  .  compute wmin=desctmp(3,1).  compute wmax=desctmp(4,1).  do if (wmodcust=0).  compute wmodvals=modvals.  compute wprobval=wmodvals.  end if.  end if.  end if.  do if ((center = 1) or (center = 2 and zdich = 0)).  do if (zprod=1 and mczok=0 and nzpval > 0).  loop i = 1 to nzs.  compute ztmp(:,i)=ztmp(:,i)-(csum(ztmp(:,i))/n).  compute centvar={centvar,znames(1,i)}.  end loop.  .  compute desctmp=make((8-(4* zmodcust )),ncol( ztmp ),-999).  loop jd=1 to ncol( ztmp ).  compute descdat= ztmp (:,jd).  compute desctmp(1,jd) = csum(descdat)/nrow(descdat).  compute desctmp(2,jd) = (nrow(descdat)*sscp(descdat))-(t(csum(descdat))*(csum(descdat))).  compute desctmp(2,jd) = sqrt(desctmp(2,jd)/(nrow(descdat)*(nrow(descdat)-1))).  compute desctmp(3,jd)=cmin(descdat).  compute desctmp(4,jd)=cmax(descdat).  do if ( zmodcust =0).  compute minwarn=0.  compute maxwarn=0.  do if ((desctmp(3,jd)=desctmp(4,jd)) and novar=0).  compute errcode(errs,1)=15.  compute errs=errs+1.  compute criterr=1.  compute novar=1.  end if.  compute tmp=((descdat(:,1)=desctmp(3,jd))+(descdat(:,1)=desctmp(4,jd))).  compute desctmp(8,jd)=(csum(tmp)=nrow(tmp)).  compute tmp = descdat.  compute tmp(GRADE(descdat),:) = descdat.  compute descdat = tmp.  release tmp.  compute decval={.16;.5;.84}.  loop kd=1 to 3.  compute low=trunc(decval(kd,1)*(nrow(descdat)+1)).  compute lowdec=decval(kd,1)*(nrow(descdat)+1)-low.  compute value=descdat(low,1)+(descdat((low+1),1)-descdat(low,1))*lowdec.  compute desctmp((4+kd),jd)=value.  end loop.  compute mnotev=1.  compute modvals=desctmp(5:7,:).  do if (quantile <> 1).  compute desctmp(5,jd)=desctmp(1,jd)-desctmp(2,jd).  compute desctmp(6,jd)=desctmp(1,jd).  compute desctmp(7,jd)=desctmp(1,jd)+desctmp(2,jd).  compute modvals=desctmp(5:7,:).  compute mnotev=2.  do if (modvals(1,1) < desctmp(3,1)).  compute modvals(1,1)=desctmp(3,1).  compute minwarn=1.  end if.  do if (modvals(3,1) > desctmp(4,1)).  compute modvals(3,1)=desctmp(4,1).  compute maxwarn=1.  end if.  end if.  do if (desctmp(8,1)=1).  compute modvals={desctmp(3,1);desctmp(4,1)}.  compute mnotev=0.  compute minwarn=0.  compute maxwarn=0.  end if.  end if.  end loop  .  compute zmin=desctmp(3,1).  compute zmax=desctmp(4,1).  do if (zmodcust=0).  compute zmodvals=modvals.  compute zprobval=zmodvals.  end if.  end if.  end if.  do if ((center = 1) or (center = 2 and xdich = 0)).  do if (xprod=1 and mcxok=0).  loop i = 1 to nxs.  compute xtmp(:,i)=xtmp(:,i)-(csum(xtmp(:,i))/n).  compute centvar={centvar,xnames(1,i)}.  end loop.  .  compute desctmp=make((8-(4* 0 )),ncol( xtmp ),-999).  loop jd=1 to ncol( xtmp ).  compute descdat= xtmp (:,jd).  compute desctmp(1,jd) = csum(descdat)/nrow(descdat).  compute desctmp(2,jd) = (nrow(descdat)*sscp(descdat))-(t(csum(descdat))*(csum(descdat))).  compute desctmp(2,jd) = sqrt(desctmp(2,jd)/(nrow(descdat)*(nrow(descdat)-1))).  compute desctmp(3,jd)=cmin(descdat).  compute desctmp(4,jd)=cmax(descdat).  do if ( 0 =0).  compute minwarn=0.  compute maxwarn=0.  do if ((desctmp(3,jd)=desctmp(4,jd)) and novar=0).  compute errcode(errs,1)=15.  compute errs=errs+1.  compute criterr=1.  compute novar=1.  end if.  compute tmp=((descdat(:,1)=desctmp(3,jd))+(descdat(:,1)=desctmp(4,jd))).  compute desctmp(8,jd)=(csum(tmp)=nrow(tmp)).  compute tmp = descdat.  compute tmp(GRADE(descdat),:) = descdat.  compute descdat = tmp.  release tmp.  compute decval={.16;.5;.84}.  loop kd=1 to 3.  compute low=trunc(decval(kd,1)*(nrow(descdat)+1)).  compute lowdec=decval(kd,1)*(nrow(descdat)+1)-low.  compute value=descdat(low,1)+(descdat((low+1),1)-descdat(low,1))*lowdec.  compute desctmp((4+kd),jd)=value.  end loop.  compute mnotev=1.  compute modvals=desctmp(5:7,:).  do if (quantile <> 1).  compute desctmp(5,jd)=desctmp(1,jd)-desctmp(2,jd).  compute desctmp(6,jd)=desctmp(1,jd).  compute desctmp(7,jd)=desctmp(1,jd)+desctmp(2,jd).  compute modvals=desctmp(5:7,:).  compute mnotev=2.  do if (modvals(1,1) < desctmp(3,1)).  compute modvals(1,1)=desctmp(3,1).  compute minwarn=1.  end if.  do if (modvals(3,1) > desctmp(4,1)).  compute modvals(3,1)=desctmp(4,1).  compute maxwarn=1.  end if.  end if.  do if (desctmp(8,1)=1).  compute modvals={desctmp(3,1);desctmp(4,1)}.  compute mnotev=0.  compute minwarn=0.  compute maxwarn=0.  end if.  end if.  end loop  .  compute xmodvals=modvals.  compute xprobval=xmodvals.  end if.  end if.  do if (nms > 0).  loop i = 1 to nms.  do if (mprod(1,i)=1).  compute mtmp(:,i)=mtmp(:,i)-(csum(mtmp(:,i))/n).  compute centvar={centvar,mnames(1,i)}.  end if.  end loop.  .  compute desctmp=make((8-(4* 0 )),ncol( mtmp ),-999).  loop jd=1 to ncol( mtmp ).  compute descdat= mtmp (:,jd).  compute desctmp(1,jd) = csum(descdat)/nrow(descdat).  compute desctmp(2,jd) = (nrow(descdat)*sscp(descdat))-(t(csum(descdat))*(csum(descdat))).  compute desctmp(2,jd) = sqrt(desctmp(2,jd)/(nrow(descdat)*(nrow(descdat)-1))).  compute desctmp(3,jd)=cmin(descdat).  compute desctmp(4,jd)=cmax(descdat).  do if ( 0 =0).  compute minwarn=0.  compute maxwarn=0.  do if ((desctmp(3,jd)=desctmp(4,jd)) and novar=0).  compute errcode(errs,1)=15.  compute errs=errs+1.  compute criterr=1.  compute novar=1.  end if.  compute tmp=((descdat(:,1)=desctmp(3,jd))+(descdat(:,1)=desctmp(4,jd))).  compute desctmp(8,jd)=(csum(tmp)=nrow(tmp)).  compute tmp = descdat.  compute tmp(GRADE(descdat),:) = descdat.  compute descdat = tmp.  release tmp.  compute decval={.16;.5;.84}.  loop kd=1 to 3.  compute low=trunc(decval(kd,1)*(nrow(descdat)+1)).  compute lowdec=decval(kd,1)*(nrow(descdat)+1)-low.  compute value=descdat(low,1)+(descdat((low+1),1)-descdat(low,1))*lowdec.  compute desctmp((4+kd),jd)=value.  end loop.  compute mnotev=1.  compute modvals=desctmp(5:7,:).  do if (quantile <> 1).  compute desctmp(5,jd)=desctmp(1,jd)-desctmp(2,jd).  compute desctmp(6,jd)=desctmp(1,jd).  compute desctmp(7,jd)=desctmp(1,jd)+desctmp(2,jd).  compute modvals=desctmp(5:7,:).  compute mnotev=2.  do if (modvals(1,1) < desctmp(3,1)).  compute modvals(1,1)=desctmp(3,1).  compute minwarn=1.  end if.  do if (modvals(3,1) > desctmp(4,1)).  compute modvals(3,1)=desctmp(4,1).  compute maxwarn=1.  end if.  end if.  do if (desctmp(8,1)=1).  compute modvals={desctmp(3,1);desctmp(4,1)}.  compute mnotev=0.  compute minwarn=0.  compute maxwarn=0.  end if.  end if.  end loop  .  compute mmodvals=modvals.  compute mprobval=mmodvals.  end if.  end if.  do if (ncol(centvar) > 1).  compute notecode(notes,1)=3.  compute notes=notes+1.  end if.  end if.  do if (criterr=0).  compute wsum=rsum(csum(wcmat)).  compute zsum=rsum(csum(zcmat)).  compute wzsum=rsum(csum(wzcmat)).  compute nump=make(1,(nys+nms),-999).  compue numint=make(1,(nys+nms),0).  compute datcount=1.  compute xtmpuse=0.  compute wtmpuse=0.  compute ztmpuse=0.  compute xwtmpus=0.  compute xztmpus=0.  compute wztmpus=0.  compute xwztmpu=0.  compute xtmploc=-999.  compute wtmploc=-999.  compute xwtmplo=-999.  compute ztmploc=-999.  compute xztmplo=-999.  compute wztmplo=-999.  compute xwztmplo=-999.  compute vlabs={' '}.  do if (ncs > 0).  compute ctmpuse=make(1,ncs,0).  end if.  do if (nms > 0).  compute mtmpuse=make(1,nms,0).  compute mwtmpus=make(1,nms,0).  compute mztmpus=make(1,nms,0).  compute mwztmpu=make(1,nms,0).  compute mtmploc=make(1,nms,0).  compute mwtmplo=make(nwvls,nms,-999).  compute mztmplo=make(nzvls,nms,-999).  compute mwztmplo=make((nwvls*nzvls),nms,-999).  end if.  do if (ncs > 0).  compute ctmploc=make(1,ncs,0).  end if.  compute fulldat=make(n,1,1).  compute datindx=make(1000,(nms+nys),-999).  compute wherew=make(2,(nms+nys),-999).  compute wherex=make(2,(nms+nys),-999).  compute wherez=make(2,(nms+nys),-999).  compute wherexw=make(2,(nms+nys),-999).  compute wherexz=make(2,(nms+nys),-999).  compute wherewz=make(2,(nms+nys),-999).  compute wherexwz=make(2,(nms+nys),-999).  do if (nms > 0).  compute wherem=make(nms,(nms+nys),-999).  compute wheremw = make(nms*2,(nms+nys),-999).  compute wheremz = make(nms*2,(nms+nys),-999).  compute wheremwz = make(nms*2,(nms+nys),-999).  end if.  compute wzhigh=make(1000,(((nms+1)*(nms+2))/2),0).  compute whigh=make(1000,(((nms+1)*(nms+2))/2),0).  compute zhigh=make(1000,(((nms+1)*(nms+2))/2),0).  compute fochigh=make(1000,(((nms+1)*(nms+2))/2),0).  compute xcoefloc={1;2;3;4;5;6;7;8;9}.  compute intkey = {' ', ' ', ' ', ' ', ' ', ' ', ' '}.  compute wzhighct=0.  compute whighct=0.  compute zhighct=0.  compute foccnt=0.  loop i = 2 to nrow(bcmat).  compute wdid=0.  compute zdid=0.  compute wzdid=0.  compute cntmp=1.  compute start=1.  do if (i < nrow(bcmat)).  compute outv=mtmp(:,(i-1)).  compute modlabel={mnames(1,(i-1));'constant'}.  end if.  do if (i = nrow(bcmat)).  compute outv=ytmp.  compute modlabel={ynames;'constant'}.  end if.  loop j = 1 to (i-1).  compute foccnt=foccnt+1.  do if (j = 1 and bcmat(i,j)=1).  compute outv={outv,xtmp}.  compute modlabel={modlabel;xcatlab(1:nxvls,1)}.  do if (xtmpuse=0).  compute fulldat={fulldat,xtmp}.  compute xtmpuse=1.  loop k4=datcount to (datcount+(nxvls-1)).  compute xtmploc={xtmploc;k4}.  end loop.  compute xtmploc=xtmploc(2:nrow(xtmploc),1).  compute datcount=datcount+nxvls.  end if.  compute datindx(start:(start+nrow(xtmploc)-1),(i-1))=xtmploc.  compute wherex(1,(i-1))=start+1.  compute wherex(2,(i-1))=start+nrow(xtmploc)-1+1.  do if (model = 74).  end if.  compute onebl=make(nrow(xtmploc),1,1).  compute fochigh((start+1):(start+nrow(xtmploc)),foccnt)=onebl.  compute start=start+nrow(xtmploc).  end if.  do if (j > 1 and bcmat(i,j)=1).  compute outv={outv,mtmp(:,(j-1))}.  compute modlabel={modlabel;mnames(1,(j-1))}.  do if (mtmpuse(1,(j-1))=0).  compute fulldat={fulldat,mtmp(:,(j-1))}.  compute mtmpuse(1,(j-1))=1.  compute mtmploc(1,(j-1))=datcount.  compute datcount=datcount+1.  end if.  compute datindx(start:(start+nrow(mtmploc)-1),(i-1))=mtmploc(1,(j-1)).  compute wherem((j-1),(i-1))=start+1.  compute onebl=make(nrow(mtmploc(1,j-1)),1,1).  compute ttt=nrow(mtmploc(1,(j-1)))+start-1.  compute fochigh((start+1):(start+nrow(mtmploc(1,(j-1)))),foccnt)=onebl.  compute start=start+nrow(mtmploc(1,(j-1))).  end if.  end loop.  do if (wsum > 0).  loop j = 1 to (i-1).  compute whighct=whighct+1.  do if (j = 1 and wcmat(i,j)=1).  do if (wdid=0).  compute outv={outv,wtmp}.  do if (ncs > 0 and wiscov > 0).  compute ccmatoff((i-1),wiscov)=0.  end if.  compute modlabel={modlabel;wcatlab(1:nwvls,1)}.  compute wdid=1.  do if (wtmpuse=0).  compute fulldat={fulldat,wtmp}.  do if (ncs > 0 and wiscov > 0).  compute ccmatoff((i-1),wiscov)=0.  end if.  compute wtmpuse=1.  loop k4=datcount to (datcount+(nwvls-1)).  compute wtmploc={wtmploc;k4}.  end loop.  compute wtmploc=wtmploc(2:nrow(wtmploc),1).  compute datcount=datcount+nwvls.  end if.  end if.  compute datindx(start:(start+nrow(wtmploc)-1),(i-1))=wtmploc.  compute wherew(1,(i-1))=start+1.  compute wherew(2,(i-1))=start+nrow(wtmploc)-1+1.  compute start=start+nrow(wtmploc).  loop k1=1 to nxvls.  loop k2 = 1 to nwvls.  compute outv={outv,(xtmp(:,k1)&*wtmp(:,k2))}.  do if (ncs > 0 and wiscov > 0).  compute ccmatoff((i-1),wiscov)=0.  end if.  compute modlabel={modlabel;intlab(cntmp,1)}.  compute intkey={intkey;intlab(cntmp,1),':',xcatlab(k1,1),'x',wcatlab(k2,1),' ',' '}.  compute cntmp=cntmp+1.  end loop.  end loop.  do if (xwtmpus=0).  compute fulldat={fulldat,outv(:,(ncol(outv)-(nxvls*nwvls)+1):ncol(outv))}.  compute xwtmpus=1.  do if (ncs > 0 and wiscov > 0).  compute ccmatoff((i-1),wiscov)=0.  end if.  loop k4=datcount to (datcount+((nwvls*nxvls)-1)).  compute xwtmplo={xwtmplo;k4}.  end loop.  compute xwtmplo=xwtmplo(2:nrow(xwtmplo),1).  compute datcount=datcount+(nxvls*nwvls).  end if.  compute datindx(start:(start+nrow(xwtmplo)-1),(i-1))=xwtmplo.  compute wherexw(1,(i-1))=start+1.  compute wherexw(2,(i-1))=start+nrow(xwtmplo)-1+1.  compute onebl=make(nrow(xwtmplo),1,1).  compute whigh((start+1):(start+nrow(xwtmplo)),whighct)=onebl.  compute start=start+nrow(xwtmplo).  end if.  do if (j > 1 and wcmat(i,j)=1).  do if (wdid=0 and model <> 74).  compute outv={outv,wtmp}.  do if (ncs > 0 and wiscov > 0).  compute ccmatoff((i-1),wiscov)=0.  end if.  compute modlabel={modlabel;wcatlab(1:nwvls,1)}.  compute wdid=1.  do if (wtmpuse=0).  compute fulldat={fulldat,wtmp}.  do if (ncs > 0 and wiscov > 0).  compute ccmatoff((i-1),wiscov)=0.  end if.  compute wtmpuse=1.  loop k4=datcount to (datcount+(nwvls-1)).  compute wtmploc={wtmploc;k4}.  end loop.  compute wtmploc=wtmploc(2:nrow(wtmploc),1).  compute datcount=datcount+nwvls.  end if.  compute datindx(start:(start+nrow(wtmploc)-1),(i-1))=wtmploc.  compute wherew(1,(i-1))=start+1.  compute wherew(2,(i-1))=start+nrow(wtmploc)-1+1.  compute start=start+nrow(wtmploc).  end if.  loop k2 = 1 to nwvls.  compute outv={outv,(mtmp(:,(j-1))&*wtmp(:,k2))}.  do if (ncs > 0 and wiscov > 0).  compute ccmatoff((i-1),wiscov)=0.  end if.  compute modlabel={modlabel;intlab(cntmp,1)}.  compute intkey={intkey;intlab(cntmp,1),':', mnames(1,(j-1)),'x',wcatlab(k2,1),' ',' '}.  compute cntmp=cntmp+1.  end loop.  do if (mwtmpus(1,(j-1))=0).  compute fulldat={fulldat,outv(:,(ncol(outv)-nwvls+1):ncol(outv))}.  do if (ncs > 0 and wiscov > 0).  compute ccmatoff((i-1),wiscov)=0.  end if.  compute mwtmpus(1,(j-1))=1.  compute mw22=-999.  loop k4=datcount to (datcount+(nwvls-1)).  compute mw22={mw22;k4}.  end loop.  compute mwtmplo(:,(j-1))=mw22(2:nrow(mw22),1).  compute datcount=datcount+nwvls.  end if.  compute datindx(start:(start+nrow(mwtmplo)-1),(i-1))=mwtmplo(:,(j-1)).  compute wheremw(((2*j)-3),(i-1))=start+1.  compute wheremw(((2*j)-2),(i-1))=start+nrow(mwtmplo)-1+1.  compute onebl=make(nrow(mwtmplo),1,1).  compute whigh((start+1):(start+nrow(mwtmplo)),whighct)=onebl.  compute start=start+nrow(mwtmplo).  end if.  end loop.  end if.  do if (zsum > 0).  loop j = 1 to (i-1).  compute zhighct=zhighct+1.  do if (j = 1 and zcmat(i,j)=1).  do if (zdid=0).  compute outv={outv,ztmp}.  do if (ncs > 0 and ziscov > 0).  compute ccmatoff((i-1),ziscov)=0.  end if.  compute modlabel={modlabel;zcatlab(1:nzvls,1)}.  compute zdid=1.  do if (ztmpuse=0).  compute fulldat={fulldat,ztmp}.  do if (ncs > 0 and ziscov > 0).  compute ccmatoff((i-1),ziscov)=0.  end if.  compute ztmpuse=1.  loop k4=datcount to (datcount+(nzvls-1)).  compute ztmploc={ztmploc;k4}.  end loop.  compute ztmploc=ztmploc(2:nrow(ztmploc),1).  compute datcount=datcount+nzvls.  end if.  end if.  compute datindx(start:(start+nrow(ztmploc)-1),(i-1))=ztmploc.  compute wherez(1,(i-1))=start+1.  compute wherez(2,(i-1))=start+nrow(ztmploc)-1+1.  compute start=start+nrow(ztmploc).  loop k1=1 to nxvls.  loop k2 = 1 to nzvls.  compute outv={outv,(xtmp(:,k1)&*ztmp(:,k2))}.  do if (ncs > 0 and ziscov > 0).  compute ccmatoff((i-1),ziscov)=0.  end if.  compute modlabel={modlabel;intlab(cntmp,1)}.  compute intkey={intkey;intlab(cntmp,1),':',xcatlab(k1,1),'x',zcatlab(k2,1),' ',' '}.  compute cntmp=cntmp+1.  end loop.  end loop.  do if (xztmpus=0).  compute fulldat={fulldat,outv(:,(ncol(outv)-(nxvls*nzvls)+1):ncol(outv))}.  do if (ncs > 0 and ziscov > 0).  compute ccmatoff((i-1),ziscov)=0.  end if.  compute xztmpus=1.  loop k4=datcount to (datcount+((nzvls*nxvls)-1)).  compute xztmplo={xztmplo;k4}.  end loop.  compute xztmplo=xztmplo(2:nrow(xztmplo),1).  compute datcount=datcount+(nxvls*nzvls).  end if.  compute datindx(start:(start+nrow(xztmplo)-1),(i-1))=xztmplo.  compute wherexz(1,(i-1))=start+1.  compute wherexz(2,(i-1))=start+nrow(xztmplo)-1+1.  compute onebl=make(nrow(xztmplo),1,1).  compute zhigh((start+1):(start+nrow(xztmplo)),zhighct)=onebl.  compute start=start+nrow(xztmplo).  end if.  do if (j > 1 and zcmat(i,j)=1).  do if (zdid=0).  compute outv={outv,ztmp}.  do if (ncs > 0 and ziscov > 0).  compute ccmatoff((i-1),ziscov)=0.  end if.  compute modlabel={modlabel;zcatlab(1:nzvls,1)}.  compute zdid=1.  do if (ztmpuse=0).  compute fulldat={fulldat,ztmp}.  do if (ncs > 0 and ziscov > 0).  compute ccmatoff((i-1),ziscov)=0.  end if.  compute ztmpuse=1.  loop k4=datcount to (datcount+(nzvls-1)).  compute ztmploc={ztmploc;k4}.  end loop.  compute ztmploc=ztmploc(2:nrow(ztmploc),1).  compute datcount=datcount+nzvls.  end if.  compute datindx(start:(start+nrow(ztmploc)-1),(i-1))=ztmploc.  compute wherez(1,(i-1))=start+1.  compute wherez(2,(i-1))=start+nrow(ztmploc)-1+1.  compute start=start+nrow(ztmploc).  end if.  loop k2 = 1 to nzvls.  compute outv={outv,(mtmp(:,(j-1))&*ztmp(:,k2))}.  do if (ncs > 0 and ziscov > 0).  compute ccmatoff((i-1),ziscov)=0.  end if.  compute modlabel={modlabel;intlab(cntmp,1)}.  compute intkey={intkey;intlab(cntmp,1),':', mnames(1,(j-1)),'x',zcatlab(k2,1),' ',' '}.  compute cntmp=cntmp+1.  end loop.  do if (mztmpus(1,(j-1))=0).  compute fulldat={fulldat,outv(:,(ncol(outv)-nzvls+1):ncol(outv))}.  do if (ncs > 0 and ziscov > 0).  compute ccmatoff((i-1),ziscov)=0.  end if.  compute mztmpus(1,(j-1))=1.  compute mz22=-999.  loop k4=datcount to (datcount+(nzvls-1)).  compute mz22={mz22;k4}.  end loop.  compute mztmplo(:,(j-1))=mz22(2:nrow(mz22),1).  compute datcount=datcount+nzvls.  end if.  compute datindx(start:(start+nrow(mztmplo)-1),(i-1))=mztmplo(:,(j-1)).  compute wheremz(((2*j)-3),(i-1))=start+1.  compute wheremz(((2*j)-2),(i-1))=start+nrow(mztmplo)-1+1.  compute onebl=make(nrow(mztmplo),1,1).  compute zhigh((start+1):(start+nrow(mztmplo)),zhighct)=onebl.  compute start=start+nrow(mztmplo).  end if.  end loop.  end if.  do if (wzsum > 0).  loop j = 1 to (i-1).  compute wzhighct=wzhighct+1.  do if (j = 1 and wzcmat(i,j)=1).  do if (wzdid=0).  loop k1=1 to nwvls.  loop k2 = 1 to nzvls.  compute outv={outv,(wtmp(:,k1)&*ztmp(:,k2))}.  do if (ncs > 0 and (ziscov > 0)).  compute ccmatoff((i-1),ziscov)=0.  end if.  do if (ncs > 0 and (wiscov > 0)).  compute ccmatoff((i-1),wiscov)=0.  end if.  compute modlabel={modlabel;intlab(cntmp,1)}.  compute intkey={intkey;intlab(cntmp,1),':',wcatlab(k1,1),'x',zcatlab(k2,1),' ',' '}.  compute cntmp=cntmp+1.  end loop.  end loop.  do if (wztmpus=0).  compute fulldat={fulldat,outv(:,(ncol(outv)-(nwvls*nzvls)+1):ncol(outv))}.  do if (ncs > 0 and (ziscov > 0)).  compute ccmatoff((i-1),ziscov)=0.  end if.  do if (ncs > 0 and (wiscov > 0)).  compute ccmatoff((i-1),wiscov)=0.  end if.  compute wztmpus=1.  loop k4=datcount to (datcount+((nwvls*nzvls)-1)).  compute wztmplo={wztmplo;k4}.  end loop.  compute wztmplo=wztmplo(2:nrow(wztmplo),1).  compute datcount=datcount+(nzvls*nwvls).  end if.  compute wzdid=1.  end if.  compute datindx(start:(start+nrow(wztmplo)-1),(i-1))=wztmplo.  compute wherewz(1,(i-1))=start+1.  compute wherewz(2,(i-1))=start+nrow(wztmplo)-1+1.  compute start=start+nrow(wztmplo).  loop k1=1 to nxvls.  loop k2=1 to nwvls.  loop k3=1 to nzvls.  compute outv={outv,(xtmp(:,k1)&*wtmp(:,k2)&*ztmp(:,k3))}.  do if (ncs > 0 and (ziscov > 0)).  compute ccmatoff((i-1),ziscov)=0.  end if.  do if (ncs > 0 and (wiscov > 0)).  compute ccmatoff((i-1),wiscov)=0.  end if.  compute modlabel={modlabel;intlab(cntmp,1)}.  compute intkey={intkey;intlab(cntmp,1),':',xcatlab(k1,1),'x',wcatlab(k2,1),'x', zcatlab(k3,1)}.  compute cntmp=cntmp+1.  end loop.  end loop.  end loop.  do if (xwztmpu=0).  compute fulldat={fulldat,outv(:,(ncol(outv)-(nxvls*nwvls*nzvls)+1):ncol(outv))}.  do if (ncs > 0 and (ziscov > 0)).  compute ccmatoff((i-1),ziscov)=0.  end if.  do if (ncs > 0 and (wiscov > 0)).  compute ccmatoff((i-1),wiscov)=0.  end if.  compute xwztmpu=1.  loop k4=datcount to (datcount+((nzvls*nxvls*nwvls)-1)).  compute xwztmplo={xwztmplo;k4}.  end loop.  compute xwztmplo=xwztmplo(2:nrow(xwztmplo),1).  compute datcount=datcount+(nxvls*nzvls*nwvls).  end if.  compute datindx(start:(start+nrow(xwztmplo)-1),(i-1))=xwztmplo.  compute wherexwz(1,(i-1))=start+1.  compute wherexwz(2,(i-1))=start+nrow(xwztmplo)-1+1.  compute onebl=make(nrow(xwztmplo),1,1).  compute wzhigh((start+1):(start+nrow(xwztmplo)),wzhighct)=onebl.  compute start=start+nrow(xwztmplo).  end if.  do if (j > 1 and wzcmat(i,j)=1).  do if (wzdid=0).  loop k1=1 to nwvls.  loop k2 = 1 to nzvls.  compute outv={outv,(wtmp(:,k1)&*ztmp(:,k2))}.  do if (ncs > 0 and (ziscov > 0)).  compute ccmatoff((i-1),ziscov)=0.  end if.  do if (ncs > 0 and (wiscov > 0)).  compute ccmatoff((i-1),wiscov)=0.  end if.  compute modlabel={modlabel;intlab(cntmp,1)}.  compute intkey={intkey;intlab(cntmp,1),':',wcatlab(k1,1),'x',zcatlab(k2,1),' ',' '}.  compute cntmp=cntmp+1.  end loop.  end loop.  do if (wztmpus=0).  compute fulldat={fulldat,outv(:,(ncol(outv)-(nwvls*nzvls)+1):ncol(outv))}.  do if (ncs > 0 and (ziscov > 0)).  compute ccmatoff((i-1),ziscov)=0.  end if.  do if (ncs > 0 and (wiscov > 0)).  compute ccmatoff((i-1),wiscov)=0.  end if.  compute wztmpus=1.  loop k4=datcount to (datcount+((nwvls*nzvls)-1)).  compute wztmplo={wztmplo;k4}.  end loop.  compute wztmplo=wztmplo(2:nrow(wztmplo),1).  compute datcount=datcount+(nzvls*nwvls).  end if.  compute wzdid=1.  compute datindx(start:(start+nrow(wztmplo)-1),(i-1))=wztmplo.  compute wherewz(1,(i-1))=start+1.  compute wherewz(2,(i-1))=start+nrow(wztmplo)-1+1.  compute start=start+nrow(wztmplo).  end if.  loop k1 = 1 to nwvls.  loop k2 = 1 to nzvls.  compute outv={outv,(mtmp(:,(j-1))&*wtmp(:,k1)&*ztmp(:,k2))}.  do if (ncs > 0 and (ziscov > 0)).  compute ccmatoff((i-1),ziscov)=0.  end if.  do if (ncs > 0 and (wiscov > 0)).  compute ccmatoff((i-1),wiscov)=0.  end if.  compute modlabel={modlabel;intlab(cntmp,1)}.  compute intkey={intkey;intlab(cntmp,1),':',mnames(1,(j-1)),'x',wcatlab(k1,1),'x', zcatlab(k2,1)}.  compute cntmp=cntmp+1.  end loop.  end loop.  do if (mwztmpu(1,(j-1))=0).  compute fulldat={fulldat,outv(:,(ncol(outv)-(nwvls*nzvls)+1):ncol(outv))}.  do if (ncs > 0 and (ziscov > 0)).  compute ccmatoff((i-1),ziscov)=0.  end if.  do if (ncs > 0 and (wiscov > 0)).  compute ccmatoff((i-1),wiscov)=0.  end if.  compute mwztmpu(1,(j-1))=1.  compute mz22=-999.  loop k4=datcount to (datcount+(nwvls*nzvls)-1).  compute mz22={mz22;k4}.  end loop.  compute mwztmplo(:,(j-1))=mz22(2:nrow(mz22),1).  compute datcount=datcount+(nwvls*nzvls).  end if.  compute datindx(start:(start+nrow(mwztmplo)-1),(i-1))=mwztmplo(:,(j-1)).  compute wheremwz(((2*j)-3),(i-1))=start+1.  compute wheremwz(((2*j)-2),(i-1))=start+nrow(mwztmplo)-1+1.  compute onebl=make(nrow(mwztmplo),1,1).  compute wzhigh((start+1):(start+nrow(mwztmplo)),wzhighct)=onebl.  compute start=start+nrow(mwztmplo).  end if.  end loop.  end if.  do if (ncs > 0).  compute ccmat=ccmat&*ccmatoff.  loop j = 1 to ncs.  do if (ccmat((i-1),j))=1.  do if (j=wiscov).  compute ctmp(:,j)=wtmp.  end if.  do if (j=ziscov).  compute ctmp(:,j)=ztmp.  end if.  compute outv={outv,ctmp(:,j)}.  compute modlabel={modlabel;covnames(1,j)}.  do if (ctmpuse(1,j)=0).  compute fulldat={fulldat,ctmp(:,j)}.  compute ctmpuse(1,j)=1.  compute ctmploc(1,j)=datcount.  compute datcount=datcount+1.  end if.  compute datindx(start:(start+nrow(ctmploc)-1),(i-1))=ctmploc(1,j).  compute start=start+nrow(ctmploc(1,j)).  end if.  end loop.  end if.  compute wdid=0.  compute zdid=0.  compute wzdid=0.  compute vlabs={vlabs;modlabel(2:nrow(modlabel),1)}.  compute numint(1,(i-1))=cntmp-1.  compute nump(1,(i-1))=nrow(modlabel)-1.  end loop.  release datcount, xtmpuse, wtmpuse, ztmpuse, xwtmpus, xztmpus, wztmpus, xwztmpu.  release xtmploc, wtmploc, xwtmplo, ztmploc, xztmplo, wztmplo, xwztmplo, foccnt.  do if (modcok=1 and ((nms > 0) or (zcmat(2,1) <> 1) or (mcx <> 0))).  compute notecode(notes,1) = 19.  compute notes = notes + 1.  compute modcok=0.  end if.  do if ((serial = 1 or (rsum(numint)>0) or nms=0) and mc > 0).  compute notecode(notes,1) = 15.  compute notes = notes + 1.  compute boot=mc.  compute mc=0.  end if.  do if (boot <> 0 or mc <> 0).  compute bootsz=boot.  do if (mc > 0).  compute bootsz=mc.  compute saveboot=0.  end if.  loop.  compute cilow = rnd(bootsz*(1-(conf/100))/2).  compute cihigh = trunc((bootsz*(conf/100)+(bootsz*(1-(conf/100))/2)))+1.  do if (cilow < 1 or cihigh > bootsz).  compute bootsz=trunc((bootsz+1000)/1000)*1000.  compute adjust = 1.  end if.  end loop if (cilow gt 0 and cihigh le bootsz).  do if (boot > 0).  compute boot=bootsz.  end if.  do if (mc > 0).  compute mc=bootsz.  end if.  do if (adjust = 1 and boot > 0).  compute notecode(notes,1) = 8.  compute notes = notes + 1.  end if.  do if (adjust = 1 and mc > 0).  compute notecode(notes,1) = 16.  compute notes = notes + 1.  end if.  end if.  compute maxboot = trunc(2*boot).  do if ( 0 > maxboot).  compute maxboot=trunc( 0 ).  end if.  do if (nms > 0).  release mtmpuse, mwtmpus, mwztmpu, mtmploc, mwtmplo, mztmplo, mwztmplo.  end if.  release wdid, zdid, wzdid, start,modlabel.  compute vlabs=vlabs(2:nrow(vlabs),1).  do if (rsum(numint) > 0).  compute intkey=intkey(2:nrow(intkey),:).  end if.  compute fulldat=fulldat(:,2:ncol(fulldat)).  compute fochigh=fochigh(1:rmax(nump),:).  compute whigh=whigh(1:rmax(nump),:).  compute zhigh=zhigh(1:rmax(nump),:).  compute wzhigh=wzhigh(1:rmax(nump),:).  compute coeffs=fochigh+whigh+zhigh+wzhigh.  compute bootloc=make(rmax(nump),ncol(nump),0).  do if (nms > 0).  compute cntmp=1.  loop i = 1 to ncol(nump).  loop j = 1 to nump(1,i).  compute bootloc(j,i)=cntmp.  compute cntmp=cntmp+1.  end loop.  end loop.  compute fochighb=make(nrow(fochigh),ncol(fochigh),0).  compute whighb=fochighb.  compute zhighb=fochighb.  compute wzhighb=fochighb.  compute thetaxmb=make(nrow(fochighb),nms,0).  compute thetaxyb=make(nrow(fochighb),1,0).  compute pathsfoc=make(nxvls,1,0).  compute cntmp=1.  loop i = 1 to (nms+nys).  loop j = 1 to i.  compute fochighb(:,cntmp)=fochigh(:,cntmp)&*bootloc(:,i).  compute whighb(:,cntmp)=whigh(:,cntmp)&*bootloc(:,i).  compute zhighb(:,cntmp)=zhigh(:,cntmp)&*bootloc(:,i).  compute wzhighb(:,cntmp)=wzhigh(:,cntmp)&*bootloc(:,i).  compute coeffsb=fochighb+whighb+zhighb+wzhighb.  do if ((i < (nms+nys)) and (j = 1)).  compute thetaxmb(:,i)=coeffsb(:,cntmp).  end if.  do if ((i = (nms+nys)) and (j = 1)).  compute thetaxyb(:,1)=coeffsb(:,cntmp).  end if.  compute cntmp=cntmp+1.  end loop.  end loop.  compute thetamyb=coeffsb(:,(ncol(coeffsb)-nms+1):ncol(coeffsb)).  do if (serial = 1).  compute thetammb=make(nrow(coeffsb),((nms*(nms-1))/2),0).  end if.  compute cntmp=1.  do if (nms > 1 and serial = 1).  loop i = 1 to (nms-1).  compute start=((i+2)*(i+1))/2.  loop j = 2 to (nms-i+1).  compute thetammb(:,cntmp)=coeffsb(:,start).  compute start=start+j+i-1.  compute cntmp=cntmp+1.  end loop.  end loop.  end if.  end if.  do if ((total = 1) and rsum(numint)=0).  compute dototal=1.  do if ((csum(bcmat(:,1)) <> (nms+nys)) or (rsum(bcmat(nrow(bcmat),:)) <> (nms+nys))).  compute dototal=0.  compute notecode(notes,1) = 12.  compute notes = notes + 1.  end if.  do if (ncs > 0).  do if ((csum(rsum(ccmat))) < (nrow(ccmat)*ncol(ccmat))).  compute dototal=0.  compute notecode(notes,1) = 11.  compute notes = notes + 1.  end if.  end if.  end if.  end if.  do if (criterr=0 and ncs > 0).  do if (rsum((csum(ccmat)=0)) <> 0).  compute errcode(errs,1)=51.  compute errs=errs+1.  compute criterr=1.  end if.  end if.  compute debug= 0.  print/title = '**************** PROCESS Procedure for SPSS Version 3.4.1 ****************'.  print/title = ' Written by Andrew F.Hayes, Ph.D.www.afhayes.com'.  print/title = ' Documentation available in Hayes (2018).www.guilford.com/p/hayes3'/space=0.  do if (criterr=0).  do if (stand=1 and ydich=1).  compute stand=0.  end if.  compute anymod2=csum(rsum(wcmat+zcmat+wzcmat)).  do if (anymod2 > 0 and stand = 1).  compute notecode(notes,1) = 27.  compute notes = notes + 1.  compute stand=0.  end if.  compute funny=1.  print modelvar/title = '**************************************************************************'/format = A8/rnames=modelvlb.  do if (ncs > 0).  print covnames/title='Covariates:'/format=A8.  end if.  print n/title='Sample'/rlabel='Size:'.  do if (( 'random' <> 'random')).  compute seedt= 'random'.  print seedt/title='Custom'/format=A12/rlabel = 'Seed:'.  end if.  do if (mcxok=1).  compute labtmp={xnames,t(xcatlab(1:nxvls,1))}.  print dummatx/title = 'Coding of categorical X variable for analysis:'/cnames = labtmp/format = F6.3.  end if.  do if (mcwok=1).  compute labtmp={wnames,t(wcatlab(1:nwvls,1))}.  print dummatw/title = 'Coding of categorical W variable for analysis:'/cnames = labtmp/format = F6.3.  end if.  do if (mczok=1).  compute labtmp={znames,t(zcatlab(1:nzvls,1))}.  print dummatz/title = 'Coding of categorical Z variable for analysis:'/cnames = labtmp/format = F6.3.  end if.  end if.  do if (criterr = 0).  compute outnames=ynames.  compute outvars=ytmp.  do if (nms > 0).  compute outnames={mnames,ynames}.  compute outvars={mtmp,ytmp}.  compute indcov=make(((nms*2)+(nms*(nxvls-1))),((nms*2)+(nms*(nxvls-1))),0).  compute mcsopath=make(((nms*2)+(nms*(nxvls-1))),1,0).  end if.  compute labstart=1.  compute intstart=1.  compute start=1.  compute coeffmat=make(1,6,0).  compute conseq={' '}.  compute dfmat=0.  compute coeffcol=0.  compute pathscnt=1.  compute pathscn2=1.  loop i = 1 to (nms+nys).  print/title = '**************************************************************************'.  compute highf=make(1,5,0).  compute highf2=highf.  do if ((i = (nms+nys)) and (ydich=1)).  compute highf=make(1,3,0).  compute highf2=highf.  end if.  compute flabel={' '}.  compute y=outvars(:,i).  compute xindx=datindx(1:(nump(1,i)-1),i).  compute x = fulldat(:,xindx).  compute x={ones,x}.  compute xsq=t(x)*x.  compute exsq=eval(xsq).  release xsq.  compute zeroeig=csum(exsq <= 0.000000000002).  print outnames(1,i)/title = 'OUTCOME VARIABLE:'/format = A8/space=0.  do if (ydich=1 and (i = (nms+nys))).  compute nmsd = {outnames(1,i), 'Analysis'}.  print rcd/title = 'Coding of binary Y for logistic regression analysis:'/cnames = nmsd/format = F9.2.  end if.  do if (zeroeig > 0).  print / title = 'SINGULAR OR NEAR SINGULAR DATA MATRIX.'.  compute criterr=1.  compute errcode(errs,1)=31.  compute errs=errs+1.  end if.  compute means=csum(x)/n.  compute vlabsm=vlabs(labstart:(labstart+(nump(1,i)-1)),1).  do if (criterr=0).  do if (ydich=0 or (i < (nms+nys))).  .  do if ( 1 =1).  compute b = inv(t( x )* x )*t( x )* y.  compute modres=b.  do if ( 1 =1).  compute n1=nrow( x ).  compute dfres=n1-(ncol( x )).  compute sstotal = t( y -(csum( y )/n1))*( y -(csum( y )/n1)).  compute resid= y - x *b.  compute ssresid = csum((resid)&**2).  compute r2 = (sstotal-ssresid)/sstotal.  compute adjr2 = 1-((1-r2)*(n1-1)/(dfres)).  compute mse=ssresid/(n1-ncol( x )).  .  compute n1=nrow( x ).  compute invXtX = inv(t( x )* x ).  compute varb = mse *invXtX.  compute k3 = ncol( x ).  compute xhc=0.  do if ( hc <> 5).  compute xhc= x.  compute hat = xhc(:,1).  loop i3=1 to nrow(xhc).  compute hat(i3,1)= xhc(i3,:)*invXtX*t(xhc(i3,:)).  end loop.  do if ( hc = 0 or hc =1).  loop i3 = 1 to k3.  compute xhc(:,i3)=xhc(:,i3)&* resid.  end loop.  end if.  do if ( hc =3 or hc =2).  loop i3=1 to k3.  compute xhc(:,i3) = ( resid &/(1-hat)&**(1/(4hc )))&*xhc(:,i3).  end loop.  end if.  do if ( hc = 4).  compute hcmn=make(n,2,4).  compute hcmn(:,2)=(n1*hat)/k3.  loop i3= 1 to k3.  compute xhc(:,i3) = ( resid &/(1-hat)&**(rmin(hcmn)/2))&*xhc(:,i3).  end loop.  end if.  compute varb=(invXtX*t(xhc)*xhc*invXtX).  do if ( hc =1).  compute varb=(n1/(n1-ncol( x )))&*varb.  end if.  end if.  compute hclab={'se(HC0)','se(HC1)','se(HC2)','se(HC3)','se(HC4)','se'}.  compute hclab=hclab(1,( hc +1)).  compute hcflab={'F(HC0)','F(HC1)','F(HC2)','F(HC3)','F(HC4)','F'}.  compute hcflab=hcflab(1,( hc +1)).  release xhc  .  compute seb=sqrt(diag(varb)).  compute trat = b&/seb.  compute p = 2*(1-tcdf(abs(trat), (dfres))).  compute tval = sqrt(dfres* (exp((dfres-(5/6))*((xp2/(dfres-(2/3)+(.11/dfres)))* (xp2/(dfres-(2/3)+(.11/dfres)))))-1)).  compute modres={modres,seb,trat,p}.  compute modres={modres,(b-tval&*seb),(b+tval&*seb)}.  compute modresl={'coeff',hclab,'t','p','LLCI','ULCI'}.  compute lmat = ident(ncol( x )).  compute lmat = lmat(:,2:ncol(lmat)).  compute fratio = (t(t(lmat)*b)*inv(t(lmat)*varb*lmat)*((t(lmat)*b)))/(ncol( x )-1).  compute pfr = 1-fcdf(fratio,(ncol( x )-1),dfres).  compute modsum={sqrt(r2),r2,mse,fratio,(ncol( x )-1),dfres,pfr}.  compute modsuml={'R','R-sq','MSE',hcflab,'df1','df2', 'p'}.  end if.  end if.  do if ( 1 = 2 or 1 =3).  compute xlp= x.  compute ylp= y.  compute pt2 = make(nrow(ylp),1,(csum(ylp)/nrow(ylp))).  do if ( 1 =2).  compute LL3 = ylp&*ln(pt2)+(1-ylp)&*ln(1-pt2).  end if.  compute LL3 = -2*csum(LL3).  compute bt1 = make(ncol(xlp),1,0).  compute LL1 = 0.  compute pt1 = make(nrow(ylp),1,0.5).  compute pt1lp=pt1.  loop jjj = 1 to iterate.  compute vt1 = mdiag(pt1lp&*(1-pt1lp)).  compute b = bt1+inv(t(xlp)*vt1*xlp)*t(xlp)*(ylp-pt1lp).  do if ( 1 =2).  compute xlpb=xlp*b.  compute xlpbt=(xlpb > -709.7).  compute xlpb709=(1-xlpbt)*(-709.7).  compute xlpb=(xlpb&*xlpbt)+xlpb709.  compute pt1lp = 1/(1+exp(-(xlpb))).  end if.  compute itprob = csum((pt1lp < .00000001) or (pt1lp > .9999999)).  do if (itprob > 0).  loop kkk = 1 to nrow(pt1lp).  do if (pt1lp(kkk,1) > .9999999).  compute pt1lp(kkk,1) = .9999999.  end if.  do if (pt1lp(kkk,1) < .00000001).  compute pt1lp(kkk,1) = .00000001.  end if.  end loop.  compute itprob = 0.  end if.  do if (itprob = 0).  do if ( 1 =2).  compute LL = ylp&*ln(pt1lp)+(1-ylp)&*ln(1-pt1lp).  end if.  compute LL2 = -2*csum(ll).  end if.  do if (abs(LL1-LL2) < converge).  do if ( 1 =1).  compute vt1 = mdiag(pt1lp&*(1-pt1lp)).  compute varb = inv(t(xlp)*vt1*xlp).  compute seb = sqrt(diag(varb)).  end if.  break.  end if.  compute bt1 = b.  compute LL1 = LL2.  end loop.  compute modres=b.  do if (jjj > iterate).  compute itprob = 2.  do if (booting=0).  compute iterrmod=1.  end if.  do if (booting=1).  compute bootiter=1.  end if.  do if (itprobtg=0).  compute itprobtg=1.  compute errcode(errs,1) = 47.  compute errs = errs + 1.  do if (booting = 0 and 1 =1).  compute vt1 = mdiag(pt1lp&*(1-pt1lp)).  compute varb = inv(t(xlp)*vt1*xlp).  compute seb = sqrt(diag(varb)).  end if.  end if.  end if.  do if ( 1 =1).  compute trat = b&/seb.  compute dfres=nrow(xlp).  compute p = 2*(1-cdfnorm(abs(trat))).  compute modres={modres,seb,trat,p}.  compute modres={modres,(b-xp2&*seb),(b+xp2&*seb)}.  compute pvchi=1-chicdf((LL3-LL2),(nrow(modres)-1)).  compute mcF = (LL3-LL2)/LL3.  compute cox = 1-exp(-(LL3-LL2)/nrow(xlp)).  compute nagel = cox/(1-exp(-(LL3)/nrow(xlp))).  compute modsum={LL2,(LL3-LL2),(nrow(modres)-1),pvchi, mcF,cox,nagel}.  compute modsuml={'-2LL','ModelLL', 'df', 'p', 'McFadden', 'CoxSnell', 'Nagelkrk'}.  compute modresl={'coeff','se','Z','p','LLCI','ULCI'}.  end if.  end if  .  compute dfmatt=make(nrow(modres),1,modsum(1,6)).  end if.  do if (ydich=1 and (i = (nms+nys))).  .  do if ( 2 =1).  compute b = inv(t( x )* x )*t( x )* y.  compute modres=b.  do if ( 1 =1).  compute n1=nrow( x ).  compute dfres=n1-(ncol( x )).  compute sstotal = t( y -(csum( y )/n1))*( y -(csum( y )/n1)).  compute resid= y - x *b.  compute ssresid = csum((resid)&**2).  compute r2 = (sstotal-ssresid)/sstotal.  compute adjr2 = 1-((1-r2)*(n1-1)/(dfres)).  compute mse=ssresid/(n1-ncol( x )).  .  compute n1=nrow( x ).  compute invXtX = inv(t( x )* x ).  compute varb = mse *invXtX.  compute k3 = ncol( x ).  compute xhc=0.  do if ( hc <> 5).  compute xhc= x.  compute hat = xhc(:,1).  loop i3=1 to nrow(xhc).  compute hat(i3,1)= xhc(i3,:)*invXtX*t(xhc(i3,:)).  end loop.  do if ( hc = 0 or hc =1).  loop i3 = 1 to k3.  compute xhc(:,i3)=xhc(:,i3)&* resid.  end loop.  end if.  do if ( hc =3 or hc =2).  loop i3=1 to k3.  compute xhc(:,i3) = ( resid &/(1-hat)&**(1/(4hc )))&*xhc(:,i3).  end loop.  end if.  do if ( hc = 4).  compute hcmn=make(n,2,4).  compute hcmn(:,2)=(n1*hat)/k3.  loop i3= 1 to k3.  compute xhc(:,i3) = ( resid &/(1-hat)&**(rmin(hcmn)/2))&*xhc(:,i3).  end loop.  end if.  compute varb=(invXtX*t(xhc)*xhc*invXtX).  do if ( hc =1).  compute varb=(n1/(n1-ncol( x )))&*varb.  end if.  end if.  compute hclab={'se(HC0)','se(HC1)','se(HC2)','se(HC3)','se(HC4)','se'}.  compute hclab=hclab(1,( hc +1)).  compute hcflab={'F(HC0)','F(HC1)','F(HC2)','F(HC3)','F(HC4)','F'}.  compute hcflab=hcflab(1,( hc +1)).  release xhc  .  compute seb=sqrt(diag(varb)).  compute trat = b&/seb.  compute p = 2*(1-tcdf(abs(trat), (dfres))).  compute tval = sqrt(dfres* (exp((dfres-(5/6))*((xp2/(dfres-(2/3)+(.11/dfres)))* (xp2/(dfres-(2/3)+(.11/dfres)))))-1)).  compute modres={modres,seb,trat,p}.  compute modres={modres,(b-tval&*seb),(b+tval&*seb)}.  compute modresl={'coeff',hclab,'t','p','LLCI','ULCI'}.  compute lmat = ident(ncol( x )).  compute lmat = lmat(:,2:ncol(lmat)).  compute fratio = (t(t(lmat)*b)*inv(t(lmat)*varb*lmat)*((t(lmat)*b)))/(ncol( x )-1).  compute pfr = 1-fcdf(fratio,(ncol( x )-1),dfres).  compute modsum={sqrt(r2),r2,mse,fratio,(ncol( x )-1),dfres,pfr}.  compute modsuml={'R','R-sq','MSE',hcflab,'df1','df2', 'p'}.  end if.  end if.  do if ( 2 = 2 or 2 =3).  compute xlp= x.  compute ylp= y.  compute pt2 = make(nrow(ylp),1,(csum(ylp)/nrow(ylp))).  do if ( 2 =2).  compute LL3 = ylp&*ln(pt2)+(1-ylp)&*ln(1-pt2).  end if.  compute LL3 = -2*csum(LL3).  compute bt1 = make(ncol(xlp),1,0).  compute LL1 = 0.  compute pt1 = make(nrow(ylp),1,0.5).  compute pt1lp=pt1.  loop jjj = 1 to iterate.  compute vt1 = mdiag(pt1lp&*(1-pt1lp)).  compute b = bt1+inv(t(xlp)*vt1*xlp)*t(xlp)*(ylp-pt1lp).  do if ( 2 =2).  compute xlpb=xlp*b.  compute xlpbt=(xlpb > -709.7).  compute xlpb709=(1-xlpbt)*(-709.7).  compute xlpb=(xlpb&*xlpbt)+xlpb709.  compute pt1lp = 1/(1+exp(-(xlpb))).  end if.  compute itprob = csum((pt1lp < .00000001) or (pt1lp > .9999999)).  do if (itprob > 0).  loop kkk = 1 to nrow(pt1lp).  do if (pt1lp(kkk,1) > .9999999).  compute pt1lp(kkk,1) = .9999999.  end if.  do if (pt1lp(kkk,1) < .00000001).  compute pt1lp(kkk,1) = .00000001.  end if.  end loop.  compute itprob = 0.  end if.  do if (itprob = 0).  do if ( 2 =2).  compute LL = ylp&*ln(pt1lp)+(1-ylp)&*ln(1-pt1lp).  end if.  compute LL2 = -2*csum(ll).  end if.  do if (abs(LL1-LL2) < converge).  do if ( 1 =1).  compute vt1 = mdiag(pt1lp&*(1-pt1lp)).  compute varb = inv(t(xlp)*vt1*xlp).  compute seb = sqrt(diag(varb)).  end if.  break.  end if.  compute bt1 = b.  compute LL1 = LL2.  end loop.  compute modres=b.  do if (jjj > iterate).  compute itprob = 2.  do if (booting=0).  compute iterrmod=1.  end if.  do if (booting=1).  compute bootiter=1.  end if.  do if (itprobtg=0).  compute itprobtg=1.  compute errcode(errs,1) = 47.  compute errs = errs + 1.  do if (booting = 0 and 1 =1).  compute vt1 = mdiag(pt1lp&*(1-pt1lp)).  compute varb = inv(t(xlp)*vt1*xlp).  compute seb = sqrt(diag(varb)).  end if.  end if.  end if.  do if ( 1 =1).  compute trat = b&/seb.  compute dfres=nrow(xlp).  compute p = 2*(1-cdfnorm(abs(trat))).  compute modres={modres,seb,trat,p}.  compute modres={modres,(b-xp2&*seb),(b+xp2&*seb)}.  compute pvchi=1-chicdf((LL3-LL2),(nrow(modres)-1)).  compute mcF = (LL3-LL2)/LL3.  compute cox = 1-exp(-(LL3-LL2)/nrow(xlp)).  compute nagel = cox/(1-exp(-(LL3)/nrow(xlp))).  compute modsum={LL2,(LL3-LL2),(nrow(modres)-1),pvchi, mcF,cox,nagel}.  compute modsuml={'-2LL','ModelLL', 'df', 'p', 'McFadden', 'CoxSnell', 'Nagelkrk'}.  compute modresl={'coeff','se','Z','p','LLCI','ULCI'}.  end if.  end if  .  compute dfmatt=make(nrow(modres),1,-999).  end if.  compute obscoeff={obscoeff,t(b)}.  print modsum/title = 'Model Summary'/cnames = modsuml/format= F10.4.  print modres/title='Model'/rnames=vlabsm/cnames=modresl/format= F10.4.  compute basemod=modsum(1,1).  compute basemodx=basemod.  do if (ydich=1 and (i = (nms+nys))).  print/title='These results are expressed in a log-odds metric.'.  compute notecode(notes,1) = 26.  compute notes = notes + 1.  end if.  compute coeffmat={coeffmat;modres}.  compute conseqt=make(nrow(modres),1,outnames(1,i)).  compute conseq={conseq;conseqt}.  compute dfmat={dfmat;dfmatt}.  compute labstart=labstart+nump(1,i).  do if (stand=1).  compute predsd=make(nrow(modres),1,0).  compute stdmod=modres(:,1)&/ovsd(1,i).  loop jd=1 to ncol(x).  compute descdat=x(:,jd).  compute predsd(jd,1) = (nrow(descdat)*sscp(descdat))-(t(csum(descdat))*(csum(descdat))).  compute predsd(jd,1) = sqrt(predsd(jd,1)/(nrow(descdat)*(nrow(descdat)-1))).  end loop.  do if (wherex(1,i) <> -999 and ((nxvls > 1) or (xdich=1))).  compute sdmsone=make(nxvls,1,1).  compute predsd(wherex(1,i):wherex(2,i),1)=sdmsone.  compute pstog=1.  end if.  compute predsd(1,1)=1.  compute stdmod=stdmod&*predsd.  compute stdmod=stdmod(2:nrow(stdmod),1).  compute sdvlabs=vlabsm(2:nrow(vlabsm),1).  print stdmod/title='Standardized coefficients'/clabels='coeff'/rnames=sdvlabs/format= F10.4.  end if.  do if (nms > 0 and serial = 0 and (rsum(numint) = 0) and (normal=1 or mc > 0)).  do if (i < (nms+nys)).  compute indcov((((i-1)*nxvls)+1):(i*nxvls),(((i-1)*nxvls)+1):(i*nxvls))=varb(2:(1+nxvls), 2:(1+nxvls)).  compute mcsopath((((i-1)*nxvls)+1):(i*nxvls) ,1)=modres(2:(1+nxvls),1).  end if.  do if (i = (nms+nys)).  compute atm=ncol(wherem).  compute indcov(((nms*nxvls)+1):nrow(mcsopath),((nms*nxvls)+1):nrow(mcsopath))= varb(wherem(1,atm):(wherem(1,atm)+nms-1),wherem(1,atm):(wherem(1,atm)+nms-1)).  compute mcsopath(((nms*nxvls)+1):nrow(mcsopath),1)=modres(wherem(1,atm):(wherem(1,atm)+ nms-1),1).  compute sobelok=1.  end if.  end if.  do if ((i = (nms+nys)) and (bcmat(nrow(bcmat),1)=1)).  compute direff=modres(2:(1+nxvls),:).  compute direfflb=modresl.  compute direffl2=vlabsm(2:(1+nxvls),:).  compute lmat=make(nrow(b),1,0).  compute lmat2=make(nxvls,1,1).  compute lmat(2:(1+nxvls),1)=lmat2.  do if (ydich <> 1).  .  compute lmat2= lmat.  do if ( 0 =0).  compute lmat2 = mdiag( lmat ).  compute lmat3=make(nrow(lmat2),1,0).  loop flp=1 to ncol(lmat2).  do if (csum(lmat2(:,flp))=1).  compute lmat3={lmat3,lmat2(:,flp)}.  end if.  end loop.  compute lmat2=lmat3(:,2:ncol(lmat3)).  end if.  compute fratio = (t(t(lmat2)* b )*inv(t(lmat2)* varb *lmat2)*((t(lmat2)* b )))/ncol(lmat2).  compute pfr = 1-fcdf(fratio,ncol(lmat2),(n-nrow( b ))).  compute fresult={fratio,ncol(lmat2),(n-nrow( b )),pfr}.  do if (i = (nms+nys) and (ydich=1)).  compute fratio=fratio*ncol(lmat2).  compute pfr=1-chicdf(fratio,ncol(lmat2)).  compute fresult={fratio,ncol(lmat2),pfr}.  end if.  do if ( 1 =1).  compute lmat3=1-rsum(lmat2).  compute xfm=make(n,csum(lmat3),0).  compute flpc=1.  loop flp=1 to nrow(lmat3).  do if (lmat3(flp,1)=1).  compute xfm(:,flpc)=x(:,flp).  compute flpc=flpc+1.  end if.  end loop.  compute bfm=inv(t(xfm)*xfm)*t(xfm)*y.  compute resid=y-(xfm*bfm).  compute sstotal=(y-(csum(y)/n)).  compute sstotal=csum(sstotal&*sstotal).  compute ssresid=csum(resid&*resid).  compute rsqch= r2 -((sstotal-ssresid)/sstotal).  compute fresult={rsqch,fresult}.  release xfm,flpc, resid, ssresid, bfm.  end if  .  compute diromni=fresult.  end if.  do if (ydich = 1 ).  .  compute btemphld=b.  compute llrdat=make(nrow(x),nrow( lmat )-csum( lmat ),-999).  compute llrdf=ncol(x)-ncol(llrdat).  compute llrcnt=0.  loop llri=1 to nrow( lmat ).  do if ( lmat (llri,1)=0).  compute llrcnt=llrcnt+1.  compute llrdat(:,llrcnt)=x(:,llri).  end if.  end loop.  .  do if ( 2 =1).  compute b = inv(t( llrdat )* llrdat )*t( llrdat )* y.  compute modres=b.  do if ( 0 =1).  compute n1=nrow( llrdat ).  compute dfres=n1-(ncol( llrdat )).  compute sstotal = t( y -(csum( y )/n1))*( y -(csum( y )/n1)).  compute resid= y - llrdat *b.  compute ssresid = csum((resid)&**2).  compute r2 = (sstotal-ssresid)/sstotal.  compute adjr2 = 1-((1-r2)*(n1-1)/(dfres)).  compute mse=ssresid/(n1-ncol( llrdat )).  .  compute n1=nrow( x ).  compute invXtX = inv(t( x )* x ).  compute varb = mse *invXtX.  compute k3 = ncol( x ).  compute xhc=0.  do if ( hc <> 5).  compute xhc= x.  compute hat = xhc(:,1).  loop i3=1 to nrow(xhc).  compute hat(i3,1)= xhc(i3,:)*invXtX*t(xhc(i3,:)).  end loop.  do if ( hc = 0 or hc =1).  loop i3 = 1 to k3.  compute xhc(:,i3)=xhc(:,i3)&* resid.  end loop.  end if.  do if ( hc =3 or hc =2).  loop i3=1 to k3.  compute xhc(:,i3) = ( resid &/(1-hat)&**(1/(4hc )))&*xhc(:,i3).  end loop.  end if.  do if ( hc = 4).  compute hcmn=make(n,2,4).  compute hcmn(:,2)=(n1*hat)/k3.  loop i3= 1 to k3.  compute xhc(:,i3) = ( resid &/(1-hat)&**(rmin(hcmn)/2))&*xhc(:,i3).  end loop.  end if.  compute varb=(invXtX*t(xhc)*xhc*invXtX).  do if ( hc =1).  compute varb=(n1/(n1-ncol( x )))&*varb.  end if.  end if.  compute hclab={'se(HC0)','se(HC1)','se(HC2)','se(HC3)','se(HC4)','se'}.  compute hclab=hclab(1,( hc +1)).  compute hcflab={'F(HC0)','F(HC1)','F(HC2)','F(HC3)','F(HC4)','F'}.  compute hcflab=hcflab(1,( hc +1)).  release xhc  .  compute seb=sqrt(diag(varb)).  compute trat = b&/seb.  compute p = 2*(1-tcdf(abs(trat), (dfres))).  compute tval = sqrt(dfres* (exp((dfres-(5/6))*((xp2/(dfres-(2/3)+(.11/dfres)))* (xp2/(dfres-(2/3)+(.11/dfres)))))-1)).  compute modres={modres,seb,trat,p}.  compute modres={modres,(b-tval&*seb),(b+tval&*seb)}.  compute modresl={'coeff',hclab,'t','p','LLCI','ULCI'}.  compute lmat = ident(ncol( llrdat )).  compute lmat = lmat(:,2:ncol(lmat)).  compute fratio = (t(t(lmat)*b)*inv(t(lmat)*varb*lmat)*((t(lmat)*b)))/(ncol( llrdat )-1).  compute pfr = 1-fcdf(fratio,(ncol( llrdat )-1),dfres).  compute modsum={sqrt(r2),r2,mse,fratio,(ncol( llrdat )-1),dfres,pfr}.  compute modsuml={'R','R-sq','MSE',hcflab,'df1','df2', 'p'}.  end if.  end if.  do if ( 2 = 2 or 2 =3).  compute xlp= llrdat.  compute ylp= y.  compute pt2 = make(nrow(ylp),1,(csum(ylp)/nrow(ylp))).  do if ( 2 =2).  compute LL3 = ylp&*ln(pt2)+(1-ylp)&*ln(1-pt2).  end if.  compute LL3 = -2*csum(LL3).  compute bt1 = make(ncol(xlp),1,0).  compute LL1 = 0.  compute pt1 = make(nrow(ylp),1,0.5).  compute pt1lp=pt1.  loop jjj = 1 to iterate.  compute vt1 = mdiag(pt1lp&*(1-pt1lp)).  compute b = bt1+inv(t(xlp)*vt1*xlp)*t(xlp)*(ylp-pt1lp).  do if ( 2 =2).  compute xlpb=xlp*b.  compute xlpbt=(xlpb > -709.7).  compute xlpb709=(1-xlpbt)*(-709.7).  compute xlpb=(xlpb&*xlpbt)+xlpb709.  compute pt1lp = 1/(1+exp(-(xlpb))).  end if.  compute itprob = csum((pt1lp < .00000001) or (pt1lp > .9999999)).  do if (itprob > 0).  loop kkk = 1 to nrow(pt1lp).  do if (pt1lp(kkk,1) > .9999999).  compute pt1lp(kkk,1) = .9999999.  end if.  do if (pt1lp(kkk,1) < .00000001).  compute pt1lp(kkk,1) = .00000001.  end if.  end loop.  compute itprob = 0.  end if.  do if (itprob = 0).  do if ( 2 =2).  compute LL = ylp&*ln(pt1lp)+(1-ylp)&*ln(1-pt1lp).  end if.  compute LL2 = -2*csum(ll).  end if.  do if (abs(LL1-LL2) < converge).  do if ( 0 =1).  compute vt1 = mdiag(pt1lp&*(1-pt1lp)).  compute varb = inv(t(xlp)*vt1*xlp).  compute seb = sqrt(diag(varb)).  end if.  break.  end if.  compute bt1 = b.  compute LL1 = LL2.  end loop.  compute modres=b.  do if (jjj > iterate).  compute itprob = 2.  do if (booting=0).  compute iterrmod=1.  end if.  do if (booting=1).  compute bootiter=1.  end if.  do if (itprobtg=0).  compute itprobtg=1.  compute errcode(errs,1) = 47.  compute errs = errs + 1.  do if (booting = 0 and 0 =1).  compute vt1 = mdiag(pt1lp&*(1-pt1lp)).  compute varb = inv(t(xlp)*vt1*xlp).  compute seb = sqrt(diag(varb)).  end if.  end if.  end if.  do if ( 0 =1).  compute trat = b&/seb.  compute dfres=nrow(xlp).  compute p = 2*(1-cdfnorm(abs(trat))).  compute modres={modres,seb,trat,p}.  compute modres={modres,(b-xp2&*seb),(b+xp2&*seb)}.  compute pvchi=1-chicdf((LL3-LL2),(nrow(modres)-1)).  compute mcF = (LL3-LL2)/LL3.  compute cox = 1-exp(-(LL3-LL2)/nrow(xlp)).  compute nagel = cox/(1-exp(-(LL3)/nrow(xlp))).  compute modsum={LL2,(LL3-LL2),(nrow(modres)-1),pvchi, mcF,cox,nagel}.  compute modsuml={'-2LL','ModelLL', 'df', 'p', 'McFadden', 'CoxSnell', 'Nagelkrk'}.  compute modresl={'coeff','se','Z','p','LLCI','ULCI'}.  end if.  end if  .  compute b=btemphld.  compute fresult={(LL2-basemod),llrdf,1-chicdf((LL2-basemod),llrdf)}  .  compute diromni=fresult.  end if.  end if.  do if (numint(1,i) > 0).  compute intkeym=intkey(intstart:(intstart+numint(1,i)-1),:).  print intkeym/title='Product terms key:'/format = A8.  end if.  do if (covcoeff=1).  print varb/title='Covariance matrix of regression parameter estimates:'/rnames=vlabsm /cnames=vlabsm/format= F10.4.  end if.  do if (model <> 74 and xmtest=1 and nms > 0).  compute r2tmp=r2.  compute btmp=b.  compute varbtmp=varb.  compute dfrestmp=dfres.  compute tvaltmp=tval.  compute xmtst=make(nms,4,0).  compute xmtstlbc={hcflab,'df1','df2','p'}.  do if ((i=(nms+nys)) and (ydich=1)).  compute xmtst=make(nms,3,0).  compute xmtstlbc={'Chi-sq','df','p'}.  end if.  compute xmtstlb={' '}.  compute xmtmat=x.  compute numxint=0.  loop xmint=2 to i.  compute x=xmtmat.  do if ((bcmat((i+1),xmint)=1) and (wzcmat((i+1),xmint) <> 1)).  do if (bcmat((i+1),1)=0).  compute x={xmtmat,xtmp}.  do if ((ydich=1) and (i=(nms+nys))).  .  do if ( 2 =1).  compute b = inv(t( x )* x )*t( x )* y.  compute modres=b.  do if ( 1 =1).  compute n1=nrow( x ).  compute dfres=n1-(ncol( x )).  compute sstotal = t( y -(csum( y )/n1))*( y -(csum( y )/n1)).  compute resid= y - x *b.  compute ssresid = csum((resid)&**2).  compute r2 = (sstotal-ssresid)/sstotal.  compute adjr2 = 1-((1-r2)*(n1-1)/(dfres)).  compute mse=ssresid/(n1-ncol( x )).  .  compute n1=nrow( x ).  compute invXtX = inv(t( x )* x ).  compute varb = mse *invXtX.  compute k3 = ncol( x ).  compute xhc=0.  do if ( hc <> 5).  compute xhc= x.  compute hat = xhc(:,1).  loop i3=1 to nrow(xhc).  compute hat(i3,1)= xhc(i3,:)*invXtX*t(xhc(i3,:)).  end loop.  do if ( hc = 0 or hc =1).  loop i3 = 1 to k3.  compute xhc(:,i3)=xhc(:,i3)&* resid.  end loop.  end if.  do if ( hc =3 or hc =2).  loop i3=1 to k3.  compute xhc(:,i3) = ( resid &/(1-hat)&**(1/(4hc )))&*xhc(:,i3).  end loop.  end if.  do if ( hc = 4).  compute hcmn=make(n,2,4).  compute hcmn(:,2)=(n1*hat)/k3.  loop i3= 1 to k3.  compute xhc(:,i3) = ( resid &/(1-hat)&**(rmin(hcmn)/2))&*xhc(:,i3).  end loop.  end if.  compute varb=(invXtX*t(xhc)*xhc*invXtX).  do if ( hc =1).  compute varb=(n1/(n1-ncol( x )))&*varb.  end if.  end if.  compute hclab={'se(HC0)','se(HC1)','se(HC2)','se(HC3)','se(HC4)','se'}.  compute hclab=hclab(1,( hc +1)).  compute hcflab={'F(HC0)','F(HC1)','F(HC2)','F(HC3)','F(HC4)','F'}.  compute hcflab=hcflab(1,( hc +1)).  release xhc  .  compute seb=sqrt(diag(varb)).  compute trat = b&/seb.  compute p = 2*(1-tcdf(abs(trat), (dfres))).  compute tval = sqrt(dfres* (exp((dfres-(5/6))*((xp2/(dfres-(2/3)+(.11/dfres)))* (xp2/(dfres-(2/3)+(.11/dfres)))))-1)).  compute modres={modres,seb,trat,p}.  compute modres={modres,(b-tval&*seb),(b+tval&*seb)}.  compute modresl={'coeff',hclab,'t','p','LLCI','ULCI'}.  compute lmat = ident(ncol( x )).  compute lmat = lmat(:,2:ncol(lmat)).  compute fratio = (t(t(lmat)*b)*inv(t(lmat)*varb*lmat)*((t(lmat)*b)))/(ncol( x )-1).  compute pfr = 1-fcdf(fratio,(ncol( x )-1),dfres).  compute modsum={sqrt(r2),r2,mse,fratio,(ncol( x )-1),dfres,pfr}.  compute modsuml={'R','R-sq','MSE',hcflab,'df1','df2', 'p'}.  end if.  end if.  do if ( 2 = 2 or 2 =3).  compute xlp= x.  compute ylp= y.  compute pt2 = make(nrow(ylp),1,(csum(ylp)/nrow(ylp))).  do if ( 2 =2).  compute LL3 = ylp&*ln(pt2)+(1-ylp)&*ln(1-pt2).  end if.  compute LL3 = -2*csum(LL3).  compute bt1 = make(ncol(xlp),1,0).  compute LL1 = 0.  compute pt1 = make(nrow(ylp),1,0.5).  compute pt1lp=pt1.  loop jjj = 1 to iterate.  compute vt1 = mdiag(pt1lp&*(1-pt1lp)).  compute b = bt1+inv(t(xlp)*vt1*xlp)*t(xlp)*(ylp-pt1lp).  do if ( 2 =2).  compute xlpb=xlp*b.  compute xlpbt=(xlpb > -709.7).  compute xlpb709=(1-xlpbt)*(-709.7).  compute xlpb=(xlpb&*xlpbt)+xlpb709.  compute pt1lp = 1/(1+exp(-(xlpb))).  end if.  compute itprob = csum((pt1lp < .00000001) or (pt1lp > .9999999)).  do if (itprob > 0).  loop kkk = 1 to nrow(pt1lp).  do if (pt1lp(kkk,1) > .9999999).  compute pt1lp(kkk,1) = .9999999.  end if.  do if (pt1lp(kkk,1) < .00000001).  compute pt1lp(kkk,1) = .00000001.  end if.  end loop.  compute itprob = 0.  end if.  do if (itprob = 0).  do if ( 2 =2).  compute LL = ylp&*ln(pt1lp)+(1-ylp)&*ln(1-pt1lp).  end if.  compute LL2 = -2*csum(ll).  end if.  do if (abs(LL1-LL2) < converge).  do if ( 1 =1).  compute vt1 = mdiag(pt1lp&*(1-pt1lp)).  compute varb = inv(t(xlp)*vt1*xlp).  compute seb = sqrt(diag(varb)).  end if.  break.  end if.  compute bt1 = b.  compute LL1 = LL2.  end loop.  compute modres=b.  do if (jjj > iterate).  compute itprob = 2.  do if (booting=0).  compute iterrmod=1.  end if.  do if (booting=1).  compute bootiter=1.  end if.  do if (itprobtg=0).  compute itprobtg=1.  compute errcode(errs,1) = 47.  compute errs = errs + 1.  do if (booting = 0 and 1 =1).  compute vt1 = mdiag(pt1lp&*(1-pt1lp)).  compute varb = inv(t(xlp)*vt1*xlp).  compute seb = sqrt(diag(varb)).  end if.  end if.  end if.  do if ( 1 =1).  compute trat = b&/seb.  compute dfres=nrow(xlp).  compute p = 2*(1-cdfnorm(abs(trat))).  compute modres={modres,seb,trat,p}.  compute modres={modres,(b-xp2&*seb),(b+xp2&*seb)}.  compute pvchi=1-chicdf((LL3-LL2),(nrow(modres)-1)).  compute mcF = (LL3-LL2)/LL3.  compute cox = 1-exp(-(LL3-LL2)/nrow(xlp)).  compute nagel = cox/(1-exp(-(LL3)/nrow(xlp))).  compute modsum={LL2,(LL3-LL2),(nrow(modres)-1),pvchi, mcF,cox,nagel}.  compute modsuml={'-2LL','ModelLL', 'df', 'p', 'McFadden', 'CoxSnell', 'Nagelkrk'}.  compute modresl={'coeff','se','Z','p','LLCI','ULCI'}.  end if.  end if  .  compute basemodx=LL2.  end if.  end if.  loop xmtlp1=1 to nxvls.  compute x={x,xtmp(:,xmtlp1)&*(mtmp(:,(xmint-1))-(csum(mtmp(:,(xmint-1)))/nrow(mtmp))) }.  end loop.  do if ((i < (nms+nys)) or (ydich=0)).  .  do if ( 1 =1).  compute b = inv(t( x )* x )*t( x )* y.  compute modres=b.  do if ( 1 =1).  compute n1=nrow( x ).  compute dfres=n1-(ncol( x )).  compute sstotal = t( y -(csum( y )/n1))*( y -(csum( y )/n1)).  compute resid= y - x *b.  compute ssresid = csum((resid)&**2).  compute r2 = (sstotal-ssresid)/sstotal.  compute adjr2 = 1-((1-r2)*(n1-1)/(dfres)).  compute mse=ssresid/(n1-ncol( x )).  .  compute n1=nrow( x ).  compute invXtX = inv(t( x )* x ).  compute varb = mse *invXtX.  compute k3 = ncol( x ).  compute xhc=0.  do if ( hc <> 5).  compute xhc= x.  compute hat = xhc(:,1).  loop i3=1 to nrow(xhc).  compute hat(i3,1)= xhc(i3,:)*invXtX*t(xhc(i3,:)).  end loop.  do if ( hc = 0 or hc =1).  loop i3 = 1 to k3.  compute xhc(:,i3)=xhc(:,i3)&* resid.  end loop.  end if.  do if ( hc =3 or hc =2).  loop i3=1 to k3.  compute xhc(:,i3) = ( resid &/(1-hat)&**(1/(4hc )))&*xhc(:,i3).  end loop.  end if.  do if ( hc = 4).  compute hcmn=make(n,2,4).  compute hcmn(:,2)=(n1*hat)/k3.  loop i3= 1 to k3.  compute xhc(:,i3) = ( resid &/(1-hat)&**(rmin(hcmn)/2))&*xhc(:,i3).  end loop.  end if.  compute varb=(invXtX*t(xhc)*xhc*invXtX).  do if ( hc =1).  compute varb=(n1/(n1-ncol( x )))&*varb.  end if.  end if.  compute hclab={'se(HC0)','se(HC1)','se(HC2)','se(HC3)','se(HC4)','se'}.  compute hclab=hclab(1,( hc +1)).  compute hcflab={'F(HC0)','F(HC1)','F(HC2)','F(HC3)','F(HC4)','F'}.  compute hcflab=hcflab(1,( hc +1)).  release xhc  .  compute seb=sqrt(diag(varb)).  compute trat = b&/seb.  compute p = 2*(1-tcdf(abs(trat), (dfres))).  compute tval = sqrt(dfres* (exp((dfres-(5/6))*((xp2/(dfres-(2/3)+(.11/dfres)))* (xp2/(dfres-(2/3)+(.11/dfres)))))-1)).  compute modres={modres,seb,trat,p}.  compute modres={modres,(b-tval&*seb),(b+tval&*seb)}.  compute modresl={'coeff',hclab,'t','p','LLCI','ULCI'}.  compute lmat = ident(ncol( x )).  compute lmat = lmat(:,2:ncol(lmat)).  compute fratio = (t(t(lmat)*b)*inv(t(lmat)*varb*lmat)*((t(lmat)*b)))/(ncol( x )-1).  compute pfr = 1-fcdf(fratio,(ncol( x )-1),dfres).  compute modsum={sqrt(r2),r2,mse,fratio,(ncol( x )-1),dfres,pfr}.  compute modsuml={'R','R-sq','MSE',hcflab,'df1','df2', 'p'}.  end if.  end if.  do if ( 1 = 2 or 1 =3).  compute xlp= x.  compute ylp= y.  compute pt2 = make(nrow(ylp),1,(csum(ylp)/nrow(ylp))).  do if ( 1 =2).  compute LL3 = ylp&*ln(pt2)+(1-ylp)&*ln(1-pt2).  end if.  compute LL3 = -2*csum(LL3).  compute bt1 = make(ncol(xlp),1,0).  compute LL1 = 0.  compute pt1 = make(nrow(ylp),1,0.5).  compute pt1lp=pt1.  loop jjj = 1 to iterate.  compute vt1 = mdiag(pt1lp&*(1-pt1lp)).  compute b = bt1+inv(t(xlp)*vt1*xlp)*t(xlp)*(ylp-pt1lp).  do if ( 1 =2).  compute xlpb=xlp*b.  compute xlpbt=(xlpb > -709.7).  compute xlpb709=(1-xlpbt)*(-709.7).  compute xlpb=(xlpb&*xlpbt)+xlpb709.  compute pt1lp = 1/(1+exp(-(xlpb))).  end if.  compute itprob = csum((pt1lp < .00000001) or (pt1lp > .9999999)).  do if (itprob > 0).  loop kkk = 1 to nrow(pt1lp).  do if (pt1lp(kkk,1) > .9999999).  compute pt1lp(kkk,1) = .9999999.  end if.  do if (pt1lp(kkk,1) < .00000001).  compute pt1lp(kkk,1) = .00000001.  end if.  end loop.  compute itprob = 0.  end if.  do if (itprob = 0).  do if ( 1 =2).  compute LL = ylp&*ln(pt1lp)+(1-ylp)&*ln(1-pt1lp).  end if.  compute LL2 = -2*csum(ll).  end if.  do if (abs(LL1-LL2) < converge).  do if ( 1 =1).  compute vt1 = mdiag(pt1lp&*(1-pt1lp)).  compute varb = inv(t(xlp)*vt1*xlp).  compute seb = sqrt(diag(varb)).  end if.  break.  end if.  compute bt1 = b.  compute LL1 = LL2.  end loop.  compute modres=b.  do if (jjj > iterate).  compute itprob = 2.  do if (booting=0).  compute iterrmod=1.  end if.  do if (booting=1).  compute bootiter=1.  end if.  do if (itprobtg=0).  compute itprobtg=1.  compute errcode(errs,1) = 47.  compute errs = errs + 1.  do if (booting = 0 and 1 =1).  compute vt1 = mdiag(pt1lp&*(1-pt1lp)).  compute varb = inv(t(xlp)*vt1*xlp).  compute seb = sqrt(diag(varb)).  end if.  end if.  end if.  do if ( 1 =1).  compute trat = b&/seb.  compute dfres=nrow(xlp).  compute p = 2*(1-cdfnorm(abs(trat))).  compute modres={modres,seb,trat,p}.  compute modres={modres,(b-xp2&*seb),(b+xp2&*seb)}.  compute pvchi=1-chicdf((LL3-LL2),(nrow(modres)-1)).  compute mcF = (LL3-LL2)/LL3.  compute cox = 1-exp(-(LL3-LL2)/nrow(xlp)).  compute nagel = cox/(1-exp(-(LL3)/nrow(xlp))).  compute modsum={LL2,(LL3-LL2),(nrow(modres)-1),pvchi, mcF,cox,nagel}.  compute modsuml={'-2LL','ModelLL', 'df', 'p', 'McFadden', 'CoxSnell', 'Nagelkrk'}.  compute modresl={'coeff','se','Z','p','LLCI','ULCI'}.  end if.  end if  .  end if.  do if ((i = (nms+nys)) and (ydich=1)).  .  do if ( 2 =1).  compute b = inv(t( x )* x )*t( x )* y.  compute modres=b.  do if ( 1 =1).  compute n1=nrow( x ).  compute dfres=n1-(ncol( x )).  compute sstotal = t( y -(csum( y )/n1))*( y -(csum( y )/n1)).  compute resid= y - x *b.  compute ssresid = csum((resid)&**2).  compute r2 = (sstotal-ssresid)/sstotal.  compute adjr2 = 1-((1-r2)*(n1-1)/(dfres)).  compute mse=ssresid/(n1-ncol( x )).  .  compute n1=nrow( x ).  compute invXtX = inv(t( x )* x ).  compute varb = mse *invXtX.  compute k3 = ncol( x ).  compute xhc=0.  do if ( hc <> 5).  compute xhc= x.  compute hat = xhc(:,1).  loop i3=1 to nrow(xhc).  compute hat(i3,1)= xhc(i3,:)*invXtX*t(xhc(i3,:)).  end loop.  do if ( hc = 0 or hc =1).  loop i3 = 1 to k3.  compute xhc(:,i3)=xhc(:,i3)&* resid.  end loop.  end if.  do if ( hc =3 or hc =2).  loop i3=1 to k3.  compute xhc(:,i3) = ( resid &/(1-hat)&**(1/(4hc )))&*xhc(:,i3).  end loop.  end if.  do if ( hc = 4).  compute hcmn=make(n,2,4).  compute hcmn(:,2)=(n1*hat)/k3.  loop i3= 1 to k3.  compute xhc(:,i3) = ( resid &/(1-hat)&**(rmin(hcmn)/2))&*xhc(:,i3).  end loop.  end if.  compute varb=(invXtX*t(xhc)*xhc*invXtX).  do if ( hc =1).  compute varb=(n1/(n1-ncol( x )))&*varb.  end if.  end if.  compute hclab={'se(HC0)','se(HC1)','se(HC2)','se(HC3)','se(HC4)','se'}.  compute hclab=hclab(1,( hc +1)).  compute hcflab={'F(HC0)','F(HC1)','F(HC2)','F(HC3)','F(HC4)','F'}.  compute hcflab=hcflab(1,( hc +1)).  release xhc  .  compute seb=sqrt(diag(varb)).  compute trat = b&/seb.  compute p = 2*(1-tcdf(abs(trat), (dfres))).  compute tval = sqrt(dfres* (exp((dfres-(5/6))*((xp2/(dfres-(2/3)+(.11/dfres)))* (xp2/(dfres-(2/3)+(.11/dfres)))))-1)).  compute modres={modres,seb,trat,p}.  compute modres={modres,(b-tval&*seb),(b+tval&*seb)}.  compute modresl={'coeff',hclab,'t','p','LLCI','ULCI'}.  compute lmat = ident(ncol( x )).  compute lmat = lmat(:,2:ncol(lmat)).  compute fratio = (t(t(lmat)*b)*inv(t(lmat)*varb*lmat)*((t(lmat)*b)))/(ncol( x )-1).  compute pfr = 1-fcdf(fratio,(ncol( x )-1),dfres).  compute modsum={sqrt(r2),r2,mse,fratio,(ncol( x )-1),dfres,pfr}.  compute modsuml={'R','R-sq','MSE',hcflab,'df1','df2', 'p'}.  end if.  end if.  do if ( 2 = 2 or 2 =3).  compute xlp= x.  compute ylp= y.  compute pt2 = make(nrow(ylp),1,(csum(ylp)/nrow(ylp))).  do if ( 2 =2).  compute LL3 = ylp&*ln(pt2)+(1-ylp)&*ln(1-pt2).  end if.  compute LL3 = -2*csum(LL3).  compute bt1 = make(ncol(xlp),1,0).  compute LL1 = 0.  compute pt1 = make(nrow(ylp),1,0.5).  compute pt1lp=pt1.  loop jjj = 1 to iterate.  compute vt1 = mdiag(pt1lp&*(1-pt1lp)).  compute b = bt1+inv(t(xlp)*vt1*xlp)*t(xlp)*(ylp-pt1lp).  do if ( 2 =2).  compute xlpb=xlp*b.  compute xlpbt=(xlpb > -709.7).  compute xlpb709=(1-xlpbt)*(-709.7).  compute xlpb=(xlpb&*xlpbt)+xlpb709.  compute pt1lp = 1/(1+exp(-(xlpb))).  end if.  compute itprob = csum((pt1lp < .00000001) or (pt1lp > .9999999)).  do if (itprob > 0).  loop kkk = 1 to nrow(pt1lp).  do if (pt1lp(kkk,1) > .9999999).  compute pt1lp(kkk,1) = .9999999.  end if.  do if (pt1lp(kkk,1) < .00000001).  compute pt1lp(kkk,1) = .00000001.  end if.  end loop.  compute itprob = 0.  end if.  do if (itprob = 0).  do if ( 2 =2).  compute LL = ylp&*ln(pt1lp)+(1-ylp)&*ln(1-pt1lp).  end if.  compute LL2 = -2*csum(ll).  end if.  do if (abs(LL1-LL2) < converge).  do if ( 1 =1).  compute vt1 = mdiag(pt1lp&*(1-pt1lp)).  compute varb = inv(t(xlp)*vt1*xlp).  compute seb = sqrt(diag(varb)).  end if.  break.  end if.  compute bt1 = b.  compute LL1 = LL2.  end loop.  compute modres=b.  do if (jjj > iterate).  compute itprob = 2.  do if (booting=0).  compute iterrmod=1.  end if.  do if (booting=1).  compute bootiter=1.  end if.  do if (itprobtg=0).  compute itprobtg=1.  compute errcode(errs,1) = 47.  compute errs = errs + 1.  do if (booting = 0 and 1 =1).  compute vt1 = mdiag(pt1lp&*(1-pt1lp)).  compute varb = inv(t(xlp)*vt1*xlp).  compute seb = sqrt(diag(varb)).  end if.  end if.  end if.  do if ( 1 =1).  compute trat = b&/seb.  compute dfres=nrow(xlp).  compute p = 2*(1-cdfnorm(abs(trat))).  compute modres={modres,seb,trat,p}.  compute modres={modres,(b-xp2&*seb),(b+xp2&*seb)}.  compute pvchi=1-chicdf((LL3-LL2),(nrow(modres)-1)).  compute mcF = (LL3-LL2)/LL3.  compute cox = 1-exp(-(LL3-LL2)/nrow(xlp)).  compute nagel = cox/(1-exp(-(LL3)/nrow(xlp))).  compute modsum={LL2,(LL3-LL2),(nrow(modres)-1),pvchi, mcF,cox,nagel}.  compute modsuml={'-2LL','ModelLL', 'df', 'p', 'McFadden', 'CoxSnell', 'Nagelkrk'}.  compute modresl={'coeff','se','Z','p','LLCI','ULCI'}.  end if.  end if  .  compute chidfxm=basemodx-LL2.  end if.  compute lmat=make(nrow(b),nxvls,0).  compute lmattmp=ident(nxvls).  compute lmat((nrow(lmat)-nxvls+1):nrow(lmat),:)=lmattmp.  .  compute lmat2= lmat.  do if ( 1 =0).  compute lmat2 = mdiag( lmat ).  compute lmat3=make(nrow(lmat2),1,0).  loop flp=1 to ncol(lmat2).  do if (csum(lmat2(:,flp))=1).  compute lmat3={lmat3,lmat2(:,flp)}.  end if.  end loop.  compute lmat2=lmat3(:,2:ncol(lmat3)).  end if.  compute fratio = (t(t(lmat2)* b )*inv(t(lmat2)* varb *lmat2)*((t(lmat2)* b )))/ncol(lmat2).  compute pfr = 1-fcdf(fratio,ncol(lmat2),(n-nrow( b ))).  compute fresult={fratio,ncol(lmat2),(n-nrow( b )),pfr}.  do if (i = (nms+nys) and (ydich=1)).  compute fratio=fratio*ncol(lmat2).  compute pfr=1-chicdf(fratio,ncol(lmat2)).  compute fresult={fratio,ncol(lmat2),pfr}.  end if.  do if ( 0 =1).  compute lmat3=1-rsum(lmat2).  compute xfm=make(n,csum(lmat3),0).  compute flpc=1.  loop flp=1 to nrow(lmat3).  do if (lmat3(flp,1)=1).  compute xfm(:,flpc)=x(:,flp).  compute flpc=flpc+1.  end if.  end loop.  compute bfm=inv(t(xfm)*xfm)*t(xfm)*y.  compute resid=y-(xfm*bfm).  compute sstotal=(y-(csum(y)/n)).  compute sstotal=csum(sstotal&*sstotal).  compute ssresid=csum(resid&*resid).  compute rsqch= 0 -((sstotal-ssresid)/sstotal).  compute fresult={rsqch,fresult}.  release xfm,flpc, resid, ssresid, bfm.  end if  .  compute numxint=numxint+1.  compute xmtst(numxint,:)=fresult.  do if ((i = (nms+nys)) and (ydich=1)).  compute xmtst(numxint,1)=chidfxm.  compute xmtst(numxint,3)=1-chicdf(chidfxm,nxvls).  end if.  compute xmtstlb={xmtstlb;highlbx((xmint-1),1)}.  end if.  end loop.  compute x=xmtmat.  release xmtmat.  do if (numxint>0).  compute xmtstlb=xmtstlb((2:(numxint+1)),:).  compute xmtst=xmtst(1:numxint,:).  do if (nms=1).  compute xmtstlb=' '.  end if.  do if ((i < (nms+nys)) or (ydich=0)).  print xmtst/title='Test(s) of X by M interaction:'/rnames=xmtstlb/cnames=xmtstlbc/format= F10.4.  end if.  do if ((i = (nms+nys)) and (ydich=1)).  print xmtst/title='Likelihood ratio test(s) of X by M interaction:'/rnames=xmtstlb/cnames=xmtstlbc/format= F10.4.  end if.  end if.  compute r2=r2tmp.  compute b=btmp.  compute varb=varbtmp.  compute dfres=dfrestmp.  compute tval=tvaltmp.  end if.  do if (criterr = 0).  compute jj=0.  loop j = start to ((start+i)-1).  compute dbint=0.  compute lmat=whigh(1:nump(1,i),j).  compute lmat2=wzhigh(1:nump(1,i),j).  do if ((csum(lmat) > 0) and (csum(lmat2) = 0)).  do if ((i < (nms+nys)) or (ydich <> 1)).  .  compute lmat2= lmat.  do if ( 0 =0).  compute lmat2 = mdiag( lmat ).  compute lmat3=make(nrow(lmat2),1,0).  loop flp=1 to ncol(lmat2).  do if (csum(lmat2(:,flp))=1).  compute lmat3={lmat3,lmat2(:,flp)}.  end if.  end loop.  compute lmat2=lmat3(:,2:ncol(lmat3)).  end if.  compute fratio = (t(t(lmat2)* b )*inv(t(lmat2)* varb *lmat2)*((t(lmat2)* b )))/ncol(lmat2).  compute pfr = 1-fcdf(fratio,ncol(lmat2),(n-nrow( b ))).  compute fresult={fratio,ncol(lmat2),(n-nrow( b )),pfr}.  do if (i = (nms+nys) and (ydich=1)).  compute fratio=fratio*ncol(lmat2).  compute pfr=1-chicdf(fratio,ncol(lmat2)).  compute fresult={fratio,ncol(lmat2),pfr}.  end if.  do if ( 1 =1).  compute lmat3=1-rsum(lmat2).  compute xfm=make(n,csum(lmat3),0).  compute flpc=1.  loop flp=1 to nrow(lmat3).  do if (lmat3(flp,1)=1).  compute xfm(:,flpc)=x(:,flp).  compute flpc=flpc+1.  end if.  end loop.  compute bfm=inv(t(xfm)*xfm)*t(xfm)*y.  compute resid=y-(xfm*bfm).  compute sstotal=(y-(csum(y)/n)).  compute sstotal=csum(sstotal&*sstotal).  compute ssresid=csum(resid&*resid).  compute rsqch= r2 -((sstotal-ssresid)/sstotal).  compute fresult={rsqch,fresult}.  release xfm,flpc, resid, ssresid, bfm.  end if  .  compute lmatdb=lmat.  compute dbint=dbint+1.  end if.  do if ((ydich = 1) and (i = (nms+nys))).  .  compute btemphld=b.  compute llrdat=make(nrow(x),nrow( lmat )-csum( lmat ),-999).  compute llrdf=ncol(x)-ncol(llrdat).  compute llrcnt=0.  loop llri=1 to nrow( lmat ).  do if ( lmat (llri,1)=0).  compute llrcnt=llrcnt+1.  compute llrdat(:,llrcnt)=x(:,llri).  end if.  end loop.  .  do if ( 2 =1).  compute b = inv(t( llrdat )* llrdat )*t( llrdat )* y.  compute modres=b.  do if ( 0 =1).  compute n1=nrow( llrdat ).  compute dfres=n1-(ncol( llrdat )).  compute sstotal = t( y -(csum( y )/n1))*( y -(csum( y )/n1)).  compute resid= y - llrdat *b.  compute ssresid = csum((resid)&**2).  compute r2 = (sstotal-ssresid)/sstotal.  compute adjr2 = 1-((1-r2)*(n1-1)/(dfres)).  compute mse=ssresid/(n1-ncol( llrdat )).  .  compute n1=nrow( x ).  compute invXtX = inv(t( x )* x ).  compute varb = mse *invXtX.  compute k3 = ncol( x ).  compute xhc=0.  do if ( hc <> 5).  compute xhc= x.  compute hat = xhc(:,1).  loop i3=1 to nrow(xhc).  compute hat(i3,1)= xhc(i3,:)*invXtX*t(xhc(i3,:)).  end loop.  do if ( hc = 0 or hc =1).  loop i3 = 1 to k3.  compute xhc(:,i3)=xhc(:,i3)&* resid.  end loop.  end if.  do if ( hc =3 or hc =2).  loop i3=1 to k3.  compute xhc(:,i3) = ( resid &/(1-hat)&**(1/(4hc )))&*xhc(:,i3).  end loop.  end if.  do if ( hc = 4).  compute hcmn=make(n,2,4).  compute hcmn(:,2)=(n1*hat)/k3.  loop i3= 1 to k3.  compute xhc(:,i3) = ( resid &/(1-hat)&**(rmin(hcmn)/2))&*xhc(:,i3).  end loop.  end if.  compute varb=(invXtX*t(xhc)*xhc*invXtX).  do if ( hc =1).  compute varb=(n1/(n1-ncol( x )))&*varb.  end if.  end if.  compute hclab={'se(HC0)','se(HC1)','se(HC2)','se(HC3)','se(HC4)','se'}.  compute hclab=hclab(1,( hc +1)).  compute hcflab={'F(HC0)','F(HC1)','F(HC2)','F(HC3)','F(HC4)','F'}.  compute hcflab=hcflab(1,( hc +1)).  release xhc  .  compute seb=sqrt(diag(varb)).  compute trat = b&/seb.  compute p = 2*(1-tcdf(abs(trat), (dfres))).  compute tval = sqrt(dfres* (exp((dfres-(5/6))*((xp2/(dfres-(2/3)+(.11/dfres)))* (xp2/(dfres-(2/3)+(.11/dfres)))))-1)).  compute modres={modres,seb,trat,p}.  compute modres={modres,(b-tval&*seb),(b+tval&*seb)}.  compute modresl={'coeff',hclab,'t','p','LLCI','ULCI'}.  compute lmat = ident(ncol( llrdat )).  compute lmat = lmat(:,2:ncol(lmat)).  compute fratio = (t(t(lmat)*b)*inv(t(lmat)*varb*lmat)*((t(lmat)*b)))/(ncol( llrdat )-1).  compute pfr = 1-fcdf(fratio,(ncol( llrdat )-1),dfres).  compute modsum={sqrt(r2),r2,mse,fratio,(ncol( llrdat )-1),dfres,pfr}.  compute modsuml={'R','R-sq','MSE',hcflab,'df1','df2', 'p'}.  end if.  end if.  do if ( 2 = 2 or 2 =3).  compute xlp= llrdat.  compute ylp= y.  compute pt2 = make(nrow(ylp),1,(csum(ylp)/nrow(ylp))).  do if ( 2 =2).  compute LL3 = ylp&*ln(pt2)+(1-ylp)&*ln(1-pt2).  end if.  compute LL3 = -2*csum(LL3).  compute bt1 = make(ncol(xlp),1,0).  compute LL1 = 0.  compute pt1 = make(nrow(ylp),1,0.5).  compute pt1lp=pt1.  loop jjj = 1 to iterate.  compute vt1 = mdiag(pt1lp&*(1-pt1lp)).  compute b = bt1+inv(t(xlp)*vt1*xlp)*t(xlp)*(ylp-pt1lp).  do if ( 2 =2).  compute xlpb=xlp*b.  compute xlpbt=(xlpb > -709.7).  compute xlpb709=(1-xlpbt)*(-709.7).  compute xlpb=(xlpb&*xlpbt)+xlpb709.  compute pt1lp = 1/(1+exp(-(xlpb))).  end if.  compute itprob = csum((pt1lp < .00000001) or (pt1lp > .9999999)).  do if (itprob > 0).  loop kkk = 1 to nrow(pt1lp).  do if (pt1lp(kkk,1) > .9999999).  compute pt1lp(kkk,1) = .9999999.  end if.  do if (pt1lp(kkk,1) < .00000001).  compute pt1lp(kkk,1) = .00000001.  end if.  end loop.  compute itprob = 0.  end if.  do if (itprob = 0).  do if ( 2 =2).  compute LL = ylp&*ln(pt1lp)+(1-ylp)&*ln(1-pt1lp).  end if.  compute LL2 = -2*csum(ll).  end if.  do if (abs(LL1-LL2) < converge).  do if ( 0 =1).  compute vt1 = mdiag(pt1lp&*(1-pt1lp)).  compute varb = inv(t(xlp)*vt1*xlp).  compute seb = sqrt(diag(varb)).  end if.  break.  end if.  compute bt1 = b.  compute LL1 = LL2.  end loop.  compute modres=b.  do if (jjj > iterate).  compute itprob = 2.  do if (booting=0).  compute iterrmod=1.  end if.  do if (booting=1).  compute bootiter=1.  end if.  do if (itprobtg=0).  compute itprobtg=1.  compute errcode(errs,1) = 47.  compute errs = errs + 1.  do if (booting = 0 and 0 =1).  compute vt1 = mdiag(pt1lp&*(1-pt1lp)).  compute varb = inv(t(xlp)*vt1*xlp).  compute seb = sqrt(diag(varb)).  end if.  end if.  end if.  do if ( 0 =1).  compute trat = b&/seb.  compute dfres=nrow(xlp).  compute p = 2*(1-cdfnorm(abs(trat))).  compute modres={modres,seb,trat,p}.  compute modres={modres,(b-xp2&*seb),(b+xp2&*seb)}.  compute pvchi=1-chicdf((LL3-LL2),(nrow(modres)-1)).  compute mcF = (LL3-LL2)/LL3.  compute cox = 1-exp(-(LL3-LL2)/nrow(xlp)).  compute nagel = cox/(1-exp(-(LL3)/nrow(xlp))).  compute modsum={LL2,(LL3-LL2),(nrow(modres)-1),pvchi, mcF,cox,nagel}.  compute modsuml={'-2LL','ModelLL', 'df', 'p', 'McFadden', 'CoxSnell', 'Nagelkrk'}.  compute modresl={'coeff','se','Z','p','LLCI','ULCI'}.  end if.  end if  .  compute b=btemphld.  compute fresult={(LL2-basemod),llrdf,1-chicdf((LL2-basemod),llrdf)}  .  compute lmatdb=lmat.  compute dbint=dbint+1.  end if.  compute highf={highf;fresult}.  compute highf2={highf2;fresult}.  do if (j = start).  compute flabel={flabel;'X*W'}.  end if.  do if (j > start).  do if (nms > 1).  compute flabel={flabel;highlbw(jj,1)}.  else if (nms = 1).  compute flabel={flabel;'M*W'}.  end if.  end if.  end if.  compute lmat=zhigh(1:nump(1,i),j).  compute lmat2=wzhigh(1:nump(1,i),j).  do if ((csum(lmat) > 0) and (csum(lmat2) = 0)).  do if ((i < (nms+nys)) or (ydich <> 1)).  .  compute lmat2= lmat.  do if ( 0 =0).  compute lmat2 = mdiag( lmat ).  compute lmat3=make(nrow(lmat2),1,0).  loop flp=1 to ncol(lmat2).  do if (csum(lmat2(:,flp))=1).  compute lmat3={lmat3,lmat2(:,flp)}.  end if.  end loop.  compute lmat2=lmat3(:,2:ncol(lmat3)).  end if.  compute fratio = (t(t(lmat2)* b )*inv(t(lmat2)* varb *lmat2)*((t(lmat2)* b )))/ncol(lmat2).  compute pfr = 1-fcdf(fratio,ncol(lmat2),(n-nrow( b ))).  compute fresult={fratio,ncol(lmat2),(n-nrow( b )),pfr}.  do if (i = (nms+nys) and (ydich=1)).  compute fratio=fratio*ncol(lmat2).  compute pfr=1-chicdf(fratio,ncol(lmat2)).  compute fresult={fratio,ncol(lmat2),pfr}.  end if.  do if ( 1 =1).  compute lmat3=1-rsum(lmat2).  compute xfm=make(n,csum(lmat3),0).  compute flpc=1.  loop flp=1 to nrow(lmat3).  do if (lmat3(flp,1)=1).  compute xfm(:,flpc)=x(:,flp).  compute flpc=flpc+1.  end if.  end loop.  compute bfm=inv(t(xfm)*xfm)*t(xfm)*y.  compute resid=y-(xfm*bfm).  compute sstotal=(y-(csum(y)/n)).  compute sstotal=csum(sstotal&*sstotal).  compute ssresid=csum(resid&*resid).  compute rsqch= r2 -((sstotal-ssresid)/sstotal).  compute fresult={rsqch,fresult}.  release xfm,flpc, resid, ssresid, bfm.  end if  .  compute dbint=dbint+1.  end if.  do if ((ydich = 1) and (i = (nms+nys))).  .  compute btemphld=b.  compute llrdat=make(nrow(x),nrow( lmat )-csum( lmat ),-999).  compute llrdf=ncol(x)-ncol(llrdat).  compute llrcnt=0.  loop llri=1 to nrow( lmat ).  do if ( lmat (llri,1)=0).  compute llrcnt=llrcnt+1.  compute llrdat(:,llrcnt)=x(:,llri).  end if.  end loop.  .  do if ( 2 =1).  compute b = inv(t( llrdat )* llrdat )*t( llrdat )* y.  compute modres=b.  do if ( 0 =1).  compute n1=nrow( llrdat ).  compute dfres=n1-(ncol( llrdat )).  compute sstotal = t( y -(csum( y )/n1))*( y -(csum( y )/n1)).  compute resid= y - llrdat *b.  compute ssresid = csum((resid)&**2).  compute r2 = (sstotal-ssresid)/sstotal.  compute adjr2 = 1-((1-r2)*(n1-1)/(dfres)).  compute mse=ssresid/(n1-ncol( llrdat )).  .  compute n1=nrow( x ).  compute invXtX = inv(t( x )* x ).  compute varb = mse *invXtX.  compute k3 = ncol( x ).  compute xhc=0.  do if ( hc <> 5).  compute xhc= x.  compute hat = xhc(:,1).  loop i3=1 to nrow(xhc).  compute hat(i3,1)= xhc(i3,:)*invXtX*t(xhc(i3,:)).  end loop.  do if ( hc = 0 or hc =1).  loop i3 = 1 to k3.  compute xhc(:,i3)=xhc(:,i3)&* resid.  end loop.  end if.  do if ( hc =3 or hc =2).  loop i3=1 to k3.  compute xhc(:,i3) = ( resid &/(1-hat)&**(1/(4hc )))&*xhc(:,i3).  end loop.  end if.  do if ( hc = 4).  compute hcmn=make(n,2,4).  compute hcmn(:,2)=(n1*hat)/k3.  loop i3= 1 to k3.  compute xhc(:,i3) = ( resid &/(1-hat)&**(rmin(hcmn)/2))&*xhc(:,i3).  end loop.  end if.  compute varb=(invXtX*t(xhc)*xhc*invXtX).  do if ( hc =1).  compute varb=(n1/(n1-ncol( x )))&*varb.  end if.  end if.  compute hclab={'se(HC0)','se(HC1)','se(HC2)','se(HC3)','se(HC4)','se'}.  compute hclab=hclab(1,( hc +1)).  compute hcflab={'F(HC0)','F(HC1)','F(HC2)','F(HC3)','F(HC4)','F'}.  compute hcflab=hcflab(1,( hc +1)).  release xhc  .  compute seb=sqrt(diag(varb)).  compute trat = b&/seb.  compute p = 2*(1-tcdf(abs(trat), (dfres))).  compute tval = sqrt(dfres* (exp((dfres-(5/6))*((xp2/(dfres-(2/3)+(.11/dfres)))* (xp2/(dfres-(2/3)+(.11/dfres)))))-1)).  compute modres={modres,seb,trat,p}.  compute modres={modres,(b-tval&*seb),(b+tval&*seb)}.  compute modresl={'coeff',hclab,'t','p','LLCI','ULCI'}.  compute lmat = ident(ncol( llrdat )).  compute lmat = lmat(:,2:ncol(lmat)).  compute fratio = (t(t(lmat)*b)*inv(t(lmat)*varb*lmat)*((t(lmat)*b)))/(ncol( llrdat )-1).  compute pfr = 1-fcdf(fratio,(ncol( llrdat )-1),dfres).  compute modsum={sqrt(r2),r2,mse,fratio,(ncol( llrdat )-1),dfres,pfr}.  compute modsuml={'R','R-sq','MSE',hcflab,'df1','df2', 'p'}.  end if.  end if.  do if ( 2 = 2 or 2 =3).  compute xlp= llrdat.  compute ylp= y.  compute pt2 = make(nrow(ylp),1,(csum(ylp)/nrow(ylp))).  do if ( 2 =2).  compute LL3 = ylp&*ln(pt2)+(1-ylp)&*ln(1-pt2).  end if.  compute LL3 = -2*csum(LL3).  compute bt1 = make(ncol(xlp),1,0).  compute LL1 = 0.  compute pt1 = make(nrow(ylp),1,0.5).  compute pt1lp=pt1.  loop jjj = 1 to iterate.  compute vt1 = mdiag(pt1lp&*(1-pt1lp)).  compute b = bt1+inv(t(xlp)*vt1*xlp)*t(xlp)*(ylp-pt1lp).  do if ( 2 =2).  compute xlpb=xlp*b.  compute xlpbt=(xlpb > -709.7).  compute xlpb709=(1-xlpbt)*(-709.7).  compute xlpb=(xlpb&*xlpbt)+xlpb709.  compute pt1lp = 1/(1+exp(-(xlpb))).  end if.  compute itprob = csum((pt1lp < .00000001) or (pt1lp > .9999999)).  do if (itprob > 0).  loop kkk = 1 to nrow(pt1lp).  do if (pt1lp(kkk,1) > .9999999).  compute pt1lp(kkk,1) = .9999999.  end if.  do if (pt1lp(kkk,1) < .00000001).  compute pt1lp(kkk,1) = .00000001.  end if.  end loop.  compute itprob = 0.  end if.  do if (itprob = 0).  do if ( 2 =2).  compute LL = ylp&*ln(pt1lp)+(1-ylp)&*ln(1-pt1lp).  end if.  compute LL2 = -2*csum(ll).  end if.  do if (abs(LL1-LL2) < converge).  do if ( 0 =1).  compute vt1 = mdiag(pt1lp&*(1-pt1lp)).  compute varb = inv(t(xlp)*vt1*xlp).  compute seb = sqrt(diag(varb)).  end if.  break.  end if.  compute bt1 = b.  compute LL1 = LL2.  end loop.  compute modres=b.  do if (jjj > iterate).  compute itprob = 2.  do if (booting=0).  compute iterrmod=1.  end if.  do if (booting=1).  compute bootiter=1.  end if.  do if (itprobtg=0).  compute itprobtg=1.  compute errcode(errs,1) = 47.  compute errs = errs + 1.  do if (booting = 0 and 0 =1).  compute vt1 = mdiag(pt1lp&*(1-pt1lp)).  compute varb = inv(t(xlp)*vt1*xlp).  compute seb = sqrt(diag(varb)).  end if.  end if.  end if.  do if ( 0 =1).  compute trat = b&/seb.  compute dfres=nrow(xlp).  compute p = 2*(1-cdfnorm(abs(trat))).  compute modres={modres,seb,trat,p}.  compute modres={modres,(b-xp2&*seb),(b+xp2&*seb)}.  compute pvchi=1-chicdf((LL3-LL2),(nrow(modres)-1)).  compute mcF = (LL3-LL2)/LL3.  compute cox = 1-exp(-(LL3-LL2)/nrow(xlp)).  compute nagel = cox/(1-exp(-(LL3)/nrow(xlp))).  compute modsum={LL2,(LL3-LL2),(nrow(modres)-1),pvchi, mcF,cox,nagel}.  compute modsuml={'-2LL','ModelLL', 'df', 'p', 'McFadden', 'CoxSnell', 'Nagelkrk'}.  compute modresl={'coeff','se','Z','p','LLCI','ULCI'}.  end if.  end if  .  compute b=btemphld.  compute fresult={(LL2-basemod),llrdf,1-chicdf((LL2-basemod),llrdf)}  .  compute dbint=dbint+1.  end if.  compute highf={highf;fresult}.  compute highf2={highf2;fresult}.  do if (j = start).  compute flabel={flabel;'X*Z'}.  end if.  do if (j > start).  do if (nms > 1).  compute flabel={flabel;highlbz(jj,1)}.  else if (nms = 1).  compute flabel={flabel;'M*Z'}.  end if.  end if.  end if.  do if (dbint=2).  compute lmatdb=lmatdb+lmat.  do if ((ydich = 1) and (i = (nms+nys))).  .  compute btemphld=b.  compute llrdat=make(nrow(x),nrow( lmatdb )-csum( lmatdb ),-999).  compute llrdf=ncol(x)-ncol(llrdat).  compute llrcnt=0.  loop llri=1 to nrow( lmatdb ).  do if ( lmatdb (llri,1)=0).  compute llrcnt=llrcnt+1.  compute llrdat(:,llrcnt)=x(:,llri).  end if.  end loop.  .  do if ( 2 =1).  compute b = inv(t( llrdat )* llrdat )*t( llrdat )* y.  compute modres=b.  do if ( 0 =1).  compute n1=nrow( llrdat ).  compute dfres=n1-(ncol( llrdat )).  compute sstotal = t( y -(csum( y )/n1))*( y -(csum( y )/n1)).  compute resid= y - llrdat *b.  compute ssresid = csum((resid)&**2).  compute r2 = (sstotal-ssresid)/sstotal.  compute adjr2 = 1-((1-r2)*(n1-1)/(dfres)).  compute mse=ssresid/(n1-ncol( llrdat )).  .  compute n1=nrow( x ).  compute invXtX = inv(t( x )* x ).  compute varb = mse *invXtX.  compute k3 = ncol( x ).  compute xhc=0.  do if ( hc <> 5).  compute xhc= x.  compute hat = xhc(:,1).  loop i3=1 to nrow(xhc).  compute hat(i3,1)= xhc(i3,:)*invXtX*t(xhc(i3,:)).  end loop.  do if ( hc = 0 or hc =1).  loop i3 = 1 to k3.  compute xhc(:,i3)=xhc(:,i3)&* resid.  end loop.  end if.  do if ( hc =3 or hc =2).  loop i3=1 to k3.  compute xhc(:,i3) = ( resid &/(1-hat)&**(1/(4hc )))&*xhc(:,i3).  end loop.  end if.  do if ( hc = 4).  compute hcmn=make(n,2,4).  compute hcmn(:,2)=(n1*hat)/k3.  loop i3= 1 to k3.  compute xhc(:,i3) = ( resid &/(1-hat)&**(rmin(hcmn)/2))&*xhc(:,i3).  end loop.  end if.  compute varb=(invXtX*t(xhc)*xhc*invXtX).  do if ( hc =1).  compute varb=(n1/(n1-ncol( x )))&*varb.  end if.  end if.  compute hclab={'se(HC0)','se(HC1)','se(HC2)','se(HC3)','se(HC4)','se'}.  compute hclab=hclab(1,( hc +1)).  compute hcflab={'F(HC0)','F(HC1)','F(HC2)','F(HC3)','F(HC4)','F'}.  compute hcflab=hcflab(1,( hc +1)).  release xhc  .  compute seb=sqrt(diag(varb)).  compute trat = b&/seb.  compute p = 2*(1-tcdf(abs(trat), (dfres))).  compute tval = sqrt(dfres* (exp((dfres-(5/6))*((xp2/(dfres-(2/3)+(.11/dfres)))* (xp2/(dfres-(2/3)+(.11/dfres)))))-1)).  compute modres={modres,seb,trat,p}.  compute modres={modres,(b-tval&*seb),(b+tval&*seb)}.  compute modresl={'coeff',hclab,'t','p','LLCI','ULCI'}.  compute lmat = ident(ncol( llrdat )).  compute lmat = lmat(:,2:ncol(lmat)).  compute fratio = (t(t(lmat)*b)*inv(t(lmat)*varb*lmat)*((t(lmat)*b)))/(ncol( llrdat )-1).  compute pfr = 1-fcdf(fratio,(ncol( llrdat )-1),dfres).  compute modsum={sqrt(r2),r2,mse,fratio,(ncol( llrdat )-1),dfres,pfr}.  compute modsuml={'R','R-sq','MSE',hcflab,'df1','df2', 'p'}.  end if.  end if.  do if ( 2 = 2 or 2 =3).  compute xlp= llrdat.  compute ylp= y.  compute pt2 = make(nrow(ylp),1,(csum(ylp)/nrow(ylp))).  do if ( 2 =2).  compute LL3 = ylp&*ln(pt2)+(1-ylp)&*ln(1-pt2).  end if.  compute LL3 = -2*csum(LL3).  compute bt1 = make(ncol(xlp),1,0).  compute LL1 = 0.  compute pt1 = make(nrow(ylp),1,0.5).  compute pt1lp=pt1.  loop jjj = 1 to iterate.  compute vt1 = mdiag(pt1lp&*(1-pt1lp)).  compute b = bt1+inv(t(xlp)*vt1*xlp)*t(xlp)*(ylp-pt1lp).  do if ( 2 =2).  compute xlpb=xlp*b.  compute xlpbt=(xlpb > -709.7).  compute xlpb709=(1-xlpbt)*(-709.7).  compute xlpb=(xlpb&*xlpbt)+xlpb709.  compute pt1lp = 1/(1+exp(-(xlpb))).  end if.  compute itprob = csum((pt1lp < .00000001) or (pt1lp > .9999999)).  do if (itprob > 0).  loop kkk = 1 to nrow(pt1lp).  do if (pt1lp(kkk,1) > .9999999).  compute pt1lp(kkk,1) = .9999999.  end if.  do if (pt1lp(kkk,1) < .00000001).  compute pt1lp(kkk,1) = .00000001.  end if.  end loop.  compute itprob = 0.  end if.  do if (itprob = 0).  do if ( 2 =2).  compute LL = ylp&*ln(pt1lp)+(1-ylp)&*ln(1-pt1lp).  end if.  compute LL2 = -2*csum(ll).  end if.  do if (abs(LL1-LL2) < converge).  do if ( 0 =1).  compute vt1 = mdiag(pt1lp&*(1-pt1lp)).  compute varb = inv(t(xlp)*vt1*xlp).  compute seb = sqrt(diag(varb)).  end if.  break.  end if.  compute bt1 = b.  compute LL1 = LL2.  end loop.  compute modres=b.  do if (jjj > iterate).  compute itprob = 2.  do if (booting=0).  compute iterrmod=1.  end if.  do if (booting=1).  compute bootiter=1.  end if.  do if (itprobtg=0).  compute itprobtg=1.  compute errcode(errs,1) = 47.  compute errs = errs + 1.  do if (booting = 0 and 0 =1).  compute vt1 = mdiag(pt1lp&*(1-pt1lp)).  compute varb = inv(t(xlp)*vt1*xlp).  compute seb = sqrt(diag(varb)).  end if.  end if.  end if.  do if ( 0 =1).  compute trat = b&/seb.  compute dfres=nrow(xlp).  compute p = 2*(1-cdfnorm(abs(trat))).  compute modres={modres,seb,trat,p}.  compute modres={modres,(b-xp2&*seb),(b+xp2&*seb)}.  compute pvchi=1-chicdf((LL3-LL2),(nrow(modres)-1)).  compute mcF = (LL3-LL2)/LL3.  compute cox = 1-exp(-(LL3-LL2)/nrow(xlp)).  compute nagel = cox/(1-exp(-(LL3)/nrow(xlp))).  compute modsum={LL2,(LL3-LL2),(nrow(modres)-1),pvchi, mcF,cox,nagel}.  compute modsuml={'-2LL','ModelLL', 'df', 'p', 'McFadden', 'CoxSnell', 'Nagelkrk'}.  compute modresl={'coeff','se','Z','p','LLCI','ULCI'}.  end if.  end if  .  compute b=btemphld.  compute fresult={(LL2-basemod),llrdf,1-chicdf((LL2-basemod),llrdf)}  .  end if.  do if (ydich <> 1 or i < (nms+nys)).  .  compute lmat2= lmatdb.  do if ( 0 =0).  compute lmat2 = mdiag( lmatdb ).  compute lmat3=make(nrow(lmat2),1,0).  loop flp=1 to ncol(lmat2).  do if (csum(lmat2(:,flp))=1).  compute lmat3={lmat3,lmat2(:,flp)}.  end if.  end loop.  compute lmat2=lmat3(:,2:ncol(lmat3)).  end if.  compute fratio = (t(t(lmat2)* b )*inv(t(lmat2)* varb *lmat2)*((t(lmat2)* b )))/ncol(lmat2).  compute pfr = 1-fcdf(fratio,ncol(lmat2),(n-nrow( b ))).  compute fresult={fratio,ncol(lmat2),(n-nrow( b )),pfr}.  do if (i = (nms+nys) and (ydich=1)).  compute fratio=fratio*ncol(lmat2).  compute pfr=1-chicdf(fratio,ncol(lmat2)).  compute fresult={fratio,ncol(lmat2),pfr}.  end if.  do if ( 1 =1).  compute lmat3=1-rsum(lmat2).  compute xfm=make(n,csum(lmat3),0).  compute flpc=1.  loop flp=1 to nrow(lmat3).  do if (lmat3(flp,1)=1).  compute xfm(:,flpc)=x(:,flp).  compute flpc=flpc+1.  end if.  end loop.  compute bfm=inv(t(xfm)*xfm)*t(xfm)*y.  compute resid=y-(xfm*bfm).  compute sstotal=(y-(csum(y)/n)).  compute sstotal=csum(sstotal&*sstotal).  compute ssresid=csum(resid&*resid).  compute rsqch= r2 -((sstotal-ssresid)/sstotal).  compute fresult={rsqch,fresult}.  release xfm,flpc, resid, ssresid, bfm.  end if  .  end if.  compute dbint=0.  compute highf={highf;fresult}.  do if (jj=0 and nms > 0).  compute flabel={flabel;'BOTH(X)'}.  end if.  do if (jj=0 and nms = 0).  compute flabel={flabel;'BOTH'}.  end if.  do if (jj>0 and nms = 1).  compute flabel={flabel;'BOTH(M)'}.  end if.  do if (nms > 1 and jj > 0).  compute flabel={flabel;highlbbt(jj,1)}.  end if.  end if.  compute lmat2=wzhigh(1:nump(1,i),j).  do if (csum(lmat2) > 0).  do if ((i < (nms+nys)) or (ydich <> 1)).  .  compute lmat2= lmat2.  do if ( 0 =0).  compute lmat2 = mdiag( lmat2 ).  compute lmat3=make(nrow(lmat2),1,0).  loop flp=1 to ncol(lmat2).  do if (csum(lmat2(:,flp))=1).  compute lmat3={lmat3,lmat2(:,flp)}.  end if.  end loop.  compute lmat2=lmat3(:,2:ncol(lmat3)).  end if.  compute fratio = (t(t(lmat2)* b )*inv(t(lmat2)* varb *lmat2)*((t(lmat2)* b )))/ncol(lmat2).  compute pfr = 1-fcdf(fratio,ncol(lmat2),(n-nrow( b ))).  compute fresult={fratio,ncol(lmat2),(n-nrow( b )),pfr}.  do if (i = (nms+nys) and (ydich=1)).  compute fratio=fratio*ncol(lmat2).  compute pfr=1-chicdf(fratio,ncol(lmat2)).  compute fresult={fratio,ncol(lmat2),pfr}.  end if.  do if ( 1 =1).  compute lmat3=1-rsum(lmat2).  compute xfm=make(n,csum(lmat3),0).  compute flpc=1.  loop flp=1 to nrow(lmat3).  do if (lmat3(flp,1)=1).  compute xfm(:,flpc)=x(:,flp).  compute flpc=flpc+1.  end if.  end loop.  compute bfm=inv(t(xfm)*xfm)*t(xfm)*y.  compute resid=y-(xfm*bfm).  compute sstotal=(y-(csum(y)/n)).  compute sstotal=csum(sstotal&*sstotal).  compute ssresid=csum(resid&*resid).  compute rsqch= r2 -((sstotal-ssresid)/sstotal).  compute fresult={rsqch,fresult}.  release xfm,flpc, resid, ssresid, bfm.  end if  .  end if.  do if ((ydich = 1) and (i = (nms+nys))).  .  compute btemphld=b.  compute llrdat=make(nrow(x),nrow( lmat2 )-csum( lmat2 ),-999).  compute llrdf=ncol(x)-ncol(llrdat).  compute llrcnt=0.  loop llri=1 to nrow( lmat2 ).  do if ( lmat2 (llri,1)=0).  compute llrcnt=llrcnt+1.  compute llrdat(:,llrcnt)=x(:,llri).  end if.  end loop.  .  do if ( 2 =1).  compute b = inv(t( llrdat )* llrdat )*t( llrdat )* y.  compute modres=b.  do if ( 0 =1).  compute n1=nrow( llrdat ).  compute dfres=n1-(ncol( llrdat )).  compute sstotal = t( y -(csum( y )/n1))*( y -(csum( y )/n1)).  compute resid= y - llrdat *b.  compute ssresid = csum((resid)&**2).  compute r2 = (sstotal-ssresid)/sstotal.  compute adjr2 = 1-((1-r2)*(n1-1)/(dfres)).  compute mse=ssresid/(n1-ncol( llrdat )).  .  compute n1=nrow( x ).  compute invXtX = inv(t( x )* x ).  compute varb = mse *invXtX.  compute k3 = ncol( x ).  compute xhc=0.  do if ( hc <> 5).  compute xhc= x.  compute hat = xhc(:,1).  loop i3=1 to nrow(xhc).  compute hat(i3,1)= xhc(i3,:)*invXtX*t(xhc(i3,:)).  end loop.  do if ( hc = 0 or hc =1).  loop i3 = 1 to k3.  compute xhc(:,i3)=xhc(:,i3)&* resid.  end loop.  end if.  do if ( hc =3 or hc =2).  loop i3=1 to k3.  compute xhc(:,i3) = ( resid &/(1-hat)&**(1/(4hc )))&*xhc(:,i3).  end loop.  end if.  do if ( hc = 4).  compute hcmn=make(n,2,4).  compute hcmn(:,2)=(n1*hat)/k3.  loop i3= 1 to k3.  compute xhc(:,i3) = ( resid &/(1-hat)&**(rmin(hcmn)/2))&*xhc(:,i3).  end loop.  end if.  compute varb=(invXtX*t(xhc)*xhc*invXtX).  do if ( hc =1).  compute varb=(n1/(n1-ncol( x )))&*varb.  end if.  end if.  compute hclab={'se(HC0)','se(HC1)','se(HC2)','se(HC3)','se(HC4)','se'}.  compute hclab=hclab(1,( hc +1)).  compute hcflab={'F(HC0)','F(HC1)','F(HC2)','F(HC3)','F(HC4)','F'}.  compute hcflab=hcflab(1,( hc +1)).  release xhc  .  compute seb=sqrt(diag(varb)).  compute trat = b&/seb.  compute p = 2*(1-tcdf(abs(trat), (dfres))).  compute tval = sqrt(dfres* (exp((dfres-(5/6))*((xp2/(dfres-(2/3)+(.11/dfres)))* (xp2/(dfres-(2/3)+(.11/dfres)))))-1)).  compute modres={modres,seb,trat,p}.  compute modres={modres,(b-tval&*seb),(b+tval&*seb)}.  compute modresl={'coeff',hclab,'t','p','LLCI','ULCI'}.  compute lmat = ident(ncol( llrdat )).  compute lmat = lmat(:,2:ncol(lmat)).  compute fratio = (t(t(lmat)*b)*inv(t(lmat)*varb*lmat)*((t(lmat)*b)))/(ncol( llrdat )-1).  compute pfr = 1-fcdf(fratio,(ncol( llrdat )-1),dfres).  compute modsum={sqrt(r2),r2,mse,fratio,(ncol( llrdat )-1),dfres,pfr}.  compute modsuml={'R','R-sq','MSE',hcflab,'df1','df2', 'p'}.  end if.  end if.  do if ( 2 = 2 or 2 =3).  compute xlp= llrdat.  compute ylp= y.  compute pt2 = make(nrow(ylp),1,(csum(ylp)/nrow(ylp))).  do if ( 2 =2).  compute LL3 = ylp&*ln(pt2)+(1-ylp)&*ln(1-pt2).  end if.  compute LL3 = -2*csum(LL3).  compute bt1 = make(ncol(xlp),1,0).  compute LL1 = 0.  compute pt1 = make(nrow(ylp),1,0.5).  compute pt1lp=pt1.  loop jjj = 1 to iterate.  compute vt1 = mdiag(pt1lp&*(1-pt1lp)).  compute b = bt1+inv(t(xlp)*vt1*xlp)*t(xlp)*(ylp-pt1lp).  do if ( 2 =2).  compute xlpb=xlp*b.  compute xlpbt=(xlpb > -709.7).  compute xlpb709=(1-xlpbt)*(-709.7).  compute xlpb=(xlpb&*xlpbt)+xlpb709.  compute pt1lp = 1/(1+exp(-(xlpb))).  end if.  compute itprob = csum((pt1lp < .00000001) or (pt1lp > .9999999)).  do if (itprob > 0).  loop kkk = 1 to nrow(pt1lp).  do if (pt1lp(kkk,1) > .9999999).  compute pt1lp(kkk,1) = .9999999.  end if.  do if (pt1lp(kkk,1) < .00000001).  compute pt1lp(kkk,1) = .00000001.  end if.  end loop.  compute itprob = 0.  end if.  do if (itprob = 0).  do if ( 2 =2).  compute LL = ylp&*ln(pt1lp)+(1-ylp)&*ln(1-pt1lp).  end if.  compute LL2 = -2*csum(ll).  end if.  do if (abs(LL1-LL2) < converge).  do if ( 0 =1).  compute vt1 = mdiag(pt1lp&*(1-pt1lp)).  compute varb = inv(t(xlp)*vt1*xlp).  compute seb = sqrt(diag(varb)).  end if.  break.  end if.  compute bt1 = b.  compute LL1 = LL2.  end loop.  compute modres=b.  do if (jjj > iterate).  compute itprob = 2.  do if (booting=0).  compute iterrmod=1.  end if.  do if (booting=1).  compute bootiter=1.  end if.  do if (itprobtg=0).  compute itprobtg=1.  compute errcode(errs,1) = 47.  compute errs = errs + 1.  do if (booting = 0 and 0 =1).  compute vt1 = mdiag(pt1lp&*(1-pt1lp)).  compute varb = inv(t(xlp)*vt1*xlp).  compute seb = sqrt(diag(varb)).  end if.  end if.  end if.  do if ( 0 =1).  compute trat = b&/seb.  compute dfres=nrow(xlp).  compute p = 2*(1-cdfnorm(abs(trat))).  compute modres={modres,seb,trat,p}.  compute modres={modres,(b-xp2&*seb),(b+xp2&*seb)}.  compute pvchi=1-chicdf((LL3-LL2),(nrow(modres)-1)).  compute mcF = (LL3-LL2)/LL3.  compute cox = 1-exp(-(LL3-LL2)/nrow(xlp)).  compute nagel = cox/(1-exp(-(LL3)/nrow(xlp))).  compute modsum={LL2,(LL3-LL2),(nrow(modres)-1),pvchi, mcF,cox,nagel}.  compute modsuml={'-2LL','ModelLL', 'df', 'p', 'McFadden', 'CoxSnell', 'Nagelkrk'}.  compute modresl={'coeff','se','Z','p','LLCI','ULCI'}.  end if.  end if  .  compute b=btemphld.  compute fresult={(LL2-basemod),llrdf,1-chicdf((LL2-basemod),llrdf)}  .  end if.  compute highf={highf;fresult}.  compute highf2={highf2;fresult}.  do if (j = start).  compute flabel={flabel;'X*W*Z'}.  end if.  do if (j > start).  do if (nms > 1).  compute flabel={flabel;highlbwz(jj,1)}.  else if (nms = 1).  compute flabel={flabel;'M*W*Z'}.  end if.  end if.  end if.  compute jj=jj+1.  end loop.  release jj.  compute start=start+i.  end if.  do if (nrow(highf) > 1).  compute highf=highf(2:nrow(highf),:).  compute highf2=highf2(2:nrow(highf2),:).  compute flabel=flabel(2:nrow(flabel),1).  do if ((i < nms+nys) or (ydich=0)).  compute clabtmp={'R2-chng', hcflab,'df1','df2','p'}.  print highf/format = F10.4 /rnames=flabel/cnames=clabtmp/ title = 'Test(s) of highest order unconditional interaction(s):'.  end if.  do if (ydich=1 and i=(nms+nys)).  compute clabtmp={'Chi-sq', 'df','p'}.  print/title='Likelihood ratio test(s) of highest order'.  print highf/format = F10.4 /rnames=flabel/cnames=clabtmp/ title = 'unconditional interactions(s):'/space=0.  end if.  compute intpb=highf2(:,ncol(highf2)).  end if.  compute intstart=intstart+numint(1,i).  end if.  .  do if (criterr=0).  compute threeway=0.  compute didprint=0.  compute didsome=0.  compute sigintct=0.  loop jmed =1 to (nms+1).  compute hasw=0.  compute hasz=0.  compute jnok=0.  compute nm1vls=0.  compute nm2vls=0.  compute panelgrp=0.  compute graphixs={'WITH', outnames(1,i), 'BY'}.  compute focpred4={' '}.  compute intprint=0.  compute modcat=0.  do if (jmed <= i).  do if ((jmed = 1) and ((i+1) = nrow(bcmat))).  compute pathscnt=pathscnt+1.  else.  compute paths={paths,bcmat((i+1),jmed)}.  compute pathsw={pathsw,wcmat((i+1),jmed)}.  compute pathsz={pathsz,zcmat((i+1),jmed)}.  compute pathswz={pathswz,wzcmat((i+1),jmed)}.  compute temp=fochigh(:,pathscnt)&*bootloc(:,i).  compute pathsfoc={pathsfoc,pathsfoc(:,1)}.  do if (jmed=1).  compute pathtype={pathtype,1}.  end if.  do if ((i+1)=nrow(bcmat)).  compute pathtype={pathtype,3}.  end if.  do if (jmed > 1) and ((i+1) < nrow(bcmat)).  compute pathtype={pathtype,2}.  end if.  do if (jmed=1 and nxvls > 1 and (bcmat((i+1),jmed)=1)).  compute pathsfoc(:,(pathscn2+1))=temp(2:(nxvls+1),1).  end if.  do if ((jmed > 1) or (jmed=1 and nxvls=1)).  compute temp=cmax(temp).  compute pathsfoc(1,(pathscn2+1))=temp.  end if.  compute pathscnt=pathscnt+1.  compute pathscn2=pathscn2+1.  do if (i <= nms).  compute pathsdv={pathsdv,mnames(1,i)}.  end if.  do if (i > nms).  compute pathsdv={pathsdv,ynames}.  end if.  end if.  compute coeffcol=coeffcol+1.  compute probettt=coeffs(1:nrow(b),coeffcol).  do if (jmed=1 and (bcmat((i+1),jmed)=1)).  compute omni=make(nrow(probettt),nxvls,0).  compute omnitmp=ident(nxvls).  compute omni(2:(1+nxvls),:)=omnitmp.  end if.  do if (csum(probettt)>0).  compute probvarb=make(csum(probettt),csum(probettt),999).  compute probcoef=make(csum(probettt),1,999).  compute coefflp2=1.  loop coefflp=1 to nrow(probettt).  do if (probettt(coefflp,1)=1).  compute probcoef(coefflp2,1)=b(coefflp,1).  compute coefflp2=coefflp2+1.  end if.  end loop.  compute coefflp=0.  compute coefflp2=0.  loop iclp=1 to nrow(probettt).  do if probettt(iclp,1)=1.  compute coefflp=coefflp+1.  compute coefflp2=coefflp.  compute probvarb(coefflp,coefflp) = varb(iclp,iclp).  do if (iclp < nrow(probettt)).  loop jclp=(iclp+1) to nrow(probettt).  do if (probettt(jclp,1)=1).  compute coefflp2=coefflp2+1.  compute probvarb(coefflp,coefflp2)=varb(iclp, jclp).  compute probvarb(coefflp2,coefflp)=varb(iclp, jclp).  end if.  end loop.  end if.  end if.  end loop.  end if.  end if.  compute xprobval=xmodvals.  do if (nxvls > 1 or mcx > 0).  compute xprobval=dummatx(:,2:ncol(dummatx)).  end if.  do if ((wcmat((i+1),jmed)=1) and (zcmat((i+1),jmed)=0)).  compute numplps=1.  compute modvals=wmodvals.  compute probeval=wmodvals.  compute wheremv1=wherexw.  compute nm1vls=nwvls.  compute lpstsp={1,1}.  compute modcat=0.  compute jnmod=wtmp.  compute jnmodlab=wnames.  compute jnok=1.  compute jnmin=wmin.  compute jnmax=wmax.  compute wherejn1=2.  do if (jmed=1).  compute wherejn3=wherexw(1,i).  do if (nxvls > 1).  compute jnok=0.  end if.  end if.  do if (jmed > 1).  compute wherejn1=wherem((jmed-1),i).  compute wherejn3=wheremw(((2*jmed)-3),i).  end if.  do if (nwvls > 1).  compute probeval=wprobval.  compute lpstsp(1,2)=ncol(probeval).  compute modcat=1.  compute jnok=0.  end if.  do if (wdich = 1).  compute modcat=1.  compute jnok=0.  end if.  compute problabs=wnames.  compute focpred3={wnames,'(W)'}.  compute hasw=1.  compute modgrph=wnames.  compute intprint=1.  compute sigintct=sigintct+1.  compute printpbe=intpb(sigintct,1).  end if.  do if ((wcmat((i+1),jmed)=0) and (zcmat((i+1),jmed)=1)).  compute numplps=1.  compute modvals=zmodvals.  compute probeval=zmodvals.  compute wheremv1=wherexz.  compute nm1vls=nzvls.  compute lpstsp={1,1}.  compute jnok=1.  compute jnmod=ztmp.  compute jnmin=zmin.  compute jnmax=zmax.  compute jnmodlab=znames.  compute wherejn1=2.  do if (jmed=1).  compute wherejn3=wherexz(1,i).  do if (nxvls > 1).  compute jnok=0.  end if.  end if.  do if (jmed > 1).  compute wherejn1=wherem((jmed-1),i).  compute wherejn3=wheremz(((2*jmed)-3),i).  end if.  do if (nzvls > 1).  compute probeval=zprobval.  compute lpstsp(1,2)=ncol(probeval).  compute modcat=1.  compute jnok=0.  end if.  do if (zdich = 1).  compute modcat=1.  compute jnok=0.  end if.  compute problabs=znames.  compute focpred3={znames,'(Z)'}.  compute modgrph=znames.  compute hasz=1.  compute intprint=1.  compute sigintct=sigintct+1.  compute printpbe=intpb(sigintct,1).  end if.  do if ((wzcmat((i+1),jmed)=1) or ((wcmat((i+1),jmed)=1) and (zcmat((i+1),jmed)=1))).  compute numplps=2.  compute probecnt=1.  compute intprint=1.  do if (wzcmat((i+1),jmed)=1).  compute sigintct=sigintct+1.  compute printpbe=intpb(sigintct,1).  else.  compute sigintct=sigintct+2.  compute printpbe=cmin(intpb((sigintct-1):sigintct,1)).  end if.  compute panelgrp=1.  compute hasw=1.  compute hasz=1.  compute panelcde={'/PANEL','ROWVAR=',znames,'.'}.  compute modgrph=wnames.  compute lpstsp={1,1;1,1}.  compute wheremv1=wherexw.  compute nm1vls=nwvls.  compute wheremv2=wherexz.  compute nm2vls=nzvls.  compute jnok=0.  do if (wzcmat((i+1),jmed)=1).  compute jnok=1.  end if.  do if (jmed > 1).  compute mprobval=mmodvals.  end if.  do if (jmed=1).  do if (nxvls > 1).  compute jnok=0.  end if.  end if.  do if (nwvls > 1).  compute lpstsp(1,2)=ncol(wprobval).  compute modcat=1.  compute jnok=0.  end if.  do if (zdich=1).  compute modcat=1.  compute jnok=0.  end if.  compute lpstsp(2,1)=lpstsp(1,2)+1.  compute lpstsp(2,2)=lpstsp(1,2)+1.  do if (nzvls > 1).  compute lpstsp(2,1)=lpstsp(1,2)+1.  compute lpstsp(2,2)=lpstsp(1,2)+ncol(zprobval).  compute jnok=0.  end if.  do if (zdich=1).  compute jnok=0.  end if.  compute omni3=make(nrow(b),(nxvls*nwvls),0).  do if (jmed > 1).  compute omni3=make(nrow(b),nwvls,0).  end if.  compute focpred3={wnames,'(W)'}.  compute focpred4={' ', 'Mod var:', znames, '(Z)'}.  compute modvals=make((nrow(wmodvals)*nrow(zmodvals)),2,0).  compute probeval=make((nrow(wmodvals)*nrow(zmodvals)),(ncol(wprobval)+ncol(zprobval)),0).  loop probei= 1 to nrow(wmodvals).  loop probej =1 to nrow(zmodvals).  compute modvals(probecnt,1)=wmodvals(probei,1).  compute probeval(probecnt,1:nwvls)=wprobval(probei,:).  compute modvals(probecnt,2)=zmodvals(probej,1).  compute probeval(probecnt,(nwvls+1):(nwvls+nzvls))=zprobval(probej,:).  compute probecnt=probecnt+1.  end loop.  end loop.  do if (wzcmat((i+1),jmed)=1).  compute numplps=numplps+1.  compute probprod=make(1,(ncol(wprobval)*ncol(zprobval)),0).  compute lpstsp2={1,1}.  compute lpstsp={lpstsp;lpstsp2}.  compute lpstsp(3,1)=lpstsp(2,2)+1.  compute lpstsp(3,2)=lpstsp(2,2)+ncol(probprod).  compute jnmod=ztmp.  compute jnmin=zmin.  compute jnmax=zmax.  compute jnmodlab=znames.  do if (jmed = 1).  compute wherejn1=wherexw(1,i).  compute wherejn3=wherexwz(1,i).  end if.  do if (jmed > 1).  compute wherejn1=wheremw(((2*jmed)-3),i).  compute wherejn3=wheremwz(((2*jmed)-3),i).  end if.  loop probei = 1 to nrow(wmodvals).  loop probej = 1 to nrow(zmodvals).  compute probtemp=1.  loop probek = 1 to ncol(wprobval).  compute probtemp={probtemp,(wprobval(probei,probek)&*zprobval(probej,:))}.  end loop.  compute probprod={probprod;probtemp(1,2:ncol(probtemp))}.  end loop.  end loop.  compute probprod=probprod(2:nrow(probprod),:).  compute probeval={probeval,probprod}.  end if.  compute problabs={wnames,znames}.  release probecnt, probei, probej.  end if.  do if (intprint=1).  compute focpred={' Focal', 'predict:'}.  do if (jmed=1).  compute focpred={focpred,xnames,'(X)'}.  compute focplotv=xmodvals.  end if.  do if (jmed >1).  do if (nms > 1).  compute focpred={focpred,mnames(1,(jmed-1)), medlb2(1,(jmed-1))}.  end if.  do if (nms = 1).  compute focpred={focpred,mnames(1,(jmed-1)), '(M)'}.  end if.  compute focplotv=mmodvals(:,(jmed-1)).  end if.  compute focpred2={' ', 'Mod var:',focpred3}.  compute focpred={focpred;focpred2}.  do if (ncol(focpred4) > 1).  compute focpred={focpred;focpred4}.  compute focpred4={' '}.  end if.  release focpred2,focpred3.  do if ((plot = 1 or plot = 2) or (printpbe <= intprobe)).  print focpred/title='----------'/format=A8/space=0.  end if.  compute foctmp=make(nrow(modvals),1,1).  compute probexpl=1.  compute probeva2={foctmp,probeval}.  do if (jmed=1 and nxs > 0 and mcx > 0).  compute probexpl=nxvls.  end if.  compute foctmp=make(nrow(modvals),1,1).  compute modvals3=make(1,(6+ncol(problabs)),0).  compute probrown=make(nrow(probeval),1,0).  compute jtmp=1.  loop probei = 1 to nrow(probeval).  compute probrown(probei,1)=jtmp.  compute jtmp=jtmp+nxvls.  end loop.  release jtmp.  compute probrow=999.  compute modvarl=problabs.  do if (plot = 1 or plot = 2 or nxvls > 1).  compute plotvals=make((nrow(modvals)*nrow(focplotv)),(ncol(modvals)+1),999).  loop ploti=1 to nrow(modvals).  loop plotj=1 to nrow(focplotv).  compute plotvals((((ploti-1)*nrow(focplotv))+plotj),2:ncol(plotvals))=modvals(ploti,:).  compute plotvals((((ploti-1)*nrow(focplotv))+plotj),1)=focplotv(plotj,1).  end loop.  end loop.  compute focpredn=3.  do if (jmed=1).  do if (nxvls > 1).  compute focpredn=(nxvls+1).  end if.  do if (nxvls=1 and xdich=1).  compute focpredn=2.  end if.  end if.  compute meanmat=mdiag(means).  compute onesmat=make(nrow(meanmat),(nrow(probeval)*focpredn),1).  compute probeplt=t(mdiag(means)*onesmat).  do if (jmed=1).  do if ((wcmat((i+1),1)=1) or (zcmat((i+1),1)=1)).  compute plotcnt=1.  compute iloops=nwpval*nzpval.  compute plotmx=nxpval*nzpval.  do if ((wcmat((i+1),1)=1) and (zcmat((i+1),1)=0)).  compute iloops=nwpval.  compute plotmx=nxpval.  end if.  do if ((wcmat((i+1),1)=0) and (zcmat((i+1),1)=1)).  compute iloops=nzpval.  compute plotmx=nxpval.  end if.  compute xestvals=make((nxpval*iloops),ncol(xprobval),-999).  do if (wcmat((i+1),1)=1).  compute westvals=make(nrow(xestvals),ncol(wprobval),-999).  end if.  do if (zcmat((i+1),1)=1).  compute zestvals=make(nrow(xestvals),ncol(zprobval),-999).  end if.  loop ploti=1 to iloops.  loop plotj=1 to nxpval.  compute xestvals(plotcnt,:)=xprobval(plotj,:).  compute plotcnt=plotcnt+1.  end loop.  end loop.  compute plotcnt=1.  compute plotcnt1=1.  compute plotcnt2=1.  compute plotcntz=1.  loop ploti = 1 to (iloops*nxpval).  do if (wcmat((i+1),1)=1).  compute westvals(ploti,:)=wprobval(plotcnt1,:).  end if.  do if ((wcmat((i+1),1)=0) and (zcmat((i+1),1)=1)).  compute zestvals(ploti,:)=zprobval(plotcnt1,:).  end if.  do if ((wcmat((i+1),1)=1) and (zcmat((i+1),1)=1)).  compute zestvals(ploti,:)=zprobval(plotcnt2,:).  compute plotcntz=plotcntz+1.  end if.  compute plotcnt=plotcnt+1.  do if (plotcnt > plotmx).  compute plotcnt=1.  compute plotcnt1=plotcnt1+1.  end if.  do if (plotcntz > nxpval).  compute plotcnt2=plotcnt2+1.  compute plotcntz=1.  do if (plotcnt2 > nzpval).  compute plotcnt2=1.  end if.  end if.  end loop.  compute probeplt(:,2:(1+(ncol(xestvals))))=xestvals.  do if (wcmat((i+1),1)=1).  compute probeplt(:,(wherew(1,i)):(wherew(2,i)))=westvals.  end if.  do if (zcmat((i+1),1)=1).  compute probeplt(:,(wherez(1,i)):(wherez(2,i)))=zestvals.  end if.  end if.  end if.  do if (jmed > 1).  do if ((wcmat((i+1),jmed)=1) or (zcmat((i+1),jmed)=1)).  compute plotcnt=1.  compute iloops=nwpval*nzpval.  compute plotmx=3*nzpval.  do if ((wcmat((i+1),jmed)=1) and (zcmat((i+1),jmed)=0)).  compute iloops=nwpval.  compute plotmx=3.  end if.  do if ((wcmat((i+1),jmed)=0) and (zcmat((i+1),jmed)=1)).  compute iloops=nzpval.  compute plotmx=3.  end if.  compute mestvals=make((3*iloops),1,-999).  do if (wcmat((i+1),jmed)=1).  compute westvals=make(nrow(mestvals),ncol(wprobval),-999).  end if.  do if (zcmat((i+1),jmed)=1).  compute zestvals=make(nrow(mestvals),ncol(zprobval),-999).  end if.  loop ploti=1 to iloops.  loop plotj=1 to 3.  compute mestvals(plotcnt,:)=mprobval(plotj,(jmed-1)).  compute plotcnt=plotcnt+1.  end loop.  end loop.  compute plotcnt=1.  compute plotcnt1=1.  compute plotcnt2=1.  compute plotcntz=1.  loop ploti = 1 to (iloops*3).  do if (wcmat((i+1),jmed)=1).  compute westvals(ploti,:)=wprobval(plotcnt1,:).  end if.  do if ((wcmat((i+1),jmed)=0) and (zcmat((i+1),jmed)=1)).  compute zestvals(ploti,:)=zprobval(plotcnt1,:).  end if.  do if ((wcmat((i+1),jmed)=1) and (zcmat((i+1),jmed)=1)).  compute zestvals(ploti,:)=zprobval(plotcnt2,:).  compute plotcntz=plotcntz+1.  end if.  compute plotcnt=plotcnt+1.  do if (plotcnt > plotmx).  compute plotcnt=1.  compute plotcnt1=plotcnt1+1.  end if.  do if (plotcntz > 3).  compute plotcnt2=plotcnt2+1.  compute plotcntz=1.  do if (plotcnt2 > nzpval).  compute plotcnt2=1.  end if.  end if.  end loop.  compute probeplt(:,wherem((jmed-1),i))=mestvals.  do if (wcmat((i+1),jmed)=1).  do if (model <> 74).  compute probeplt(:,(wherew(1,i)):(wherew(2,i)))=westvals.  end if.  do if (model = 74).  compute probeplt(:,(wherex(1,i)):(wherex(2,i)))=westvals.  end if.  end if.  do if (zcmat((i+1),jmed)=1).  compute probeplt(:,(wherez(1,i)):(wherez(2,i)))=zestvals.  end if.  end if.  end if.  compute prodloop = 1.  do if (jmed=1).  compute prodloop=ncol(xestvals).  end if.  do if (wcmat((i+1),jmed))=1.  compute plotcnt=0.  loop ploti = 1 to prodloop.  loop plotj = 1 to ncol(westvals).  do if (jmed=1).  compute probeplt(:,(wherexw(1,i)+plotcnt))=xestvals(:,ploti)&*westvals(:,plotj).  end if.  do if (jmed > 1).  compute probeplt(:,(wheremw(((jmed*2)-3) ,i)+plotcnt))=mestvals(:,ploti)&*westvals(:,plotj).  end if.  compute plotcnt=plotcnt+1.  end loop.  end loop.  end if.  do if (zcmat((i+1),jmed))=1.  compute plotcnt=0.  loop ploti = 1 to prodloop.  loop plotj = 1 to ncol(zestvals).  do if (jmed = 1).  compute probeplt(:,(wherexz(1,i)+plotcnt))=xestvals(:,ploti)&*zestvals(:,plotj).  end if.  do if (jmed > 1).  compute probeplt(:,(wheremz(((jmed*2)-3),i)+plotcnt))=mestvals(:,ploti)&*zestvals(:, plotj).  end if.  compute plotcnt=plotcnt+1.  end loop.  end loop.  end if.  do if (wzcmat((i+1),jmed))=1.  compute plotcnt=0.  compute threeway=1.  loop ploti = 1 to ncol(westvals).  loop plotj = 1 to ncol(zestvals).  compute probeplt(:,(wherewz(1,i)+plotcnt))=westvals(:,ploti)&*zestvals(:,plotj).  compute plotcnt=plotcnt+1.  end loop.  end loop.  compute plotcnt=0.  loop plotk = 1 to prodloop.  loop ploti = 1 to ncol(westvals).  loop plotj = 1 to ncol(zestvals).  do if (jmed = 1).  compute probeplt(:,(wherexwz(1,i)+plotcnt))=xestvals(:,plotk)&*westvals(:,ploti)&* zestvals(:,plotj).  end if.  do if (jmed > 1).  compute probeplt(:,(wheremwz(((jmed*2)-3),i)+plotcnt))=mestvals(:,plotk)&* westvals(:,ploti)&*zestvals(:,plotj).  end if.  compute plotcnt=plotcnt+1.  end loop.  end loop.  end loop.  end if.  loop newplp=1 to i.  do if (newplp <> jmed).  do if (wcmat((i+1),newplp))=1.  compute prodloop=1.  do if (newplp=1).  compute prodloop=nxvls.  end if.  compute plotcnt=0.  loop ploti = 1 to prodloop.  loop plotj = 1 to nwvls.  do if (newplp = 1).  compute probeplt(:,(wherexw(1,i)+plotcnt))=probeplt(:,(1+ploti))&*probeplt(:, (wherew(1,i)+plotj-1)).  end if.  do if (newplp > 1).  do if (model <> 74).  compute probeplt(:,(wheremw(((newplp*2)-3) ,i)+plotcnt))=probeplt(:,wherem((newplp-1),i))&*probeplt(:,(wherew(1,i)+plotj-1)).  end if.  do if (model = 74).  compute probeplt(:,(wheremw(((newplp*2)-3) ,i)+plotcnt))=probeplt(:,wherem((newplp-1),i))&*probeplt(:,(wherex(1,i)+plotj-1)).  end if.  end if.  compute plotcnt=plotcnt+1.  end loop.  end loop.  end if.  do if (zcmat((i+1),newplp))=1.  compute prodloop=1.  do if (newplp=1).  compute prodloop=nxvls.  end if.  compute plotcnt=0.  loop ploti = 1 to prodloop.  loop plotj = 1 to nzvls.  do if (newplp = 1).  compute probeplt(:,(wherexz(1,i)+plotcnt))=probeplt(:,(1+ploti))&*probeplt(:, (wherez(1,i)+plotj-1)).  end if.  do if (newplp > 1).  compute probeplt(:,(wheremz(((newplp*2)-3),i)+plotcnt))=probeplt(:, wherem((newplp-1),i))&*probeplt(:,(wherez(1,i)+plotj-1)).  end if.  compute plotcnt=plotcnt+1.  end loop.  end loop.  end if.  do if (wzcmat((i+1),newplp))=1.  compute plotcnt=0.  do if (threeway=0).  loop ploti = 1 to nwvls.  loop plotj = 1 to nzvls.  compute probeplt(:,(wherewz(1,i)+plotcnt))=probeplt(:,(wherew(1,i)+ploti-1))&* probeplt(:,(wherez(1,i)+plotj-1)).  compute plotcnt=plotcnt+1.  end loop.  end loop.  end if.  compute prodloop=1.  do if (newplp=1).  compute prodloop=nxvls.  end if.  compute plotcnt=0.  loop plotk = 1 to prodloop.  loop ploti = 1 to nwvls.  loop plotj = 1 to nzvls.  do if (newplp = 1).  compute probeplt(:,(wherexwz(1,i)+plotcnt))=probeplt(:,(1+plotk))&*probeplt(:, (wherew(1,i)+ploti-1))&*probeplt(:,(wherez(1,i)+plotj-1)).  end if.  do if (newplp > 1).  compute probeplt(:,(wheremwz(((newplp*2)-3),i)+plotcnt))=probeplt(:, wherem((newplp-1),i))&*probeplt(:,(wherew(1,i)+ploti-1))&*probeplt(:,(wherez(1,i)+plotj-1)).  end if.  compute plotcnt=plotcnt+1.  end loop.  end loop.  end loop.  end if.  end if.  end loop.  compute predvals=probeplt*b.  do if (debug <> 0).  print probeplt.  end if.  do if (i = nms+nys) and (ydich=1).  compute predvalt=(predvals < 709.7).  compute prevalt7=(1-predvalt)*(709.7).  compute predvals=(predvals&*predvalt)+prevalt7.  compute expyhat=exp(predvals)&/(1+exp(predvals)).  end if.  compute sepred=make(nrow(plotvals),3,999).  loop sei=1 to nrow(plotvals).  compute ask=probeplt(sei,:).  compute sepred(sei,1)=sqrt(ask*varb*t(ask)).  do if ((i < nms+nys) or (ydich=0)).  compute sepred(sei,2)=predvals(sei,1)-tval*sepred(sei,1).  compute sepred(sei,3)=predvals(sei,1)+tval*sepred(sei,1).  end if.  do if ((i = nms+nys) and (ydich=1)).  compute sepred(sei,2)=predvals(sei,1)-xp2*sepred(sei,1).  compute sepred(sei,3)=predvals(sei,1)+xp2*sepred(sei,1).  end if.  end loop.  compute prevloc=ncol(plotvals)+1.  compute probeplt={plotvals,predvals}.  do if (plot = 2).  compute probeplt={probeplt,sepred}.  end if.  do if ((i = nms+nys) and (ydich=1)).  compute probeplt={probeplt,expyhat}.  end if.  compute didsome=0.  end if.  do if ((wzcmat((i+1),jmed)=1) and (printpbe <= intprobe)).  do if (jmed=1).  compute omnilp2=nxvls*nwvls.  compute omnitmp=ident(omnilp2).  compute omni3(wherexw(1,i):wherexw(2,i),:)=omnitmp.  end if.  do if (jmed>1).  compute omnilp2=nwvls.  compute omnitmp=ident(omnilp2).  compute omni3(wheremw(((jmed*2)-3),i):wheremw(((jmed*2)-2),i),:)=omnitmp.  end if.  compute omnif=make(1,4,0).  do if ((i = nms+nys) and (ydich=1)).  compute omnif=make(1,3,0).  end if.  compute condeff3=0.  loop omnilp1=1 to nrow(zprobval).  loop omnilp=1 to (omnilp2).  do if (jmed=1).  compute omni3((wherexwz(1,i)+((omnilp-1)*nzvls)):(wherexwz(1,i)+((omnilp-1)*nzvls)+ (nzvls-1)),omnilp)=t(zprobval(omnilp1,:)).  end if.  do if (jmed > 1).  compute omni3((wheremwz(((jmed*2)-3),i)+((omnilp-1)*nzvls)):(wheremwz(((jmed*2)-3),i)+ ((omnilp-1)*nzvls)+(nzvls-1)),omnilp)=t(zprobval(omnilp1,:)).  end if.  end loop.  compute condeff=t(omni3)*b.  compute condeff3={condeff3;condeff}.  .  compute lmat2= omni3.  do if ( 1 =0).  compute lmat2 = mdiag( omni3 ).  compute lmat3=make(nrow(lmat2),1,0).  loop flp=1 to ncol(lmat2).  do if (csum(lmat2(:,flp))=1).  compute lmat3={lmat3,lmat2(:,flp)}.  end if.  end loop.  compute lmat2=lmat3(:,2:ncol(lmat3)).  end if.  compute fratio = (t(t(lmat2)* b )*inv(t(lmat2)* varb *lmat2)*((t(lmat2)* b )))/ncol(lmat2).  compute pfr = 1-fcdf(fratio,ncol(lmat2),(n-nrow( b ))).  compute fresult={fratio,ncol(lmat2),(n-nrow( b )),pfr}.  do if (i = (nms+nys) and (ydich=1)).  compute fratio=fratio*ncol(lmat2).  compute pfr=1-chicdf(fratio,ncol(lmat2)).  compute fresult={fratio,ncol(lmat2),pfr}.  end if.  do if ( 0 =1).  compute lmat3=1-rsum(lmat2).  compute xfm=make(n,csum(lmat3),0).  compute flpc=1.  loop flp=1 to nrow(lmat3).  do if (lmat3(flp,1)=1).  compute xfm(:,flpc)=x(:,flp).  compute flpc=flpc+1.  end if.  end loop.  compute bfm=inv(t(xfm)*xfm)*t(xfm)*y.  compute resid=y-(xfm*bfm).  compute sstotal=(y-(csum(y)/n)).  compute sstotal=csum(sstotal&*sstotal).  compute ssresid=csum(resid&*resid).  compute rsqch= 0 -((sstotal-ssresid)/sstotal).  compute fresult={rsqch,fresult}.  release xfm,flpc, resid, ssresid, bfm.  end if  .  compute omnif={omnif;fresult}.  end loop.  compute omnif=omnif(2:nrow(omnif),:).  compute clabtmp=znames.  compute condeff3=condeff3(2:nrow(condeff3),:).  do if ((nxvls*nwvls)=1).  compute omnif={condeff3,omnif}.  compute clabtmp={clabtmp,'Effect'}.  end if.  compute omnif={zmodvals,omnif}.  do if ((i < nms+nys) or (ydich=0)).  compute clabtmp={clabtmp,hcflab,'df1','df2','p'}.  end if.  do if ((i = (nms+nys)) and (ydich=1)).  compute clabtmp={clabtmp,'Chi-sq','df','p'}.  end if.  do if (jmed=1).  print omnif/title='Test of conditional X*W interaction at value(s) of Z:'/cnames=clabtmp/format= F10.4.  end if.  do if (jmed>1).  print omnif/title='Test of conditional M*W interaction at value(s) of Z:'/cnames=clabtmp/format= F10.4.  end if.  release omni3.  end if.  loop probei = 1 to probexpl.  do if (probexpl > 1).  compute foctmp=make(nrow(modvals),probexpl,0).  compute foctmp(:,probei)=foctmp(:,probei)+1.  compute probtemp=make(nrow(modvals),1,0).  loop probem = 1 to numplps.  loop probek = 1 to nxvls.  loop probej=lpstsp(probem,1) to lpstsp(probem,2).  compute probtemp={probtemp,foctmp(:,probek)&*probeval(:,probej)}.  end loop.  end loop.  end loop.  compute probeva2=probtemp(:,2:ncol(probtemp)).  compute probeva2={foctmp,probeva2}.  end if.  compute probres=probeva2*probcoef.  compute probrese=sqrt(diag(probeva2*probvarb*t(probeva2))).  compute tratio = probres&/probrese.  compute p = 2*(1-tcdf(abs(tratio), dfres)).  do if (ydich=1 and i = (nms+nys)).  compute p = 2*(1-cdfnorm(abs(tratio))).  end if.  compute modvals2={modvals,probres,probrese,tratio, p}.  do if ((i < nms+nys) or (ydich=0)).  compute modvals2={modvals2,(probres-tval&*probrese),(probres+tval&*probrese)}.  compute problabs={problabs,'Effect',hclab,'t', 'p', 'LLCI', 'ULCI'}.  end if.  do if (ydich=1 and i = (nms+nys)).  compute modvals2={modvals2,(probres-xp2&*probrese),(probres+xp2&*probrese)}.  compute problabs={problabs,'Effect','se','Z', 'p', 'LLCI', 'ULCI'}.  end if.  do if (probexpl > 1 and (printpbe <= intprobe)).  do if (hasz = 1).  compute printz=1.  end if.  do if (hasw=1).  compute printw=1.  end if.  compute probrlab=make(nrow(modvals),1,xcatlab(probei,1)).  compute modvals3={modvals3; modvals2}.  compute probrow={probrow;probrown}.  compute probrown=probrown+1.  do if (probei=probexpl).  compute xproblab=xcatlab(1:nxvls,1).  compute probrow=probrow(2:nrow(probrow),1).  compute modvals3=modvals3(2:nrow(modvals3),:).  compute temp=modvals3.  compute temp(GRADE(probrow(:,1)),:)=modvals3.  compute modvals3=temp.  compute start2=1.  compute problabs=problabs(1,(1+(ncol(modvarl))):ncol(problabs)).  compute pstart=1.  loop probek= 1 to nrow(probeval).  compute endstart=start2+(nxvls-1).  compute temp=modvals3(start2:endstart,(1+ncol(modvarl)):ncol(modvals3)).  compute temp2=t(modvals3(start2:start2,1:ncol(modvarl))).  compute trnames=t(modvarl).  do if (probek > 1).  print/title='----------'/space=0.  else.  print/title = 'Conditional effects of the focal predictor at values of the moderator(s):'.  do if ((jmed=1) and (i = (nms+nys)) and (nms > 0)).  do if (nxvls = 1).  print/title = '(These are also the conditional direct effects of X on Y)'/space=0.  else.  print/title = '(These are also the relative conditional direct effects of X on Y)'/space=0.  end if.  end if.  print.  end if.  print temp2/title = 'Moderator value(s):'/rnames=trnames/format= F10.4 /space=0.  print temp/title = ' '/cnames=problabs/rnames=xproblab/format= F10.4 /space=0.  compute start2=start2+nxvls.  compute didsome=1.  do if (jmed=1).  compute mod1val=probeval(probek,1:nm1vls).  loop omnilp=1 to nxvls.  compute omni((wheremv1(1,i)+((omnilp-1)*nm1vls)):(wheremv1(1,i)+((omnilp-1)* nm1vls)+(nm1vls-1)),omnilp)=t(mod1val).  do if (nm1vls < ncol(probeval)).  compute mod2val=probeval(probek,(nm1vls+1):(nm1vls+nm2vls)).  compute omni((wheremv2(1,i)+((omnilp-1)*nm2vls)):(wheremv2(1,i)+((omnilp-1)* nm2vls)+(nm2vls-1)),omnilp)=t(mod2val).  do if ((nm1vls+nm2vls) < ncol(probeval)).  compute intlen=nm1vls*nm2vls.  compute modintvl=probeval(probek,(nm1vls+nm2vls+1):ncol(probeval)).  compute omni((wherexwz(1,i)+((omnilp-1)*intlen)):(wherexwz(1,i)+((omnilp-1)* intlen)+(intlen-1)),omnilp)=t(modintvl).  end if.  end if.  end loop.  .  compute lmat2= omni.  do if ( 1 =0).  compute lmat2 = mdiag( omni ).  compute lmat3=make(nrow(lmat2),1,0).  loop flp=1 to ncol(lmat2).  do if (csum(lmat2(:,flp))=1).  compute lmat3={lmat3,lmat2(:,flp)}.  end if.  end loop.  compute lmat2=lmat3(:,2:ncol(lmat3)).  end if.  compute fratio = (t(t(lmat2)* b )*inv(t(lmat2)* varb *lmat2)*((t(lmat2)* b )))/ncol(lmat2).  compute pfr = 1-fcdf(fratio,ncol(lmat2),(n-nrow( b ))).  compute fresult={fratio,ncol(lmat2),(n-nrow( b )),pfr}.  do if (i = (nms+nys) and (ydich=1)).  compute fratio=fratio*ncol(lmat2).  compute pfr=1-chicdf(fratio,ncol(lmat2)).  compute fresult={fratio,ncol(lmat2),pfr}.  end if.  do if ( 0 =1).  compute lmat3=1-rsum(lmat2).  compute xfm=make(n,csum(lmat3),0).  compute flpc=1.  loop flp=1 to nrow(lmat3).  do if (lmat3(flp,1)=1).  compute xfm(:,flpc)=x(:,flp).  compute flpc=flpc+1.  end if.  end loop.  compute bfm=inv(t(xfm)*xfm)*t(xfm)*y.  compute resid=y-(xfm*bfm).  compute sstotal=(y-(csum(y)/n)).  compute sstotal=csum(sstotal&*sstotal).  compute ssresid=csum(resid&*resid).  compute rsqch= 0 -((sstotal-ssresid)/sstotal).  compute fresult={rsqch,fresult}.  release xfm,flpc, resid, ssresid, bfm.  end if  .  do if (i < (nms + nys) or (ydich = 0)).  compute clabtmp={hcflab,'df1','df2','p'}.  print fresult/title='Test of equality of conditional means'/cnames=clabtmp/format= F10.4.  end if.  do if (i = (nms + nys) and (ydich = 1)).  compute clabtmp={'Chi-sq','df','p'}.  print fresult/title='Test of equality of conditional logits or probabilities'/cnames=clabtmp/format= F10.4.  end if.  compute probetmp=probeplt(pstart:(pstart+nxvls),1).  compute probetmp={probetmp,probeplt(pstart:(pstart+nxvls),prevloc:ncol(probeplt))}.  compute pstart=pstart+(nxvls+1).  do if (i < ((nms + nys)) or (ydich = 0)).  compute clabtmp={xnames, outnames(1,i), hclab, 'LLCI', 'ULCI'}.  print probetmp/title = 'Estimated conditional means being compared:'/cnames=clabtmp/format= F10.4.  end if.  do if ((i = (nms+nys)) and (ydich = 1)).  compute clabtmp={xnames, outnames(1,i), 'prob'}.  compute probetm2=probetmp(:,1:2).  compute probetm2={probetm2,probetmp(:,ncol(probetmp))}.  print probetm2/title = 'Estimated conditional logits and probabilities:'/cnames=clabtmp/format= F10.4.  end if.  end if.  end loop.  release probrow, start2, endstart, temp, temp2.  end if.  end if.  do if (probexpl = 1 and (printpbe <= intprobe)).  print/title = 'Conditional effects of the focal predictor at values of the moderator(s):'.  print modvals2/cnames=problabs/title = ' '/space=0/format= F10.4.  compute didsome=1.  do if (hasz = 1).  compute printz=1.  end if.  do if (hasw=1).  compute printw=1.  end if.  do if (jn = 1 and jnok=1).  do if (criterr = 0).  compute dfres=n-nrow(b).  compute jncrit =(dfres* (exp((dfres-(5/6))*((xp2/(dfres-(2/3)+(.11/dfres)))* (xp2/(dfres-(2/3)+(.11/dfres)))))-1)).  do if (i = (nms+nys) and (ydich=1)).  compute jncrit=xp2*xp2.  end if.  compute jnb1=b(wherejn1,1).  compute jnb3=b(wherejn3,1).  compute jnsb1=varb(wherejn1,wherejn1).  compute jnsb3=varb(wherejn3,wherejn3).  compute jnsb1b3=varb(wherejn1,wherejn3).  compute ajn =(jncrit*jnsb3)-(jnb3*jnb3).  compute bjn = 2*((jncrit*jnsb1b3)-(jnb1*jnb3)).  compute cjn = (jncrit*jnsb1)-(jnb1*jnb1).  compute radarg = (bjn*bjn)-(4*ajn*cjn).  compute den = 2*ajn.  compute nrts = 0.  do if (radarg >= 0 and den <> 0).  compute x21 = (-bjn+sqrt(radarg))/den.  compute x22 = (-bjn-sqrt(radarg))/den.  compute roots = 0.  do if (x21 >= jnmin and x21 <= jnmax).  compute nrts = 1.  compute roots = {roots; x21}.  end if.  do if (x22 >= jnmin and x22 <= jnmax).  compute nrts = nrts + 1.  compute roots = {roots; x22}.  end if.  compute roots={roots,make(nrow(roots),2,0)}.  end if.  do if (nrts > 0).  compute roots = roots(2:nrow(roots),1:3).  compute roots(1,2)=(csum(jnmod < roots(1,1))/n)*100.  compute roots(1,3)=(csum(jnmod > roots(1,1))/n)*100.  do if (nrow(roots)=2).  compute roots(2,2)=(csum(jnmod < roots(2,1))/n)*100.  compute roots(2,3)=(csum(jnmod > roots(2,1))/n)*100.  end if.  print roots/title = 'Moderator value(s) defining Johnson-Neyman significance region(s):'/clabels = 'Value', '% below', '% above'/format F10.4.  end if.  do if (nrts = 0).  print/title = 'There are no statistical significance transition points within the observed'.  print/title = 'range of the moderator found using the Johnson-Neyman method.'/space=0.  end if.  compute jnvals=make((21+nrts),7,0).  loop jni= 0 to 20.  compute jnvals((jni+1),1)=jnmin+(jni*((jnmax-jnmin)/20)).  end loop.  do if (nrts > 0).  loop jni = 1 to nrts.  loop jnj = 2 to nrow(jnvals).  do if ((roots(jni,1) > jnvals((jnj-1),1)) and (roots(jni,1) < jnvals(jnj,1))).  compute jnvals((jnj+1):(21+jni),1)=jnvals(jnj:(20+jni),1).  compute jnvals(jnj,1)=roots(jni,1).  end if.  end loop.  end loop.  end if.  loop jni = 1 to nrow(jnvals).  compute jnvals(jni,2)=jnb1+jnb3*jnvals(jni,1).  compute jnvals(jni,3)=sqrt(jnsb1+2*jnvals(jni,1)*jnsb1b3+(jnvals(jni,1)*jnvals(jni, 1))*jnsb3).  compute jnvals(jni,4)=jnvals(jni,2)/jnvals(jni,3).  compute jnvals(jni,5)=2*(1-tcdf(abs(jnvals(jni,4)), dfres)).  compute jnvals(jni,6)=jnvals(jni,2)-sqrt(jncrit)*jnvals(jni,3).  compute jnvals(jni,7)=jnvals(jni,2)+sqrt(jncrit)*jnvals(jni,3).  do if ((i = nms + nys) and (ydich=1)).  compute jnvals(jni,5)=2*(1-cdfnorm(abs(jnvals(jni,4)))).  compute jnvals(jni,6)=jnvals(jni,2)-xp2*jnvals(jni,3).  compute jnvals(jni,7)=jnvals(jni,2)+xp2*jnvals(jni,3).  end if.  end loop.  do if ((i < nms+nys) or (ydich=0)).  compute jnclbs={jnmodlab,'Effect',hclab,'t', 'p', 'LLCI', 'ULCI'}.  end if.  do if ((i = nms + nys) and (ydich=1)).  compute jnclbs={jnmodlab,'Effect','se','Z', 'p', 'LLCI', 'ULCI'}.  end if.  do if (((wcmat((i+1),jmed)=1) or (zcmat((i+1),jmed)=1)) and (wzcmat((i+1),jmed)=0)).  print jnvals/title = 'Conditional effect of focal predictor at values of the moderator:'/cnames =jnclbs/format = F10.4.  end if.  do if ((jmed = 1) and (wzcmat((i+1),jmed)=1)).  print jnvals/title = 'Conditional X*W interaction at values of the moderator Z:'/cnames =jnclbs/format = F10.4.  end if.  do if ((jmed > 1) and (wzcmat((i+1),jmed)=1)).  print jnvals/title = 'Conditional M*W interaction at values of the moderator Z:'/cnames =jnclbs/format = F10.4.  end if.  end if.  end if.  end if.  do if ((i = (nms+nys)) and (jmed=1) and (bcmat(nrow(bcmat),1)=1)).  do if (probei=1).  compute direfflb=problabs.  compute direff=modvals2.  end if.  do if (probei>1).  compute direff={direff;modvals2}.  end if.  end if.  compute intprint=0.  do if ((jmed=1) and (i=1) and nms=0) and modcok=1).  compute contvec2=make(2,1,1).  compute contvec2={contvec2,wcontval,zcontval}.  do if (wzcmat((i+1),jmed)=1).  loop conti= 1 to ncol(wcontval).  loop contj = 1 to ncol(zcontval).  compute contvec2={contvec2,wcontval(:,conti)&*zcontval(:,contj)}.  end loop.  end loop.  end if.  compute conteff=contvec2*probcoef.  compute contdiff=contvec2(1,:)-contvec2(2,:).  compute contse=sqrt(contdiff*probvarb*t(contdiff)).  compute conteffd=conteff(1,1)-conteff(2,1).  compute contvec={contvec,conteff}.  print/title='Contrast between conditional effects of X:'.  print contvec/title=' '/rlabels='Effect1:','Effect2:'/cnames=problabs/format = F10.4 /space=0.  do if (ydich=0).  compute p=2*(1-tcdf(abs(conteffd/contse), dfres)).  compute contvec={conteffd,contse,conteffd/contse, p}.  compute contvec={contvec,(conteffd-(tval*contse))}.  compute contvec={contvec,(conteffd+(tval*contse))}.  compute contlabs={'Contrast', hclab, 't', 'p', 'LLCI', 'ULCI'}.  end if.  do if (ydich=1).  compute p=2*(1-cdfnorm(abs(conteffd/contse))).  compute contvec={conteffd,contse,conteffd/contse, p}.  compute contvec={contvec,(conteffd-(xp2*contse))}.  compute contvec={contvec,(conteffd+(xp2*contse))}.  compute contlabs={'Contrast', 'se', 'Z', 'p', 'LLCI', 'ULCI'}.  end if.  print contvec/title='Test of Effect1 minus Effect2'/format= F10.4 /cnames=contlabs.  end if.  end loop.  do if (plot = 1 or plot = 2).  compute datalabs={t(focpred(:,3)),outnames(1,i)}.  do if (plot = 1).  compute datalabs={datalabs}.  end if.  do if (plot = 2).  compute datalabs={datalabs,'se', 'LLCI', 'ULCI'}.  end if.  do if ((i = nms+nys) and (ydich=1)).  compute datalabs={datalabs,'prob'}.  end if.  compute datalabs={datalabs,'.'}.  print/title = 'Data for visualizing the conditional effect of the focal predictor:'.  print/title = 'Paste text below into a SPSS syntax window and execute to produce plot.'/space=0.  compute dumb = {' ', ' ', ' ', ' ', ' ', ' ', ' '}.  print datalabs/title = 'DATA LIST FREE/'/format=A10.  print probeplt/title = 'BEGIN DATA.'/format.= F10.4 /space=0.  print/title = 'END DATA.'/space=0.  compute focgrph=datalabs(1,1).  compute graphix={focgrph,graphixs,modgrph}.  do if (((xdich=1) or (nxvls > 1)) and ((modcat=0) and (focgrph = xnames))).  compute graphix={modgrph,graphixs,focgrph}.  end if.  do if (panelgrp = 0).  compute graphix={graphix,'.'}.  else.  compute graphix={graphix,panelcde}.  end if.  print graphix/title = 'GRAPH/SCATTERPLOT='/format=A8/space=0.  do if (i = (nms + nys) and ydich = 1).  compute graphixd=graphix.  compute graphixd(1,3)='prob'.  print graphixd/title = 'GRAPH/SCATTERPLOT='/format=A8/space=0.  end if.  end if.  end if.  end loop.  release jmed, intprint,didprint.  end if  .  do if (model = 74 and i <= nms).  print/title='Here are estimates of M'.  compute onetemp=make(nrow(xprobval),1,1).  compute mestmt74={onetemp,xprobval}.  do if (ncs > 0).  compute ncovmdl=rsum(ccmat(i,:)).  do if (ncovmdl > 0).  compute cvmnc=make(nrow(mestmt74),ncovmdl,1).  compute cvmnctmp=csum(x(:,(ncol(x)-ncovmdl+1):ncol(x)))/nrow(x).  loop mestlp=1 to ncovmdl.  compute cvmnc(:,mestlp)=cvmnc(:,mestlp)*cvmnctmp(:,mestlp).  end loop.  compute mestmt74={mestmt74,cvmnc}.  end if.  end if.  print mestmt74.  compute mest74t=mestmt74*b.  do if (i = 1).  compute mest74=mest74t.  end if.  do if (i > 1).  compute mest74={mest74,mest74t}.  end if.  print mest74.  end if.  end loop if criterr=1.  do if (criterr=0 and dototal = 1).  print/title = '************************** TOTAL EFFECT MODEL ****************************'.  print outnames(1,ncol(outnames))/title = 'OUTCOME VARIABLE:'/format = A8/space=0.  compute x=xtmp.  compute vlabsm={'constant';xcatlab(1:nxvls,1)}.  do if (ncs > 0).  compute x = {x,ctmp}.  compute vlabsm={vlabsm;t(covnames)}.  end if.  compute x = {ones,x}.  .  do if ( 1 =1).  compute b = inv(t( x )* x )*t( x )* y.  compute modres=b.  do if ( 1 =1).  compute n1=nrow( x ).  compute dfres=n1-(ncol( x )).  compute sstotal = t( y -(csum( y )/n1))*( y -(csum( y )/n1)).  compute resid= y - x *b.  compute ssresid = csum((resid)&**2).  compute r2 = (sstotal-ssresid)/sstotal.  compute adjr2 = 1-((1-r2)*(n1-1)/(dfres)).  compute mse=ssresid/(n1-ncol( x )).  .  compute n1=nrow( x ).  compute invXtX = inv(t( x )* x ).  compute varb = mse *invXtX.  compute k3 = ncol( x ).  compute xhc=0.  do if ( hc <> 5).  compute xhc= x.  compute hat = xhc(:,1).  loop i3=1 to nrow(xhc).  compute hat(i3,1)= xhc(i3,:)*invXtX*t(xhc(i3,:)).  end loop.  do if ( hc = 0 or hc =1).  loop i3 = 1 to k3.  compute xhc(:,i3)=xhc(:,i3)&* resid.  end loop.  end if.  do if ( hc =3 or hc =2).  loop i3=1 to k3.  compute xhc(:,i3) = ( resid &/(1-hat)&**(1/(4hc )))&*xhc(:,i3).  end loop.  end if.  do if ( hc = 4).  compute hcmn=make(n,2,4).  compute hcmn(:,2)=(n1*hat)/k3.  loop i3= 1 to k3.  compute xhc(:,i3) = ( resid &/(1-hat)&**(rmin(hcmn)/2))&*xhc(:,i3).  end loop.  end if.  compute varb=(invXtX*t(xhc)*xhc*invXtX).  do if ( hc =1).  compute varb=(n1/(n1-ncol( x )))&*varb.  end if.  end if.  compute hclab={'se(HC0)','se(HC1)','se(HC2)','se(HC3)','se(HC4)','se'}.  compute hclab=hclab(1,( hc +1)).  compute hcflab={'F(HC0)','F(HC1)','F(HC2)','F(HC3)','F(HC4)','F'}.  compute hcflab=hcflab(1,( hc +1)).  release xhc  .  compute seb=sqrt(diag(varb)).  compute trat = b&/seb.  compute p = 2*(1-tcdf(abs(trat), (dfres))).  compute tval = sqrt(dfres* (exp((dfres-(5/6))*((xp2/(dfres-(2/3)+(.11/dfres)))* (xp2/(dfres-(2/3)+(.11/dfres)))))-1)).  compute modres={modres,seb,trat,p}.  compute modres={modres,(b-tval&*seb),(b+tval&*seb)}.  compute modresl={'coeff',hclab,'t','p','LLCI','ULCI'}.  compute lmat = ident(ncol( x )).  compute lmat = lmat(:,2:ncol(lmat)).  compute fratio = (t(t(lmat)*b)*inv(t(lmat)*varb*lmat)*((t(lmat)*b)))/(ncol( x )-1).  compute pfr = 1-fcdf(fratio,(ncol( x )-1),dfres).  compute modsum={sqrt(r2),r2,mse,fratio,(ncol( x )-1),dfres,pfr}.  compute modsuml={'R','R-sq','MSE',hcflab,'df1','df2', 'p'}.  end if.  end if.  do if ( 1 = 2 or 1 =3).  compute xlp= x.  compute ylp= y.  compute pt2 = make(nrow(ylp),1,(csum(ylp)/nrow(ylp))).  do if ( 1 =2).  compute LL3 = ylp&*ln(pt2)+(1-ylp)&*ln(1-pt2).  end if.  compute LL3 = -2*csum(LL3).  compute bt1 = make(ncol(xlp),1,0).  compute LL1 = 0.  compute pt1 = make(nrow(ylp),1,0.5).  compute pt1lp=pt1.  loop jjj = 1 to iterate.  compute vt1 = mdiag(pt1lp&*(1-pt1lp)).  compute b = bt1+inv(t(xlp)*vt1*xlp)*t(xlp)*(ylp-pt1lp).  do if ( 1 =2).  compute xlpb=xlp*b.  compute xlpbt=(xlpb > -709.7).  compute xlpb709=(1-xlpbt)*(-709.7).  compute xlpb=(xlpb&*xlpbt)+xlpb709.  compute pt1lp = 1/(1+exp(-(xlpb))).  end if.  compute itprob = csum((pt1lp < .00000001) or (pt1lp > .9999999)).  do if (itprob > 0).  loop kkk = 1 to nrow(pt1lp).  do if (pt1lp(kkk,1) > .9999999).  compute pt1lp(kkk,1) = .9999999.  end if.  do if (pt1lp(kkk,1) < .00000001).  compute pt1lp(kkk,1) = .00000001.  end if.  end loop.  compute itprob = 0.  end if.  do if (itprob = 0).  do if ( 1 =2).  compute LL = ylp&*ln(pt1lp)+(1-ylp)&*ln(1-pt1lp).  end if.  compute LL2 = -2*csum(ll).  end if.  do if (abs(LL1-LL2) < converge).  do if ( 1 =1).  compute vt1 = mdiag(pt1lp&*(1-pt1lp)).  compute varb = inv(t(xlp)*vt1*xlp).  compute seb = sqrt(diag(varb)).  end if.  break.  end if.  compute bt1 = b.  compute LL1 = LL2.  end loop.  compute modres=b.  do if (jjj > iterate).  compute itprob = 2.  do if (booting=0).  compute iterrmod=1.  end if.  do if (booting=1).  compute bootiter=1.  end if.  do if (itprobtg=0).  compute itprobtg=1.  compute errcode(errs,1) = 47.  compute errs = errs + 1.  do if (booting = 0 and 1 =1).  compute vt1 = mdiag(pt1lp&*(1-pt1lp)).  compute varb = inv(t(xlp)*vt1*xlp).  compute seb = sqrt(diag(varb)).  end if.  end if.  end if.  do if ( 1 =1).  compute trat = b&/seb.  compute dfres=nrow(xlp).  compute p = 2*(1-cdfnorm(abs(trat))).  compute modres={modres,seb,trat,p}.  compute modres={modres,(b-xp2&*seb),(b+xp2&*seb)}.  compute pvchi=1-chicdf((LL3-LL2),(nrow(modres)-1)).  compute mcF = (LL3-LL2)/LL3.  compute cox = 1-exp(-(LL3-LL2)/nrow(xlp)).  compute nagel = cox/(1-exp(-(LL3)/nrow(xlp))).  compute modsum={LL2,(LL3-LL2),(nrow(modres)-1),pvchi, mcF,cox,nagel}.  compute modsuml={'-2LL','ModelLL', 'df', 'p', 'McFadden', 'CoxSnell', 'Nagelkrk'}.  compute modresl={'coeff','se','Z','p','LLCI','ULCI'}.  end if.  end if  .  print modsum/title = 'Model Summary'/cnames = modsuml/format= F10.4.  print modres/title='Model'/rnames=vlabsm/cnames=modresl/format= F10.4.  compute toteff=modres(2:(1+nxvls),:).  compute totefflb=modresl.  compute toteffl2=vlabsm(2:(1+nxvls),:).  compute lmat=make(nrow(b),1,0).  compute lmat2=make(nxvls,1,1).  compute lmat(2:(1+nxvls),1)=lmat2.  do if (ydich <> 1).  .  compute lmat2= lmat.  do if ( 0 =0).  compute lmat2 = mdiag( lmat ).  compute lmat3=make(nrow(lmat2),1,0).  loop flp=1 to ncol(lmat2).  do if (csum(lmat2(:,flp))=1).  compute lmat3={lmat3,lmat2(:,flp)}.  end if.  end loop.  compute lmat2=lmat3(:,2:ncol(lmat3)).  end if.  compute fratio = (t(t(lmat2)* b )*inv(t(lmat2)* varb *lmat2)*((t(lmat2)* b )))/ncol(lmat2).  compute pfr = 1-fcdf(fratio,ncol(lmat2),(n-nrow( b ))).  compute fresult={fratio,ncol(lmat2),(n-nrow( b )),pfr}.  do if (i = (nms+nys) and (ydich=1)).  compute fratio=fratio*ncol(lmat2).  compute pfr=1-chicdf(fratio,ncol(lmat2)).  compute fresult={fratio,ncol(lmat2),pfr}.  end if.  do if ( 1 =1).  compute lmat3=1-rsum(lmat2).  compute xfm=make(n,csum(lmat3),0).  compute flpc=1.  loop flp=1 to nrow(lmat3).  do if (lmat3(flp,1)=1).  compute xfm(:,flpc)=x(:,flp).  compute flpc=flpc+1.  end if.  end loop.  compute bfm=inv(t(xfm)*xfm)*t(xfm)*y.  compute resid=y-(xfm*bfm).  compute sstotal=(y-(csum(y)/n)).  compute sstotal=csum(sstotal&*sstotal).  compute ssresid=csum(resid&*resid).  compute rsqch= r2 -((sstotal-ssresid)/sstotal).  compute fresult={rsqch,fresult}.  release xfm,flpc, resid, ssresid, bfm.  end if  .  compute totomni=fresult.  end if.  do if (stand=1).  compute predsd=make(nrow(modres),1,0).  compute stdmod=modres(:,1)&/ovsd(1,ncol(ovsd)).  loop jd=1 to ncol(x).  compute descdat=x(:,jd).  compute predsd(jd,1) = (nrow(descdat)*sscp(descdat))-(t(csum(descdat))*(csum(descdat))).  compute predsd(jd,1) = sqrt(predsd(jd,1)/(nrow(descdat)*(nrow(descdat)-1))).  end loop.  do if (wherex(1,ncol(wherex)) <> -999 and ((nxvls > 1) or (xdich=1))).  compute sdmsone=make(nxvls,1,1).  compute predsd(wherex(1,ncol(wherex)):wherex(2,ncol(wherex)),1)=sdmsone.  compute pstog=1.  end if.  compute predsd(1,1)=1.  compute stdmod=stdmod&*predsd.  compute stdmod=stdmod(2:nrow(stdmod),1).  compute sdvlabs=vlabsm(2:nrow(vlabsm),1).  print stdmod/title='Standardized coefficients'/clabels='coeff'/rnames=sdvlabs/format= F10.4.  end if.  do if (covcoeff=1).  print varb/title='Covariance matrix of regression parameter estimates:'/rnames=vlabsm /cnames=vlabsm/format= F10.4.  end if.  end if.  end if.  do if (criterr=0 and boot > 0).  compute bootres=make(1,rsum(nump),-999).  do if (effsize=1).  compute bootysd=make(1,1,-999).  compute bootxsd=make(1,1,-999).  end if.  compute badboot=0.  compute goodboot=0.  compute smallest=1.  compute booting=1.  loop j = 1 to maxboot.  compute nobootx=1.  compute modres2=999.  compute v=trunc(uniform(n,1)*n)+1.  compute bad=0.  loop i = 1 to (nms+nys).  compute y=outvars(v,i).  compute ynovar= (nrow(y)*sscp(y))-(t(csum(y))*(csum(y))).  do if (ynovar = 0).  compute bad=1.  end if.  compute xindx=datindx(1:(nump(1,i)-1),i).  compute hello=0.  compute x = fulldat(v,xindx).  compute x={ones,x}.  compute xsq=t(x)*x.  compute exsq=eval(xsq).  release xsq.  compute holymoly=cmin(exsq).  compute zeroeig=csum(exsq <= 0.000000000002).  compute bad=bad + (zeroeig > 0).  .  compute desctmp=make((8-(4* 1 )),ncol( y ),-999).  loop jd=1 to ncol( y ).  compute descdat= y (:,jd).  compute desctmp(1,jd) = csum(descdat)/nrow(descdat).  compute desctmp(2,jd) = (nrow(descdat)*sscp(descdat))-(t(csum(descdat))*(csum(descdat))).  compute desctmp(2,jd) = sqrt(desctmp(2,jd)/(nrow(descdat)*(nrow(descdat)-1))).  compute desctmp(3,jd)=cmin(descdat).  compute desctmp(4,jd)=cmax(descdat).  do if ( 1 =0).  compute minwarn=0.  compute maxwarn=0.  do if ((desctmp(3,jd)=desctmp(4,jd)) and novar=0).  compute errcode(errs,1)=15.  compute errs=errs+1.  compute criterr=1.  compute novar=1.  end if.  compute tmp=((descdat(:,1)=desctmp(3,jd))+(descdat(:,1)=desctmp(4,jd))).  compute desctmp(8,jd)=(csum(tmp)=nrow(tmp)).  compute tmp = descdat.  compute tmp(GRADE(descdat),:) = descdat.  compute descdat = tmp.  release tmp.  compute decval={.16;.5;.84}.  loop kd=1 to 3.  compute low=trunc(decval(kd,1)*(nrow(descdat)+1)).  compute lowdec=decval(kd,1)*(nrow(descdat)+1)-low.  compute value=descdat(low,1)+(descdat((low+1),1)-descdat(low,1))*lowdec.  compute desctmp((4+kd),jd)=value.  end loop.  compute mnotev=1.  compute modvals=desctmp(5:7,:).  do if (quantile <> 1).  compute desctmp(5,jd)=desctmp(1,jd)-desctmp(2,jd).  compute desctmp(6,jd)=desctmp(1,jd).  compute desctmp(7,jd)=desctmp(1,jd)+desctmp(2,jd).  compute modvals=desctmp(5:7,:).  compute mnotev=2.  do if (modvals(1,1) < desctmp(3,1)).  compute modvals(1,1)=desctmp(3,1).  compute minwarn=1.  end if.  do if (modvals(3,1) > desctmp(4,1)).  compute modvals(3,1)=desctmp(4,1).  compute maxwarn=1.  end if.  end if.  do if (desctmp(8,1)=1).  compute modvals={desctmp(3,1);desctmp(4,1)}.  compute mnotev=0.  compute minwarn=0.  compute maxwarn=0.  end if.  end if.  end loop  .  compute bad=bad+ (desctmp(2,1) <= 0.00000000001).  do if (bad = 0).  do if (holymoly < smallest).  compute smallest=holymoly.  end if.  do if (ydich=0 or (i < (nms+nys))).  .  do if ( 1 =1).  compute b = inv(t( x )* x )*t( x )* y.  compute modres=b.  do if ( 0 =1).  compute n1=nrow( x ).  compute dfres=n1-(ncol( x )).  compute sstotal = t( y -(csum( y )/n1))*( y -(csum( y )/n1)).  compute resid= y - x *b.  compute ssresid = csum((resid)&**2).  compute r2 = (sstotal-ssresid)/sstotal.  compute adjr2 = 1-((1-r2)*(n1-1)/(dfres)).  compute mse=ssresid/(n1-ncol( x )).  .  compute n1=nrow( x ).  compute invXtX = inv(t( x )* x ).  compute varb = mse *invXtX.  compute k3 = ncol( x ).  compute xhc=0.  do if ( hc <> 5).  compute xhc= x.  compute hat = xhc(:,1).  loop i3=1 to nrow(xhc).  compute hat(i3,1)= xhc(i3,:)*invXtX*t(xhc(i3,:)).  end loop.  do if ( hc = 0 or hc =1).  loop i3 = 1 to k3.  compute xhc(:,i3)=xhc(:,i3)&* resid.  end loop.  end if.  do if ( hc =3 or hc =2).  loop i3=1 to k3.  compute xhc(:,i3) = ( resid &/(1-hat)&**(1/(4hc )))&*xhc(:,i3).  end loop.  end if.  do if ( hc = 4).  compute hcmn=make(n,2,4).  compute hcmn(:,2)=(n1*hat)/k3.  loop i3= 1 to k3.  compute xhc(:,i3) = ( resid &/(1-hat)&**(rmin(hcmn)/2))&*xhc(:,i3).  end loop.  end if.  compute varb=(invXtX*t(xhc)*xhc*invXtX).  do if ( hc =1).  compute varb=(n1/(n1-ncol( x )))&*varb.  end if.  end if.  compute hclab={'se(HC0)','se(HC1)','se(HC2)','se(HC3)','se(HC4)','se'}.  compute hclab=hclab(1,( hc +1)).  compute hcflab={'F(HC0)','F(HC1)','F(HC2)','F(HC3)','F(HC4)','F'}.  compute hcflab=hcflab(1,( hc +1)).  release xhc  .  compute seb=sqrt(diag(varb)).  compute trat = b&/seb.  compute p = 2*(1-tcdf(abs(trat), (dfres))).  compute tval = sqrt(dfres* (exp((dfres-(5/6))*((xp2/(dfres-(2/3)+(.11/dfres)))* (xp2/(dfres-(2/3)+(.11/dfres)))))-1)).  compute modres={modres,seb,trat,p}.  compute modres={modres,(b-tval&*seb),(b+tval&*seb)}.  compute modresl={'coeff',hclab,'t','p','LLCI','ULCI'}.  compute lmat = ident(ncol( x )).  compute lmat = lmat(:,2:ncol(lmat)).  compute fratio = (t(t(lmat)*b)*inv(t(lmat)*varb*lmat)*((t(lmat)*b)))/(ncol( x )-1).  compute pfr = 1-fcdf(fratio,(ncol( x )-1),dfres).  compute modsum={sqrt(r2),r2,mse,fratio,(ncol( x )-1),dfres,pfr}.  compute modsuml={'R','R-sq','MSE',hcflab,'df1','df2', 'p'}.  end if.  end if.  do if ( 1 = 2 or 1 =3).  compute xlp= x.  compute ylp= y.  compute pt2 = make(nrow(ylp),1,(csum(ylp)/nrow(ylp))).  do if ( 1 =2).  compute LL3 = ylp&*ln(pt2)+(1-ylp)&*ln(1-pt2).  end if.  compute LL3 = -2*csum(LL3).  compute bt1 = make(ncol(xlp),1,0).  compute LL1 = 0.  compute pt1 = make(nrow(ylp),1,0.5).  compute pt1lp=pt1.  loop jjj = 1 to iterate.  compute vt1 = mdiag(pt1lp&*(1-pt1lp)).  compute b = bt1+inv(t(xlp)*vt1*xlp)*t(xlp)*(ylp-pt1lp).  do if ( 1 =2).  compute xlpb=xlp*b.  compute xlpbt=(xlpb > -709.7).  compute xlpb709=(1-xlpbt)*(-709.7).  compute xlpb=(xlpb&*xlpbt)+xlpb709.  compute pt1lp = 1/(1+exp(-(xlpb))).  end if.  compute itprob = csum((pt1lp < .00000001) or (pt1lp > .9999999)).  do if (itprob > 0).  loop kkk = 1 to nrow(pt1lp).  do if (pt1lp(kkk,1) > .9999999).  compute pt1lp(kkk,1) = .9999999.  end if.  do if (pt1lp(kkk,1) < .00000001).  compute pt1lp(kkk,1) = .00000001.  end if.  end loop.  compute itprob = 0.  end if.  do if (itprob = 0).  do if ( 1 =2).  compute LL = ylp&*ln(pt1lp)+(1-ylp)&*ln(1-pt1lp).  end if.  compute LL2 = -2*csum(ll).  end if.  do if (abs(LL1-LL2) < converge).  do if ( 0 =1).  compute vt1 = mdiag(pt1lp&*(1-pt1lp)).  compute varb = inv(t(xlp)*vt1*xlp).  compute seb = sqrt(diag(varb)).  end if.  break.  end if.  compute bt1 = b.  compute LL1 = LL2.  end loop.  compute modres=b.  do if (jjj > iterate).  compute itprob = 2.  do if (booting=0).  compute iterrmod=1.  end if.  do if (booting=1).  compute bootiter=1.  end if.  do if (itprobtg=0).  compute itprobtg=1.  compute errcode(errs,1) = 47.  compute errs = errs + 1.  do if (booting = 0 and 0 =1).  compute vt1 = mdiag(pt1lp&*(1-pt1lp)).  compute varb = inv(t(xlp)*vt1*xlp).  compute seb = sqrt(diag(varb)).  end if.  end if.  end if.  do if ( 0 =1).  compute trat = b&/seb.  compute dfres=nrow(xlp).  compute p = 2*(1-cdfnorm(abs(trat))).  compute modres={modres,seb,trat,p}.  compute modres={modres,(b-xp2&*seb),(b+xp2&*seb)}.  compute pvchi=1-chicdf((LL3-LL2),(nrow(modres)-1)).  compute mcF = (LL3-LL2)/LL3.  compute cox = 1-exp(-(LL3-LL2)/nrow(xlp)).  compute nagel = cox/(1-exp(-(LL3)/nrow(xlp))).  compute modsum={LL2,(LL3-LL2),(nrow(modres)-1),pvchi, mcF,cox,nagel}.  compute modsuml={'-2LL','ModelLL', 'df', 'p', 'McFadden', 'CoxSnell', 'Nagelkrk'}.  compute modresl={'coeff','se','Z','p','LLCI','ULCI'}.  end if.  end if  .  end if.  do if (ydich=1 and (i = (nms+nys))).  .  do if ( 2 =1).  compute b = inv(t( x )* x )*t( x )* y.  compute modres=b.  do if ( 0 =1).  compute n1=nrow( x ).  compute dfres=n1-(ncol( x )).  compute sstotal = t( y -(csum( y )/n1))*( y -(csum( y )/n1)).  compute resid= y - x *b.  compute ssresid = csum((resid)&**2).  compute r2 = (sstotal-ssresid)/sstotal.  compute adjr2 = 1-((1-r2)*(n1-1)/(dfres)).  compute mse=ssresid/(n1-ncol( x )).  .  compute n1=nrow( x ).  compute invXtX = inv(t( x )* x ).  compute varb = mse *invXtX.  compute k3 = ncol( x ).  compute xhc=0.  do if ( hc <> 5).  compute xhc= x.  compute hat = xhc(:,1).  loop i3=1 to nrow(xhc).  compute hat(i3,1)= xhc(i3,:)*invXtX*t(xhc(i3,:)).  end loop.  do if ( hc = 0 or hc =1).  loop i3 = 1 to k3.  compute xhc(:,i3)=xhc(:,i3)&* resid.  end loop.  end if.  do if ( hc =3 or hc =2).  loop i3=1 to k3.  compute xhc(:,i3) = ( resid &/(1-hat)&**(1/(4hc )))&*xhc(:,i3).  end loop.  end if.  do if ( hc = 4).  compute hcmn=make(n,2,4).  compute hcmn(:,2)=(n1*hat)/k3.  loop i3= 1 to k3.  compute xhc(:,i3) = ( resid &/(1-hat)&**(rmin(hcmn)/2))&*xhc(:,i3).  end loop.  end if.  compute varb=(invXtX*t(xhc)*xhc*invXtX).  do if ( hc =1).  compute varb=(n1/(n1-ncol( x )))&*varb.  end if.  end if.  compute hclab={'se(HC0)','se(HC1)','se(HC2)','se(HC3)','se(HC4)','se'}.  compute hclab=hclab(1,( hc +1)).  compute hcflab={'F(HC0)','F(HC1)','F(HC2)','F(HC3)','F(HC4)','F'}.  compute hcflab=hcflab(1,( hc +1)).  release xhc  .  compute seb=sqrt(diag(varb)).  compute trat = b&/seb.  compute p = 2*(1-tcdf(abs(trat), (dfres))).  compute tval = sqrt(dfres* (exp((dfres-(5/6))*((xp2/(dfres-(2/3)+(.11/dfres)))* (xp2/(dfres-(2/3)+(.11/dfres)))))-1)).  compute modres={modres,seb,trat,p}.  compute modres={modres,(b-tval&*seb),(b+tval&*seb)}.  compute modresl={'coeff',hclab,'t','p','LLCI','ULCI'}.  compute lmat = ident(ncol( x )).  compute lmat = lmat(:,2:ncol(lmat)).  compute fratio = (t(t(lmat)*b)*inv(t(lmat)*varb*lmat)*((t(lmat)*b)))/(ncol( x )-1).  compute pfr = 1-fcdf(fratio,(ncol( x )-1),dfres).  compute modsum={sqrt(r2),r2,mse,fratio,(ncol( x )-1),dfres,pfr}.  compute modsuml={'R','R-sq','MSE',hcflab,'df1','df2', 'p'}.  end if.  end if.  do if ( 2 = 2 or 2 =3).  compute xlp= x.  compute ylp= y.  compute pt2 = make(nrow(ylp),1,(csum(ylp)/nrow(ylp))).  do if ( 2 =2).  compute LL3 = ylp&*ln(pt2)+(1-ylp)&*ln(1-pt2).  end if.  compute LL3 = -2*csum(LL3).  compute bt1 = make(ncol(xlp),1,0).  compute LL1 = 0.  compute pt1 = make(nrow(ylp),1,0.5).  compute pt1lp=pt1.  loop jjj = 1 to iterate.  compute vt1 = mdiag(pt1lp&*(1-pt1lp)).  compute b = bt1+inv(t(xlp)*vt1*xlp)*t(xlp)*(ylp-pt1lp).  do if ( 2 =2).  compute xlpb=xlp*b.  compute xlpbt=(xlpb > -709.7).  compute xlpb709=(1-xlpbt)*(-709.7).  compute xlpb=(xlpb&*xlpbt)+xlpb709.  compute pt1lp = 1/(1+exp(-(xlpb))).  end if.  compute itprob = csum((pt1lp < .00000001) or (pt1lp > .9999999)).  do if (itprob > 0).  loop kkk = 1 to nrow(pt1lp).  do if (pt1lp(kkk,1) > .9999999).  compute pt1lp(kkk,1) = .9999999.  end if.  do if (pt1lp(kkk,1) < .00000001).  compute pt1lp(kkk,1) = .00000001.  end if.  end loop.  compute itprob = 0.  end if.  do if (itprob = 0).  do if ( 2 =2).  compute LL = ylp&*ln(pt1lp)+(1-ylp)&*ln(1-pt1lp).  end if.  compute LL2 = -2*csum(ll).  end if.  do if (abs(LL1-LL2) < converge).  do if ( 0 =1).  compute vt1 = mdiag(pt1lp&*(1-pt1lp)).  compute varb = inv(t(xlp)*vt1*xlp).  compute seb = sqrt(diag(varb)).  end if.  break.  end if.  compute bt1 = b.  compute LL1 = LL2.  end loop.  compute modres=b.  do if (jjj > iterate).  compute itprob = 2.  do if (booting=0).  compute iterrmod=1.  end if.  do if (booting=1).  compute bootiter=1.  end if.  do if (itprobtg=0).  compute itprobtg=1.  compute errcode(errs,1) = 47.  compute errs = errs + 1.  do if (booting = 0 and 0 =1).  compute vt1 = mdiag(pt1lp&*(1-pt1lp)).  compute varb = inv(t(xlp)*vt1*xlp).  compute seb = sqrt(diag(varb)).  end if.  end if.  end if.  do if ( 0 =1).  compute trat = b&/seb.  compute dfres=nrow(xlp).  compute p = 2*(1-cdfnorm(abs(trat))).  compute modres={modres,seb,trat,p}.  compute modres={modres,(b-xp2&*seb),(b+xp2&*seb)}.  compute pvchi=1-chicdf((LL3-LL2),(nrow(modres)-1)).  compute mcF = (LL3-LL2)/LL3.  compute cox = 1-exp(-(LL3-LL2)/nrow(xlp)).  compute nagel = cox/(1-exp(-(LL3)/nrow(xlp))).  compute modsum={LL2,(LL3-LL2),(nrow(modres)-1),pvchi, mcF,cox,nagel}.  compute modsuml={'-2LL','ModelLL', 'df', 'p', 'McFadden', 'CoxSnell', 'Nagelkrk'}.  compute modresl={'coeff','se','Z','p','LLCI','ULCI'}.  end if.  end if  .  end if.  compute modres2={modres2,t(modres)}.  do if ( (bcmat((i+1),1) = 1) and (nobootx=1) and (effsize=1)).  compute nobootx=0.  compute xsdtemp= (nrow(x)*sscp(x(:,2)))-(t(csum(x(:,2)))*(csum(x(:,2)))).  compute xsdtemp= sqrt(xsdtemp/(nrow(x)*(nrow(x)-1))).  compute bootxsd={bootxsd;xsdtemp}.  end if.  end if.  end loop.  do if (bad = 0).  compute bootres={bootres;modres2(:,2:ncol(modres2))}.  do if (effsize=1).  compute ysdtemp= (nrow(y)*sscp(y))-(t(csum(y))*(csum(y))).  compute ysdtemp= sqrt(ysdtemp/(nrow(y)*(nrow(y)-1))).  compute bootysd={bootysd;ysdtemp}.  end if.  compute goodboot=goodboot+1.  end if.  do if (bad <> 0).  compute badboot=badboot+1.  end if.  end loop if (goodboot = boot).  compute bootres=bootres(2:nrow(bootres),:).  do if (effsize=1).  compute bootysd=bootysd(2:nrow(bootysd),:).  do if (nrow(bootxsd) > 1).  compute bootxsd=bootxsd(2:nrow(bootxsd),:).  end if.  end if.  do if (goodboot < (boot)).  compute boot=0.  compute modelbt=0.  compute notecode(notes,1) = 7.  compute notes = notes + 1.  end if.  do if (boot > 0).  do if (effsize=1).  compute bootysd={ysd;bootysd}.  compute bootxsd={xsd;bootxsd}.  end if.  do if (saveboot = 1).  save bootres/outfile = *.  end if.  compute bootcim=make(ncol(bootres),5,-99999).  compute bootcim(:,2) = t(csum(bootres)/nrow(bootres)).  compute bootcim(:,1) = coeffmat(2:nrow(coeffmat),1).  loop i = 1 to ncol(bootres).  .  compute temp = bootres(:,i).  compute temp(GRADE( bootres(:,i) )) = bootres(:,i).  compute badlo = 0.  compute badhi = 0.  do if ( 9999 <> 9999).  compute pv=csum(temp < 9999 )/nrow(temp).  compute ppv = pv.  do if (pv > .5).  compute ppv = 1-pv.  end if.  compute y5=sqrt(-2*ln(ppv)).  compute xp=y5+((((y5*p4+p3)*y5+p2)*y5+p1)*y5+p0)/((((y5*q4+q3)*y5+q2)*y5+q1)*y5+q0).  do if (pv <= .5).  compute xp = -xp.  end if.  compute cilow=rnd(nrow(temp)*(cdfnorm(2*xp+xp2))).  compute cihigh=trunc(nrow(temp)*(cdfnorm(2*xp+(-xp2))))+1.  do if (cilow < 1).  compute cilow = 1.  compute booterr=1.  compute badlo = 1.  end if.  do if (cihigh > nrow(temp)).  compute cihigh = boot.  compute booterr=1.  compute badhi = 1.  end if.  compute llcit=temp(cilow,1).  compute ulcit=temp(cihigh,1).  end if.  do if ( 9999 = 9999).  compute llcit=temp(cilow,1).  compute ulcit=temp(cihigh,1).  end if.  compute bootse=t(sqrt((cssq(temp)-((csum(temp)&**2)/nrow(temp)))/(nrow(temp)-1)))  .  compute bootcim(i,4:5)={llcit,ulcit}.  compute bootcim(i,3)=bootse.  end loop.  end if.  do if (badboot > 0).  compute notecode(notes,1) = 6.  compute notes = notes + 1.  end if.  end if.  do if (criterr=0).  release fulldat,x,y.  end if.  .  do if (criterr = 0 and nms > 0).  compute paths=paths(:,2:ncol(paths)).  compute pathsw=pathsw(:,2:ncol(pathsw)).  compute pathsz=pathsz(:,2:ncol(pathsz)).  compute pathswz=pathswz(:,2:ncol(pathswz)).  compute pathsmod=pathsw+pathsz+pathswz.  compute pathsdv=pathsdv(:,2:ncol(pathsdv)).  compute pathsfoc=pathsfoc(:,2:ncol(pathsfoc)).  compute pathtype=pathtype(:,2:ncol(pathtype)).  compute anymod=(rsum(pathsmod) > 0).  compute obscoeff=obscoeff(1,2:ncol(obscoeff)).  do if (dototal = 0).  print/title = '****************** DIRECT AND INDIRECT EFFECTS OF X ON Y *****************'.  end if.  do if (dototal = 1).  print/title = '************** TOTAL, DIRECT, AND INDIRECT EFFECTS OF X ON Y **************'.  compute totefflb(1,1)='Effect'.  do if (counterf <> 1).  do if (effsize=1).  compute toteffsz=toteff(:,1)/ysd.  compute totefflb={totefflb,'c_ps'}.  do if (xdich = 0 and mcx = 0).  compute toteffsz={toteffsz, (toteffsz*xsd)}.  compute totefflb={totefflb,'c_cs'}.  end if.  compute toteff={toteff,toteffsz}.  end if.  do if (nxvls > 1).  print toteff/title='Relative total effects of X on Y:'/rnames=toteffl2/cnames=totefflb/format= F10.4.  compute clabtmp={'R2-chng', hcflab, 'df1','df2','p'}.  print totomni/title='Omnibus test of total effect of X on Y:'/cnames=clabtmp/format= F10.4.  print/title= '----------'/space=0.  else.  print toteff/title='Total effect of X on Y'/cnames=totefflb/format= F10.4.  end if.  end if.  end if.  compute moddir=wcmat(nrow(bcmat),1)+zcmat(nrow(bcmat),1).  do if (bcmat(nrow(bcmat),1)=1 and counterf <> 1).  do if (ydich=1).  compute direfflb(:,(ncol(direfflb)-5):ncol(direfflb))={'Effect','se','Z','p','LLCI','ULCI'}.  end if.  do if (moddir=0).  compute direfflb(1,1)='Effect'.  end if.  do if (effsize=1 and moddir=0 and anymod = 0).  compute direffsz=direff(:,1)/ysd.  compute direfflb={direfflb,'c''_ps'}.  do if (xdich = 0 and mcx = 0).  compute direffsz={direffsz, (direffsz*xsd)}.  compute direfflb={direfflb,'c''_cs'}.  end if.  compute direff={direff,direffsz}.  end if.  do if (moddir = 0 and nxvls=1).  print direff/title='Direct effect of X on Y'/cnames=direfflb/format= F10.4.  end if.  do if (moddir = 0 and nxvls>1).  print direff/title='Relative direct effects of X on Y'/rnames=direffl2/cnames=direfflb/format= F10.4.  do if (ydich = 0).  compute clabtmp={'R2-chng', hcflab, 'df1','df2','p'}.  print diromni/title='Omnibus test of direct effect of X on Y:'/cnames=clabtmp/format= F10.4.  end if.  do if (ydich = 1).  compute clabtmp={'Chi-sq', 'df', 'p'}.  print diromni/title='Omnibus likelihood ratio test of direct effect of X on Y:'/cnames=clabtmp/format= F10.4.  end if.  print/title= '----------'/space=0.  end if.  do if (moddir > 0 and nxvls=1).  print direff/title='Conditional direct effect(s) of X on Y:'/cnames=direfflb/format= F10.4.  end if.  do if (moddir > 0 and nxvls>1).  compute direffl2={' '}.  loop i = 1 to nxvls.  loop j = 1 to (nrow(direff)/nxvls).  compute direffl2={direffl2;xcatlab(i,1)}.  end loop.  end loop.  compute direffl2=direffl2(2:nrow(direffl2),1).  print direff/title='Relative conditional direct effect(s) of X on Y:'/cnames=direfflb/rnames=direffl2/format= F10.4.  end if.  end if.  do if (bcmat(nrow(bcmat),1)=0 and counterf <> 1).  print/title='The direct effect of X on Y is fixed to zero.'.  end if.  do if (nms = 1).  compute indmark={2}.  compute indsets={1,2}.  end if.  do if (nms = 2).  compute indmark={2,2,3}.  compute indsets={1,4,2,5,1,3,5}.  compute thetam=1.  end if.  do if (nms = 3).  compute indmark={2,2,2,3,3,3,4}.  compute indsets={1,7,2,8,4,9,1,3,8,1,5,9,2,6,9,1,3,6,9}.  compute thetam={1,2,3}.  end if.  do if (nms = 4).  compute indmark={2,2,2,2,3,3,3,3,3,3,4,4,4,4,5}.  compute indsets={1,11,2,12,4,13,7,14,1,3,12,1,5,13,1,8,14,2,6,13,2,9,14,4,10,14,1,3,6,13,1,3,9, 14,1,5,10,14,2,6,10,14,1,3,6,10,14}.  compute thetam={1,2,4,3,5,6}.  end if.  do if (nms = 5).  compute indmark={2,2,2,2,2,3,3,3,3,3,3,3,3,3,3,4,4,4,4,4,4,4,4,4,4,5,5,5,5,5,6}.  compute indsets={1,16,2,17,4,18,7,19,11,20,1,3,17,1,5,18,1,8,19,1,12,20,2,6,18,2,9,19,2,13,20,4, 10,19,4,14,20,7,15,20,1,3,6,18, 1,3,9,19,1,3,13,20,1,5,10,19,1,5,14,20,1,8,15,20,2,6,10,19,2,6, 14,20,2,9,15,20,4,10,15,20,1,3,6,10,19, 1,3,6,14,20,1,3,9,15,20,1,5,10,15,20,2,6,10,15,20,1,3,6,10,15, 20}.  compute thetam={1,2,5,3,6,8,4,7,9,10}.  end if.  do if (nms = 6).  compute indmark={2,2,2,2,2,2,3,3,3,3,3,3,3,3,3,3,3,3,3,3,3,4,4,4,4,4,4,4,4,4,4,4,4,4,4,4,4,4,4, 4,4,5,5,5,5,5,5,5,5,5,5,5,5,5,5,5,6,6,6,6,6,6,7}.  compute indsets={1,22,2,23,4,24,7,25,11,26,16,27,1,3,23,1,5,24,1,8,25,1,12,26,1,17,27,2,6,24,2, 9,25,2,13,26,2,18,27,4,10,25,4,14,26, 4,19,27,7,15,26,7,20,27,11,21,27,1,3,6,24,1,3,9,25,1,3,13,26,1, 3,18,27,1,5,10,25,1,5,14,26,1,5,19,27,1,8,15,26,1,8,20,27, 1,12,21,27,2,6,10,25,2,6,14,26,2,6,19,27,2,9,15,26,2,9,20,27,2, 13,21,27,4,10,15,26,4,10,20,27,4,14,21,27,7,15,21,27, 1,3,6,10,25,1,3,6,14,26,1,3,6,19,27,1,3,9,15,26,1,3,9,20,27,1,3, 13,21,27,1,5,10,15,26,1,5,10,20,27,1,5,14,21,27, 1,8,15,21,27,2,6,10,15,26,2,6,10,20,27,2,6,14,21,27,2,9,15,21, 27,4,10,15,21,27,1,3,6,10,15,26,1,3,6,10,20,27, 1,3,6,14,21,27,1,3,9,15,21,27,1,5,10,15,21,27,2,6,10,15,21,27, 1,3,6,10,15,21,27}.  compute thetam={1,2,6,3,7,10,4,8,11,13,5,9,12,14,15}.  end if.  do if (nms = 7).  compute indmark={2,2,2,2,2,2,2}.  compute indsets={1,29,2,30,4,31,7,32,11,33,16,34,22,35}.  end if.  do if (nms = 8).  compute indmark={2,2,2,2,2,2,2,2}.  compute indsets={1,37,2,38,4,39,7,40,11,41,16,42,22,43,29,44}.  end if.  do if (nms = 9).  compute indmark={2,2,2,2,2,2,2,2,2}.  compute indsets={1,46,2,47,4,48,7,49,11,50,16,51,22,52,29,53,37,54}.  end if.  do if (nms=10).  compute indmark={2,2,2,2,2,2,2,2,2,2}.  compute indsets={1,56,2,57,4,58,7,59,11,60,16,61,22,62,29,63,37,64,46,65}.  end if.  compute indlbl = {'Ind1'; 'Ind2'; 'Ind3'; 'Ind4'; 'Ind5'; 'Ind6'; 'Ind7'; 'Ind8'; 'Ind9'; 'Ind10'; 'Ind11'; 'Ind12'; 'Ind13'; 'Ind14'; 'Ind15'}.  compute indlbl = {indlbl; 'Ind16';'Ind17';'Ind18';'Ind19';'Ind20';'Ind21';'Ind22';'Ind23';'Ind24';'Ind25';'Ind26';'Ind27';'Ind28';'Ind29';'Ind30'}.  compute indlbl = {indlbl; 'Ind31';'Ind32';'Ind33';'Ind34';'Ind35';'Ind36';'Ind37';'Ind38';'Ind39';'Ind40';'Ind41';'Ind42';'Ind43';'Ind44';'Ind45'}.  compute indlbl = {indlbl; 'Ind46';'Ind47';'Ind48';'Ind49';'Ind50';'Ind51';'Ind52';'Ind53';'Ind54';'Ind55';'Ind56';'Ind57';'Ind58';'Ind59';'Ind60'}.  compute indlbl = {indlbl; 'Ind61';'Ind62';'Ind63'}.  compute cntname={'(C1)';'(C2)';'(C3)';'(C4)';'(C5)';'(C6)';'(C7)';'(C8)';'(C9)';'(C10)';'(C11)';'(C12)';'(C13)';'(C14)';'(C15)';'(C16)';'(C17)'}.  compute cntname={cntname;'(C18)';'(C19)';'(C20)';'(C21)';'(C22)';'(C23)';'(C24)';'(C25)';'(C26)';'(C27)';'(C28)';'(C29)';'(C30)';'(C31)'}.  compute cntname={cntname;'(C32)';'(C33)';'(C34)';'(C35)';'(C36)';'(C37)';'(C38)';'(C39)';'(C40)';'(C41)';'(C42)';'(C43)';'(C44)';'(C45)'}.  compute cntname={cntname;'(C46)';'(C47)';'(C48)';'(C49)';'(C50)';'(C51)';'(C52)';'(C53)';'(C54)';'(C55)';'(C56)';'(C57)';'(C58)';'(C59)'}.  compute cntname={cntname;'(C60)';'(C61)';'(C62)';'(C63)';'(C64)';'(C65)';'(C66)';'(C67)';'(C68)';'(C69)';'(C70)';'(C71)';'(C72)';'(C73)'}.  compute cntname={cntname;'(C74)';'(C75)';'(C76)';'(C77)';'(C78)';'(C79)';'(C80)';'(C81)';'(C82)';'(C83)';'(C84)';'(C85)';'(C86)';'(C87)'}.  compute cntname={cntname;'(C88)';'(C89)';'(C90)';'(C91)';'(C92)';'(C93)';'(C94)';'(C95)';'(C96)';'(C97)';'(C98)';'(C99)';'(C100)';'(C101)'}.  compute cntname={cntname;'(C102)';'(C103)';'(C104)';'(C105)'}.  compute indmake=make(ncol(indmark),(nms+2),0).  compute indmod=make(ncol(indmark),1,999).  compute indmmm=make(ncol(indmark),1,0).  compute indmmmt=make(ncol(indmark),1,0).  compute start=1.  compute end=0.  compute nindfx=0.  compute indlocs=make(nrow(thetaxmb),ncol(paths),999).  compute indkey=make(ncol(indmark),1+((rmax(indmark)*2)+1),' ').  compute c1=1.  compute c2=1.  compute c3=1.  loop i = 1 to ncol(paths).  do if (pathtype(1,i)=1).  compute indlocs(:,i)=thetaxmb(:,c1).  compute c1=c1+1.  end if.  do if (pathtype(1,i)=3).  compute indlocs(:,i)=thetamyb(:,c2).  compute c2=c2+1.  end if.  do if (pathtype(1,i)=2 and nms < 7 and serial=1).  compute indlocs(:,i)=thetammb(:,thetam(1,c3)).  compute c3=c3+1.  end if.  end loop.  loop i = 1 to ncol(indlocs).  compute c1=2.  loop j = 2 to nrow(indlocs).  do if (indlocs(j,i) <> 0).  compute indlocs(c1,i)=indlocs(j,i).  compute c1=c1+1.  end if.  end loop.  compute indlocs(1,i)=c1-2.  end loop.  compute indlocs=indlocs(1:rmax((indlocs(1,:))+1),:).  loop i = 1 to ncol(indmark).  compute numget=indmark(1,i).  compute end=end+numget.  compute gotcha=indsets(1,start:end).  compute start=end+1.  compute ok=1.  compute temp=0.  compute repoman=make(4,1,0).  loop j = 1 to ncol(gotcha).  do if paths(1,gotcha(1,j))=0.  compute ok=0.  end if.  do if (pathsmod(1,gotcha(1,j)) > 0).  compute temp=1.  compute temp2={pathsw(1,gotcha(1,j));pathsz(1,gotcha(1,j));pathswz(1,gotcha(1,j));0}.  do if (temp2(1,1)=1 and temp2(2,1)=1 and temp2(3,1)=0).  compute temp2(4,1)=1.  end if.  compute repoman=repoman+temp2.  end if.  end loop.  compute temp=0.  compute tempmmm=0.  compute typemmm=0.  do if ((repoman(1,1) > 0) and (repoman(2,1) = 0)).  compute temp=1.  do if (repoman(1,1)=1).  compute tempmmm=1.  end if.  do if (repoman(1,1) > 1 and (wdich=1 or mcw > 0)).  compute tempmmm=12.  compute typemmm=mcw.  do if (wdich=1).  compute typemmm=1.  end if.  end if.  do if (repoman(1,1) > 1 and (wdich=0 and mcw = 0)).  compute tempmmm=101.  end if.  end if.  do if ((repoman(1,1) = 0) and (repoman(2,1) > 0)).  compute temp=2.  do if (repoman(2,1)=1).  compute tempmmm=2.  end if.  do if (repoman(2,1) > 1 and (zdich = 1 or mcz > 0)).  compute tempmmm=22.  compute typemmm=mcz.  do if (zdich=1).  compute typemmm=1.  end if.  end if.  do if (repoman(2,1) > 1 and (zdich = 0 and mcw = 0)).  compute tempmmm=102.  end if.  end if.  do if (repoman(1,1)>0 and repoman(2,1)>0).  compute temp=3.  do if (repoman(1,1)=1 and repoman(2,1)=1).  do if (repoman(4,1)=1).  compute tempmmm=31.  end if.  do if (repoman(3,1)=1).  compute tempmmm=41.  end if.  end if.  end if.  do if (repoman(1,1)=1 and repoman(2,1)=1 and repoman(3,1)=0 and repoman(4,1)=0).  compute tempmmm=51.  end if.  do if (ok = 1).  compute nindfx=nindfx+1.  compute indmake(nindfx,1)=numget.  compute indmod(nindfx,1)=temp.  compute indmmm(nindfx,1)=tempmmm.  compute indmmmt(nindfx,1)=typemmm.  compute indmake(nindfx,2:(1+numget))=gotcha.  compute indkey(nindfx,1)=xnames.  loop j = 1 to numget.  compute indkey(nindfx,(j*2+1))=pathsdv(1,gotcha(1,j)).  compute indkey(nindfx,(j*2))=' -> '.  end loop.  end if.  end loop.  compute indkey=indkey(1:nindfx,1:((cmax(indmake(:,1))*2)+1)).  compute indmake=indmake(1:nindfx,1:(cmax(indmake(:,1))+1)).  compute indmod=indmod(1:nrow(indmake),1).  compute indmmm=indmmm(1:nrow(indmake),1).  compute indmmmt=indmmmt(1:nrow(indmake),1).  compute ncpairs = (((nindfx)*(nindfx-1))/2).  do if ((contrast = 1 or contrast = 2) and (ncpairs > 105)).  compute contrast=0.  compute notecode(notes,1) = 13.  compute notes = notes + 1.  end if.  do if (contrast = 3).  do if (ncol(contvec) <> nindfx).  compute contrast=0.  compute notecode(notes,1) = 14.  compute notes = notes + 1.  end if.  end if.  do if (anymod=0).  do if (nms = 1 and contrast > 0).  compute contrast=0.  end if.  compute efloop=(((1-(effsize=0))*2)+1)-(((mcx>0 or xdich=1))*(1-(effsize=0))).  loop kk=1 to efloop.  do if (boot = 0).  compute bootres=obscoeff.  compute indtab=999.  compute inddiff=999.  compute bootysd=ysd.  compute bootxsd=xsd.  end if.  do if (boot > 0).  compute bootres={obscoeff;bootres}.  compute indtab=make(1,4,999).  compute inddiff=make(nrow(bootres),1,999).  end if.  compute indtotal=make(nrow(bootres),1,0).  loop i = 1 to nrow(indmake).  loop j = 1 to nxvls.  compute indtemp=make(nrow(bootres),1,1).  loop k = 1 to indmake(i,1).  compute jtemp=1.  do if (j > 1 and k=1).  compute jtemp=j.  end if.  compute indtemp=indtemp&*bootres(:,pathsfoc(jtemp,indmake(i,(k+1)))).  end loop.  do if (kk = 2).  compute indtemp=indtemp/bootysd.  end if.  do if (kk = 3).  compute indtemp=(bootxsd&*indtemp)/bootysd.  end if.  do if (contrast <> 0).  compute inddiff={inddiff,indtemp}.  end if.  do if (nxvls=1).  compute indtotal=indtotal+indtemp.  end if.  compute indeff=indtemp(1,1).  do if (boot > 0).  .  compute temp = indtemp(2:nrow(indtemp),1).  compute temp(GRADE( indtemp(2:nrow(indtemp),1) )) = indtemp(2:nrow(indtemp),1).  compute badlo = 0.  compute badhi = 0.  do if ( 9999 <> 9999).  compute pv=csum(temp < 9999 )/nrow(temp).  compute ppv = pv.  do if (pv > .5).  compute ppv = 1-pv.  end if.  compute y5=sqrt(-2*ln(ppv)).  compute xp=y5+((((y5*p4+p3)*y5+p2)*y5+p1)*y5+p0)/((((y5*q4+q3)*y5+q2)*y5+q1)*y5+q0).  do if (pv <= .5).  compute xp = -xp.  end if.  compute cilow=rnd(nrow(temp)*(cdfnorm(2*xp+xp2))).  compute cihigh=trunc(nrow(temp)*(cdfnorm(2*xp+(-xp2))))+1.  do if (cilow < 1).  compute cilow = 1.  compute booterr=1.  compute badlo = 1.  end if.  do if (cihigh > nrow(temp)).  compute cihigh = boot.  compute booterr=1.  compute badhi = 1.  end if.  compute llcit=temp(cilow,1).  compute ulcit=temp(cihigh,1).  end if.  do if ( 9999 = 9999).  compute llcit=temp(cilow,1).  compute ulcit=temp(cihigh,1).  end if.  compute bootse=t(sqrt((cssq(temp)-((csum(temp)&**2)/nrow(temp)))/(nrow(temp)-1)))  .  compute indeff={indeff,bootse,llcit,ulcit}.  end if.  compute indtab={indtab;indeff}.  end loop.  end loop.  compute indtab=indtab(2:nrow(indtab),:).  compute rowlbs=indlbl(1:nrow(indtab),1).  do if (mc > 0).  compute inddiff=make(mc,1,-999).  compute indtab2=make(nrow(indtab),4,-999).  compute indtab2(:,1)=indtab.  compute indtab=indtab2.  release indtab2.  compute mcct=0.  compute indtotal=make(mc,1,0).  do if (kk = 1).  compute x1 = sqrt(-2*ln(uniform(mc,nrow(mcsopath))))&*cos((2*3.14159265358979)*uniform(mc, nrow(mcsopath))).  compute x1=x1*chol(indcov).  loop ii=1 to nrow(x1).  compute x1(ii,:)=x1(ii,:)+t(mcsopath).  end loop.  end if.  loop ii=1 to nms.  compute tmpb=x1(:,((nms*nxvls)+ii)).  compute tmpb2=tmpb.  do if (nxvls > 1).  loop jj=1 to (nxvls-1).  compute tmpb2={tmpb2,tmpb}.  end loop.  end if.  compute indtemp=x1(:,(((ii-1)*nxvls)+1):(ii*nxvls))&*tmpb2.  loop jj=1 to ncol(indtemp).  do if (kk = 2).  compute indtemp(:,jj)=indtemp(:,jj)/ysd.  end if.  do if (kk = 3).  compute indtemp(:,jj)=(xsd*indtemp(:,jj))/ysd.  end if.  .  compute temp = indtemp(:,jj).  compute temp(GRADE( indtemp(:,jj) )) = indtemp(:,jj).  compute badlo = 0.  compute badhi = 0.  do if ( 9999 <> 9999).  compute pv=csum(temp < 9999 )/nrow(temp).  compute ppv = pv.  do if (pv > .5).  compute ppv = 1-pv.  end if.  compute y5=sqrt(-2*ln(ppv)).  compute xp=y5+((((y5*p4+p3)*y5+p2)*y5+p1)*y5+p0)/((((y5*q4+q3)*y5+q2)*y5+q1)*y5+q0).  do if (pv <= .5).  compute xp = -xp.  end if.  compute cilow=rnd(nrow(temp)*(cdfnorm(2*xp+xp2))).  compute cihigh=trunc(nrow(temp)*(cdfnorm(2*xp+(-xp2))))+1.  do if (cilow < 1).  compute cilow = 1.  compute booterr=1.  compute badlo = 1.  end if.  do if (cihigh > nrow(temp)).  compute cihigh = boot.  compute booterr=1.  compute badhi = 1.  end if.  compute llcit=temp(cilow,1).  compute ulcit=temp(cihigh,1).  end if.  do if ( 9999 = 9999).  compute llcit=temp(cilow,1).  compute ulcit=temp(cihigh,1).  end if.  compute bootse=t(sqrt((cssq(temp)-((csum(temp)&**2)/nrow(temp)))/(nrow(temp)-1)))  .  compute mcct=mcct+1.  compute indtab(mcct,2:4)={bootse,llcit,ulcit}.  end loop.  do if (nxvls=1).  compute indtotal=indtotal+indtemp.  do if (contrast <> 0).  compute inddiff={inddiff,indtemp}.  end if.  end if.  end loop.  release indtemp,tmpb.  end if.  do if (normal = 1 and sobelok=1).  compute sobelmat=indtab(:,1).  compute sobelmat={sobelmat,(sobelmat/2),sobelmat,sobelmat}.  loop ii=1 to nms.  compute se2b=indcov(((nms*nxvls)+ii),((nms*nxvls)+ii)).  compute bpath2=mcsopath(((nms*nxvls)+ii),1)&**2.  compute se2a=diag(indcov((((ii-1)*nxvls)+1):(ii*nxvls),(((ii-1)*nxvls)+1):(ii*nxvls))).  compute apath2=mcsopath((((ii-1)*nxvls)+1):(ii*nxvls) ,1)&**2.  compute sesobel=sqrt(apath2*se2b+bpath2*se2a+se2a*se2b).  compute sobelmat((((ii-1)*nxvls)+1):(ii*nxvls),2)=sesobel.  end loop.  release se2b,bpath2,se2a,apath2,sesobel,ii.  compute sobelmat(:,3)=sobelmat(:,1)&/sobelmat(:,2).  compute sobelmat(:,4) = 2*(1-cdfnorm(abs(sobelmat(:,3)))).  end if.  do if (serial = 0).  compute rowlbs=t(mnames).  end if.  do if (nxvls=1 and nms > 1).  compute rowlbs={'TOTAL';rowlbs}.  compute indtemp=indtotal(1,1).  do if (boot > 0 and nxvls=1).  .  compute temp = indtotal(2:nrow(indtotal),1).  compute temp(GRADE( indtotal(2:nrow(indtotal),1) )) = indtotal(2:nrow(indtotal),1).  compute badlo = 0.  compute badhi = 0.  do if ( 9999 <> 9999).  compute pv=csum(temp < 9999 )/nrow(temp).  compute ppv = pv.  do if (pv > .5).  compute ppv = 1-pv.  end if.  compute y5=sqrt(-2*ln(ppv)).  compute xp=y5+((((y5*p4+p3)*y5+p2)*y5+p1)*y5+p0)/((((y5*q4+q3)*y5+q2)*y5+q1)*y5+q0).  do if (pv <= .5).  compute xp = -xp.  end if.  compute cilow=rnd(nrow(temp)*(cdfnorm(2*xp+xp2))).  compute cihigh=trunc(nrow(temp)*(cdfnorm(2*xp+(-xp2))))+1.  do if (cilow < 1).  compute cilow = 1.  compute booterr=1.  compute badlo = 1.  end if.  do if (cihigh > nrow(temp)).  compute cihigh = boot.  compute booterr=1.  compute badhi = 1.  end if.  compute llcit=temp(cilow,1).  compute ulcit=temp(cihigh,1).  end if.  do if ( 9999 = 9999).  compute llcit=temp(cilow,1).  compute ulcit=temp(cihigh,1).  end if.  compute bootse=t(sqrt((cssq(temp)-((csum(temp)&**2)/nrow(temp)))/(nrow(temp)-1)))  .  compute indtemp={indtemp, bootse,llcit,ulcit}.  end if.  do if (mc > 0).  compute obtmc=indtab(:,1).  compute indtemp=csum(obtmc).  .  compute temp = indtotal(:,1).  compute temp(GRADE( indtotal(:,1) )) = indtotal(:,1).  compute badlo = 0.  compute badhi = 0.  do if ( 9999 <> 9999).  compute pv=csum(temp < 9999 )/nrow(temp).  compute ppv = pv.  do if (pv > .5).  compute ppv = 1-pv.  end if.  compute y5=sqrt(-2*ln(ppv)).  compute xp=y5+((((y5*p4+p3)*y5+p2)*y5+p1)*y5+p0)/((((y5*q4+q3)*y5+q2)*y5+q1)*y5+q0).  do if (pv <= .5).  compute xp = -xp.  end if.  compute cilow=rnd(nrow(temp)*(cdfnorm(2*xp+xp2))).  compute cihigh=trunc(nrow(temp)*(cdfnorm(2*xp+(-xp2))))+1.  do if (cilow < 1).  compute cilow = 1.  compute booterr=1.  compute badlo = 1.  end if.  do if (cihigh > nrow(temp)).  compute cihigh = boot.  compute booterr=1.  compute badhi = 1.  end if.  compute llcit=temp(cilow,1).  compute ulcit=temp(cihigh,1).  end if.  do if ( 9999 = 9999).  compute llcit=temp(cilow,1).  compute ulcit=temp(cihigh,1).  end if.  compute bootse=t(sqrt((cssq(temp)-((csum(temp)&**2)/nrow(temp)))/(nrow(temp)-1)))  .  compute indtemp={indtemp, bootse,llcit,ulcit}.  end if.  compute indtab={indtemp;indtab}.  end if.  compute bootlbs={'Effect', 'BootSE','BootLLCI','BootULCI'}.  do if (mc > 0).  compute bootlbs={'Effect', 'MC SE','MC LLCI','MC ULCI'}.  end if.  do if (nxvls = 1).  do if (contrast <> 0).  compute inddiff=inddiff(:,2:ncol(inddiff)).  do if (mc > 0).  compute inddiff={t(obtmc);inddiff}.  end if.  do if (contrast = 3).  compute inddifft=inddiff*t(contvec).  compute indtemp=inddifft(1,1).  do if (boot > 0 or mc > 0).  .  compute temp = inddifft(2:nrow(inddifft),1).  compute temp(GRADE( inddifft(2:nrow(inddifft),1) )) = inddifft(2:nrow(inddifft),1).  compute badlo = 0.  compute badhi = 0.  do if ( 9999 <> 9999).  compute pv=csum(temp < 9999 )/nrow(temp).  compute ppv = pv.  do if (pv > .5).  compute ppv = 1-pv.  end if.  compute y5=sqrt(-2*ln(ppv)).  compute xp=y5+((((y5*p4+p3)*y5+p2)*y5+p1)*y5+p0)/((((y5*q4+q3)*y5+q2)*y5+q1)*y5+q0).  do if (pv <= .5).  compute xp = -xp.  end if.  compute cilow=rnd(nrow(temp)*(cdfnorm(2*xp+xp2))).  compute cihigh=trunc(nrow(temp)*(cdfnorm(2*xp+(-xp2))))+1.  do if (cilow < 1).  compute cilow = 1.  compute booterr=1.  compute badlo = 1.  end if.  do if (cihigh > nrow(temp)).  compute cihigh = boot.  compute booterr=1.  compute badhi = 1.  end if.  compute llcit=temp(cilow,1).  compute ulcit=temp(cihigh,1).  end if.  do if ( 9999 = 9999).  compute llcit=temp(cilow,1).  compute ulcit=temp(cihigh,1).  end if.  compute bootse=t(sqrt((cssq(temp)-((csum(temp)&**2)/nrow(temp)))/(nrow(temp)-1)))  .  compute indtemp={indtemp, bootse,llcit,ulcit}.  end if.  compute indtab={indtab;indtemp}.  end if.  do if (contrast = 1 or contrast = 2).  compute conkey=make(1,4,' ').  loop i = 1 to ncol(inddiff)-1.  loop j = (i+1) to ncol(inddiff).  compute inddifft=inddiff(:,i)-inddiff(:,j).  do if (contrast=2).  compute inddifft=abs(inddiff(:,i))-abs(inddiff(:,j)).  end if.  compute indtemp=inddifft(1,1).  compute conkeyt={' ', rowlbs((i+1),1),' minus ',rowlbs((j+1),1)}.  compute conkey={conkey;conkeyt}.  do if (boot > 0 or mc > 0).  .  compute temp = inddifft(2:nrow(inddifft),1).  compute temp(GRADE( inddifft(2:nrow(inddifft),1) )) = inddifft(2:nrow(inddifft),1).  compute badlo = 0.  compute badhi = 0.  do if ( 9999 <> 9999).  compute pv=csum(temp < 9999 )/nrow(temp).  compute ppv = pv.  do if (pv > .5).  compute ppv = 1-pv.  end if.  compute y5=sqrt(-2*ln(ppv)).  compute xp=y5+((((y5*p4+p3)*y5+p2)*y5+p1)*y5+p0)/((((y5*q4+q3)*y5+q2)*y5+q1)*y5+q0).  do if (pv <= .5).  compute xp = -xp.  end if.  compute cilow=rnd(nrow(temp)*(cdfnorm(2*xp+xp2))).  compute cihigh=trunc(nrow(temp)*(cdfnorm(2*xp+(-xp2))))+1.  do if (cilow < 1).  compute cilow = 1.  compute booterr=1.  compute badlo = 1.  end if.  do if (cihigh > nrow(temp)).  compute cihigh = boot.  compute booterr=1.  compute badhi = 1.  end if.  compute llcit=temp(cilow,1).  compute ulcit=temp(cihigh,1).  end if.  do if ( 9999 = 9999).  compute llcit=temp(cilow,1).  compute ulcit=temp(cihigh,1).  end if.  compute bootse=t(sqrt((cssq(temp)-((csum(temp)&**2)/nrow(temp)))/(nrow(temp)-1)))  .  compute indtemp={indtemp, bootse,llcit,ulcit}.  end if.  compute indtab={indtab;indtemp}.  end loop.  end loop.  end if.  release inddiff.  compute contlbs=cntname(1:(((nindfx)*(nindfx-1))/2),1).  compute rowlbs={rowlbs;contlbs}.  end if.  do if (kk=1).  print indtab/title = 'Indirect effect(s) of X on Y:'/rnames=rowlbs/cnames=bootlbs/format= F10.4.  end if.  do if (kk = 2).  print indtab/title = 'Partially standardized indirect effect(s) of X on Y:'/rnames=rowlbs/cnames=bootlbs/format= F10.4.  end if.  do if (kk = 3).  print indtab/title = 'Completely standardized indirect effect(s) of X on Y:'/rnames=rowlbs/cnames=bootlbs/format= F10.4.  end if.  do if (normal=1 and sobelok=1 and kk=1).  compute sobellab={'Effect',hclab,'Z','p'}.  compute sobelrlb=rowlbs.  do if (nms > 1).  compute sobelrlb=rowlbs(2:(1+nms),1).  end if.  print sobelmat/title=' Normal theory test for indirect effect(s):'/cnames=sobellab/rnames=sobelrlb/format= F10.4.  end if.  do if (contrast <> 0).  do if ((contrast=1 or contrast = 2) and kk=efloop ).  compute conkey=conkey(2:nrow(conkey),:).  print conkey/title = 'Specific indirect effect contrast definition(s):'/rnames=contlbs/format=A8.  end if.  do if (contrast = 3 and kk=efloop).  compute crowlbs=rowlbs(2:(nindfx+1),1).  print contvec/title = 'Specific indirect effect contrast weights:'/cnames=crowlbs/rlabels='(C1)'/format= F10.4.  end if.  do if (contrast = 2 and kk=efloop).  print/title = 'Contrasts are differences between absolute values of indirect effects'.  end if.  end if.  do if (serial = 1 and kk=efloop).  compute rowlbst=rowlbs(2:nrow(rowlbs),1).  print indkey/title = 'Indirect effect key:'/rnames=rowlbst/format = A8.  end if.  else.  do if (kk = 1).  print/title = 'Relative indirect effects of X on Y'.  end if.  do if (kk = 2).  print/title = 'Partially standardized relative indirect effect(s) of X on Y:'.  end if.  do if (kk = 3).  print/title = 'Completely standardized relative indirect effect(s) of X on Y:'.  end if.  loop i = 1 to nrow(indmake).  compute indtabsm=indtab((((i-1)*nxvls)+1):(nxvls*i),:).  compute indkeyt=indkey(i,:).  print indkeyt/title=' '/space=0/format=A8.  do if (bcmat(nrow(bcmat),1)=0)).  compute direffl2=xcatlab(1:nxvls,1).  end if.  print indtabsm/title = ' '/cnames=bootlbs/rnames=direffl2/format= F10.4 /space=0.  do if (normal=1 and sobelok=1 and kk=1).  compute sobelsm=sobelmat((((i-1)*nxvls)+1):(nxvls*i),:).  compute sobellab={'Effect',hclab,'Z','p'}.  print sobelsm/title=' Normal theory test for relative indirect effects:'/cnames=sobellab/rnames=direffl2/format= F10.4.  end if.  end loop.  end if.  do if (effsize = 1 and boot > 0).  compute bootres=bootres(2:nrow(bootres),:).  end if.  end loop.  end if.  do if (anymod > 0).  do if (boot = 0).  compute bootres=obscoeff.  compute indtab=999.  end if.  do if (boot > 0).  compute bootres={obscoeff;bootres}.  compute indtab=make(1,4,999).  end if.  do if (csum((indmod > 0))=nrow(indmod)).  do if (nxvls > 1).  print/title= 'Relative conditional indirect effects of X on Y:'.  end if.  do if (nxvls = 1).  print/title= 'Conditional indirect effects of X on Y:'.  end if.  end if.  do if (csum((indmod > 0)) < nrow(indmod)).  do if (nxvls > 1).  print/title= 'Relative conditional and unconditional indirect effects of X on Y:'.  end if.  do if (nxvls = 1).  print/title= 'Conditional and unconditional indirect effects of X on Y:'.  end if.  end if.  loop i = 1 to nrow(indmake).  compute indtab=indtab(1,:)*0.  print indkey(i,:)/title = 'INDIRECT EFFECT:'/format=A8.  do if (indmod(i,1)=0).  loop j = 1 to nxvls.  compute indtemp=make(nrow(bootres),1,1).  loop k = 1 to indmake(i,1).  compute jtemp=1.  do if (j > 1 and k=1).  compute jtemp=j.  end if.  compute indtemp=indtemp&*bootres(:,pathsfoc(jtemp,indmake(i,(k+1)))).  end loop.  compute indeff=indtemp(1,1).  do if (boot > 0).  .  compute temp = indtemp(2:nrow(indtemp),1).  compute temp(GRADE( indtemp(2:nrow(indtemp),1) )) = indtemp(2:nrow(indtemp),1).  compute badlo = 0.  compute badhi = 0.  do if ( 9999 <> 9999).  compute pv=csum(temp < 9999 )/nrow(temp).  compute ppv = pv.  do if (pv > .5).  compute ppv = 1-pv.  end if.  compute y5=sqrt(-2*ln(ppv)).  compute xp=y5+((((y5*p4+p3)*y5+p2)*y5+p1)*y5+p0)/((((y5*q4+q3)*y5+q2)*y5+q1)*y5+q0).  do if (pv <= .5).  compute xp = -xp.  end if.  compute cilow=rnd(nrow(temp)*(cdfnorm(2*xp+xp2))).  compute cihigh=trunc(nrow(temp)*(cdfnorm(2*xp+(-xp2))))+1.  do if (cilow < 1).  compute cilow = 1.  compute booterr=1.  compute badlo = 1.  end if.  do if (cihigh > nrow(temp)).  compute cihigh = boot.  compute booterr=1.  compute badhi = 1.  end if.  compute llcit=temp(cilow,1).  compute ulcit=temp(cihigh,1).  end if.  do if ( 9999 = 9999).  compute llcit=temp(cilow,1).  compute ulcit=temp(cihigh,1).  end if.  compute bootse=t(sqrt((cssq(temp)-((csum(temp)&**2)/nrow(temp)))/(nrow(temp)-1)))  .  compute indeff={indeff,bootse,llcit,ulcit}.  end if.  compute indtab={indtab;indeff}.  end loop.  do if (nxvls > 1).  compute indefflb=xcatlab(1:nxvls,1).  print indtab(2:nrow(indtab),:)/title = ' '/clabels='Effect', 'BootSE', 'BootLLCI', 'BootULCI'/rnames=indefflb/format= F10.4 /space=0.  end if.  do if (nxvls = 1).  print indtab(2:nrow(indtab),:)/title = ' '/clabels='Effect', 'BootSE', 'BootLLCI', 'BootULCI'/format= F10.4 /space=0.  end if.  end if.  do if (indmod(i,1)>0).  do if (indmod(i,1)=1).  compute indmodva=wmodvals.  compute indprova=wprobval.  compute condlbs={wnames}.  compute printw=1.  else if (indmod(i,1)=2).  compute indmodva=zmodvals.  compute indprova=zprobval.  compute condlbs={znames}.  compute printz=1.  else if (indmod(i,1)=3).  compute cntmp=1.  compute printz=1.  compute printw=1.  compute indmodva=make((nrow(wmodvals)*nrow(zmodvals)),2,999).  loop k7 = 1 to nrow(wmodvals).  loop k8 = 1 to nrow(zmodvals).  compute indmodva(cntmp,:)={wmodvals(k7,1),zmodvals(k8,1)}.  compute cntmp=cntmp+1.  end loop.  end loop.  compute condlbs={wnames,znames}.  end if.  compute condres=make(nrow(indmodva),1,999).  do if (boot > 0).  compute condres=make(nrow(indmodva),4,999).  end if.  compute condres={indmodva,condres}.  loop k4 = 1 to nxvls.  compute imm3=make(nrow(bootres),1,1).  compute imm4=make(nrow(bootres),1,1).  compute indcontr=0.  do if (indmod(i,1)=3).  compute tihsw=wprobval.  compute tihsz=zprobval.  end if.  loop k1=1 to nrow(indmodva).  compute tucker2=make(nrow(bootres),1,1).  compute imm2=make(nrow(bootres),1,1).  compute wfirst=0.  compute zfirst=0.  compute immset=0.  loop k2=1 to indmake(i,1).  compute colnumb=indmake(i,(k2+1)).  do if (k2=1).  compute wbb=make(nrow(bootres),(nwvls*nxvls),0).  compute zbb=make(nrow(bootres),(nzvls*nxvls),0).  compute wzbb=make(nrow(bootres),(nwvls*nzvls*nxvls),0).  end if.  do if (k2<>1).  compute wbb=make(nrow(bootres),nwvls,0).  compute zbb=make(nrow(bootres),nzvls,0).  compute wzbb=make(nrow(bootres),(nwvls*nzvls),0).  end if.  compute cnt=1.  compute tihs=indlocs(2:((indlocs(1,colnumb))+1),colnumb).  do if (k2 = 1).  compute focbb=tihs(1:nxvls,1).  compute focbb=bootres(:,focbb).  do if (indmmm(i,1)>0).  compute imm=focbb(:,k4).  compute condbb=imm.  end if.  compute focaddon=make(1,nxvls,0).  compute focaddon(1,k4)=1.  compute cnt=cnt+nxvls.  compute placeh=nxvls.  do if (indmod(i,1)=1).  compute tihsz=make(nrow(wprobval),(nzvls*nxvls),0).  compute tihswz=make(nrow(wprobval),(nwvls*nzvls*nxvls),0).  do if (pathsw(1,colnumb)=1).  compute temp=make(nrow(wprobval),(nxvls*nwvls),0).  loop k5 = 1 to nrow(wprobval).  loop k6=1 to nwvls.  compute temp(k5, (((k4-1)*nwvls)+k6))=wprobval(k5,k6).  end loop.  end loop.  compute indprova={temp,tihsz,tihswz}.  else.  compute indprova={wprobval,tihsz,tihswz}.  end if.  end if.  do if (indmod(i,1)=2).  compute tihsw=make(nrow(zprobval),(nwvls*nxvls),0).  compute tihswz=make(nrow(zprobval),(nwvls*nzvls*nxvls),0).  do if (pathsz(1,colnumb)=1).  compute temp=make(nrow(zprobval),(nxvls*nzvls),0).  loop k5 = 1 to nrow(zprobval).  loop k6 =1 to nzvls.  compute temp(k5,(((k4-1)*nzvls)+k6))=zprobval(k5,k6).  end loop.  end loop.  compute indprova={tihsw,temp,tihswz}.  else.  compute indprova={tihsw,zprobval,tihswz}.  end if.  end if.  do if (indmod(i,1)=3).  compute indprova=make((nrow(wprobval)*nrow(zprobval)),((ncol(wprobval)*nxvls)+ (ncol(zprobval)*nxvls)+(nwvls*nzvls*nxvls)),0).  compute cntemp=1.  loop k7=1 to nrow(wprobval).  loop k8 =1 to nrow(zprobval).  compute temp=wprobval(k7,:)*focaddon(1,k4).  compute indprova(cntemp,(((k4-1)*nwvls)+1):(k4*(nwvls)))=temp.  compute temp=zprobval(k8,:)*focaddon(1,k4).  compute indprova(cntemp, ((((k4-1)*nzvls)+1)+(nxvls*nwvls)) : ((((k4-1)*nzvls)+1)+(nxvls*nwvls)+(nzvls-1)))=temp.  compute cntemp=cntemp+1.  end loop.  end loop.  do if (pathsz(1,colnumb)=0).  compute temp=make(nrow(indprova),(ncol(zprobval)*nxvls),0).  compute indprova(:,((ncol(wprobval)*nxvls)+1):((ncol(wprobval)+ncol(zprobval))* nxvls))=temp.  end if.  do if (pathsw(1,colnumb)=0).  compute temp=make(nrow(indprova),(ncol(wprobval)*nxvls),0).  compute indprova(:,1:(ncol(wprobval)*nxvls))=temp.  end if.  do if (pathswz(1,colnumb)=1).  compute cntemp=(ncol(wprobval)*nxvls)+(ncol(zprobval)*nxvls)+((k4-1)* ncol(wprobval)*ncol(zprobval))+1.  loop k7=1 to ncol(wprobval).  loop k8=1 to ncol(zprobval).  compute indprova(:,cntemp)=indprova(:,((ncol(wprobval)*(k4-1))+k7))&* indprova(:,((((k4-1)*ncol(zprobval))+k8)+(nxvls*ncol(wprobval)))).  compute cntemp=cntemp+1.  end loop.  end loop.  end if.  end if.  end if.  do if (k2 > 1).  compute focbb=tihs(1,1).  compute focbb=bootres(:,focbb).  do if (indmmm(i,1)>0).  compute imm=focbb(:,1).  compute condbb=imm.  end if.  compute focaddon=1.  compute cnt=cnt+1.  compute placeh=1.  do if (indmod(i,1)=1).  compute tihsz=make(nrow(wprobval),nzvls,0).  compute tihswz=make(nrow(wprobval),(nwvls*nzvls),0).  compute indprova={wprobval,tihsz,tihswz}.  end if.  do if (indmod(i,1)=2).  compute tihsw=make(nrow(zprobval),nwvls,0).  compute tihswz=make(nrow(zprobval),(nwvls*nzvls),0).  compute indprova={tihsw,zprobval,tihswz}.  end if.  do if (indmod(i,1)=3).  compute indprova=make((nrow(wprobval)*nrow(zprobval)),((ncol(wprobval)+ ncol(zprobval))+(nwvls*nzvls)),0).  compute cntemp=1.  loop k7=1 to nrow(wprobval).  loop k8 =1 to nrow(zprobval).  compute indprova(cntemp,1:(ncol(wprobval)+ncol(zprobval)))={wprobval(k7,:), zprobval(k8,:)}.  compute cntemp=cntemp+1.  end loop.  end loop.  do if (pathsz(1,colnumb)=0).  compute temp=make(nrow(indprova),ncol(zprobval),0).  compute indprova(:,(ncol(wprobval)+1):(ncol(wprobval)+ncol(zprobval)))=temp.  end if.  do if (pathsw(1,colnumb)=0).  compute temp=make(nrow(indprova),ncol(wprobval),0).  compute indprova(:,1:ncol(wprobval))=temp.  end if.  do if (pathswz(1,colnumb)=1).  compute cntemp=ncol(wprobval)+ncol(zprobval)+1.  loop k7=1 to ncol(wprobval).  loop k8=1 to ncol(zprobval).  compute indprova(:,cntemp)=indprova(:,k7)&*indprova(:,(ncol(wprobval)+k8)).  compute cntemp=cntemp+1.  end loop.  end loop.  end if.  end if.  end if.  do if (pathsw(1,colnumb)) = 1.  compute wbb=tihs(cnt:(cnt+(placeh*nwvls)-1),1).  compute wbb=bootres(:,wbb).  compute immlbs2=wcatlab(1:nwvls,1).  do if (zfirst=0).  compute wfirst=1.  end if.  do if (indmmm(i,1)=1 or indmmm(i,1)=31 or indmmm(i,1)=51).  compute imm=wbb(:,1).  loop k7 = 1 to nwvls.  compute imm={imm,wbb(:,(((k4-1)*nwvls*(k2=1))+k7))}.  end loop.  compute imm=imm(:,2:ncol(imm)).  end if.  do if (indmmm(i,1)=41 or indmmm(i,1)=51).  compute condbb=make(nrow(bootres),1,0).  loop k7 = 1 to nwvls.  compute condbb={condbb,wbb(:,(((k4-1)*nwvls*(k2=1))+k7))}.  end loop.  compute condbb=condbb(:,2:ncol(condbb)).  end if.  compute cnt=cnt+(placeh*nwvls).  end if.  do if (pathsz(1,colnumb)) = 1.  compute zbb=tihs(cnt:(cnt+(placeh*nzvls)-1),1).  compute zbb=bootres(:,zbb).  do if (wfirst=0).  compute zfirst=1.  end if.  do if (indmmm(i,1) <> 31).  compute immlbs2=zcatlab(1:nzvls,1).  end if.  do if (indmmm(i,1)=2 or indmmm(i,1)=31 or indmmm(i,1)=51).  do if (indmmm(i,1)=2).  compute imm=zbb(:,1).  end if.  loop k7 = 1 to nzvls.  compute imm={imm,zbb(:,(((k4-1)*nzvls*(k2=1))+k7))}.  end loop.  do if (indmmm(i,1)=2 or indmmm(i,1)=51).  compute imm=imm(:,2:ncol(imm)).  do if (indmmm(i,1)=51).  compute condbb={condbb,imm}.  end if.  end if.  end if.  compute cnt=cnt+(placeh*nzvls).  end if.  do if (pathswz(1,colnumb)) = 1.  compute wzbb=tihs(cnt:(cnt+(placeh*nwvls*nzvls)-1),1).  compute wzbb=bootres(:,wzbb).  do if (indmmm(i,1)=41).  compute imm=wzbb(:,1).  loop k7=1 to nwvls*nzvls.  compute imm={imm,wzbb(:,(((k4-1)*nzvls*nwvls*(k2=1))+k7))}.  end loop.  end if.  do if (indmmm(i,1)=41).  compute imm=imm(:,2:ncol(imm)).  compute condbb={condbb,imm(:,(ncol(imm)-(nwvls*nzvls)+1):ncol(imm))}.  end if.  compute cnt=cnt+(placeh*nzvls*nwvls).  end if.  compute indprobe={focaddon,indprova(k1,:)}.  compute tucker={focbb,wbb,zbb,wzbb}.  loop k3=1 to ncol(indprobe).  compute tucker(:,k3)=tucker(:,k3)*indprobe(1,k3).  end loop.  compute tucker2=tucker2&*rsum(tucker).  do if (indmmm(i,1) = 1 or indmmm(i,1)=2 or indmmm(i,1)=31 or indmmm(i,1)=41 or indmmm(i,1)=51).  do if (immset=1).  do if (ncol(imm2)=1 and ncol(imm) = 1).  compute imm2=imm2&*imm.  end if.  do if (indmmm(i,1)=41 or indmmm(i,1)=51).  do if ((ncol(condbb2) > 1) and (ncol(condbb)>1)).  compute condbb2t=make(nrow(condbb2),(ncol(condbb2)*ncol(condbb)),-999999).  compute k9=1.  do if (wfirst=1).  loop k7=1 to ncol(condbb2).  loop k8 = 1 to ncol(condbb).  compute condbb2t(:,k9)=condbb2(:,k7)&*condbb(:,k8).  compute k9=k9+1.  end loop.  end loop.  end if.  do if (zfirst=1).  loop k7=1 to ncol(condbb).  loop k8 = 1 to ncol(condbb2).  compute condbb2t(:,k9)=condbb(:,k7)&*condbb2(:,k8).  compute k9=k9+1.  end loop.  end loop.  end if.  compute condbb2=condbb2t.  release condbb2t.  end if.  do if ((ncol(condbb2) > 1) and (ncol(condbb)=1)).  loop k7 = 1 to ncol(condbb2).  compute condbb2(:,k7)=condbb2(:,k7)&*condbb.  end loop.  end if.  do if ((ncol(condbb2) = 1) and (ncol(condbb)>1)).  loop k7 = 1 to ncol(condbb).  compute condbb(:,k7)=condbb2&*condbb(:,k7).  end loop.  compute condbb2=condbb.  end if.  end if.  do if (ncol(imm2) <> 1 and ncol(imm) <> 1).  compute imm2t=make(nrow(imm2),(ncol(imm2)*ncol(imm)),-999999).  compute k9=1.  do if (wfirst=1).  loop k7=1 to ncol(imm2).  loop k8 = 1 to ncol(imm).  compute imm2t(:,k9)=imm2(:,k7)&*imm(:,k8).  compute k9=k9+1.  end loop.  end loop.  end if.  do if (zfirst=1).  loop k7=1 to ncol(imm).  loop k8 = 1 to ncol(imm2).  compute imm2t(:,k9)=imm(:,k7)&*imm2(:,k8).  compute k9=k9+1.  end loop.  end loop.  end if.  compute imm2=imm2t.  release imm2t.  end if.  do if ((ncol(imm2) > 1) and (ncol(imm)=1)).  loop k7=1 to ncol(imm2).  compute imm2(:,k7)=imm2(:,k7)&*imm.  end loop.  end if.  do if ((ncol(imm2) = 1) and (ncol(imm) > 1)).  loop k7=1 to ncol(imm).  compute imm(:,k7)=imm2&*imm(:,k7).  end loop.  compute imm2=imm.  end if.  end if.  do if (immset=0).  compute imm2=imm.  do if (indmmm(i,1)=41 or indmmm(i,1)=51).  compute condbb2=condbb.  end if.  compute immset=1.  end if.  end if.  end loop.  compute indtemp=tucker2(1,1).  do if (indmmm(i,1)=12 or indmmm(i,1)=22).  compute imm3={imm3,tucker2}.  do if (k1=nrow(indmodva)).  compute imm3=imm3(:,2:ncol(imm3)).  compute immstop=ncol(imm3).  loop k8=2 to immstop.  do if (indmmmt(i,1)=1).  compute imm3={imm3,(imm3(:,k8)-imm3(:,1))}.  end if.  do if (indmmmt(i,1)=2).  compute imm3={imm3,(imm3(:,k8)-imm3(:,(k8-1)))}.  end if.  do if (indmmmt(i,1)=3).  compute imm3={imm3,((rsum(imm3(:,(k8:immstop)))/(immstop-k8+1))-imm3(:,(k8-1)))}.  end if.  do if (indmmmt(i,1)=4).  compute imm3={imm3,(imm3(:,k8)-(rsum(imm3(:,1:immstop))/immstop))}.  end if.  end loop.  do if (indmmmt(i,1)<5).  compute imm2=imm3(:,(immstop+1):ncol(imm3)).  release imm3.  end if.  end if.  end if.  do if (indmmm(i,1)>-1 and (contrast = 1 or contrast = 2)).  compute imm4={imm4,tucker2}.  do if (k1=nrow(indmodva) and k1 > 1).  compute imm4=imm4(:,2:ncol(imm4)).  compute immstop=ncol(imm4).  compute condcont=make((immstop*(immstop-1)/2),6,-999).  loop k8 = 1 to (immstop-1).  loop k9 = (k8+1) to immstop.  do if (contrast=1).  compute imm4={imm4,(imm4(:,k9)-imm4(:,k8))}.  end if.  do if (contrast=2).  compute imm4={imm4,(abs(imm4(:,k9))-abs(imm4(:,k8)))}.  end if.  compute condcont((ncol(imm4)-immstop),1)=imm4(1,k9).  compute condcont((ncol(imm4)-immstop),2)=imm4(1,k8).  end loop.  end loop.  compute imm4=imm4(:,(immstop+1):ncol(imm4)).  loop k8=1 to ncol(imm4).  compute condcont(k8,3)=imm4(1,k8).  do if (boot > 0).  .  compute temp = imm4(2:nrow(imm4),k8).  compute temp(GRADE( imm4(2:nrow(imm4),k8) )) = imm4(2:nrow(imm4),k8).  compute badlo = 0.  compute badhi = 0.  do if ( 9999 <> 9999).  compute pv=csum(temp < 9999 )/nrow(temp).  compute ppv = pv.  do if (pv > .5).  compute ppv = 1-pv.  end if.  compute y5=sqrt(-2*ln(ppv)).  compute xp=y5+((((y5*p4+p3)*y5+p2)*y5+p1)*y5+p0)/((((y5*q4+q3)*y5+q2)*y5+q1)*y5+q0).  do if (pv <= .5).  compute xp = -xp.  end if.  compute cilow=rnd(nrow(temp)*(cdfnorm(2*xp+xp2))).  compute cihigh=trunc(nrow(temp)*(cdfnorm(2*xp+(-xp2))))+1.  do if (cilow < 1).  compute cilow = 1.  compute booterr=1.  compute badlo = 1.  end if.  do if (cihigh > nrow(temp)).  compute cihigh = boot.  compute booterr=1.  compute badhi = 1.  end if.  compute llcit=temp(cilow,1).  compute ulcit=temp(cihigh,1).  end if.  do if ( 9999 = 9999).  compute llcit=temp(cilow,1).  compute ulcit=temp(cihigh,1).  end if.  compute bootse=t(sqrt((cssq(temp)-((csum(temp)&**2)/nrow(temp)))/(nrow(temp)-1)))  .  compute condcont(k8,4:6)={bootse,llcit,ulcit}.  end if.  end loop.  do if (boot=0).  compute condcont=condcont(:,1:3).  end if.  compute indcontr=1.  end if.  end if.  do if (boot > 0).  .  compute temp = tucker2(2:nrow(tucker2),1).  compute temp(GRADE( tucker2(2:nrow(tucker2),1) )) = tucker2(2:nrow(tucker2),1).  compute badlo = 0.  compute badhi = 0.  do if ( 9999 <> 9999).  compute pv=csum(temp < 9999 )/nrow(temp).  compute ppv = pv.  do if (pv > .5).  compute ppv = 1-pv.  end if.  compute y5=sqrt(-2*ln(ppv)).  compute xp=y5+((((y5*p4+p3)*y5+p2)*y5+p1)*y5+p0)/((((y5*q4+q3)*y5+q2)*y5+q1)*y5+q0).  do if (pv <= .5).  compute xp = -xp.  end if.  compute cilow=rnd(nrow(temp)*(cdfnorm(2*xp+xp2))).  compute cihigh=trunc(nrow(temp)*(cdfnorm(2*xp+(-xp2))))+1.  do if (cilow < 1).  compute cilow = 1.  compute booterr=1.  compute badlo = 1.  end if.  do if (cihigh > nrow(temp)).  compute cihigh = boot.  compute booterr=1.  compute badhi = 1.  end if.  compute llcit=temp(cilow,1).  compute ulcit=temp(cihigh,1).  end if.  do if ( 9999 = 9999).  compute llcit=temp(cilow,1).  compute ulcit=temp(cihigh,1).  end if.  compute bootse=t(sqrt((cssq(temp)-((csum(temp)&**2)/nrow(temp)))/(nrow(temp)-1)))  .  compute indtemp={indtemp, bootse,llcit,ulcit}.  end if.  compute condres(k1,(ncol(indmodva)+1):ncol(condres))=indtemp.  end loop.  compute condlbs={condlbs,'Effect'}.  do if (boot > 0).  compute condlbs={condlbs,'BootSE', 'BootLLCI', 'BootULCI'}.  end if.  do if (nxvls=1).  print condres/title=' '/cnames=condlbs/format= F10.4 /space=0.  else.  compute condrlb=make(nrow(condres),1,xcatlab(k4,1)).  print condres/title=' '/cnames=condlbs/rnames=condrlb/format= F10.4 /space=0.  end if.  compute dichadj=0.  compute immcat=0.  do if (indmmm(i,1)>0).  do if (indmmm(i,1)=1 or indmmm(i,1)=12 or indmmm(i,1)=31).  do if (wdich=1 and mcw=0).  do if (indmmm(i,1) <> 12).  compute imm2(:,1)=imm2(:,1)*(wmax-wmin).  end if.  do if (indmmm(i,1) <> 31).  compute dichadj=1.  end if.  end if.  do if ((mcw = 1 or mcw = 2) and indmmm(i,1) <> 31)).  compute immcat=1.  end if.  end if.  do if (indmmm(i,1)=2 or indmmm(i,1)=22 or indmmm(i,1)=31).  do if (zdich=1 and mcz=0).  do if (indmmm(i,1) = 31).  compute imm2(:,(nwvls+1):ncol(imm2))=imm2(:,(nwvls+1):ncol(imm2))*(zmax-zmin).  end if.  do if (indmmm(i,1) = 2).  compute imm2(:,1)=imm2(:,1)*(zmax-zmin).  end if.  do if (indmmm(i,1) <> 31).  compute dichadj=1.  end if.  end if.  do if ((mcz = 1 or mcz = 2) and indmmm(i,1) <> 31)).  compute immcat=1.  end if.  end if.  compute immtemp2=t(imm2(1,:)).  compute immtemp=immtemp2.  compute immlbs={'Index'}.  do if (boot > 0).  compute immtemp=make(1,3,0).  loop k7=1 to ncol(imm2).  .  compute temp = imm2(2:nrow(imm2),k7).  compute temp(GRADE( imm2(2:nrow(imm2),k7) )) = imm2(2:nrow(imm2),k7).  compute badlo = 0.  compute badhi = 0.  do if ( 9999 <> 9999).  compute pv=csum(temp < 9999 )/nrow(temp).  compute ppv = pv.  do if (pv > .5).  compute ppv = 1-pv.  end if.  compute y5=sqrt(-2*ln(ppv)).  compute xp=y5+((((y5*p4+p3)*y5+p2)*y5+p1)*y5+p0)/((((y5*q4+q3)*y5+q2)*y5+q1)*y5+q0).  do if (pv <= .5).  compute xp = -xp.  end if.  compute cilow=rnd(nrow(temp)*(cdfnorm(2*xp+xp2))).  compute cihigh=trunc(nrow(temp)*(cdfnorm(2*xp+(-xp2))))+1.  do if (cilow < 1).  compute cilow = 1.  compute booterr=1.  compute badlo = 1.  end if.  do if (cihigh > nrow(temp)).  compute cihigh = boot.  compute booterr=1.  compute badhi = 1.  end if.  compute llcit=temp(cilow,1).  compute ulcit=temp(cihigh,1).  end if.  do if ( 9999 = 9999).  compute llcit=temp(cilow,1).  compute ulcit=temp(cihigh,1).  end if.  compute bootse=t(sqrt((cssq(temp)-((csum(temp)&**2)/nrow(temp)))/(nrow(temp)-1)))  .  compute temp={bootse,llcit,ulcit}.  compute immtemp={immtemp;temp}.  end loop.  compute immtemp=immtemp(2:nrow(immtemp),:).  compute immtemp={immtemp2,immtemp}.  compute immlbs={immlbs,'BootSE', 'BootLLCI', 'BootULCI'}.  end if.  do if (dichadj=0 and immcat=0 and indmmmt(i,1)<>5 and indmmm(i,1) < 100 ).  do if (indmmm(i,1) < 30).  print immtemp/title=' Index of moderated mediation:'/cnames=immlbs/rnames=immlbs2/format= F10.4.  end if.  do if (indmmm(i,1) = 31).  compute immlbs2={immlbs2;zcatlab(1:nzvls,1)}.  print immtemp/title=' Indices of partial moderated mediation:'/cnames=immlbs/rnames=immlbs2/format= F10.4.  end if.  do if ((nzvls = 1) and (nwvls = 1)).  do if (indmmm(i,1) = 41 or indmmm(i,1)=51).  loop k7=1 to nwvls.  compute immlbs2=zcatlab(1:nzvls,1).  compute immtemp2=immtemp((((k7-1)*nzvls)+1):(((k7-1)*nzvls)+nzvls),:).  do if (nwvls > 1).  compute primodv={' ', wcatlab(k7,1)}.  print primodv/title=' Primary moderator:'/format=A8.  end if.  do if (nzvls=1).  print immtemp2/title=' Index of moderated moderated mediation'/cnames=immlbs/format= F10.4.  else.  print immtemp2/title=' Indices of moderated moderated mediation'/cnames=immlbs/rnames=immlbs2/format= F10.4.  end if.  compute cmmtemp=make(nrow(zprobval),4,0).  loop k8=1 to nrow(zprobval).  compute condbb3=condbb2(:,((nwvls+1)+((k7-1)*nzvls)):((nwvls+1)+((k7-1)*nzvls)+ (nzvls-1))).  do if (ncol(zprobval) > 1).  compute condbb3=condbb3*mdiag(zprobval(k8,:)).  else.  compute condbb3=condbb3&*zprobval(k8,:).  end if.  compute condbb3={condbb2(:,k7),condbb3}.  compute icmm=rsum(condbb3).  compute cmmtemp(k8,1)=icmm(1,1).  do if (boot > 0).  .  compute temp = icmm(2:nrow(icmm),1).  compute temp(GRADE( icmm(2:nrow(icmm),1) )) = icmm(2:nrow(icmm),1).  compute badlo = 0.  compute badhi = 0.  do if ( 9999 <> 9999).  compute pv=csum(temp < 9999 )/nrow(temp).  compute ppv = pv.  do if (pv > .5).  compute ppv = 1-pv.  end if.  compute y5=sqrt(-2*ln(ppv)).  compute xp=y5+((((y5*p4+p3)*y5+p2)*y5+p1)*y5+p0)/((((y5*q4+q3)*y5+q2)*y5+q1)*y5+q0).  do if (pv <= .5).  compute xp = -xp.  end if.  compute cilow=rnd(nrow(temp)*(cdfnorm(2*xp+xp2))).  compute cihigh=trunc(nrow(temp)*(cdfnorm(2*xp+(-xp2))))+1.  do if (cilow < 1).  compute cilow = 1.  compute booterr=1.  compute badlo = 1.  end if.  do if (cihigh > nrow(temp)).  compute cihigh = boot.  compute booterr=1.  compute badhi = 1.  end if.  compute llcit=temp(cilow,1).  compute ulcit=temp(cihigh,1).  end if.  do if ( 9999 = 9999).  compute llcit=temp(cilow,1).  compute ulcit=temp(cihigh,1).  end if.  compute bootse=t(sqrt((cssq(temp)-((csum(temp)&**2)/nrow(temp)))/(nrow(temp)-1)))  .  compute cmmtemp(k8,2:4)={bootse,llcit,ulcit}.  end if.  end loop.  compute cmmtemp={zmodvals,cmmtemp}.  do if (boot=0).  compute cmmtemp=cmmtemp(:,1:2).  end if.  compute cmmlbs={znames,immlbs}.  print cmmtemp/title=' Indices of conditional moderated mediation by W'/cnames=cmmlbs/format= F10.4.  end loop.  end if.  end if.  end if.  do if (dichadj=1 or immcat=1 and indmmm(i,1) < 30).  print immtemp/title='Index of moderated mediation (difference between conditional indirect effects):'/cnames=immlbs/rnames=immlbs2/format= F10.4.  end if.  end if.  do if (indcontr=1).  compute condctlb={'Effect1','Effect2','Contrast','BootSE', 'BootLLCI','BootULCI'}.  print condcont/title=' Pairwise contrasts between conditional indirect effects (Effect1 minus Effect2)'/cnames=condctlb/format= F10.4.  end if.  print/title= '---'/space=0.  end loop.  end if.  end loop.  end if.  end if  .  do if (criterr=0 and (saveest = 1 or saveboot=1)).  compute coeffsav=coeffmat(2:nrow(coeffmat),:).  compute conseq=conseq(2:nrow(conseq),1).  compute dfmat=dfmat(2:nrow(dfmat),1).  compute dfmat=dfmat.  compute saven={'conseqnt','antecdnt','coeff','se','t','p','LLCI','ULCI','df'}.  do if (ydich=1).  compute saven={'conseqnt','antecdnt','coeff','se','t_or_Z','p','LLCI','ULCI','df'}.  end if.  do if (saveest=1).  save {conseq,vlabs,coeffsav,dfmat}/outfile = */names=saven/strings=conseqnt antecdnt.  end if.  do if (saveboot=1 and boot > 0 ).  print/title = '**************************************************************************'.  print/title='Bootstrap estimates were saved to a file'/space=0.  compute colslab=make(300,1,' ').  compute colslab( 1 ,1)= 'COL1'.  compute colslab( 2 ,1)= 'COL2'.  compute colslab( 3 ,1)= 'COL3'.  compute colslab( 4 ,1)= 'COL4'.  compute colslab( 5 ,1)= 'COL5'.  compute colslab( 6 ,1)= 'COL6'.  compute colslab( 7 ,1)= 'COL7'.  compute colslab( 8 ,1)= 'COL8'.  compute colslab( 9 ,1)= 'COL9'.  compute colslab( 10 ,1)= 'COL10'.  compute colslab( 11 ,1)= 'COL11'.  compute colslab( 12 ,1)= 'COL12'.  compute colslab( 13 ,1)= 'COL13'.  compute colslab( 14 ,1)= 'COL14'.  compute colslab( 15 ,1)= 'COL15'.  compute colslab( 16 ,1)= 'COL16'.  compute colslab( 17 ,1)= 'COL17'.  compute colslab( 18 ,1)= 'COL18'.  compute colslab( 19 ,1)= 'COL19'.  compute colslab( 20 ,1)= 'COL20'.  compute colslab( 21 ,1)= 'COL21'.  compute colslab( 22 ,1)= 'COL22'.  compute colslab( 23 ,1)= 'COL23'.  compute colslab( 24 ,1)= 'COL24'.  compute colslab( 25 ,1)= 'COL25'.  compute colslab( 26 ,1)= 'COL26'.  compute colslab( 27 ,1)= 'COL27'.  compute colslab( 28 ,1)= 'COL28'.  compute colslab( 29 ,1)= 'COL29'.  compute colslab( 30 ,1)= 'COL30'.  compute colslab( 31 ,1)= 'COL31'.  compute colslab( 32 ,1)= 'COL32'.  compute colslab( 33 ,1)= 'COL33'.  compute colslab( 34 ,1)= 'COL34'.  compute colslab( 35 ,1)= 'COL35'.  compute colslab( 36 ,1)= 'COL36'.  compute colslab( 37 ,1)= 'COL37'.  compute colslab( 38 ,1)= 'COL38'.  compute colslab( 39 ,1)= 'COL39'.  compute colslab( 40 ,1)= 'COL40'.  compute colslab( 41 ,1)= 'COL41'.  compute colslab( 42 ,1)= 'COL42'.  compute colslab( 43 ,1)= 'COL43'.  compute colslab( 44 ,1)= 'COL44'.  compute colslab( 45 ,1)= 'COL45'.  compute colslab( 46 ,1)= 'COL46'.  compute colslab( 47 ,1)= 'COL47'.  compute colslab( 48 ,1)= 'COL48'.  compute colslab( 49 ,1)= 'COL49'.  compute colslab( 50 ,1)= 'COL50'.  compute colslab( 51 ,1)= 'COL51'.  compute colslab( 52 ,1)= 'COL52'.  compute colslab( 53 ,1)= 'COL53'.  compute colslab( 54 ,1)= 'COL54'.  compute colslab( 55 ,1)= 'COL55'.  compute colslab( 56 ,1)= 'COL56'.  compute colslab( 57 ,1)= 'COL57'.  compute colslab( 58 ,1)= 'COL58'.  compute colslab( 59 ,1)= 'COL59'.  compute colslab( 60 ,1)= 'COL60'.  compute colslab( 61 ,1)= 'COL61'.  compute colslab( 62 ,1)= 'COL62'.  compute colslab( 63 ,1)= 'COL63'.  compute colslab( 64 ,1)= 'COL64'.  compute colslab( 65 ,1)= 'COL65'.  compute colslab( 66 ,1)= 'COL66'.  compute colslab( 67 ,1)= 'COL67'.  compute colslab( 68 ,1)= 'COL68'.  compute colslab( 69 ,1)= 'COL69'.  compute colslab( 70 ,1)= 'COL70'.  compute colslab( 71 ,1)= 'COL71'.  compute colslab( 72 ,1)= 'COL72'.  compute colslab( 73 ,1)= 'COL73'.  compute colslab( 74 ,1)= 'COL74'.  compute colslab( 75 ,1)= 'COL75'.  compute colslab( 76 ,1)= 'COL76'.  compute colslab( 77 ,1)= 'COL77'.  compute colslab( 78 ,1)= 'COL78'.  compute colslab( 79 ,1)= 'COL79'.  compute colslab( 80 ,1)= 'COL80'.  compute colslab( 81 ,1)= 'COL81'.  compute colslab( 82 ,1)= 'COL82'.  compute colslab( 83 ,1)= 'COL83'.  compute colslab( 84 ,1)= 'COL84'.  compute colslab( 85 ,1)= 'COL85'.  compute colslab( 86 ,1)= 'COL86'.  compute colslab( 87 ,1)= 'COL87'.  compute colslab( 88 ,1)= 'COL88'.  compute colslab( 89 ,1)= 'COL89'.  compute colslab( 90 ,1)= 'COL90'.  compute colslab( 91 ,1)= 'COL91'.  compute colslab( 92 ,1)= 'COL92'.  compute colslab( 93 ,1)= 'COL93'.  compute colslab( 94 ,1)= 'COL94'.  compute colslab( 95 ,1)= 'COL95'.  compute colslab( 96 ,1)= 'COL96'.  compute colslab( 97 ,1)= 'COL97'.  compute colslab( 98 ,1)= 'COL98'.  compute colslab( 99 ,1)= 'COL99'.  compute colslab( 100 ,1)= 'COL100'.  compute colslab( 101 ,1)= 'COL101'.  compute colslab( 102 ,1)= 'COL102'.  compute colslab( 103 ,1)= 'COL103'.  compute colslab( 104 ,1)= 'COL104'.  compute colslab( 105 ,1)= 'COL105'.  compute colslab( 106 ,1)= 'COL106'.  compute colslab( 107 ,1)= 'COL107'.  compute colslab( 108 ,1)= 'COL108'.  compute colslab( 109 ,1)= 'COL109'.  compute colslab( 110 ,1)= 'COL110'.  compute colslab( 111 ,1)= 'COL111'.  compute colslab( 112 ,1)= 'COL112'.  compute colslab( 113 ,1)= 'COL113'.  compute colslab( 114 ,1)= 'COL114'.  compute colslab( 115 ,1)= 'COL115'.  compute colslab( 116 ,1)= 'COL116'.  compute colslab( 117 ,1)= 'COL117'.  compute colslab( 118 ,1)= 'COL118'.  compute colslab( 119 ,1)= 'COL119'.  compute colslab( 120 ,1)= 'COL120'.  compute colslab( 121 ,1)= 'COL121'.  compute colslab( 122 ,1)= 'COL122'.  compute colslab( 123 ,1)= 'COL123'.  compute colslab( 124 ,1)= 'COL124'.  compute colslab( 125 ,1)= 'COL125'.  compute colslab( 126 ,1)= 'COL126'.  compute colslab( 127 ,1)= 'COL127'.  compute colslab( 128 ,1)= 'COL128'.  compute colslab( 129 ,1)= 'COL129'.  compute colslab( 130 ,1)= 'COL130'.  compute colslab( 131 ,1)= 'COL131'.  compute colslab( 132 ,1)= 'COL132'.  compute colslab( 133 ,1)= 'COL133'.  compute colslab( 134 ,1)= 'COL134'.  compute colslab( 135 ,1)= 'COL135'.  compute colslab( 136 ,1)= 'COL136'.  compute colslab( 137 ,1)= 'COL137'.  compute colslab( 138 ,1)= 'COL138'.  compute colslab( 139 ,1)= 'COL139'.  compute colslab( 140 ,1)= 'COL140'.  compute colslab( 141 ,1)= 'COL141'.  compute colslab( 142 ,1)= 'COL142'.  compute colslab( 143 ,1)= 'COL143'.  compute colslab( 144 ,1)= 'COL144'.  compute colslab( 145 ,1)= 'COL145'.  compute colslab( 146 ,1)= 'COL146'.  compute colslab( 147 ,1)= 'COL147'.  compute colslab( 148 ,1)= 'COL148'.  compute colslab( 149 ,1)= 'COL149'.  compute colslab( 150 ,1)= 'COL150'.  compute colslab( 151 ,1)= 'COL151'.  compute colslab( 152 ,1)= 'COL152'.  compute colslab( 153 ,1)= 'COL153'.  compute colslab( 154 ,1)= 'COL154'.  compute colslab( 155 ,1)= 'COL155'.  compute colslab( 156 ,1)= 'COL156'.  compute colslab( 157 ,1)= 'COL157'.  compute colslab( 158 ,1)= 'COL158'.  compute colslab( 159 ,1)= 'COL159'.  compute colslab( 160 ,1)= 'COL160'.  compute colslab( 161 ,1)= 'COL161'.  compute colslab( 162 ,1)= 'COL162'.  compute colslab( 163 ,1)= 'COL163'.  compute colslab( 164 ,1)= 'COL164'.  compute colslab( 165 ,1)= 'COL165'.  compute colslab( 166 ,1)= 'COL166'.  compute colslab( 167 ,1)= 'COL167'.  compute colslab( 168 ,1)= 'COL168'.  compute colslab( 169 ,1)= 'COL169'.  compute colslab( 170 ,1)= 'COL170'.  compute colslab( 171 ,1)= 'COL171'.  compute colslab( 172 ,1)= 'COL172'.  compute colslab( 173 ,1)= 'COL173'.  compute colslab( 174 ,1)= 'COL174'.  compute colslab( 175 ,1)= 'COL175'.  compute colslab( 176 ,1)= 'COL176'.  compute colslab( 177 ,1)= 'COL177'.  compute colslab( 178 ,1)= 'COL178'.  compute colslab( 179 ,1)= 'COL179'.  compute colslab( 180 ,1)= 'COL180'.  compute colslab( 181 ,1)= 'COL181'.  compute colslab( 182 ,1)= 'COL182'.  compute colslab( 183 ,1)= 'COL183'.  compute colslab( 184 ,1)= 'COL184'.  compute colslab( 185 ,1)= 'COL185'.  compute colslab( 186 ,1)= 'COL186'.  compute colslab( 187 ,1)= 'COL187'.  compute colslab( 188 ,1)= 'COL188'.  compute colslab( 189 ,1)= 'COL189'.  compute colslab( 190 ,1)= 'COL190'.  compute colslab( 191 ,1)= 'COL191'.  compute colslab( 192 ,1)= 'COL192'.  compute colslab( 193 ,1)= 'COL193'.  compute colslab( 194 ,1)= 'COL194'.  compute colslab( 195 ,1)= 'COL195'.  compute colslab( 196 ,1)= 'COL196'.  compute colslab( 197 ,1)= 'COL197'.  compute colslab( 198 ,1)= 'COL198'.  compute colslab( 199 ,1)= 'COL199'.  compute colslab( 200 ,1)= 'COL200'.  compute colslab( 201 ,1)= 'COL201'.  compute colslab( 202 ,1)= 'COL202'.  compute colslab( 203 ,1)= 'COL203'.  compute colslab( 204 ,1)= 'COL204'.  compute colslab( 205 ,1)= 'COL205'.  compute colslab( 206 ,1)= 'COL206'.  compute colslab( 207 ,1)= 'COL207'.  compute colslab( 208 ,1)= 'COL208'.  compute colslab( 209 ,1)= 'COL209'.  compute colslab( 210 ,1)= 'COL210'.  compute colslab( 211 ,1)= 'COL211'.  compute colslab( 212 ,1)= 'COL212'.  compute colslab( 213 ,1)= 'COL213'.  compute colslab( 214 ,1)= 'COL214'.  compute colslab( 215 ,1)= 'COL215'.  compute colslab( 216 ,1)= 'COL216'.  compute colslab( 217 ,1)= 'COL217'.  compute colslab( 218 ,1)= 'COL218'.  compute colslab( 219 ,1)= 'COL219'.  compute colslab( 220 ,1)= 'COL220'.  compute colslab( 221 ,1)= 'COL221'.  compute colslab( 222 ,1)= 'COL222'.  compute colslab( 223 ,1)= 'COL223'.  compute colslab( 224 ,1)= 'COL224'.  compute colslab( 225 ,1)= 'COL225'.  compute colslab( 226 ,1)= 'COL226'.  compute colslab( 227 ,1)= 'COL227'.  compute colslab( 228 ,1)= 'COL228'.  compute colslab( 229 ,1)= 'COL229'.  compute colslab( 230 ,1)= 'COL230'.  compute colslab( 231 ,1)= 'COL231'.  compute colslab( 232 ,1)= 'COL232'.  compute colslab( 233 ,1)= 'COL233'.  compute colslab( 234 ,1)= 'COL234'.  compute colslab( 235 ,1)= 'COL235'.  compute colslab( 236 ,1)= 'COL236'.  compute colslab( 237 ,1)= 'COL237'.  compute colslab( 238 ,1)= 'COL238'.  compute colslab( 239 ,1)= 'COL239'.  compute colslab( 240 ,1)= 'COL240'.  compute colslab( 241 ,1)= 'COL241'.  compute colslab( 242 ,1)= 'COL242'.  compute colslab( 243 ,1)= 'COL243'.  compute colslab( 244 ,1)= 'COL244'.  compute colslab( 245 ,1)= 'COL245'.  compute colslab( 246 ,1)= 'COL246'.  compute colslab( 247 ,1)= 'COL247'.  compute colslab( 248 ,1)= 'COL248'.  compute colslab( 249 ,1)= 'COL249'.  compute colslab( 250 ,1)= 'COL250'.  compute colslab( 251 ,1)= 'COL251'.  compute colslab( 252 ,1)= 'COL252'.  compute colslab( 253 ,1)= 'COL253'.  compute colslab( 254 ,1)= 'COL254'.  compute colslab( 255 ,1)= 'COL255'.  compute colslab( 256 ,1)= 'COL256'.  compute colslab( 257 ,1)= 'COL257'.  compute colslab( 258 ,1)= 'COL258'.  compute colslab( 259 ,1)= 'COL259'.  compute colslab( 260 ,1)= 'COL260'.  compute colslab( 261 ,1)= 'COL261'.  compute colslab( 262 ,1)= 'COL262'.  compute colslab( 263 ,1)= 'COL263'.  compute colslab( 264 ,1)= 'COL264'.  compute colslab( 265 ,1)= 'COL265'.  compute colslab( 266 ,1)= 'COL266'.  compute colslab( 267 ,1)= 'COL267'.  compute colslab( 268 ,1)= 'COL268'.  compute colslab( 269 ,1)= 'COL269'.  compute colslab( 270 ,1)= 'COL270'.  compute colslab( 271 ,1)= 'COL271'.  compute colslab( 272 ,1)= 'COL272'.  compute colslab( 273 ,1)= 'COL273'.  compute colslab( 274 ,1)= 'COL274'.  compute colslab( 275 ,1)= 'COL275'.  compute colslab( 276 ,1)= 'COL276'.  compute colslab( 277 ,1)= 'COL277'.  compute colslab( 278 ,1)= 'COL278'.  compute colslab( 279 ,1)= 'COL279'.  compute colslab( 280 ,1)= 'COL280'.  compute colslab( 281 ,1)= 'COL281'.  compute colslab( 282 ,1)= 'COL282'.  compute colslab( 283 ,1)= 'COL283'.  compute colslab( 284 ,1)= 'COL284'.  compute colslab( 285 ,1)= 'COL285'.  compute colslab( 286 ,1)= 'COL286'.  compute colslab( 287 ,1)= 'COL287'.  compute colslab( 288 ,1)= 'COL288'.  compute colslab( 289 ,1)= 'COL289'.  compute colslab( 290 ,1)= 'COL290'.  compute colslab( 291 ,1)= 'COL291'.  compute colslab( 292 ,1)= 'COL292'.  compute colslab( 293 ,1)= 'COL293'.  compute colslab( 294 ,1)= 'COL294'.  compute colslab( 295 ,1)= 'COL295'.  compute colslab( 296 ,1)= 'COL296'.  compute colslab( 297 ,1)= 'COL297'.  compute colslab( 298 ,1)= 'COL298'.  compute colslab( 299 ,1)= 'COL299'.  compute colslab( 300 ,1)= 'COL300'.  compute colslab=colslab(1:ncol(bootres),1).  compute colslab={colslab,conseq,vlabs}.  print colslab/title='Map of column names to model coefficients:'/clabels=' ','Conseqnt','Antecdnt'/format=a8.  end if.  end if.  do if (criterr=0 and boot > 0 and modelbt=1).  compute labstart=1.  print/title = '*********** BOOTSTRAP RESULTS FOR REGRESSION MODEL PARAMETERS ************'.  loop iboot = 1 to (nms+nys).  print outnames(1,iboot)/title = 'OUTCOME VARIABLE:'/format = A8.  compute vlabsm=vlabs(labstart:(labstart+(nump(1,iboot)-1)),1).  print bootcim(labstart:(labstart+(nump(1,iboot)-1)),:)/title=' '/rnames=vlabsm/ clabels='Coeff' 'BootMean' 'BootSE' 'BootLLCI' 'BootULCI'/format= F10.4 /space=0.  compute labstart=labstart+nump(1,iboot).  do if (iboot < (nms+nys)).  print/title= '----------'.  end if.  end loop.  end if.  do if (criterr = 0 and matrices=1).  print/title = '************************ MODEL DEFINITION MATRICES ************************'.  print/title = 'FROM variables are columns, TO variables are rows.'.  compute temp2=make(nrow(bcmat),ncol(bcmat),'0').  loop i = 2 to nrow(bcmat).  loop j = 1 to (ncol(bcmat)-1).  do if (bcmat(i,j)=1).  compute temp2(i,j)='1'.  end if.  do if (j >= i).  compute temp2(i,j)=' '.  end if.  end loop.  end loop.  compute temp2=temp2(2:nrow(bcmat),(1:(ncol(bcmat)-1))).  do if (nms > 0).  compute cmatlabs={xnames,mnames}.  compute rmatlabs={mnames,ynames}.  end if.  do if (nms = 0).  compute cmatlabs={xnames}.  compute rmatlabs={ynames}.  end if.  print temp2/title='BMATRIX: Paths freely estimated (1) and fixed to zero (0):'/cnames=cmatlabs/rnames=rmatlabs/format A3.  compute z=0.  do if (rsum(csum(wcmat))<>0).  compute temp2=make(nrow(wcmat),ncol(wcmat),'0').  loop i = 2 to nrow(wcmat).  loop j = 1 to (ncol(wcmat)-1).  do if (wcmat(i,j)=1).  compute temp2(i,j)='1'.  end if.  do if (j >= i).  compute temp2(i,j)=' '.  end if.  end loop.  end loop.  compute temp2=temp2(2:nrow(wcmat),(1:(ncol(wcmat)-1))).  print temp2/title='WMATRIX: Paths moderated (1) and not moderated (0) by W:'/cnames=cmatlabs/rnames=rmatlabs/format A3.  end if.  do if (rsum(csum(zcmat))<>0).  compute temp2=make(nrow(zcmat),ncol(zcmat),'0').  loop i = 2 to nrow(zcmat).  loop j = 1 to (ncol(zcmat)-1).  do if (zcmat(i,j)=1).  compute temp2(i,j)='1'.  end if.  do if (j >= i).  compute temp2(i,j)=' '.  end if.  end loop.  end loop.  compute temp2=temp2(2:nrow(zcmat),(1:(ncol(zcmat)-1))).  print temp2/title='ZMATRIX: Paths moderated (1) and not moderated (0) by Z:'/cnames=cmatlabs/rnames=rmatlabs/format a3.  end if.  do if (rsum(csum(wzcmat))<>0).  compute temp2=make(nrow(wzcmat),ncol(wzcmat),'0').  loop i = 2 to nrow(wzcmat).  loop j = 1 to (ncol(wzcmat)-1).  do if (wzcmat(i,j)=1).  compute temp2(i,j)='1'.  end if.  do if (j >= i).  compute temp2(i,j)=' '.  end if.  end loop.  end loop.  compute temp2=temp2(2:nrow(wzcmat),(1:(ncol(wzcmat)-1))).  print temp2/title='WZMATRIX: W moderated paths moderated (1) and not moderated (0) by Z:'/cnames=cmatlabs/rnames=rmatlabs/format a3.  end if.  do if (ncs > 0).  print ccmat/title='CMATRIX: Covariates (columns) in (1) and not in (0) the models of M and Y (rows):'/rnames=rmatlabs/cnames=covnames.  end if.  end if.  print/title = '*********************** ANALYSIS NOTES AND ERRORS ************************'.  do if (criterr=0).  print conf/title = 'Level of confidence for all confidence intervals in output:'/format = F8.4.  do if (boot > 0).  do if (goodboot = boot).  print boot/title='Number of bootstrap samples for percentile bootstrap confidence intervals:'.  end if.  end if.  do if (mc > 0).  print mc/title='Number of samples for Monte Carlo confidence intervals:'.  end if.  do if (wnotev > 0 and printw=1).  do if (wnotev=1).  print/title = 'W values in conditional tables are the 16th, 50th, and 84th percentiles.'.  else if (wnotev=2).  do if (minwwarn=0 and maxwwarn=0).  print/title = 'W values in conditional tables are the mean and +/- SD from the mean.'.  end if.  do if (minwwarn=1).  print/title = 'W values in conditional tables are the minimum, the mean, and 1 SD above the mean.'.  end if.  do if (maxwwarn=1).  print/title = 'W values in conditional tables are 1 SD below the mean, the mean, and the maximum.'.  end if.  end if.  end if.  do if (znotev > 0 and printz=1).  do if (znotev=1).  print/title = 'Z values in conditional tables are the 16th, 50th, and 84th percentiles.'.  else if (znotev=2).  do if (minzwarn=0 and maxzwarn=0).  print/title = 'Z values in conditional tables are the mean and +/- SD from the mean.'.  end if.  do if (minzwarn=1).  print/title = 'Z values in conditional tables are the minimum, the mean, and 1 SD above the mean.'.  end if.  do if (maxzwarn=1).  print/title = 'Z values in conditional tables are 1 SD below the mean, the mean, and the maximum.'.  end if.  end if.  end if.  do if (minwwarn > 0).  print/title = 'NOTE: One SD below the mean is below the minimum observed in the data for W,'.  print/title = ' so the minimum measurement on W is used for conditioning instead.'/space=0.  end if.  do if (maxwwarn > 0).  print/title = 'NOTE: One SD above the mean is above the maximum observed in the data for W,'.  print/title = ' so the maximum measurement for W is used for conditioning instead.'/space=0.  end if.  do if (minzwarn > 0).  print/title = 'NOTE: One SD below the mean is below the minimum observed in the data for Z,'.  print/title = ' so the minimum measurement for Z is used for conditioning instead.'/space=0.  end if.  do if (maxzwarn > 0).  print/title = 'NOTE: One SD above the mean is above the maximum observed in the data for Z,'.  print/title = ' so the maximum measurement for Z is used for conditioning instead.'/space=0.  end if.  do if (pstog=1).  print/title= 'NOTE: Standardized coefficients for dichotomous or multicategorical X are in'.  print/title= ' partially standardized form.'/space=0.  end if.  loop i = 1 to 100.  do if (notecode(i,1)=1).  print/title = 'NOTE: COVMY is ignored when using CMATRIX option.'.  end if.  do if (notecode(i,1)=2).  print/title = 'NOTE: Confidence level restricted to between 50 and 99.9999%.95% confidence is provided in output'.  end if.  do if (notecode(i,1)=3).  print centvar/title = 'NOTE: The following variables were mean centered prior to analysis:'/format = a8.  end if.  do if (notecode(i,1) = 4).  print/title = 'NOTE: A heteroscedasticity consistent standard error and covariance matrix estimator was used.'.  end if.  do if (notecode(i,1) = 5).  print/title = 'NOTE: The HC3 option has been replaced with HC.See the documentation.'.  end if.  do if (notecode(i,1) = 6).  print/title = 'NOTE: Due to estimation problems, some bootstrap samples had to be replaced.'.  print badboot/title=' The number of times this happened was:'/space=0/format=F8.0.  end if.  do if (notecode(i,1) = 7).  print/title = 'NOTE: The bootstrapping was not completed due to problematic bootstrap samples.'.  print/title = ' Bootstrap confidence intervals are therefore suppressed.'/space=0.  end if.  do if (notecode(i,1) = 8).  print/title = 'NOTE: The number of bootstrap samples was adjusted upward given your desired confidence.'.  end if.  do if (notecode(i,1) = 9).  print/title = 'NOTE: WMODVAL is ignored when W is specified as multicategorical.'.  end if.  do if (notecode(i,1) = 10).  print/title = 'NOTE: ZMODVAL is ignored when Z is specified as multicategorical.'.  end if.  do if (notecode(i,1) = 11).  print/title = 'NOTE: Total effect model and estimate generated only when all covariates are specified in all'.  print/title = ' models of M and Y.'/space=0.  end if.  do if (notecode(i,1) = 12).  print/title = 'NOTE: Total effect model and estimate generated only when X is freely estimated to affect each M'.  print/title = ' and both X and M are freely estimated to affect Y'/space=0.  end if.  do if (notecode(i,1) = 13).  print/title = 'NOTE: There are too many pairwise contrasts to conduct with this model.'.  end if.  do if (notecode(i,1) = 14).  print/title = 'NOTE: The number of contrast weights must equal the number of indirect effects.'.  end if.  do if (notecode(i,1) = 15).  print/title = 'NOTE: Monte Carlo confidence intervals not available for this model.'.  print/title = ' Bootstrapping is used instead.'/space=0.  end if.  do if (notecode(i,1) = 16).  print/title = 'NOTE: The number of Monte Carlo samples was adjusted upward given your desired confidence.'.  end if.  do if (notecode(i,1) = 19).  print/title = 'NOTE: Your contrast matrix is invalid or not applicable to this model.'.  end if.  do if (notecode(i,1) = 20).  print/title = 'NOTE: One of the groups specified by your contrast matrix does not exist in the data.'.  end if.  do if (notecode(i,1) = 21).  print/title = 'NOTE: The VARORDER option is not available in this release.'.  end if.  do if (notecode(i,1) = 22).  print/title = 'NOTE: The VMODVAL and QMODVAL options are not available in this release.'.  end if.  do if (notecode(i,1) = 23).  print/title = 'NOTE: The QUANTILE option is not available in this release.'.  end if.  do if (notecode(i,1) = 24).  print/title = 'NOTE: Total effect model not available with dichotomous Y'.  end if.  do if (notecode(i,1) = 25).  print/title = 'NOTE: Effect size option not available with dichotomous Y'.  end if.  do if ((notecode(i,1) = 26) and nms > 0).  print/title = 'NOTE: Direct and indirect effects of X on Y are on a log-odds metric.'.  end if.  do if (notecode(i,1) = 27).  print/title = 'NOTE: Standardized coefficients not available for models with moderators.'.  end if.  do if (notecode(i,1) = 28).  print/title = 'NOTE: The contrast option is not available with a multicategorical X.'.  end if.  end loop.  do if (toomany=1).  print/title='NOTE: Variables names longer than eight characters can produce incorrect output.'.  print/title=' Shorter variable names are recommended.'/space=0.  end if.  end if.  loop i = 1 to 100.  do if (errcode(i,1)=1).  print/title = 'ERROR: You must specify a Y and an X variable.'.  end if.  do if (errcode(i,1)=2).  print/title = 'ERROR: X, M, or Y variable used more than once or W and Z are the same variable.'.  do if (toomany = 1).  print/title = ' This could be caused by the use of variables names longer'/space=0.  print varnames/title = ' than eight characters.Here are the variables I see:'/space=0/format=A8.  end if.  end if.  do if (errcode(i,1)=3).  print/title = 'ERROR: You have specified more than one variable for W, Y, X, or Z'.  end if.  do if (errcode(i,1)=4).  print/title = 'ERROR: A variable specified as multicategorical has more than nine categories.'.  end if.  do if (errcode(i,1)=5).  print/title = 'ERROR: One of the categories contains only a single case.'.  end if.  do if (errcode(i,1)=6).  print/title = 'ERROR: Invalid model number in this version of PROCESS.'.  end if.  do if (errcode(i,1)=7).  print/title = 'ERROR: Invalid model number.'.  end if.  do if (errcode(i,1)=8).  print/title = 'ERROR: You must specify an M variable for this model.'.  end if.  do if (errcode(i,1)=9).  print/title = 'ERROR: You have specified an M variable in a model that does not use it.'.  print/title = 'In this release of PROCESS, moderators are W and Z in models 1, 2, and 3.'/space=0.  end if.  do if (errcode(i,1)=10).  print/title = 'ERROR: You have specified a W variable in a model that does not use it.'.  end if.  do if (errcode(i,1)=11).  print/title = 'ERROR: You have not specified a W variable in a model that requires it.'.  end if.  do if (errcode(i,1)=12).  print/title = 'ERROR: You have specified a Z variable in a model that does not use it.'.  end if.  do if (errcode(i,1)=13).  print/title = 'ERROR: You have not specified a Z variable in a model that requires it.'.  end if.  do if (errcode(i,1)=14).  print/title = 'ERROR: V and Q are not proper specifications in this release of PROCESS.'.  print/title = ' Moderators must be specified as W and/or Z.'/space=0.  end if.  do if (errcode(i,1)=15).  print/title = 'ERROR: One of your model variables exhibits no variation (it is a constant).'.  end if.  do if (errcode(i,1)=16).  print/title = 'ERROR: BMATRIX is not the correct length or is otherwise invalid.'.  end if.  do if (errcode(i,1)=17).  print/title = 'ERROR: WMATRIX is not the correct length or is otherwise invalid.'.  end if.  do if (errcode(i,1)=18).  print/title = 'ERROR: ZMATRIX is not the correct length or is otherwise invalid.'.  end if.  do if (errcode(i,1)=19).  print/title = 'ERROR: WZMATRIX is not the correct length or is otherwise invalid.'.  end if.  do if (errcode(i,1)=20).  print/title = 'ERROR: A path fixed at zero cannot be moderated.'.  end if.  do if (errcode(i,1)=21).  print/title = 'ERROR: If only one moderator is specified, it must be specified as W.'.  end if.  do if (errcode(i,1)=22).  print/title = 'ERROR: In BMATRIX, X must be specified to affect at least one variable.'.  end if.  do if (errcode(i,1)=23).  print/title = 'ERROR: In BMATRIX, at least one variable must be specified to affect Y.'.  end if.  do if (errcode(i,1)=24).  print/title = 'ERROR: You must specify a model number or a custom BMATRIX specification.'.  end if.  do if (errcode(i,1)=25).  print/title = 'ERROR: BMATRIX cannot be used in conjunction with a model number.'.  end if.  do if (errcode(i,1)=26).  print/title = 'ERROR: Your model has a dangling mediator (all Ms must affect and be affected).'.  end if.  do if (errcode(i,1)=27).  print/title = 'ERROR: CLUSTER is not available on this release of PROCESS.'.  end if.  do if (errcode(i,1)=29).  print/title = 'ERROR: CMATRIX is not the correct length or is otherwise invalid.'.  end if.  do if (errcode(i,1)=30).  print/title = 'ERROR: In CMATRIX, all covariates must be assigned to an M or a Y.'.  end if.  do if (errcode(i,1)=31).  print/title = 'ERROR: A linear or near linear dependency (singularity) exists in the data.'.  end if.  do if (errcode(i,1)=32).  print/title = 'ERROR: Models 80 and 81 require between 3 and 6 mediators.'.  end if.  do if (errcode(i,1)=33).  print/title = 'ERROR: Model 82 requires 4 mediators.'.  end if.  do if (errcode(i,1)=34).  print/title = 'ERROR: This model number requires between 2 and 6 mediators.'.  end if.  do if (errcode(i,1)=35).  print/title = 'ERROR: In a model with only one moderator, that moderator must be W.'.  end if.  do if (errcode(i,1)=36).  print/title = 'ERROR: A serial mediation model cannot have more than 6 mediators.'.  end if.  do if (errcode(i,1)=37).  print/title = 'ERROR: No more than 10 mediators are allowed in a PROCESS command.'.  end if.  do if (errcode(i,1)=38).  print/title = 'ERROR: XCATCODE is not provided, not the correct length, or is otherwise invalid.'.  end if.  do if (errcode(i,1)=39).  print/title = 'ERROR: WCATCODE is not provided, not the correct length, or is otherwise invalid.'.  end if.  do if (errcode(i,1)=40).  print/title = 'ERROR: ZCATCODE is not provided, not the correct length, or is otherwise invalid.'.  end if.  do if (errcode(i,1)=41).  print/title = 'ERROR: Models 1, 2, 3, and 74 cannot be customized.'.  end if.  do if (errcode(i,1)=42).  print/title = 'ERROR: WS option available only in PROCESS v2. Or use the MEMORE macro instead.'.  print/title = ' MEMORE can be downloaded from www.akmontoya.com.'/space=0.  end if.  do if (errcode(i,1)=43).  print/title = 'ERROR: PROCESS does not allow dichotomous mediators.'.  end if.  do if (errcode(i,1)=45).  print/title = 'ERROR: In model 74, X and W must be the same variable.'.  end if.  do if (errcode(i,1)=46).  print/title = 'ERROR: Model 74 is temporarily disabled in this release of PROCESS.'.  end if.  do if (errcode(i,1)=50).  print/title = 'ERROR: A multicategorical moderator cannot be specified as a covariate.'.  end if.  do if (errcode(i,1)=51).  print/title = 'ERROR: A variable you specified as a covariate is a moderator in all equations.'.  end if.  do if (errcode(i,1)=52 and mcerpt=0).  compute mcerpt=1.  print/title = 'ERROR: A variable specified as multicategorical must have at least three categories'.  end if.  do if (errcode(i,1) = 47).  do if (iterrmod=1).  print/title = 'ERROR: Iteration for Y model didn''t converge to a solution.Interpret results with caution.'.  print/title = ' Try increasing the number of iterations, though this will slow down computation.'/space=0.  end if.  do if (bootiter=1).  print/title = 'ERROR: Nonconvergence during bootstrapping.Interpet bootstrap results with caution.'.  end if.  end if.  end loop.  END MATRIX. |
| resource | Processor time | 00 00:00:12.672 |
|  | Time used | 00 00:00:13.077 |

[Dataset 1] F: \ submitted version \ data.sav

Run MATRIX procedure:

**************** PROCESS Procedure for SPSS Version 3.4.1 ****************

Written by Andrew F.Hayes, Ph.D.www.afhayes.com

Documentation available in Hayes (2018).www.guilford.com/p/hayes3

**************************************************************************

Model : 4

Y: Z Mental health

X: Z stress perception

M: Z job burnout

Sample

Size: 792

**************************************************************************

OUTCOME VARIABLE:

Z job burnout

Model Summary

R	R-sq	MSE	F	df1	df2	p

.6986	.4880	.5126	753.1001	1.0000	790.0000	.0000

Model

coeff	se	t	p	LLCI	ULCI

constant	.0000	.0254	.0000	1.0000	-.0499	.0499

Z stress perception.6986	.0255	27.4427	.0000	.6486	.7486

Standardized coefficients

coeff

Z stress perception.6986

**************************************************************************

OUTCOME VARIABLE:

Z mental health

Model Summary

R	R-sq	MSE	F	df1	df2	p

.6129	.3756	.6260	237.3291	2.0000	789.0000	.0000

Model

coeff	se	t	p	LLCI	ULCI

constant	.0000	.0281	.0000	1.0000	-.0552	.0552

Z stress perception.3975	.0393	10.1112	.0000	.3204	.4747

Z job burnout.2652	.0393	6.7444	.0000	.1880	.3423

Standardized coefficients

coeff

Z stress perception.3975

Z job burnout.2652

************************** TOTAL EFFECT MODEL ****************************

OUTCOME VARIABLE:

Z mental health

Model Summary

R	R-sq	MSE	F	df1	df2	p

.5828	.3396	.6612	406.2912	1.0000	790.0000	.0000

Model

coeff	se	t	p	LLCI	ULCI

constant	.0000	.0289	.0000	1.0000	-.0567	.0567

Z stress perception.5828	.0289	20.1567	.0000	.5260	.6395

Standardized coefficients

coeff

Z stress perception.5828

************** TOTAL, DIRECT, AND INDIRECT EFFECTS OF X ON Y **************

Total effect of X on Y

Effect	se	t	p	LLCI	ULCI	c_ps	c_cs

.5828	.0289	20.1567	.0000	.5260	.6395	.5828	.5828

Direct effect of X on Y

Effect	se	t	p	LLCI	ULCI	c'_ps	c'_cs

.3975	.0393	10.1112	.0000	.3204	.4747	.3975	.3975

Indirect effect(s) of X on Y:

Effect	BootSE	BootLLCI	BootULCI

Z: job burnout.1852	.0334	.1199	.2507

Partially standardized indirect effect(s) of X on Y:

Effect	BootSE	BootLLCI	BootULCI

Z: job burnout.1852	.0319	.1218	.2470

Completely standardized indirect effect(s) of X on Y:

Effect	BootSE	BootLLCI	BootULCI

Z: job burnout.1852	.0318	.1224	.2478

*********** BOOTSTRAP RESULTS FOR REGRESSION MODEL PARAMETERS ************

OUTCOME VARIABLE:

Z job burnout

Coeff	BootMean	BootSE	BootLLCI	BootULCI

constant	.0000	-.0002	.0249	-.0492	.0484

Z stress perception.6986	.6986	.0269	.6466	.7499

----------

OUTCOME VARIABLE:

Z mental health

Coeff	BootMean	BootSE	BootLLCI	BootULCI

constant	.0000	-.0001	.0282	-.0546	.0554

Z stress perceptionn.3975	.3972	.0400	.3204	.4748

Z job burnout.2652	.2652	.0454	.1753	.3526

*********************** ANALYSIS NOTES AND ERRORS ************************

Level of confidence for all confidence intervals in output:

95.0000

Number of bootstrap samples for percentile bootstrap confidence intervals:

5000

NOTE: Variables names longer than eight characters can produce incorrect output.

Shorter variable names are recommended.

------ END MATRIX -----
