# Supplementary material for: The influence of perceived stress of Chinese healthcare workers after the opening of COVID-19: the bidirectional mediation between mental health and job burnout
Source: Front Public Health. 2023 Aug 17;11:1252103. doi: 10.3389/fpubh.2023.1252103 (PMC10470117; doi:10.3389/fpubh.2023.1252103)
Supplement: Supplementary file 2 [file Data_Sheet_2.docx]

Dear employees:

In order to timely understand and evaluate the mental health status of medical staff after the epidemic, investigate and analyze various factors affecting mental health, and better provide psychological support and psychological services for healthcare workers, this questionnaire is designed. This questionnaire will not disclose personal information, the questionnaire takes about 5-8 minutes, please feel free to fill in.

Wuxi Maternal and Child Health Care Hospital psychology Department

In January, 2023

**1. general information**

1. Name:

2. Work number:

3. Department:

4. Age:

5. Sex:

male

female

6. Marital status:

married

unmarried

7. Educational level:

College and below

Bachelor's degree

Master's degree

Doctor's degree

8. Job title:

College and below

Bachelor's degree

Master's degree

Doctor's degree

9. Employment:

Permanent

Contract

10. Monthly salary level:

＜3000

3000-6000

6000-10000

＞10000

11. Whether they have been frontline anti-epidemic workers (staff in the fever room, COVID-19 nucleic acid sampling, emergency department, pre-test and triage, nucleic acid testing, isolation ward, etc.)

yes

No

**2. The World Health Organization Mental Health Self-Assessment Questionnaire**

The following questions are related to certain pain and problems that may have plagued you in the past 30 days. If you feel the question fits your situation and exists within the past 30 days, please answer Yes. On the other hand, if the question is not appropriate for your situation or does not exist in the past 30 days, please answer no.. Please do not discuss with anyone when answering the questionnaire. If you are not sure how to answer the question, please try to give what you think is the most appropriate answer.

1. Do you often have a headache?

yes

no

2. Do you have a poor appetite?

yes

no

3. Do you have a poor sleep?

yes

no

4. Are you easily frightened?

yes

no

5. Are your hands shaking?

yes

no

6. Do you feel uneasy, nervous, or worried?

yes

no

7. Do you experience any symptoms of indigestion?

Yes

No

1. Do you have any trouble with cognitive function?
2. yes

no

9. Do you feel unhappy?

yes

no

10.Do you cry more than before?

yes

no

11. Do you find it difficult to have fun with your daily activities?

yes

no

12. Do you find it hard for yourself to make a decision?

yes

no

13. Do you feel miserable from your daily work and study?

yes

no

14. Do you not play a proper role in your daily life?

yes

no

15. Have you lost your interest in things?

yes

no

16. Do you feel like a worthless person?

yes

no

17. Have you ever ended your life in your mind?

yes

no

18.Do you feel tired at any time?

yes

no

19. Do you feel an upset stomach?

yes

no

20. Are you getting tired easily?

yes

no

**3. Job Burnout scale**

Please according to their own feelings and experience, judge them in your unit or your frequency, and on the right number, each item has the following seven options: options from 0 to 6 said event frequency gradually increased, in turn is: never, rarely (a few times a year or less), occasionally (once a month or less), often (a few times for a month), frequent (once a week), very frequent (several times a week), every day.

(1)emotional exhaustion (Score for this dimension = total scores for all questions / 5)

1. Work makes me feel physically exhausted 0 1 2 3 4 5 6

2. I feel exhausted when I leave work 0 1 2 3 4 5 6

3. I feel very tired when I get up in the morning and have to face the day's work 0 1 2 3 4 5 6

4.Working all day long is really stressful for me 0 1 2 3 4 5 6

5. Work makes me feel devastated 0 1 2 3 4 5 6

(2)depersonalization (Score for this dimension = total scores for all questions / 4)

6. Since I started this job, I have been less and less interested in it 0 1 2 3 4 5 6

7. I am not as enthusiastic about my work as before 0 1 2 3 4 5 6

8. I doubt the significance of what I do 0 1 2 3 4 5 6

9. I care less and less about whether I contribute to my work 0 1 2 3 4 5 6

(3)low personal accomplishment (Score for this dimension = total scores for all questions / 6)

10. I can effectively solve the problems in my work (reverse scoring) 0 1 2 3 4 5 6

11. I feel like I am making a useful contribution to the unit (reverse scoring) 0 1 2 3 4 5 6

12. In my opinion, I am good at my own work (reverse scoring) 0 1 2 3 4 5 6

13. I feel very happy to do something at work (reverse score) 0 1 2 3 4 5 6

14. I have done a lot of valuable work (reverse scoring) 0 1 2 3 4 5 6

15. I am confident that I can complete all the work effectively (reverse score) 0 1 2 3 4 5 6

**4. Stress perception scale**

In the last month, your personal feelings and thoughts, please point out how often you feel or think of a specific idea when answering each item. Although some questions look similar, they are actually different, so each question needs to be answered. Try to answer in a quick and unthinking way, that is, do not think about the meaning behind the score of each question, in order to really reflect your real stress perception situation. Each item has the following five options: options from top to bottom indicates that the event frequency gradually increases, in order: never — occasionally — sometimes — often — always.

Remember the frequency of the following conditions in the last month.

1. Be upset by something unexpected happening

never

once in a while

sometimes

often

always

2. Feel like you can't control the important things in your life

never

once in a while

sometimes

often

always

3. Feel nervous and stressed

never

once in a while

sometimes

often

always

4. Successfully deal with the annoying life troubles

never

once in a while

sometimes

often

always

5. Feel effective in handling the important changes that occur in your life

never

once in a while

sometimes

often

always

6. Feel confident about the ability to deal with your own personal problems

never

once in a while

sometimes

often

always

7. Feel things going well

never

once in a while

sometimes

often

always

8. Find out that you can't handle everything you have to do

never

once in a while

sometimes

often

always

9. There are ways to control the annoying things in your life

never

once in a while

sometimes

often

always

10. Often feel like the master of things

never

once in a while

sometimes

often

always

11. Often angry, because many things happen beyond your control

never

once in a while

sometimes

often

always

12 often think of some things you must do

never

once in a while

sometimes

often

always

13. Always master the schedule

never

once in a while

sometimes

often

always

14. Difficult things are hard to overcome them

never

once in a while

sometimes

often

always
